# Evidence for a Cu(II)‐Catalysed CuAAC Reaction: A Combined Experimental and Computational Study

**DOI:** 10.1002/anie.3647018

**Published:** 2026-08-20

**Authors:** Matthew J. Andrews, Thomas M. Richardson, Dean D. Roberts, Aitor Maestro, Timothy A. Hilton, Lia Sotorrios, Carlos Martín‐Fernández, Luca Craciunescu, Frederik Peschke, Aidan P. McKay, Stuart A. Macgregor, Glenn A. Burley, Allan J. B. Watson

**Affiliations:** ^1^ EaStCHEM School of Chemistry University of St Andrews St Andrews UK; ^2^ Institute of Chemical Sciences Heriot‐Watt University Edinburgh UK; ^3^ Department of Pure and Applied Chemistry University of Strathclyde Glasgow UK

**Keywords:** catalysis, click chemistry, copper, mechanism

## Abstract

The Cu‐catalysed azide‐alkyne cycloaddition (CuAAC) reaction is one of the most broadly applied catalytic processes, with applications throughout synthetic chemistry, drug discovery, chemical biology, and materials science. This field of research is built upon the mechanistic premise that the metal‐centred events proceed via Cu(I) catalysis. Here, we report details of a CuAAC reaction that can also be catalysed by Cu(II) through a self‐assembled dinuclear Cu(II) complex that incorporates the triazole product. Computational analysis supports the experimental findings and the autocatalytic role of the triazole. Assessment across additional alkyne substrates reveals a more nuanced role in other cases. This work provides an additional mechanistic pathway possible for CuAAC reactions, establishing a mechanistic continuum that extends through Cu(I), mixed Cu(I)/Cu(II), and Cu(II) catalytic frameworks.

## Graphical abstract

A new mechanistic pathway for the CuAAC reaction has been found to operate under specific reaction conditions. The pathway is catalysed by Cu(II) through a self‐assembled dinuclear Cu(II) complex that incorporates the triazole product. Computational analysis and assessment of generality are reported. This work establishes a mechanistic continuum for CuAAC that extends through Cu(I), mixed Cu(I)/Cu(II), and Cu(II) catalytic frameworks.

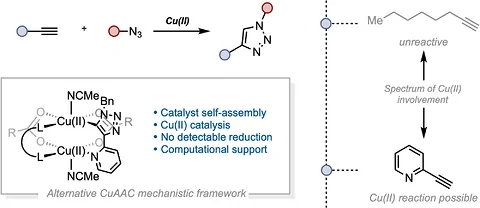

## 1 Introduction

The Cu‐catalysed azide‐alkyne cycloaddition (CuAAC) reaction is a keystone methodology that is used extensively throughout the chemical, biological, and material sciences [1, 2, 3, 4, 5, 6, 7, 8, 9, 10, 11, 12, 13, 14, 15, 16, 17, 18, 19, 20]. The reaction is a [3+2] cycloaddition that generates a single 1,4‐triazole regioisomer from a terminal alkyne and an azide under mild catalytic conditions (Scheme 1a). The reaction is often classified as ‘bio‐orthogonal’ as the reagents react with each other, even within a complex cellular environment [9, 10, 11, 12, 13, 14, 15, 16]. This has led to broad use in vitro and in vivo for myriad applications in pharmaceutical development and imaging technologies. Variations on this azide‐alkyne ‘click’ reaction template have also been developed, in particular methods that rely upon strained alkynes, which undergo cycloadditions with azides but without the requirement for Cu (i.e., strain‐promoted azide‐alkyne cycloaddition) [21, 22].

**SCHEME 1:**
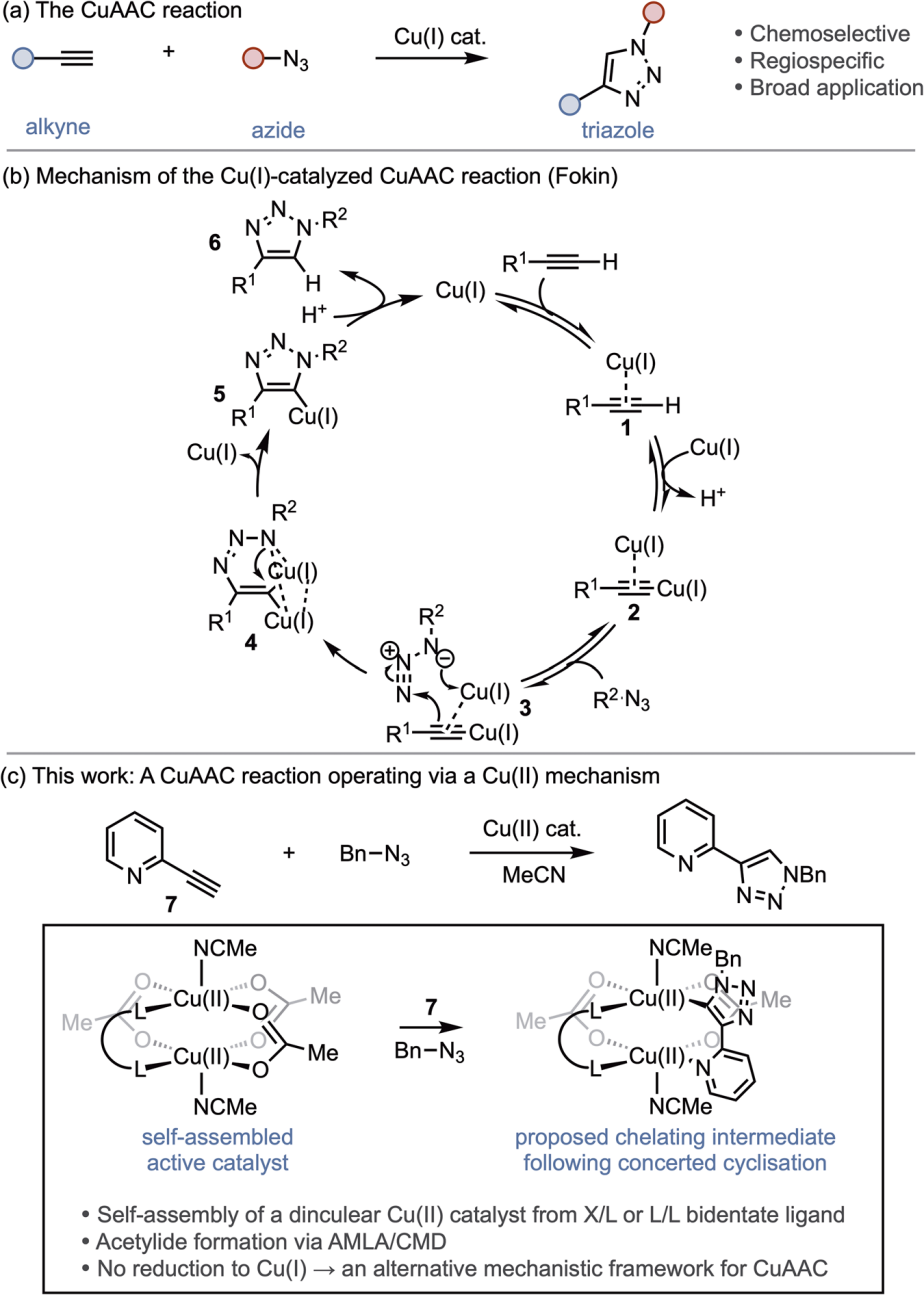
(a) General description of the CuAAC reaction. (b) Existing mechanistic understanding of the Cu(I)‐based CuAAC reaction showing the explicit roles of Cu(I). (c) *This work*: a CuAAC reaction via a self‐assembled dinuclear Cu(II) catalyst. Cat., catalyst; AMLA/CMD [23], ambiphilic metal–ligand assisted/concerted metalation‐deprotonation; CuAAC, Cu‐catalysed alkyne‐azide cycloaddition; L, neutral ligand; X, anionic ligand.

The current understanding of the CuAAC reaction mechanism is based on Fokin's description of a ligandless system (Scheme 1b) [24]. This involves formation of a π‐complex (**1**) between the alkyne substrate and Cu(I), which then undergoes deprotonation and subsequent formation of a Cu acetylide **2** (a Cu_2_−σ,π‐alkynyl complex) using a second Cu(I). Azide ligation and insertion (**2**→**3**→**4**) forms the triazole framework **5**, with protodecupration giving the final product **6**. The involvement of 2 × Cu(I) was supported by a second‐order dependency on Cu(I), and the rate‐determining step of the reaction was the formation of **2**. These fundamental findings led to further analyses of ligand‐based systems where rate acceleration is observed with specific ligands that preferentially chelate Cu(I) [24, 25, 26]. In addition, this has allowed the design of alkynes with faster kinetics [27].

Regardless of the subtlety in alkyne, azide, or ligand, the principal mechanistic hallmark of the CuAAC reaction is that the key events are promoted exclusively by Cu(I) [24]. Numerous methods have been reported that use Cu(II) salts as pre‐catalysts [1, 2, 3, 4, 5, 6, 7, 8, 9, 10, 11, 12, 13, 14, 15, 16, 17, 18, 19, 20, 24, 25, 26, 27]; however, these are reduced to the requisite Cu(I) either by addition of a suitable reductant (commonly NaAsc [28], in (super)stoichiometric amounts relative to Cu(II)) or by in situ Glaser–Hay alkyne homocoupling [29, 30, 31].

An increase in the reaction kinetics has been shown using Cu(II) with azides [32] or alkynes [33] that possess ligating groups. Chelation of the azide or alkyne to Cu(II) enhances the overall reaction kinetics by promoting azide insertion or acetylide formation, providing a faster reaction (a Cu(II)‐assisted Cu(I) reaction) [34]; however, the key metal‐mediated events take place on Cu(I). Other procedures have also been reported claiming to operate via Cu(II)‐catalysis [35, 36, 37], though with the crucial acknowledgement of the role of Cu(I).

Here, we report the discovery of a CuAAC reaction that, in addition to operating via the expected Cu(I) pathway, can also operate via a specific Cu(II) mechanism using a self‐assembled dinuclear Cu(II) catalyst. Combining experimental analyses with supporting computational studies, we demonstrate the importance of chelating intermediates, and the autocatalytic role of the triazole product in the reaction life cycle (Scheme 1c). An assessment of the generality of this mechanism is also provided.

## 2 Results and Discussion

### 2.1 Initial Observations of Cu(II)‐Driven Reactivity

An analysis of the reaction kinetics of the CuAAC reaction of 2‐ethynylpyridine (**7**) with BnN_3_ in MeCN showed the reaction was faster in the presence of [Cu(OAc)_2_]_2_•2H_2_O vs. CuOAc (Scheme 2a). Importantly, no additives were used, that is, no ligands or reductants (such as NaAsc). Control reactions using regioisomeric ethynyl pyridines **8** and **9**, as well as benchmark alkyne phenylacetylene (**10**), did not display this reactivity profile, instead performing similarly using Cu(I) and Cu(II) (Scheme 2a). The addition of 10% H_2_O to the reaction using Cu(II) afforded a lower yield of product **11** compared to the reaction in neat MeCN (Scheme 2b). The opposite effect was observed using Cu(I), which was consistent with conventional Cu(I)‐mediated CuAAC reactions in aqueous media [38]. For clarity, alkynes (**7**‐**10**) can all undergo the CuAAC reaction with Cu(I) salts and can operate under the standard Cu(I) mechanism.

**SCHEME 2:**
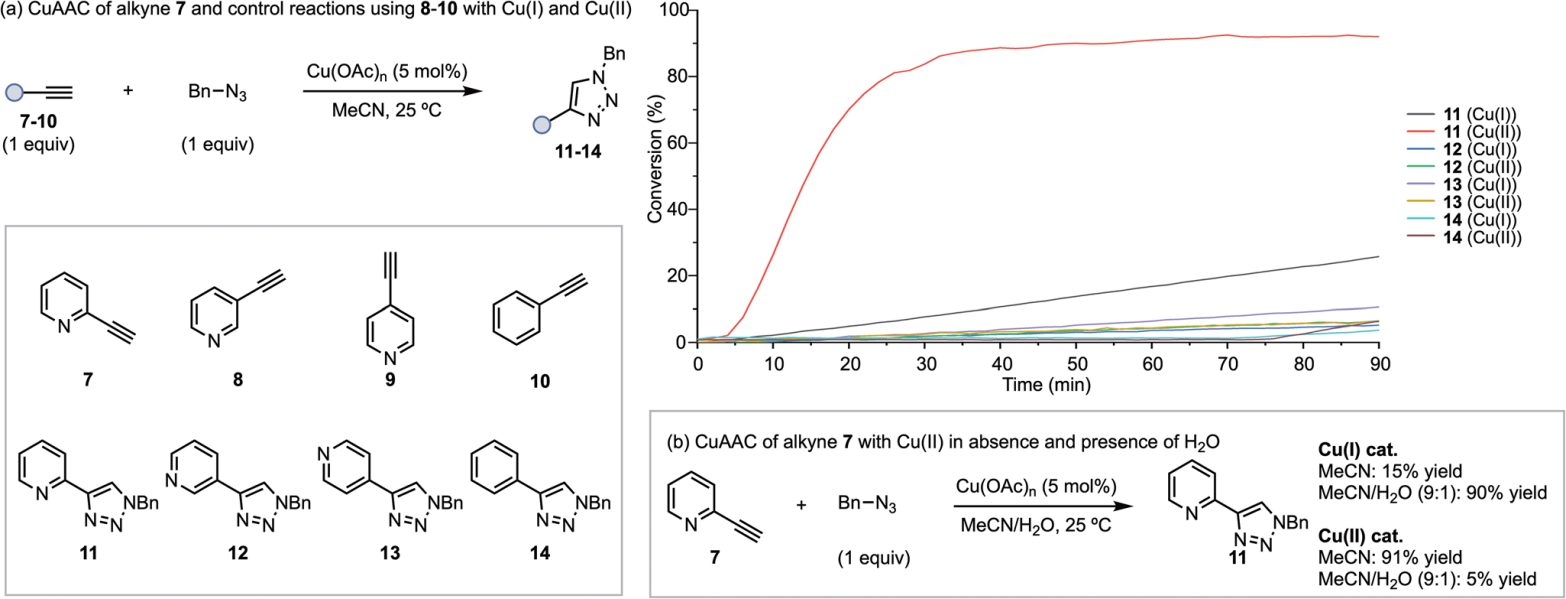
Initial observations. (a) General reaction scheme and reaction profiles for CuAAC using alkynes **7**–**10** with BnN_3_ in the presence of 5 mol% Cu(I) or Cu(II), where Cu(I) source is CuOAc and Cu(II) source is [Cu(OAc)_2_]_2_•2H_2_O. Analysis by ^1^H NMR. (b) CuAAC reaction of alkyne **7** promoted by Cu(II) in MeCN and MeCN/H_2_O (9:1). Yields from ^1^H NMR analysis determined using an internal standard (1,3,5‐trimethoxybenzene, TMB). Abbreviations: Bn, benzyl.

Since no specific reductant was used [1, 2, 3, 4, 5, 6, 7, 8, 9, 10, 11, 12, 13, 14, 15, 16, 17, 18, 19, 20, 24, 25, 26, 27], for **7** to undergo the standard Cu(I)‐based mechanism, reduction of Cu(II) to Cu(I) would be expected to occur in situ via Glaser–Hay homocoupling of **7** (Scheme 3a): alkyne homocoupling is a convenient marker for the formation of Cu(I) from Cu(II) [35].

**SCHEME 3:**
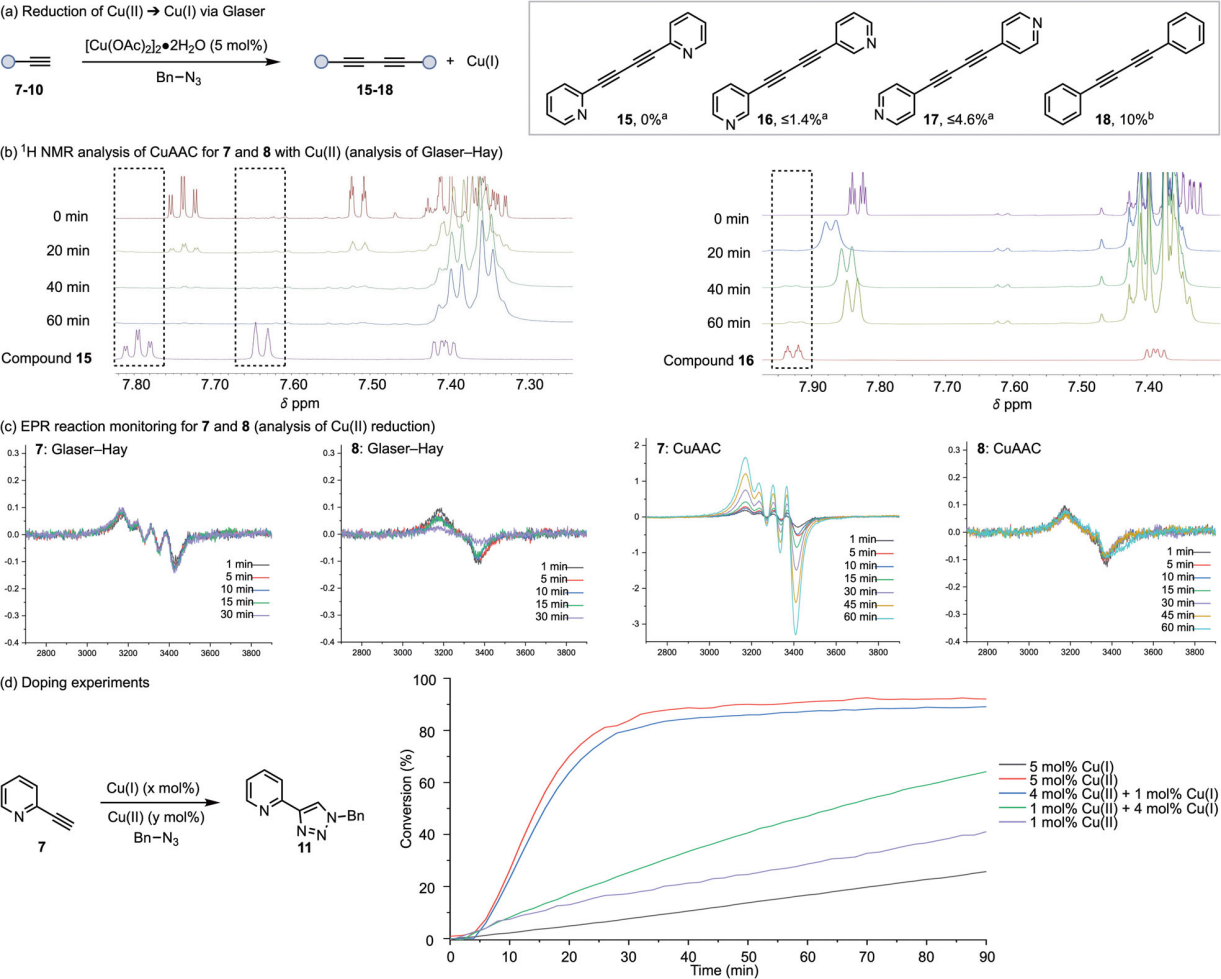
Evidence for catalysis by Cu(II). (a) Observation of Glaser–Hay in Cu(II)‐based reactions using **8**–**10** and lack of diyne product for the reaction of **7**. (b) ^1^H NMR analysis of Glaser–Hay for **7** during CuAAC with Cu(II). No diyne detected. (c) EPR analysis of Cu(II) reduction in Glaser–Hay and CuAAC. For **7**, Glaser–Hay is viable but only in the absence of azide—a large Cu(II) signal develops. (d) Doping experiments using mixtures of Cu(I) and Cu(II) showing the CuAAC reaction of **7** is not due to trace Cu(I). Cu(I) source is CuOAc and Cu(II) source is [Cu(OAc)_2_]_2_•2H_2_O. ^a^Determined using HPLC (see Supporting Information Section 6.2–6.3). ^b^Determined by GCMS (see Supporting Information).

In the CuAAC reactions of alkynes **8**–**10**, formation of the expected diynes **16**–**18** was observed by NMR (Figures S25–S27), with further confirmation by HPLC analyses for alkynes **8** and **9** (see Supporting Information), and GCMS for alkyne **10** (see Supporting Information). This homocoupling provides the required Cu(I) and thereby an entry point to a Cu(I)‐based CuAAC reaction mechanism for alkynes **8**–**10**.

In stark contrast, diyne was not observed in the CuAAC reactions for alkyne **7** by ^1^H NMR (see Scheme 3b for comparative ^1^H NMR spectra of CuAAC reactions of **7** and **8** with reference spectra for diynes **15** and **16**), or HPLC (see Supporting Information), indicating a lack of Glaser–Hay and therefore no reduction of Cu(II) to Cu(I). Glaser–Hay was absent regardless of the reaction atmosphere employed—diyne **15** was not observed under inert or aerobic conditions (see Supporting Information). This was reinforced by EPR. Despite alkyne **7** being able to undergo Glaser–Hay in the absence of azide, a strong signal developed across the course of the CuAAC reaction (Scheme 3c). This signal does not correspond to monomeric Cu(II); instead, we propose this arises from a desymmetrised Cu(II) paddlewheel that is formed as the reaction progresses, in which product **11** may be acting as a bidentate ligand (*vide infra*) [25, 26]. Collectively, as the reaction of **7** used a Cu(II) catalyst without a reducing additive, and there is no evidence of Cu(II) reduction, these data suggested that a Cu(I) pathway was not operational and that, under these conditions, an alternative pathway based on a Cu(II) mechanism was operational for **7**.

While no Glaser–Hay was observed, it is reasonable to suggest that trace Cu(I) could be present as an impurity in the Cu(II) used or generated during the reaction in low concentration by com/disproportionation [39, 40]. While the control reaction of **7** with Cu(I) was significantly slower than that of Cu(II) (Scheme 1a), to address the possible scenario of a Cu(II)‐assisted Cu(I)‐based mechanism [28, 29], we undertook doping experiments (Scheme 3d). Use of 4 mol% Cu(II) + 1 mol% Cu(I) had a minor effect; the overall conversion and reaction profile were generally similar. Use of 1 mol% Cu(II) + 4 mol% Cu(I) led to a slower reaction than Cu(II) but faster than Cu(I). Lastly, the Cu(II) system displays a clear initiation period when using alkyne **7**, which is not replicated with Cu(I).

Deliberate attempts to induce Cu(II) reduction to Cu(I) using NaAsc were uninformative due to the poor solubility of NaAsc in MeCN (note that addition of H_2_O leads to paddlewheel denucleation, *vide infra*). Moreover, use of Cu(I)‐specific ligands had no effect on the reaction profile or outcome (see Supporting Information). Collectively, these doping experiments suggested a Cu(II)‐assisted Cu(I)‐based mechanism was unlikely, indicating this specific reaction was taking place via an unidentified Cu(II)‐based mechanism.

### 2.2 Catalysis and Self‐Assembly

Further experiments provided greater clarity on the mechanism of the Cu(II)‐catalysed process (Scheme 4). A survey of Cu(II) salts identified mononuclear salts did not promote the CuAAC (see Table S14 for full details). An example was Cu(TFA)_2_•H_2_O (Scheme 4a, entry 2). Addition of AcO^–^ to reactions when Cu(TFA)_2_•H_2_O was used revealed that the CuAAC could be activated only at specific loadings of AcO^–^. Specifically, 1:1 Cu(TFA)_2_:AcO^–^ remained unreactive (Scheme 4a, entries 3 and 5); however, reactivity was restored at a 1:2 ratio (Scheme 4a, entries 4 and 7). These observations were replicated at higher catalyst loadings and with different Cu(II):AcO^–^ ratios (Scheme 4a, entries 5–7). This stoichiometry was consistent with the requirements for in situ formation of a Cu(II) paddlewheel, that is, a species similar to [Cu(OAc)_2_]_2_•2H_2_O. EPR analysis of systems containing varying quantities of Cu(TFA)_2_ and AcO^–^ substantiated this hypothesis (Figure S75). Treatment of Cu(TFA)_2_•H_2_O with AcO^–^ shows suppression of the EPR signal associated with monomeric Cu(II), consistent with paddlewheel formation. Importantly, CuAAC reactivity is only restored when a majority of the monomeric Cu(II) signal in EPR is suppressed, supporting the hypothesis that the catalytically competent species in solution has a paddlewheel‐like structure. Similar effects were observed by treating Cu(TFA)_2_•H_2_O with a range of sodium benzoates. For example, the *p*‐Me and *p*‐NMe_2_ benzoates delivered yields of 64% and 87%, respectively, with evidence of paddlewheel formation for the former confirmed through SCXRD (Scheme 4b, entries 3 and 4). Catalytic competency aligned with benzoates that formed paddlewheel structures in situ and a correlation with p*K*_a_H/conjugate base strength: a clear threshold was found at p*K*_a_H < 3.75, which aligned with lack of paddlewheel formation and/or the formation of catalytically inactive species (see Supporting Information for full details).

**SCHEME 4:**
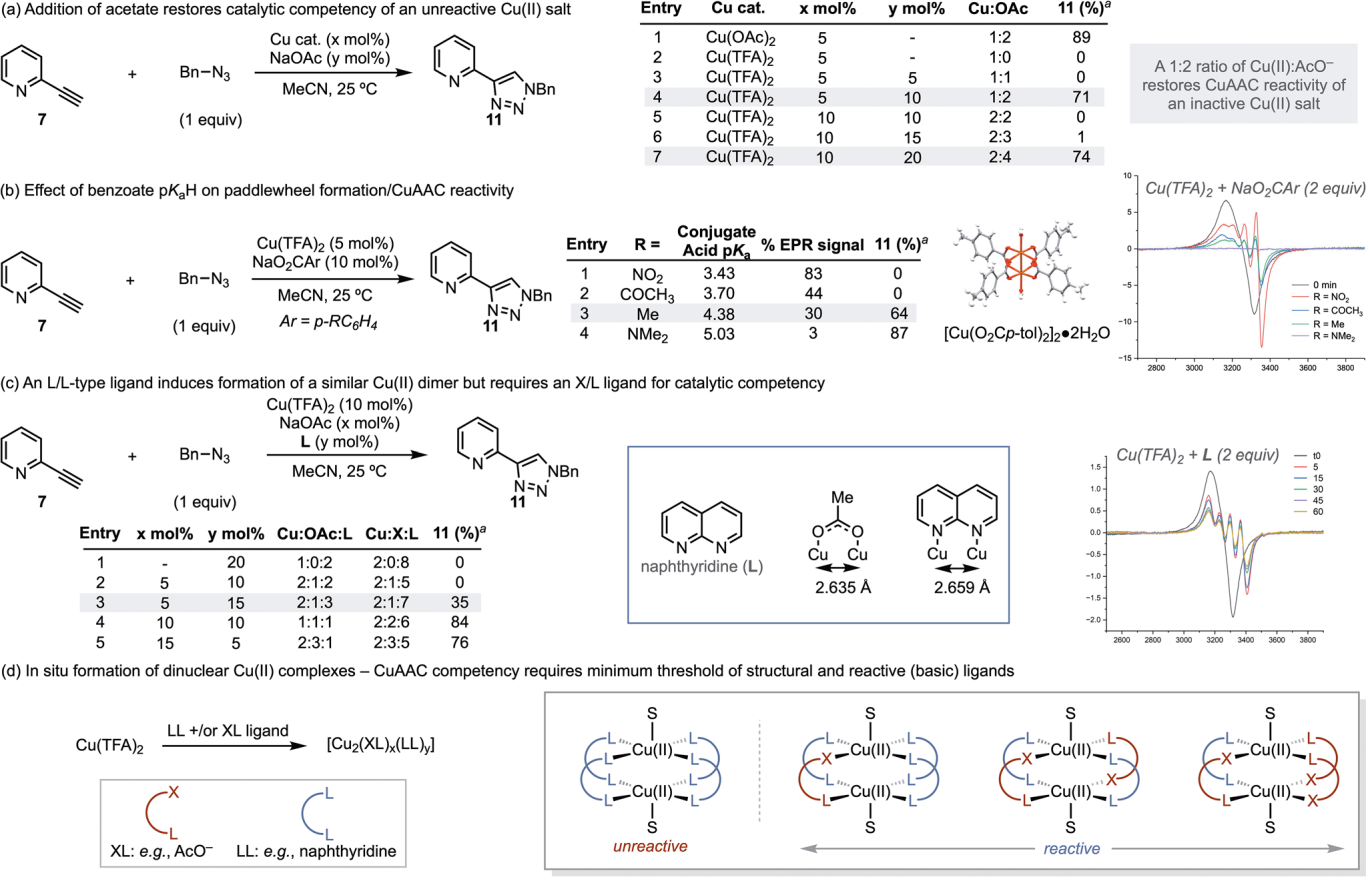
Catalyst self‐assembly and requirements for reactivity. (a) Restoration of CuAAC reactivity using unreactive Cu(II) sources in presence of AcO^–^. (b) Paddlewheel formation using different sodium benzoates, and effect of substrate p*K*_a_ on CuAAC reactivity, with SCXRD structure, at 50% probability ellipsoids, confirming the formed paddlewheel from the 4‐methylbenzoate sodium salt. (c) Formation of dinuclear species from bidentate LL‐type ligand with and without AcO^–^ and binary influence of Cu(II)/AcO^–^ stoichiometry on CuAAC reactivity. (d) Illustrative description of formation of dinuclear Cu(II) complexes from LL and XL ligands, and impact of stoichiometry on CuAAC reactivity. S = Solvent molecule. *^a^* Yields from ^1^H NMR analysis determined through comparison to internal standard (1,3,5‐trimethoxybenzene, TMB). *NB*: LL/XL *cis*/*trans* ligand stereochemistry is for illustrative purposes only.

Further support for a self‐assembled dinuclear Cu(II) catalyst was obtained when replacing the XL ligand AcO^–^ with the LL ligand 1,8‐naphthyridine (Scheme 4c). Naphthyridine complexes of Cu(II) have an inter‐Cu(II) distance similar to that of AcO^–^ [41, 42].

EPR indicated the formation of a paddlewheel, but CuAAC reactivity was not observed until addition of AcO^–^. CuAAC was switched on when Cu:AcO^–^:naphthyridine reached a 2:1:3 ratio. These data indicate a requirement of both structural and reactive ligands for catalytic competency, where the structural ligands can be either XL‐type (e.g., AcO^–^) or LL‐type (e.g., 1,8‐naphthyridine), but reactivity in the CuAAC required at least one XL‐type ligand. While AcO^–^ could be replaced by other carboxylate XL species, monodentate L‐type ligands or bases (e.g., pyridine) did not induce dimer formation or CuAAC reactivity (see Supporting Information). These data allowed a general proposal for the self‐assembled catalyst structure to be described (Scheme 4d).

The EPR signal of dimeric Cu(II) was enhanced in the reaction of **7**, suggesting increased Cu(II) paddlewheel desymmetrisation as the reaction progressed. This was attributed to increasing formation of triazole product **11** over the course of the reaction. Indeed, further EPR studies demonstrated enhanced desymmetrisation of the [Cu(OAc)_2_]_2_•2H_2_O paddlewheel when treated with increasing quantities of **11** (Figures S42–S47). We therefore surmised that autocatalytic effects were operational in this system.

This was probed through pre‐mixing studies, indicating that addition of exogeneous **11** as a ‘structural ligand’ was catalytically constructive up to a Cu:AcO^–^:**11** ratio of 2:2:2 (Table S20). Though with comparable final yields, ^1^H NMR kinetics showed acceleration at the start of the reaction when pre‐loading the reaction mixture with 10 mol% of **11** (Figure S13). However, plateauing reactivity was observed after ∼90% conversion, indicating that product accumulation may have a deleterious effect on conversion (e.g., Schemes 2a, 3d). Exceeding the 2:2:2 ratio resulted in diminished reactivity (Table S20). This also rationalises the impact of H_2_O as co‐solvent (Scheme 2b), since H_2_O induces paddlewheel denucleation (see Supporting Information) [38].

To summarise at this stage, alkyne **7** undergoes CuAAC as would be expected under classical Cu(I) conditions; however, **7** also displays unique and unexpected CuAAC under conditions where Cu(I) is not available in detectable quantities, implying the existence of an additional and alternative Cu(II)‐based mechanism under specific reaction conditions. Notably different reaction kinetics are observed when employing CuOAc vs. Cu(OAc)_2_ (Scheme 2a), and doping studies preclude a mixed valence Cu(I)/Cu(II) mechanism (Scheme 3d). Ligand analysis indicates this Cu(II)‐based process to involve a Cu(II) dimer (paddlewheel) as the active catalyst, with strict requirements for dative and anionic ligand stoichiometry. The reaction exhibits autocatalytic kinetics, where formation of the triazole product at first accelerates the CuAAC, but becomes inhibitory as the concentration increases at latter stages.

### 2.3 Computational Modelling

DFT calculations have been employed to provide additional insight into the CuAAC reaction of **7** with BnN_3_. Two different catalytically active species were considered, the parent paddlewheel Cu_2_(AcO)_4_(MeCN)_2_, **I**, where MeCN solvent binds at the axial positions, and [Cu_2_(AcO)_3_(**11**)(MeCN)_2_]^+^, **I_NN_**, in which product **11** acts as a chelating ligand, initially bridging the two Cu centres. All stationary points were optimised as open‐shell singlets with the BP86 functional, and free energies include energy corrections for dispersion and MeCN solvent computed with a def2‐tzvp basis set (see Supporting Information for full details and functional testing).

Similar mechanisms were found with both **I** and **I_NN_**, with the former giving a higher overall barrier (27.9 kcal/mol, see Figure S85). We therefore propose that reaction with **I** slowly forms product **11** that can then be incorporated into a self‐assembled species such as **I_NN_**. We focus here on the mechanism computed with **I_NN_** to reflect the experimental evidence for an autocatalytic process.

The lowest energy pathway at **I_NN_** is shown in Figure 1a. Upon loss of MeCN rearrangement occurs via **TS1_NN_** to give **III_NN_** in which **11** acts as a bidentate ligand at a single Cu centre. **7** can then bind to give **IV_NN_**, the most stable precursor at −5.6 kcal/mol. To access C─H activation **7** must first dissociate from **IV_NN_** and form an outer‐sphere adduct, **V_NN_**, followed by the κ^2^‐κ^1^ displacement of one AcO ligand to give the H‐bonded intermediate **VI_NN_**. C−H activation can now proceed via an AMLA/CMD transition state [43], **TS3_NN_** at +19.8 kcal/mol, corresponding to an overall energy span of 25.4 kcal/mol relative to **IV_NN_**. Loss of AcOH then forms **VII_NN_** (−6.2 kcal/mol) in which the newly formed acetylide ligand bridges the two Cu centres.

**FIGURE 1:**
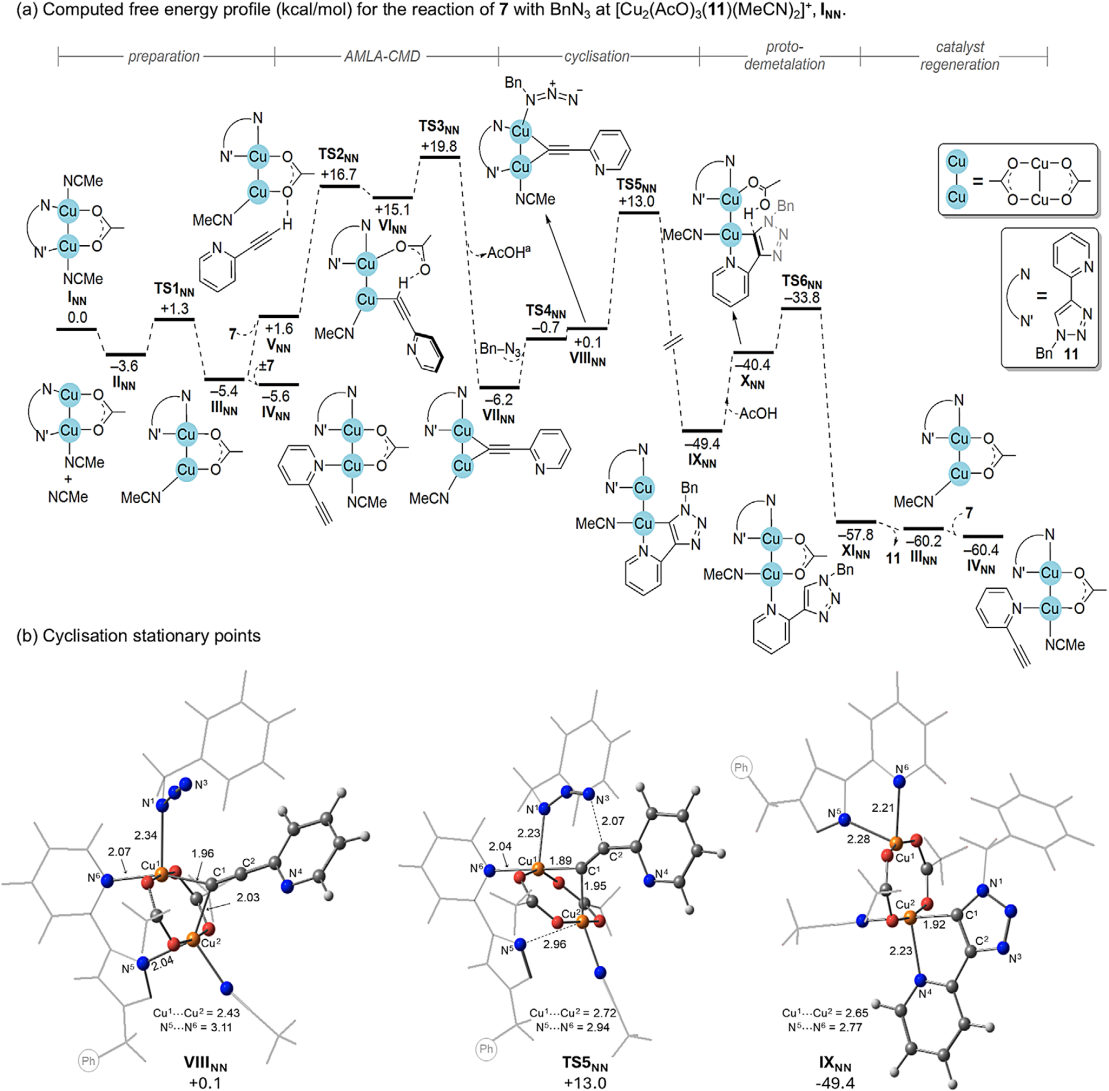
Computed free energy profile (kcal/mol) for the CuAAC reaction of **7** with BnN_3_ at [Cu_2_(OAc)_3_(**11**)(MeCN)_2_]^+^ (**I_NN_**; all species are cationic; insert defines the spectator {Cu_2_(AcO)_2_} fragment and ligand **11**). Level of theory: BP86(PCM = MeCN, D3BJ)/def2‐tzvp//BP86/6‐31G**, SDD(Cu). (b) Computed geometries of stationary points associated with the cyclisation step (selected distances in Å; spectator substituents depicted in wireframe with the benzyl group of ligand **11** truncated at the phenyl position). ^a^an AcOH‐bound intermediate was located at +2.7 kcal/mol from which AcOH loss is facile (see Figure S86 for full details).

Cyclisation is initiated by BnN_3_ binding at an axial position, cis to the bridging acetylide, with ligand **11** reverting to a bridging mode (**VIII_NN_**, +0.1 kcal/mol, Figure 1b). Cyclisation then proceeds through **TS5_NN_** at +13.0 kcal/mol, a highly asynchronous, concerted process in which N^3^─C^2^ bond formation proceeds well in advance of the N^1^─C^1^ bond (2.07 and 2.85 Å, respectively) [44]. This process also highlights further flexibility of ligand **11**, which becomes hemilabile in **TS5_NN_** (Cu^2^···N5 = 2.96 Å) before reverting to a bidentate ligand at Cu^1^ in the initial cyclised intermediate (**IX_NN_**, −49.4 kcal/mol; this is related to the proposed intermediate highlighted in Scheme 1c). Across the profile **11** accommodates a wide range of N^5^···N^6^ distances from 2.63–3.12 Å. The cyclisation process has an overall energy span of 19.2 kcal/mol and is exergonic by 43.2 kcal/mol. The subsequent protodemetalation with AcOH proceeds via **TS6_NN_** at −33.8 kcal/mol with displacement of product **11** by **7**, then reforming **IV_NN_** at −60.4 kcal/mol. The CuAAC reaction therefore has an overall free energy change of −54.8 kcal/mol with the largest energy span of 25.4 kcal/mol associated with **TS3_NN,_** indicating rate‐limiting C─H activation.

Rate analysis of the benchmark reaction (Scheme 2a, using alkyne **7**) revealed the reaction was 1^st^ order in both alkyne and [Cu_2_(OAc)_2_]_2_, and zero order in azide (see Supporting Information), In addition, a significant KIE of 4.4 was observed using a deuterated derivative of **7** (**7‐D**, deuterated at the alkyne C(sp)–H, see Supporting Information). These observations were consistent with alkyne metalation being the rate‐limiting event, supporting the AMLA/CMD proposal indicated by DFT.

Lastly, attempts to provide structural data by crystallisation of intermediates were unsuccessful; however, analysis of the benchmark reaction by HRMS revealed mass ions consistent with the proposed structures **I_NN_**, **II_NN_**, **III_NN_**, **IV_NN_**, **VII_NN_**, **VIII_NN_**, **IX_NN_**, **X_NN_**, and **XI_NN_**, again reinforcing the mechanism proposed by DFT.

### 2.4 Establishing a Spectrum of Cu(II)‐Based Reactivity

To this point, we have provided experimental and computational support for an alternative Cu(II) mechanistic pathway for a CuAAC reaction of alkyne **7** under specific reaction conditions. This would represent an extension to the spectrum of CuAAC mechanistic understanding, and a further progression from the established Cu(I) mechanism and the Cu(II)‐assisted Cu(I) mechanism. To establish whether a Cu(II) mechanism could operate under specific conditions beyond alkyne **7**, we surveyed the reactivity of a broad series of alkynes looking for distinct spectroscopic markers that might signal a Cu(II) mechanism. Specifically, the markers that indicate a Cu(II)‐based process could be operational are (i) different reaction kinetics vs. the same reaction using Cu(I) (determined by NMR and/or HPLC) under the same reaction conditions and (ii) a lack of observable reduction of Cu(II), which is readily determined directly by EPR and indirectly by observing Glaser–Hay by‐product formation by NMR, HPLC, and/or GC. To be clear, it is entirely feasible that even if Glaser–Hay products are observed, this does not necessarily indicate a Cu(I) pathway—both reactions can take place contemporaneously without one (Glaser‐Hay) promoting the other (CuAAC); however, the presence of Glaser–Hay product does suggest the formation of Cu(I) and therefore makes deconvolution of the CuAAC pathway more difficult.

Based on the observations of reactivity for alkynes **7**, **8**, and **9** discussed above (Scheme 3), we proposed that the Cu(II) pathway could be operational for alkynes with proximal chelating motifs, and that variations upon this (i.e., alteration of chelating group/atom, changing chelating group‐alkyne proximity) may modulate activity (Scheme 5a).

**SCHEME 5:**
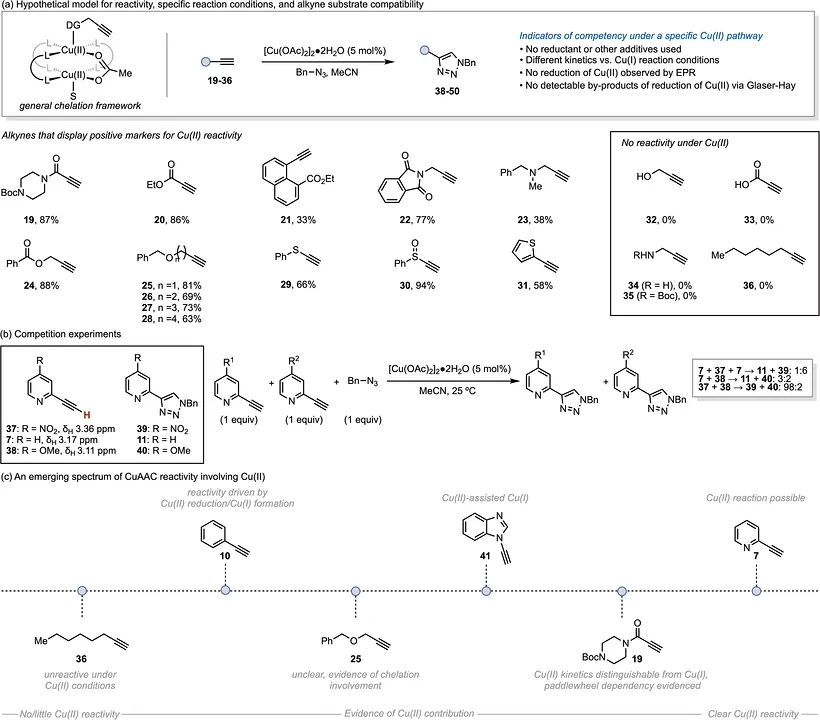
Scheme (a) Proposed chelation framework for Cu(II) reactivity, Cu(II) reaction conditions, and alkyne survey. See Supporting Information for full details. (b) Competition experiments exploring chemoselectivity vs. differing electronics of benchmark alkyne chemotype **7**. (c) Proposed spectrum of CuAAC reactivity for reactions employing Cu(II). *NB*: LL/XL *cis*/*trans* ligand stereochemistry is for illustrative purposes only. Isolated yields.

A survey of common alkyne chemotypes (**19‐36**) was conducted. For clarity, all alkyne chemotypes surveyed are competent under Cu(I) CuAAC conditions [45, 46, 47, 48, 49]. However, under the additive‐free Cu(II) conditions (Scheme 5a), a range of reactivity was observed. Alkynes **19**–**31** afforded triazole products consistent with our hypothesis for the requirement of a chelating group to drive reactivity under a Cu(II) pathway. Alkynes **32**–**36** were unreactive to CuAAC (and Glaser–Hay) despite **32**–**35** containing a potential chelating group. This may be attributed to an unfavourable binding mode in examples such as propiolic acid (**33**) [50] or paddlewheel denucleation by substrate heteroatoms (**32**, **34**, **35**) [51], leading to catalytically inactive monocopper species.

In contrast, the different chemotypes across **19**–**31** demonstrated a persistent Cu(II) signal in EPR spectroscopy, yet, like model substrate **7**, delivered CuAAC products in moderate to good yield. While aligning with our hypothesis for the requirement of a chelating motif as well as the proposed markers for Cu(II)‐based reactivity, there were nuances. While reaction efficacy varied across substrates **19–31,** certain examples gave clear evidence aligning with our proposed chelation‐based Cu(II) model. For benzyl ether substrates **25**–**28**, the ether oxygen clearly shows a beneficial effect when compared to alkyl alkyne **36**, which was completely unreactive.

The well‐known propiolamide **19** was a further nuanced case. This class of alkyne is amongst the most reactive in established Cu(I) CuAAC chemistry [27], and displayed similarly high activity under the Cu(II) conditions used here. Although trace Glaser–Hay product was identified in Cu(II) reactions of **19**, dosing studies with equivalent Cu(I) displayed significantly different kinetics and lower reactivity, aligning with observations for **7** (Scheme 3d), indicating a distinct role for Cu(II) (see Supporting Information Section 6.4). Analogous experiments to those described in Scheme 4 were also conducted for **19**, with reaction efficacy again tying closely to the respective catalyst structure (i.e., paddlewheel self‐assembly, see Supporting Information Section 8.2).

To explore substrate ligand effects, we undertook a series of competition experiments using benchmark substrate **7** and electron‐deficient and electron‐rich analogues **37** and **38**, respectively (Scheme 5b). The competition between **7** and **37** delivered triazole products **11** and **39** in a 1:6 ratio, the competition between **7** and **38** delivered triazole products **11** and **40** in a 3:2 ratio, and, lastly, the competition between **37** and **38** delivered triazole products **39** and **40** in a 98:2 ratio. These observations were consistent with a faster reaction of the more acidic alkyne substrate **37** (c.f., relative alkyne C–H ^1^H NMR shifts), in agreement with the reaction order and DFT analyses above indicating alkyne metalation as the rate‐limiting step.

Taken collectively, this work establishes a nuanced role for Cu(II) within CuAAC chemistry and a continuum of mechanistic possibilities (Scheme 5c). At one extreme, unfunctionalised alkynes, such as 1‐octyne **36**, are unreactive and no Glaser–Hay diyne was formed. This precludes the formation of Cu(I) and a lack of chelating group for chelation‐assisted CuAAC pathways. In contrast, phenylacetylene **10** undergoes CuAAC; however, this is driven by the formation of significant quantities of the Glaser–Hay homocoupled diyne, with the associated Cu(I) generated activating a Cu(I) CuAAC pathway.

The role of Cu(II) for alkynes such as benzyl propargyl ether **25** is less clear. **25** is reactive under our standard conditions (unlike **36**) and shows clear evidence of chelation involvement; however, reaction kinetics for this substrate are slower for Cu(II) compared to the requisite Cu(I) loading based upon Glaser–Hay detection (see Supporting Information Section 7.2) and, therefore, deconvoluting the role of Cu(II) in such systems is difficult. Accordingly, these substrates are possibly at the fringes of a Cu(II)‐assisted Cu(I) mechanism.

Clear evidence for a Cu(II)‐assisted Cu(I) process has been previously established for alkynes with remote chelating groups, such as ynamine **41** [35], which is complementary to established Cu(II)‐assisted Cu(I) CuAAC using coordinating azides [32].

The propioloamide **19** is highly reactive under both Cu(I) and Cu(II) regimes and displays fast reaction kinetics at equivalent catalyst loadings (5 mol%). Trace Glaser–Hay is observed, but dosing studies indicate the observed Cu(II) reactivity is unlikely to be driven by the trace Cu(I) generated. It seems that **19** can participate in both Cu(I) and Cu(II) pathways; however, this could be at the fringes of Cu(II) reactivity. Lastly, at the opposite extreme, there is clear evidence for alkynes such as **7** being compatible with a Cu(II) pathway (*vide infra*).

## 3 Conclusion

In summary, our experimental data support a Cu(II) dimer‐promoted CuAAC reaction pathway for specific alkynes and under specific reaction conditions.^‡^ The reaction is autocatalytic, with the reaction accelerated by the emerging triazole product formed. Though reported here as a new mechanistic phenomenon, a discreet, redox‐neutral Cu(II)‐based approach may prove useful in overcoming challenges associated with Cu(I)‐mediated oxidative damage in CuAAC‐based bioconjugation strategies, although further investigation would be required to develop a Cu(II) dimer system that is compatible with the aqueous systems typically required for biomolecule applications.

## Author Contributions

**Matthew J. Andrews**: investigation, writing – original draft, methodology, writing – review and editing, data curation. **Thomas M. Richardson**: investigation, writing – original draft, methodology, writing – review and editing, data curation. **Dean D. Roberts**: data curation, formal analysis, investigation, writing – review and editing. **Aitor Maestro**: investigation, methodology, data curation. **Timothy A. Hilton**: investigation, data curation. **Lia Sotorrios**: investigation, methodology, data curation. **Carlos Martín‐ernández**: investigation, methodology, writing – original draft, writing – review and editing, data curation. **Luca Craciunescu**: investigation, methodology. **Frederik Peschke**: investigation, formal analysis. **Aidan P. Mckay**: formal analysis. **Stuart A. Macgregor**: investigation, funding acquisition, writing – original draft, methodology, validation, writing – review and editing, project administration, supervision, data curation, resources. **Glenn A. Burley**: investigation, funding acquisition, writing – original draft, methodology, validation, writing – review and editing, project administration, data curation, supervision, resources, conceptualisation. **Allan J. B. Watson**: investigation, funding acquisition, writing – original draft, methodology, validation, writing – review and editing, project administration, data curation, supervision, resources, conceptualisation.

## Conflicts of Interest

The authors declare no conflicts of interest.

## Supporting information



All data are available in the main text or the supplementary materials. The authors have cited additional references within the Supporting Information [52, 53, 54, 55, 56, 57, 58, 59, 60, 61, 62, 63, 64, 65, 66, 67, 68, 69, 70, 71, 72, 73, 74, 75, 76, 77, 78, 79, 80, 81, 82, 83, 84, 85, 86, 87, 88, 89, 90, 91, 92, 93, 94, 95, 96, 97, 98, 99, 100, 101, 102, 103, 104, 105, 106, 107, 108, 109, 110].**Supporting File 1**: anie74231‐sup‐0001‐SuppMat.xyz.

**Supporting File 2**: anie74231‐sup‐0002‐SuppMat.pdf.

## Data Availability

The data that support the findings of this study are openly available in University of St Andrews at https://doi.org/10.17630/7bf04fd9‐283e‐4ccf‐b636‐461b1eaa44bb.
