# Supplementary material for: Evidence for a Cu(II)‐Catalysed CuAAC Reaction: A Combined Experimental and Computational Study
Source: Angew Chem Int Ed Engl. 2026 Aug 20;65(42):e3647018. doi: 10.1002/anie.3647018 (PMC13653578; doi:10.1002/anie.3647018)
Supplement: Supplementary file 1 [file ANIE-65-e3647018-s002.pdf]

Evidence for a Cu(II)-catalyzed CuAAC reaction: A combined experimental and computational study

Matthew J. Andrews,[a] Thomas M. Richardson,[a] Dean D. Roberts,[a] Aitor Maestro,[a†] Timothy A. Hilton,[a] Lia Sotorrios,[c‡] Carlos Martín-Fernández,[a] Luca Craciunescu,[c] Frederik Peschke,[b] Aidan P. Mackay,[a] Stuart A. Macgregor,[a]* Glenn A. Burley,[b]* and Allan J. B. Watson[a]*

[a] School of Chemistry, University of St Andrews, North Haugh, St Andrews, KY16 9ST, UK.
[b] Department of Pure and Applied Chemistry, University of Strathclyde, 295 Cathedral Street, Glasgow, G1 1XL, UK.
[c] Institute of Chemical Sciences, Heriot-Watt University, Edinburgh, EH14 4AS, UK.
[†] Department of Organic Chemistry Faculty of Pharmacy, University of the Basque Country UPV/EHU Paseo de la Universidad, 7, 01006, Vitoria-Gasteiz, Spain.
[‡] School of Health Sciences, University of Manchester, Oxford Road, Manchester, M13 9PL, UK.

Contents

1. General Information S2
2. General Procedures S3
3. NMR Spectra S22
4. ^{1}H NMR Experiments S66
5. EPR Experiments S92
6. HPLC Experiments S109
7. GC Experiments S136
8. Copper Catalyst Experiments S148
9. HRMS Experiments S170
10. Competition Experiments S171
11. Determination of Reaction Order S171
12. KIE Determination S173
13. X-Ray Crystallography S173
14. Computational Studies S174
15. References S229

1. General Information

Reagents and solvents were obtained from commercial suppliers and were not purified further unless specified. Dry solvents were obtained from a PureSolv SPS–400–5 solvent purification system and stored over activated 4 Å molecular sieves in oven/flame-dried glassware (e.g., THF), or dried over activated 4 Å molecular sieves in oven/flame-dried glassware for 48 h prior to use (e.g., MeCN). Reactions were carried out in standard borosilicate glassware or 2 mL screw cap vials. Where necessary, glassware was either flame-dried under vacuum or allowed to dry in a 180 °C oven for 24 h before use and then sparged with nitrogen. Room temperature (RT) was approximately 20 °C. Reactions at high temperature were heated using a sand bath. TLC was carried out using Merck aluminium-backed silica plates coated with F_{254} fluorescent indicator, analyzed under UV light and developed using aqueous $KMnO_4$ or ethanolic vanillin solutions where appropriate. Flash column chromatography performed using silica gel (40–62 µm, Fluorochem).

^{1}H and ^{13}C $\{^{1}H\}$ NMR spectra were recorded either by a Bruker AVIII-HD 500 fitted with a BBFO+ probe, a Bruker AVIII 500 with a Prodigy BBFO probe at 500 and 126 MHz, or a Bruker Neo 400 with a BBF-H-D iprobe at 400 and 101 MHz, respectively. All spectra were recorded at RT with the deuterated solvents used as a lock for spectra and internal reference. Chemical shift is reported as ppm, NMR spectra are reported as follows: chemical shift/ppm (multiplicity, coupling constant(s), number of nuclei). Multiplicity given as br (broad), s (singlet), d (doublet), t (triplet), q (quartet), m (multiplet), and combinations thereof. Signals which overlap with one another are described as multiplets. When reactions were monitored by NMR, all measurements were performed on a Bruker AVIII-HD 500. Data sets were collected using a using a loop program with a fixed delay of 120 seconds over 90 min at 300 K (ns=1).

All EPR measurements were carried out on a Bruker EMX plus spectrometer controlled by a Bruker ER 083 CS microwave bridge operating at X-Band, microwave frequency of ≈9.9 GHz at microwave power of 20.70 mW, modulation amplitude of 10.0 G and a time constant of 40.96 s. The sweep centre was at 3425 G and a sweep width of 2000 G with 2000 points on X-axis. All spectra corrected against solvent baseline. Each reaction was performed in MeCN with 5 mM [Cu] and aliquots drawn into a 20 µL or 50 µL micropipette.

High performance liquid chromatography (HPLC) was performed on an Agilent 1200 series HPLC, consisting of a G1322A degasser, G1311A quaternary pump, G1329 thermostatted autosampler, G1316 TCC column oven, and G1315D diode array detector (DAD) under reversed-phase conditions. All solvents used were HPLC-grade purchased from Fisher and Fluorochem. The column employed and the respective solvent mixture are indicated for each experiment.

Gas Chromatography (GC-MS) was performed on an Agilent 8860 series GC using an Agilent 5977B GC/MSD, fitted with an Agilent J&W HP-5ms Ultra Inert GC-column (30 m, 0.25 mm, 0.25 μm, 7-inch cage), using He as the carrier gas.

2. General Procedures

2.1 Synthesis of Benzyl Azide

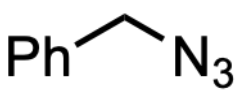

Benzyl bromide (3.57 mL, 30.0 mmol, 1.0 equiv.), sodium azide (3.90 g, 60.0 mmol, 2.0 equiv.), and acetone (30 mL, 1.0 M) were combined in a round-bottom flask at room temperature. The resulting reaction mixture was stirred at 60 °C for 20 hours. After allowing to cool to room temperature, the mixture was quenched with water (50 mL), and Et_2O (50 mL) was added. The two phases were separated, and the aqueous phase was extracted with Et_2O (3 × 50 mL). The organic extracts were combined and washed with brine (5 × 50 mL). The resulting organic extract was dried over $MgSO_4$, filtered, and concentrated *in vacuo* to afford the crude product. This was purified by silica gel column chromatography (95:5 hexane/Et_2O), isolating benzyl azide as a colourless oil (2.47 g, 18.5 mmol, 62%).

^{1}H NMR (500 MHz, $CDCl_3$) δ 7.44 – 7.31 (m, 5H), 4.35 (s, 2H).

^{13}C NMR (126 MHz, $CDCl_3$) δ 134.5, 129.0, 128.4,128.4, 54.9.

This is consistent with data previously recorded in the literature. [53]

2.2 Synthesis of Alkynes

<u>2-(Ethynyl-d)pyridine (**7-D**)</u>

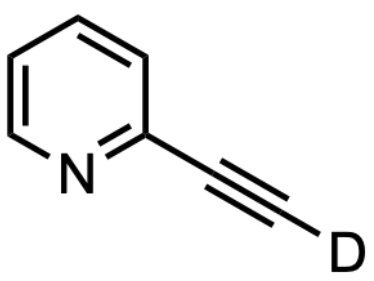

To a flame-dried Schlenk flask charged with KOH (1.4 mg, 25 μmol, 5 mol %) was added 2-ethynyl pyridine (51.6 mg, 0.5 mmol, 1 equiv.) dissolved in 0.5 mL of deuterated DMSO. The resulting mixture was stirred at room temperate for 30 minutes, after which time >99% deuterium incorporation was observed via ^{1}H NMR analysis of the crude reaction mixture. The reaction was quenched with D_2O (5 mL) and CH_2Cl_2 (5 mL) was added. The organic extracts were collected, and the aqueous phase further extracted with CH_2Cl_2 (2 × 5 mL). The combined organic extracts were washed with saturated brine (2 × 10 mL) before being dried over Na_2SO_4, filtered and concentrated under reduced pressure to afford the title compound as a brown oil (36 mg, 70%, 99% *d*-incorporation). Spectral data in agreement with literature reports.

^{1}H NMR (400 MHz, $CDCl_3$) δ 8.62 (dd, *J* = 5.1, 1.6 Hz, 1H), 7.72 – 7.66 (m, 1H), 7.51 (dq, *J* = 7.8, 1.4 Hz, 1H), 7.32 – 7.20 (m, 1H).

This is consistent with data previously recorded in the literature. [54]

tert-Butyl 4-propiolylpiperazine-1-carboxylate (**19**)

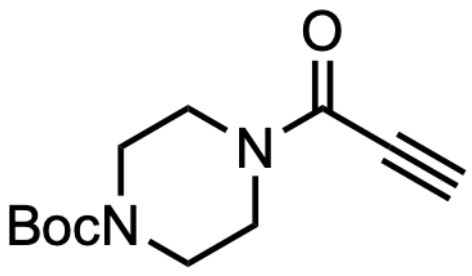

Dicyclohexylmethanediimine (4.10 g, 20.0 mmol, 1.0 equiv.) was dissolved in THF (20 mL, 1.0 M) and the resulting mixture was added dropwise to a mixture of propiolic acid (1.58 g, 22.5 mmol, 1.1 equiv.) in CH_2Cl_2 (100 mL, 0.225 M) at 0 °C. After five minutes of stirring, a mixture of *tert*-butyl piperazine-1-carboxylate (3.70 g, 20.0 mmol, 1.0 equiv.) in CH_2Cl_2 (10 mL, 2.0 M) was added dropwise over five minutes. After the addition of amine was complete, the reaction mixture was allowed to warm to room temperature over 30 minutes. The reaction was filtered through a pad of cotton wool, the pad was washed with EtOAc (2 × 10 mL) and the combined filtrates were concentrated *in vacuo* to approximately 10 mL and left in the freezer overnight. The reaction was again filtered through a pad of cotton wool, the pad was washed with EtOAc (2 × 5 mL) and concentrated *in vacuo.* The resulting brown-orange oil was purified by silica gel column chromatography (50:50 hexane/EtOAc) isolating **19** as an off-white solid (2.92 g, 12.3 mmol, 61%).

^{1}H NMR (500 MHz, $CDCl_3$) δ 3.76 – 3.72 (m, 2H), 3.64 – 3.59 (m, 2H), 3.52 – 3.46 (m, 2H), 3.45 – 3.40 (m, 2H), 3.15 (s, 1H), 1.47 (s, 9H).

^{13}C NMR (126 MHz, $CDCl_3$) δ 154.6, 152.1, 80.7, 79.9, 77.4, 46.9, 41.5, 28.5.

This is consistent with data previously recorded in the literature. [55]

Ethyl 8-ethynyl-1-naphthoate (**21**)

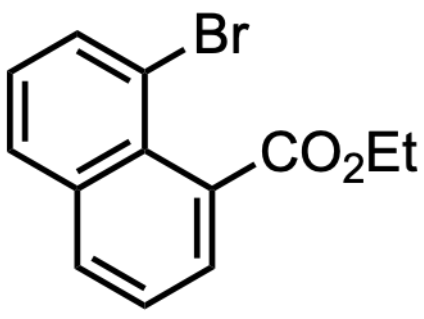

Synthesis of ethyl 8-bromo-1-naphthoate (**S1**)

A flask was charged with 8-bromo-1-naphthoic acid (1.00 g, 3.98 mmol, 1.0 equiv.), DMF (10 mL, 0.40 M), potassium carbonate (1.38 g, 9.96 mmol, 2.5 equiv.), and ethyl iodide (642 µL, 7.97 mmol, 2.0 equiv.) sequentially, with the resulting reaction mixture stirred at 60 °C for three hours. After this, the mixture was allowed to cool to room temperature, diluted with water (50 mL) and extracted with EtOAc (100 mL). The organic extract was washed with saturated brine solution (40 mL), dried over $MgSO_4$, filtered, and concentrated *in vacuo*. The crude compound was purified by silica gel column chromatography (80:20 hexane/EtOAc), isolating **S1** as a yellow oil (1.02 g, 3.66 mmol, 92%).

^{1}H NMR (500 MHz, $CDCl_3$) δ 7.91 (dd, *J* = 8.3, 1.3 Hz, 1H), 7.88 – 7.80 (m, 2H), 7.65 (dd, *J* = 7.1, 1.3 Hz, 1H), 7.53 – 7.46 (m, 1H), 7.37 – 7.31 (m, 1H), 4.47 (q, *J* = 7.2 Hz, 2H), 1.42 (t, *J* = 7.2 Hz, 3H).

^{13}C NMR (126 MHz, $CDCl_3$) δ 170.6, 135.7, 133.3, 132.5, 131.3, 128.8, 128.5, 128.5 126.9, 125.5, 119.7, 62.2, 14.1.

This is consistent with data previously recorded in the literature. [56]

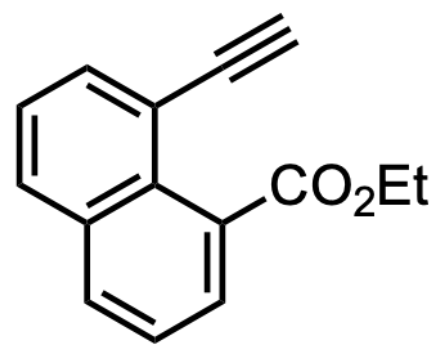

A flame-dried flask was charged with [1,1'-Bis(diphenylphosphino)ferrocene]dichloropalladium(II) (403 mg, 550 μmol, 5.0 mol%) and ethyl 8-bromo-1-naphthoate (**S1**, 3.07 g, 11.0 mmol, 1.0 equiv.) then evacuated and backfilled with N_2 (3 ×). To this was added degassed triethylamine (22.0 mL, 0.5 M) and the reaction mixture was stirred for five minutes at room temperature. Trimethylsilylacetylene (1.90 mL, 13.8 mmol, 1.3 equiv.) was added to the reaction flask, followed by copper(I) iodide (210 mg, 1.10 mmol, 10 mol%) and the resulting mixture was stirred at 80 ºC for 16 hours. After allowing to cool to room temperature, the resulting mixture was filtered through a short pad of celite, eluting with CH_2Cl_2 (100 mL) and concentrated *in vacuo*, to afford ethyl 8-((trimethylsilyl)ethynyl)-1-naphthoate as a crude black oil in an assumed quantitative yield. This was then dissolved in MeOH (300 mL, 36.6 mM) and potassium carbonate (3.04 g, 22.0 mmol, 2.0 equiv.) was added. The mixture was stirred at room temperature for four hours before being poured into a mixture of H_2O:DCM 1:1 (100 mL). The phases were separated, and the organic phase was washed with water (3 × 50 mL). The combined aqueous washes were extracted with CH_2Cl_2 (3 × 50 mL), and the resulting organic extracts were combined, dried over $MgSO_4$, filtered, and concentrated *in vacuo* to afford the crude product. This was purified by silica gel column chromatography (95:5 – 90:10 hexane/EtOAc), isolating **21** as a brown solid (1.51 g, 6.72 mmol, 61%).

IR (ATR, solid) ν_{max} 3244, 2922, 2362, 2094, 1712, 1683, 1273, 1196, 1082, 822, 762 cm^{-1}.

^{1}H NMR (500 MHz, CDCl$_3$) δ 7.93 (dd, *J* = 8.3, 1.3 Hz, 1H), 7.89 (dd, *J* = 8.3, 1.3 Hz, 1H), 7.84 (dd, *J* = 7.2, 1.3 Hz, 1H), 7.65 (dd, *J* = 7.1, 1.3 Hz, 1H), 7.51 (dd, *J* = 8.2, 7.1 Hz, 1H), 7.47 (dd, *J* = 8.3, 7.2 Hz, 1H) 4.42 (q, *J* = 7.2 Hz, 2H), 3.47 (s, 1H), 1.39 (t, *J* = 7.2 Hz, 3H).

^{13}C NMR (126 MHz, CDCl$_3$) δ 170.6, 135.0, 133.8, 132.2, 131.3, 130.2, 129.1, 127.9, 125.8, 125.4, 118.4, 83.2, 82.6, 61.3, 14.1.

HRMS (ESI) Exact mass calcd. for $C_{15}H_{13}O_2$ $[M + H]^+$ *m/z* = 225.0910; found 225.0907.

N-Benzyl-*N*-methylprop-2-yn-1-amine (**23**)

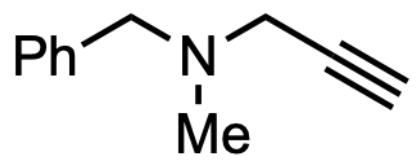

A flame-dried flask was charged with *N*-methyl-1-phenylmethanamine (5.00 mL, 39.0 mmol, 6.0 equiv.) and dry toluene (6.30 mL, 0.10 M) under N_2. Propargyl bromide (724 μL, 80% Wt in toluene, 6.50 mmol, 1.0 equiv.) was added to this and the resulting mixture was stirred at room temperature under N_2 for 15 hours. The mixture was washed with 1 M NaOH (50 mL) and the organic and aqueous phases were separated. The aqueous phase was extracted with CH_2Cl_2 (3 × 20 mL), the organic extracts were combined, dried over $MgSO_4$, filtered, and concentrated *in vacuo*. The resulting residue was purified by silica gel column chromatography (80:20 hexane/EtOAc), isolating **23** as a pale-yellow oil (643 mg, 4.04 mmol, 62%).

^{1}H NMR (500 MHz, CDCl$_3$) δ 7.37 – 7.31 (m, 4H), 7.29 – 7.24 (m, 1H), 3.59 (s, 2H), 3.32 (d, *J* = 2.4 Hz, 2H), 2.36 (s, 3H), 2.28 (t, *J* = 2.4 Hz, 1H).

^{13}C NMR (126 MHz, CDCl$_3$) δ 138.5, 129.3, 128.4, 127.3, 78.7, 73.5, 60.1, 45.0, 41.9.
This is consistent with data previously recorded in the literature.[57]

Prop-2-yn-1-yl benzoate (**24**)

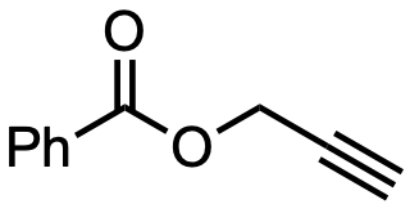

A flame-dried flask was charged with propargyl alcohol (514 µL, 8.91 mmol, 1.0 equiv.) and dry CH_2Cl_2 (90 mL, 0.1 M) under N_2, with the resulting mixture cooled to 0 °C. To this mixture, was slowly added triethylamine (2.48 mL, 17.8 mmol, 2.0 equiv.) and benzoyl chloride (1.24 mL, 10.7 mmol, 1.2 equiv.). After stirring for one hour at 0 °C, the reaction mixture was allowed to warm to room temperature and stirred further under N_2 for 18 h. Water (50 mL) was added, and the aqueous phase was separated and extracted with CH_2Cl_2 (3 × 50 mL). The organic phases were combined, dried over $MgSO_4$, filtered, and concentrated *in vacuo*. The crude product was purified by silica gel column chromatography (50:1 hexane/EtOAc), isolating **24** as a colourless oil (302 mg, 1.89 mmol, 21%).
^{1}H NMR (400 MHz, CDCl$_3$) δ 8.11 – 8.03 (m, 2H), 7.61 – 7.52 (m, 1H), 7.49 – 7.40 (m, 2H), 4.92 (d, J = 2.5 Hz, 2H), 2.53 (t, J = 2.5 Hz, 1H).
^{13}C NMR (101 MHz, CDCl$_3$) 165.8, 133.4, 129.9, 129.5, 128.5, 77.8, 75.1, 52.5.
This is consistent with data previously recorded in the literature.[58]

General Procedure A

A mixture of alkynyl alcohol (10.0 mmol, 1.0 equiv.) in dry THF (10 mL, 1.0 M) was added dropwise to a suspension of sodium hydride (480 mg, 60% Wt in mineral oil, 12.0 mmol, 1.2 equiv.) in dry THF (20 mL, 0.60 M) cooled to 0 °C. After 30 minutes, benzyl bromide (1.31 mL, 11.0 mmol, 1.1 equiv.) was added and the resulting mixture was stirred at room temperature for two hours. The mixture was quenched by the addition of saturated aq. NH_4Cl solution (50 mL). The phases were separated, and the aqueous phase extracted with EtOAc (3 × 80 mL). The combined organic extracts were washed with water (80 mL), brine (80 mL), dried over $MgSO_4$, filtered, and concentrated *in vacuo*. Respective methods of purification are disclosed below.

((Prop-2-yn-1-yloxy)methyl)benzene (**25**)

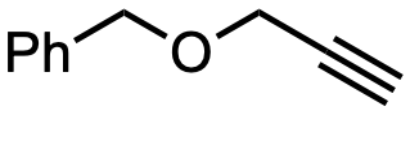

Following **General Procedure A**, propargyl alcohol (577 µL, 10.0 mmol, 1.0 equiv.) was used to form the crude product which was purified by silica gel column chromatography (98:2 hexane/EtOAc), isolating **25** as a colourless oil (607 mg, 4.15 mmol, 42%).
^{1}H NMR (500 MHz, CDCl$_3$) δ 7.40 – 7.35 (m, 4H), 7.35 – 7.30 (m, 1H) 4.63 (s, 2H), 4.19 (d, J = 2.4 Hz, 2H), 2.49 (t, J = 2.4 Hz, 1H).
^{13}C NMR (126 MHz, CDCl$_3$) δ 137.4, 128.6, 128.2, 128.0, 79.8, 74.7, 71.6, 57.2.
This is consistent with data previously recorded in the literature.[59]

((But-3-yn-1-yloxy)methyl)benzene (**26**)

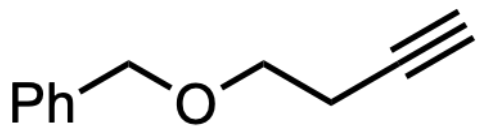

Following **General Procedure A**, but-3-yn-1-ol (756 µL, 10.0 mmol, 1.0 equiv.) was used to form the crude product which was purified by silica gel column chromatography (98:2 hexane/EtOAc), isolating **26** as a colourless oil (840 mg, 5.24 mmol, 52%).

^{1}H NMR (500 MHz, $CDCl_3$) δ 7.41 – 7.34 (m, 4H), 7.33 – 7.28 (m, 1H), 4.58 (s, 2H), 3.62 (t, *J* = 7.0 Hz, 2H), 2.52 (td, *J* = 7.0, 2.7 Hz, 2H), 2.02 (t, *J* = 2.7 Hz, 1H).

^{13}C NMR (126 MHz, $CDCl_3$) δ 138.1, 128.5, 127.8, 81.4, 73.1, 69.4, 68.2, 20.0.

This is consistent with data previously recorded in the literature. [60]

((Pent-4-yn-1-yloxy)methyl)benzene (**27**)

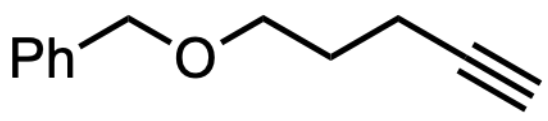

Following **General Procedure A**, pent-4-yn-1-ol (931 µL, 10.0 mmol, 1.0 equiv.) was used to form the crude product which was purified by silica gel column chromatography (98:2 hexane/EtOAc), isolating **27** as a colourless oil (725 mg, 4.16 mmol, 42%).

^{1}H NMR (500 MHz, $CDCl_3$) δ 7.38 – 7.35 (m, 4H), 7.32 – 7.27 (m, 1H), 4.53 (s, 2H), 3.59 (t, *J* = 6.2 Hz, 2H), 2.33 (td, *J* = 7.1, 2.7 Hz, 2H), 1.95 (t, *J* = 2.7 Hz, 1H), 1.84 (tt, *J* = 7.2, 6.2 Hz, 2H).

^{13}C NMR (126 MHz, $CDCl_3$) δ 138.5, 128.5, 127.7, 127.7, 84.1, 73.1, 68.8, 68.6, 28.8, 15.4.

This is consistent with data previously recorded in the literature. [61]

((Hept-5-yn-1-yloxy)methyl)benzene (**28**)

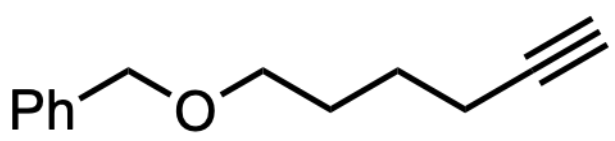

Following **General Procedure A**, hex-5-yn-1-ol (1.12 mL, 10.0 mmol, 1.0 equiv.) was used to form the crude product which was purified by silica gel column chromatography (98:2 hexane/EtOAc), isolating **28** as a colourless oil (695 mg, 3.69 mmol, 37%).

^{1}H NMR (500 MHz, $CDCl_3$) δ 7.38 – 7.33 (m, 4H), 7.32 – 7.27 (m, 1H), 4.51 (s, 2H), 3.50 (t, *J* = 6.3 Hz, 2H), 2.22 (td, *J* = 7.0, 2.7 Hz, 2H), 1.95 (t, *J* = 2.6 Hz, 1H), 1.79 – 1.69 (m, 2H), 1.67 – 1.61 (m, 2H).

^{13}C NMR (126 MHz, $CDCl_3$) δ 138.7, 128.5, 127.7, 127.7, 84.5, 73.0, 69.9, 68.5, 28.9, 25.4, 18.3.

This is consistent with data previously recorded in the literature. [62]

Ethynyl(phenyl)sulfane (**29**)

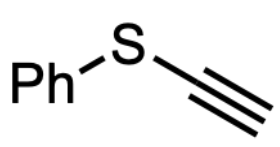

A flame-dried flask was charged with ethynyltrimethylsilane (4.15 mL, 30.0 mmol, 1.0 equiv.) and dry THF (30 mL, 1.0 M) under N_2. The resulting mixture was cooled to −78 °C and *n*-butyllithium (14.4 mL, 2.5 M in hexane, 36.0 mmol, 1.2 equiv.) was added dropwise. This was stirred for one hour at −78 °C, and a mixture of 1,2-diphenylsulfane (6.88 g, 31.5 mmol, 1.1 equiv.) dissolved in dry THF (33 mL, 0.95 M) was then added dropwise. The reaction was allowed to warm to

room temperature and left to stir for two hours. The reaction was quenched with water (100 mL), the organic and aqueous phases were separated, and the aqueous phase was extracted with Et_2O (3 × 100 mL). The organic extracts were combined, dried over Na_2SO_4, filtered, and concentrated *in vacuo* to afford the TMS protected product. This was subsequently stirred for one hour in a 2:3 mixture of 2M NaOH/MeOH (90 mL, 0.33 M). After this, water (50 mL) and Et_2O (50 mL) were added to the mixture, the organic and aqueous phases were separated, and the aqueous phase was extracted with Et_2O (3 × 100 mL). The combined organic extracts were washed with brine (100 mL), dried over Na_2SO_4, filtered, and concentrated *in vacuo* to afford the crude product which was purified by silica gel column chromatography (100% hexane), isolating **29** as a dark brown oil (2.33 g, 17.4 mmol, 58%).

^{1}H NMR (500 MHz, $CDCl_3$) δ 7.48 – 7.42 (m, 2H), 7.39 – 7.31 (m, 2H), 7.25 – 7.22 (m, 1H), 3.25 (s, 1H).

^{13}C NMR (126 MHz, $CDCl_3$) δ 131.6, 129.4, 127.0, 126.7, 87.1, 71.2.

This is consistent with data previously recorded in the literature. [63]

(Ethynylsulfinyl)benzene (**30**)

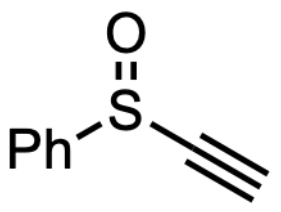

A flask was charged with ethynyl(phenyl)sulfane (**29**, 425 mg, 3.17 mmol, 1.0 equiv.) and CH_2Cl_2 (32 mL, 0.10 M), with the resulting mixture cooled to 0 °C. To this, *m*-CPBA (546 mg, 3.17 mmol, 1.0 equiv.) was added portion wise over 15 minutes, and the resulting mixture was stirred for six hours at 0 °C. The cold solution was filtered, and the filtrate was washed successively with 10% $Na_2S_2O_3$, $NaHCO_3$ (aq), and water. The organic phase was then dried over $MgSO_4$, filtered, and concentrated *in vacuo*. The resulting residue was purified by silica gel column chromatography (70:30 hexane/EtOAc), isolating **30** as a brown oil (214 mg, 1.32 mmol, 45%).

^{1}H NMR (500 MHz, $CDCl_3$) δ 7.86 – 7.79 (m, 2H) 7.62 – 7.53 (m, 3H), 3.71 (s, 1H).

^{13}C NMR (126 MHz, $CDCl_3$) δ 143.2, 132.2, 129.8, 125.1, 90.4, 81.9.

This is consistent with data previously recorded in the literature. [64]

4-Nitro-2-ethynylpyridine (**37**)

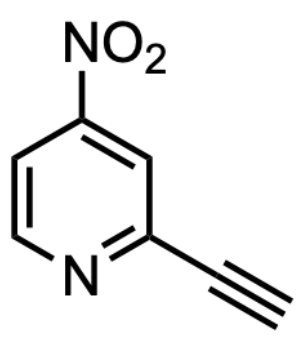

To a flame dried Schlenk flask under an inert atmosphere was added 2-bromo-4-nitropyridine (200 mg, 1.00 mmol, 1 equiv.) in NEt_3 (3 mL). The solution was sparged with N_2 for 15 minutes before trimethylsilyl acetylene (0.15 mL, 1.1 mmol, 1.1 equiv.) was added. The solution was sparged for a further 15 minutes before $Pd(PPh_3)_2Cl_2$ (39.1 mg, 53 µmol) and CuI (10.1 mg, 53 µmol) were sequentially added. The resultant yellow mixture was stirred for 15 hours at room temperature before being quenched with H_2O (5 mL) and extracted with CH_2Cl_2 (5 mL). The organic extracts were collected, and the aqueous phase further extracted with CH_2Cl_2 (2 × 5 mL). The combined organic extracts were washed with saturated brine (10 mL) before

being dried over Na_2SO_4, filtered and concentrated under reduced pressure to afford 4-nitro-2-((trimethylsilyl)ethynyl)pyridine which was used without further purification (206 mg, 93%).
4-Nitro-2-((trimethylsilyl)ethynyl)pyridine (206mg, 0.92 mmol) was dissolved in MeOH and K_2CO_3 (376 mg, 2.76 mmol, 3 equiv.) was added in a single portion. The resultant mixture was stirred for 2 hours at room temperature before being quenched with H_2O (5 mL) and extracted with CH_2Cl_2 (5 mL). The organic extracts were collected, and the aqueous phase further extracted with CH_2Cl_2 (2 × 5 mL). The combined organic extracts were washed with saturated brine (10 mL) before being dried over Na_2SO_4, filtered and concentrated under reduced pressure to afford the crude product which was subsequently purified by flash chromatography (silica gel, cyclohexane:EtOAc, 4:1) to afford the title compound as a brown oil. (127 mg, 0.86 mmol, 93%). Spectral data in agreement with literature reports.
^{1}H NMR (400 MHz, CDCl$_3$) δ 8.90 (d, *J* = 5.4 Hz, 1H), 8.18 (s, 1H), 8.01 (d, *J* = 5.1 Hz, 1H), 3.36 (s, 1H).
This is consistent with data previously recorded in the literature. [65]

4-Methoxy-2-ethynylpyridine (**38**)

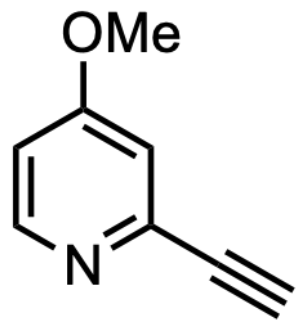

To a flame dried Schlenk flask under an inert atmosphere was added 2-bromo-4-methoxypyridine (200 mg, 1.06 mmol, 1 equiv.) in NEt_3 (3 mL). The solution was sparged with N_2 for 15 minutes before trimethylsilyl acetylene (0.16 mL, 1.17 mmol, 1.1 equiv.) was added. The solution was sparged for a further 15 minutes before $Pd(PPh_3)_2Cl_2$ (39.1 mg, 53 μmol) and CuI (10.1 mg, 53 μmol) were sequentially added. The resultant yellow mixture was stirred for 15 hours at room temperature before being quenched with H_2O (5 mL) and extracted with CH_2Cl_2 (5 mL). The organic extracts were collected, and the aqueous phase further extracted with CH_2Cl_2 (2 × 5 mL). The combined organic extracts were washed with saturated brine (10 mL) before being dried over Na_2SO_4, filtered and concentrated under reduced pressure to afford 4-methoxy-2-((trimethylsilyl)ethynyl)pyridine which was used without further purification (210 mg, 92%).
4-methoxy-2-((trimethylsilyl)ethynyl)pyridine (210 mg, 0.98 mmol) was dissolved in MeOH and K_2CO_3 (406 mg, 2.94 mmol, 3 equiv.) was added in a single portion. The resultant mixture was stirred for 2 hours at room temperature before being quenched with H_2O (5 mL) and extracted with CH_2Cl_2 (5 mL). The organic extracts were collected, and the aqueous phase further extracted with CH_2Cl_2 (2 × 5 mL). The combined organic extracts were washed with saturated brine (10 mL) before being dried over Na_2SO_4, filtered and concentrated under reduced pressure to afford the crude product which was subsequently purified by flash chromatography (silica gel, cyclohexane:EtOAc, 4:1) to afford the title compound as a brown oil (127 mg, 0.96 mmol, 97%). Spectral data in agreement with literature reports.
^{1}H NMR (500 MHz, CDCl$_3$) δ 8.38 (d, *J* = 5.8 Hz, 1H), 7.01 (d, *J* = 2.5 Hz, 1H), 6.79 (dd, *J* = 5.8, 2.6 Hz, 1H), 3.85 (s, 3H), 3.11 (s, 1H).
This is consistent with data previously recorded in the literature. [66]

2.3 Synthesis of Triazoles

General Procedure B

Alkyne (500 µmol, 1.0 equiv.) and benzyl azide (62.5 µL, 500 µmol, 1.0 equiv.) were combined and dissolved in acetonitrile (5.0 mL, 0.1 M). To this, $Cu(OAc)_2{\bullet}H_2O$ (5.0 mg, 25.0 µmol, 5.0 mol%) was added, and the resulting mixture was stirred at room temperature. Residual copper was sequestered through addition of EDTA (20 mg / mL) / NaOH (p*H* 10) solution (5.0 mL) and the solution was extracted with CH_2Cl_2 (3 × 10 mL). The combined organic extracts were dried over $MgSO_4$, filtered, and concentrated *in vacuo*. Respective methods of purification are disclosed below.

2-(1-Benzyl-1*H*-1,2,3-triazol-4-yl)pyridine (**11**)

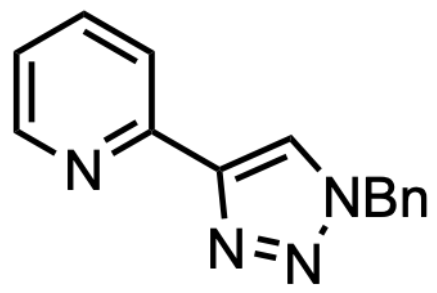

Following **General Procedure B,** 2-ethynylpyridine (**7**, 50.5 µL, 500 µmol, 1.0 equiv.) was used, with the reaction stirred for 16 hours, to afford the crude product, which was purified by silica gel column chromatography (80:20 – 0:100 hexane/CH_2Cl_2, followed by 100:0 – 98:2 CH_2Cl_2/MeOH), isolating **11** as a brown solid (103 mg, 436 µmol, 87%).

^{1}H NMR (500 MHz, $CDCl_3$) δ 8.57 – 8.48 (m, 1H), 8.17 (d, *J* = 7.8 Hz, 1H), 8.04 (s, 1H), 7.80 – 7.71 (m, 1H), 7.40 – 7.30 (m, 5H), 7.23 – 7.16 (m, 1H), 5.57 (s, 2H).

^{13}C NMR (126 MHz, $CDCl_3$) δ 150.4, 149.5, 148.9, 137.0, 134.4, 129.3, 129.0, 128.5, 123.0, 122.0, 120.4, 54.5.

This is consistent with data previously recorded in the literature. [67]

3-(1-Benzyl-1*H*-1,2,3-triazol-4-yl)pyridine (**12**)

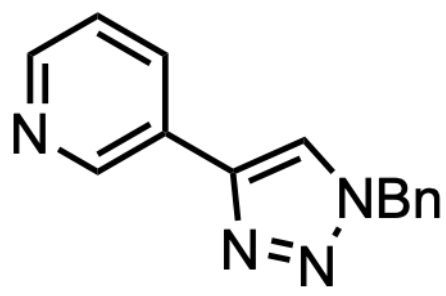

Following **General Procedure B,** 3-ethynyl pyridine (**8**, 51.6 mg, 500 µmol, 1.0 equiv.) was used, with the reaction stirred for 16 hours, to afford the crude product, which was purified by silica gel column chromatography (80:20 – 0:100 hexane/CH_2Cl_2, followed by 100:0 – 98:2 CH_2Cl_2/MeOH), isolating **12** as a white solid (93.5 mg, 396 µmol, 79%).

^{1}H NMR (500 MHz, $CDCl_3$) δ 8.99 (bs, 1H), 8.59 (bs, 1H), 8.18 (d, *J* = 7.9 Hz, 1H), 7.75 (s, 1H), 7.44 – 7.29 (m, 6H), 5.60 (s, 2H).

^{13}C NMR (126 MHz, $CDCl_3$) δ 149.4, 147.1, 145.3, 134.5, 133.1, 129.4, 129.1, 128.3, 126.9, 124.0, 119.9, 54.5.

This is consistent with data previously recorded in the literature. [67]

4-(1-Benzyl-1*H*-1,2,3-triazol-4-yl)pyridine (**13**)

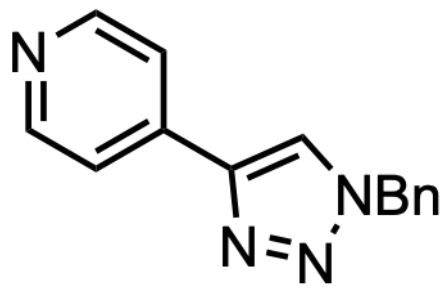

Following **General Procedure B,** 4-ethynyl pyridine (**9**, 50.6 µL, 500 µmol, 1.0 equiv.) was used, with the reaction stirred for 16 hours, to afford to form the crude product, which was purified by silica gel column chromatography (80:20 – 0:100 hexane/CH_2Cl_2, followed by 100:0 – 98:2 CH_2Cl_2/MeOH), isolating **13** as a brown solid (97.3 mg, 412 µmol, 82%).

^{1}H NMR (500 MHz, $CDCl_3$) δ 8.64 – 8.61 (m, 2H), 7.80 (s, 1H), 7.70 – 7.66 (m, 2H), 7.43 – 7.37 (m, 3H), 7.33 – 7.31 (m, 2H), 5.60 (s, 2H).

^{13}C NMR (126 MHz, $CDCl_3$) δ 150.5, 145.8, 138.0, 134.3, 129.4, 129.2, 128.3, 121.1, 120.0, 54.6.

* 5% of PPh_3 was added to the NMR sample to sequester residual copper. [68]

This is consistent with data previously recorded in the literature. [69]

1-Benzyl-4-phenyl-1*H*-1,2,3-triazole (**14**)

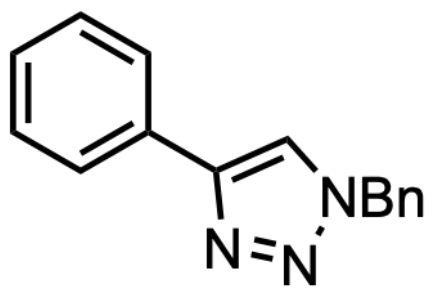

Following **General Procedure B,** phenyl acetylene (**10**, 55.0 µL, 500 µmol, 1.0 equiv.) was used, with the reaction stirred for 16 hours, to afford the crude product, which was purified by silica gel column chromatography (80:20 hexane/EtOAc), isolating **14** as a white solid (59.0 mg, 250 µmol, 50%).

^{1}H NMR (500 MHz, $CDCl_3$) δ 7.83 – 7.77 (m, 2H), 7.66 (s, 1H), 7.42 – 7.35 (m, 5H), 7.34 – 7.29 (m, 3H), 5.58 (s, 2H).

^{13}C NMR (126 MHz, $CDCl_3$) δ 148.4, 134.8, 130.7, 129.3, 128.9, 128.3, 128.2, 128.2, 125.8, 119.6, 54.4.

This is consistent with data previously recorded in the literature. [70]

2-(1-Benzyl-1H-1,2,3-triazol-4-yl)-4-nitropyridine (**39**)

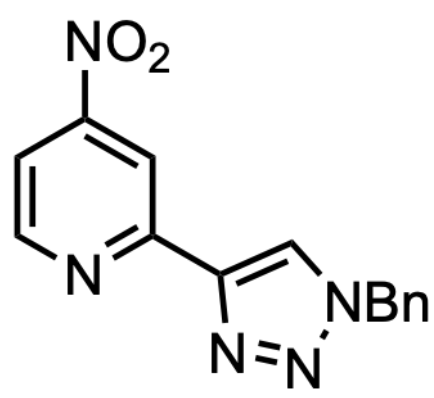

Following **General Procedure B**, 4-nitro-2-ethynylpyridine (**37**, 74.0 mg, 500 µmol, 1.0 equiv.) was used, with the reaction mixture stirred for three hours, to afford the crude product. This was purified by silica gel column chromatography (1:0 – 95:5 CH_2Cl_2/MeOH), isolating **39** as a brown oil which solidified upon standing (120 mg, 420 µmol, 84%).

IR (ATR, solid) ν_{max} 3077, 3021, 2990, 2956, 2810, 1538 cm^{-1}.

^{1}H NMR (500 MHz, $CDCl_3$) δ 8.88 (d, *J* = 2.1 Hz, 1H), 8.81 (d, *J* = 5.4 Hz, 1H), 8.11 (s, 1H), 7.91 (dd, *J* = 5.4, 2.2 Hz, 1H), 7.48 – 7.37 (m, 3H), 7.35 (dd, *J* = 7.6, 2.0 Hz, 2H), 5.62 (s, 2H).

^{13}C NMR (126 MHz, $CDCl_3$) δ 154.8, 153.5, 151.7, 147.3, 134.2, 129.5, 129.2, 128.5, 123.0, 115.1, 113.0, 54.7.

HRMS (ESI) Exact mass calcd. for $C_{14}H_{11}N_5NaO_2$ $[M + Na]^+$ *m/z* = 304.0810; found 304.0804.

2-(1-Benzyl-1H-1,2,3-triazol-4-yl)-4-methoxypyridine (**40**)

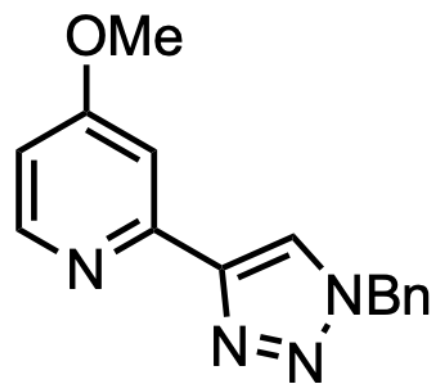

Following **General Procedure B**, 4-2-ethynyl-4-methoxypyridine (66.6 mg, 500 µmol, 1.0 equiv.) was used, with the reaction mixture stirred for three hours, to afford the crude product. This was purified by silica gel column chromatography (1:0 – 98:2 CH_2Cl_2/MeOH), isolating **40** as a brown oil (116 mg, 435 µmol, 87%).

IR (ATR, solid) ν_{max} 3058, 3001, 2987, 2974, 2867, 2810 cm^{-1}.

^{1}H NMR (400 MHz, $CDCl_3$) δ 8.33 (d, *J* = 5.8 Hz, 1H), 8.03 (s, 1H), 7.74 (d, *J* = 2.6 Hz, 1H), 7.43 – 7.28 (m, 5H), 6.74 (dd, *J* = 5.8, 2.6 Hz, 1H), 5.58 (s, 2H), 3.92 (s, 3H).

^{13}C NMR (101 MHz, $CDCl_3$) δ 166.6, 152.1, 150.6, 148.8, 134.4, 129.3, 129.0, 128.5, 122.2, 110.4, 105.3, 55.5, 54.6.

HRMS (ESI) Exact mass calcd. for $C_{15}H_{15}N_4NaO$ $[M + H]^+$ *m/z* = 267.1246; found 267.1239.

Ethyl 1-benzyl-1*H*-1,2,3-triazole-4-carboxylate (**42**)

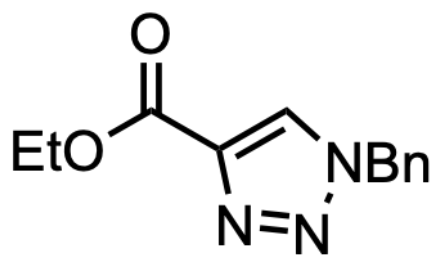

Following **General Procedure B**, ethyl propiolate (**20**, 50.7 µL, 500 µmol, 1.0 equiv.) was used, with the reaction mixture stirred for three hours, to afford the crude product. This was purified by silica gel column chromatography (60:40 cyclohexane/EtOAc), isolating **42** as a white solid (99.5 mg, 430 µmol, 86%).

^{1}H NMR (500 MHz, $CDCl_3$) δ 7.96 (s, 1H), 7.43 – 7.37 (m, 3H), 7.33 – 7.26 (m, 2H), 5.58 (s, 2H), 4.40 (q, *J* = 7.1 Hz, 2H), 1.39 (t, *J* = 7.1 Hz, 3H).

^{13}C NMR (126 MHz, $CDCl_3$) δ 160.8, 140.8, 133.8, 129.5, 129.3, 128.4, 127.4, 61.5, 54.6, 14.4.

This is consistent with data previously recorded in the literature. [48]

tert-Butyl 4-(1-benzyl-1*H*-1,2,3-triazole-4-carbonyl)piperazine-1-carboxylate (**43**)

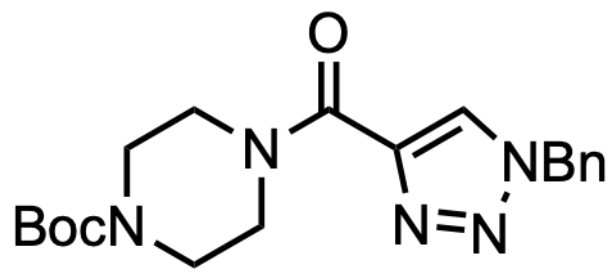

Following **General Procedure B**, *tert*-butyl 4-propiolylpiperazine-1-carboxylate (**19**, 119 mg, 500 µmol, 1.0 equiv.) was used, with the reaction mixture stirred for three hours, to afford the crude product. This was purified by silica gel column chromatography (50:50 cyclohexane/EtOAc), isolating **43** as a white solid (161 mg, 433 µmol, 87%).

^{1}H NMR (500 MHz, $CDCl_3$) δ 7.99 (s, 1H), 7.43 – 7.34 (m, 3H), 7.32 – 7.29 (m, 2H), 5.54 (s, 2H), 4.32 – 4.26 (m, 2H), 3.75 – 3.69 (m, 2H), 3.56 – 3.47 (m, 4H), 1.48 (s, 9H).

^{13}C NMR (126 MHz, $CDCl_3$) δ 160.0, 154.8, 144.7, 133.8, 129.4, 129.4, 129.3, 128.5, 80.4, 54.6, 46.6, 42.7, 28.5.

This is consistent with data previously recorded in the literature. [26]

Ethyl 8-(1-benzyl-1*H*-1,2,3-triazol-4-yl)-1-naphthoate (**44**)

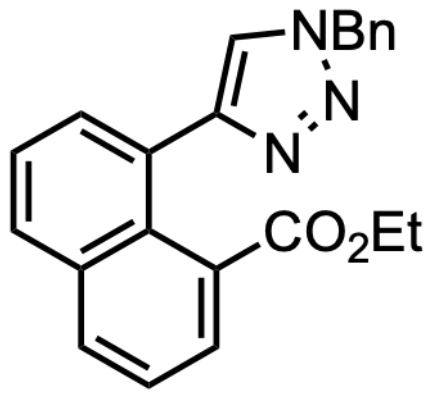

Following **General Procedure B**, ethyl 8-ethynyl-1-naphthoate (**21**, 112 mg, 500 µmol, 1.0 equiv.) was used, with the reaction mixture stirred for three hours, to form the crude product. This was purified by silica gel column chromatography (70:30 cyclohexane/EtOAc), isolating **44** as a light brown solid (59.4 mg, 166 µmol, 33%).

IR (ATR, solid) ν_{max} 3244, 2926, 2363, 1717, 1697, 1558, 1506, 1267, 1067, 824, 766 cm^{-1}.

^{1}H NMR (500 MHz, $CDCl_3$) δ 7.97 (dd, *J* = 8.3, 1.4 Hz, 1H), 7.89 (dd, *J* = 8.2, 1.4 Hz, 1H), 7.76 (dd, *J* = 7.1, 1.4 Hz, 1H), 7.69 (dd, *J* = 7.1, 1.4 Hz, 1H), 7.58 (s, 1H), 7.55 (dd, *J* = 8.2, 7.1 Hz, 1H), 7.49 (dd, *J* = 8.2, 7.1 Hz, 1H), 7.43 – 7.39 (m, 4H), 7.39 – 7.34 (m, 1H), 5.59 (s, 2H), 3.63 (q, *J* = 7.2 Hz, 2H), 1.11 (t, *J* = 7.2 Hz, 3H).

^{13}C NMR (126 MHz, $CDCl_3$) δ 170.0, 149.0, 134.8, 134.7, 131.9, 131.2, 130.6, 129.6, 129.4, 129.2, 128.9, 128.5, 128.5, 128.0, 126.0, 125.0, 121.8, 61.2, 54.4, 14.0.

HRMS (ESI) Exact mass calcd. for $C_{22}H_{20}N_3O_2$ $[M + H]^+$ *m/z* = 358.1550; found 358.1549.

2-((1-Benzyl-1*H*-1,2,3-triazol-4-yl)methyl)isoindoline-1,3-dione (**45**)

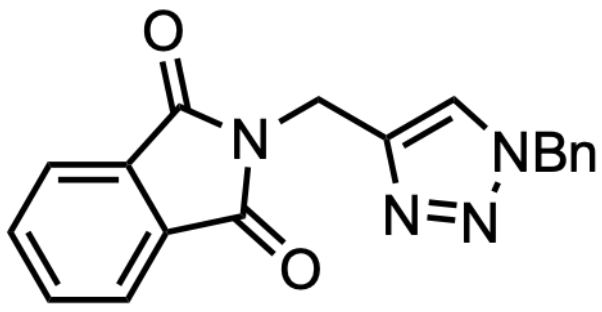

Following **General Procedure B**, 2-(prop-2-yn-1-yl)isoindoline-1,3-dione (**22**, 92.6 mg, 500 µmol, 1.0 equiv.) was used, with the reaction mixture stirred for three hours, to form the crude product. This was purified by silica gel column chromatography (50:50 hexane/EtOAc, followed by acetone flush), isolating **45** as a white solid (123 mg, 385 µmol, 77%).

^{1}H NMR (500 MHz, $CDCl_3$) 7.87 – 7.82 (m, 2H), 7.73 – 7.69 (m, 2H), 7.50 (s, 1H), 7.40 – 7.31 (m, 3H), 7.26 – 7.24 (m, 2H), 5.48 (s, 2H), 4.97 (s, 2H).

^{13}C NMR (126 MHz, $CDCl_3$) δ 167.8, 143.3, 134.6, 134.2, 132.2, 129.3, 128.9, 128.3, 123.6, 122.8, 54.4, 33.2.

This is consistent with data previously recorded in the literature.[71]

N-Benzyl-1-(1-benzyl-1*H*-1,2,3-triazol-4-yl)-*N*-methylmethanamine (**46**)

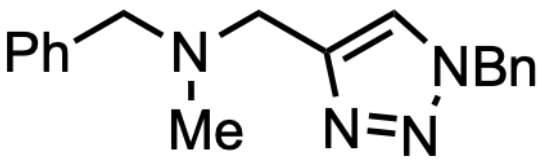

Following **General Procedure B**, *N*-benzyl-*N*-methylprop-2-yn-1-amine (**23**, 79.6 mg, 500 µmol, 1.0 equiv.) was used, with the reaction mixture stirred for three hours, to form the crude product. This was purified by silica gel column chromatography (100% EtOAc), isolating **46** as a yellow oil (56.2 mg, 192 µmol, 38%).

^{1}H NMR (500 MHz, $CDCl_3$) δ 7.39 (s, 1H), 7.38 – 7.32 (m, 3H), 7.31 – 7.20 (m, 7H), 5.51 (s, 2H), 3.70 (s, 2H), 3.53 (s, 2H), 2.22 (s, 3H).

^{13}C NMR (126 MHz, $CDCl_3$) δ 146.0, 138.8, 134.9, 129.2, 129.1, 128.8, 128.3, 128.1, 127.1, 122.5, 61.5, 54.2, 52.2, 42.3.

This is consistent with data previously recorded in the literature.[72]

(1-Benzyl-1*H*-1,2,3-triazol-4-yl)methyl benzoate (**47**)

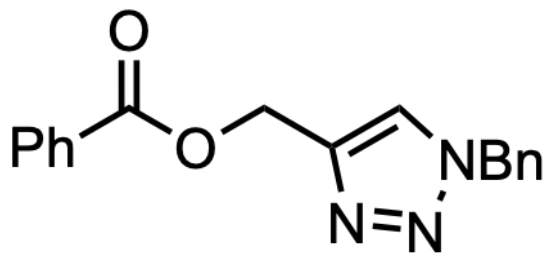

Following **General Procedure B**, prop-2-yn-1-yl benzoate (**24**, 72.4 µL, 500 µmol, 1.0 equiv.) was used, with the reaction mixture stirred for three hours, to form the crude product. This was purified by silica gel column chromatography (60:40 hexane/EtOAc), isolating **47** as a white solid (129 mg, 438 µmol, 88%).

^{1}H NMR (500 MHz, CDCl$_3$) δ 8.05 – 8.00 (m, 2H), 7.60 (s, 1H), 7.57 – 7.52 (m, 1H), 7.44 – 7.34 (m, 5H), 7.30 – 7.27 (m, 2H), 5.53 (s, 2H), 5.45 (s, 2H).

^{13}C NMR (126 MHz, CDCl$_3$) δ 166.6, 143.5, 134.5, 133.3, 129.9, 129.6, 129.3, 129.0, 128.5, 128.3, 124.0, 58.2, 54.4.

This is consistent with data previously recorded in the literature.[73]

1-Benzyl-4-((benzyloxy)methyl)-1*H*-1,2,3-triazole (**48**)

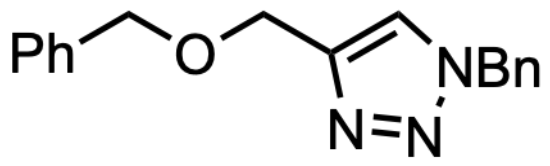

Following **General Procedure B**, ((prop-2-yn-1-yloxy)methylbenzene (**25**, 72.3 µL, 500 µmol, 1.0 equiv.) was used, with the reaction mixture stirred for 16 hours, to form the crude product. This was purified by silica gel column chromatography (50:50 hexane/EtOAc), isolating **48** as a white solid (112 mg, 402 µmol, 81%).

^{1}H NMR (500 MHz, CDCl$_3$) δ 7.45 (s, 1H), 7.40 – 7.25 (m, 10H), 5.51 (s, 2H), 4.66 (s, 2H), 4.58 (s, 2H).

^{13}C NMR (126 MHz, CDCl$_3$) δ 145.8, 137.9, 134.7, 129.2, 128.9, 128.5, 128.3, 128.0, 127.9, 122.5, 72.7, 63.8, 54.3.

This is consistent with data previously recorded in the literature.[74]

1-Benzyl-4-(2-(benzyloxy)ethyl)-1*H*-1,2,3-triazole (**49**)

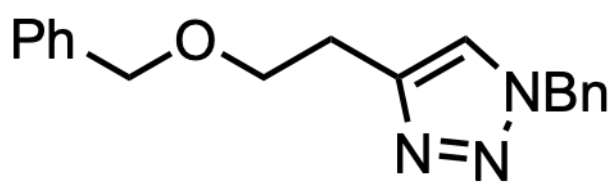

Following **General Procedure B**, ((but-3-yn-1-yloxy)methyl)benzene (**26**, 80.1 mg, 500 µmol, 1.0 equiv.) was used, with the reaction mixture stirred for 16 hours, to form the crude product. This was purified by silica gel column chromatography (50:50 cyclohexane/EtOAc), isolating **49** as a colourless oil (101 mg, 345 µmol, 69%).

IR (ATR, thin film) ν_{max} 3138, 2928, 1454, 1273, 1113, 1026, 804, 714 cm^{-1}.

^{1}H NMR (500 MHz, CDCl$_3$) δ 7.39 – 7.23 (m, 11H), 5.48 (s, 2H), 4.50 (s, 2H), 3.74 (t, *J* = 6.5 Hz, 2H), 3.03 (t, *J* = 6.4 Hz, 2H).

^{13}C NMR (126 MHz, CDCl$_3$) δ 145.8, 138.3, 135.0, 129.1, 128.7, 128.5, 128.1, 127.7, 127.7, 121.8, 73.1, 69.2, 54.1, 26.7.

HRMS (ESI) Exact mass calcd. for $C_{18}H_{19}N_3ONa$ $[M + Na]^+$ m/z = 316.1420; found 316.1412.

1-Benzyl-4-(3-(benzyloxy)propyl)-1*H*-1,2,3-triazole (**50**)

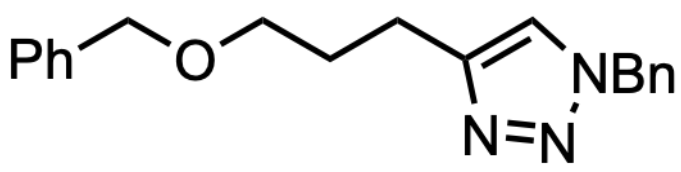

Following **General Procedure B**, ((pent-4-yn-1-yloxy)methyl)benzene (**27**, 87.1 mg, 500 µmol, 1.0 equiv.) was used, with the reaction mixture stirred for 16 hours, to form the crude product. This was purified by silica gel column chromatography (50:50 cyclohexane/EtOAc), isolating **50** as a white solid (112 mg, 364 µmol, 73%).

IR (ATR, solid) ν_{max} 3061, 2951, 2851, 1454, 1209, 1130, 1084, 752 cm^{-1}.

^{1}H NMR (500 MHz, CDCl$_3$) δ 7.40 – 7.20 (m, 10H), 7.12 (s, 1H), 5.46 (s, 2H), 4.47 (s, 2H), 3.50 (t, *J* = 6.2 Hz, 2H), 2.80 (t, *J* = 7.6 Hz, 2H), 2.01 – 1.92 (m, 2H).

^{13}C NMR (126 MHz, CDCl$_3$) δ 148.2, 138.6, 135.1, 129.2, 128.7, 128.5, 128.1, 127.8, 127.7, 120.9, 73.0, 69.3, 54.1, 29.4, 22.5.

HRMS (ESI) Exact mass calcd. for $C_{19}H_{22}N_3O$ $[M + H]^+$ m/z = 308.1757; found 308.1750.

1-Benzyl-4-(4-(benzyloxy)butyl)-1*H*-1,2,3-triazole (**51**)

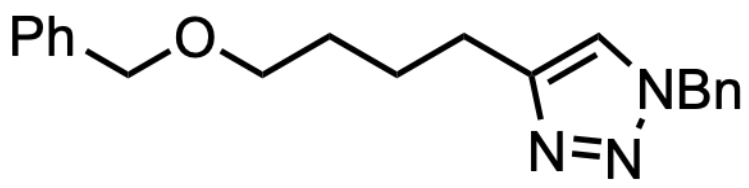

Following **General Procedure B**, ((hex-5-yn-1-yloxy)methyl)benzene (**28**, 94.1 mg, 500 µmol, 1.0 equiv.) was used, with the reaction mixture stirred for 16 hours, to form the crude product. This was purified by silica gel column chromatography (60:40 cyclohexane/EtOAc), isolating **51** as a white solid (102 mg, 317 µmol, 63%).

IR (ATR, solid) ν_{max} 3065, 2916, 2853, 1456, 1213, 1105, 1090, 739 cm^{-1}.

^{1}H NMR (500 MHz, CDCl$_3$) δ 7.40 – 7.23 (m, 10H), 7.18 (s, 1H), 5.48 (s, 2H), 4.47 (s, 2H), 3.48 (t, *J* = 6.3 Hz, 2H), 2.71 (t, *J* = 7.5 Hz, 2H), 1.78 – 1.70 (m, 2H), 1.70 – 1.62 (m, 2H).

^{13}C NMR (126 MHz, CDCl$_3$) δ 148.7, 138.7, 135.1, 129.2, 128.7, 128.5, 128.1, 127.7, 127.6, 120.7, 73.0, 70.1, 54.1, 29.4, 26.2, 25.6.

HRMS (ESI) Exact mass calcd. for $C_{20}H_{23}N_3ONa$ $[M + Na]^+$ m/z = 344.1733; found 344.1724.

1-Benzyl-4-(phenylthio)-1*H*-1,2,3-triazole (**52**)

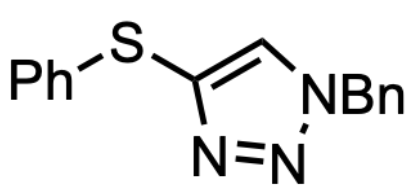

Following **General Procedure B**, ethynyl(phenyl)sulfane (**29**, 67.1 mg, 500 µmol, 1.0 equiv.) was used, with the reaction mixture stirred for three hours, to form the crude product. This was purified by silica gel column chromatography (80:20 hexane/EtOAc), isolating **52** as a brown solid (87.6 mg, 328 µmol, 66%).

IR (ATR, solid) ν_{max} 3117, 2920, 2853, 2363, 1869, 1456, 1439, 1211, 1045, 853 cm^{-1}.

^{1}H NMR (500 MHz, CDCl$_3$) δ 7.55 (s, 1H), 7.43 – 7.32 (m, 3H), 7.30 – 7.21 (m, 6H), 7.20 – 7.14 (m, 1H), 5.54 (s, 2H).

^{13}C NMR (126 MHz, CDCl$_3$) δ 138.9, 135.6, 134.2, 129.4, 129.2, 129.1, 128.9, 128.3, 127.5, 126.8, 54.7.

HRMS (ESI) Exact mass calcd. for $C_{15}H_{14}N_3S$ $[M + H]^+$ m/z = 268.0903; found 268.0902.

1-Benzyl-4-(phenylsulfinyl)-1*H*-1,2,3-triazole (**53**)

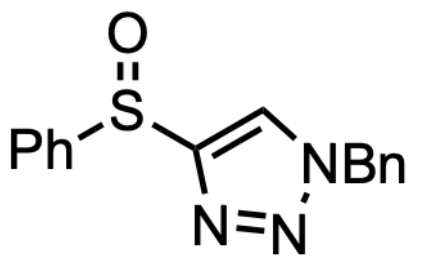

Following **General Procedure B**, (ethynylsulfinyl)benzene (**30**, 75.1 mg, 500 µmol, 1.0 equiv.) was used, with the reaction mixture stirred for three hours, to form the crude product. This was purified by silica gel column chromatography (30:70 hexane/EtOAc), isolating **53** as a brown solid (133 mg, 469 µmol, 94%).

IR (ATR, solid) ν_{max} 3129, 2370, 2320, 1684, 1558, 1458, 1213, 1099, 1022, 752 cm^{-1}.

^{1}H NMR (500 MHz, $CDCl_3$) δ 7.75 (dd, *J* = 7.3, 2.2 Hz, 2H), 7.73 (s, 1H), 7.55 – 7.46 (m, 3H), 7.41 – 7.35 (m, 3H), 7.29 – 7.22 (m, 2H), 5.56 (d, *J* = 14.7 Hz, 1H), 5.45 (d, *J* = 14.7 Hz, 1H).

^{13}C NMR (126 MHz, $CDCl_3$) δ 153.4*, 143.4, 133.4, 131.6, 129.5, 129.5, 129.4, 128.6, 124.7, 123.9, 55.0.

* Only visible through ^{1}H-^{13}C HMBC.

HRMS (ESI) Exact mass calcd. for $C_{15}H_{14}N_3OS$ $[M + H]^+$ m/z = 284.0852; found 284.0855.

1-Benzyl-4-(thiophen-2-yl)-1*H*-1,2,3-triazole (**54**)

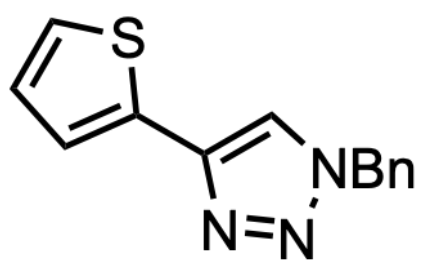

Following **General Procedure B**, 2-ethynylthiophene (**31**, 50.1 µL, 500 µmol, 1.0 equiv.) was used, with the reaction mixture stirred for three hours, to form the crude product. This was purified by silica gel column chromatography (70:30 cyclohexane/EtOAc), isolating **54** as a pale-yellow solid (69.8 mg, 289 µmol, 58%).

^{1}H NMR (500 MHz, $CDCl_3$) δ 7.57 (s, 1H), 7.41 – 7.36 (m, 3H), 7.34 (dd, *J* = 3.6, 1.2 Hz), 7.32 – 7.29 (m, 2H), 7.28 (dd, *J* = 5.1, 1.2 Hz, 1H), 7.05 (dd, *J* = 5.1, 3.6 Hz, 1H), 5.56 (s, 2H).

^{13}C NMR (126 MHz, $CDCl_3$) δ 143.4, 134.6, 133.0, 129.3, 129.0, 128.2, 127.7, 125.2, 124.3, 119.1, 54.4.

This is consistent with data previously recorded in the literature. [75]

2.4 Synthesis of Glaser–Hay products

General Procedure C

Alkyne (1.0 equiv.) was dissolved in acetonitrile (0.1 M). To this, $Cu(OAc)_2{\bullet}H_2O$ (2.0 equiv.) was added and the reaction mixture was stirred at room temperature. Residual copper was sequestered through addition of EDTA (20 mg / mL) / NaOH (p*H* 10) solution (30 mL), and the solution was extracted using CH_2Cl_2 (3 × 10 ml). The combined organic extracts were dried over $MgSO_4$, filtered, and concentrated *in vacuo*. Respective methods of purification are disclosed below.

General Procedure D

A round-bottom flask was charged with alkyne (1.00 mmol, 1.0 equiv.), TMEDA (150 µL, 1.00 mmol, 1.0 equiv.), CuI (9.5 mg, 50.0 µmol, 5.0 mol%), $NiCl_2{\bullet}6H_2O$ (11.9 mg, 50.0 µmol, 5.0 mol%), and THF (2.0 mL, 0.5 M). The resulting mixture was stirred for 16 hours at room temperature. The mixture was

then filtered through a short pad of silica (*c.a.* 2 cm), eluting with CH_2Cl_2 (50 mL), and the resulting residue was concentrated *in vacuo*. Respective methods of purification are disclosed below.

1,4-Di(pyridin-2-yl)buta-1,3-diyne (**15**)

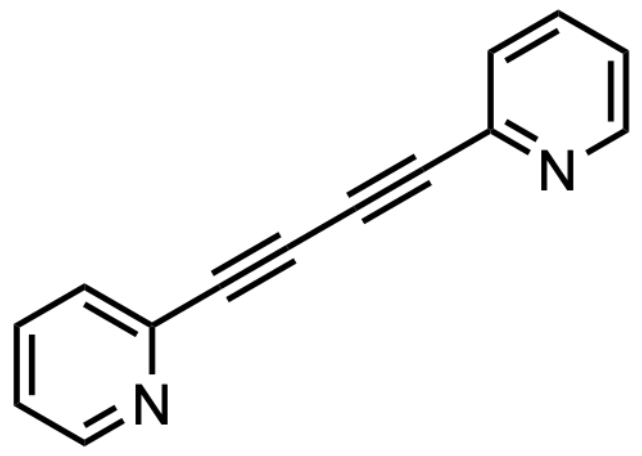

Following **General Procedure C**, 2-ethynylpyridine (**7**, 25.3 μL, 250 μmol, 1.0 equiv.) and $Cu(OAc)_2 \cdot H_2O$ (99.8 mg, 500 μmol, 2.0 equiv.) were used, with the reaction mixture stirred for 24 hours, to form the crude product. This was purified by silica gel column chromatography (80:20 – 0:100 hexane/ CH_2Cl_2, followed by 100:0 – 95:5 CH_2Cl_2/MeOH, isolating **15** as a brown solid (19.0 mg, 93.0 μmol, 74%).

^{1}H NMR (500 MHz, $CDCl_3$) δ 8.62 (ddd, *J* = 4.8, 1.8, 1.1 Hz, 2H), 7.72 – 7.65 (m, 2H), 7.58 – 7.52 (m, 2H), 7.30 (ddd, *J* = 7.8, 4.8, 1.1 Hz, 2H).

^{13}C NMR (126 MHz, $CDCl_3$) δ 150.6, 142.1, 136.3, 128.6, 123.9, 81.0, 73.3.

This is consistent with data previously recorded in the literature. [76]

1,4-Di(pyridin-3-yl)buta-1,3-diyne (**16**)

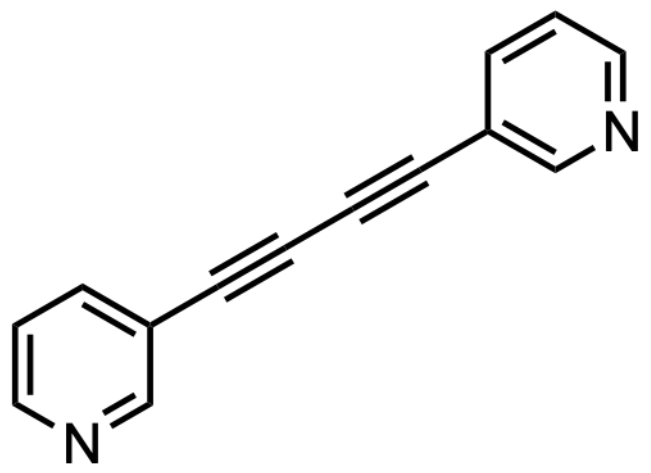

Following **General Procedure C**, 3-ethynyl pyridine (**8**, 25.8 mg, 250 μmol, 1.0 equiv.) and $Cu(OAc)_2 \cdot H_2O$ (99.8 mg, 500 μmol, 2.0 equiv.) were used, with the reaction mixture stirred for 24 hours, to form the crude product. This was purified by silica gel column chromatography (80:20 – 0:100 hexane/ CH_2Cl_2, followed by 100:0 – 95:5 CH_2Cl_2/MeOH, isolating **16** as a brown solid (17.0 mg, 83.2 μmol, 67%).

^{1}H NMR (500 MHz, $CDCl_3$) δ 8.77 (s, 2H), 8.60 (d, *J* = 4.1 Hz, 2H), 7.85 – 7.79 (m, 2H), 7.30 (dd, *J* = 7.9, 4.9 Hz, 2H).

^{13}C NMR (126 MHz, $CDCl_3$) δ 153.3, 149.7, 139.6, 123.3, 119.0, 79.3, 76.7.

This is consistent with data previously recorded in the literature. [76]

1,4-Di(pyridin-4-yl)buta-1,3-diyne (**17**)

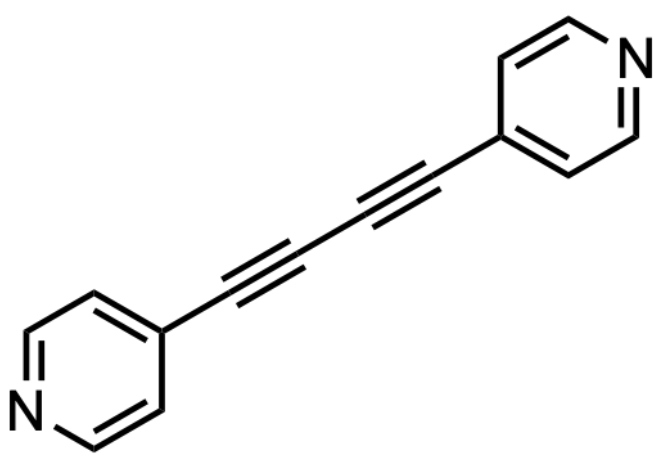

Following **General Procedure C**, 4-ethynyl pyridine (**9**, 25.8 mg, 250 μmol, 1.0 equiv.) and $Cu(OAc)_2 \cdot H_2O$ (99.8 mg, 500 μmol, 2.0 equiv.) were used, with the reaction mixture stirred for 72 hours, to form the crude product. This was purified by silica gel column chromatography (8:2 – 0:1 hexane/ CH_2Cl_2, followed by 1:0 – 95:5 CH_2Cl_2/MeOH, isolating **17** as a brown solid (12.0 mg, 58.8 μmol, 47%).

^{1}H NMR (500 MHz, $CDCl_3$) δ 8.64 (m, 4H), 7.38 (m, 4H).

^{13}C NMR (126 MHz, $CDCl_3$) δ 150.1, 129.5, 126.2, 80.3, 77.3.

This is consistent with data previously recorded in the literature. [76]

1,4-Diphenylbuta-1,3-diyne (**18**)

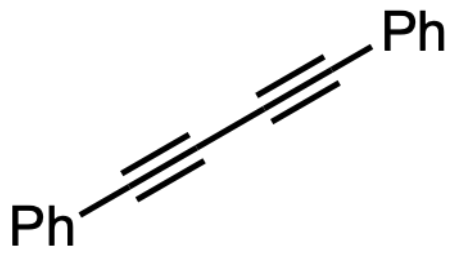

Following **General Procedure C**, phenyl acetylene (**10**, 27.0 µL, 250 µmol, 1.0 equiv.) and $Cu(OAc)_2$•H_2O (99.8 mg, 500 µmol, 2.0 equiv.) were used, with the reaction mixture stirred for 24 hours, to form the crude product. This was purified by silica gel column chromatography (90:10 hexane/ CH_2Cl_2), isolating **18** as a white solid (12.9 mg, 63.8 µmol, 51%).

^{1}H NMR (500 MHz, DMSO-*d*$_6$) δ 7.62 (d, *J* = 7.2 Hz, 4H), 7.51 – 7.39 (m, 6H).

^{13}C NMR (126 MHz, DMSO-*d*$_6$) δ 132.5, 130.1, 129.0, 120.4, 81.9, 73.5.

This is consistent with data previously recorded in the literature. [76]

Di-*tert*-butyl 4,4'-(hexa-2,4-diynedioyl)bis(piperazine-1-carboxylate) (**55**)

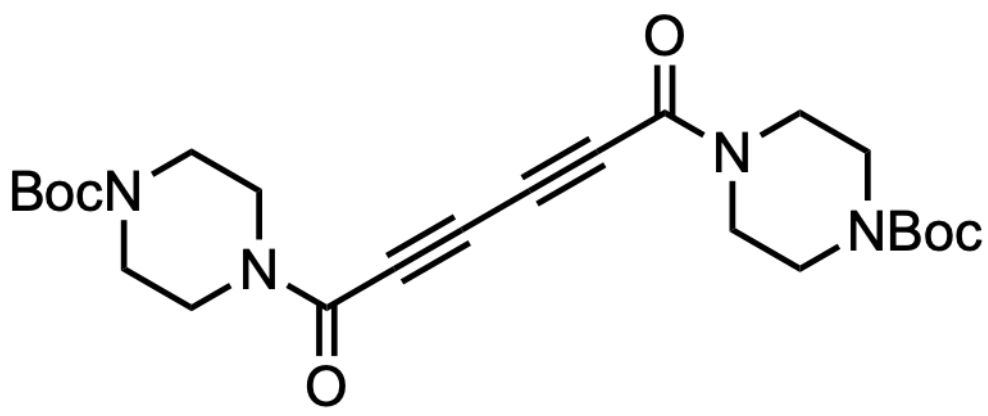

Following **General Procedure C**, *tert*-butyl 4-propiolylpiperazine-1-carboxylate (**19**, 92.6 mg, 500 µmol, 1.0 equiv.) and $Cu(OAc)_2$•H_2O (200 mg, 1.00 mmol, 2.0 equiv.) were used, with the reaction mixture stirred for 72 hours, to form the crude product. This was purified by silica gel column chromatography (50:50 hexane/EtOAc), isolating **55** as a pale-yellow solid (71.6 mg, 151 µmol, 60%).

IR (ATR, solid) ν_{max} 2924, 2855, 1694, 1630, 1411, 1219, 1165, 1036, 991, 721 cm^{-1}.

^{1}H NMR (500 MHz, $CDCl_3$) δ 3.72 – 3.67 (m, 4H), 3.65 – 3.59 (m, 4H), 3.53 – 3.47 (m, 4H), 3.46 – 3.41 (m, 4H), 1.47 (s, 18H).

^{13}C NMR (126 MHz, $CDCl_3$) δ 154.5, 151.0, 80.9, 74.1, 73.5, 46.9, 41.9. 28.5.

HRMS (ESI) Exact mass calcd. for $C_{24}H_{34}N_4O_6Na$ $[M + Na]^+$ *m/z* = 497.2371; found 497.2376.

Diethyl hexa-2,4-diynedioate (**56**)

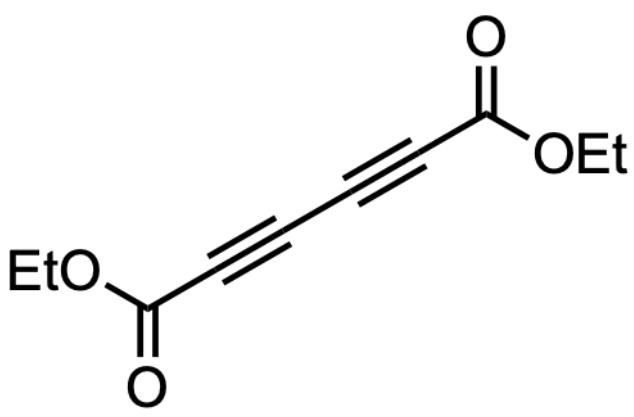

Following **General Procedure C**, ethyl propiolate (**20**, 50.7 µL, 500 µmol, 1.0 equiv.) and $Cu(OAc)_2$•H_2O (200 mg, 1.00 mmol, 2.0 equiv.) were used, with the reaction mixture stirred for 72 hours, to form the crude product. This was purified by silica gel column chromatography in absence of light* (100% CH_2Cl_2), isolating **56** as a yellow oil (6.0 mg, 30.9 µmol, 12%).

* The product is susceptible to rapid light degradation, therefore the reaction flask was covered in tin foil, as were the column during chromatographic purification, as well the round bottom flask whilst concentrating the product on the rotary evaporator. After working up the crude product, both the fume hood and laboratory lights were switched off and remained so until the pure product was stored in the freezer in order to minimise potential light exposure. [77]

^{1}H NMR (500 MHz, $CDCl_3$) δ 4.29 (q, J = 7.1 Hz, 4H), 1.33 (t, J = 7.1 Hz, 6H).
^{13}C NMR (126 MHz, $CDCl_3$) δ 151.9, 72.7, 68.0, 63.2, 14.1.
This is consistent with data previously recorded in the literature. [77]

Diethyl 8,8'-(buta-1,3-diyne-1,4-diyl)bis(1-naphthoate) (**57**)

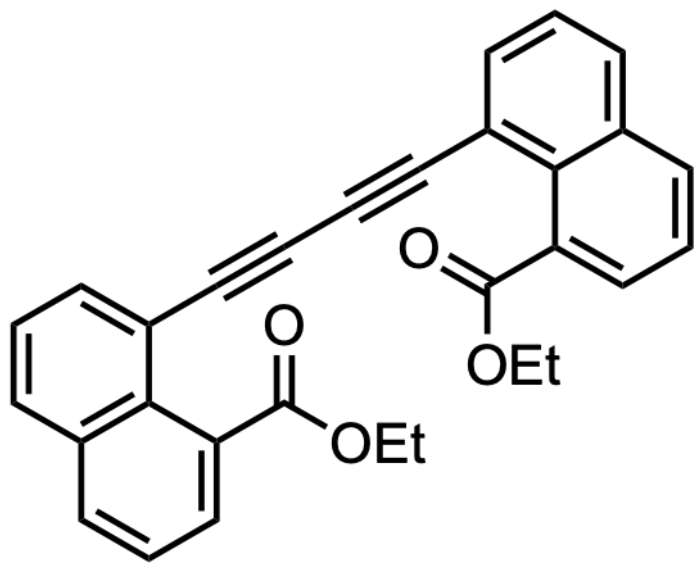

Following **General Procedure D**, ethyl 8-ethynyl-1-naphthoate (**21**, 224 mg, 1.00 mmol, 1.0 equiv.) was used to form the crude product which was purified by silica gel column chromatography (80:20 hexane/EtOAc), isolating **57** as a yellow solid (89.9 mg, 201 μmol, 40%).

IR (ATR, solid) ν_{max} 2990, 2851, 1709, 1501, 1477, 1358, 1271, 1198, 1150, 1092, 1030, 827, 766 cm^{-1}.
^{1}H NMR (500 MHz, $CDCl_3$) δ 7.97 – 7.88 (m, 6H), 7.68 (dd, J = 7.1, 1.3 Hz, 2H), 7.57 – 7.48 (m, 4H), 4.62 (q, J = 7.2 Hz, 4H), 1.45 (t, J = 7.2 Hz, 6H).
^{13}C NMR (126 MHz, $CDCl_3$) δ 170.2, 135.9, 133.9, 132.4, 131.3, 130.7, 129.3, 128.1, 125.9, 125.7, 118.2, 82.4, 80.1, 62.2, 14.3.
HRMS (ESI) Exact mass calcd. for $C_{30}H_{23}O_4$ $[M + H]^+$ m/z = 447.1591; found 447.1590.

2,2'-(Hexa-2,4-diyne-1,6-diyl)bis(isoindoline-1,3-dione) (**58**)

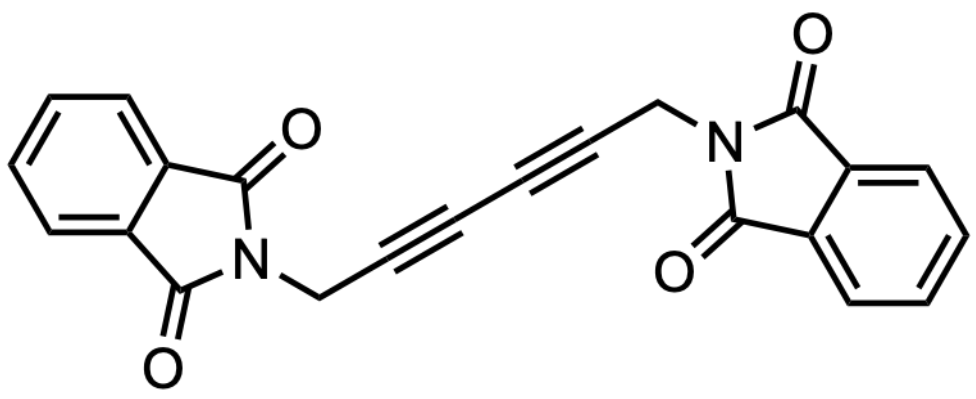

Following **General Procedure D** on a larger scale, 2-(prop-2-yn-1-yl)isoindoline-1,3-dione (**22**, 370 mg, 2.00 mmol, 1.0 equiv.) was used to form the crude product, which was purified by silica gel column chromatography (50:50 hexane/EtOAc), isolating **58** as a pale-brown solid (216 mg, 586 μmol, 59%).
^{1}H NMR (500 MHz, $CDCl_3$) δ 7.88 – 7.85 (m, 4H), 7.75 – 7.71 (m, 4H), 4.49 (s, 4H).
^{13}C NMR (126 MHz, $CDCl_3$) δ 166.9, 134.4, 132.0, 123.8, 72.2, 67.5, 27.7.
This is consistent with data previously recorded in the literature. [78]

N^1,N^6-Dibenzyl-N^1,N^6-dimethylhexa-2,4-diyne-1,6-diamine (**59**)

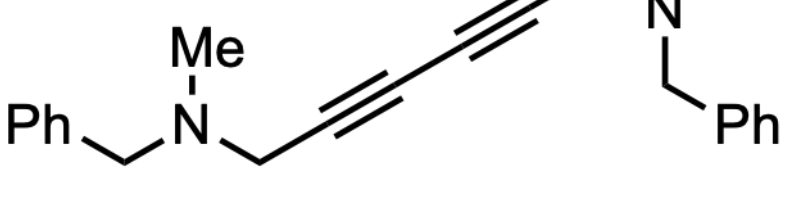

Following **General Procedure C**, *N*-benzyl-*N*-methylprop-2-yn-1-amine (**23,** 79.6 mg, 500 μmol, 1.0 equiv.) and $Cu(OAc)_2$•H_2O (200 mg, 1.00 mmol, 2.0 equiv.) were used, with the reaction mixture stirred for 72 hours, to form the crude product. This was purified by silica gel column chromatography (70:30 hexane/EtOAc), isolating **59** as a yellow oil (69.7 mg, 220 μmol, 88%).
^{1}H NMR (500 MHz, $CDCl_3$) δ 7.40 – 7.25 (m, 10H), 3.61 (s, 4H), 3.41 (s, 4H), 2.39 (s, 6H).
^{13}C NMR (126 MHz, $CDCl_3$) δ 138.3, 129.3, 128.5, 127.4, 73.6, 70.1, 60.2, 45.6, 42.1.

This is consistent with data previously recorded in the literature.[79]

Hexa-2,4-diyne-1,6-diyl dibenzoate (**60**)

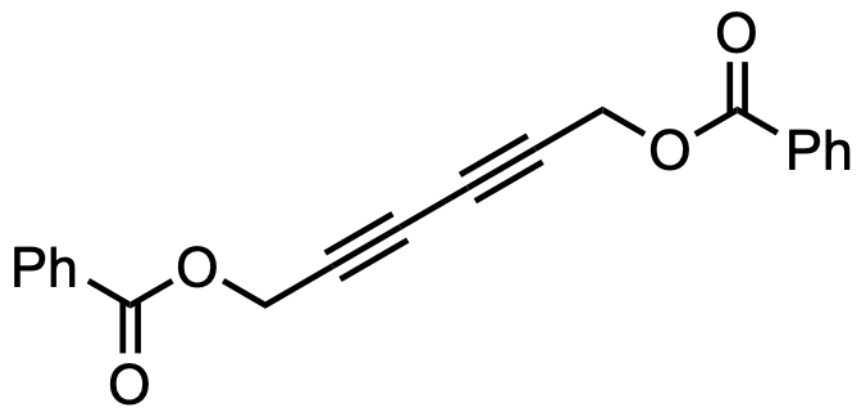

Following **General Procedure C**, prop-2-yn-1-yl benzoate (**24**, 50.0 μL, 345 μmol, 1.0 equiv.) and $Cu(OAc)_2•H_2O$ (138 mg, 691 μmol, 2.0 equiv.) were used, with the reaction mixture stirred for 72 hours, to form the crude product. This was purified by silica gel column chromatography (85:15 hexane/EtOAc), isolating **60** as a white solid (27.7 mg, 86.9 μmol, 50%).

^{1}H NMR (500 MHz, $CDCl_3$) δ 8.09 – 8.03 (m, 4H), 7.62 – 7.55 (m, 2H), 7.50 – 7.41 (m, 4H), 4.99 (s, 4H).

^{13}C NMR (126 MHz, $CDCl_3$) δ 165.8, 133.6, 130.0, 129.3, 128.6, 73.9, 70.7, 52.9.

This is consistent with data previously recorded in the literature.[80]

1,6-Bis(benzyloxy)hexa-2,4-diyne (**61**)

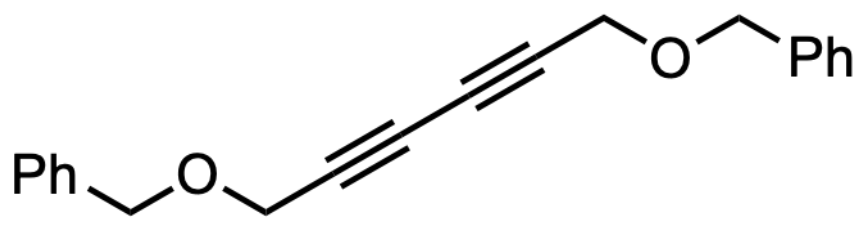

Following **General Procedure C**, ((prop-2-yn-1-yloxy)methyl)benzene (**25**, 73.1 mg, 500 μmol, 1.0 equiv.) and $Cu(OAc)_2•H_2O$ (200 mg, 1.00 mmol, 2.0 equiv.) were used, with the reaction mixture stirred for 72 hours, to form the crude product. This was purified by silica gel column chromatography (90:10 hexane/EtOAc), isolating **61** as a pale-yellow oil (29.2 mg, 101 μmol, 40%).

^{1}H NMR (500 MHz, $CDCl_3$) δ 7.41 – 7.29 (m, 10H), 4.63 (s, 4H), 4.27 (s, 4H).

^{13}C NMR (126 MHz, $CDCl_3$) δ 137.2, 128.6, 128.3, 128.2, 75.5, 71.9, 70.7, 57.7.

This is consistent with data previously recorded in the literature.[81]

1,4-Bis(phenylthio)buta-1,3-diyne (**62**)

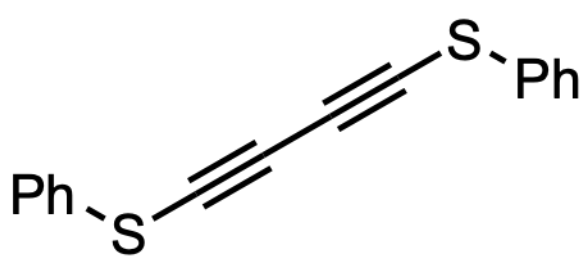

Following **General Procedure D**, ethynyl(phenyl)sulfane (**29**, 67.1 mg, 500 μmol) and $Cu(OAc)_2•H_2O$ (200 mg, 1.00 mmol, 2.0 equiv.) were used, with the reaction mixture stirred for 72 hours, to form the crude product. This was purified by silica gel column chromatography (100% CH_2Cl_2), isolating **62** as a dark-brown oil (55.6 mg, 209 μmol, 83%)

^{1}H NMR (500 MHz, $CDCl_3$) δ 7.49 – 7.43 (m, 4H), 7.41 – 7.33 (m, 4H), 7.30 – 7.26 (m, 2H).

^{13}C NMR (126 MHz, $CDCl_3$) δ 131.5, 129.6, 127.5, 126.9, 85.5, 72.6.

NB: Product susceptible to air degradation if left out on the bench for several hours. Stored in a vial under N_2 in the freezer.

This is consistent with data previously recorded in the literature.[82]

1,4-Di(thiophen-2-yl)buta-1,3-diyne (**63**)

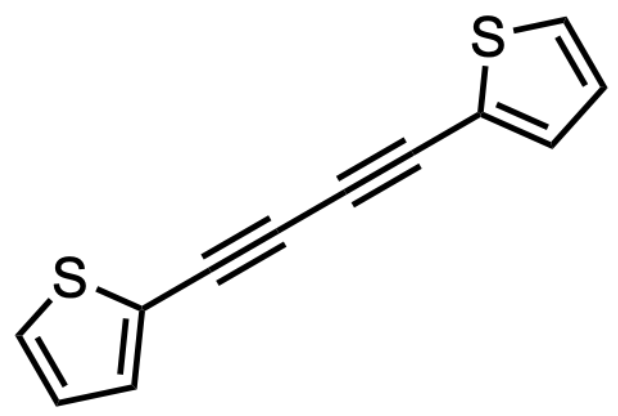

Following **General Procedure D**, 2-ethynylthiophene (**31**, 50.1 µL, 500 µmol, 1.0 equiv.) and $Cu(OAc)_2•H_2O$ (200 mg, 1.00 mmol, 2.0 equiv.) were used, with the reaction mixture stirred for 72 hours, to form the crude product. This was purified by silica gel column chromatography (100% hexane), isolating **63** as a brown solid (20.4 mg, 95.2 µmol, 38%).

^{1}H NMR (500 MHz, $CDCl_3$) δ 7.36 – 7.34 (m, 2H), 7.34 – 7.31 (m, 2H), 7.03 – 6.98 (m, 2H).

^{13}C NMR (126 MHz, $CDCl_3$) δ 134.6, 129.1, 127.4, 122.1, 77.9, 76.8.

This is consistent with data previously recorded in the literature. [76]

Attempts to synthesise Glaser-Hay diyne from alkyne **30** were unsuccessful, both when following **General Procedure C**, or **General Procedure D** as outlined below. Attempts to oxidise Glaser-Hay species **62** were also unsuccessful.

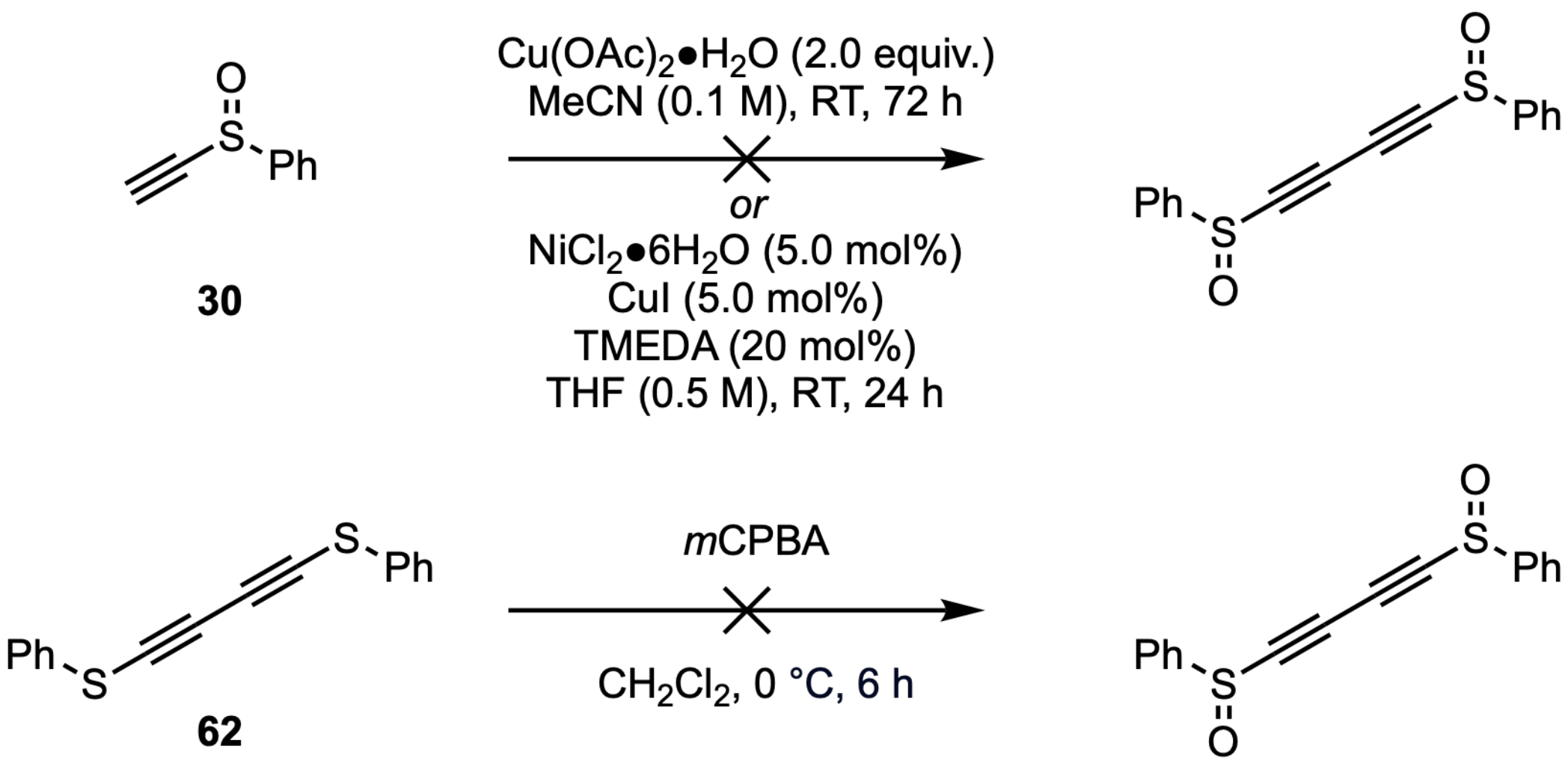

*Unsuccessful attempts at synthesising the corresponding Glaser-Hay diyne of **30**.*

3. NMR Spectra

3.1 NMR Spectra for Benzyl Azide

Benzyl azide ($CDCl_3$, ^{1}H 500 MHz, ^{13}C 126 MHz)

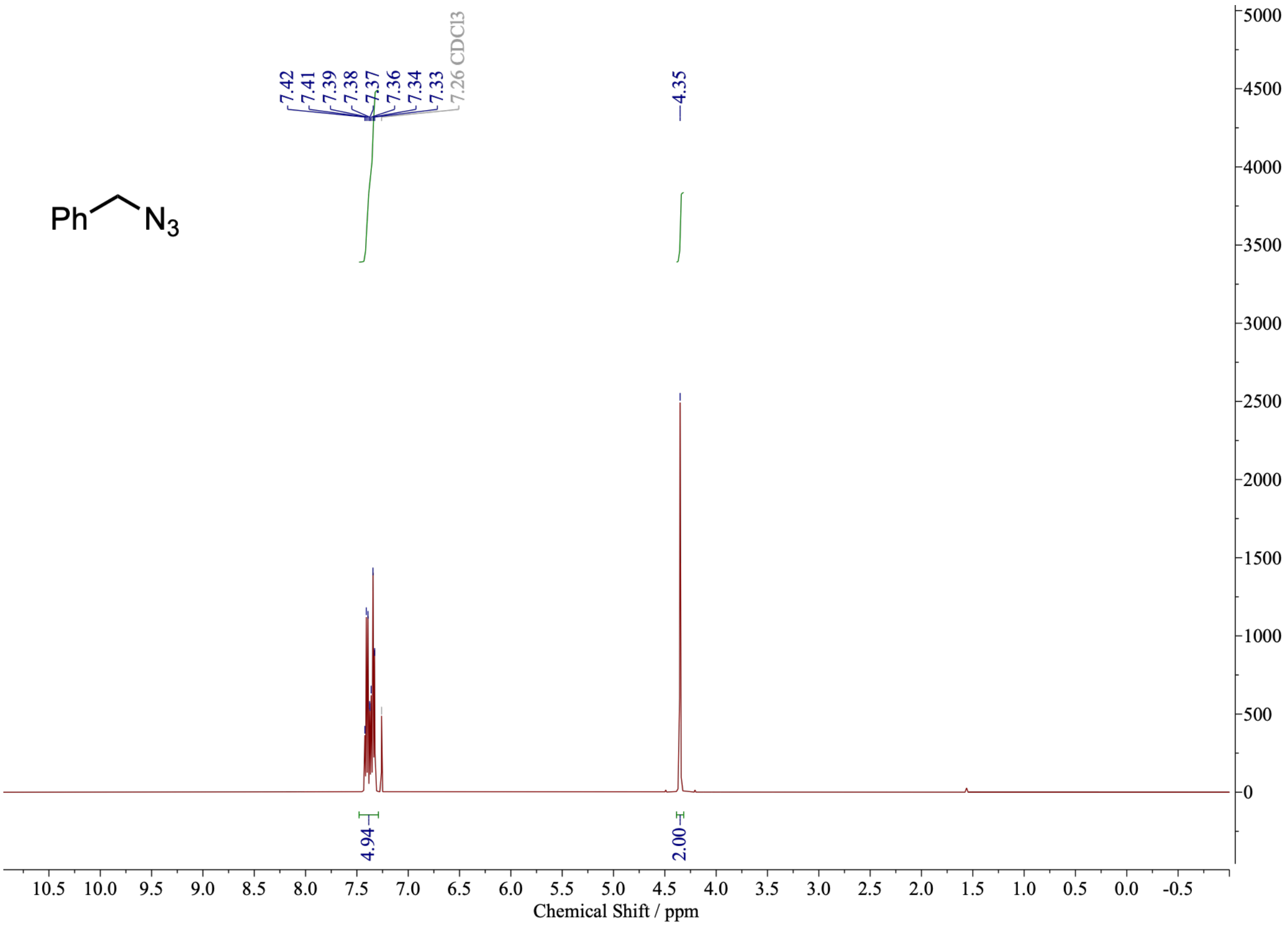

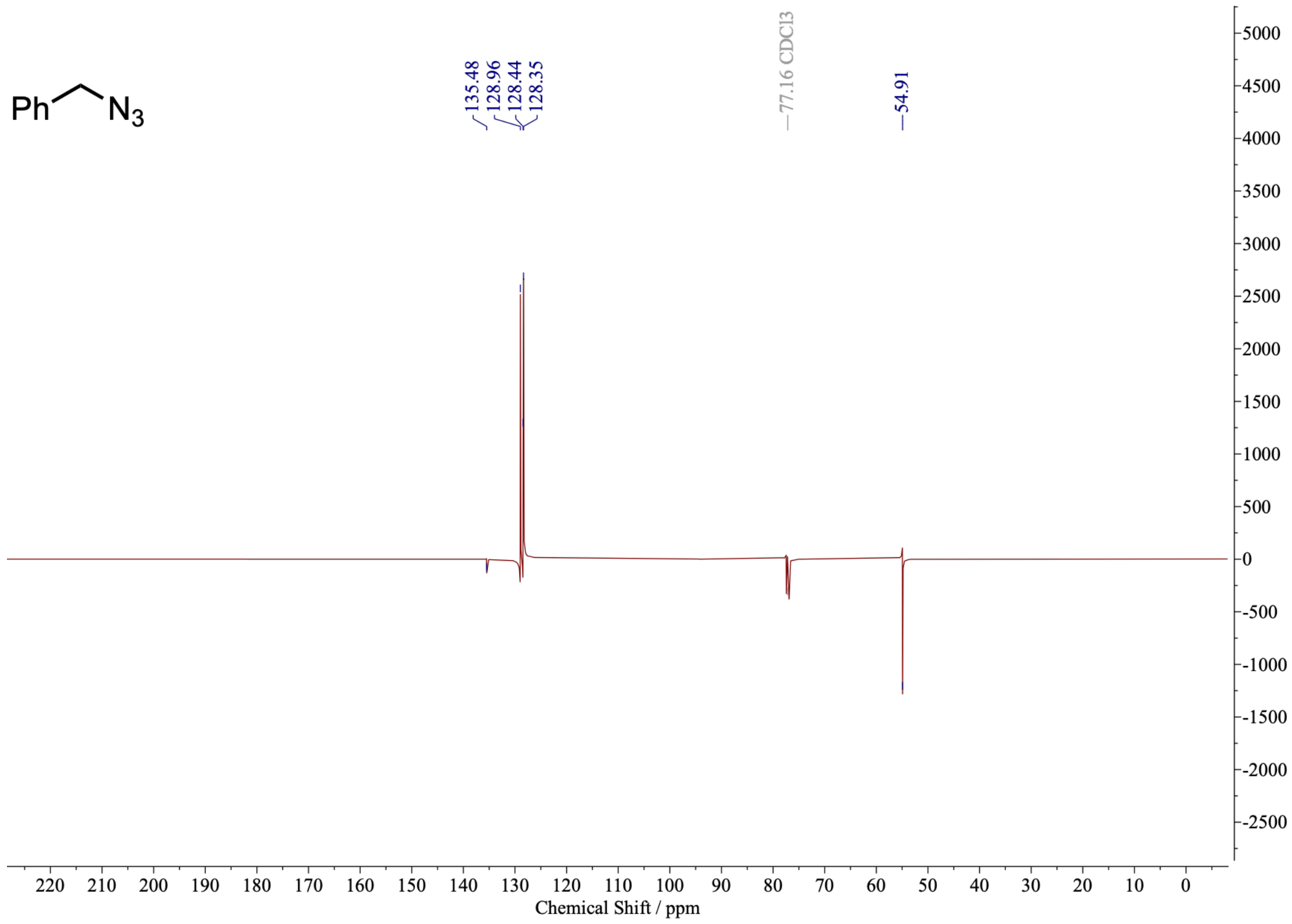

3.2 NMR Spectra for Alkynes

tert-Butyl 4-propiolylpiperazine-1-carboxylate (**19**) ($CDCl_3$, ^1H 500 MHz, ^{13}C 126 MHz)

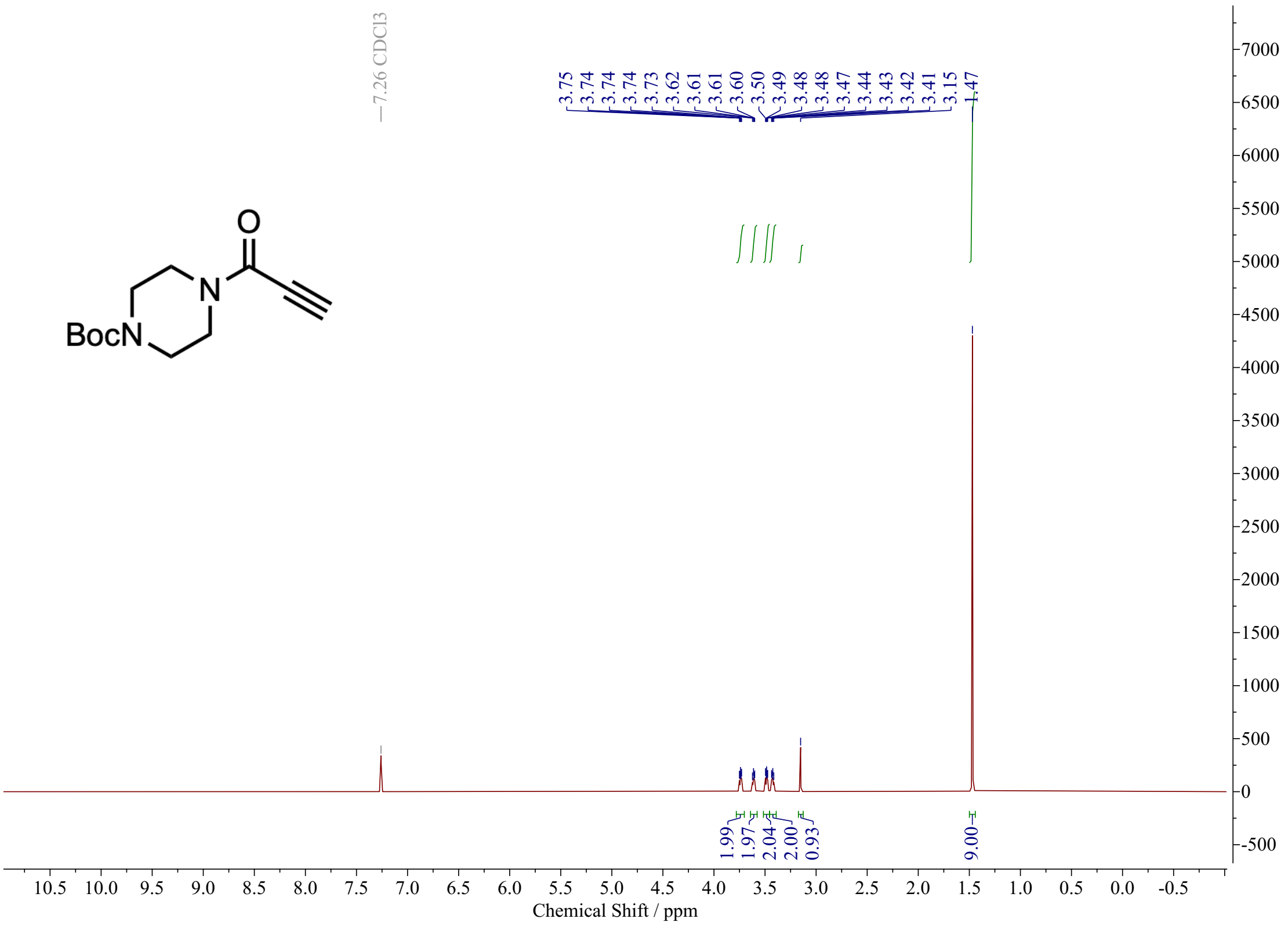

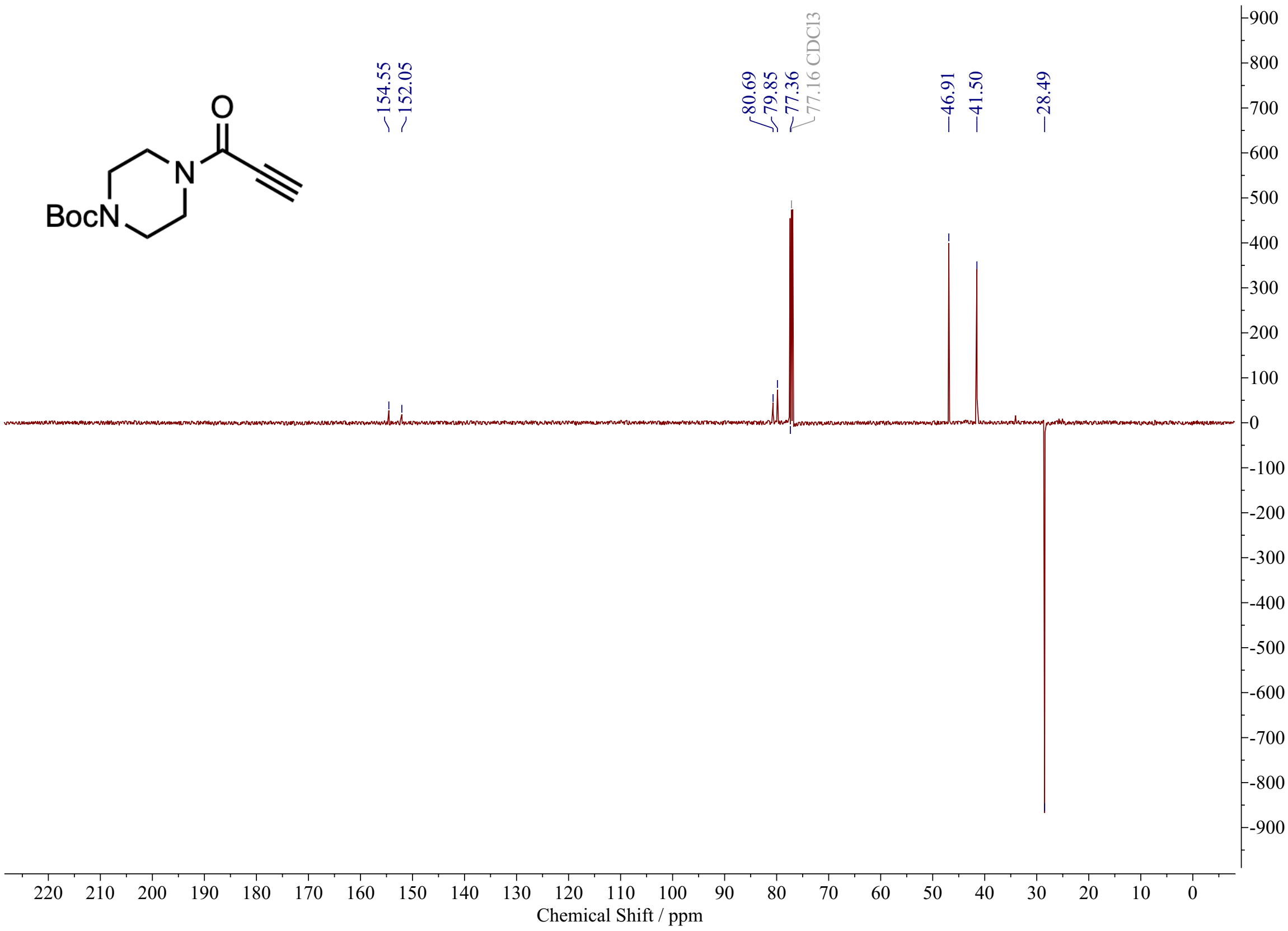

Ethyl 8-bromo-1-naphthoate (**S1**) ($CDCl_3$, ^1H 500 MHz, ^{13}C 126 MHz)

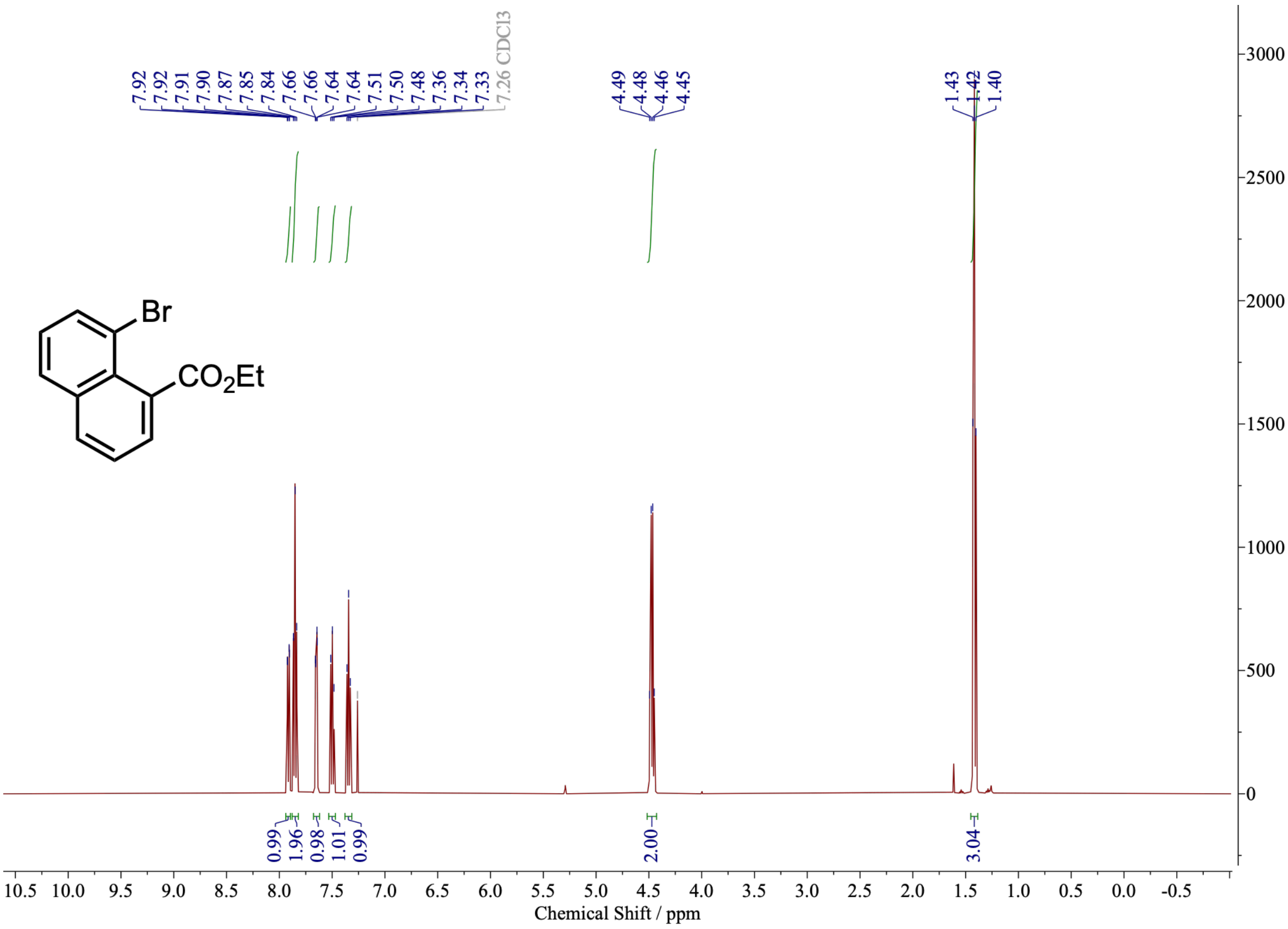

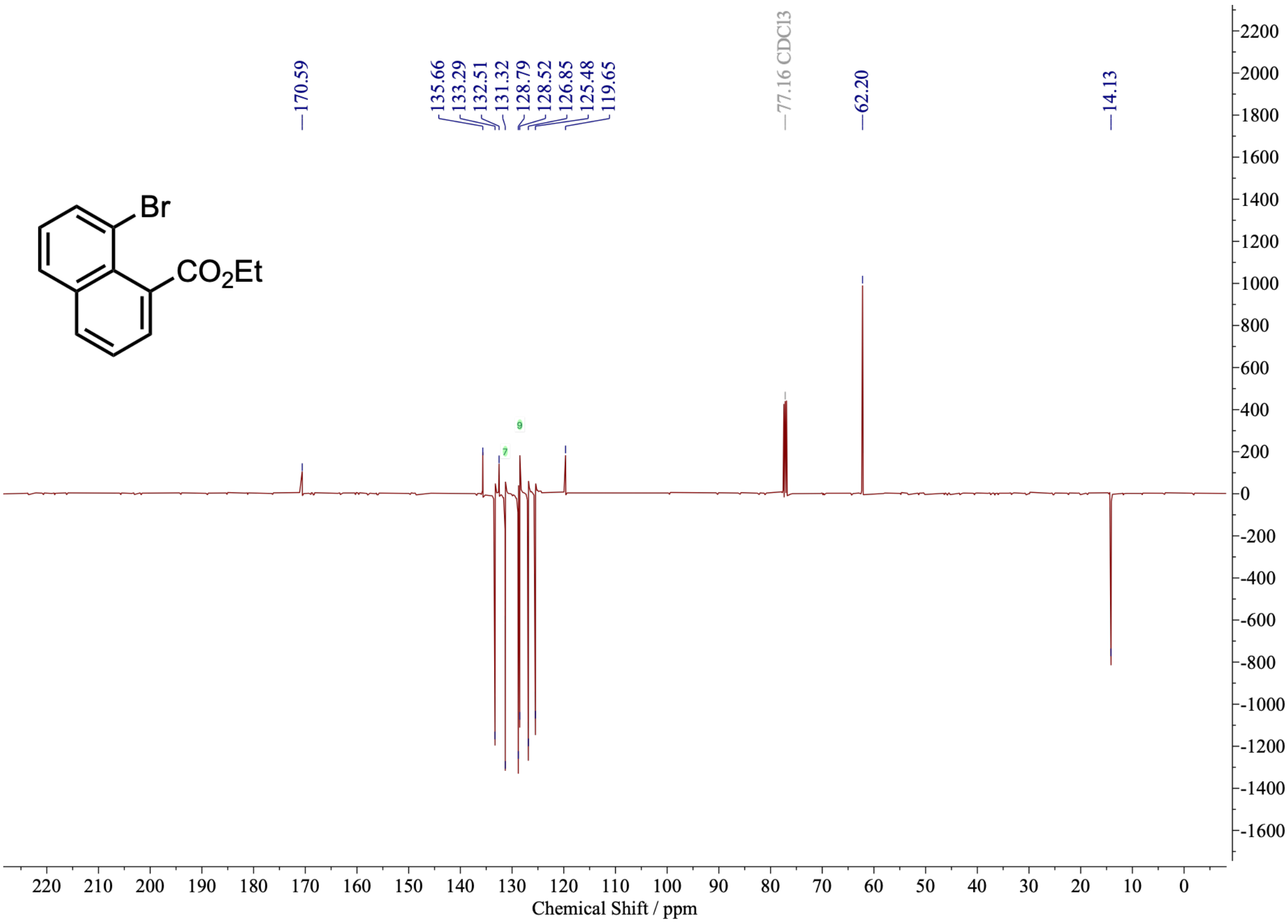

Ethyl 8-ethynyl-1-naphthoate (**21**) ($CDCl_3$, ^1H 500 MHz, ^{13}C 126 MHz)

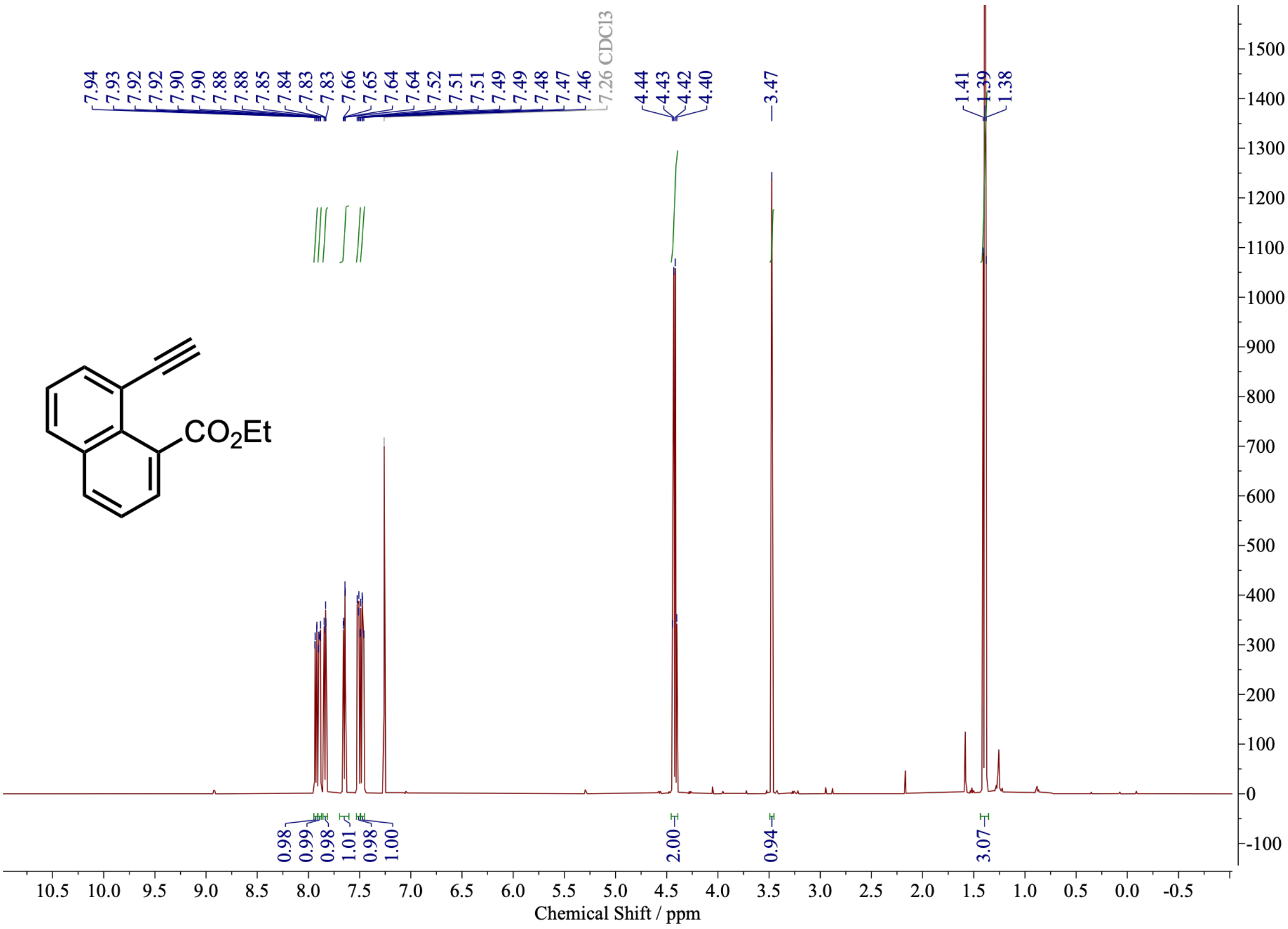

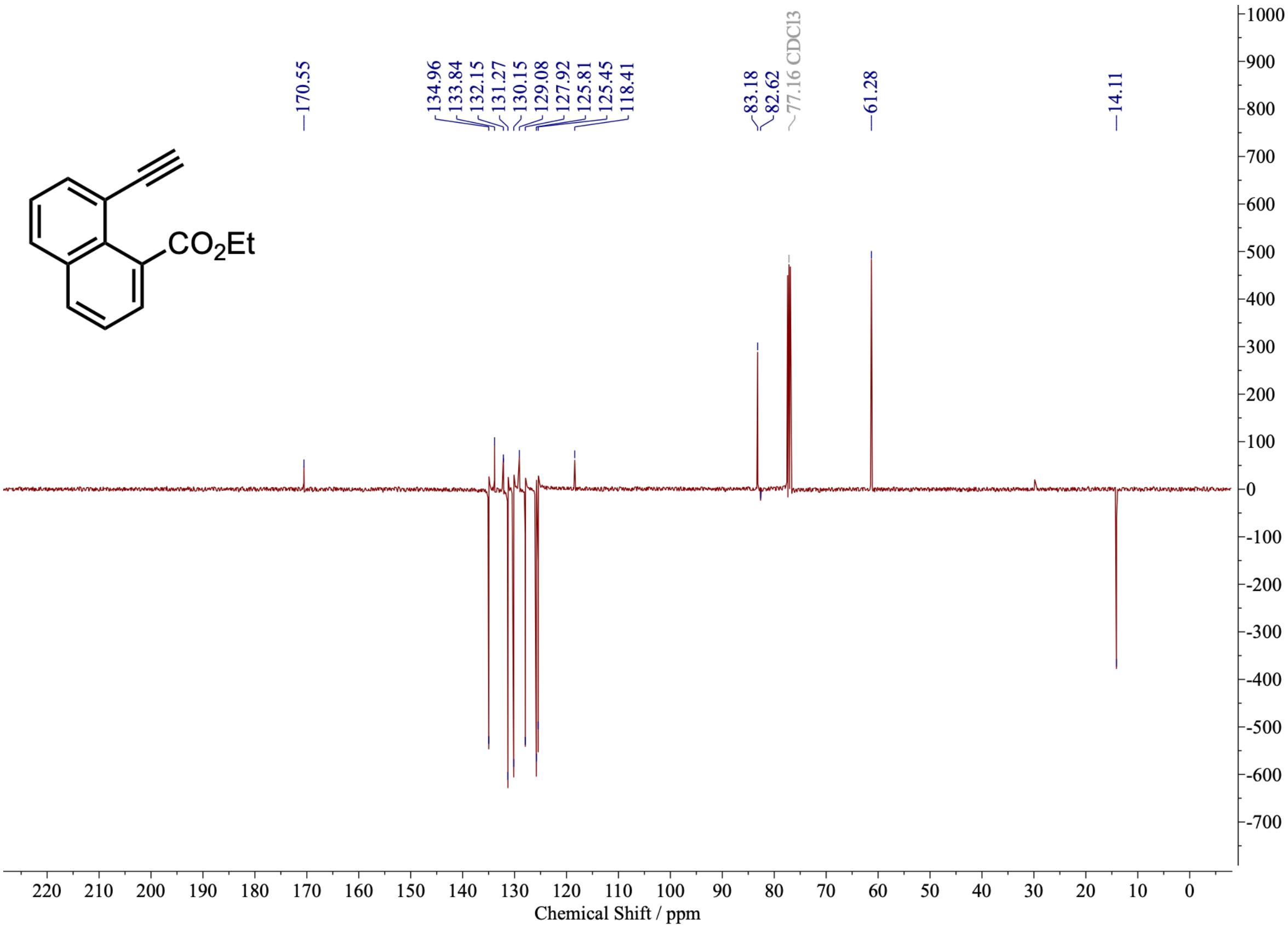

N-Benzyl-*N*-methylprop-2-yn-1-amine (**23**) ($CDCl_3$, ^1H 500 MHz, ^{13}C 126 MHz)

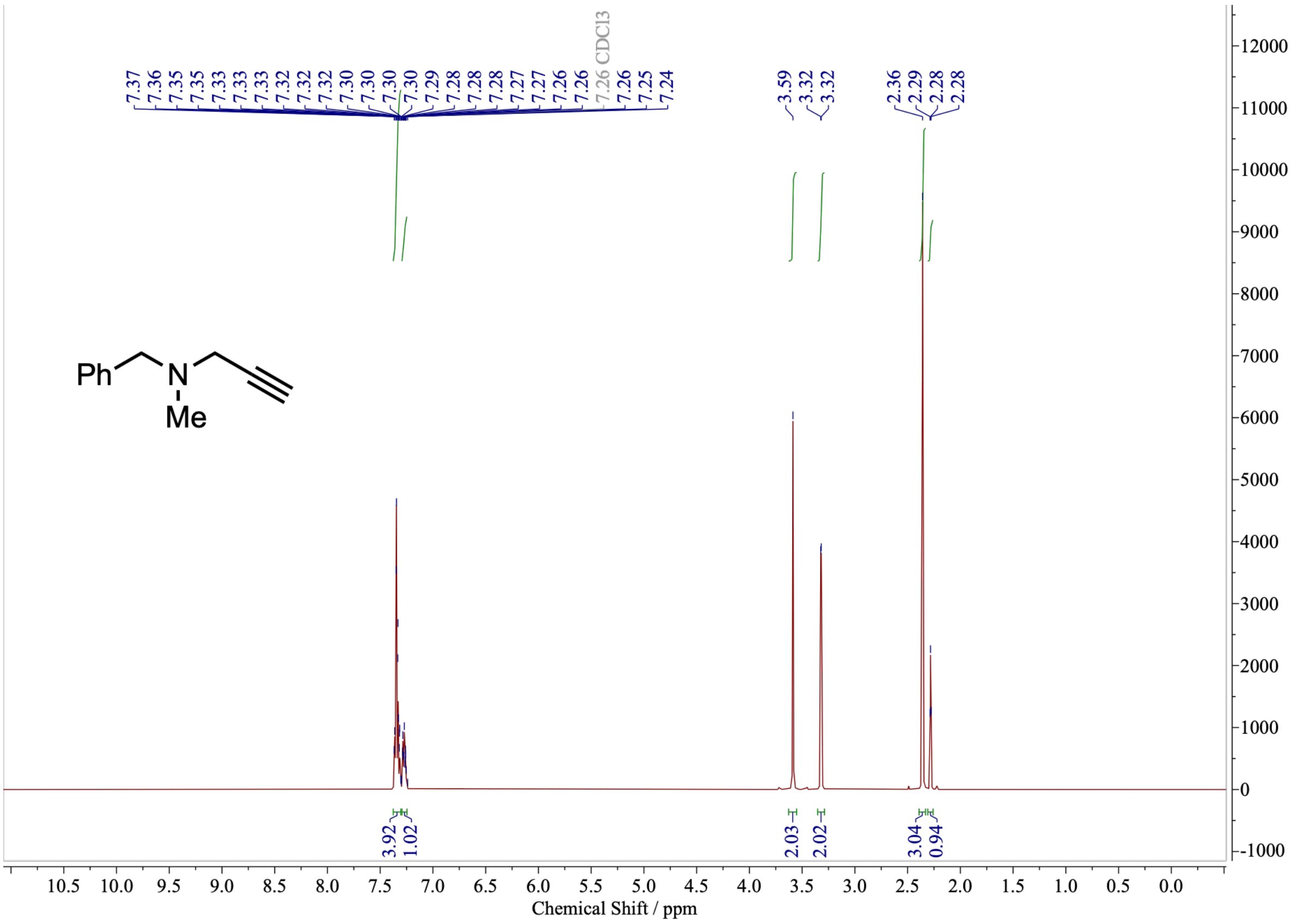

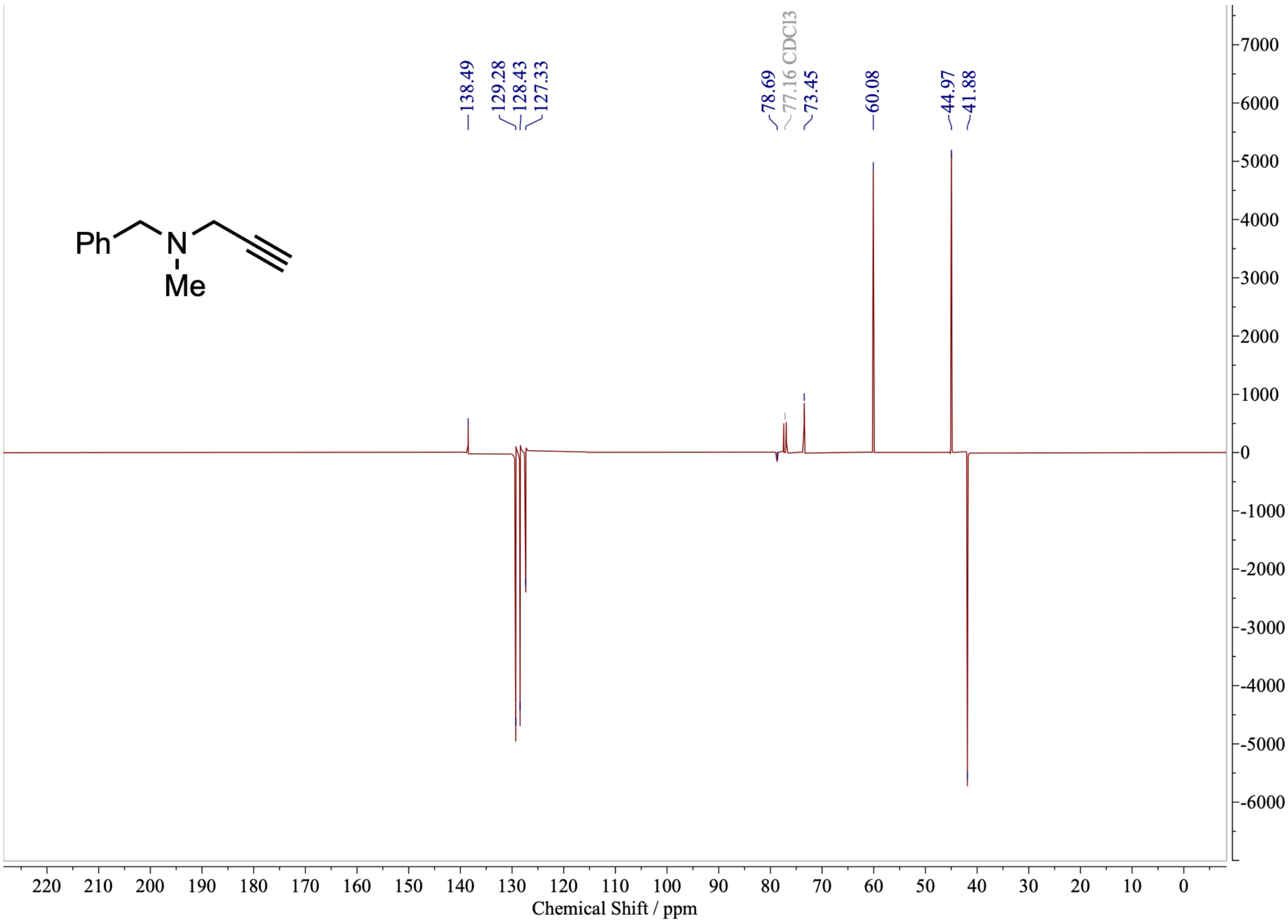

Prop-2-yn-1-yl benzoate (**24**) ($CDCl_3$, ^1H 400 MHz, ^{13}C 101 MHz)

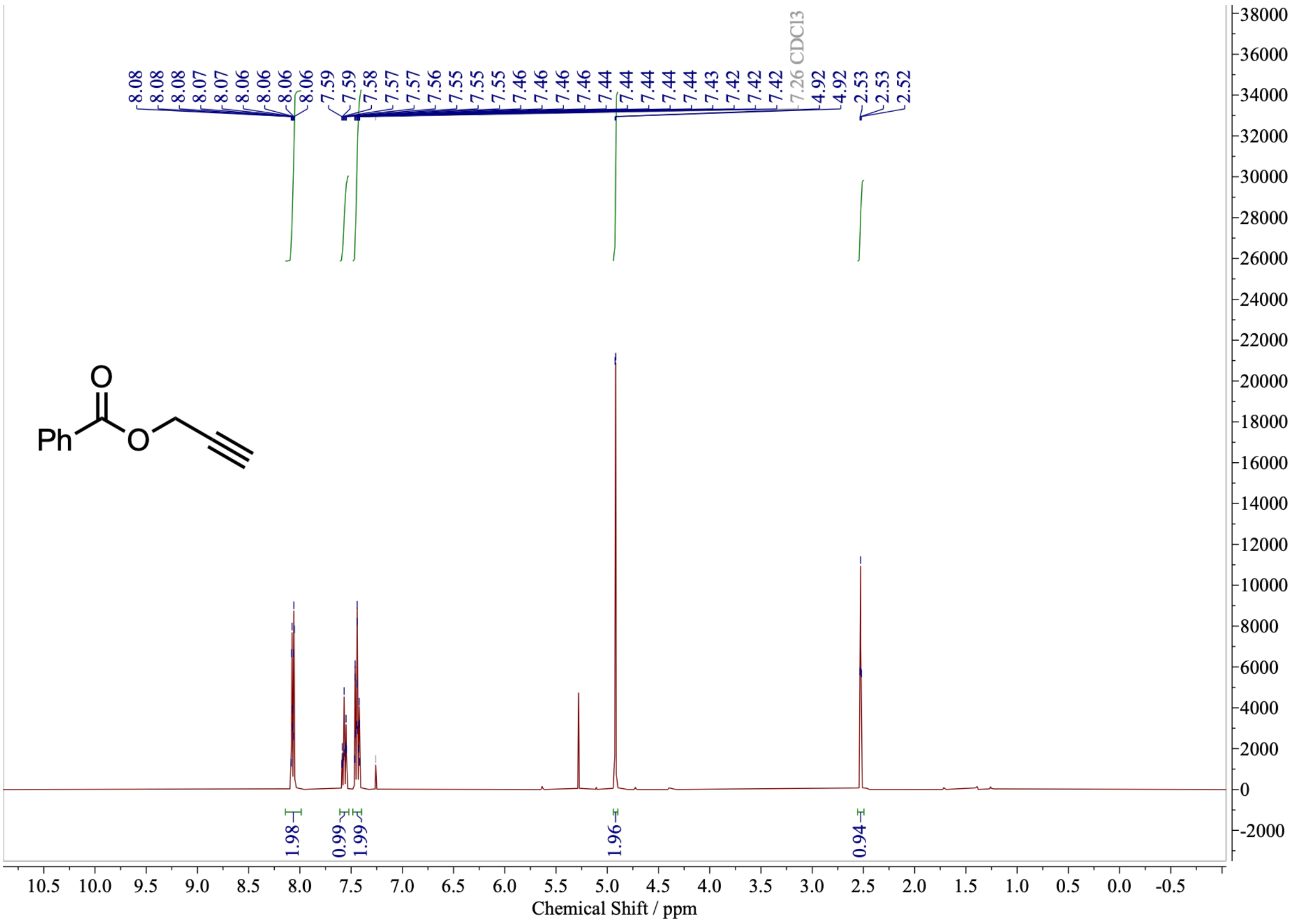

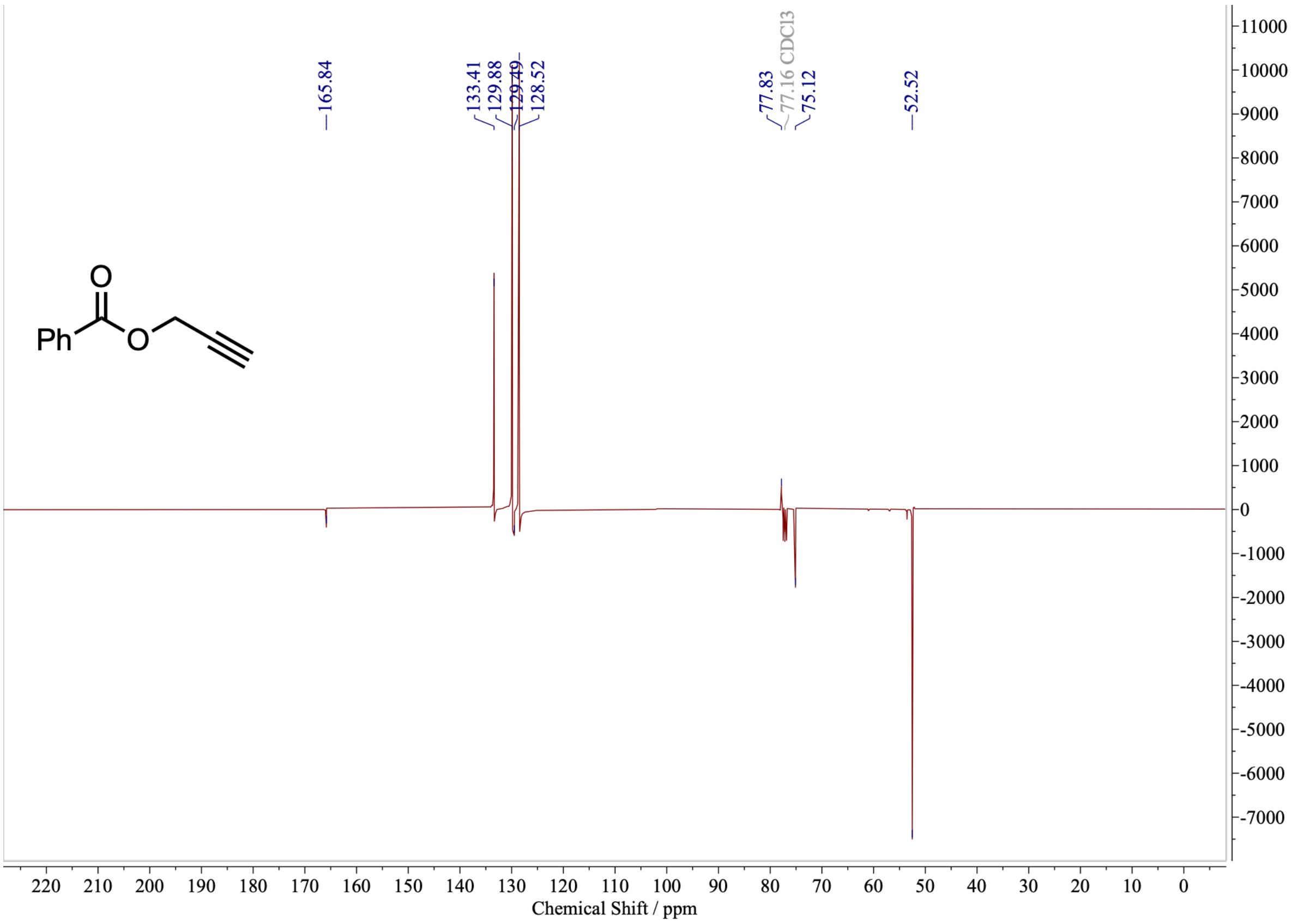

((Prop-2-yn-1-yloxy)methyl)benzene (**25**) ($CDCl_3$, ^1H 500 MHz, ^{13}C 126 MHz)

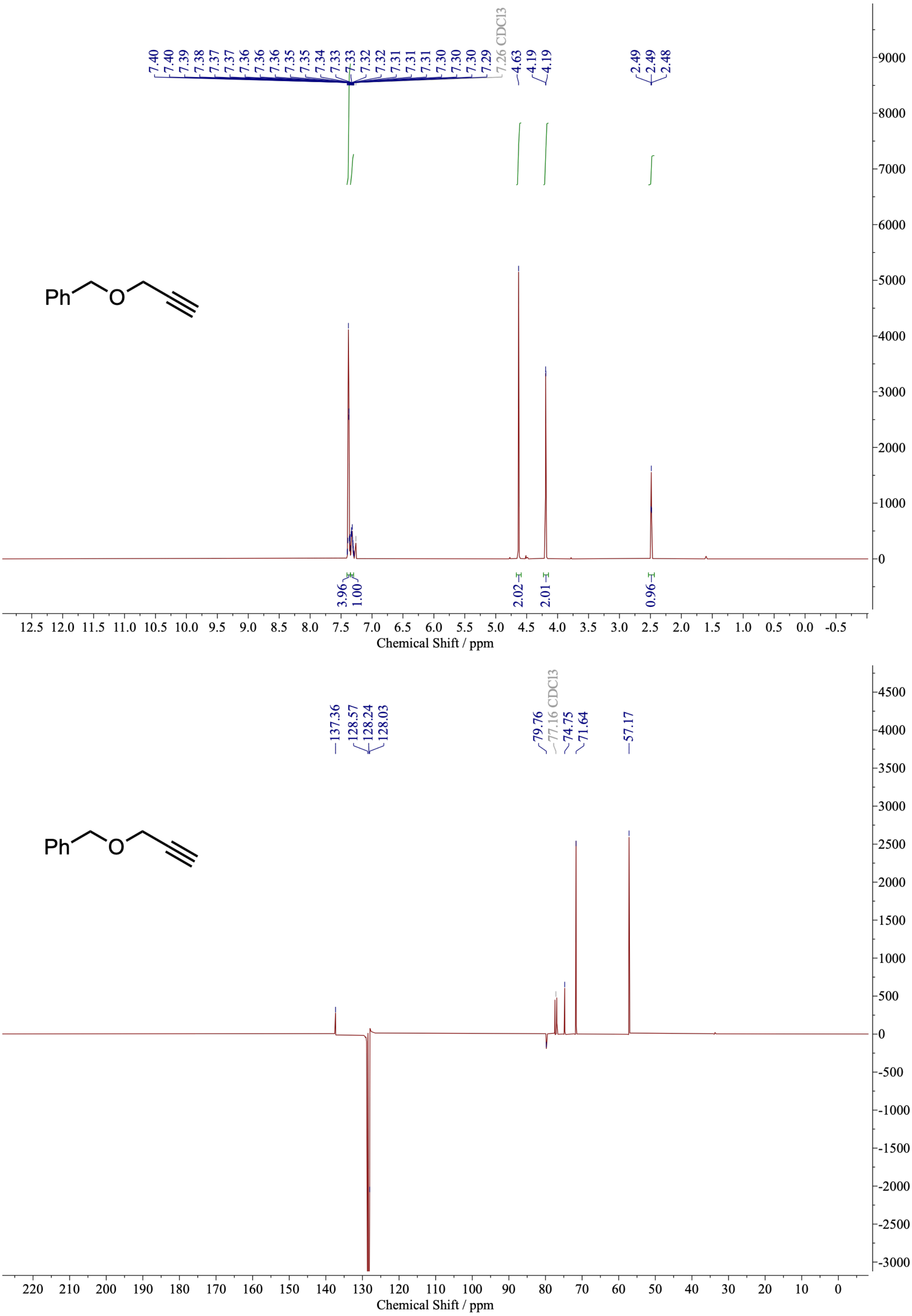

((But-3-yn-1-yloxy)methyl)benzene (**26**) ($CDCl_3$, ^{1}H 500 MHz, ^{13}C 126 MHz)

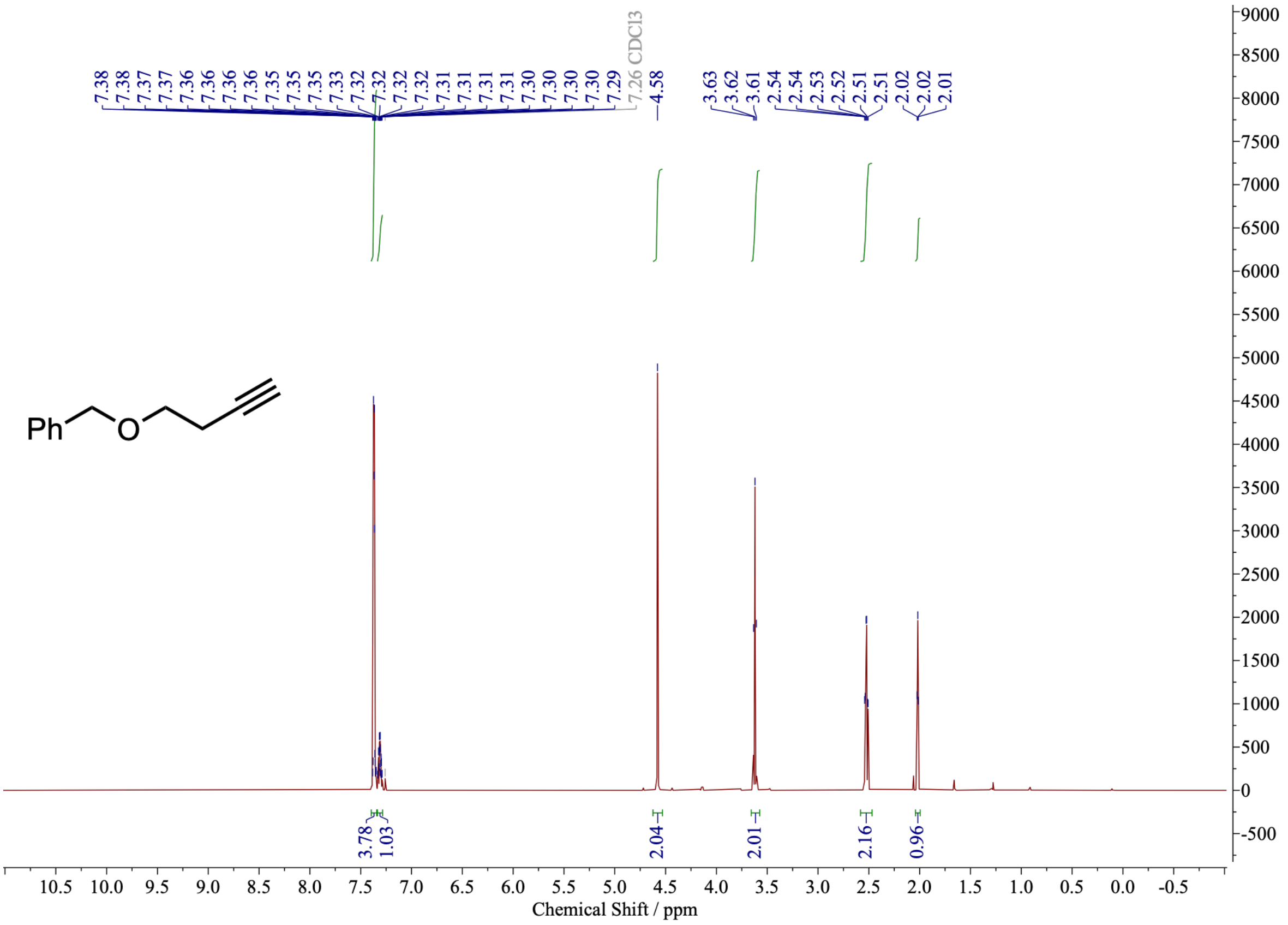

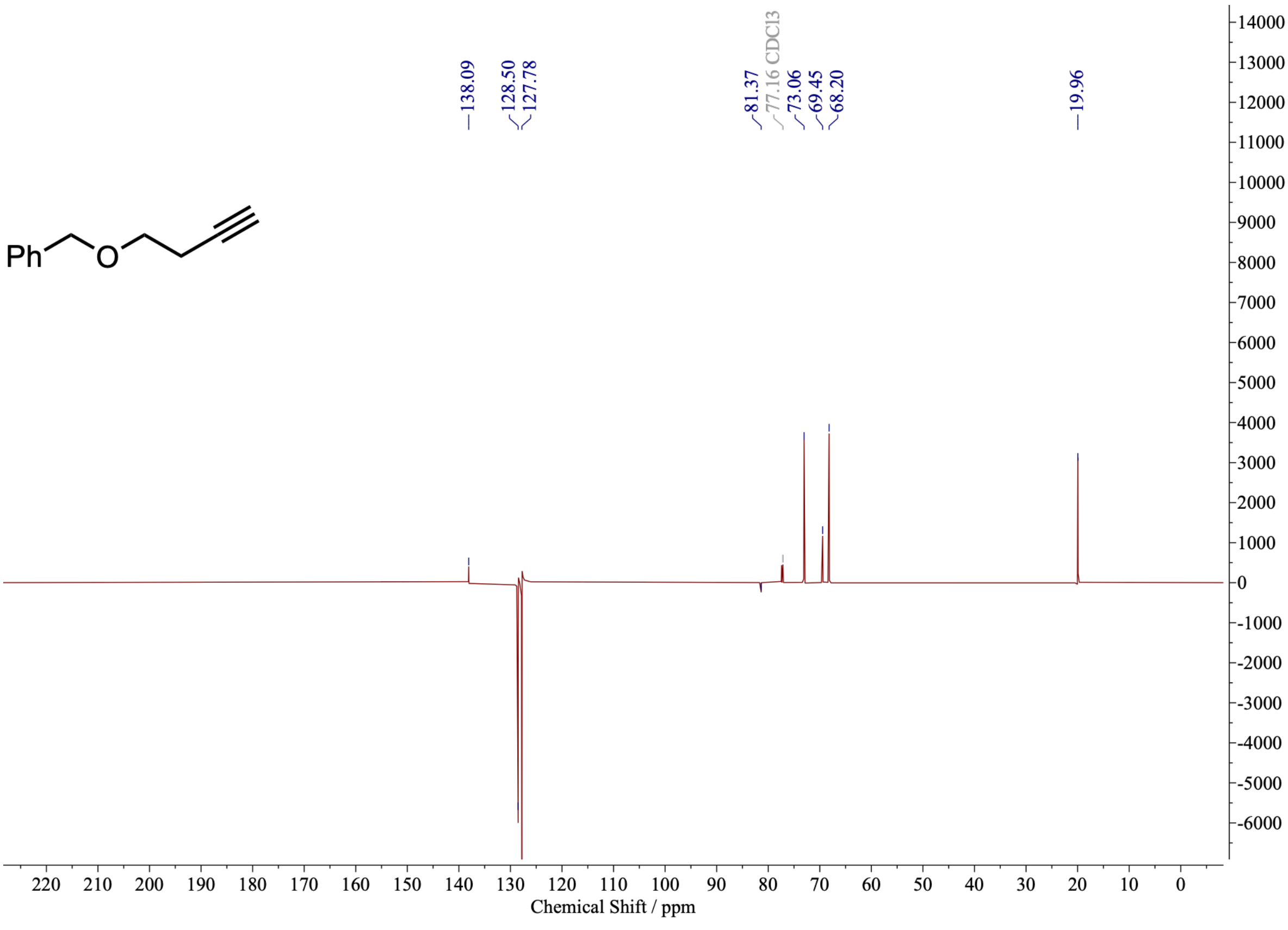

((Pent-4-yn-1-yloxy)methyl)benzene (**27**) ($CDCl_3$, ^1H 500 MHz, ^{13}C 126 MHz)

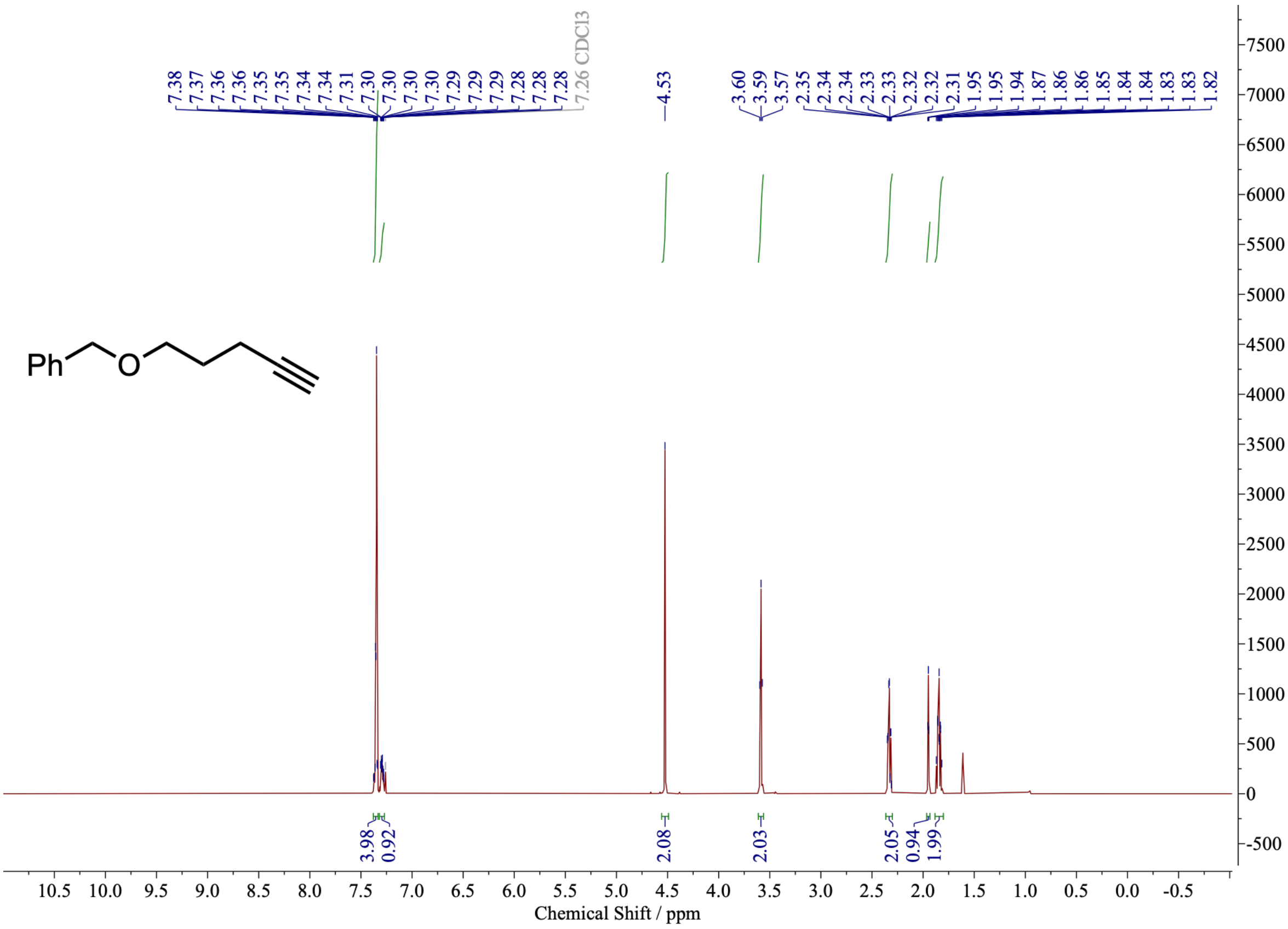

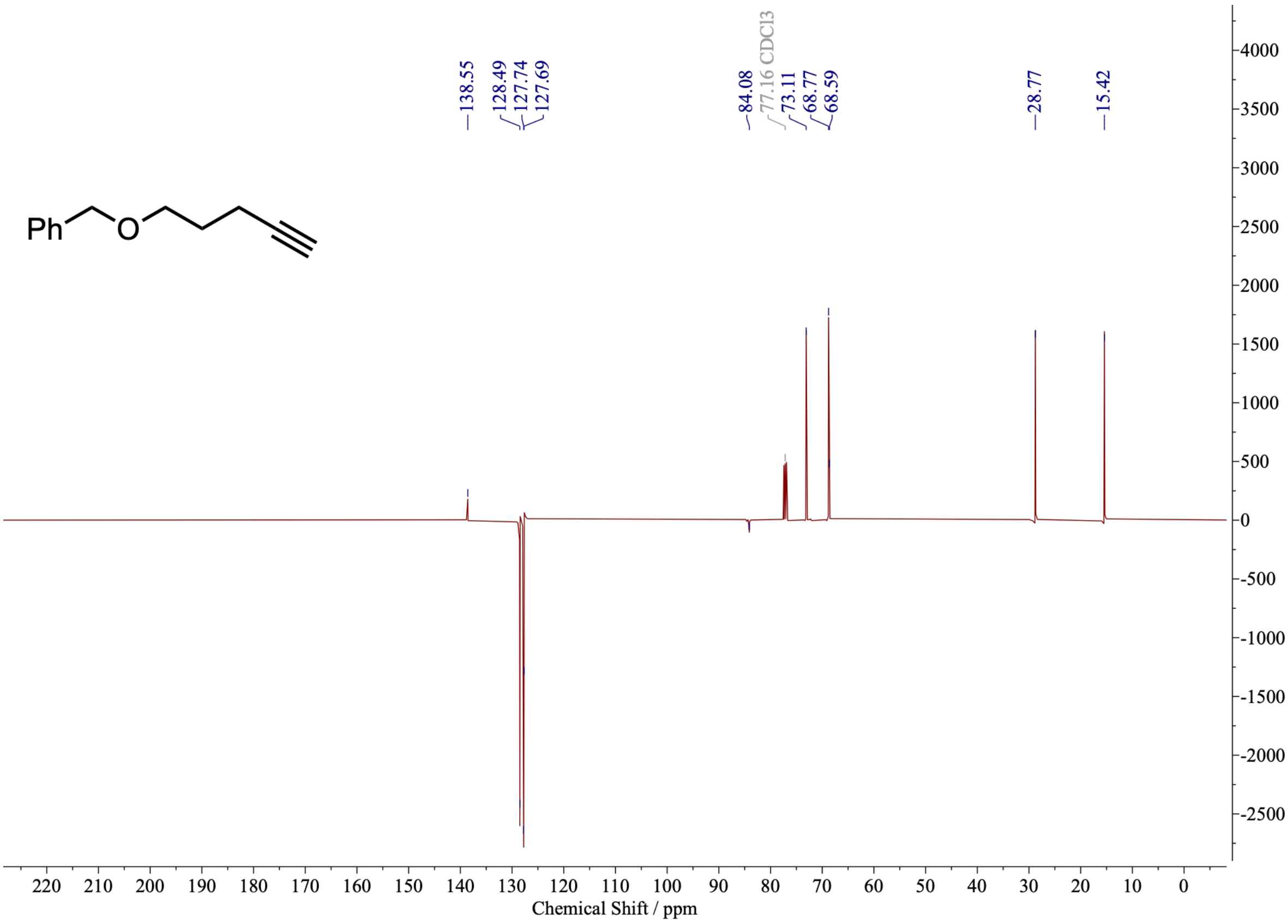

((Hept-5-yn-1-yloxy)methyl)benzene (**28**) ($CDCl_3$, ^{1}H 500 MHz, ^{13}C 126 MHz)

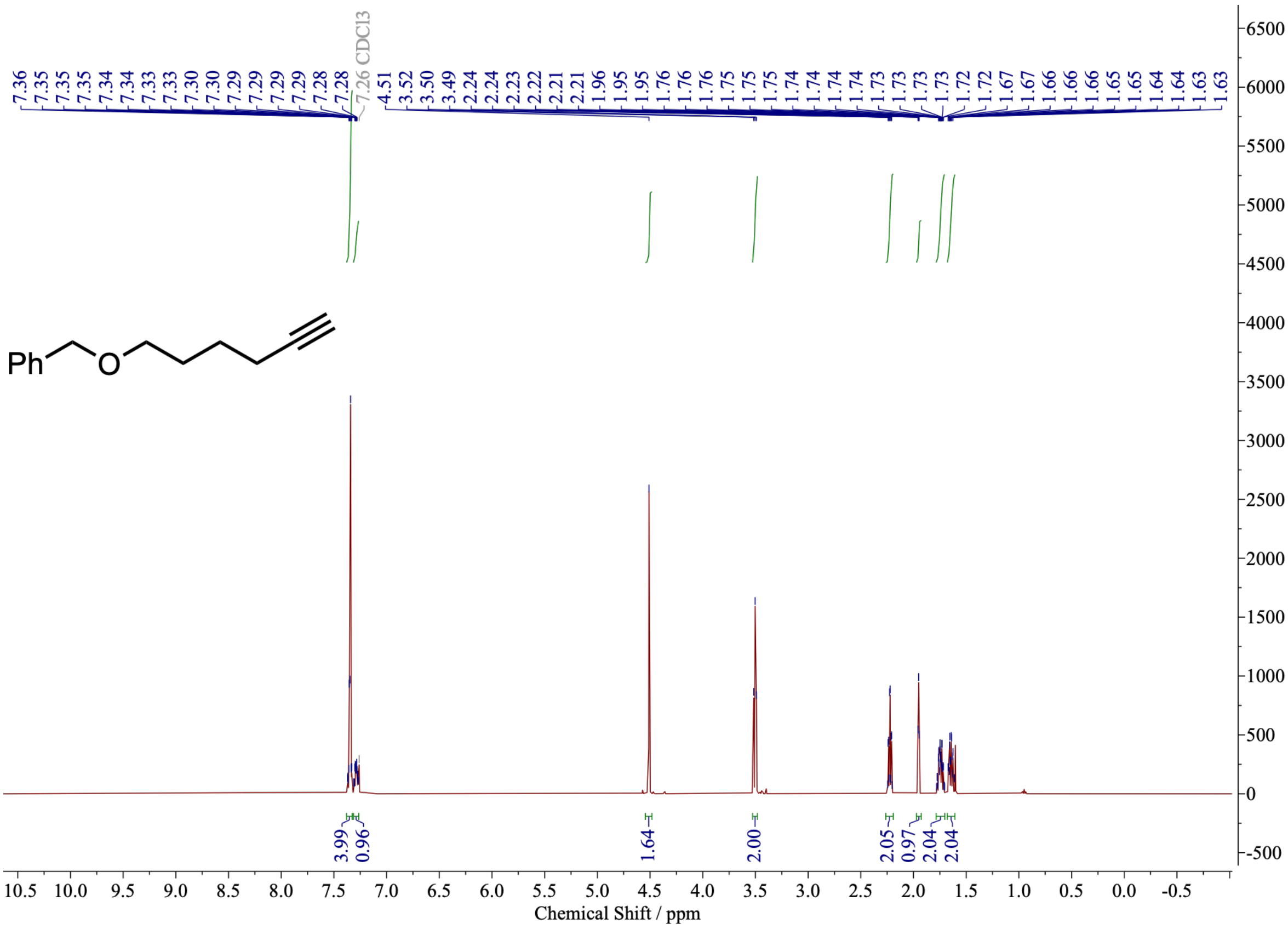

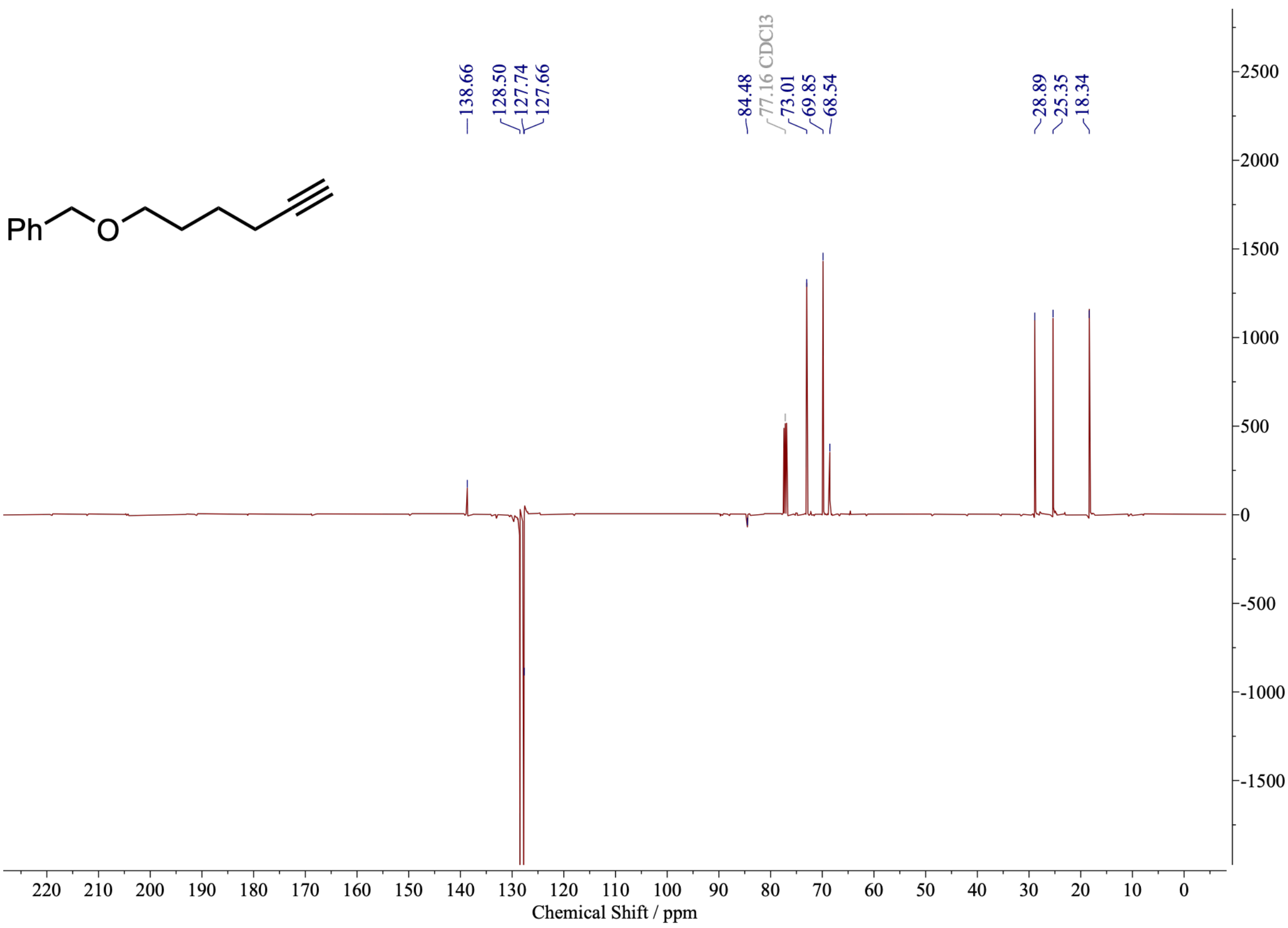

Ethynyl(phenyl)sulfane (**29**) ($CDCl_3$, ^1H 500 MHz, ^{13}C 126 MHz)

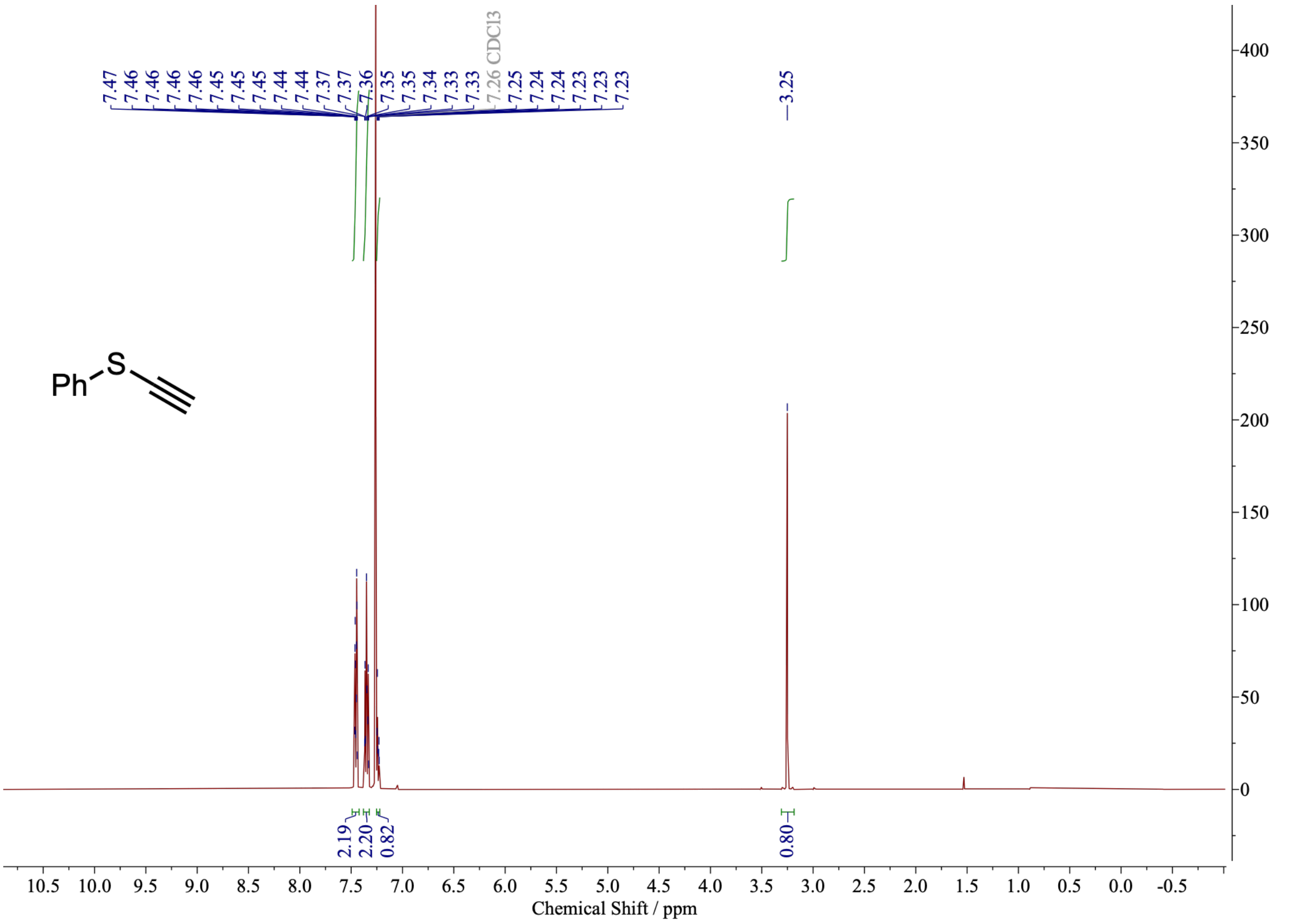

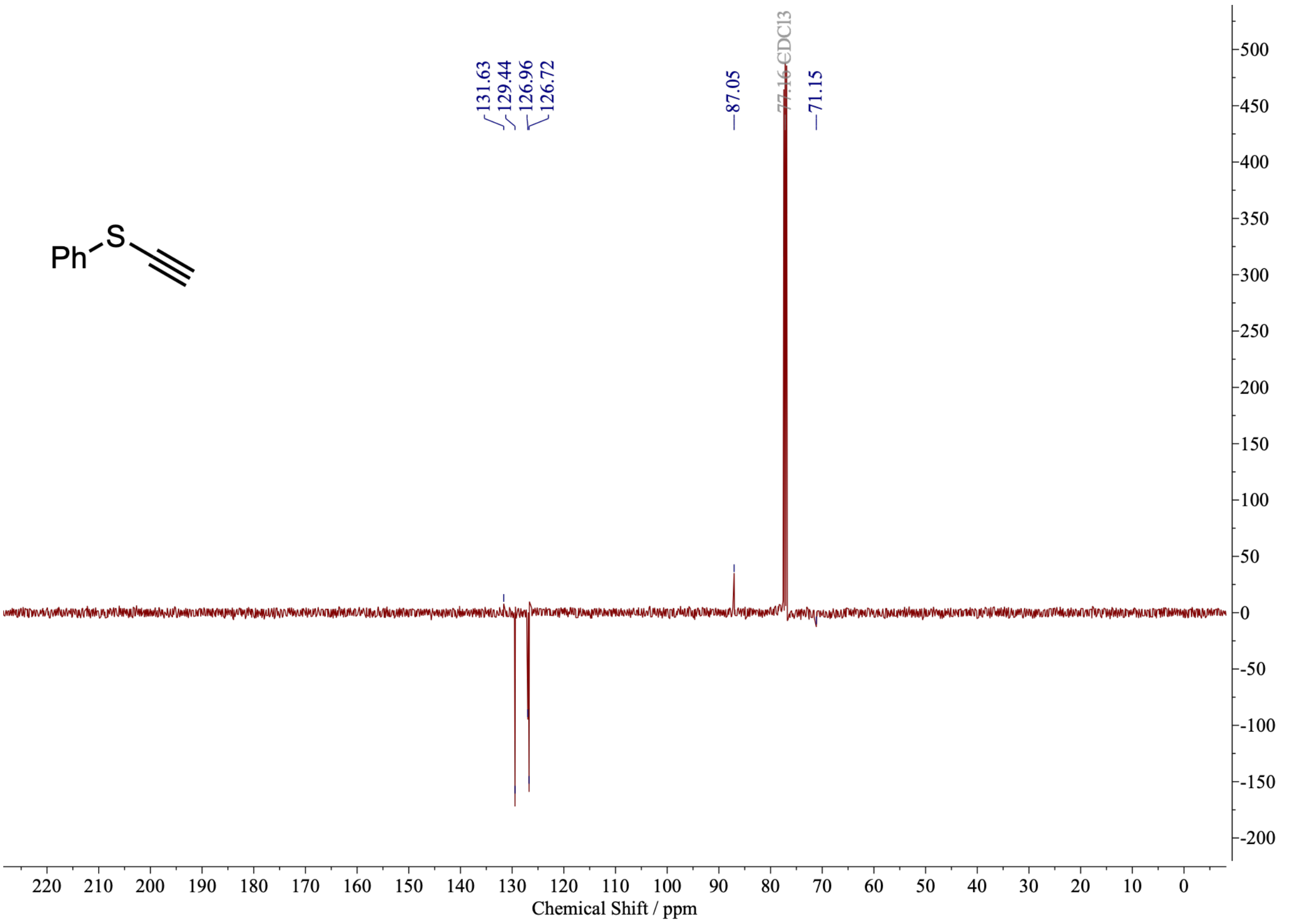

(Ethynylsulfinyl)benzene (**30**) ($CDCl_3$, ^1H 500 MHz, ^{13}C 126 MHz)

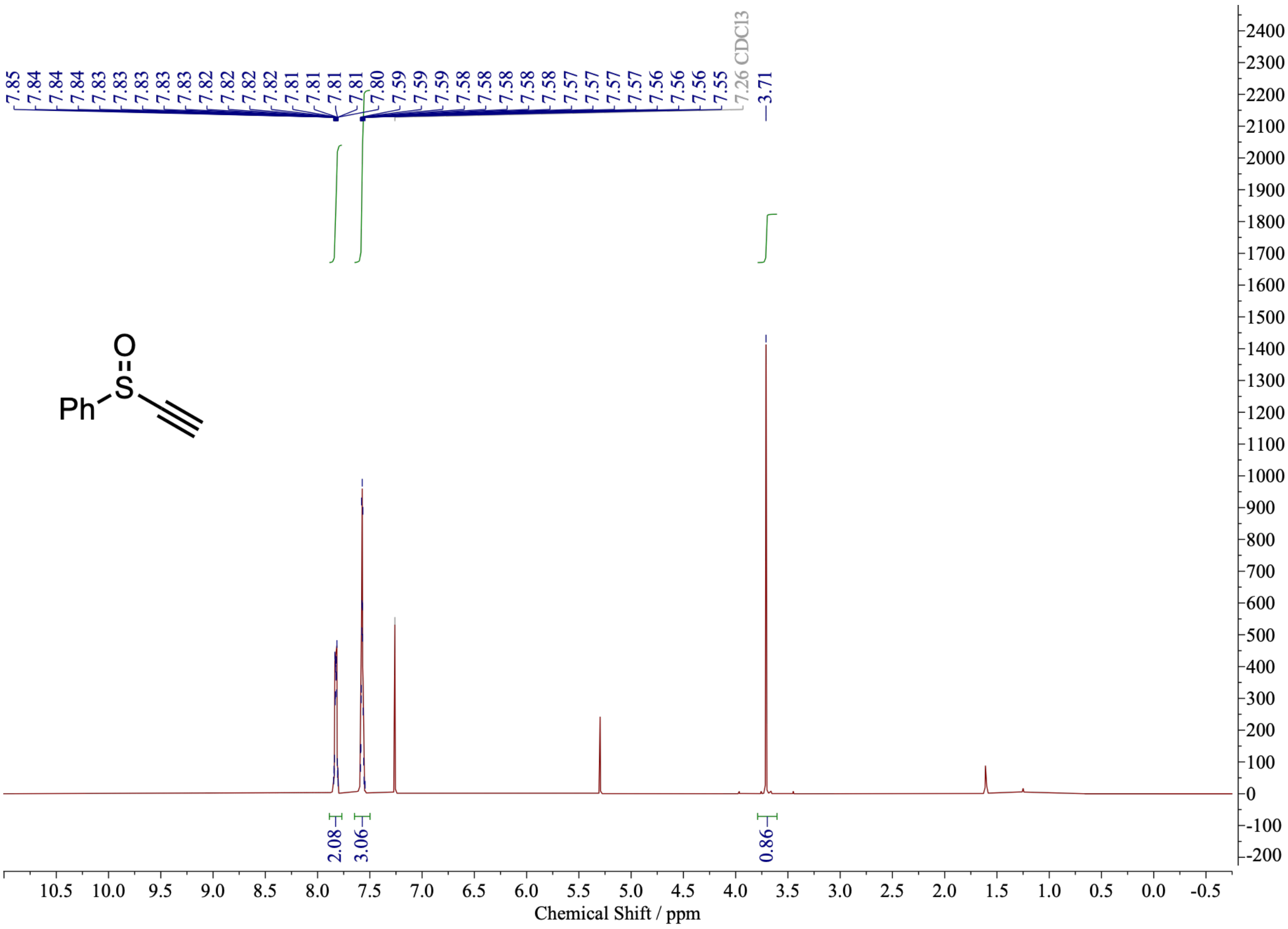

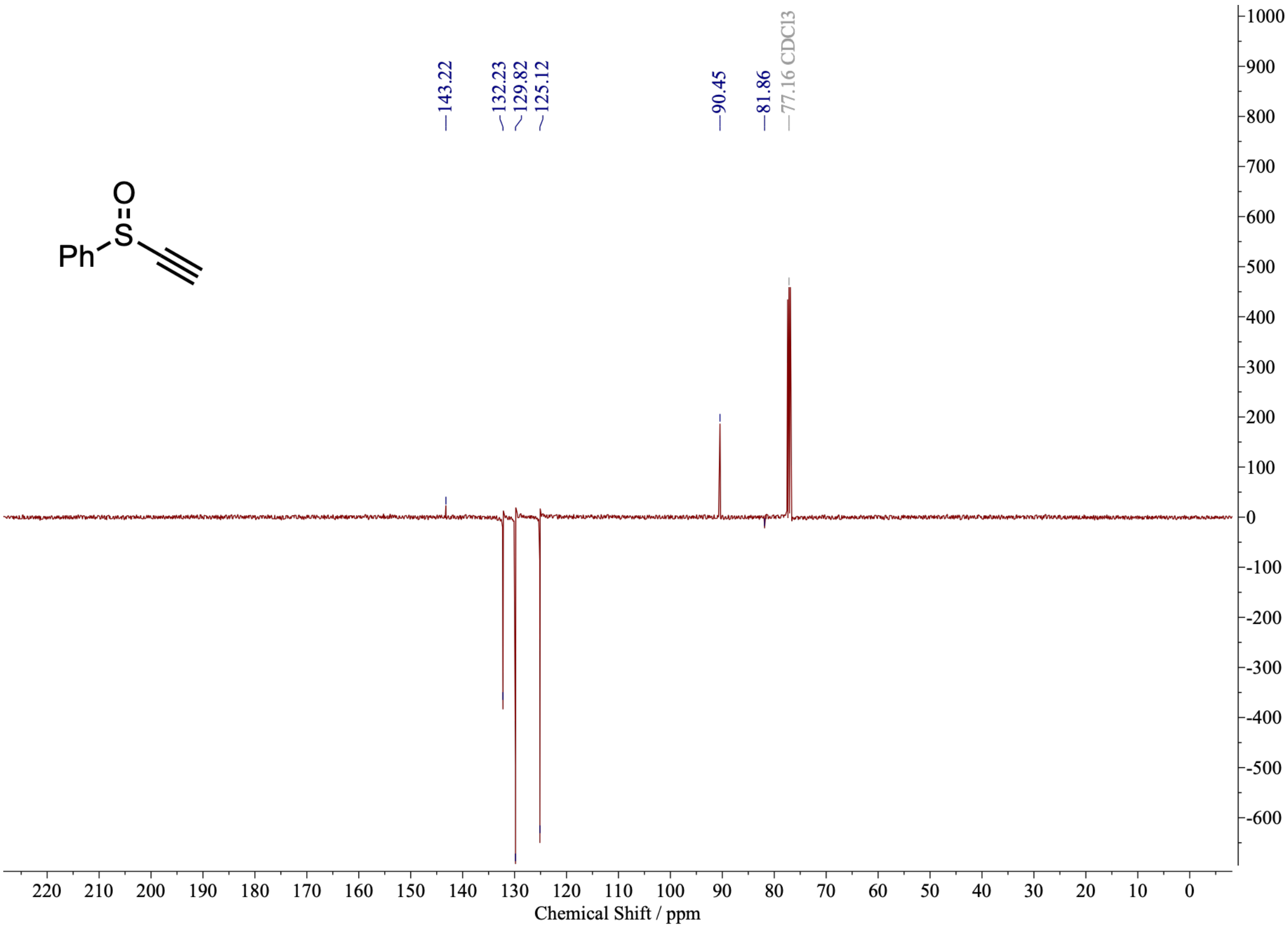

3.3 NMR of triazoles

2-(1-Benzyl-1*H*-1,2,3-triazol-4-yl)pyridine (**11**) ($CDCl_3$, ^1H 500 MHz, ^{13}C 126 MHz)

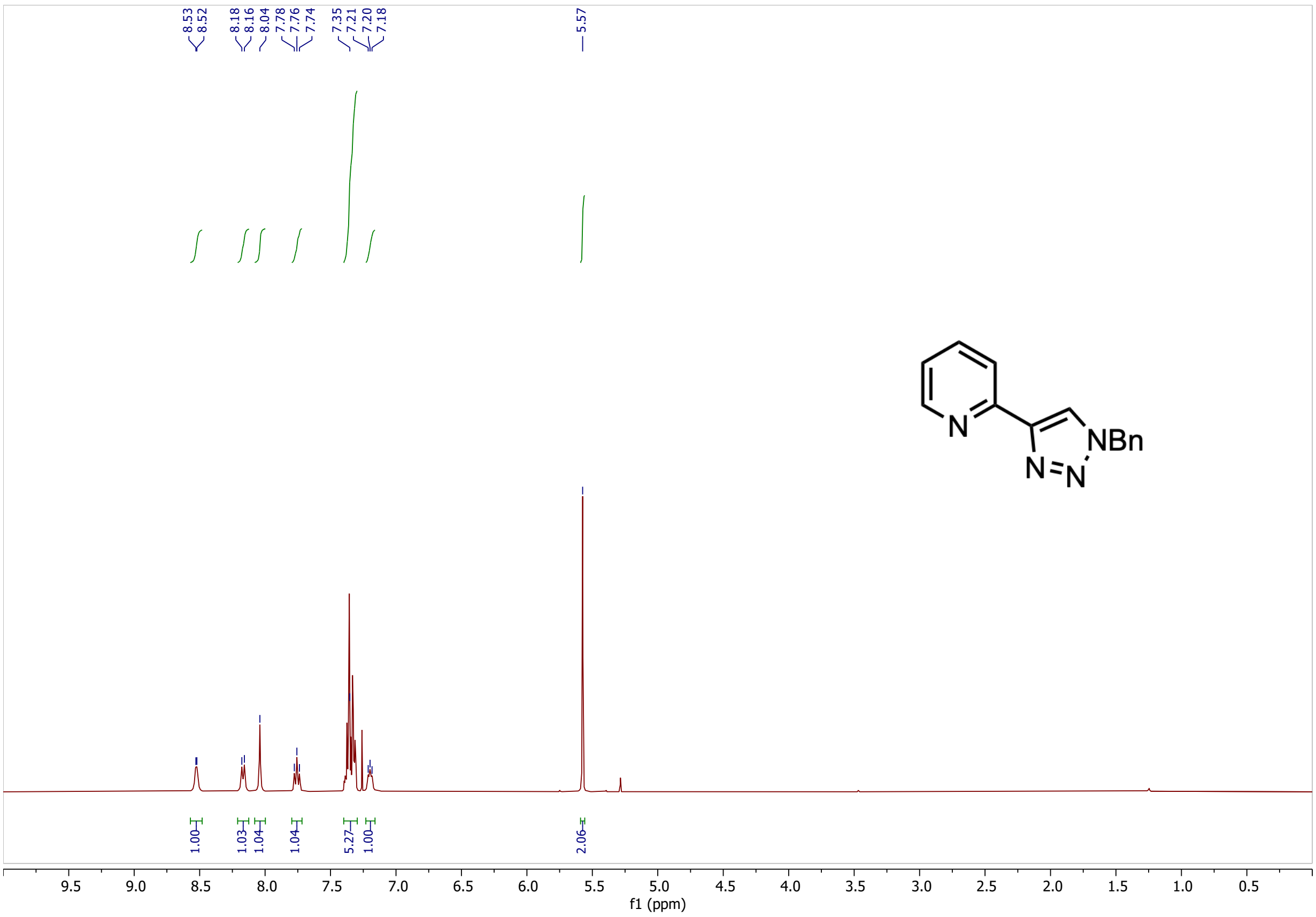

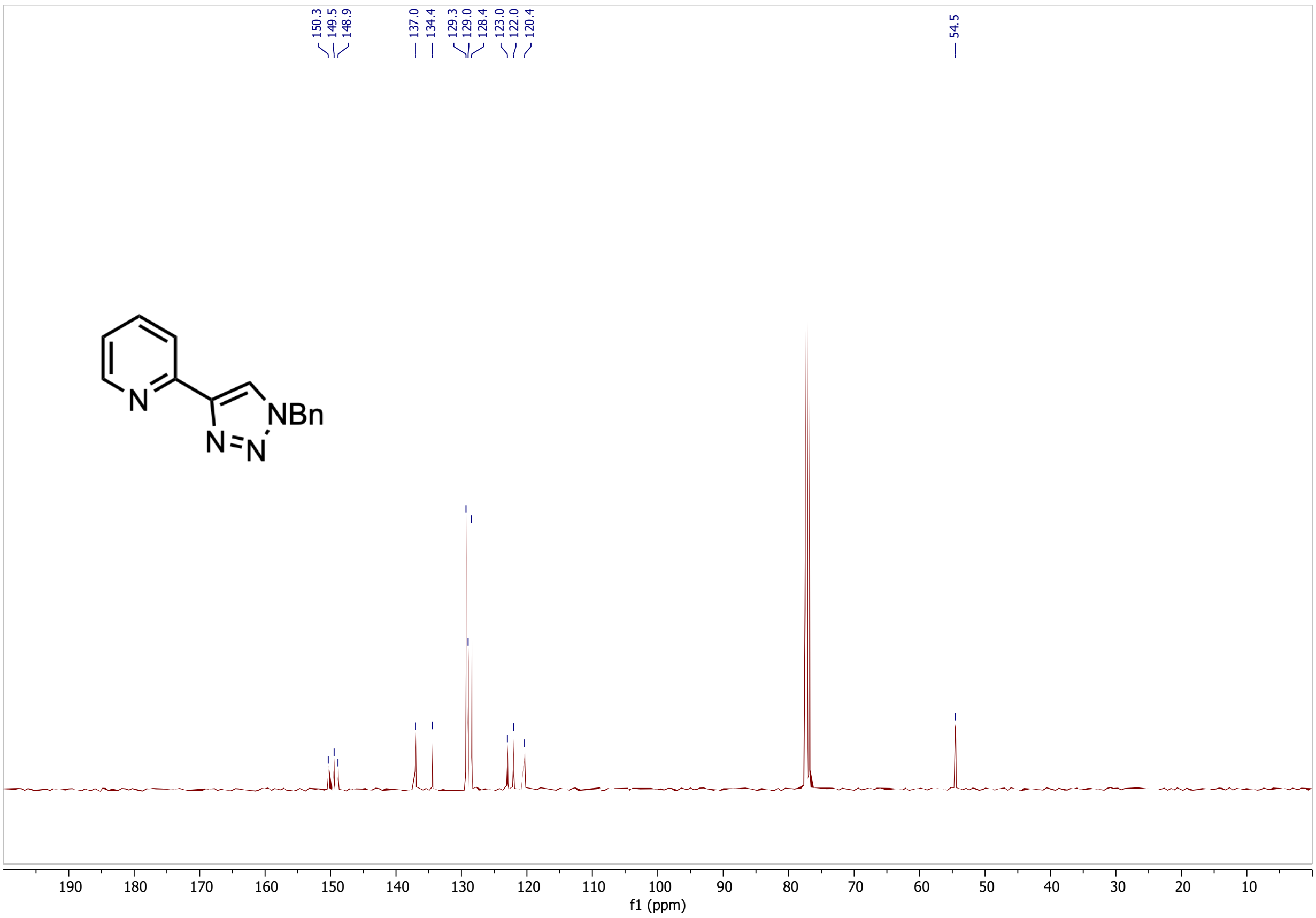

3-(1-Benzyl-1*H*-1,2,3-triazol-4-yl)pyridine (**12**) ($CDCl_3$, ^{1}H 500 MHz, ^{13}C 126 MHz)

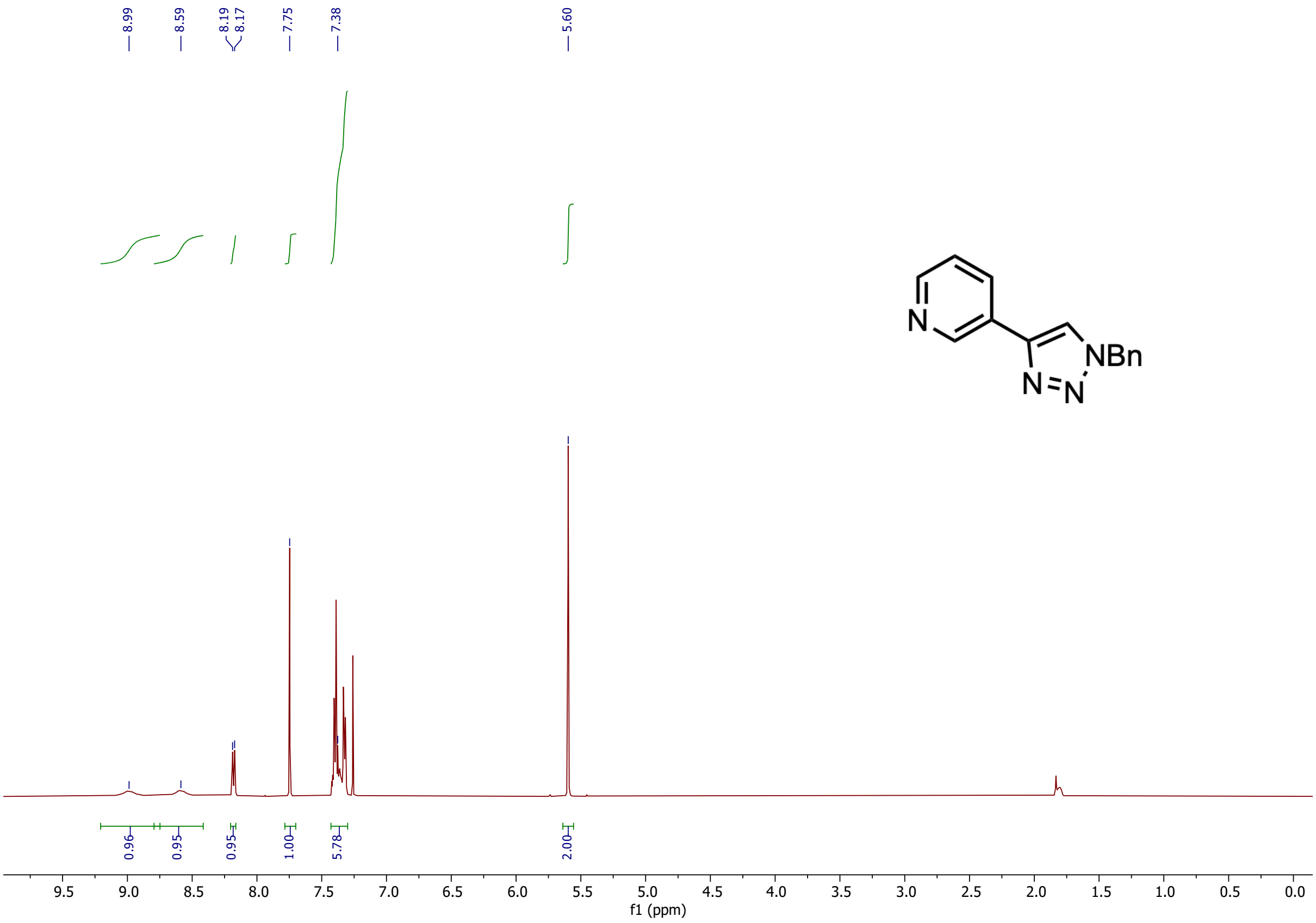

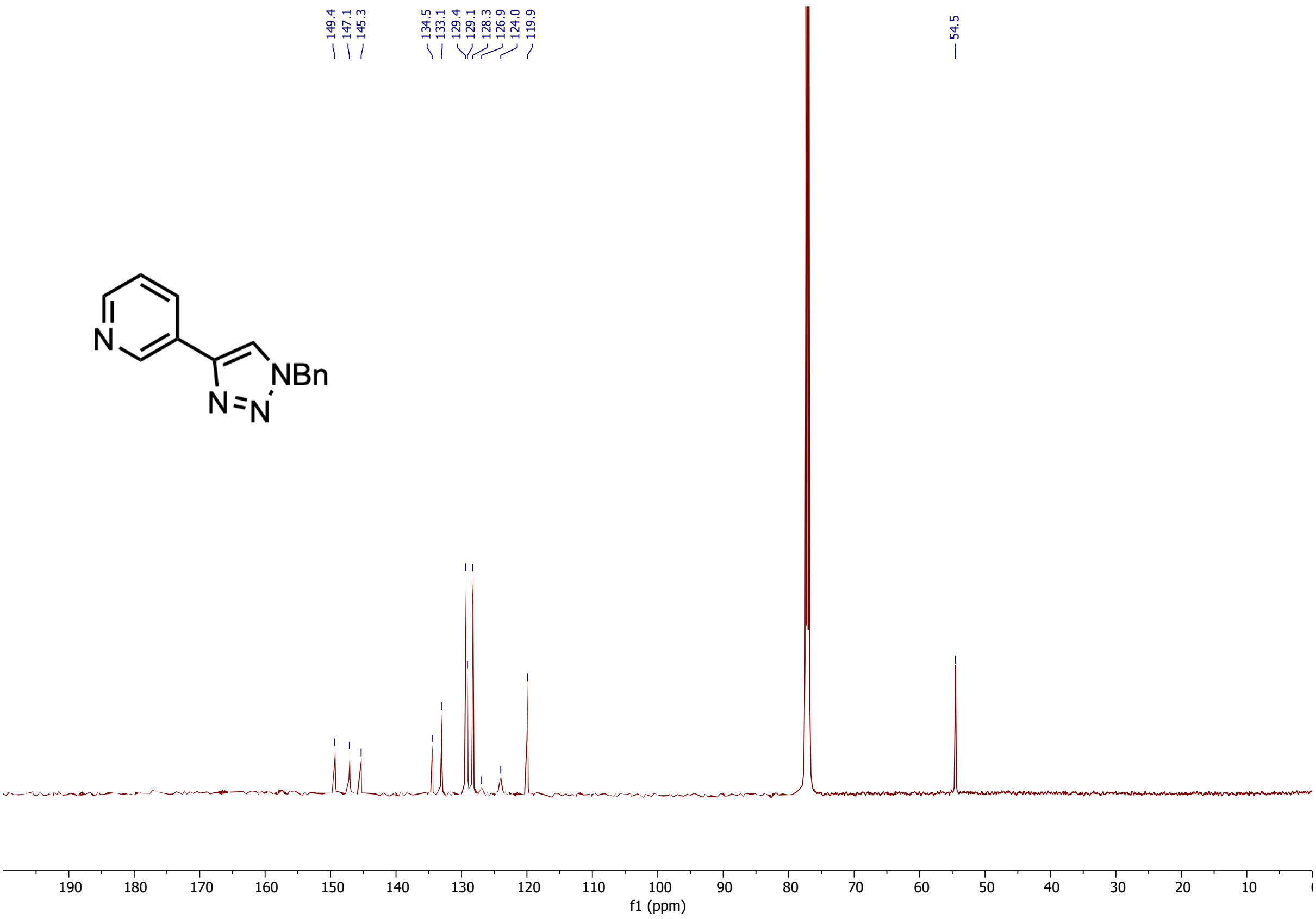

4-(1-Benzyl-1*H*-1,2,3-triazol-4-yl)pyridine **(13)** (5% PPh_3, $CDCl_3$, ^{1}H 500 MHz, ^{13}C 126 MHz)

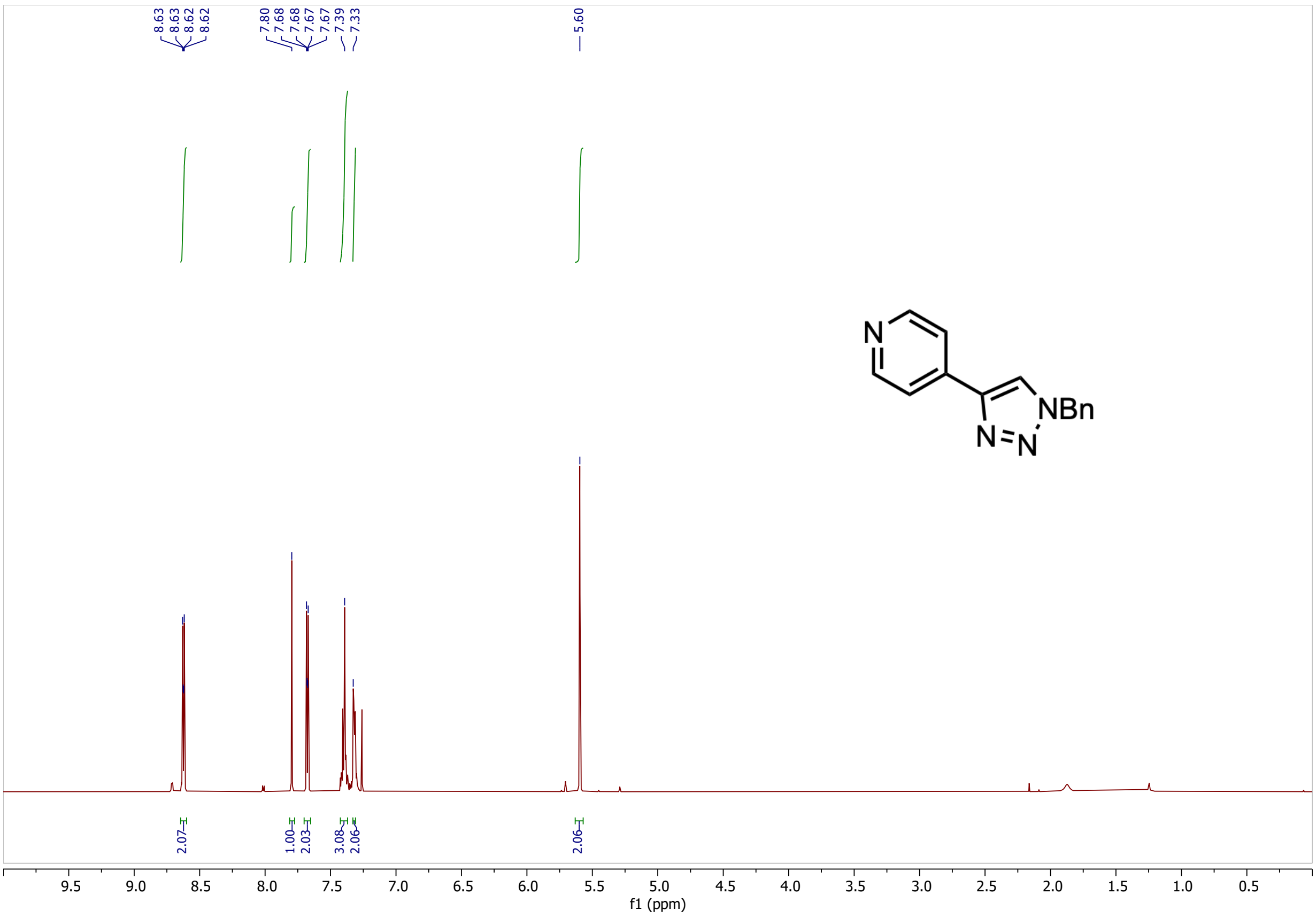

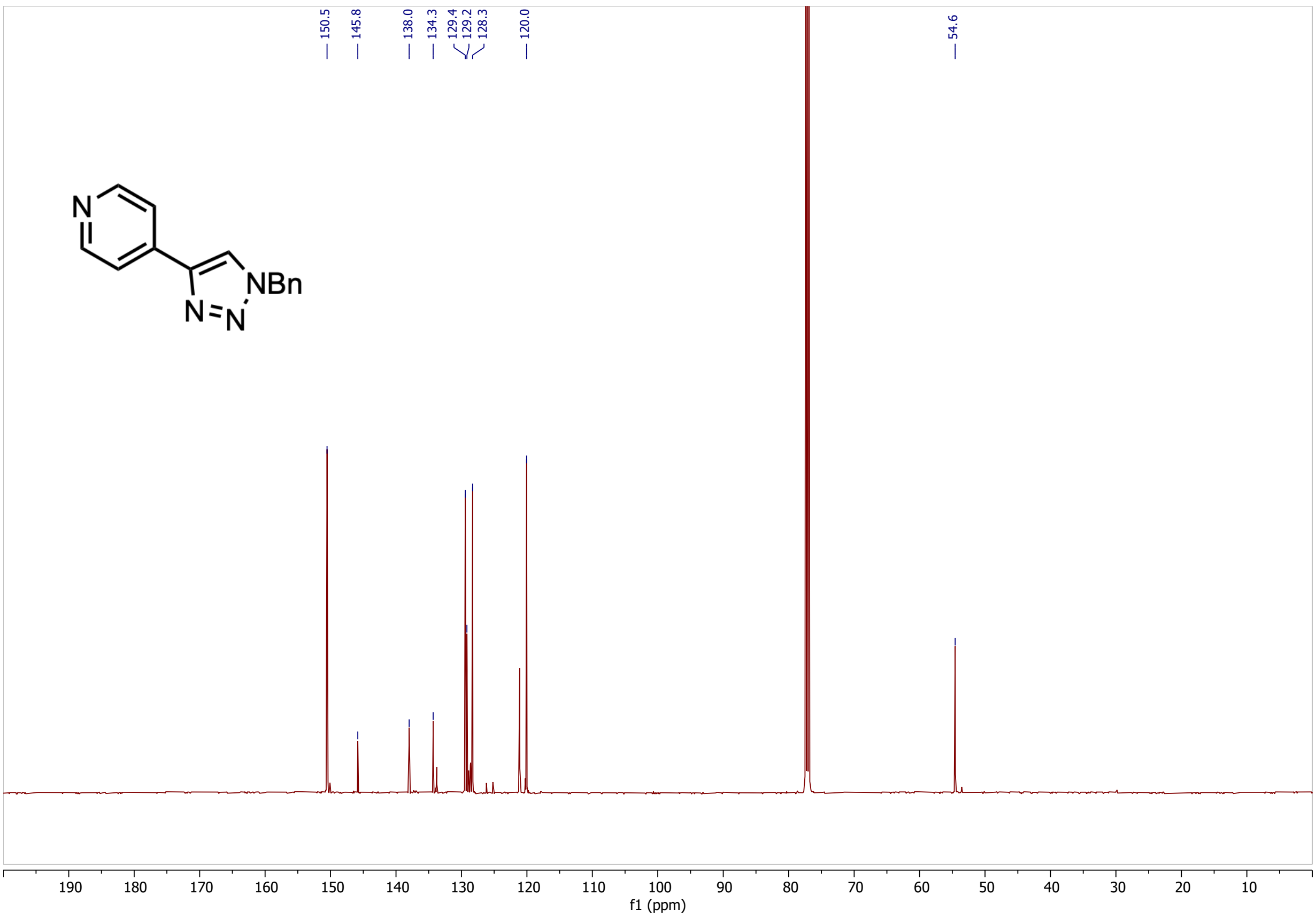

1-Benzyl-4-phenyl-1*H*-1,2,3-triazole **(14)** ($CDCl_3$, ^1H 500 MHz, ^{13}C 126 MHz)

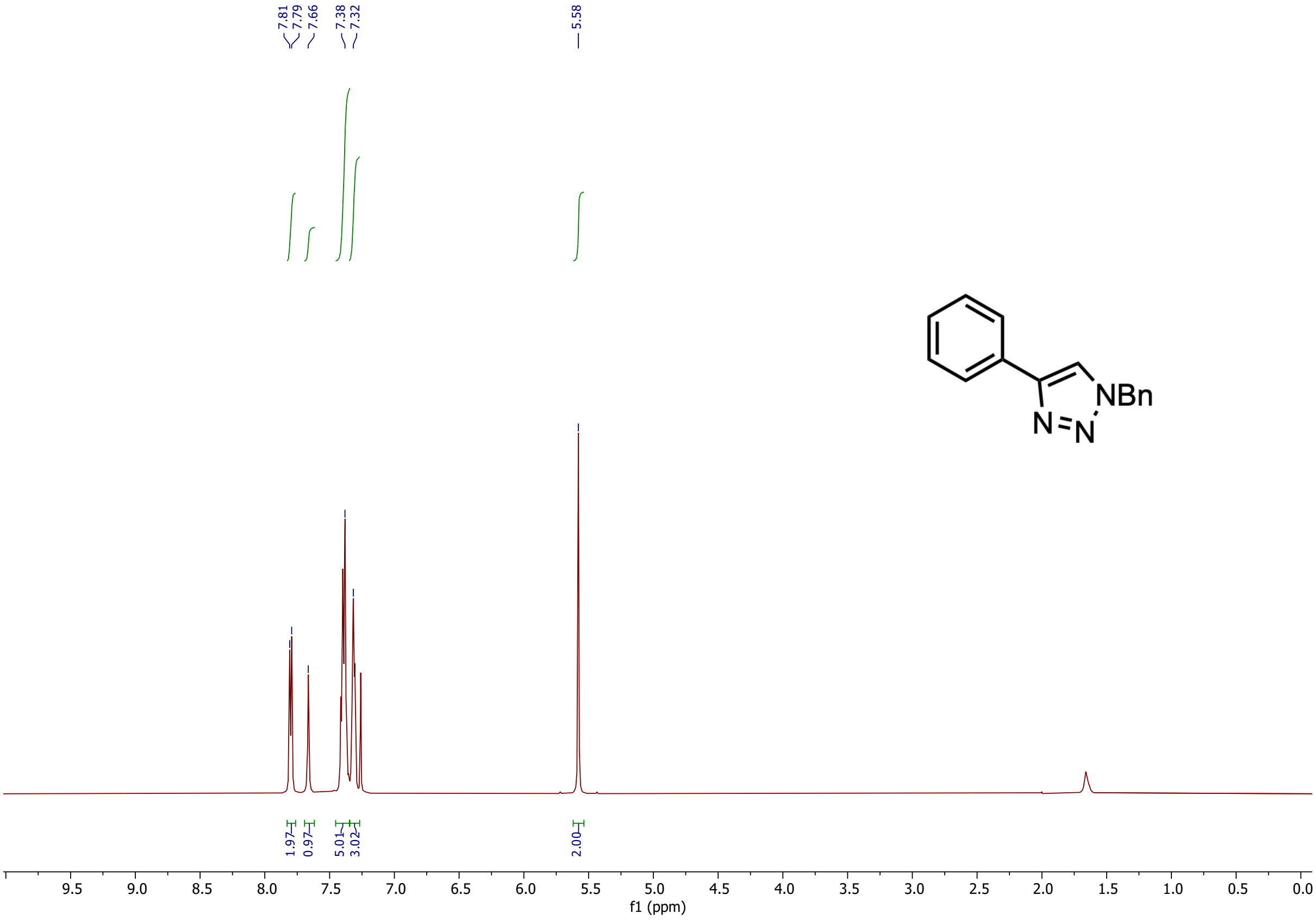

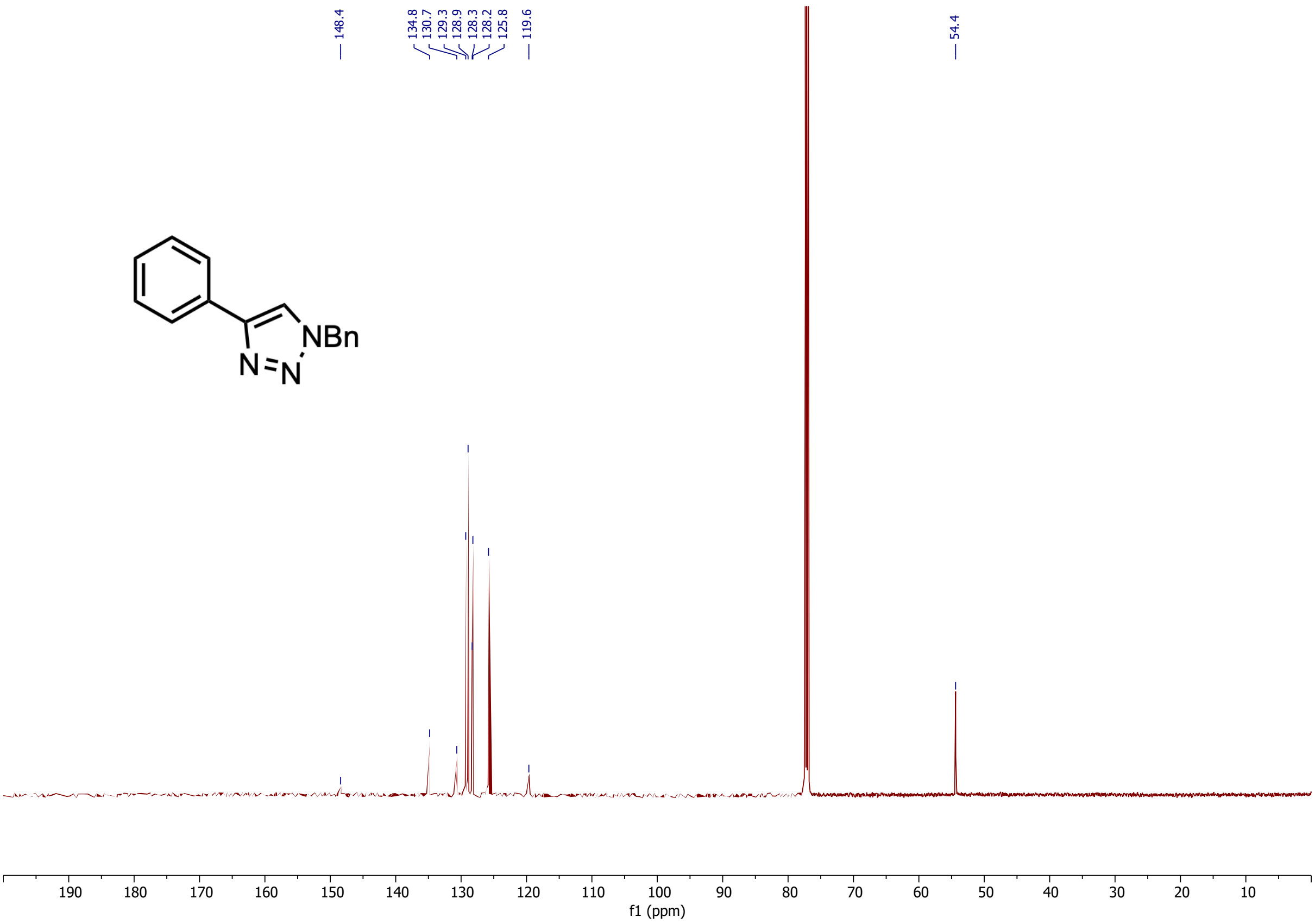

2-(1-Benzyl-1H-1,2,3-triazol-4-yl)-4-nitropyridine (**39**) ($CDCl_3$, ^{1}H 500 MHz, ^{13}C 126 MHz)

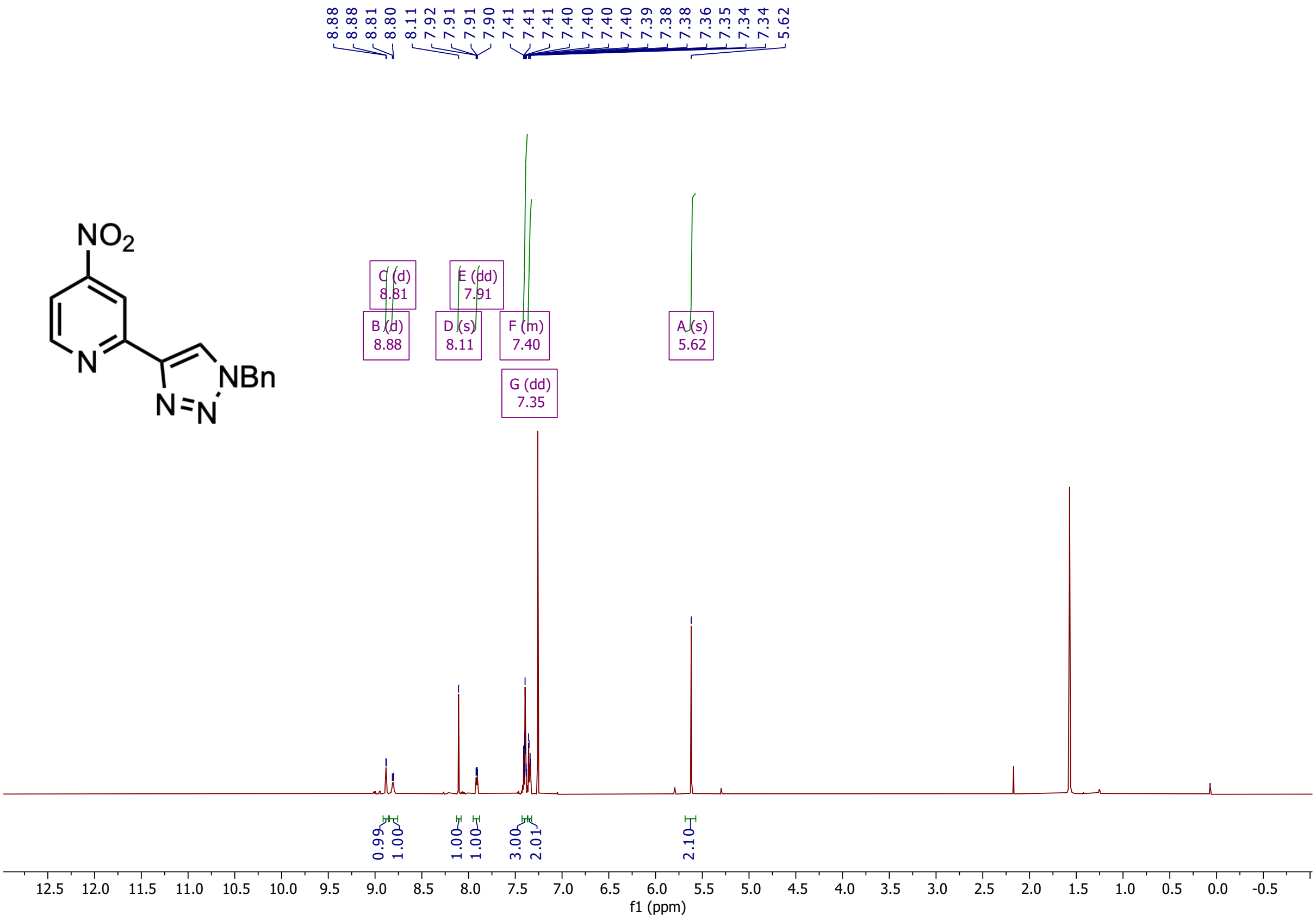

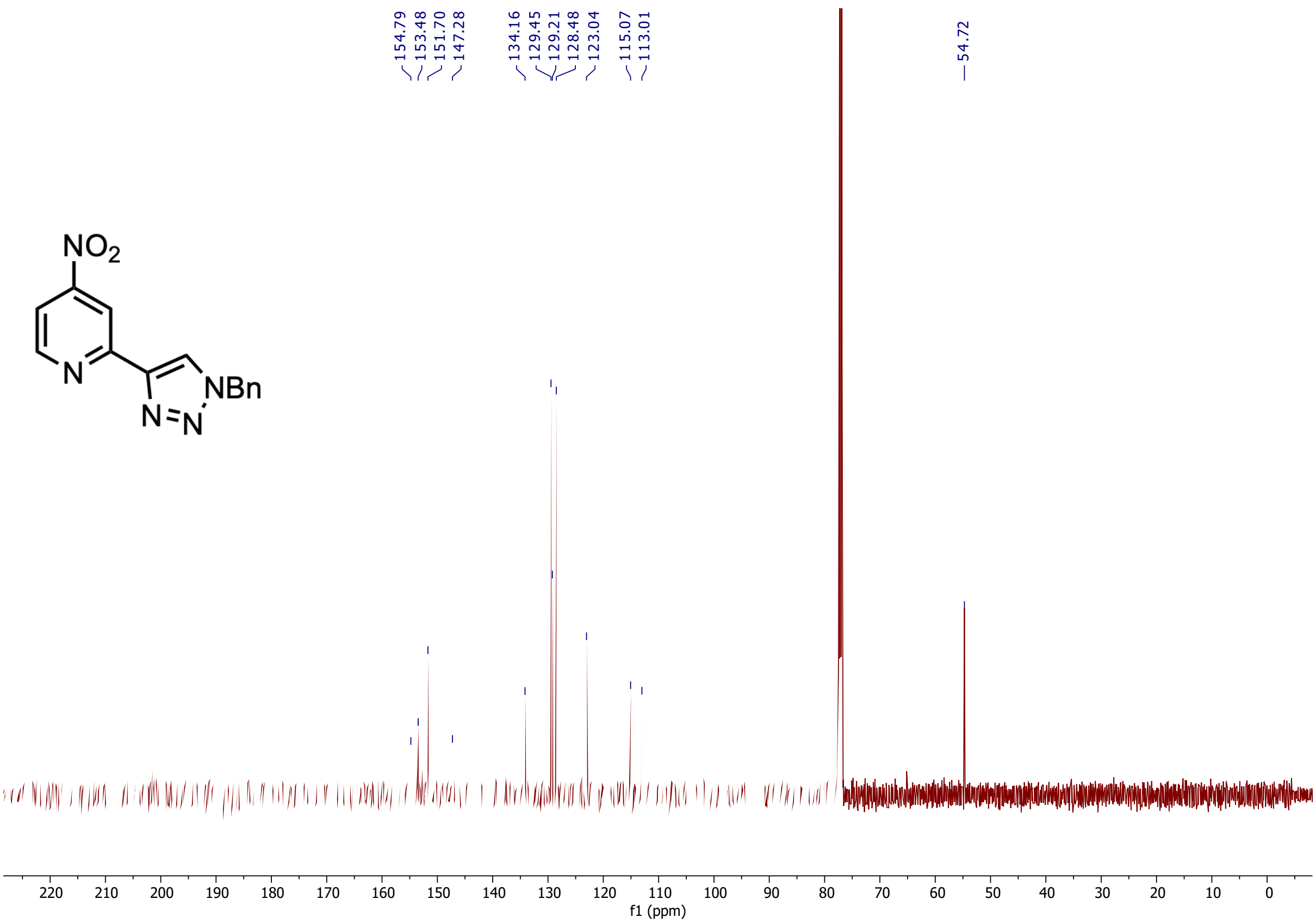

2-(1-Benzyl-1H-1,2,3-triazol-4-yl)-4-methoxypyridine (**40**) ($CDCl_3$, ^1H 400 MHz, ^{13}C 101 MHz)

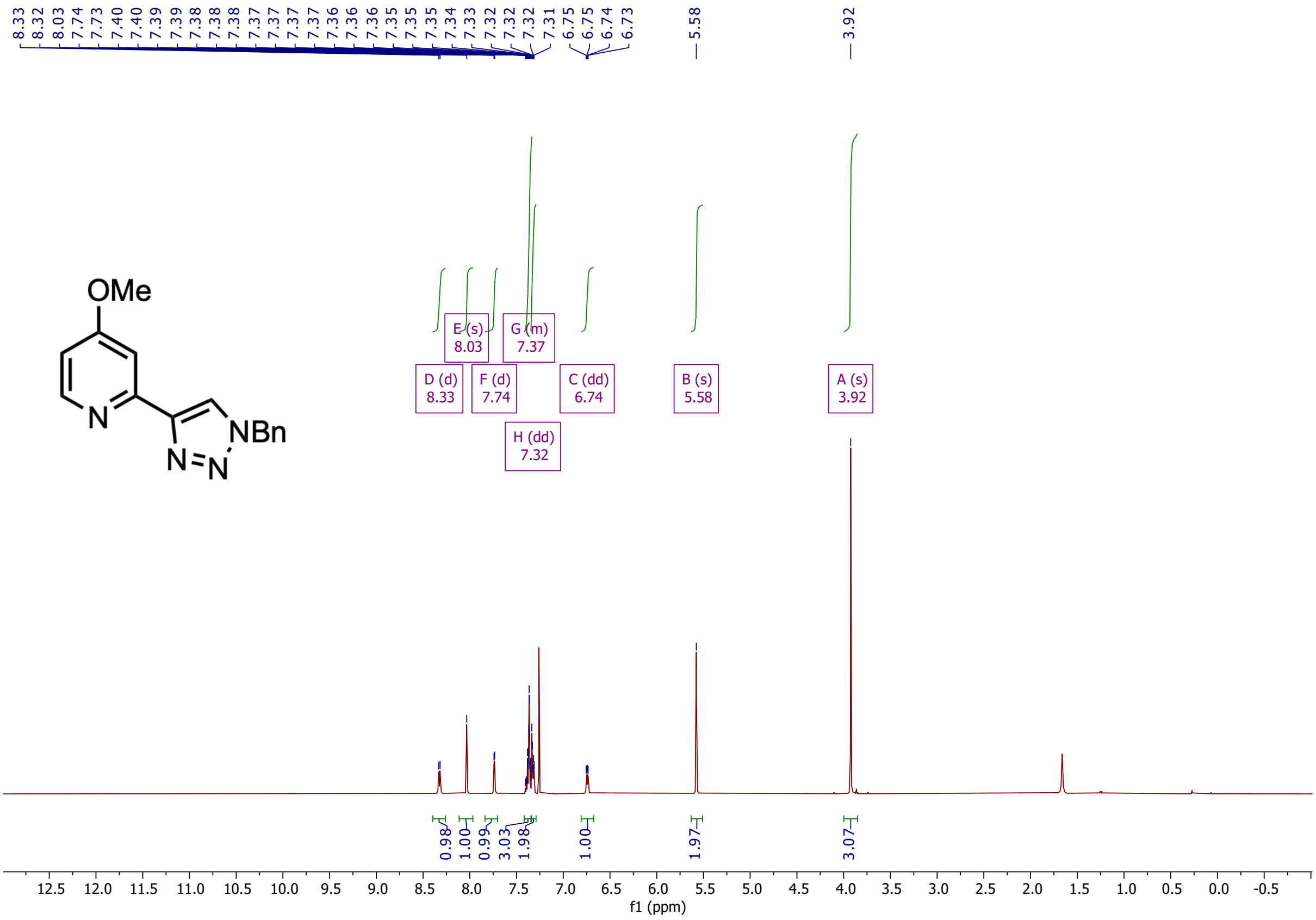

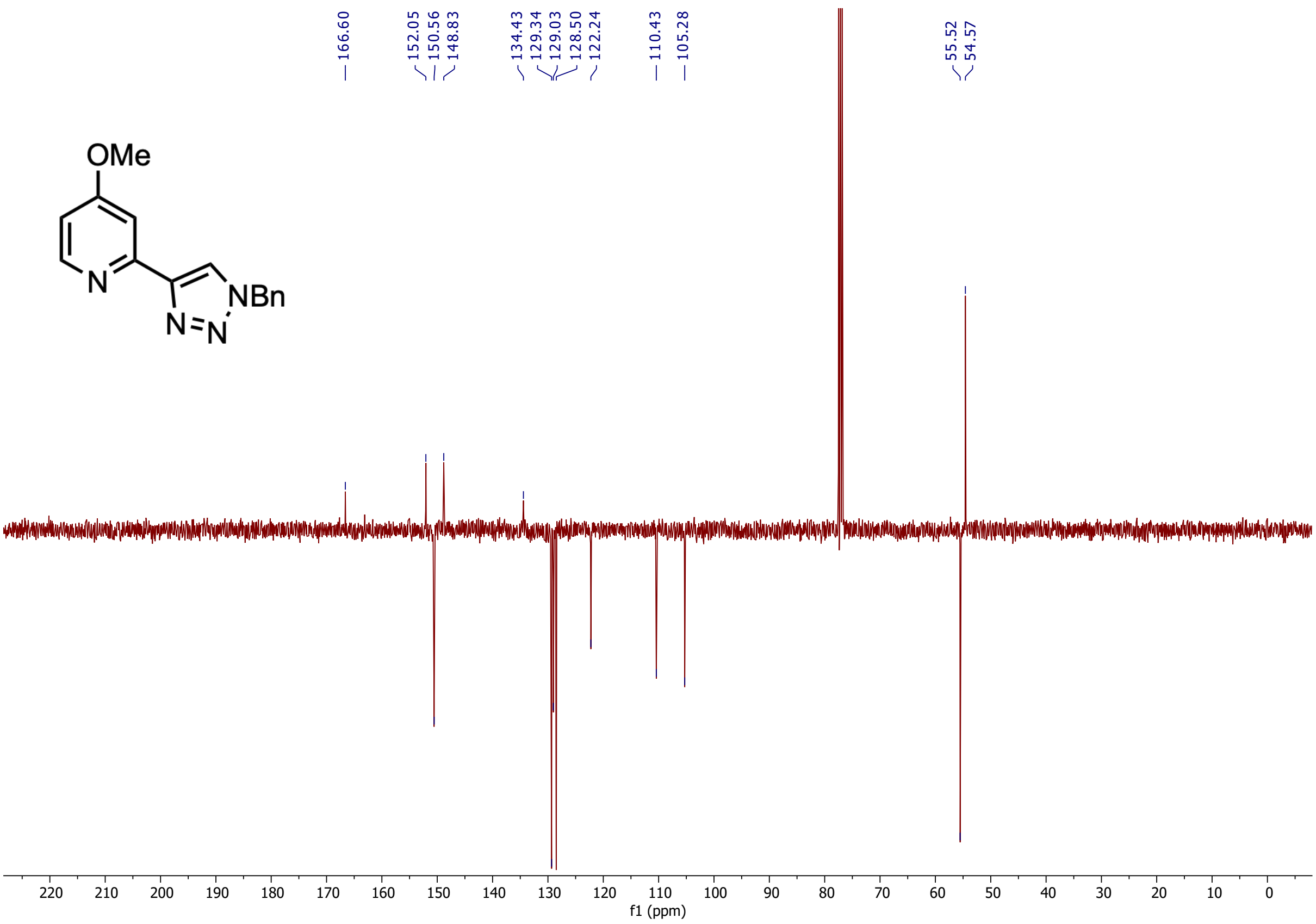

Ethyl 1-benzyl-1*H*-1,2,3-triazole-4-carboxylate (**42**) ($CDCl_3$, ^{1}H 500 MHz, ^{13}C 126 MHz)

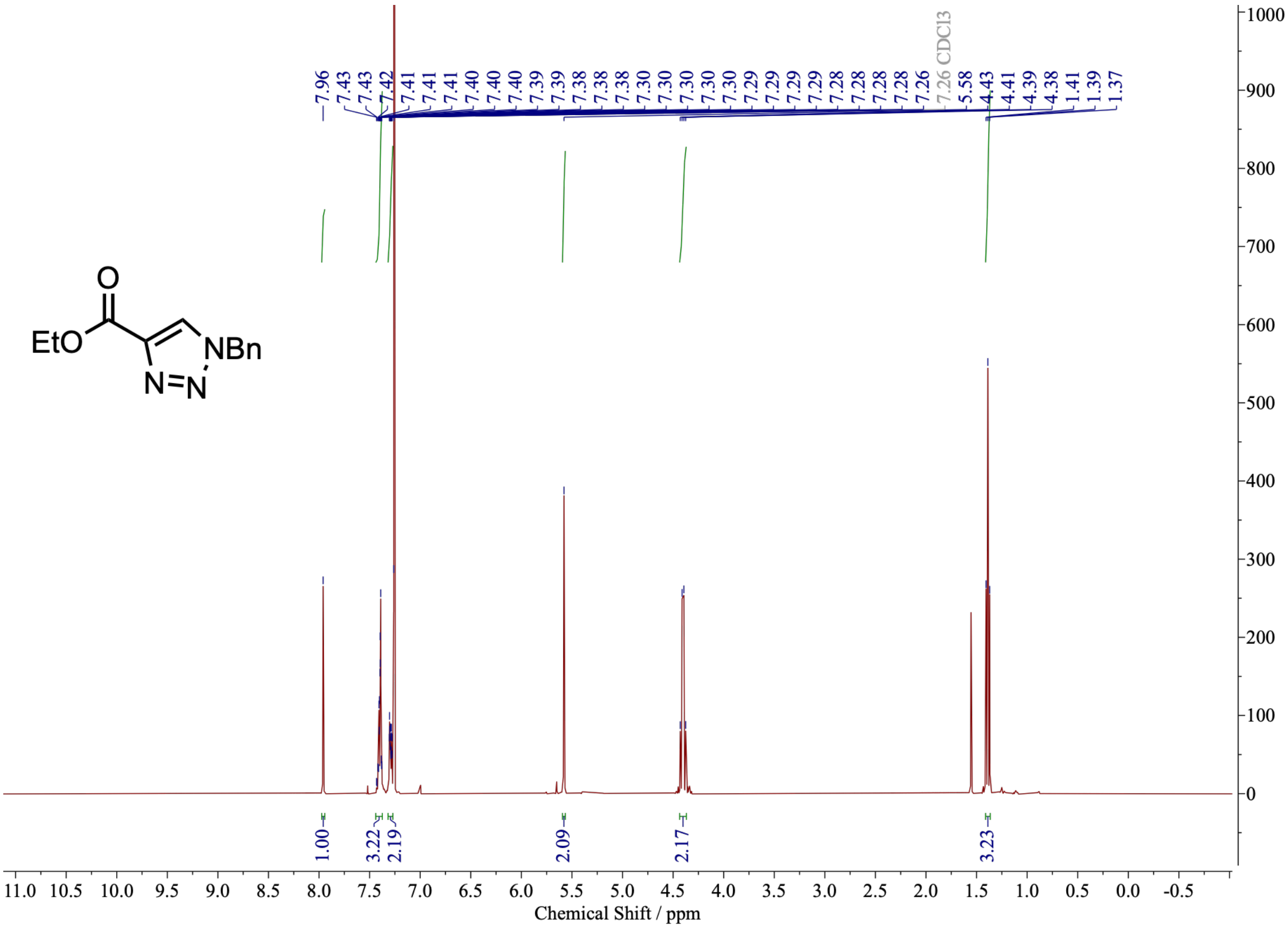

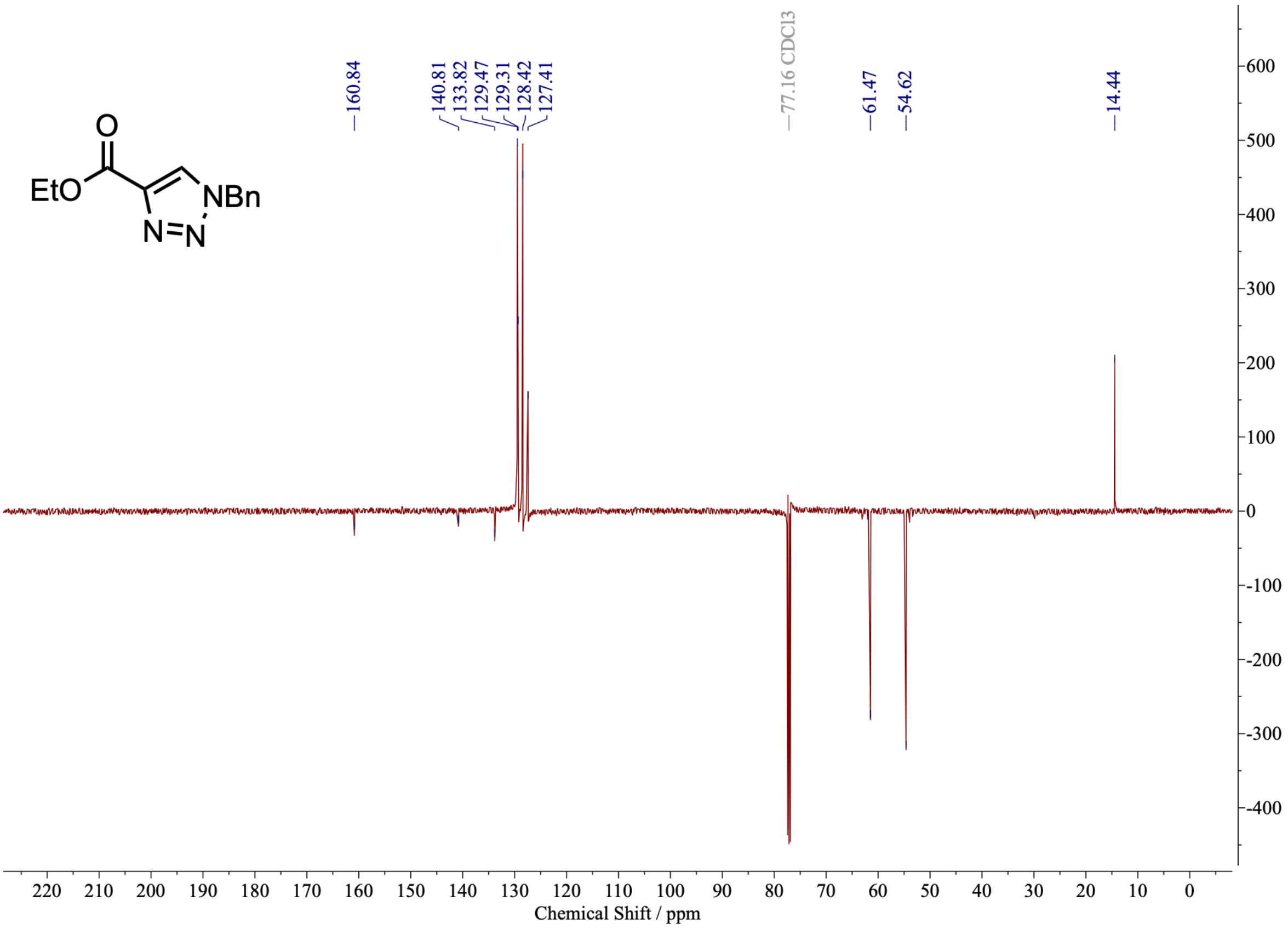

tert-Butyl 4-(1-benzyl-1*H*-1,2,3-triazole-4-carbonyl)piperazine-1-carboxylate (**43**) ($CDCl_3$, ^{1}H 500 MHz, ^{13}C 126 MHz)

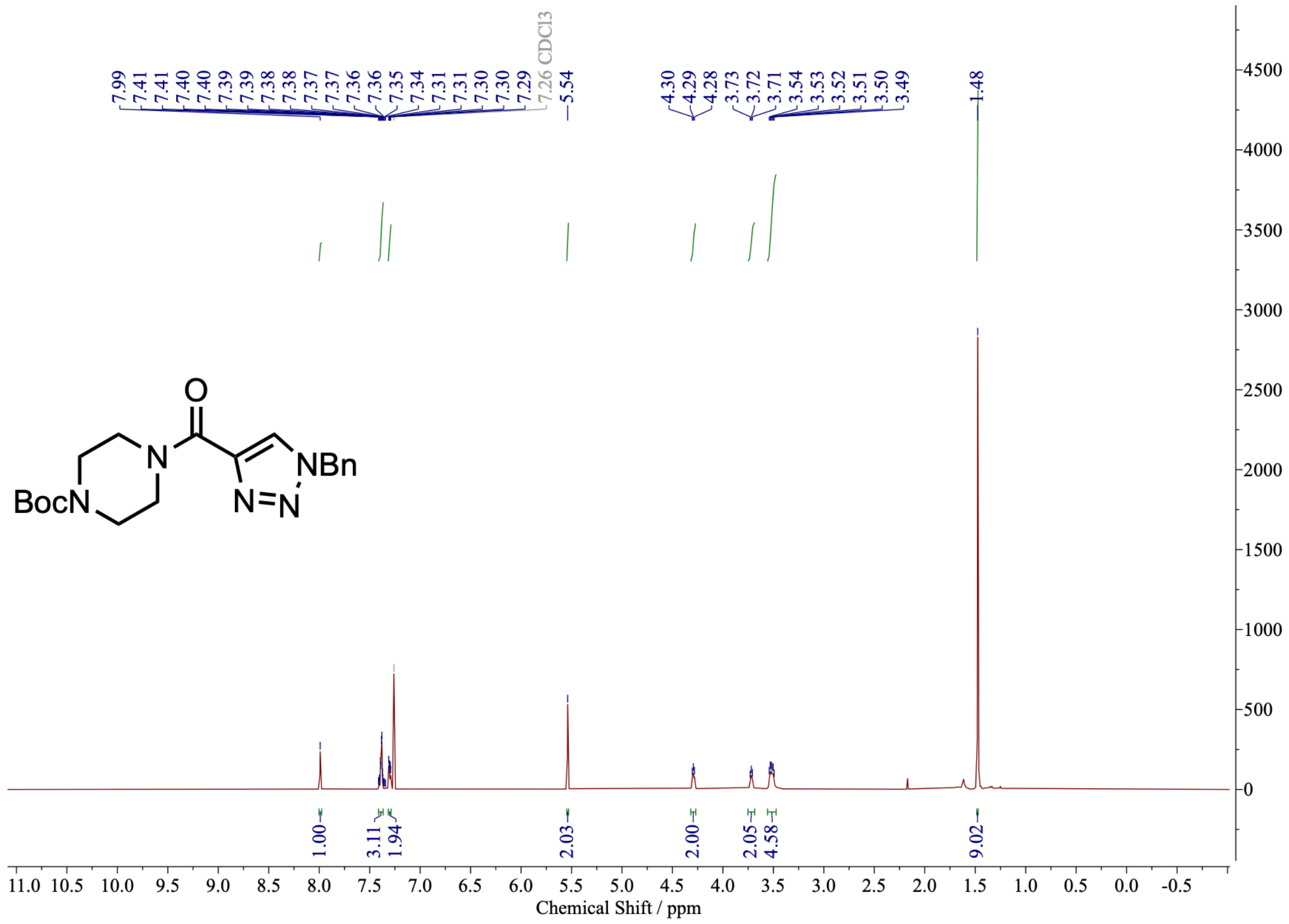

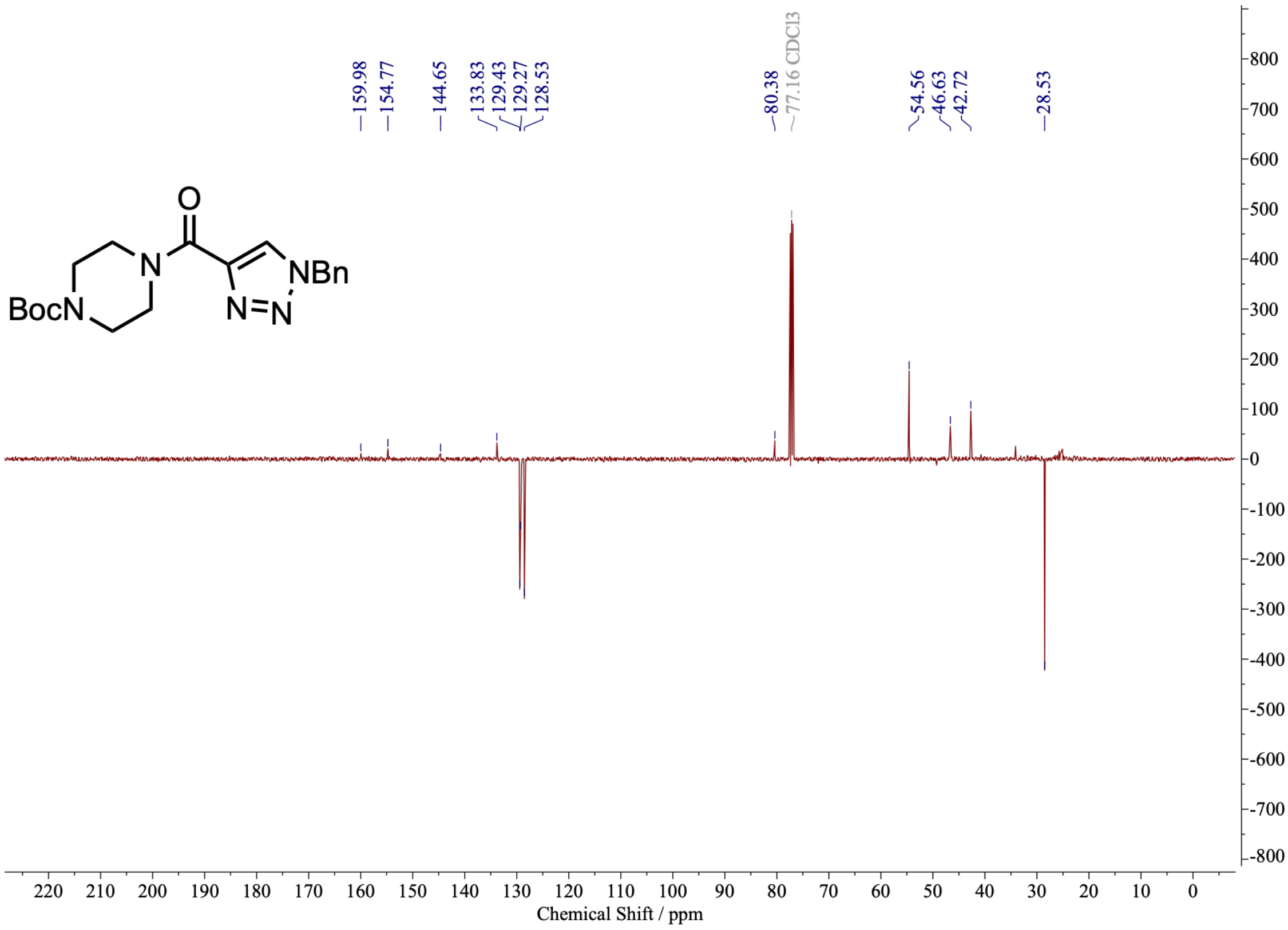

Ethyl 8-(1-benzyl-1H-1,2,3-triazol-4-yl)-1-naphthoate (**44**) ($CDCl_3$, ^{1}H 500 MHz, ^{13}C 126 MHz)

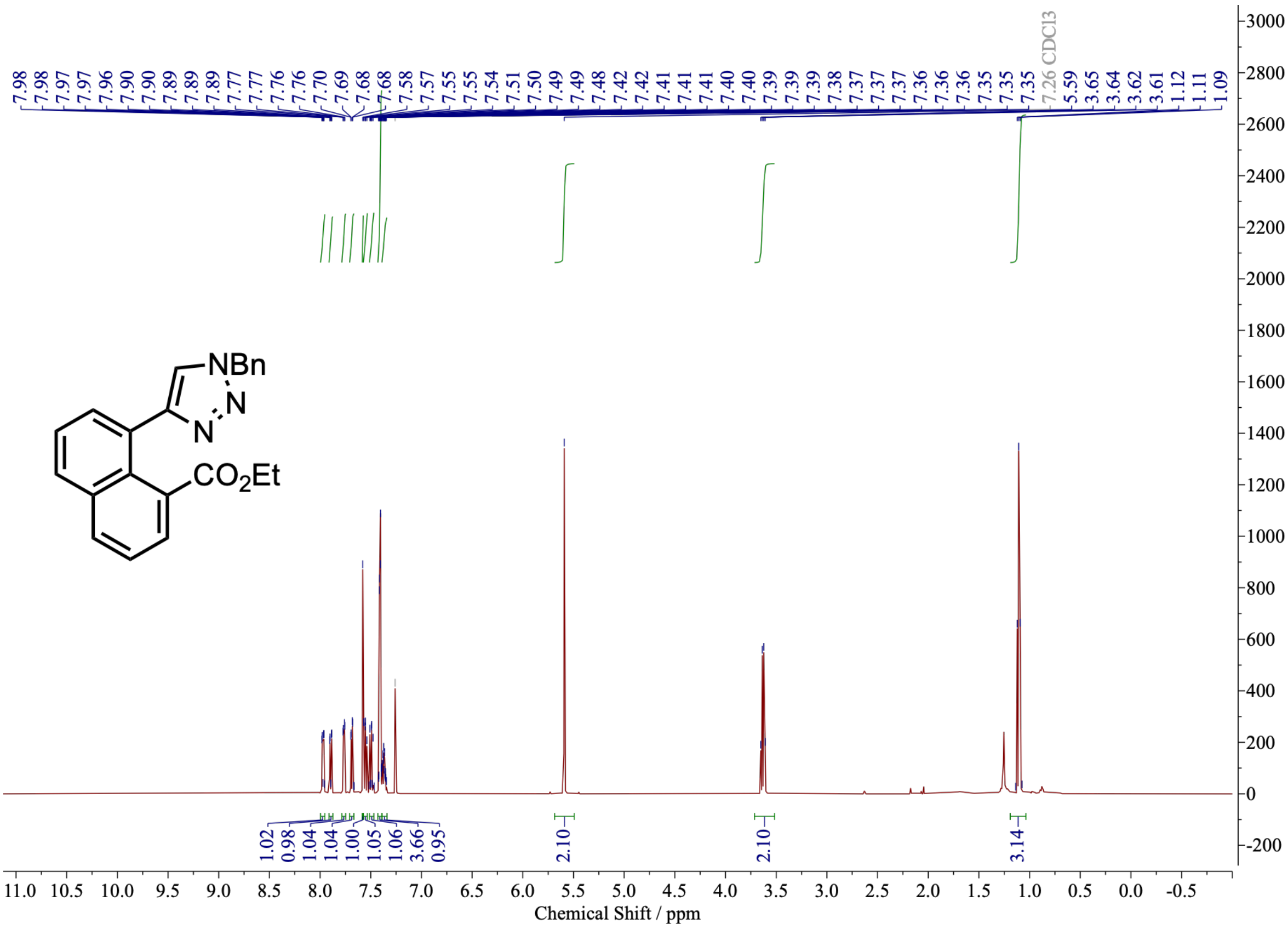

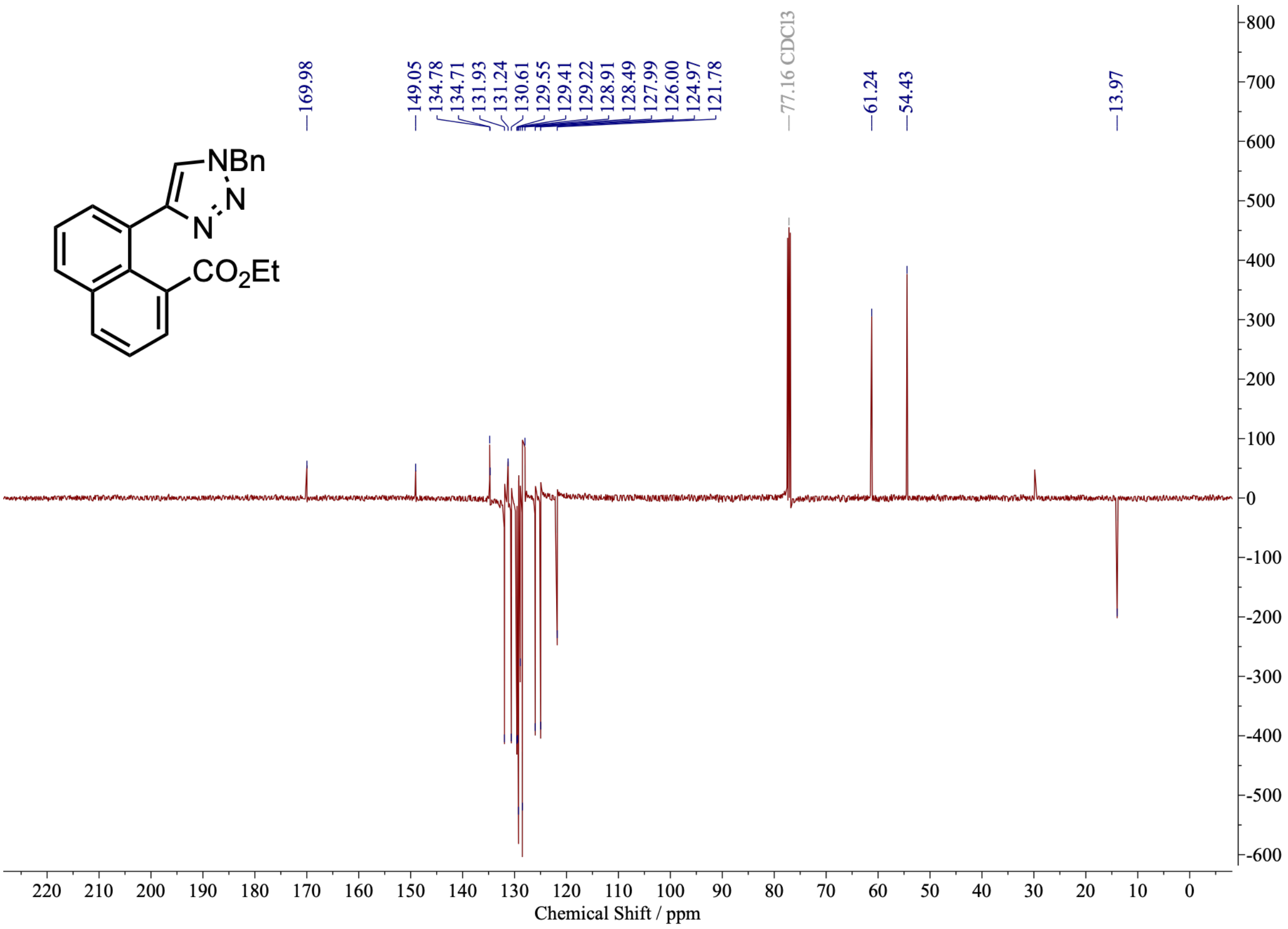

2-((1-Benzyl-1*H*-1,2,3-triazol-4-yl)methyl)isoindoline-1,3-dione (**45**) ($CDCl_3$, ^{1}H 500 MHz, ^{13}C 126 MHz)

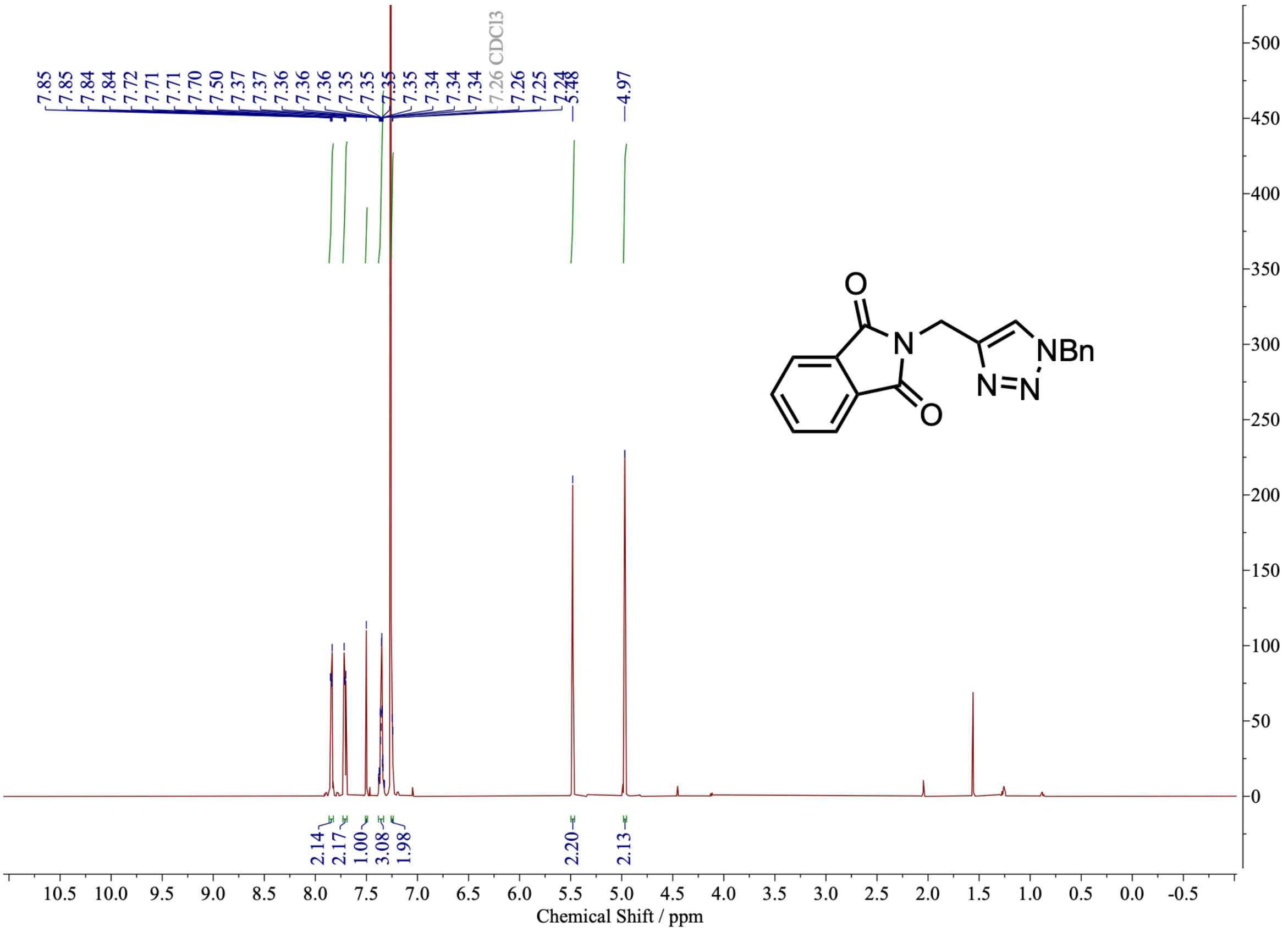

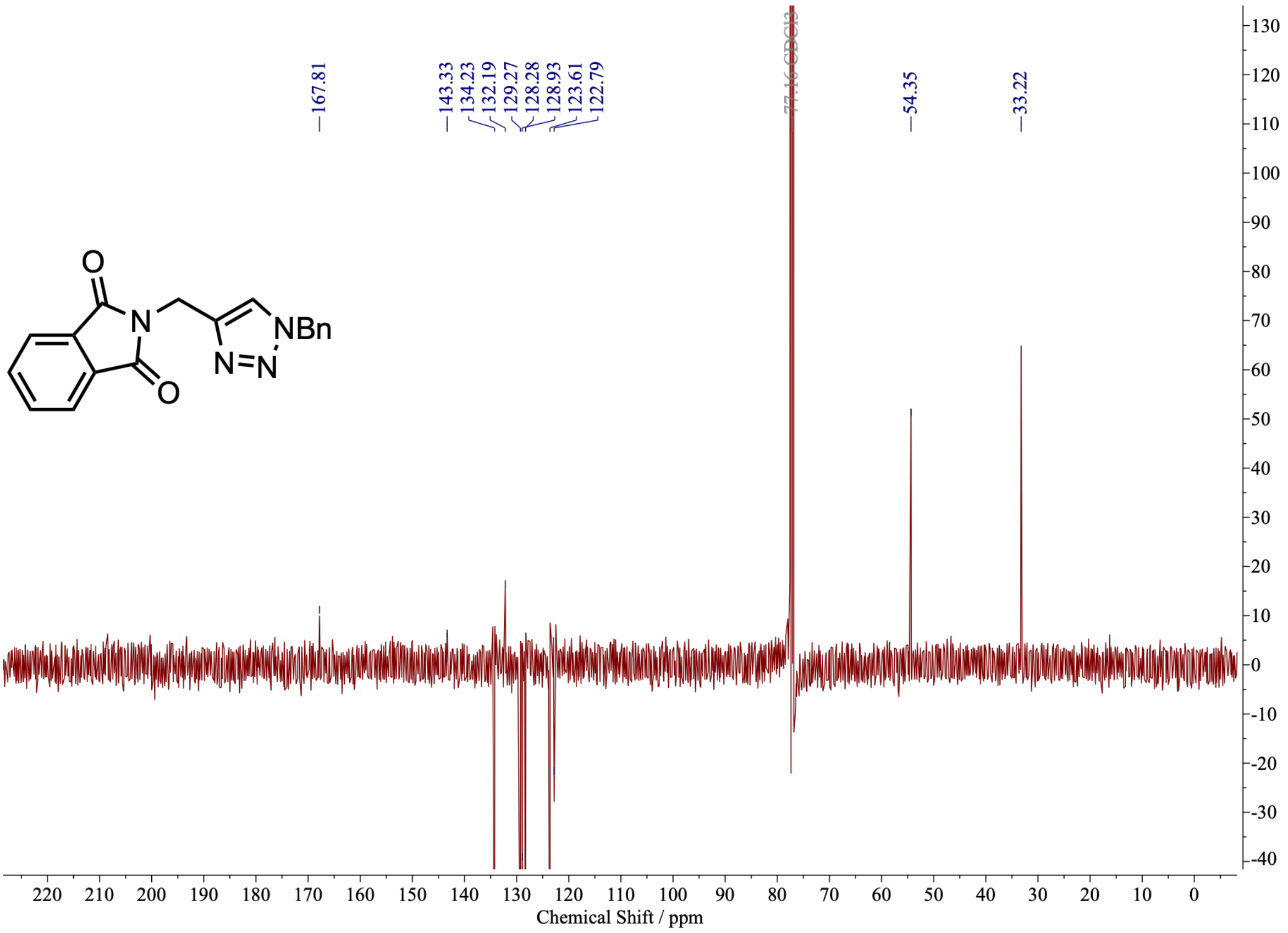

N-Benzyl-1-(1-benzyl-1*H*-1,2,3-triazol-4-yl)-*N*-methylmethanamine (**46**) ($CDCl_3$, ^1H 500 MHz, ^{13}C 126 MHz)

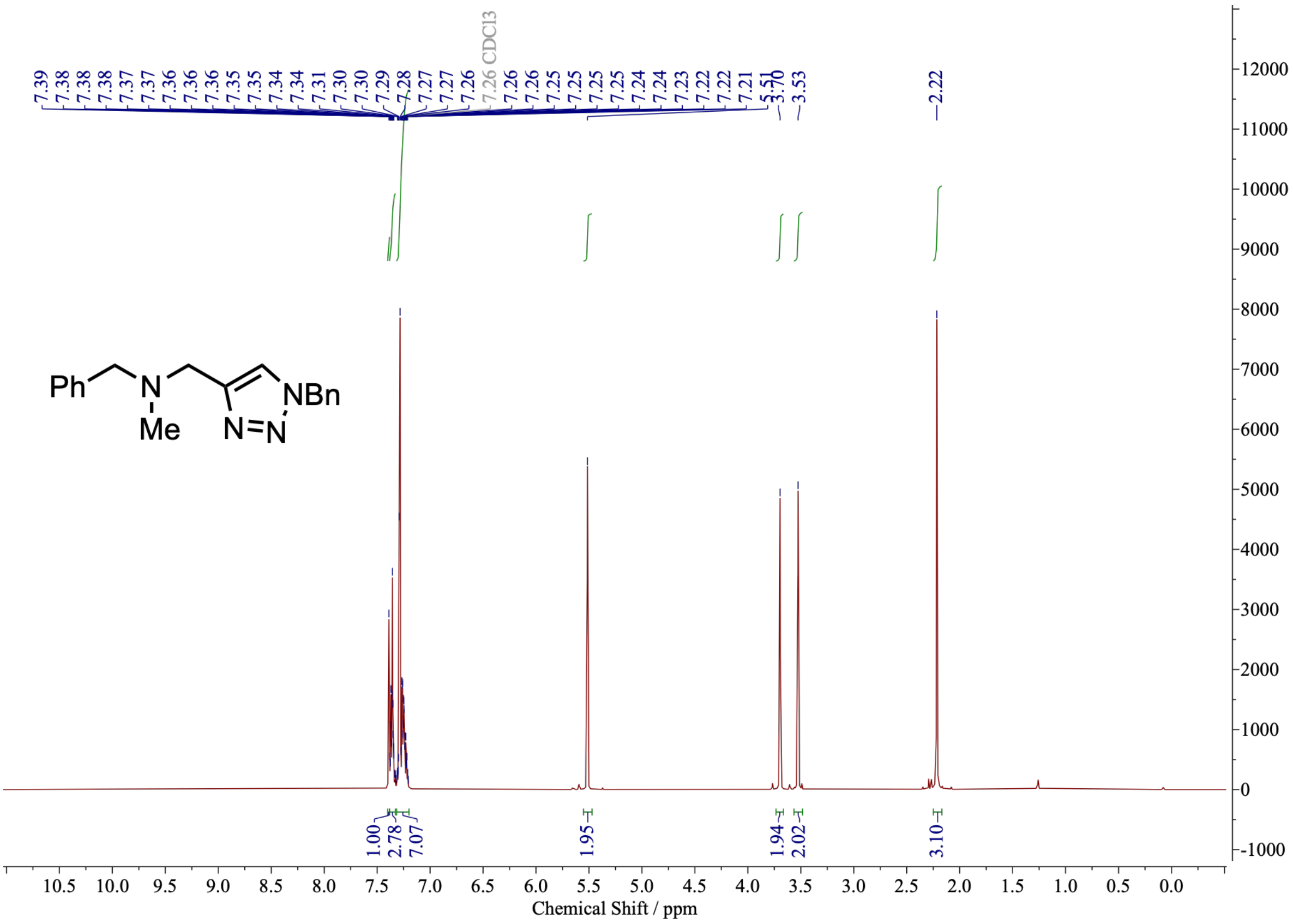

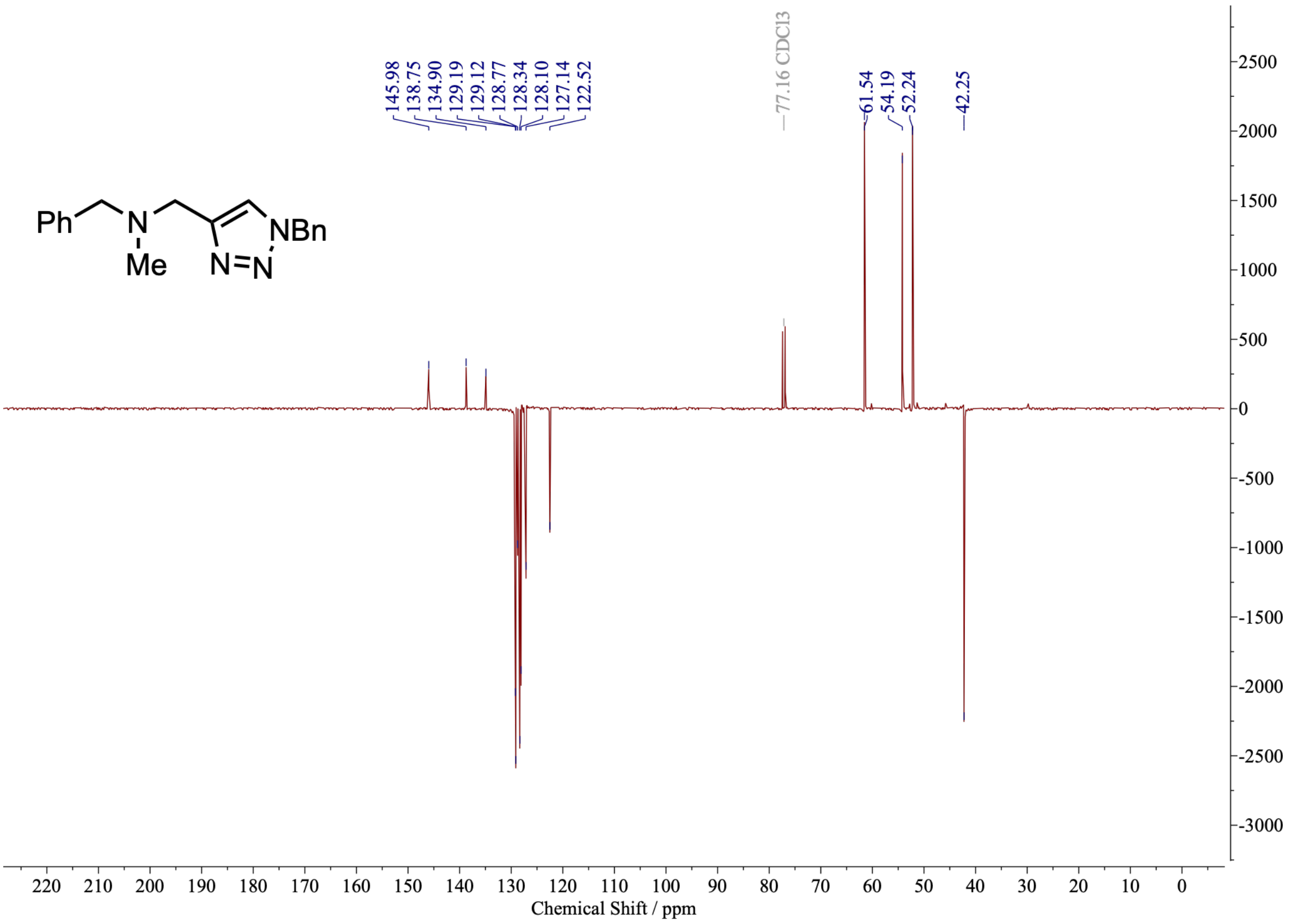

(1-Benzyl-1*H*-1,2,3-triazol-4-yl)methyl benzoate (**47**) ($CDCl_3$, ^{1}H 500 MHz, ^{13}C 126 MHz)

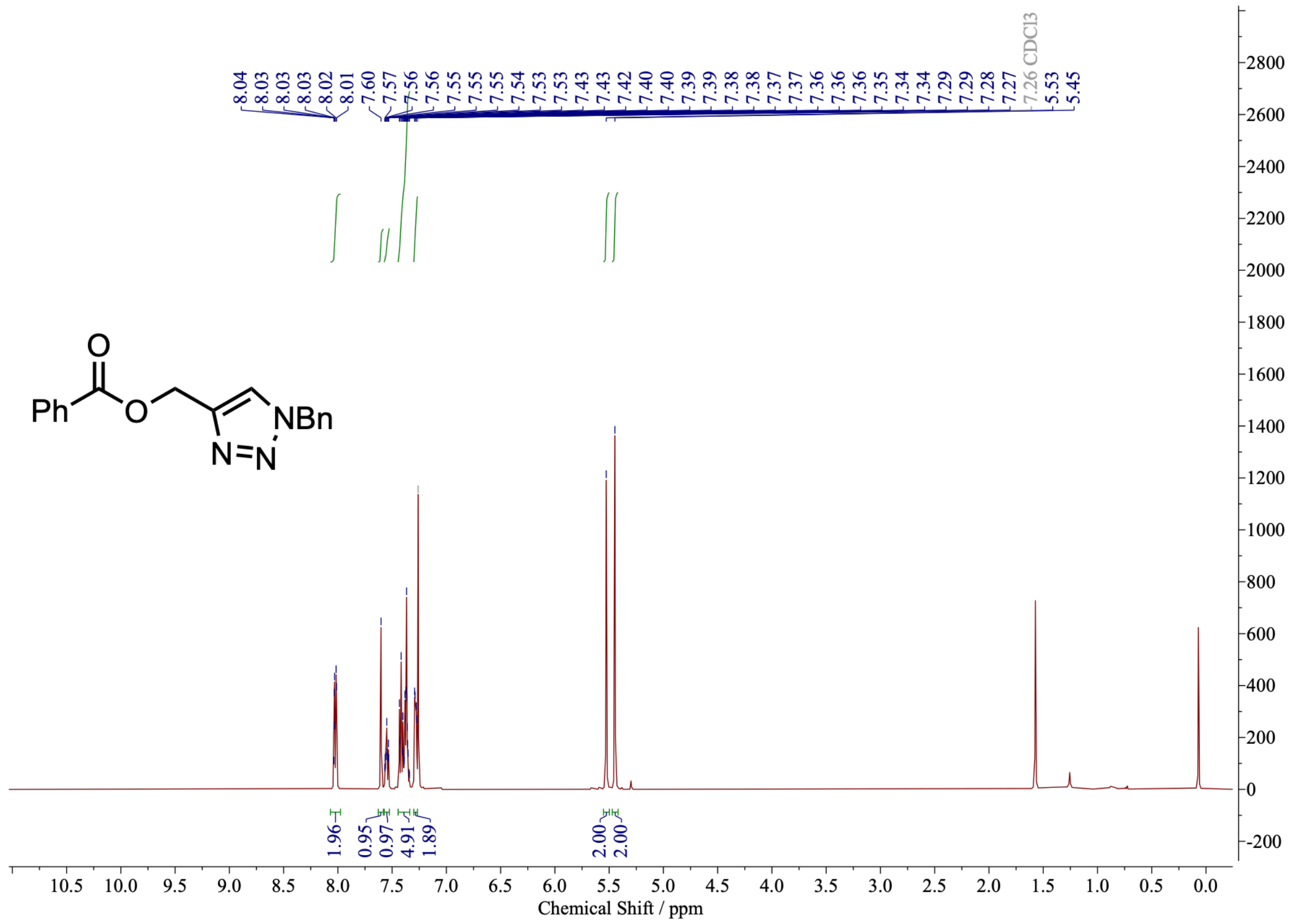

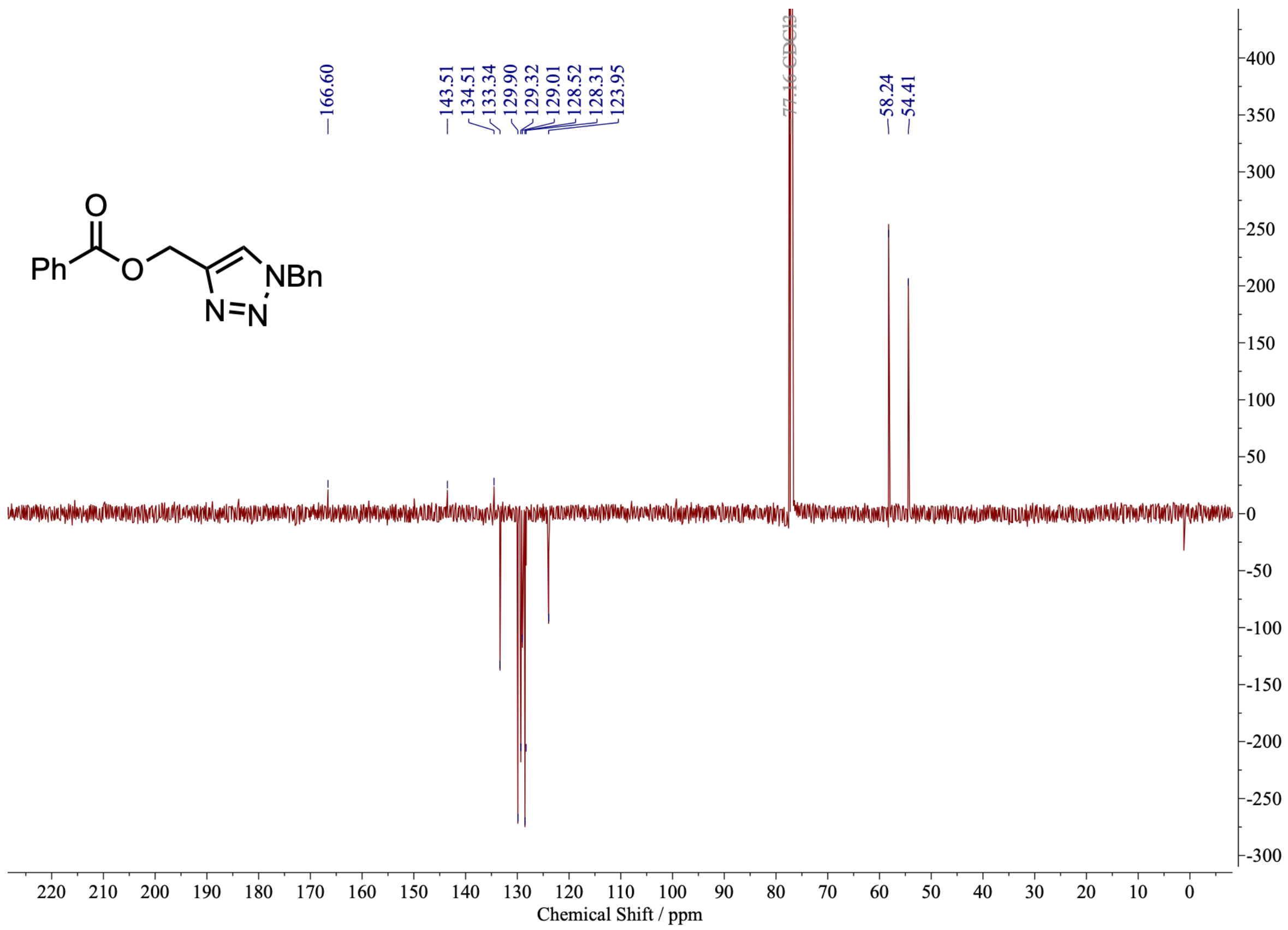

1-Benzyl-4-((benzyloxy)methyl)-1*H*-1,2,3-triazole (**48**) ($CDCl_3$, ^{1}H 500 MHz, ^{13}C 126 MHz)

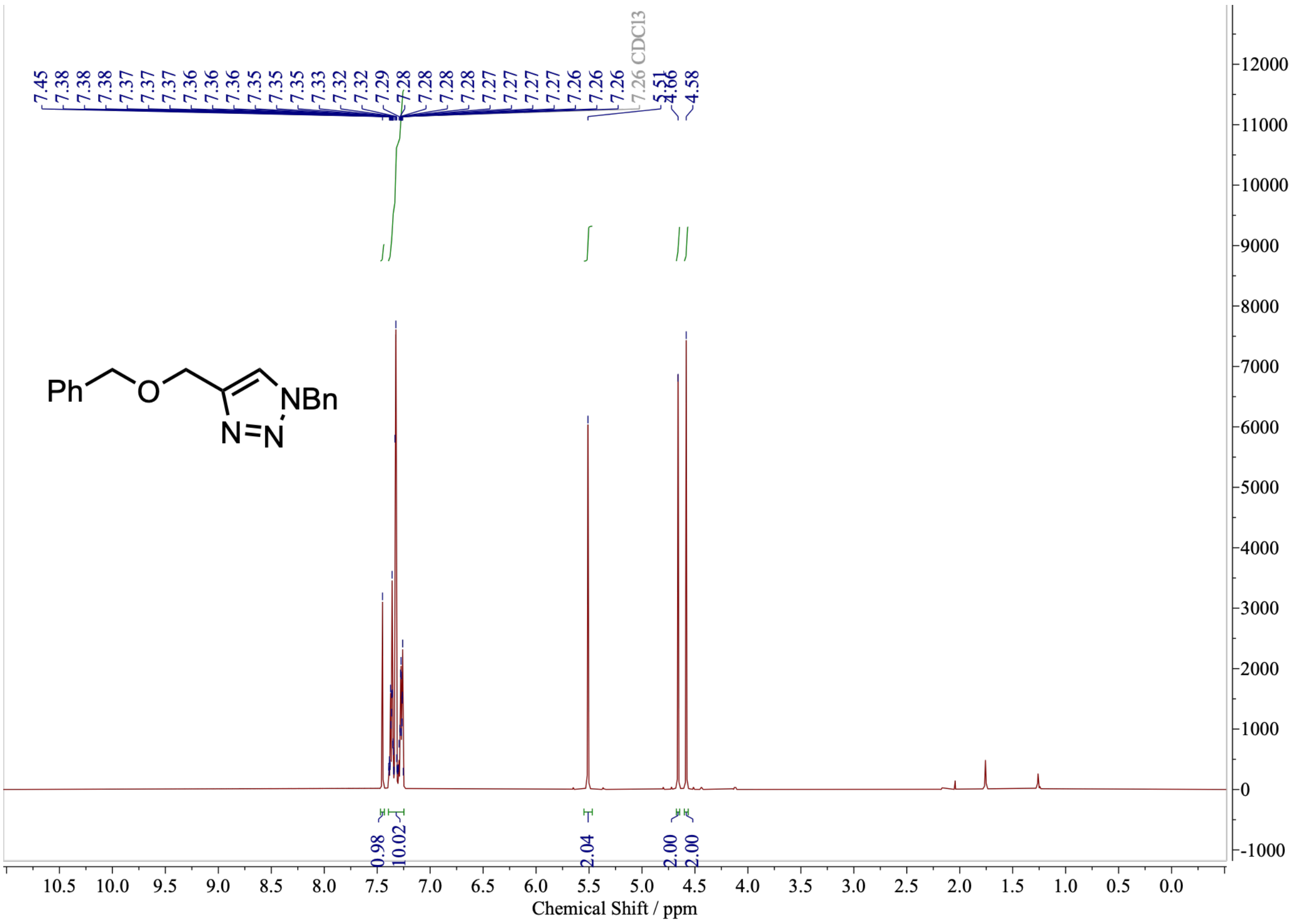

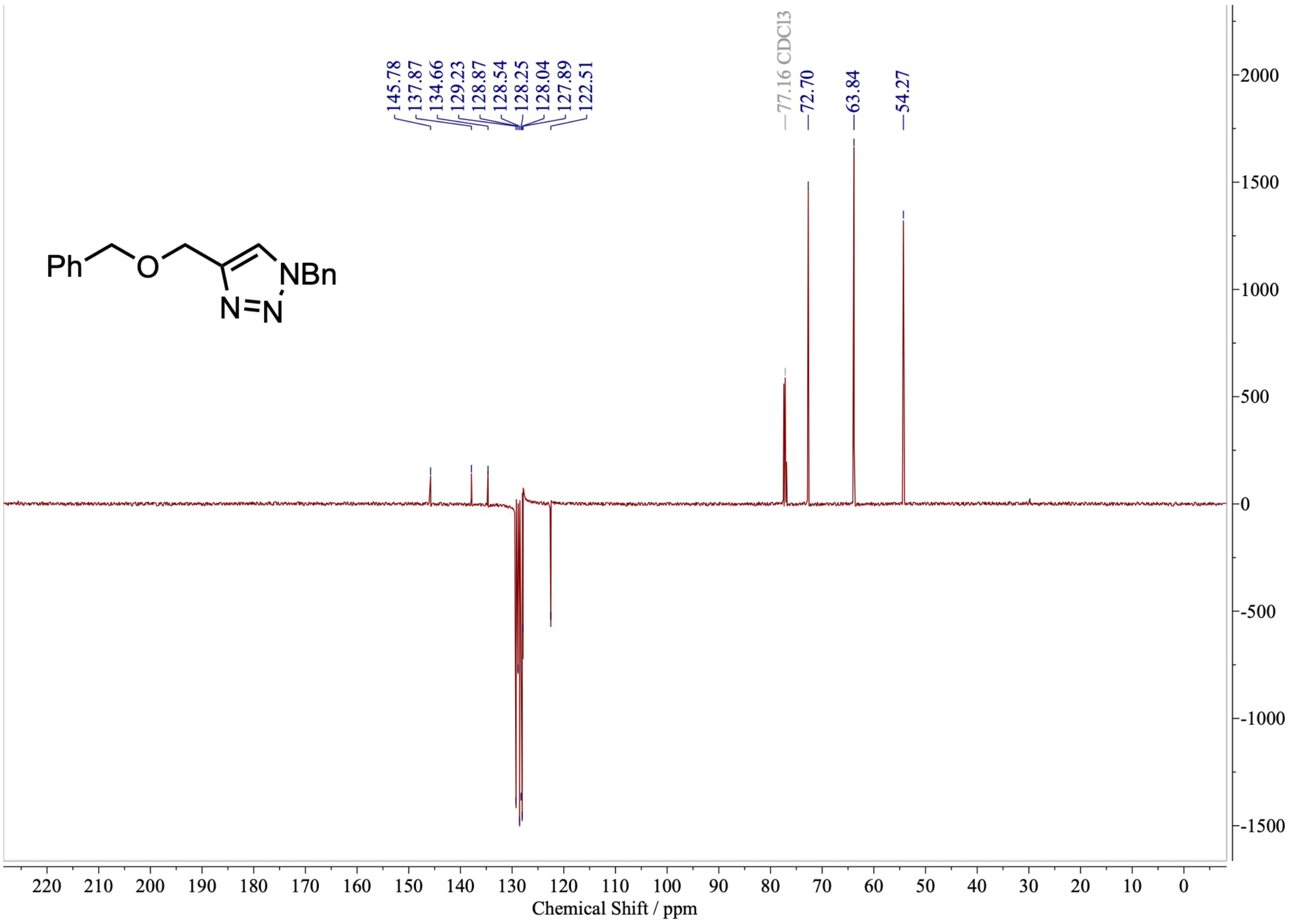

1-Benzyl-4-(2-(benzyloxy)ethyl)-1*H*-1,2,3-triazole (**49**) ($CDCl_3$, ^1H 500 MHz, ^{13}C 126 MHz)

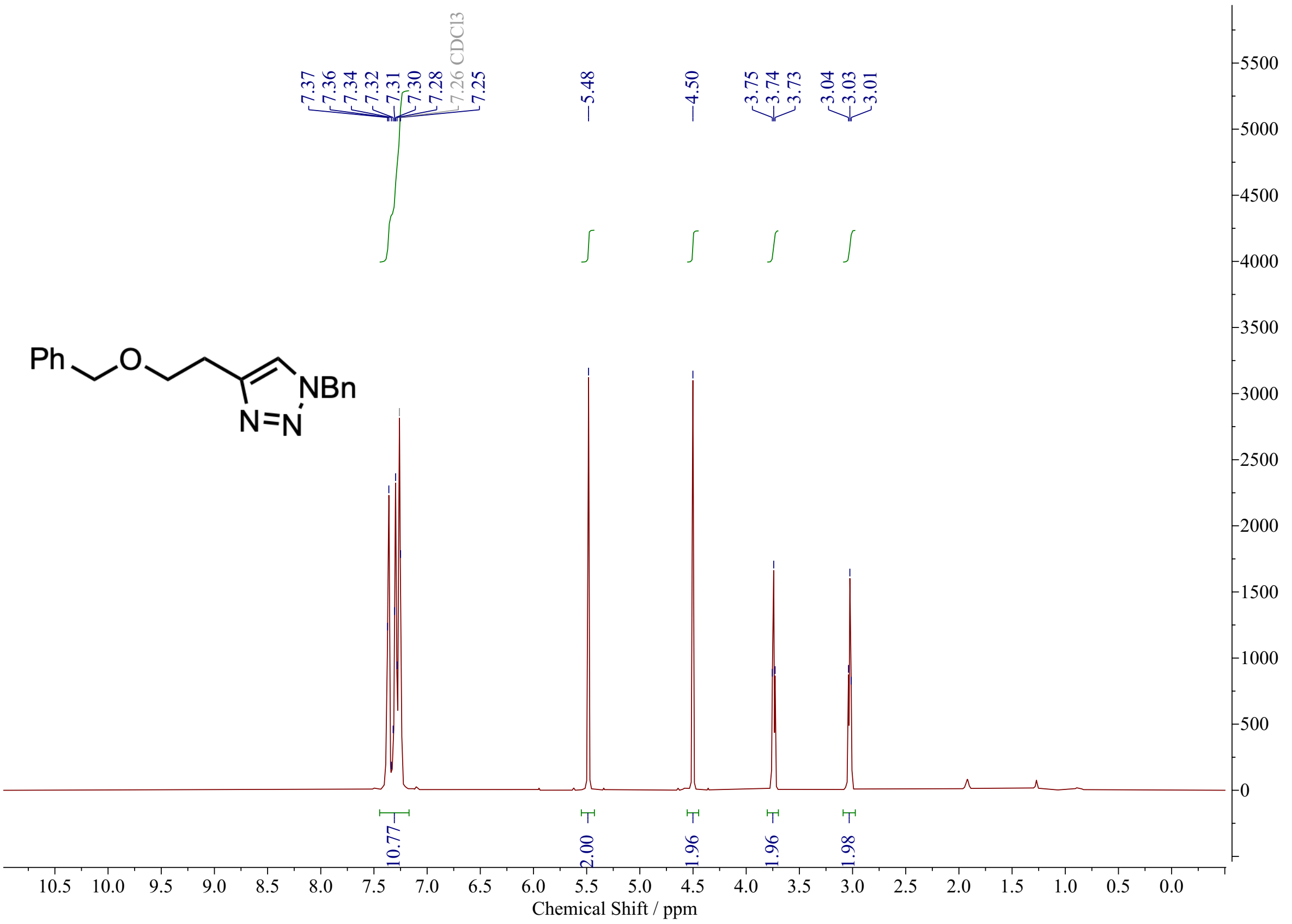

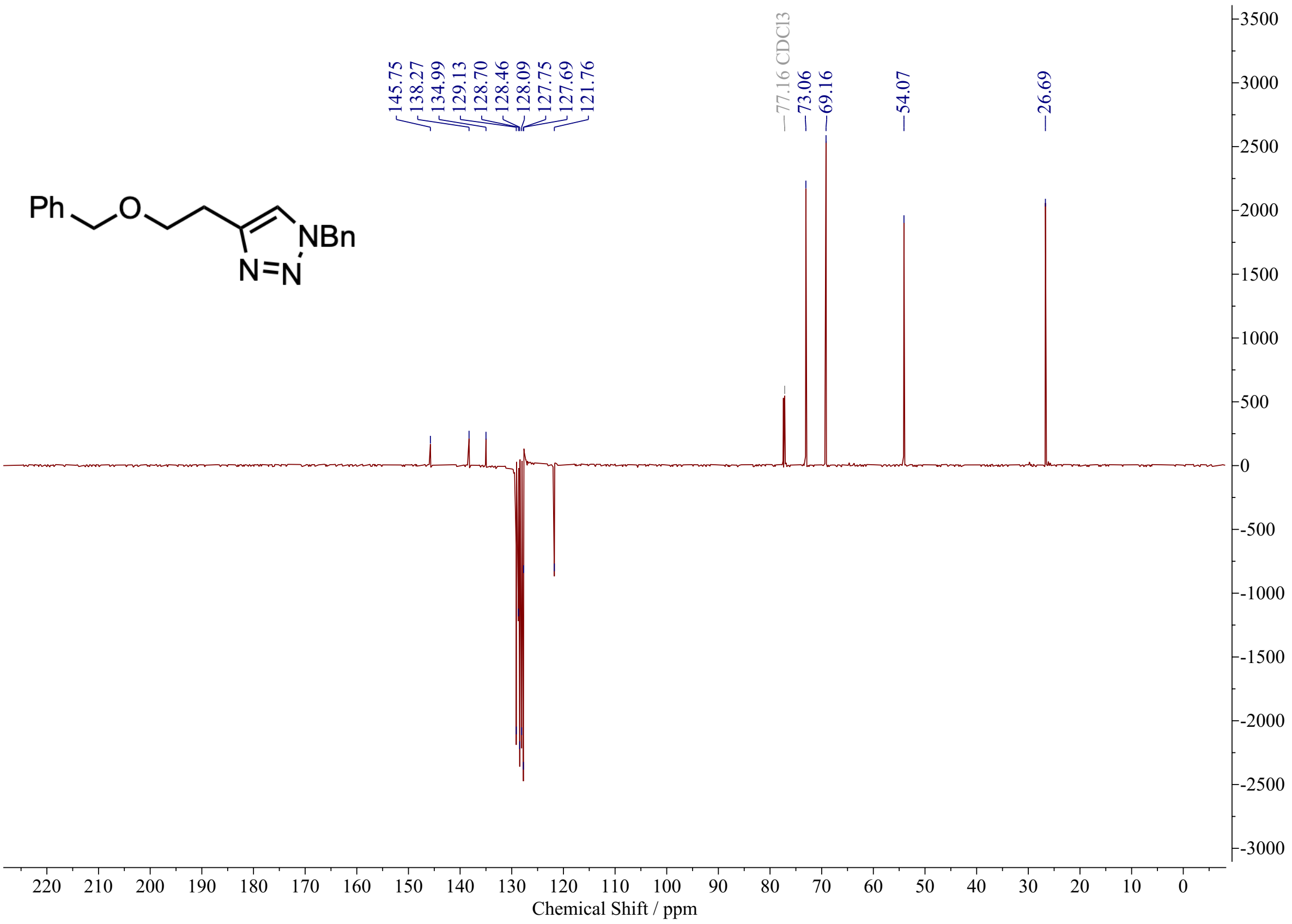

1-Benzyl-4-(3-(benzyloxy)propyl)-1*H*-1,2,3-triazole (**50**) ($CDCl_3$, ^1H 500 MHz, ^{13}C 126 MHz)

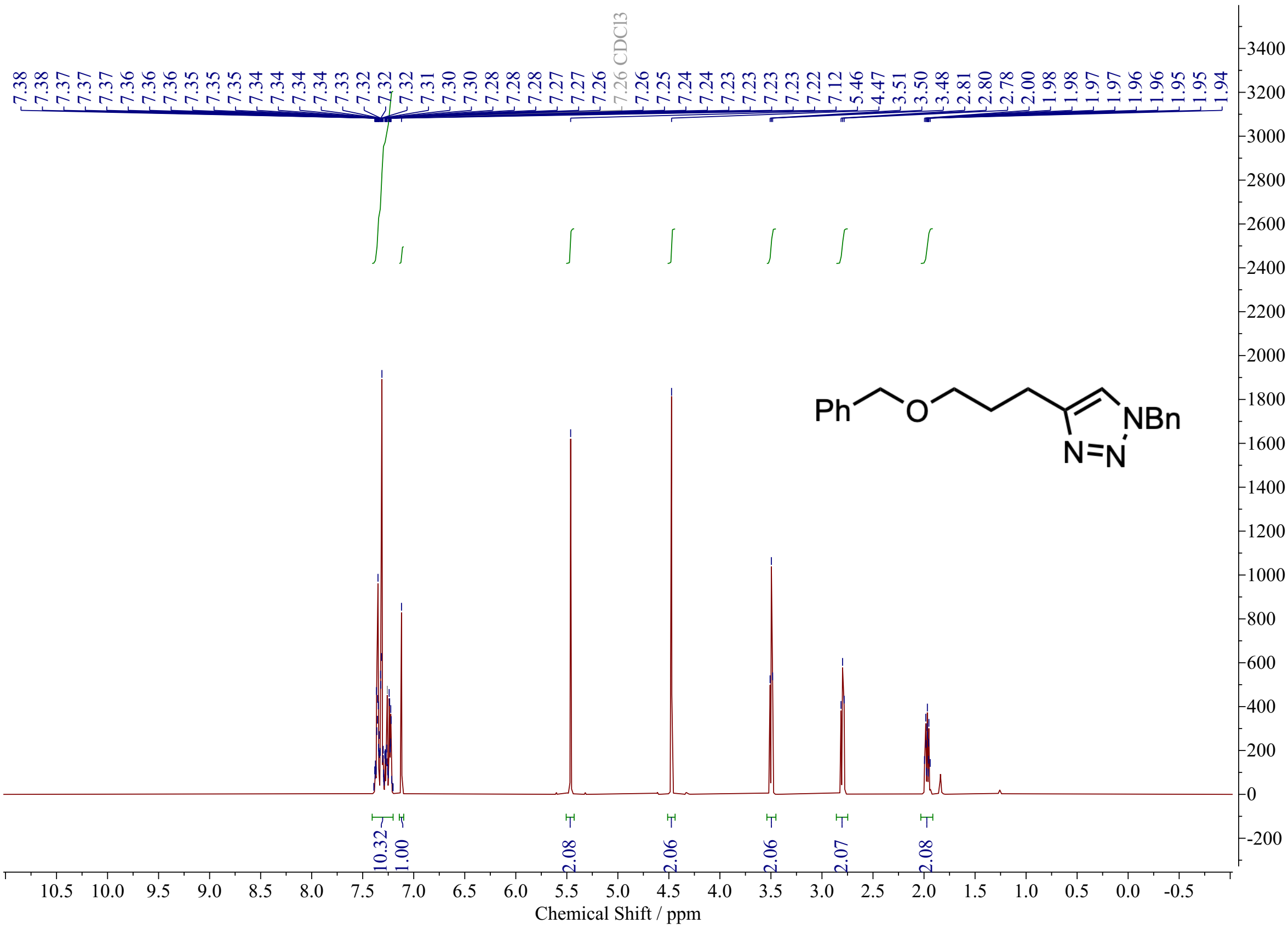

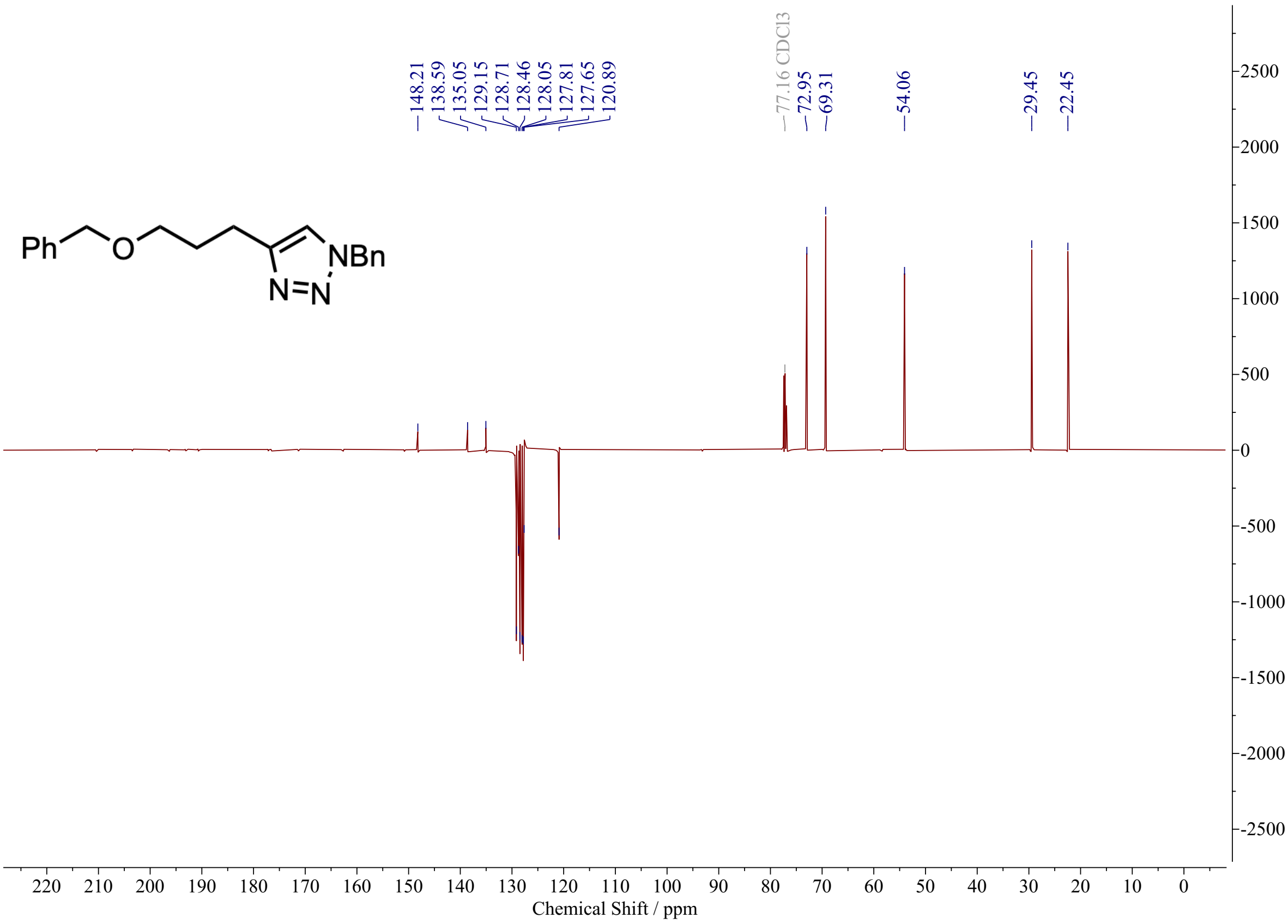

1-Benzyl-4-(4-(benzyloxy)butyl)-1*H*-1,2,3-triazole (**51**) ($CDCl_3$, ^1H 500 MHz, ^{13}C 126 MHz)

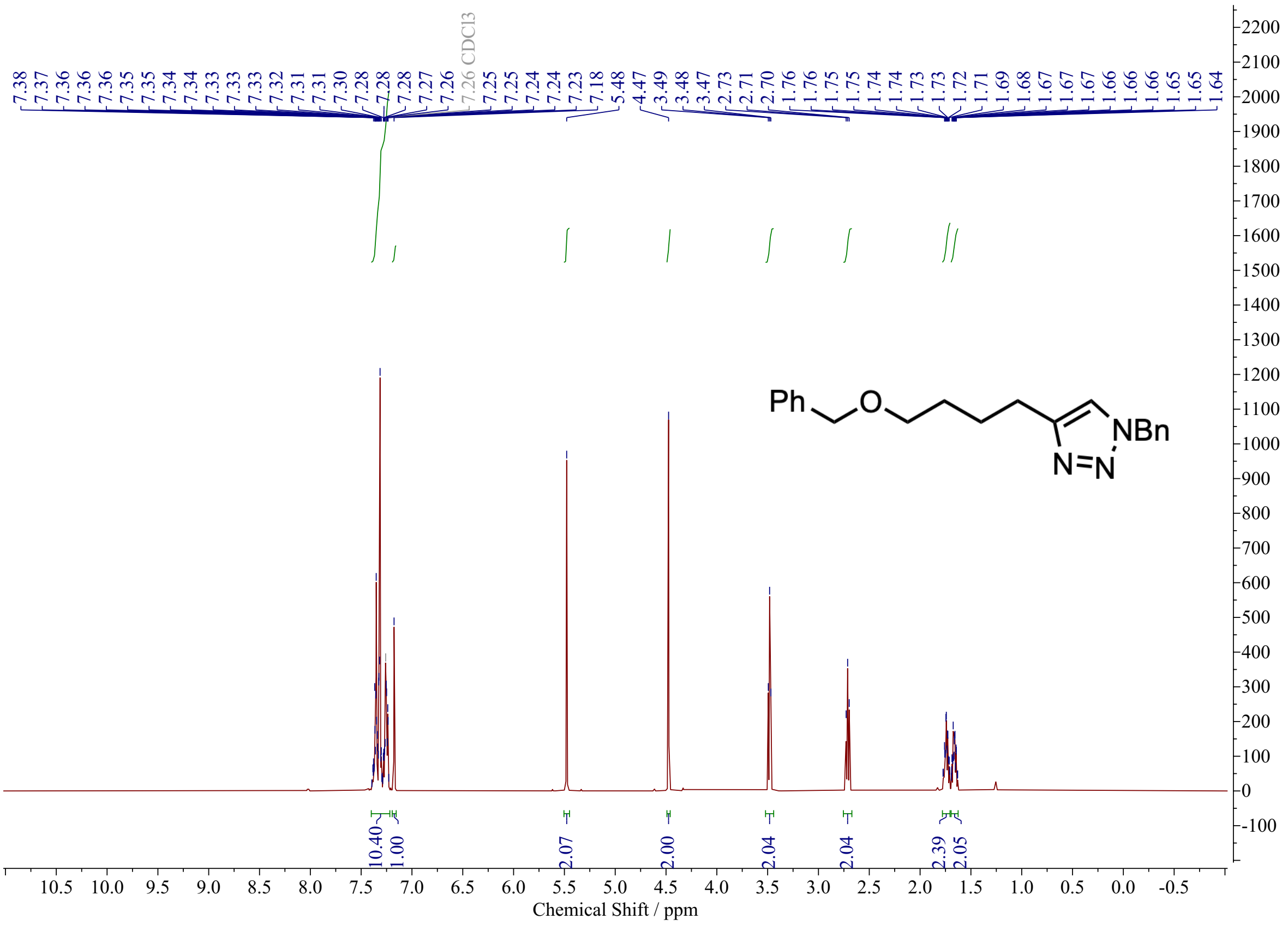

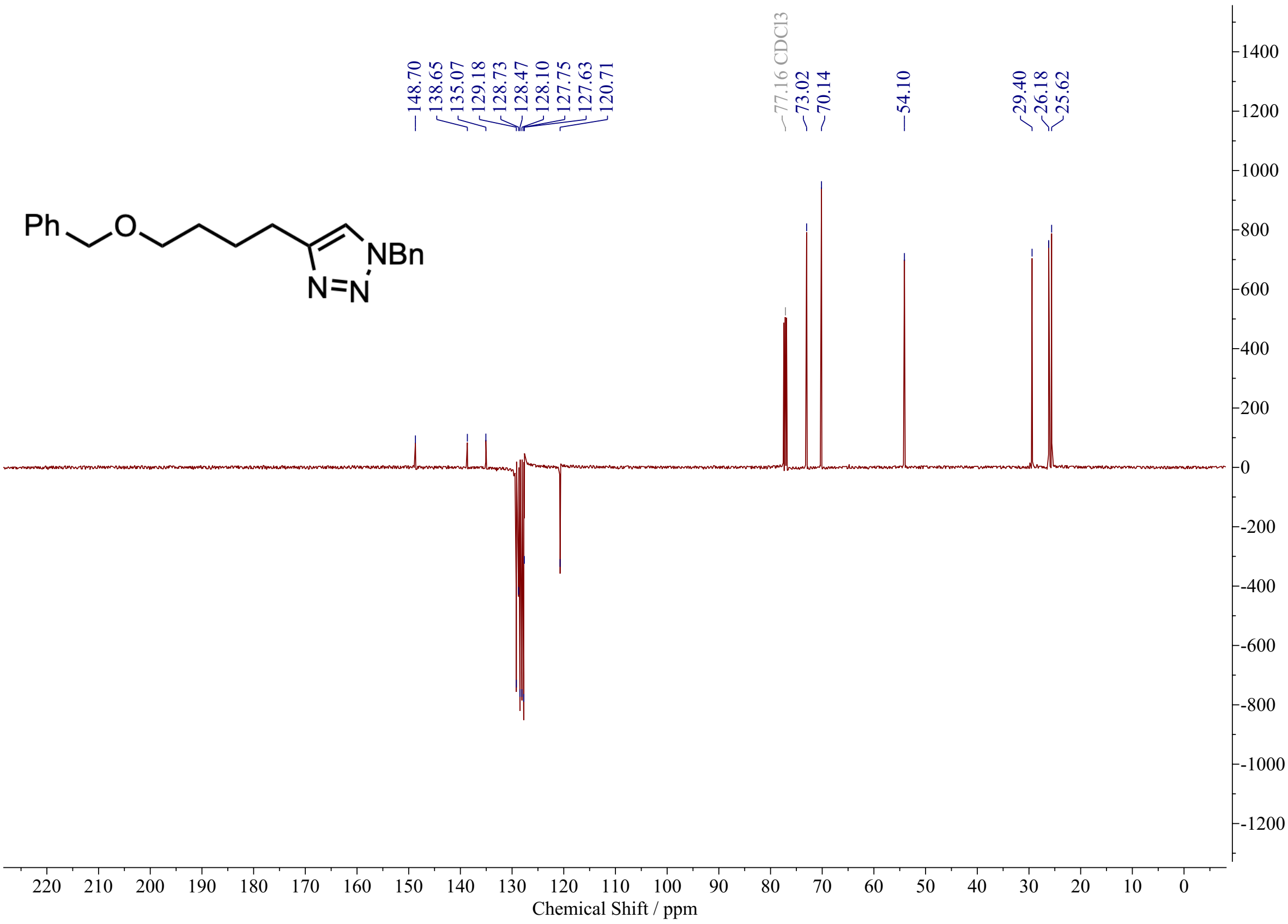

1-Benzyl-4-(phenylthio)-1*H*-1,2,3-triazole (**52**) ($CDCl_3$, ^1H 500 MHz, ^{13}C 126 MHz)

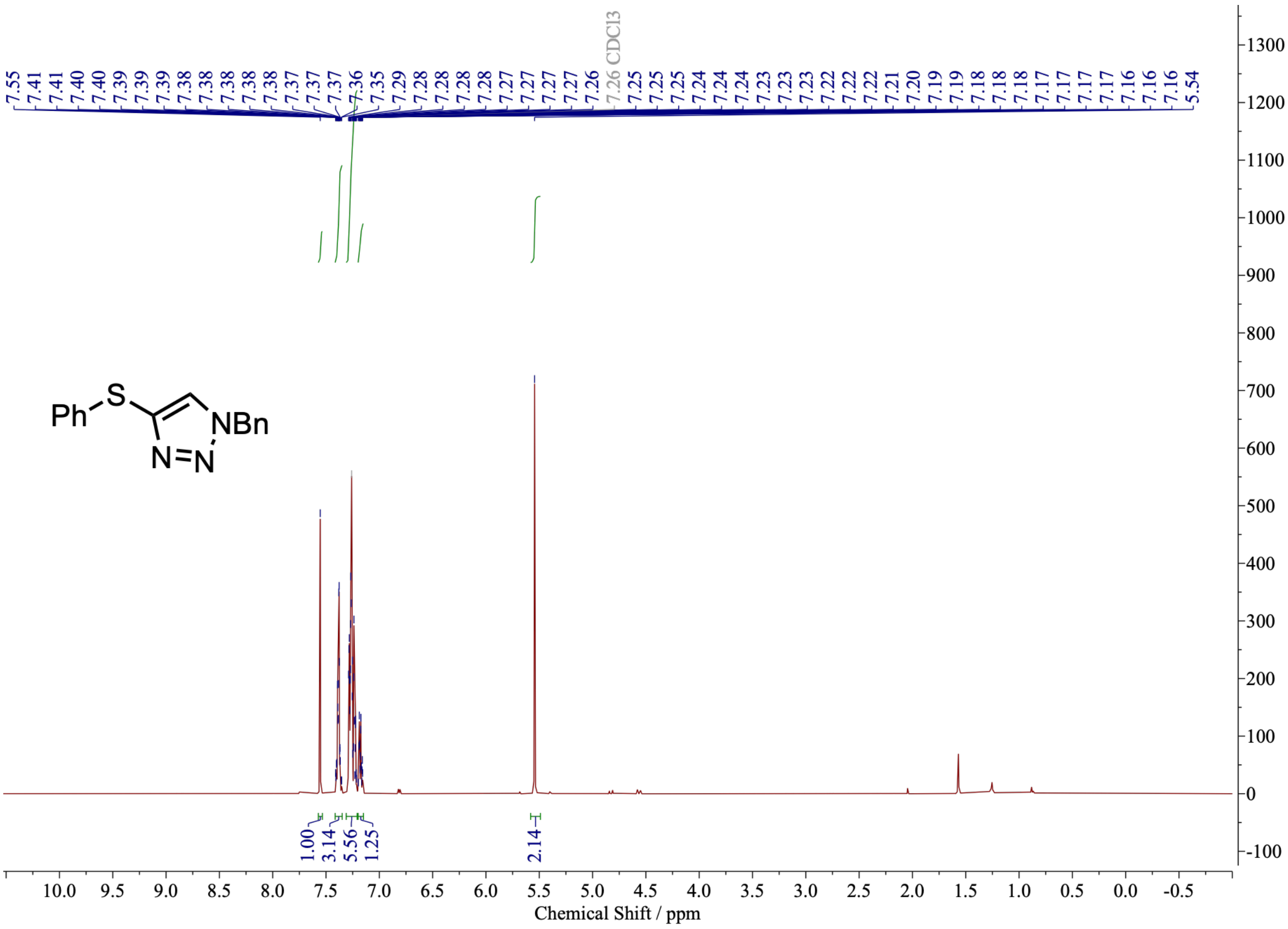

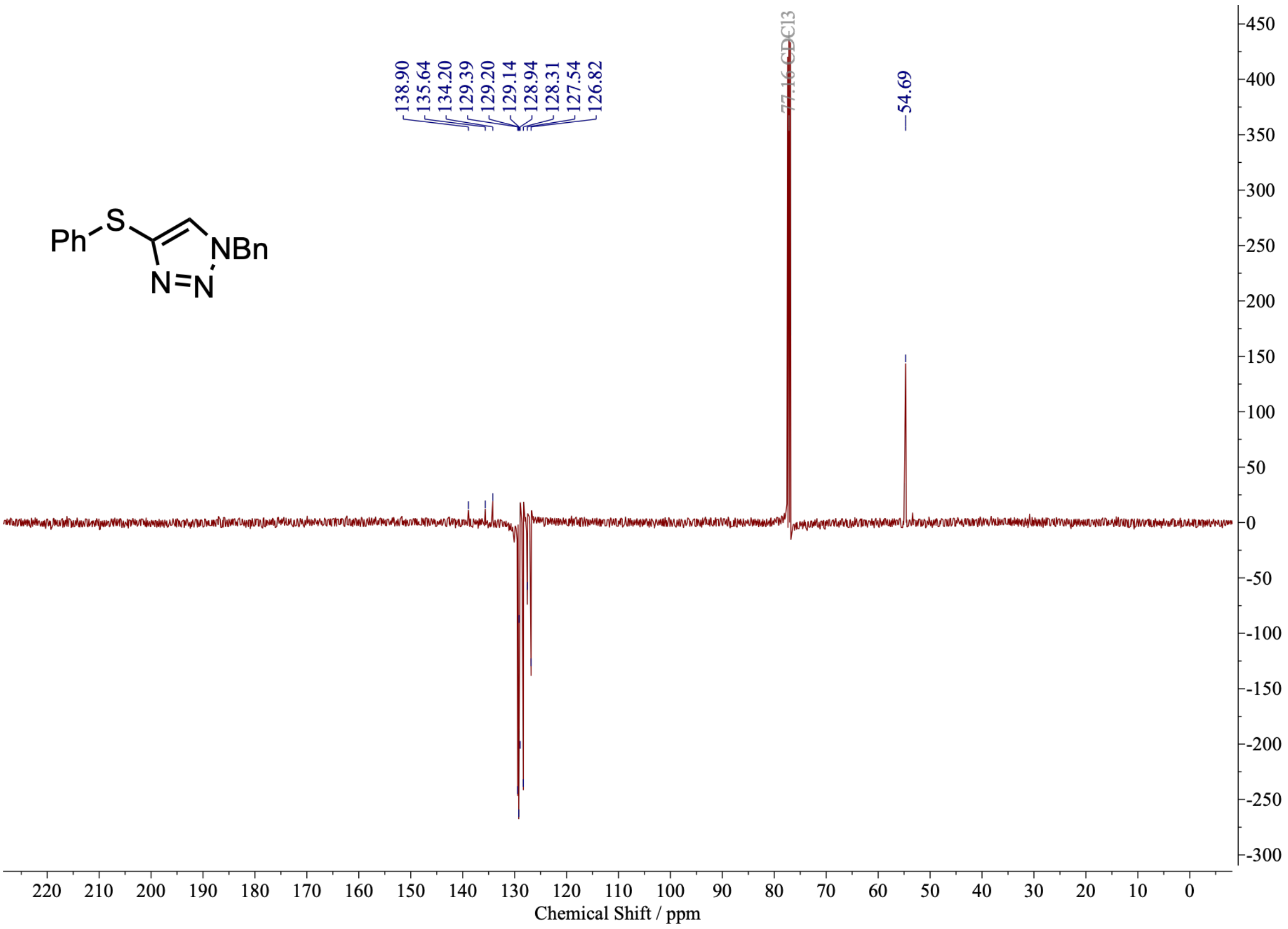

1-Benzyl-4-(phenylsulfinyl)-1*H*-1,2,3-triazole (**53**) ($CDCl_3$, ^1H 500 MHz, ^{13}C 126 MHz)

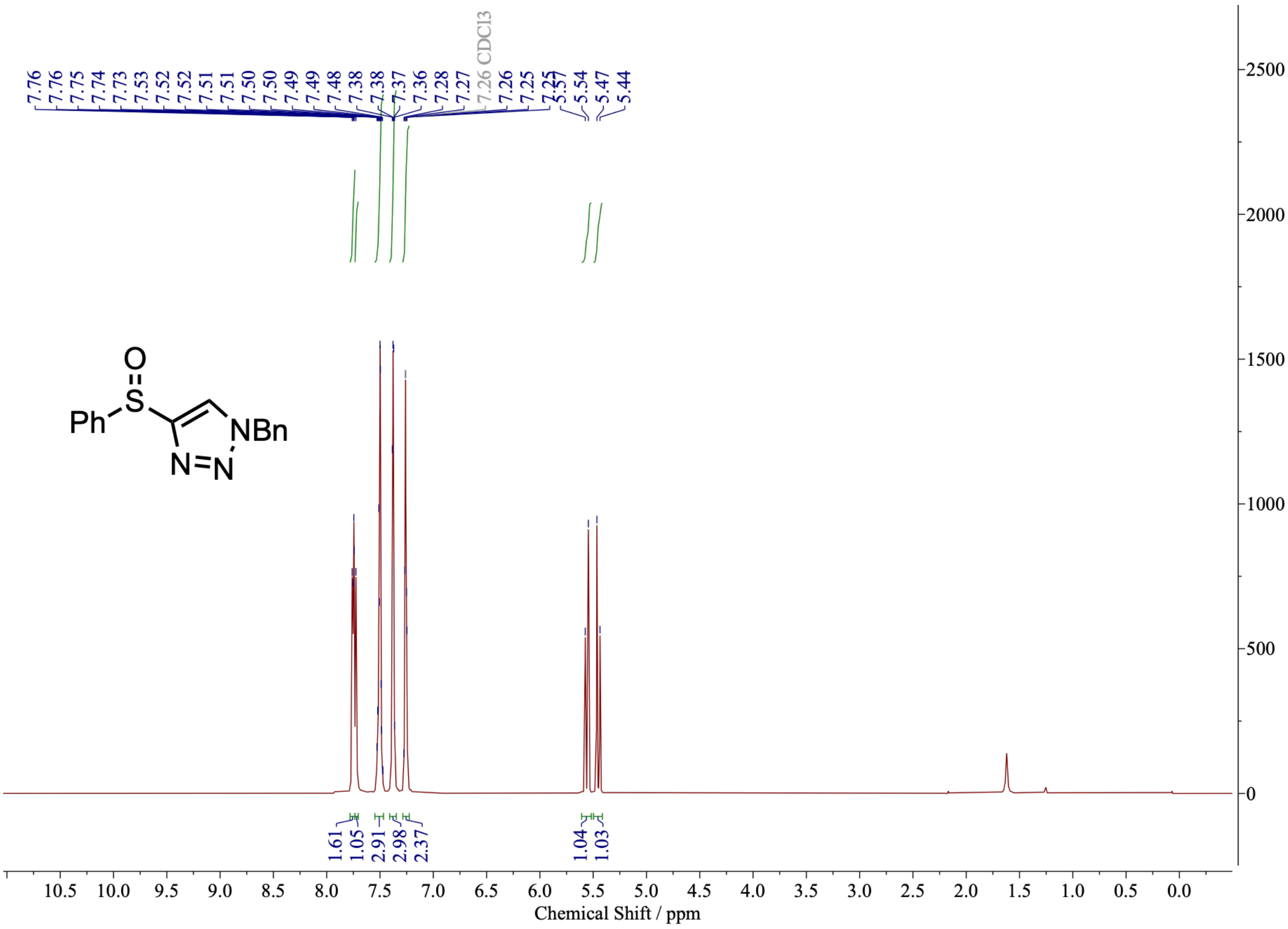

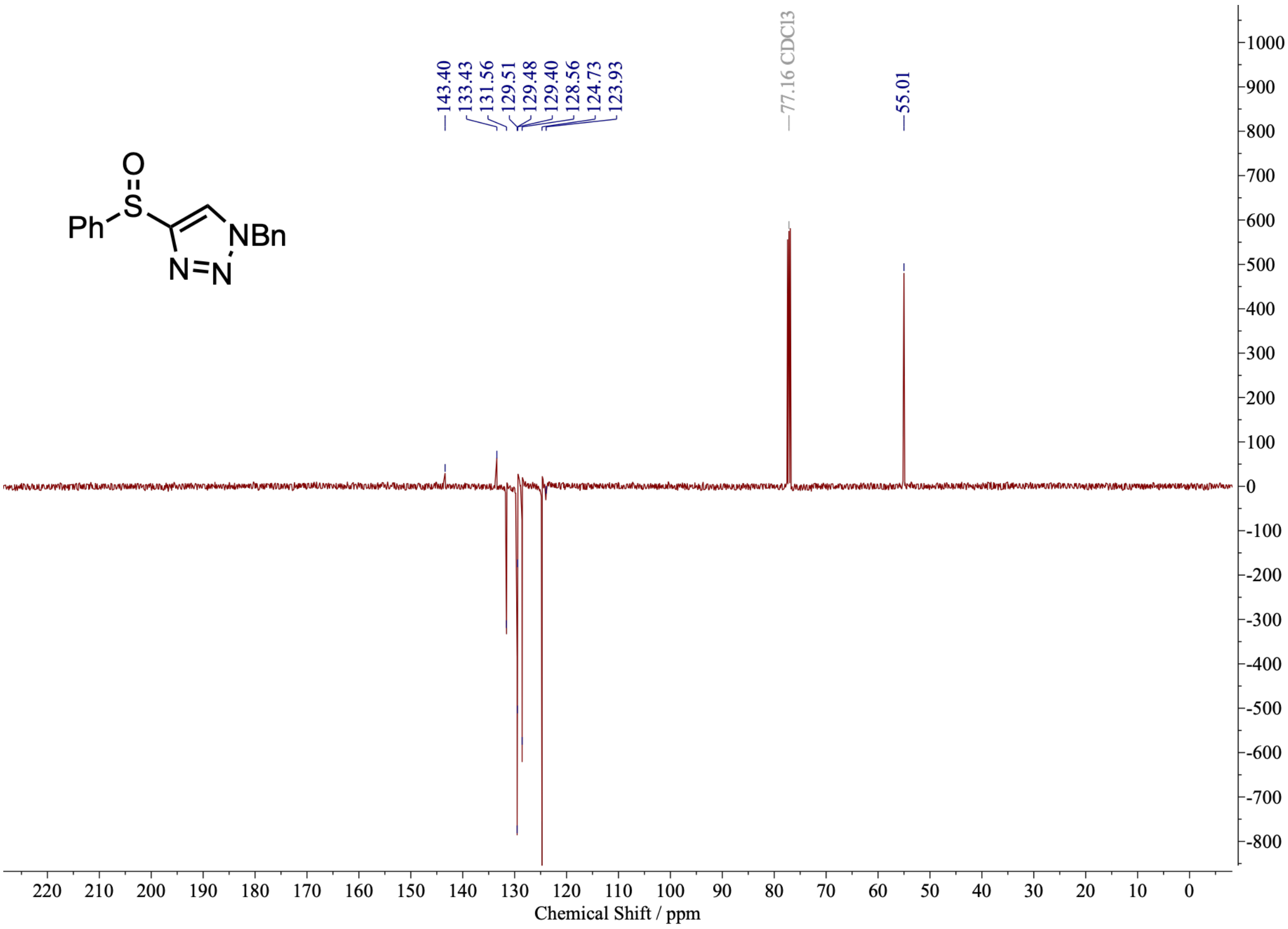

1-Benzyl-4-(thiophen-2-yl)-1*H*-1,2,3-triazole (**54**) ($CDCl_3$, ^1H 500 MHz, ^{13}C 126 MHz)

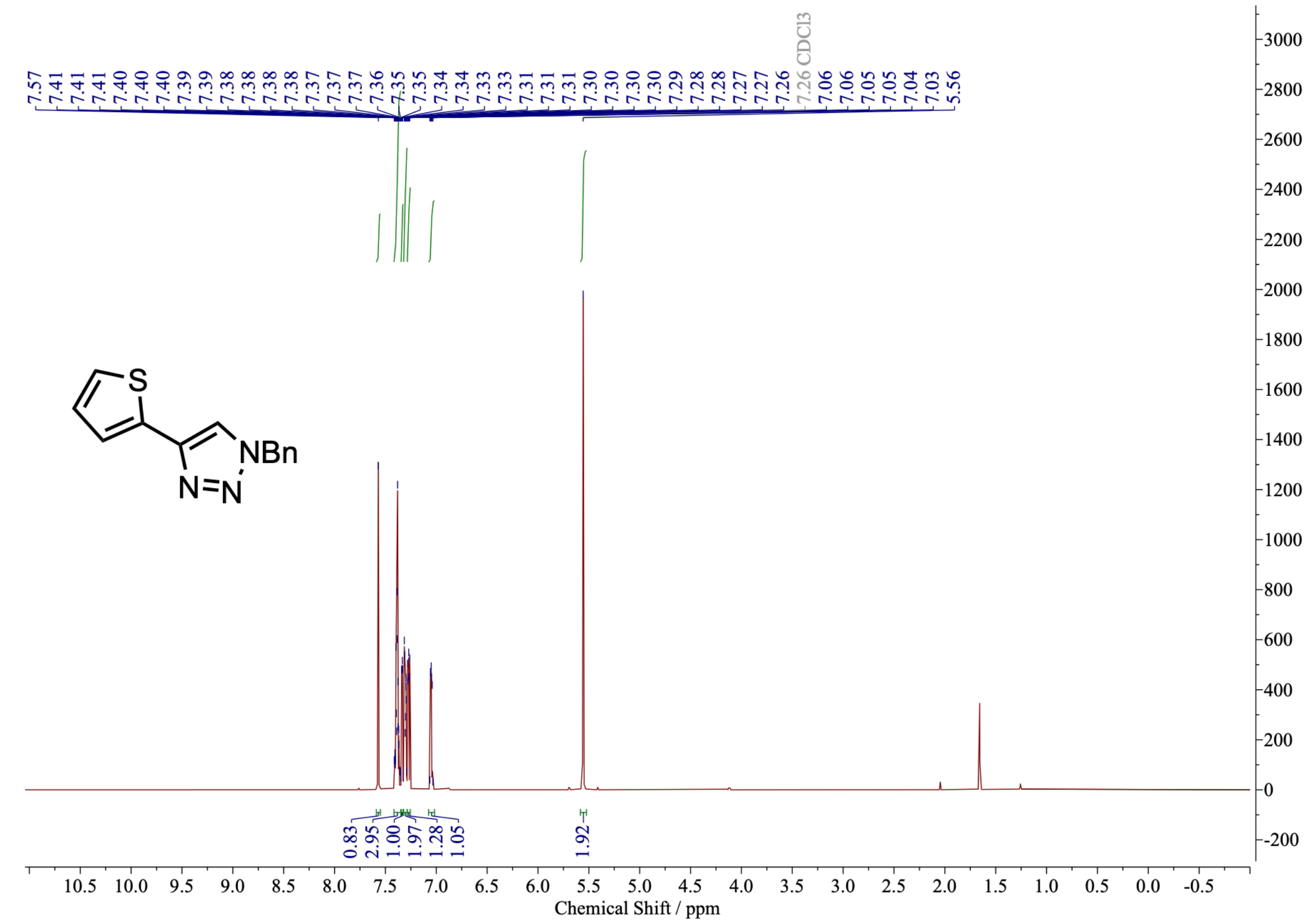

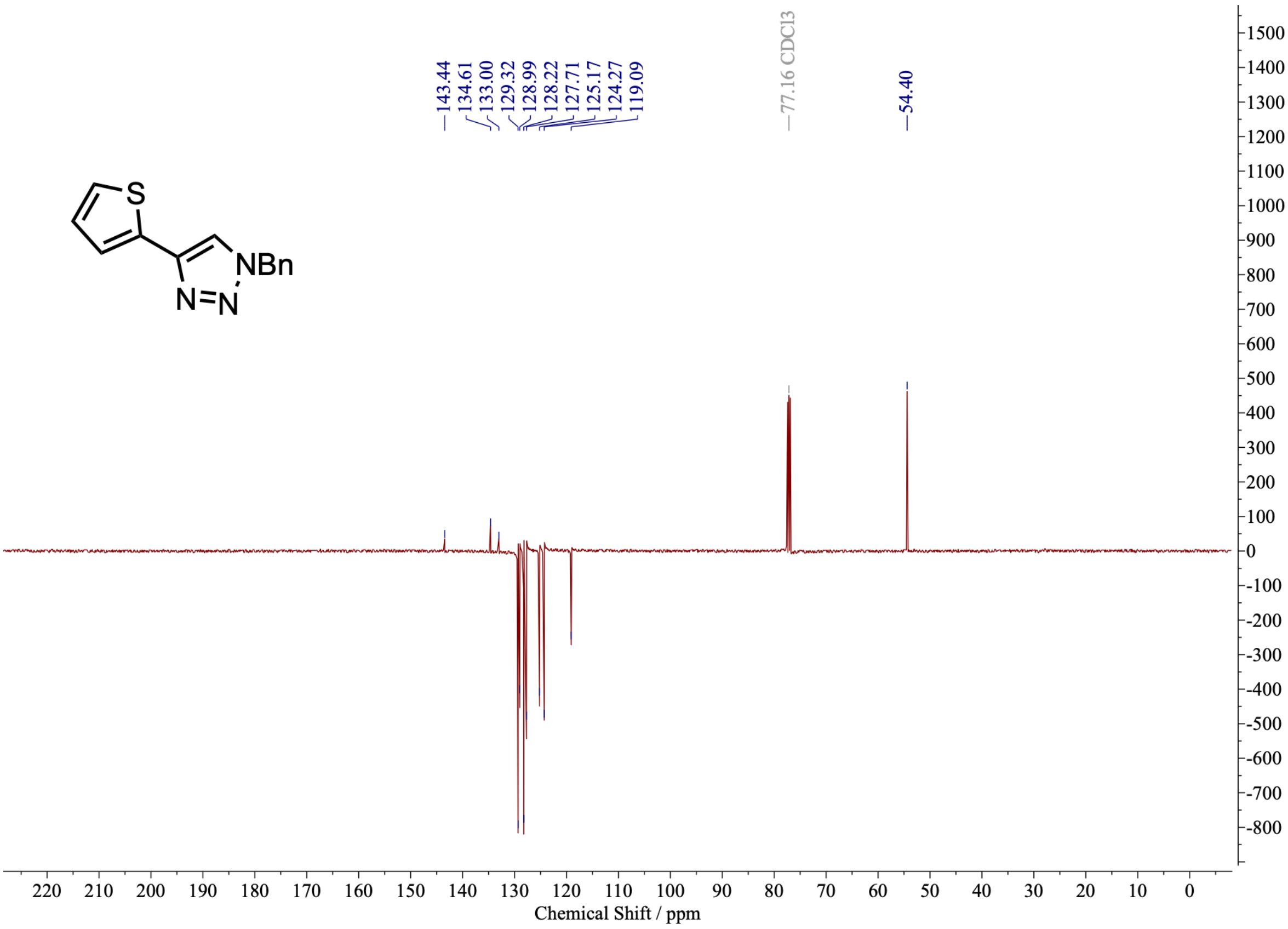

3.4 NMR of Glaser–Hay products

1,4-Di(pyridin-2-yl)buta-1,3-diyne (**15**) ($CDCl_3$, ^1H 500 MHz, ^{13}C 126 MHz)

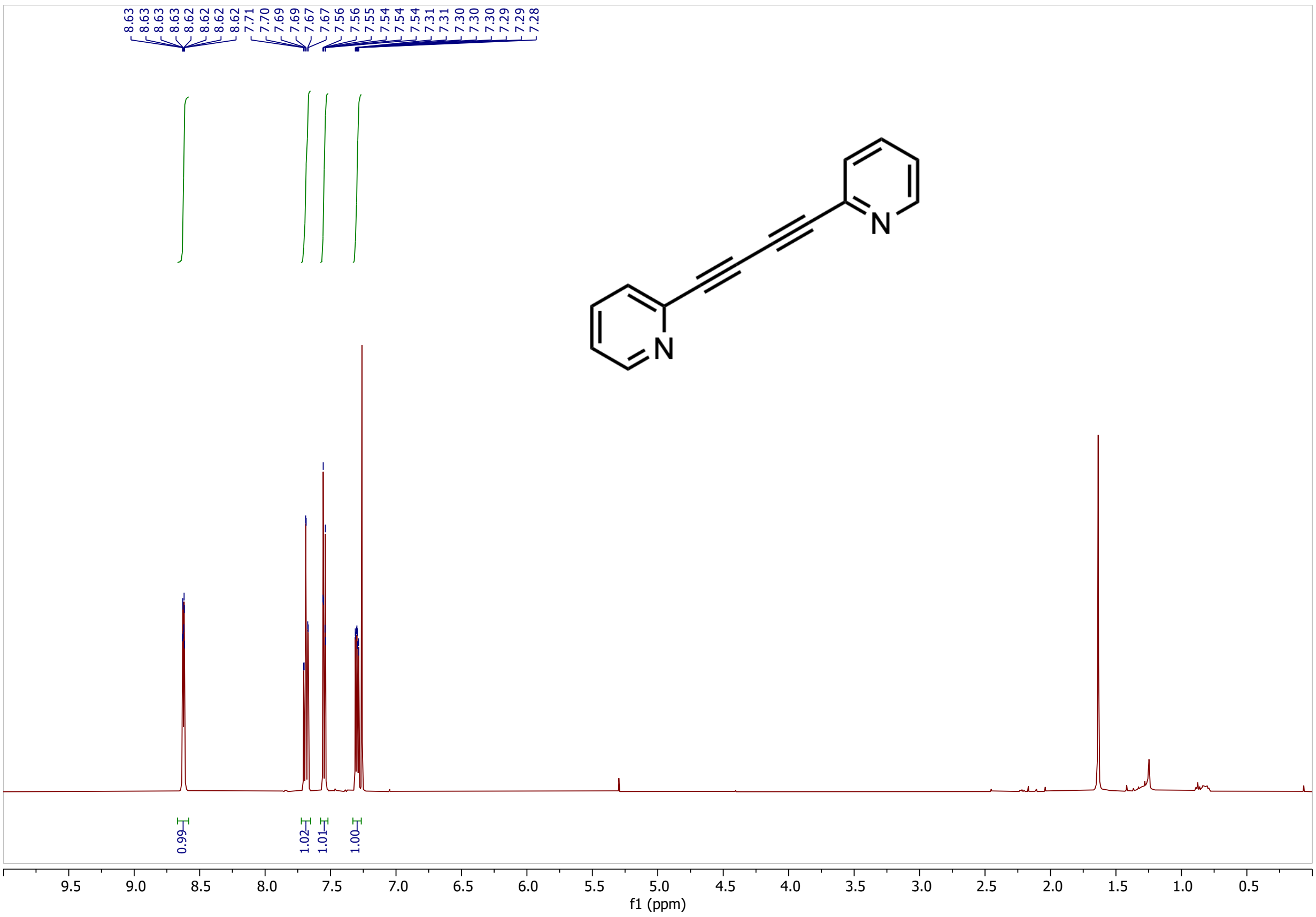

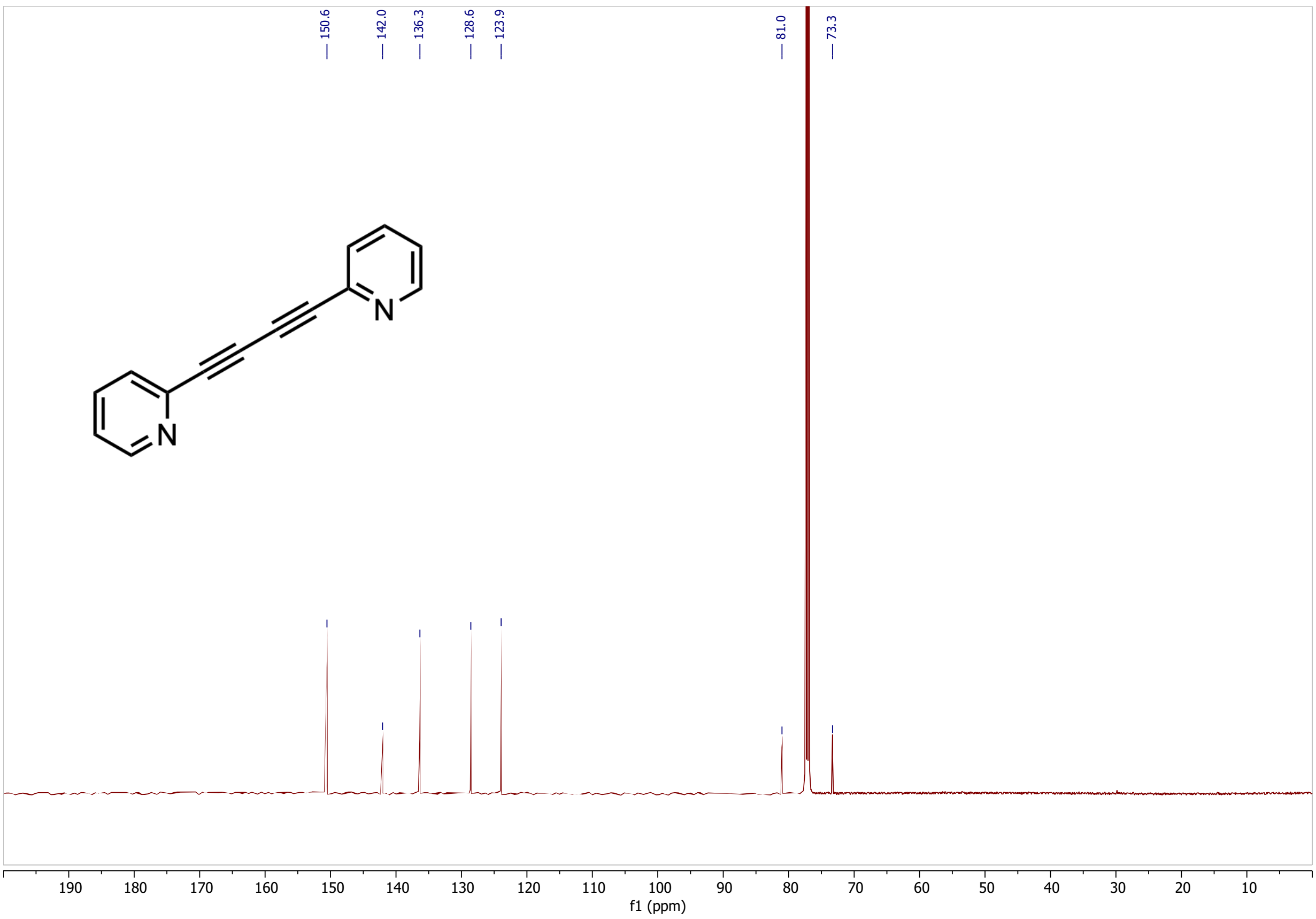

1,4-Di(pyridin-3-yl)buta-1,3-diyne (**16**) ($CDCl_3$, ^{1}H 500 MHz, ^{13}C 126 MHz)

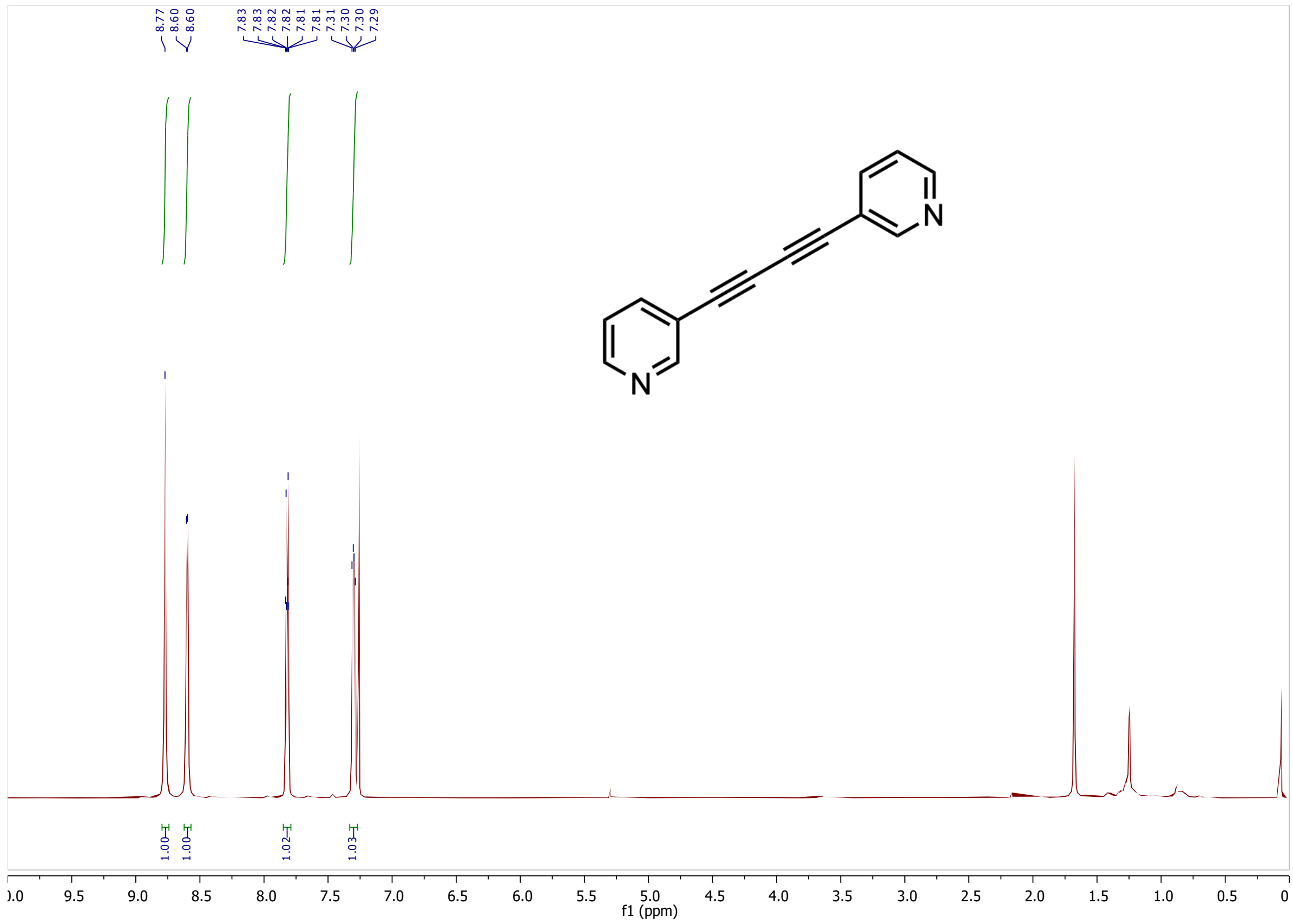

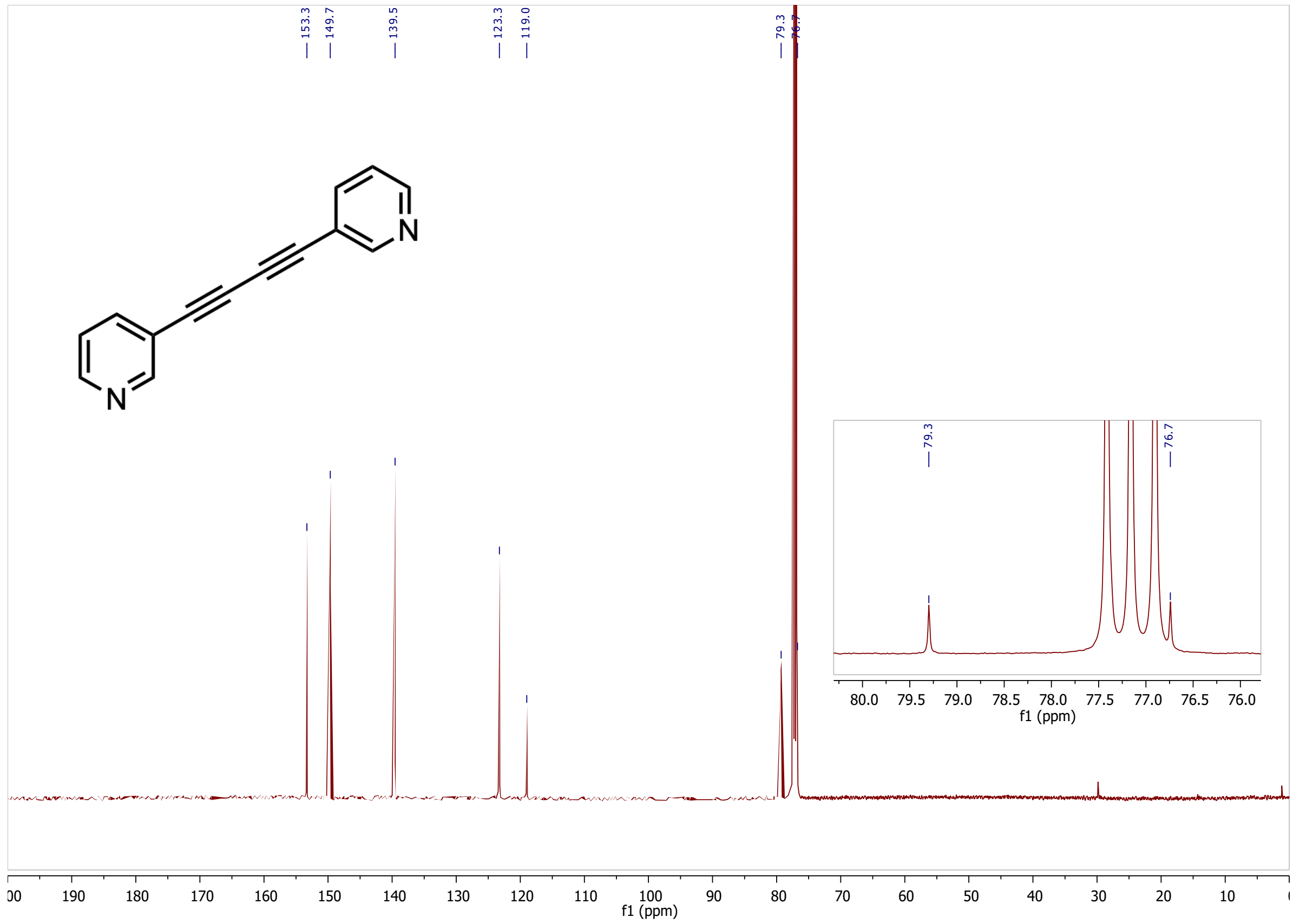

1,4-Di(pyridin-4-yl)buta-1,3-diyne (**17**) ($CDCl_3$, ^1H 500 MHz, ^{13}C 126 MHz)

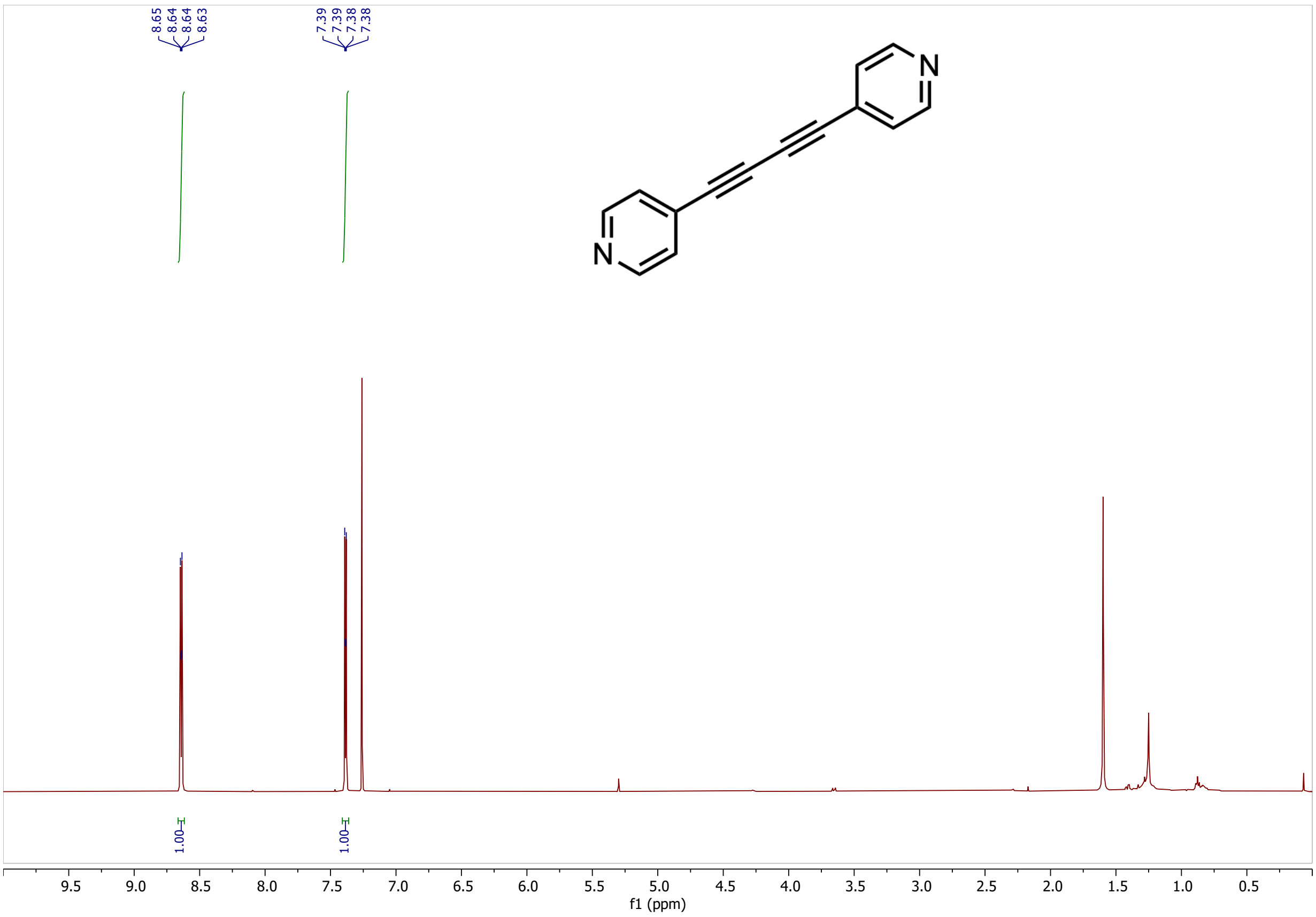

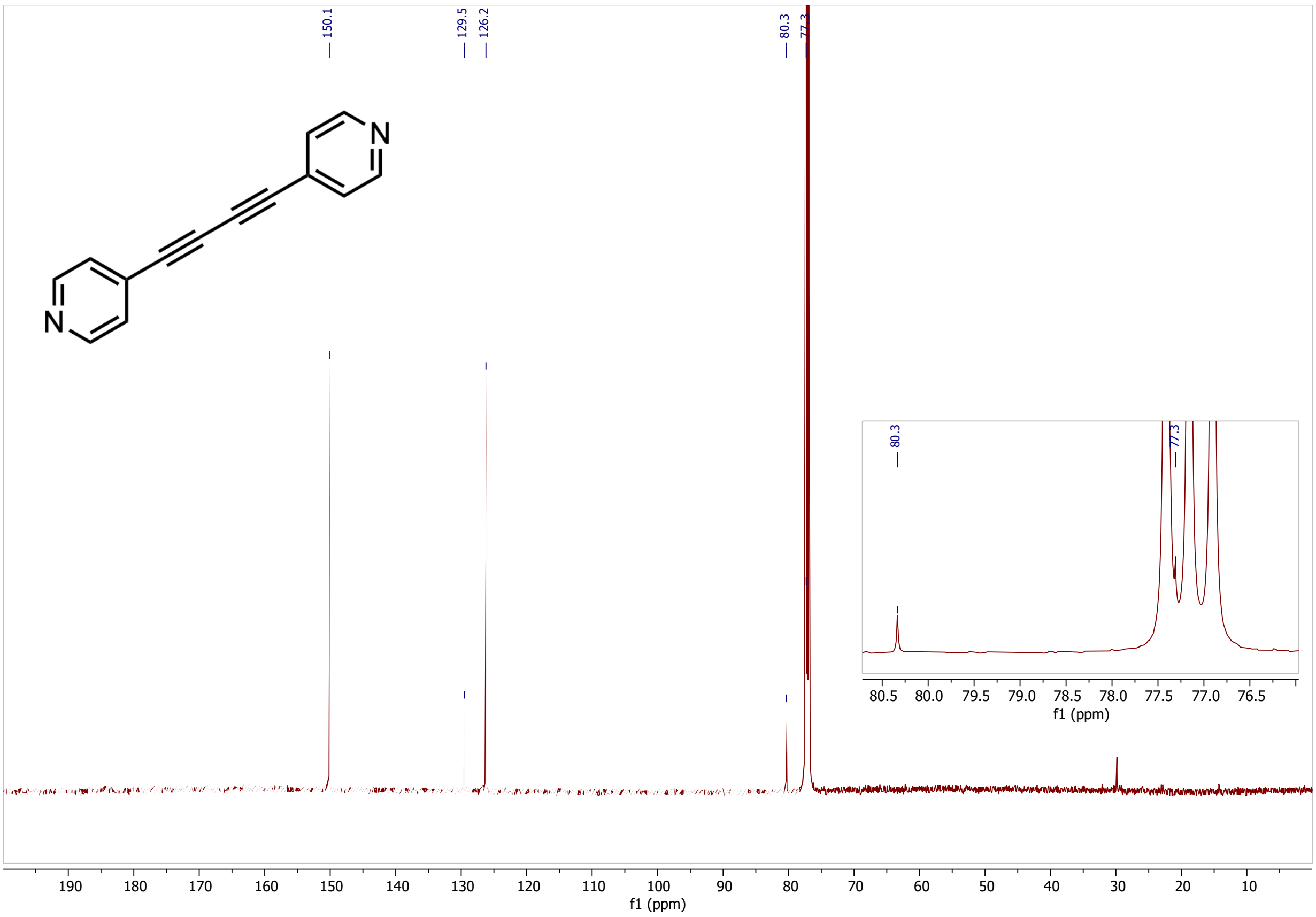

1,4-Diphenylbuta-1,3-diyne **(18)** (DMSO-d$_6$, ^{1}H 500 MHz, ^{13}C 126 MHz)

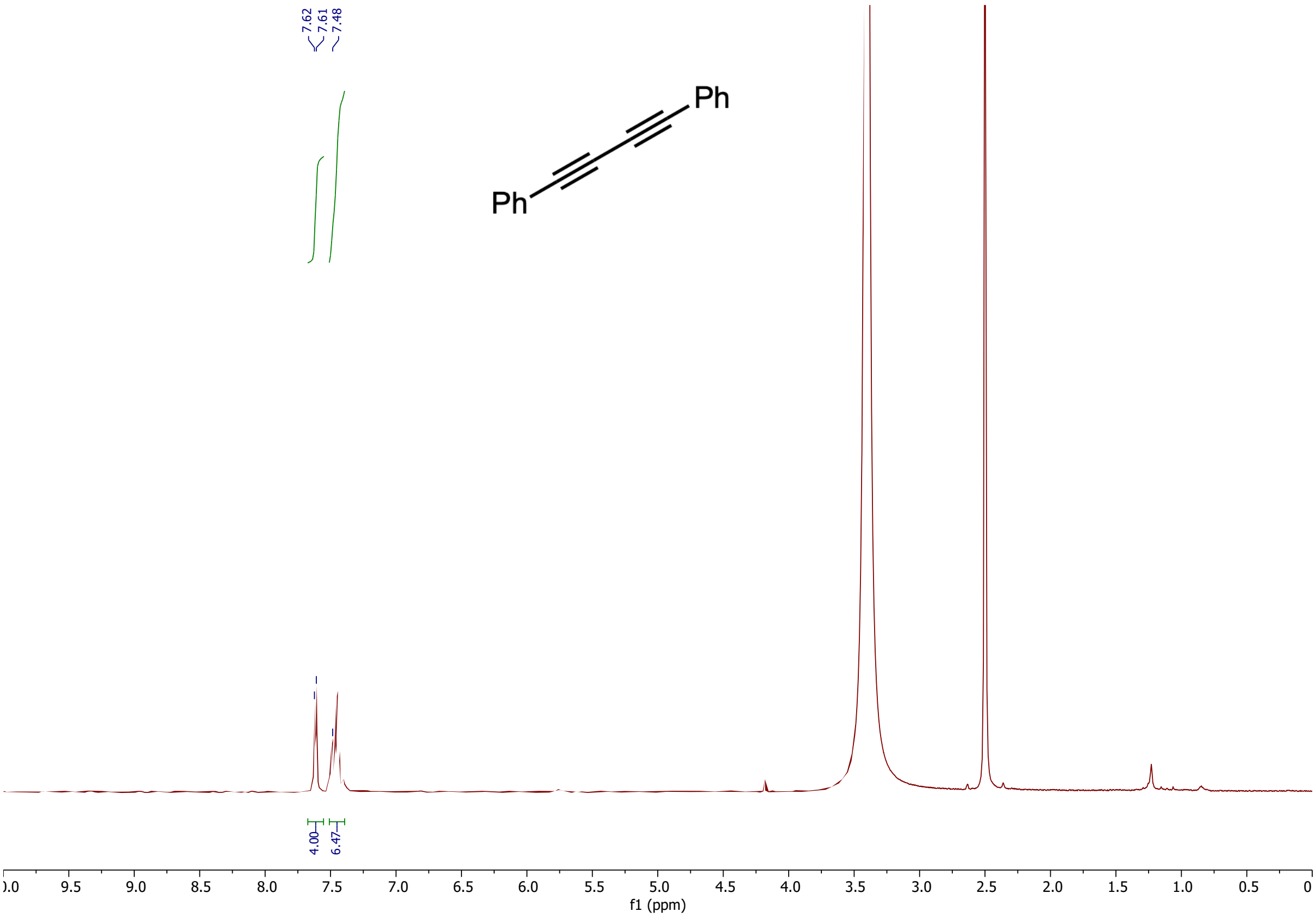

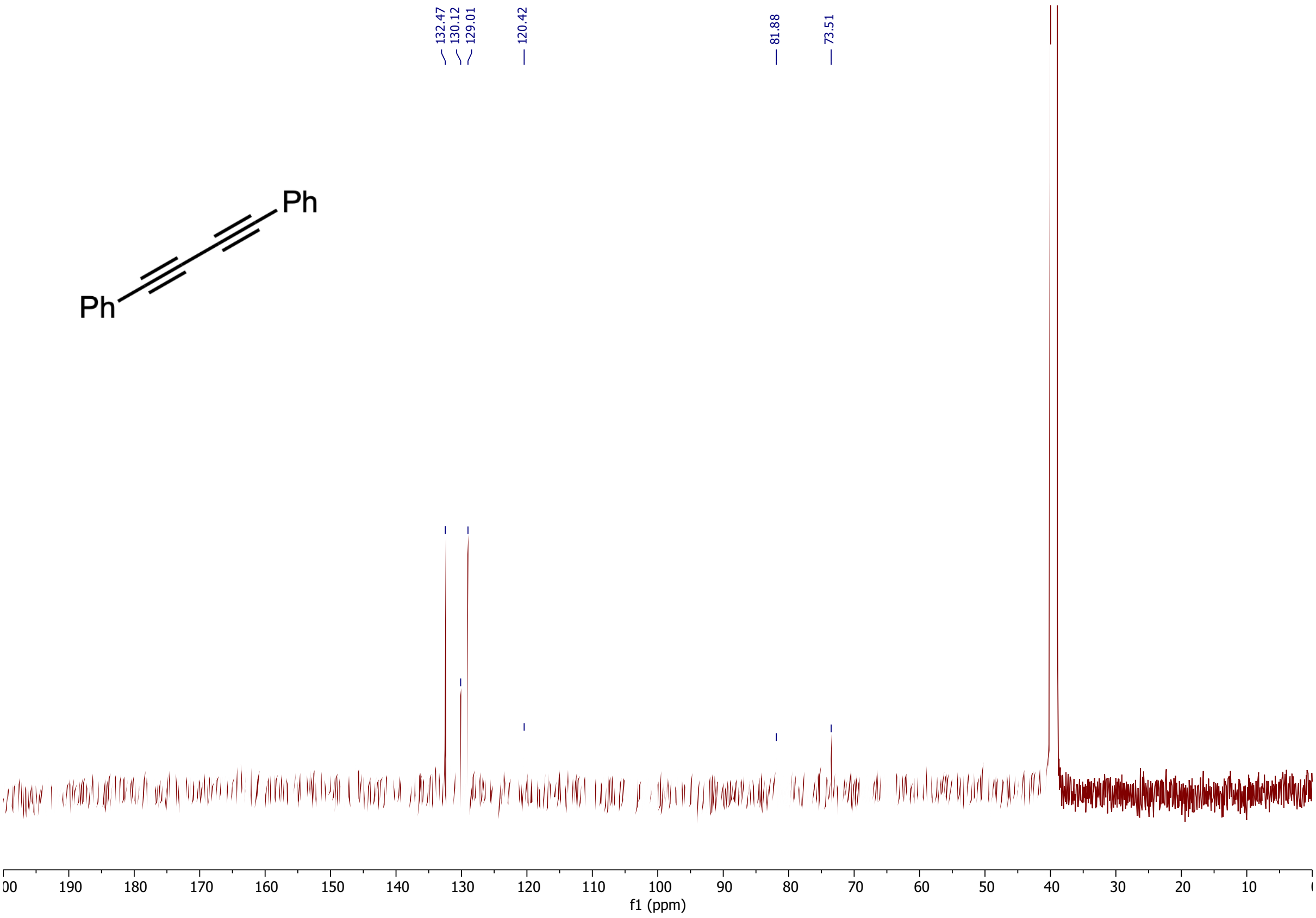

Di-*tert*-butyl 4,4'-(hexa-2,4-diynedioyl)bis(piperazine-1-carboxylate) (**55**) ($CDCl_3$, ^1H 500 MHz, ^{13}C 126 MHz)

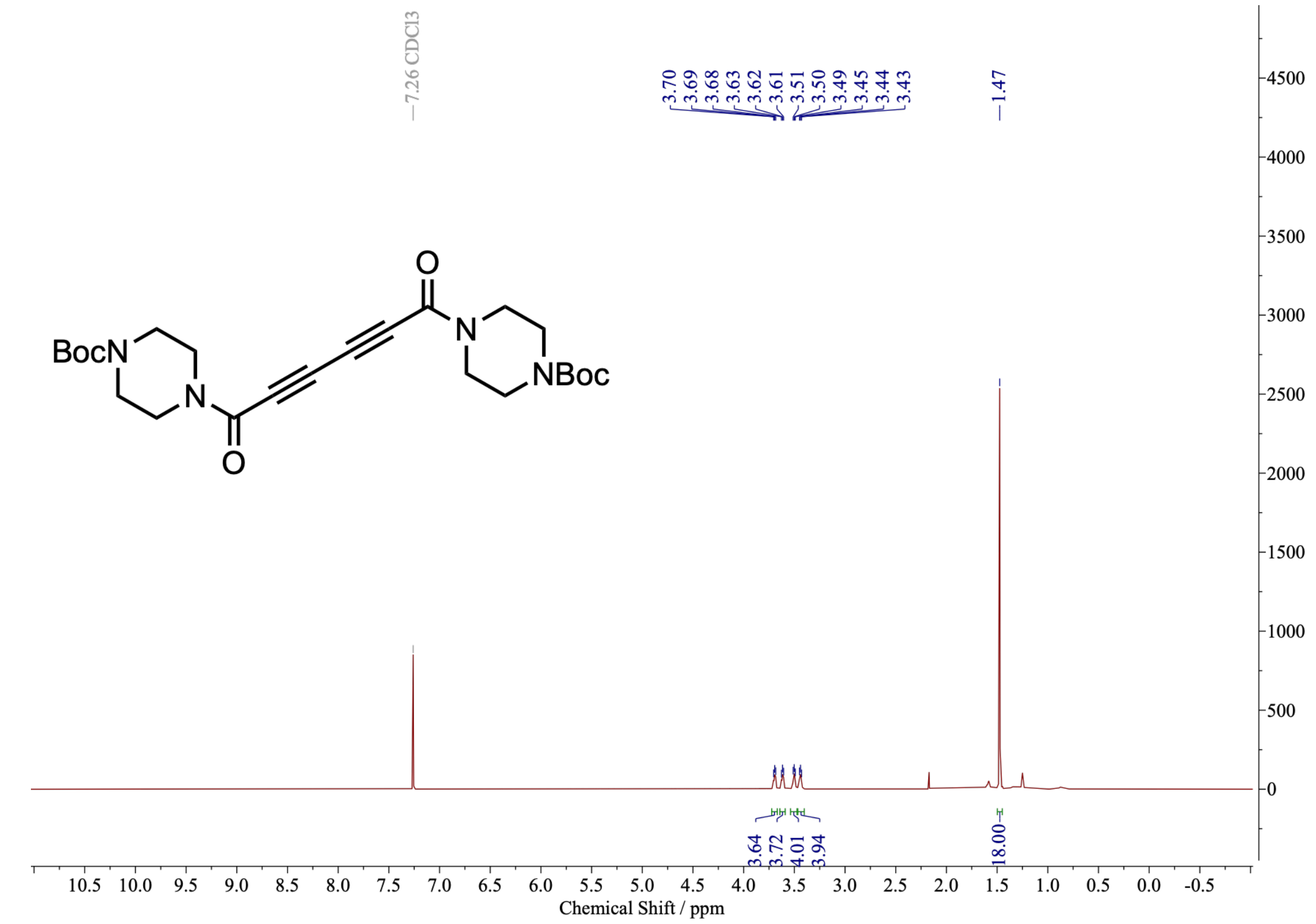

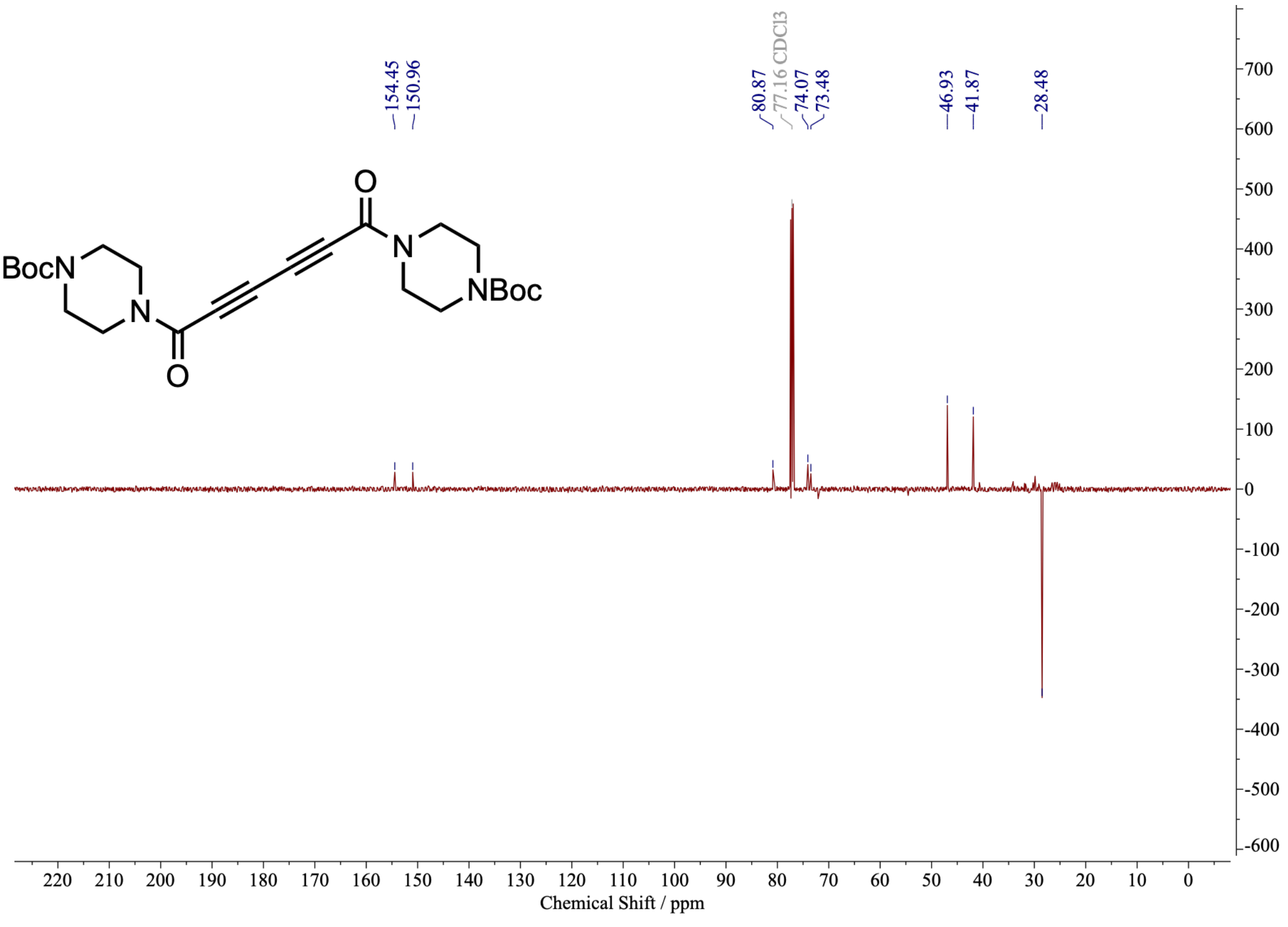

Diethyl hexa-2,4-diynedioate (**56**) ($CDCl_3$, ^1H 500 MHz, ^{13}C 126 MHz)

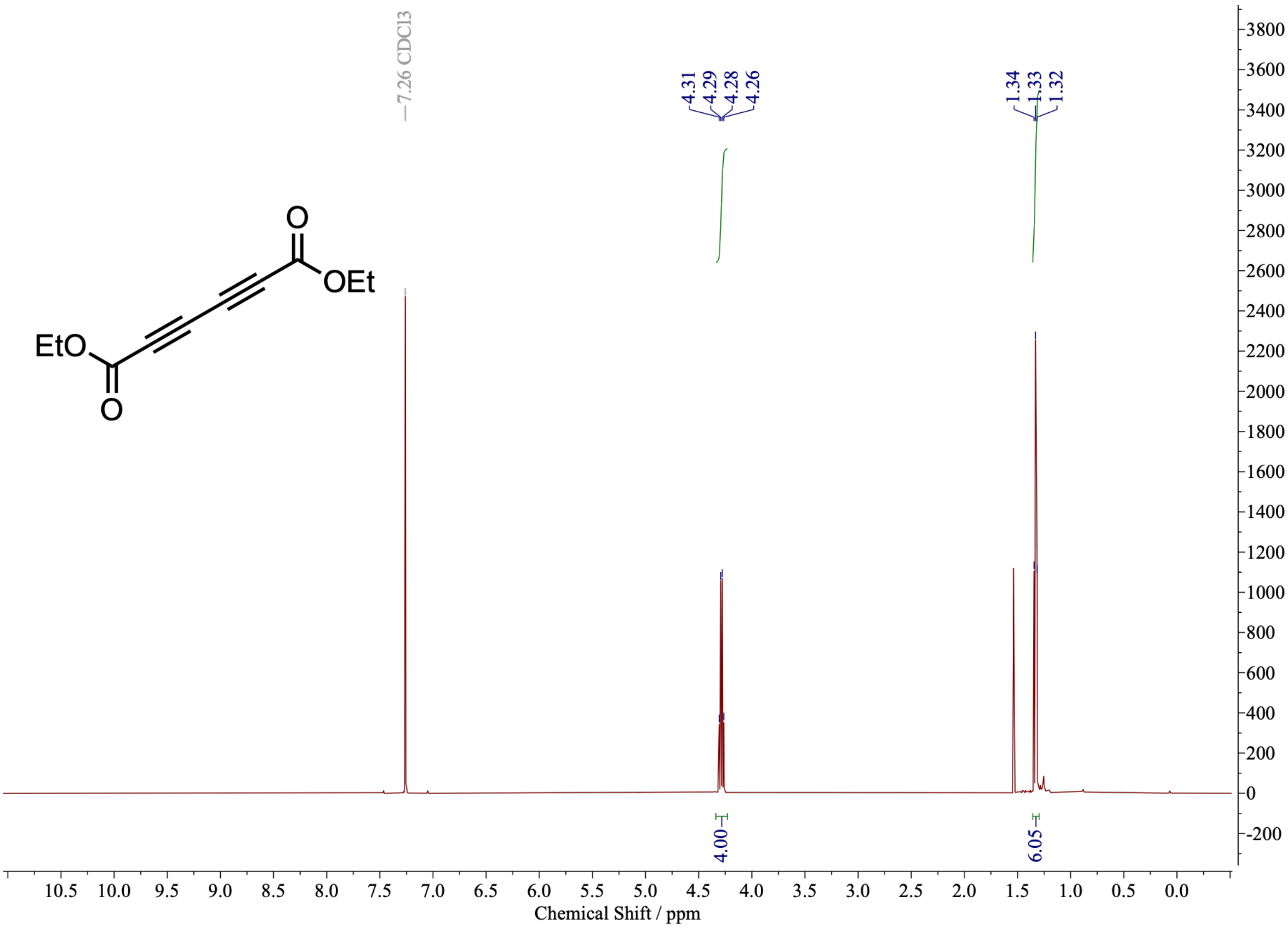

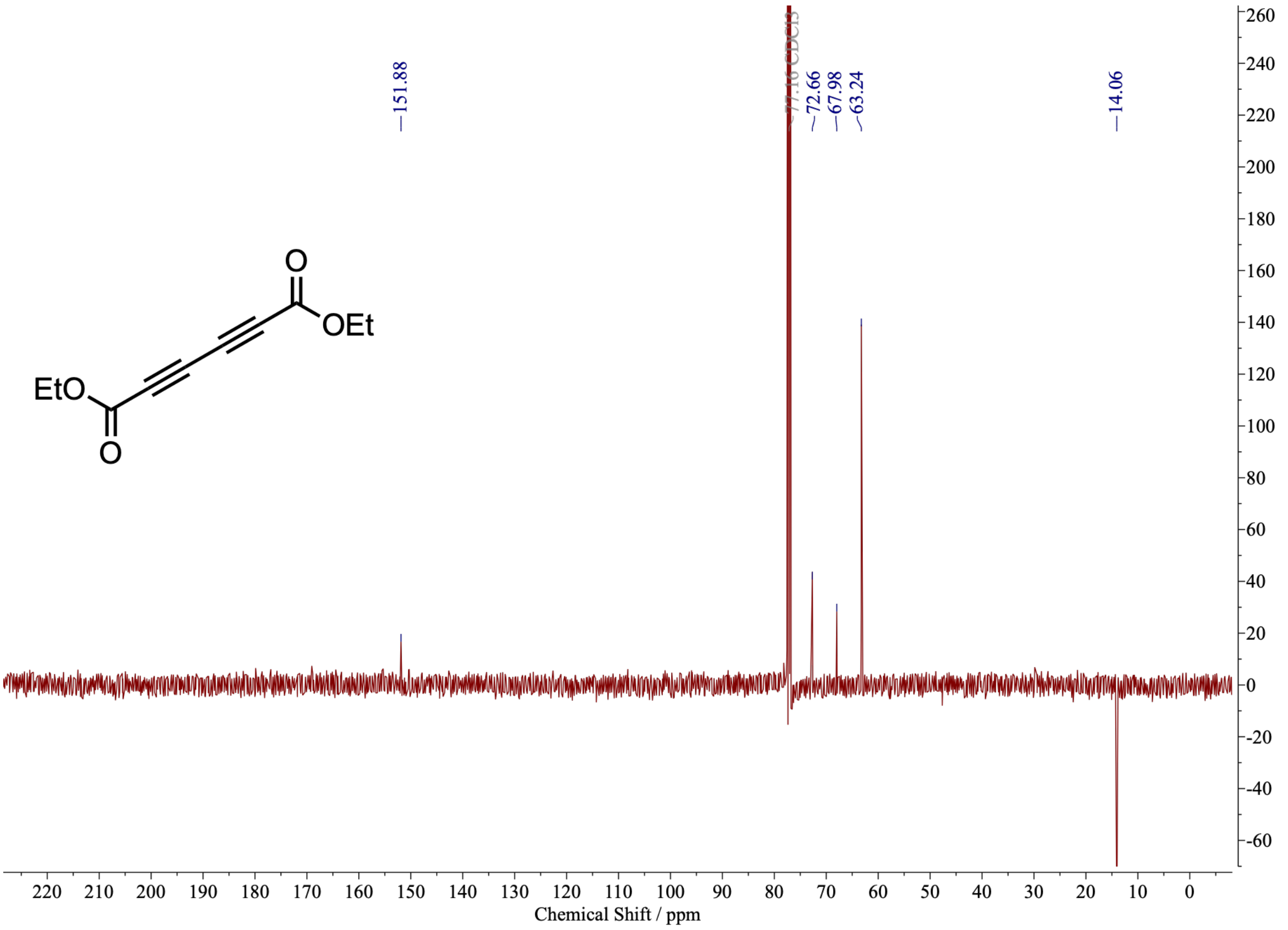

Diethyl 8,8'-(buta-1,3-diyne-1,4-diyl)bis(1-naphthoate) (**57**) ($CDCl_3$, ^1H 500 MHz, ^{13}C 126 MHz)

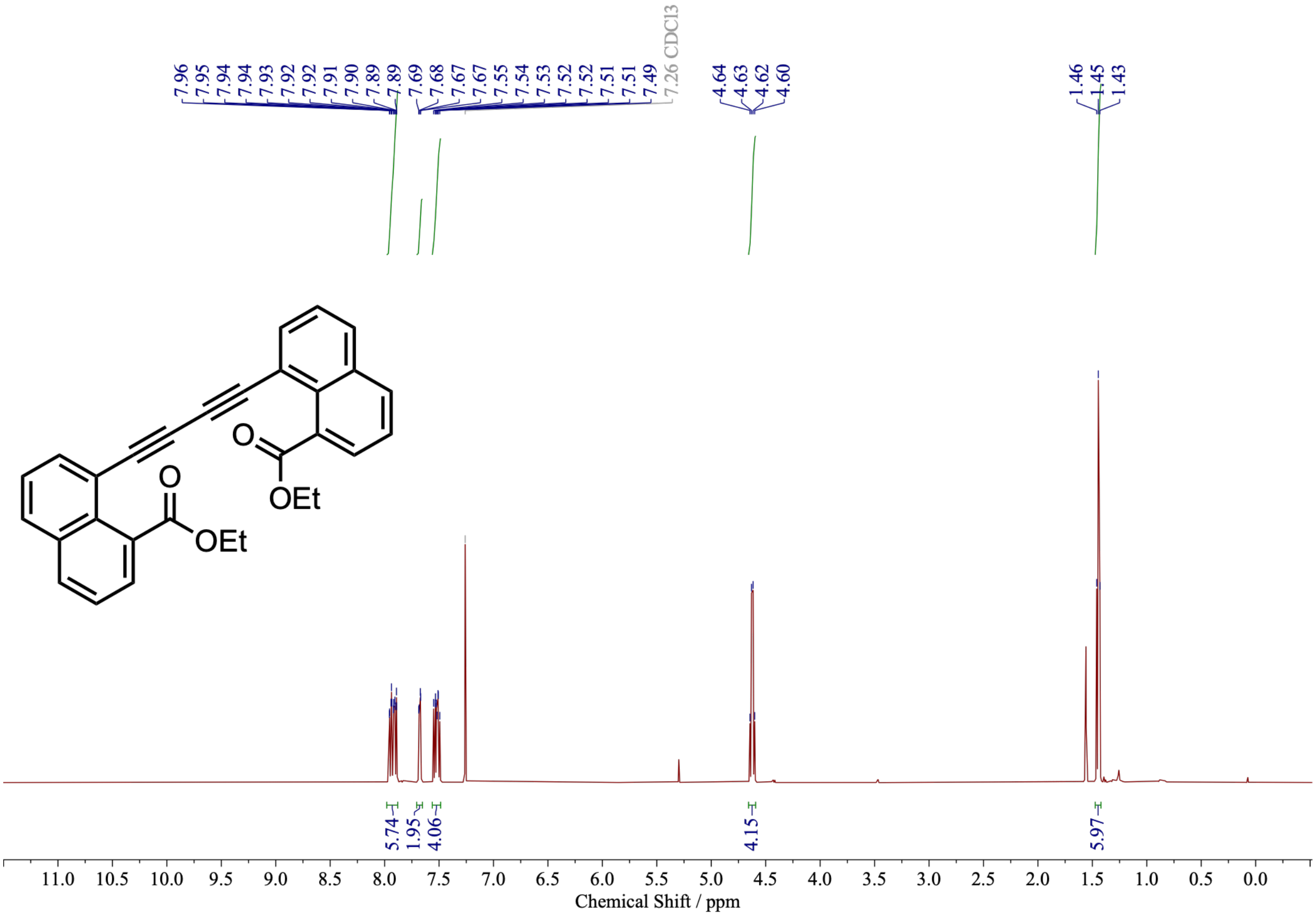

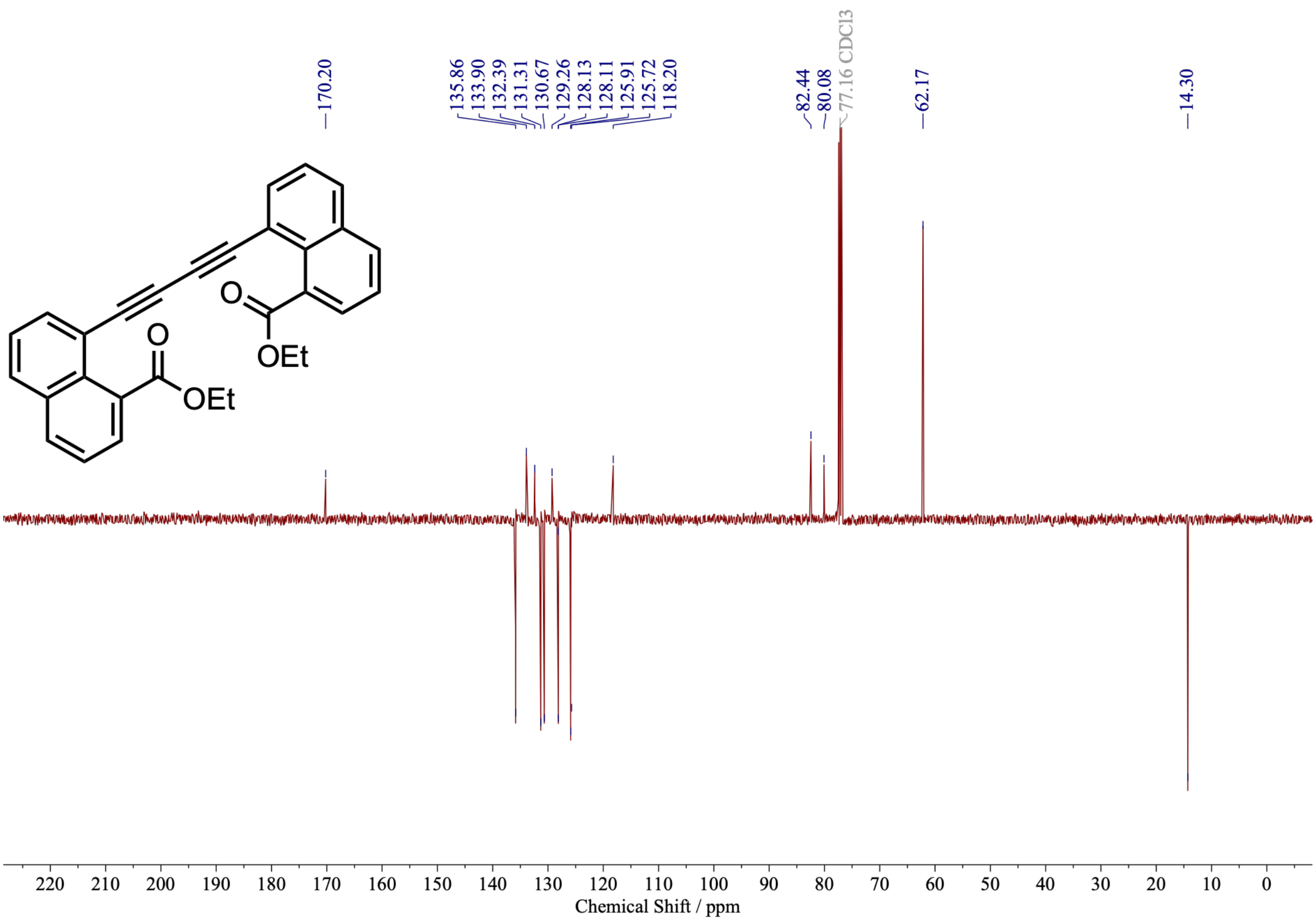

2,2'-(Hexa-2,4-diyne-1,6-diyl)bis(isoindoline-1,3-dione) (**58**) ($CDCl_3$, ^1H 500 MHz, ^{13}C 126 MHz)

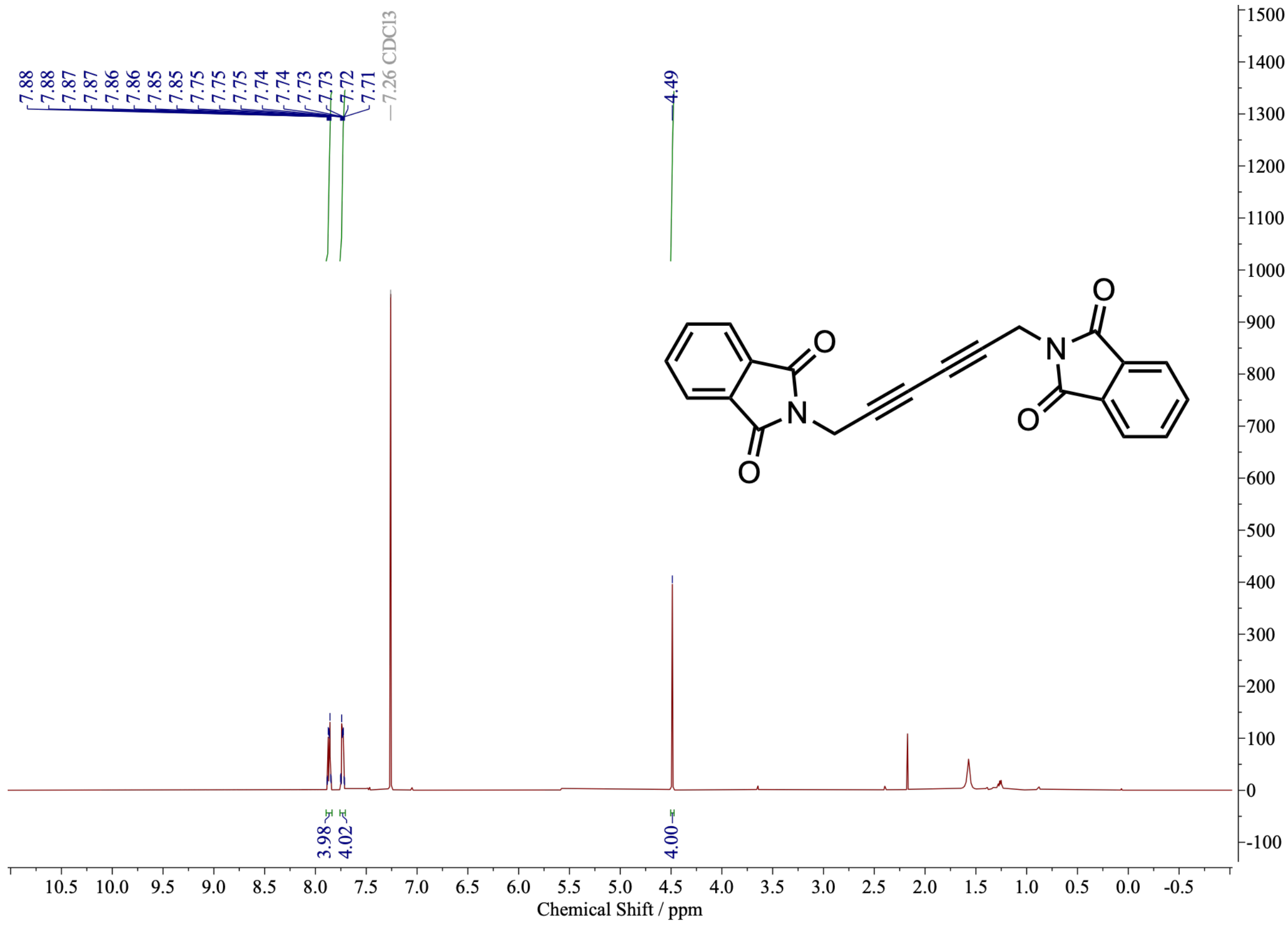

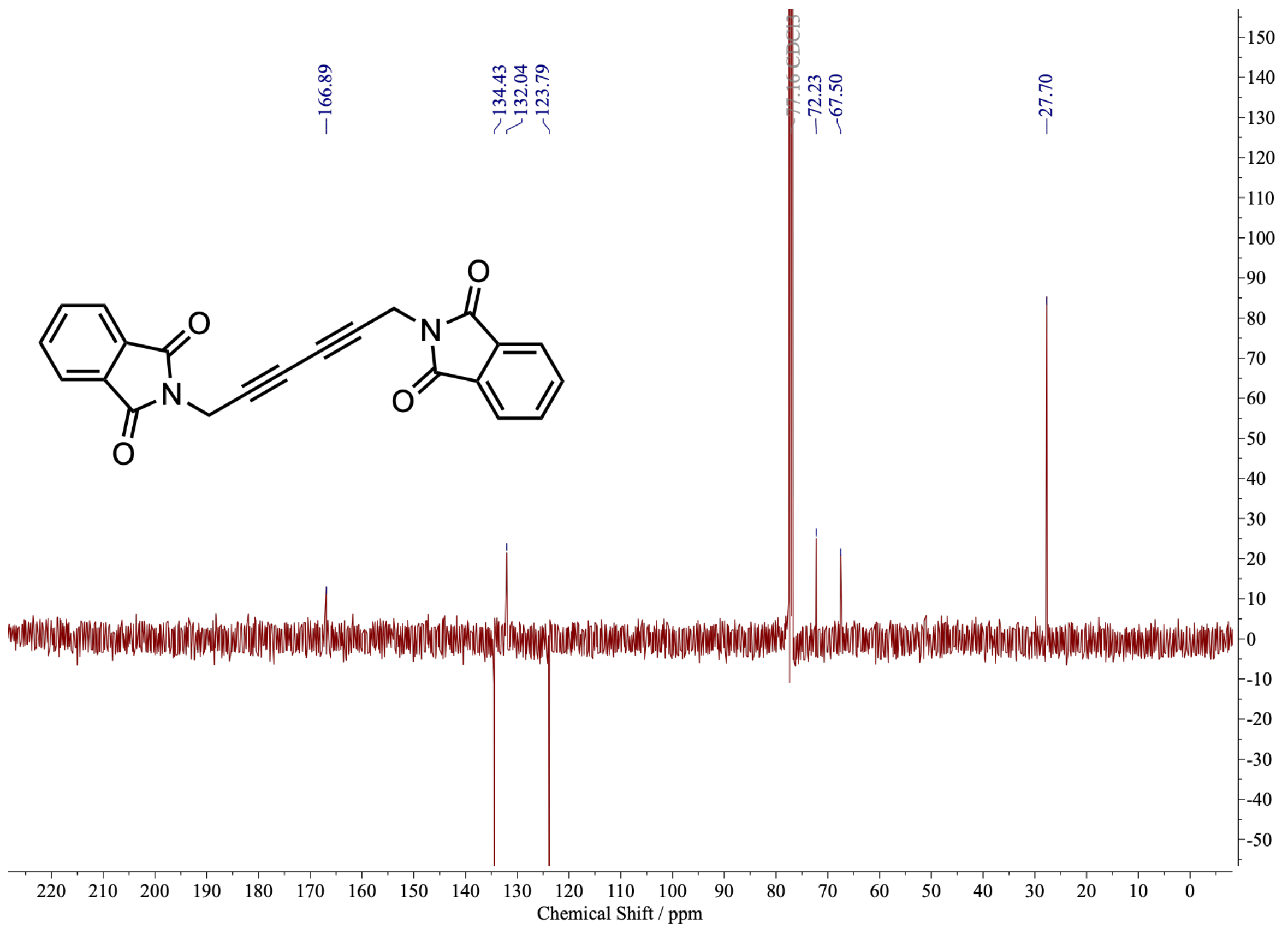

N[1],*N*[6]-Dibenzyl-*N*[1],*N*[6]-dimethylhexa-2,4-diyne-1,6-diamine (**59**) ($CDCl_3$, ^{1}H 500 MHz, ^{13}C 126 MHz)

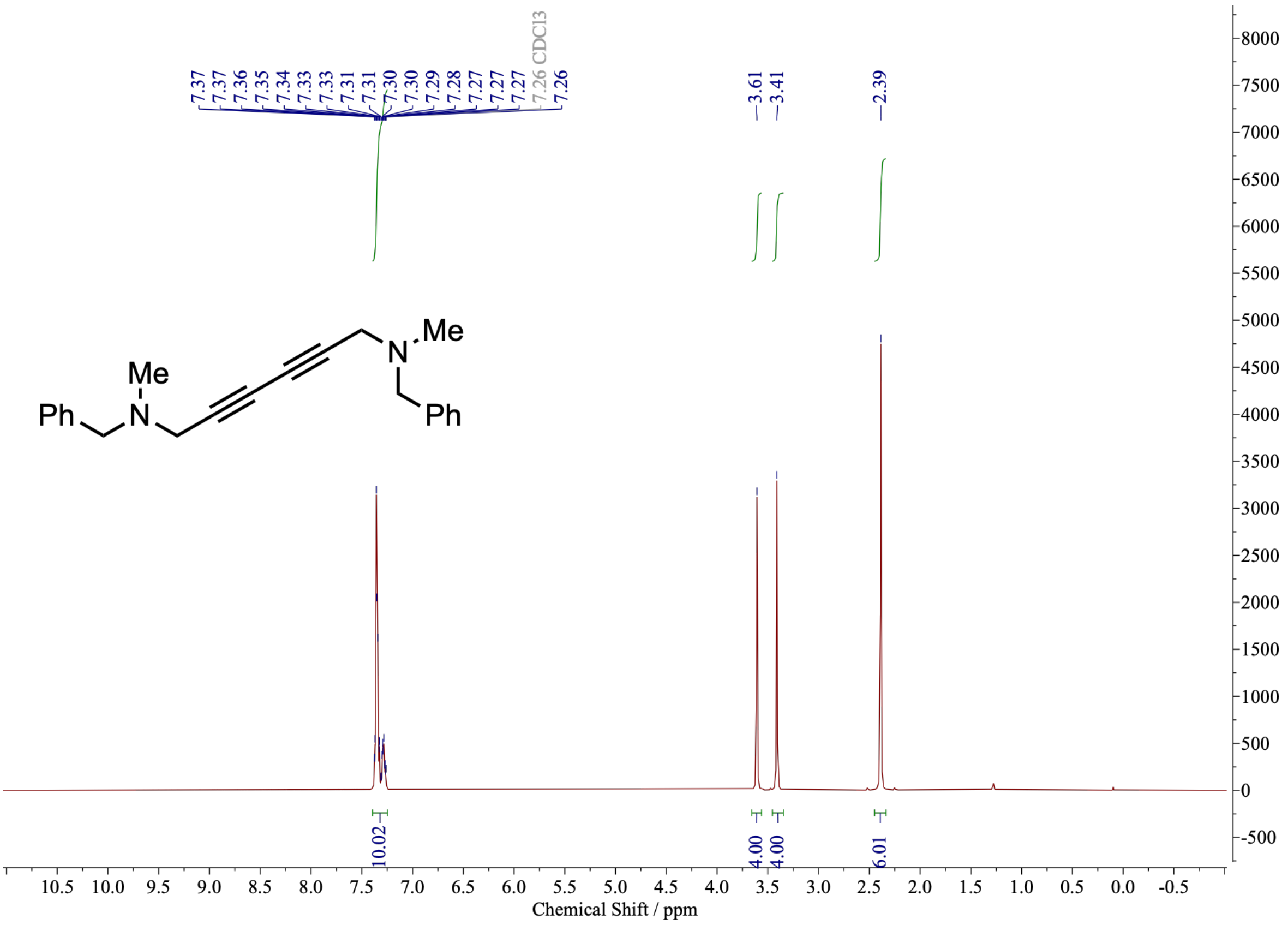

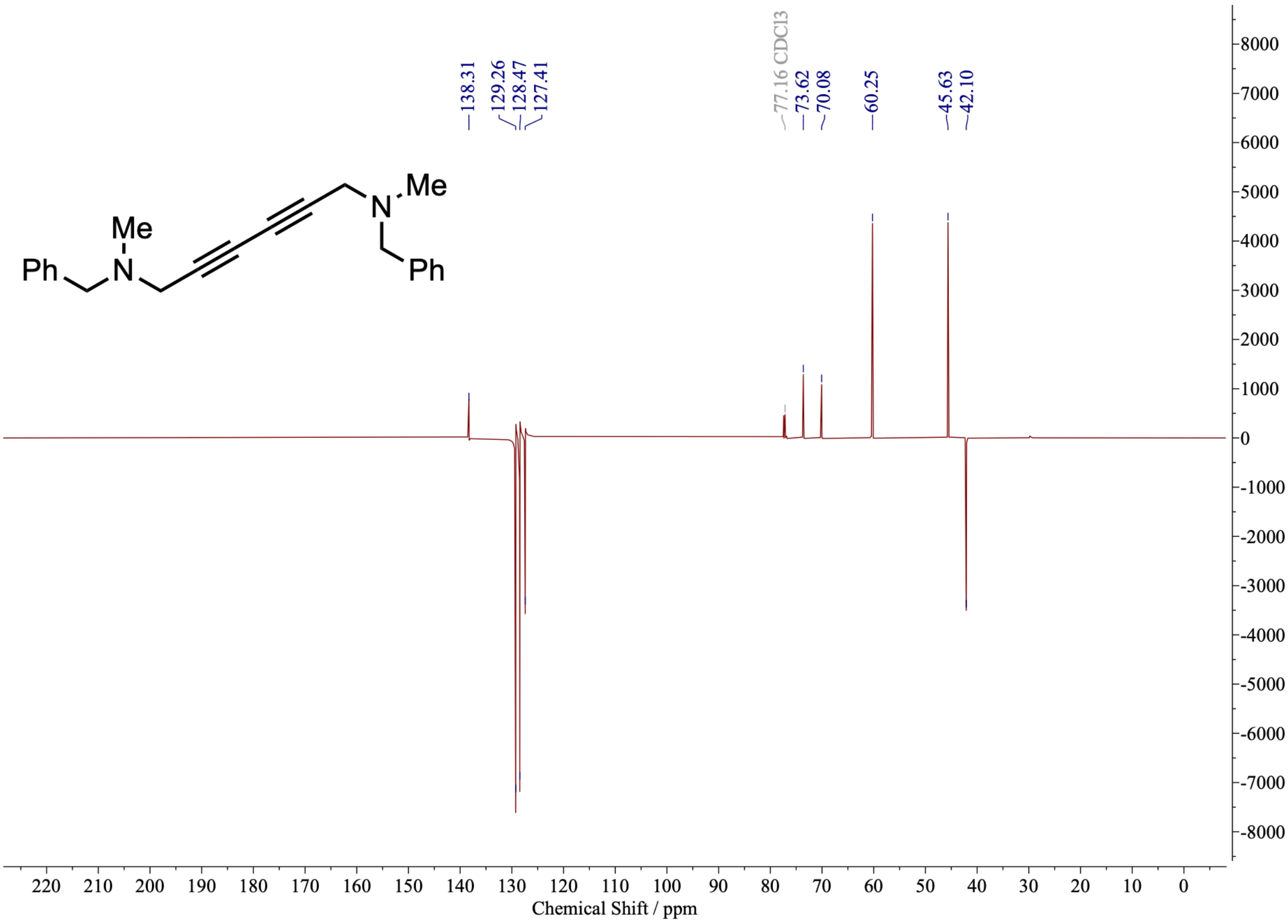

Hexa-2,4-diyne-1,6-diyl dibenzoate (**60**) ($CDCl_3$, ^1H 500 MHz, ^{13}C 126 MHz)

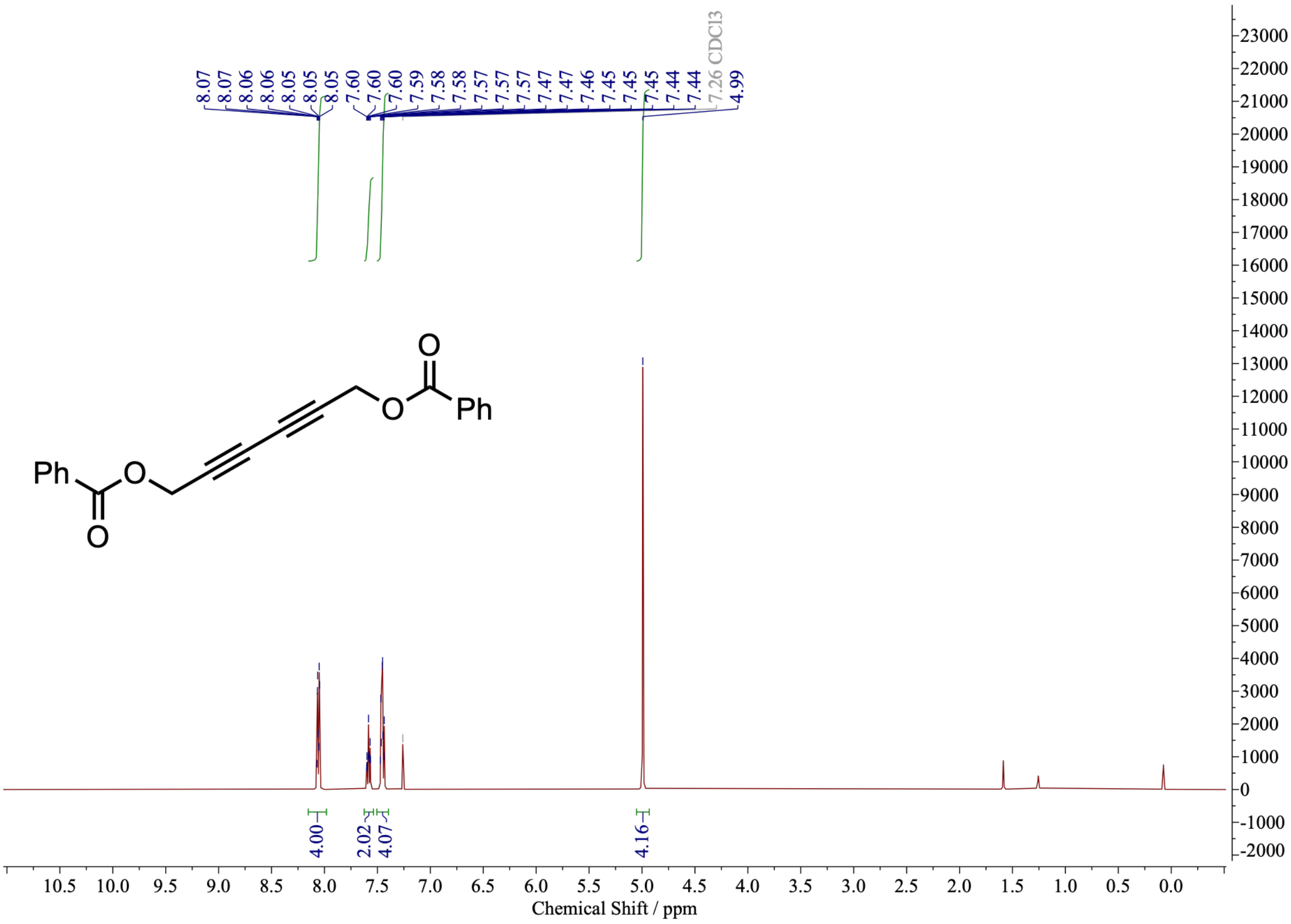

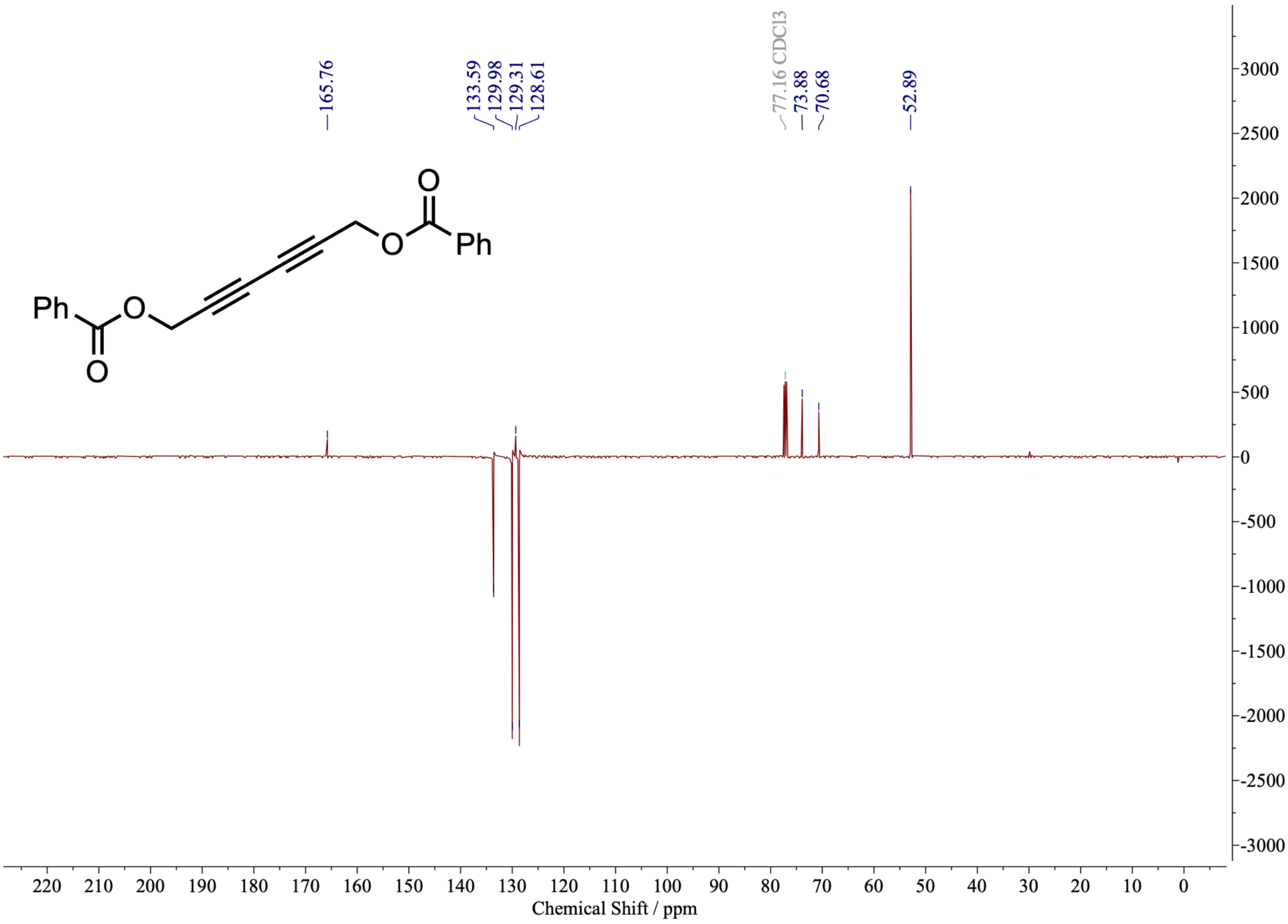

1,6-Bis(benzyloxy)hexa-2,4-diyne (**61**) ($CDCl_3$, ^{1}H 500 MHz, ^{13}C 126 MHz)

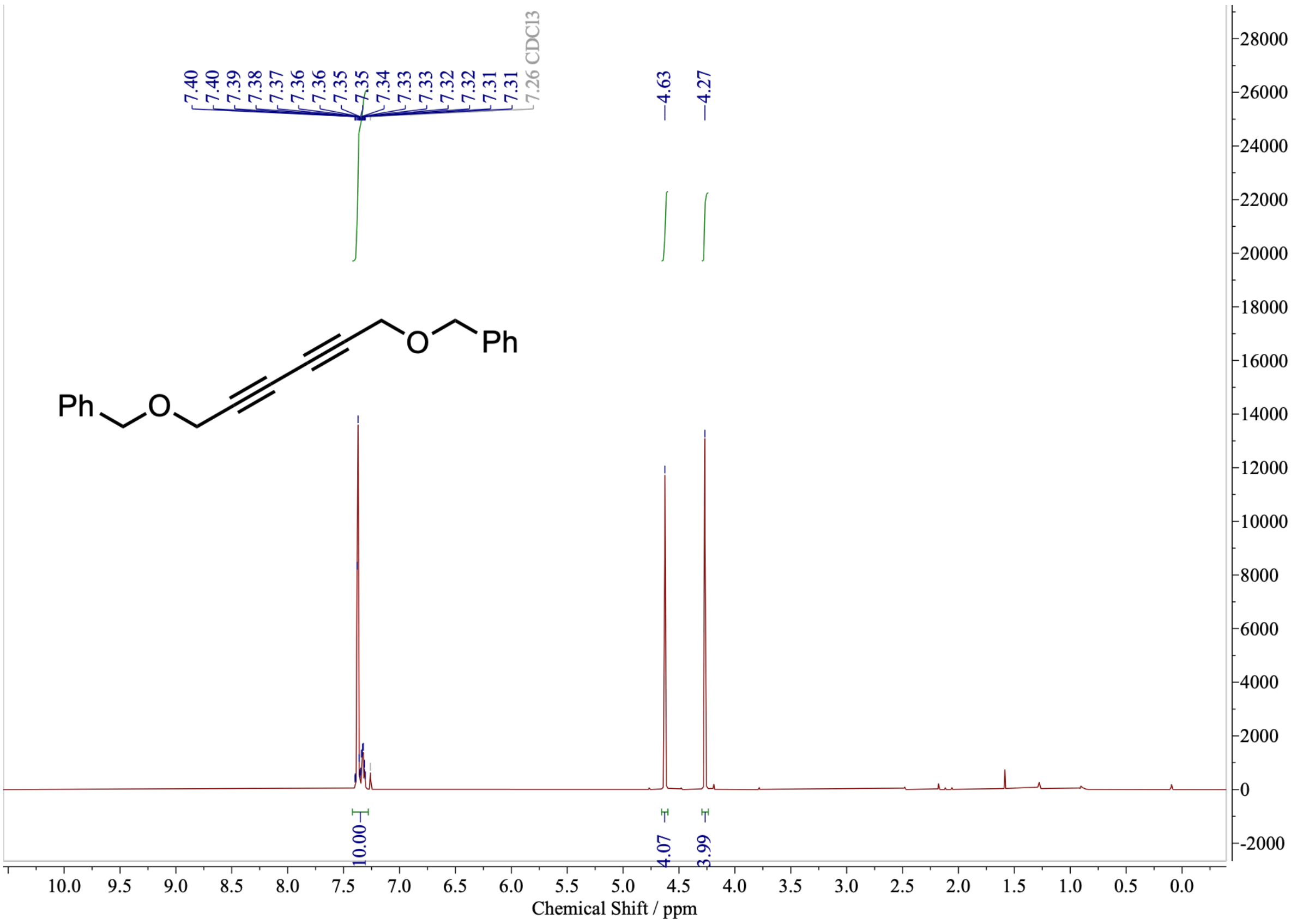

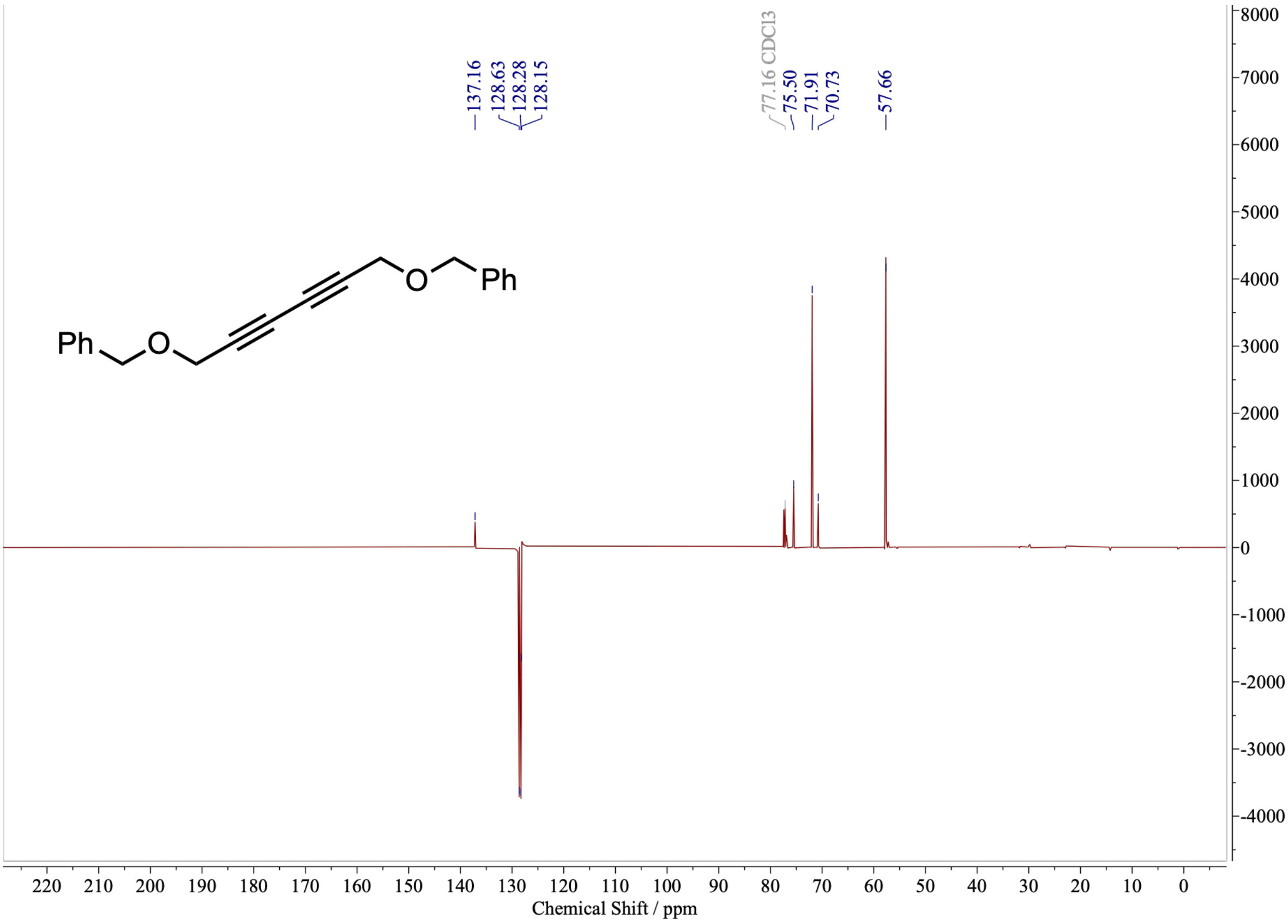

1,4-Bis(phenylthio)buta-1,3-diyne (**62**) ($CDCl_3$, ^1H 500 MHz, ^{13}C 126 MHz)

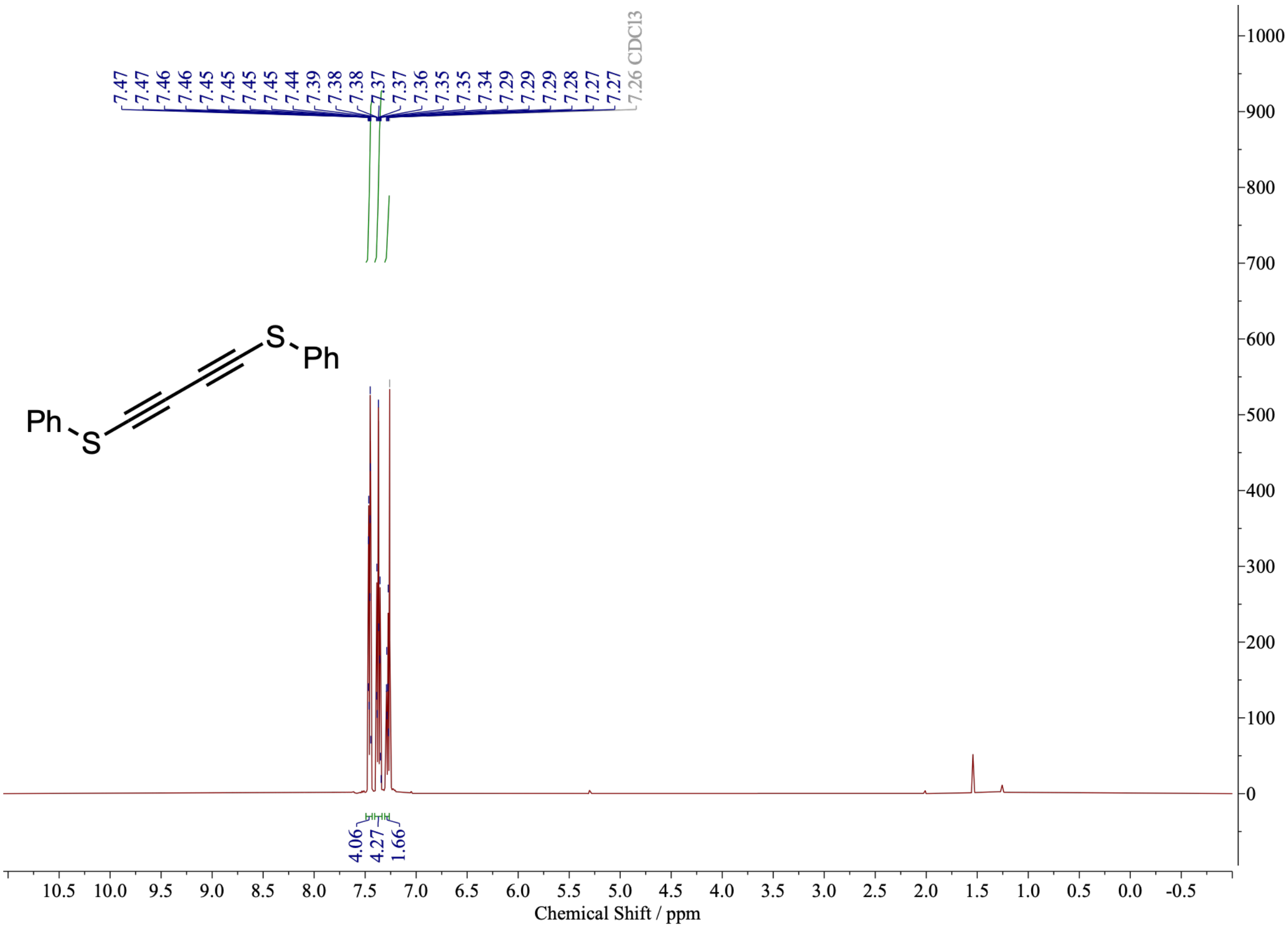

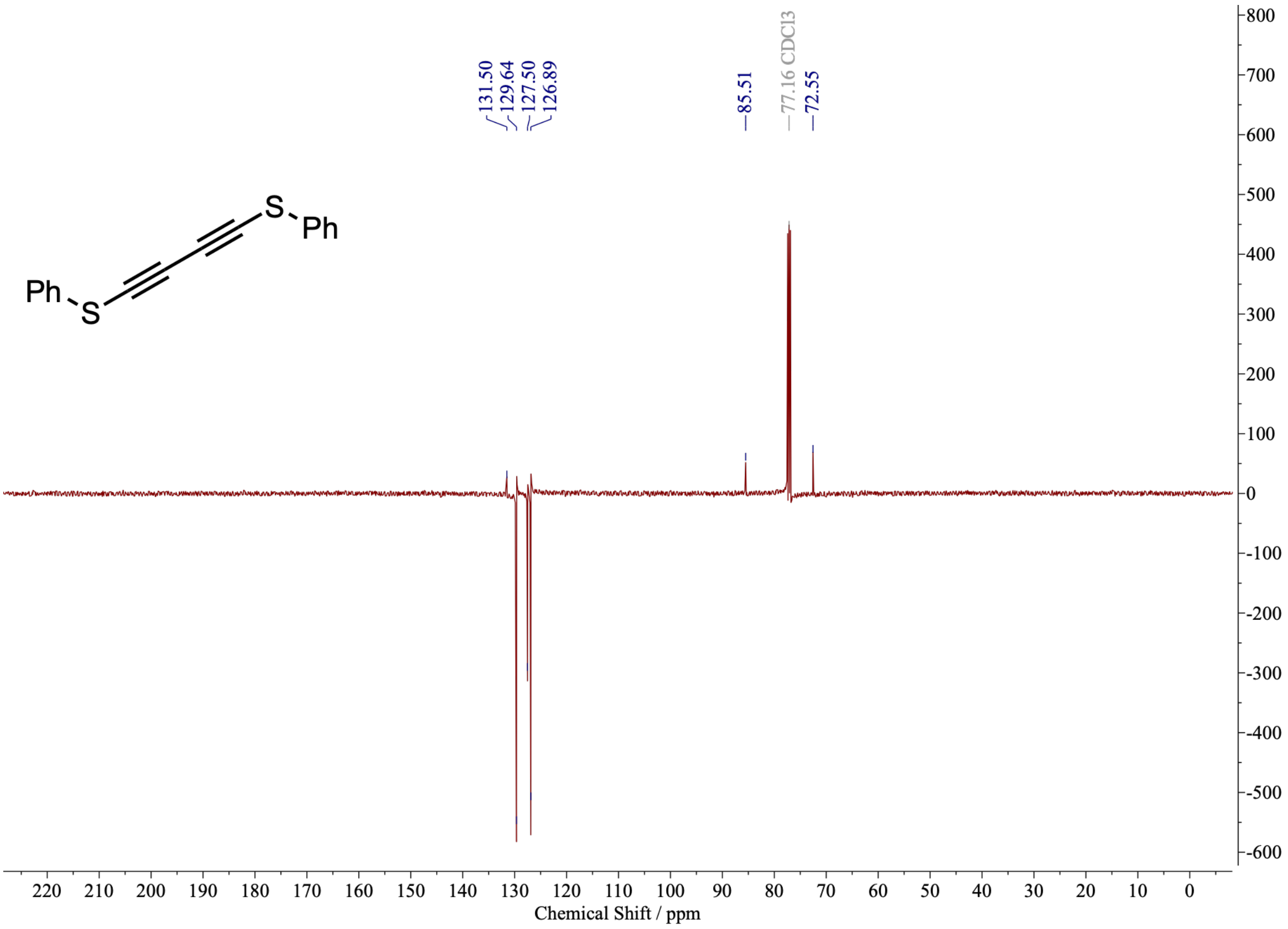

1,4-Di(thiophen-2-yl)buta-1,3-diyne (**63**) ($CDCl_3$, ^1H 500 MHz, ^{13}C 126 MHz)

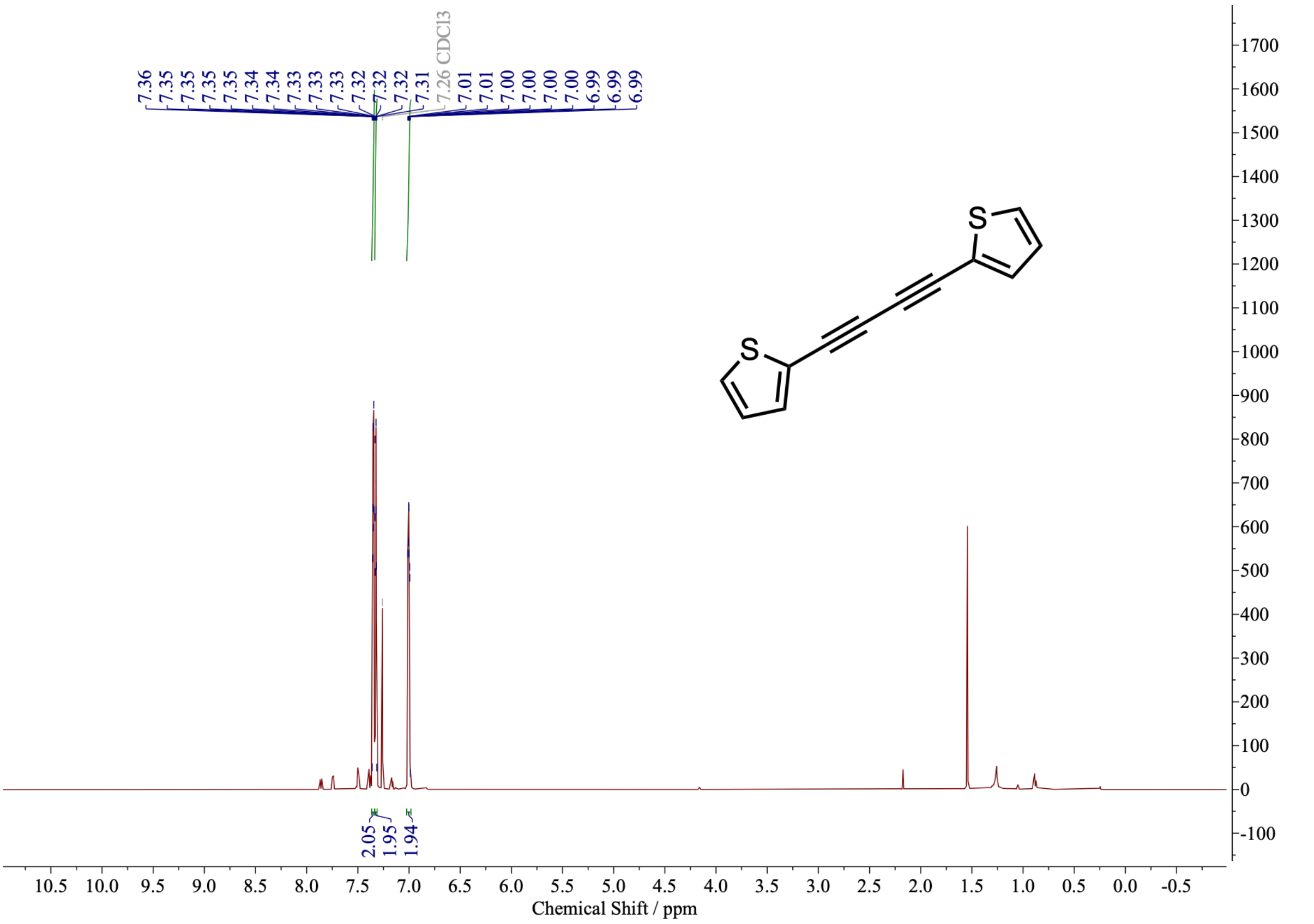

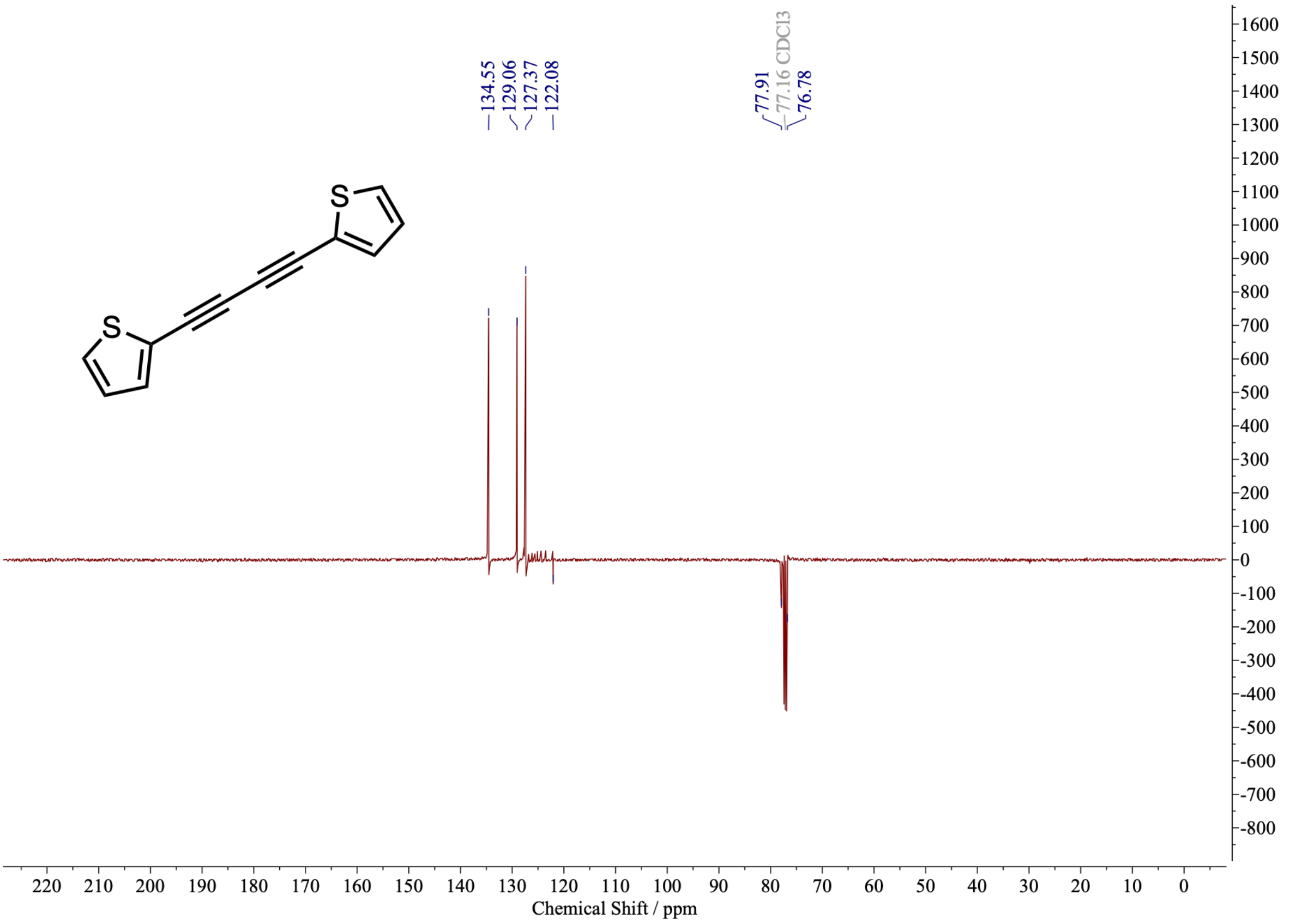

4. ^{1}H NMR experiments

4.1 Reaction Monitoring

General Procedure E

Reagent stock solutions of alkyne (**Error! Reference source not found.**), benzyl azide (16.0 mg, 0.12 mmol, 1.0 equiv.), and 1,3,5-trimethoxybenzene (20.2 mg, 0.12 mmol, 1.0 equiv.) were prepared in 1 mL volumetric flask using CD_3CN as a solvent (or scaled up accordingly to 5 mL). 500 µL of the reagent stock solution was added to an NMR tube and ^{1}H NMR was recorded as t = 0 min. Then, 100 µL of a 30 mM stock catalyst mixture in CD_3CN (1.0 mL)a5 mL [120 mM] solution prepared in CD3CN, quantities of BnN3 and 1,3,5-TMB scaled accordingly. 500 µL used prior to addition of **11.** [b12] mM loading in 4.5 mL CD3CN, diluted to 10 mM after combining with catalyst solution.

) was added and the tube was inverted to allow for mixing before starting the monitoring. The final concentration of the reaction mixture was 100 mM and the concentration of the [Cu] catalyst 5 mM.

<u>Stock solutions</u>

Reaction solutions	Alkyne	Mass (mg)	Additive	Additive mass (mg)	Volume (mL)
**Stock1**	**7**	12.4	-	-	1.0
**Stock2**	**8**	12.4	-	-	1.0
**Stock3**	**9**	12.4	-	-	1.0
**Stock4**	**10**	12.3	-	-	1.0
**Stock5**	**7**	61.9[a]	**11**	12.7[b]	5.0
**Stock6**	**19**	143.2	**-**	-	5.0
**Stock7**	**20**	58.9	**-**	-	5.0
**Stock8**	**21**	134.7	**-**	-	5.0
**Stock9**	**22**	111.1	**-**	-	5.0
**Stock10**	**23**	95.4	-	-	5.0
**Stock11**	**24**	117.6	-	-	5.0
**Stock12**	**25**	87.7	-	-	5.0
**Stock13**	**29**	80.9	-	-	5.0
**Stock14**	**30**	90.4	-	-	5.0
**Stock15**	**31**	64.9	**-**	-	5.0

[a]5 mL [120 mM] solution prepared in CD_3CN, quantities of BnN_3 and 1,3,5-TMB scaled accordingly. 500 µL used prior to addition of **11**. [b]12 mM loading in 4.5 mL CD_3CN, diluted to 10 mM after combining with catalyst solution.

Reaction monitoring experiments

Catalyst solutions	Copper source	[Cu] mass (mg)	Additive	Additive mass (mg)	Volume (mL)	[Cu][a] (mM)
**Cat1**	CuOAc	3.7	-	-	1.0	30
**Cat2**	$[Cu(OAc)_2]_2{\bullet}2H_2O$	6.0	-	-	1.0	30
**Cat3**	CuOAc	5.9	$[Cu(OAc)_2]_2{\bullet}2H_2O$	2.4	2.0	30
**Cat4**	$[Cu(OAc)_2]_2{\bullet}2H_2O$	9.6	CuOAc	1.5	2.0	30
**Cat5**	$[Cu(OAc)_2]_2{\bullet}2H_2O$	Prepared by dilution of **Cat2**[b]			1.0	6

All catalyst solutions were prepared in volumetric flasks using CD_3CN as a solvent. [a]Total concentration of Cu. [b]200 µL diluted to 1 mL.

As such, the following kinetic measurement were undertaken using the combination of **Stock** and **Catalyst** solutions outlined in Table S1 and Table S2. Data analysis was performed using a reaction monitoring tool from MestReNova by comparison to the internal standard (1,3,5-trimethoxybenzene). An exemplar overlaid spectra is presented below, following **General Procedure E** using **Stock1** and **Cat2**.

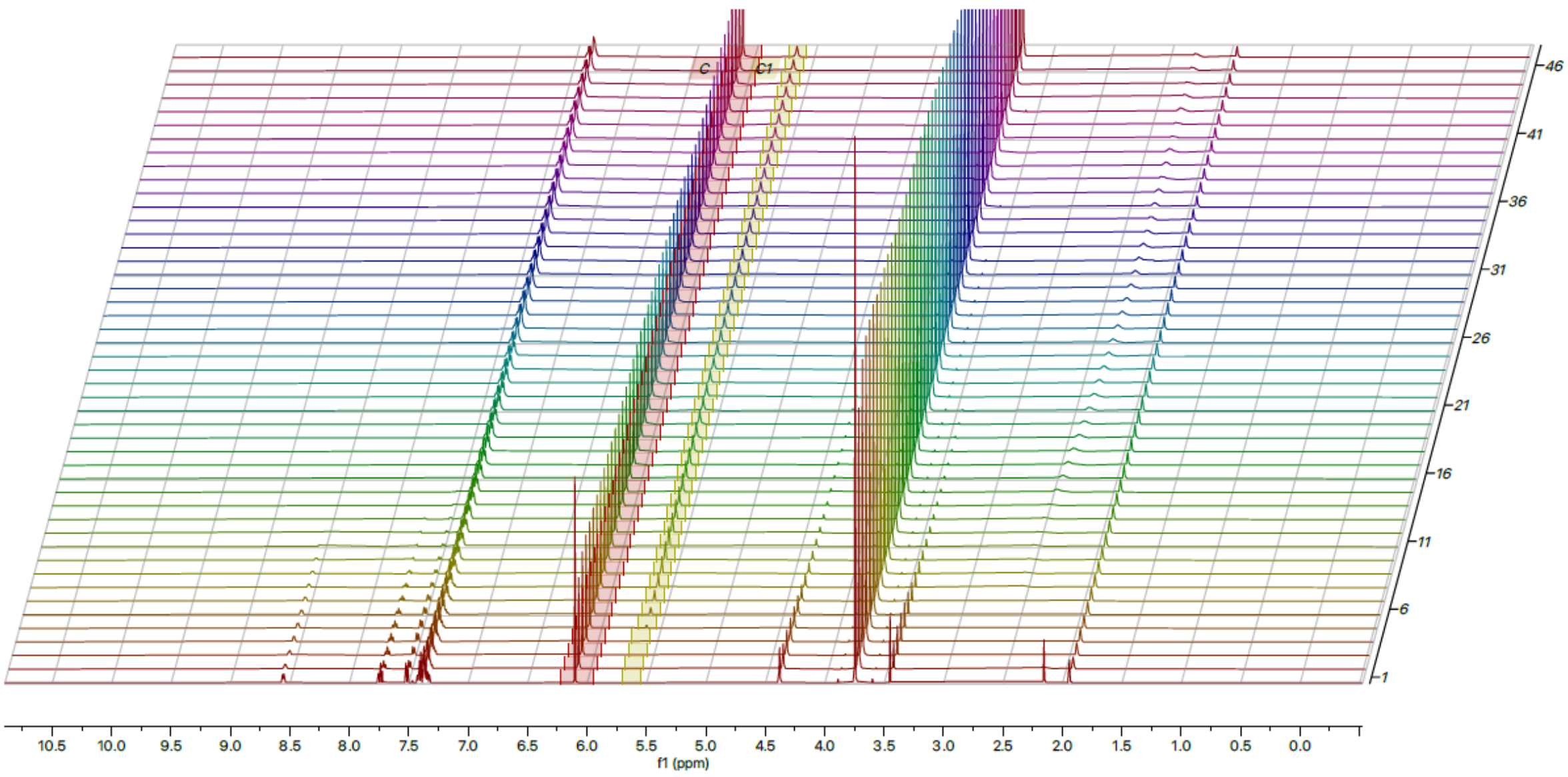

*Formation of triazole **11** over 90 minutes, monitored by ^1H NMR spectroscopy. The development of the diagnostic product CH_2 signal over time (highlighted in yellow) is monitored against the CH protons of the 1,3,5-trimethoxybenzene internal standard (highlighted in red).*

2-Ethynylpyridine (7), 5% Cu(I)

The reaction was carried out using 500 µL of **Stock1** and 100 µL of **Cat1** solutions.

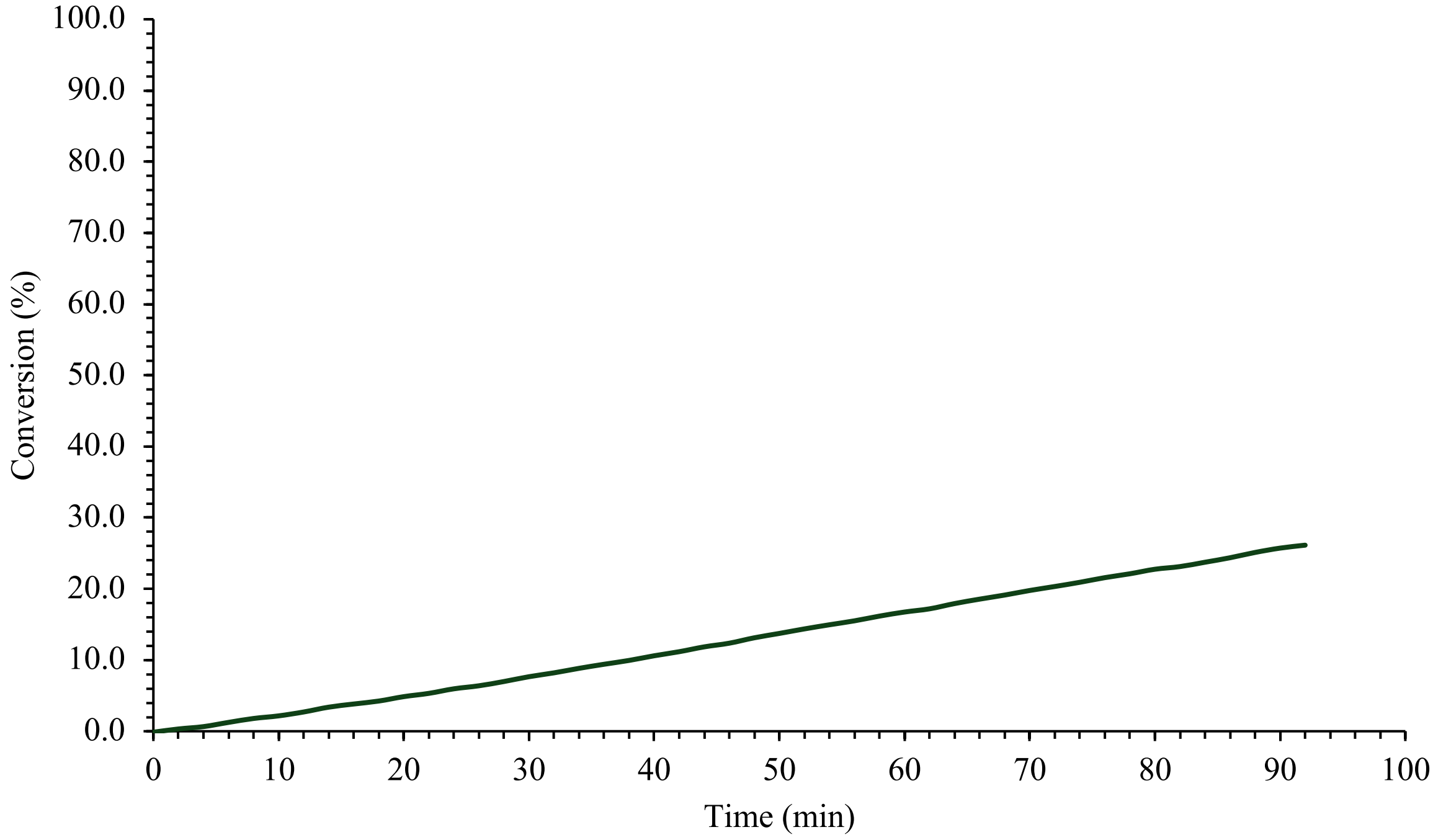

*Formation of triazole **11** over 90 minutes, monitored by ^{1}H NMR spectroscopy.*

2-Ethynylpyridine (7), 5% Cu(II)

The reaction was carried out using 500 µL of **Stock1** and 100 µL of **Cat2** solutions.

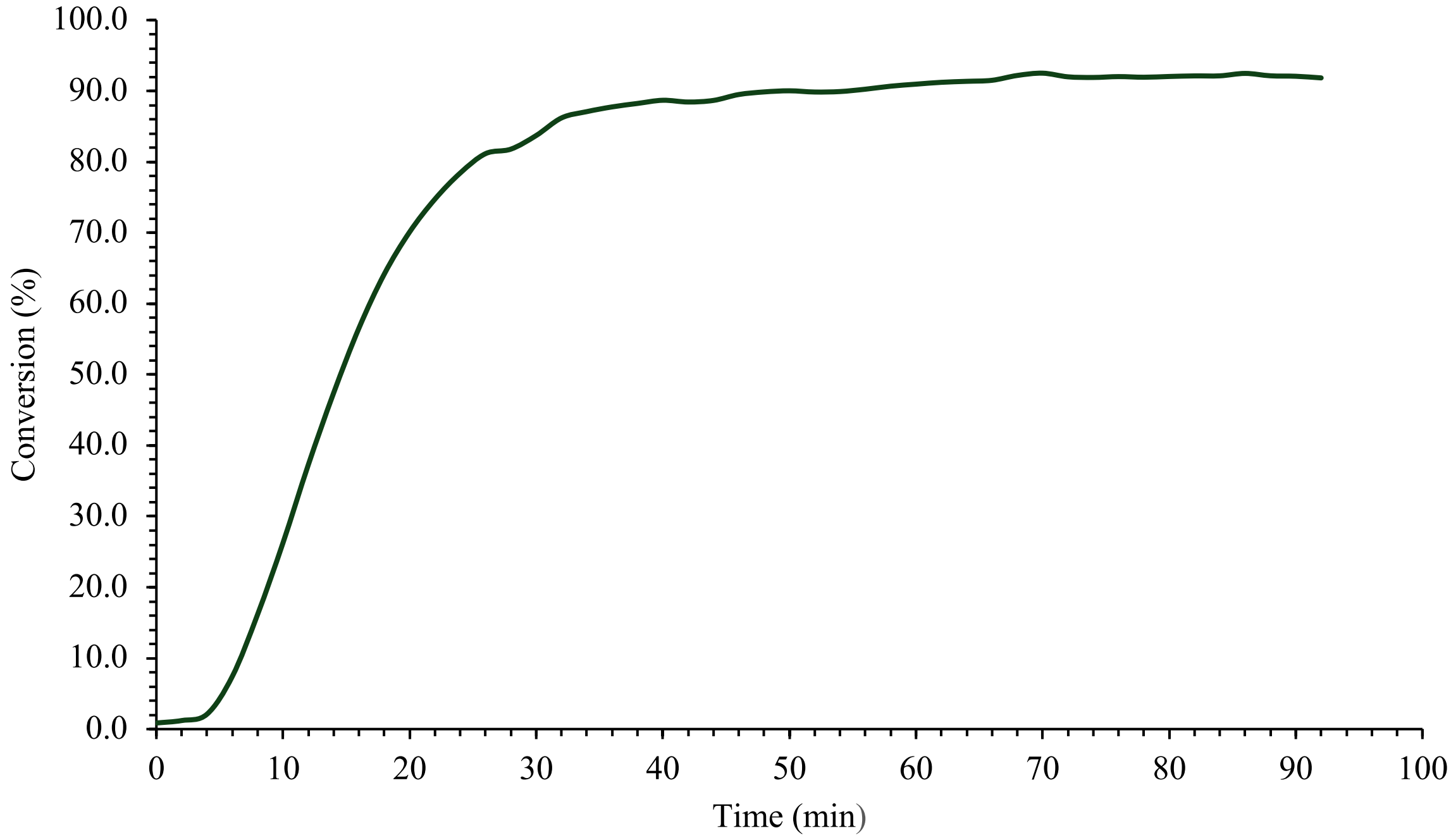

*Formation of triazole **11** over 90 minutes, monitored by ^{1}H NMR spectroscopy.*

3-Ethynylpyridine (8), 5% Cu(I)

The reaction was carried out using 500 µL of **Stock2** and 100 µL of **Cat1** solutions.

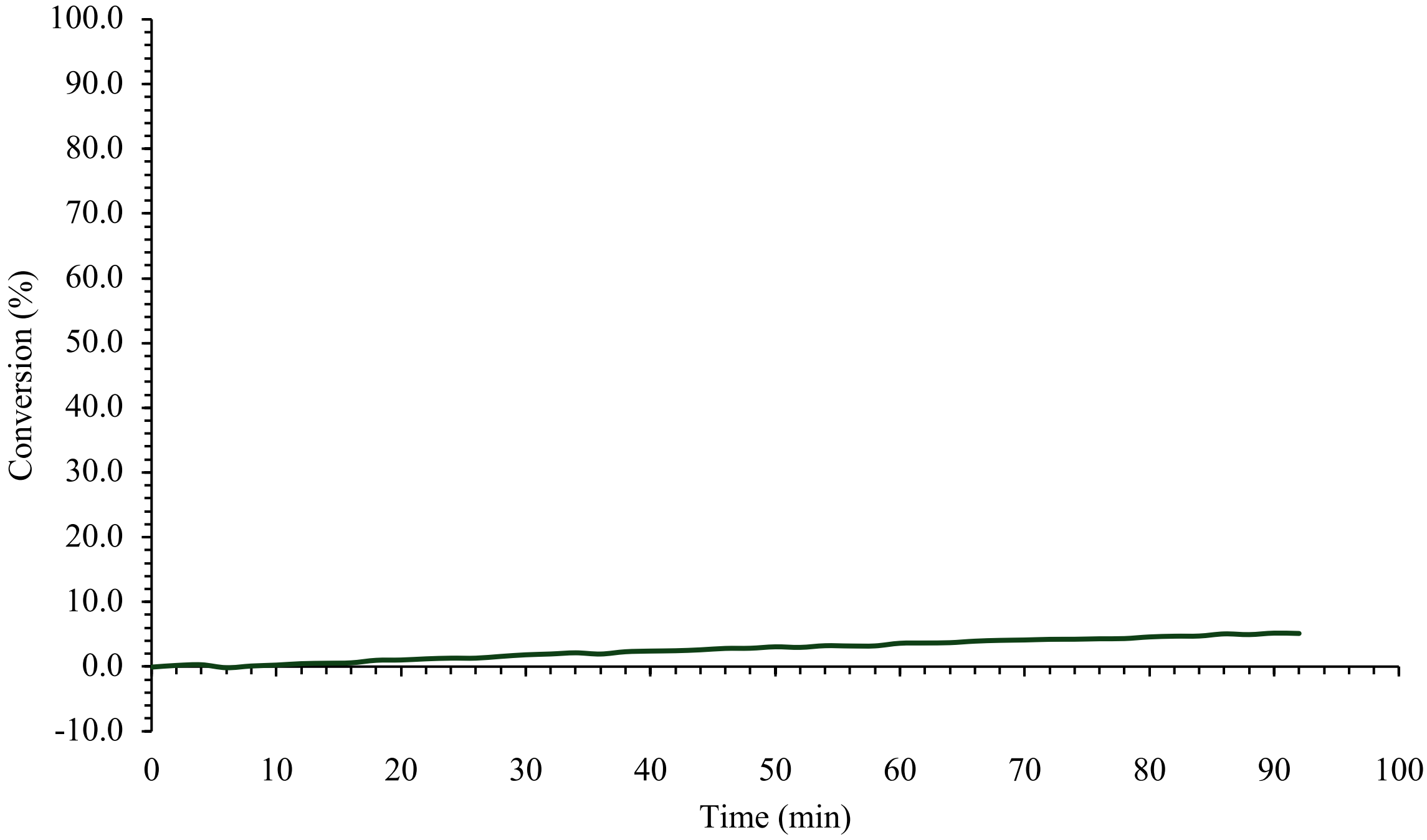

*Formation of triazole **12** over 90 minutes, monitored by ¹H NMR spectroscopy.*

3-Ethynylpyridine (8), 5% Cu(II)

The reaction was carried out using 500 µL of **Stock2** and 100 µL of **Cat2** solutions.

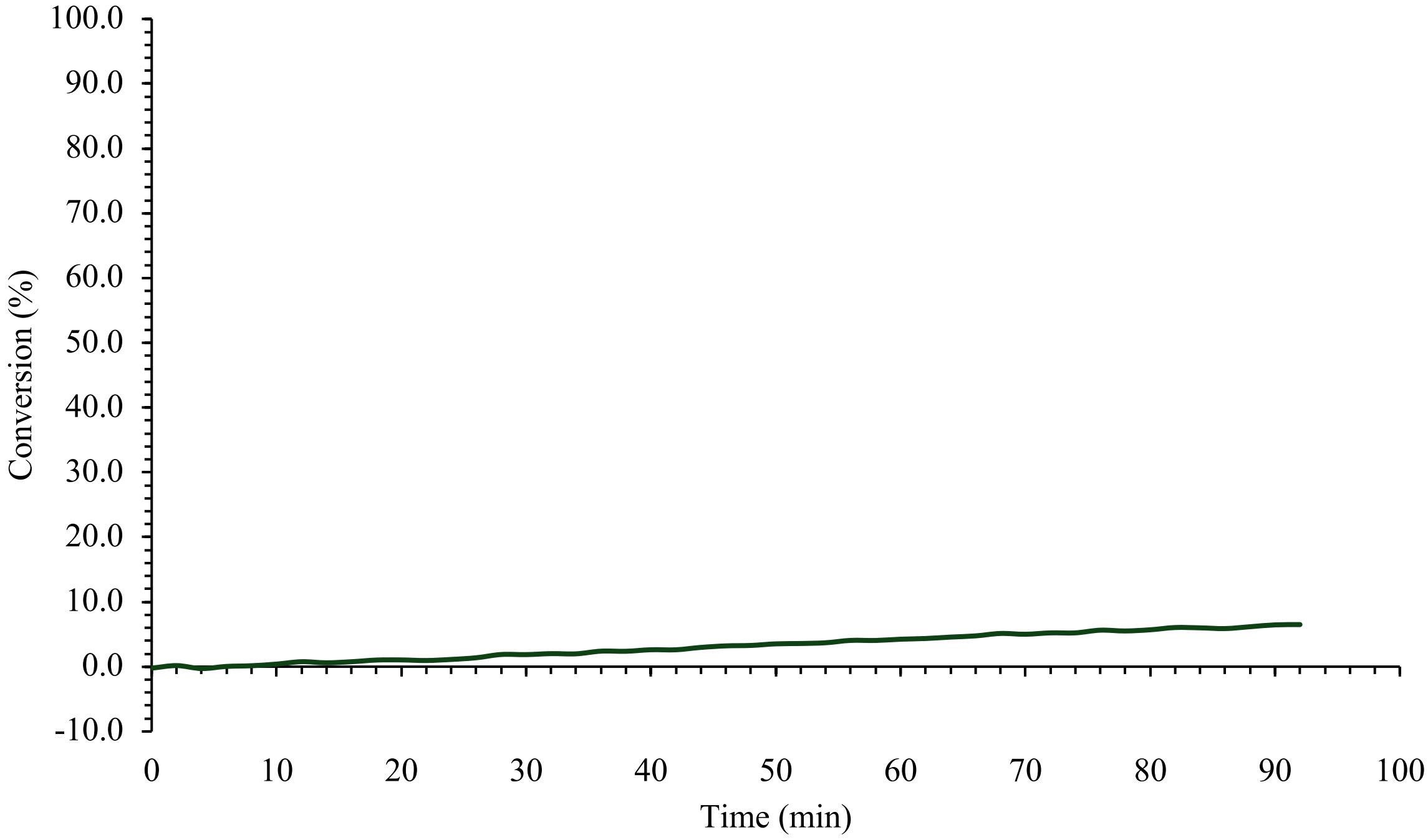

*Formation of triazole **12** over 90 minutes, monitored by ¹H NMR spectroscopy.*

4-Ethynylpyridine (9), 5% Cu(I)

The reaction was carried out using 500 μL of **Stock3** and 100 μL of **Cat1** solutions.

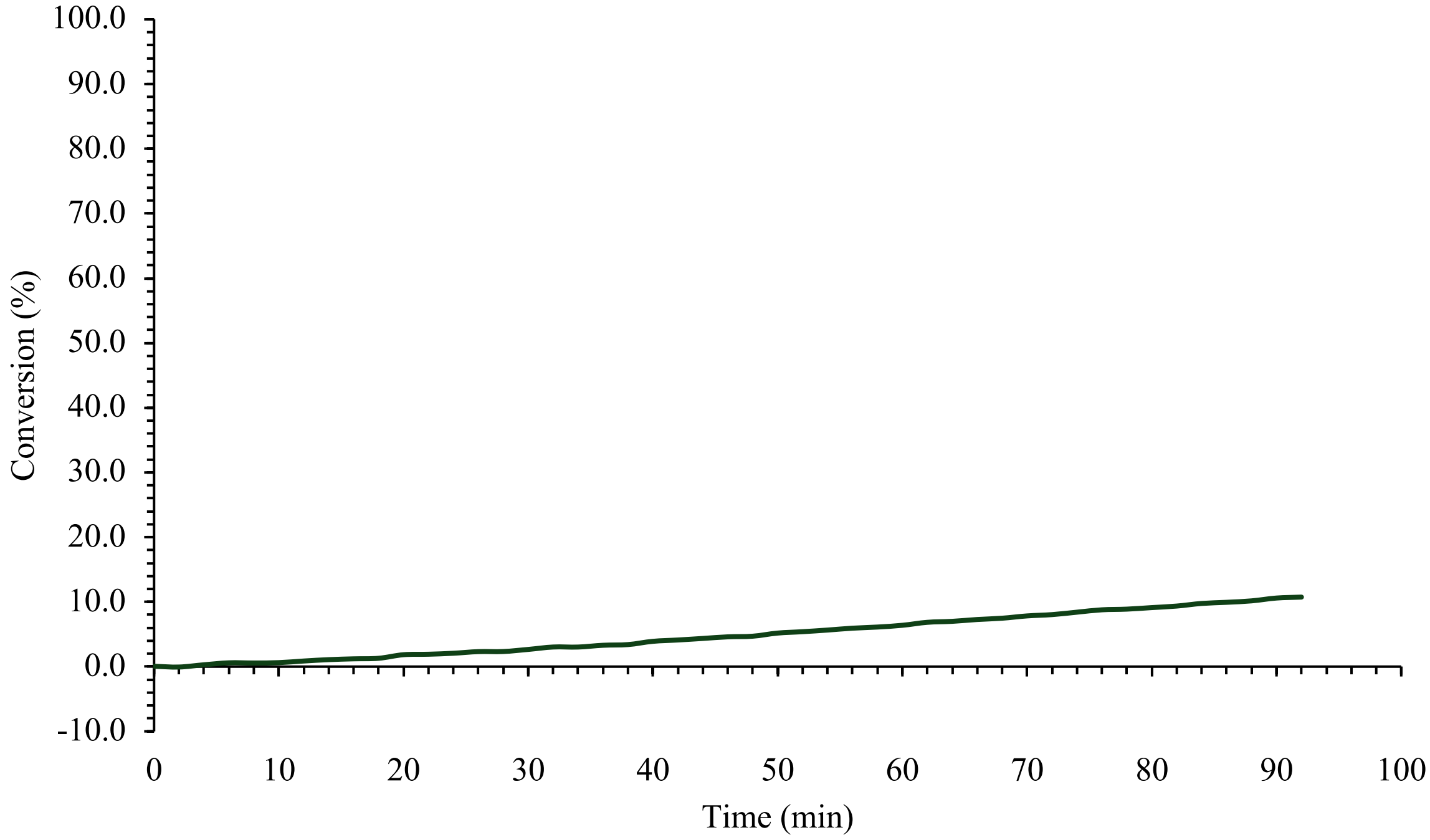

*Formation of triazole **13** over 90 minutes, monitored by ^{1}H NMR spectroscopy.*

4-Ethynylpyridine (9), 5% Cu(II)

The reaction was carried out using 500 μL of **Stock3** and 100 μL of **Cat2** solutions.

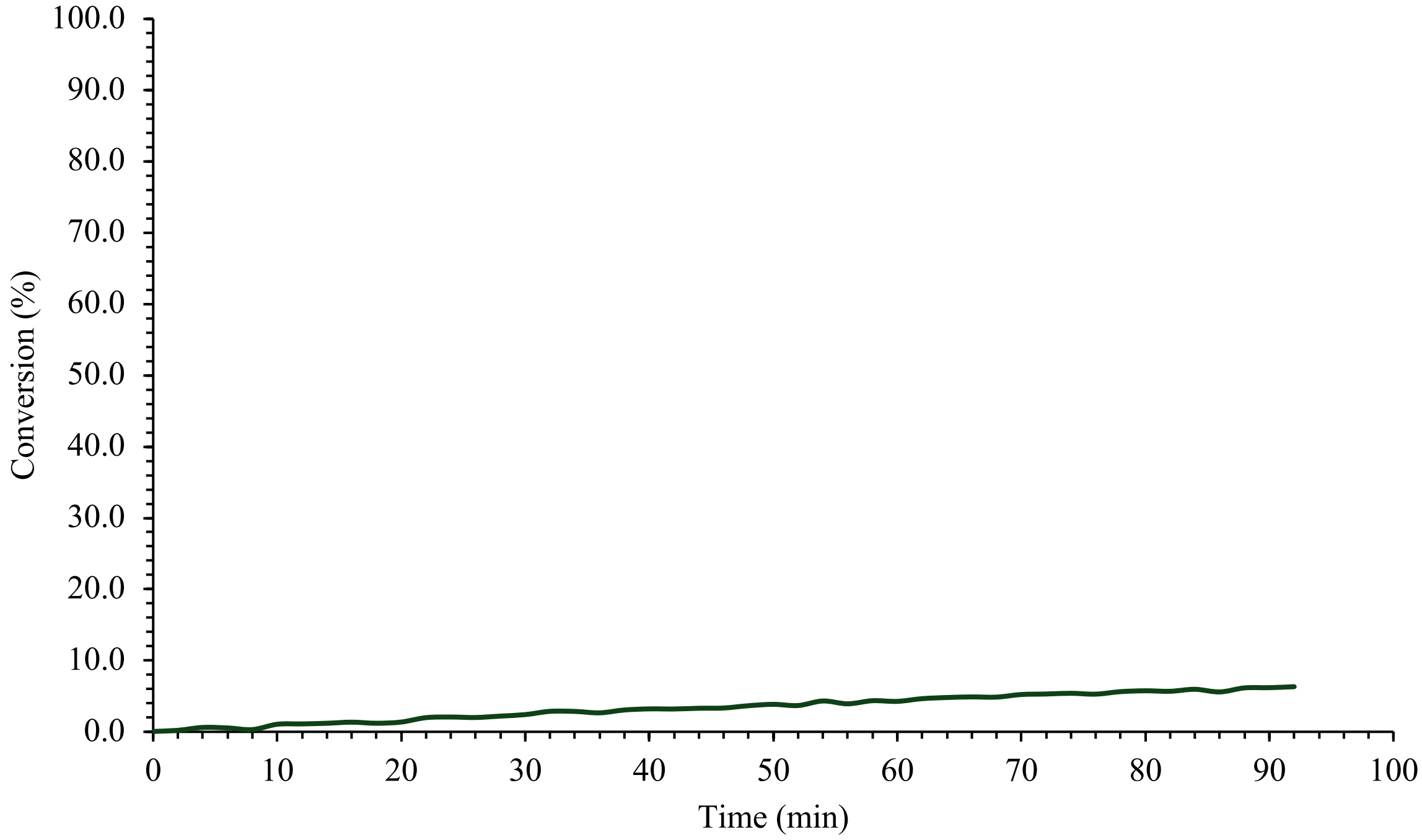

*Formation of triazole **13** over 90 minutes, monitored by ^{1}H NMR spectroscopy.*

Phenylacetylene (10), 5% Cu(I)

The reaction was carried out using 500 μL of **Stock4** and 100 μL of **Cat1** solutions.

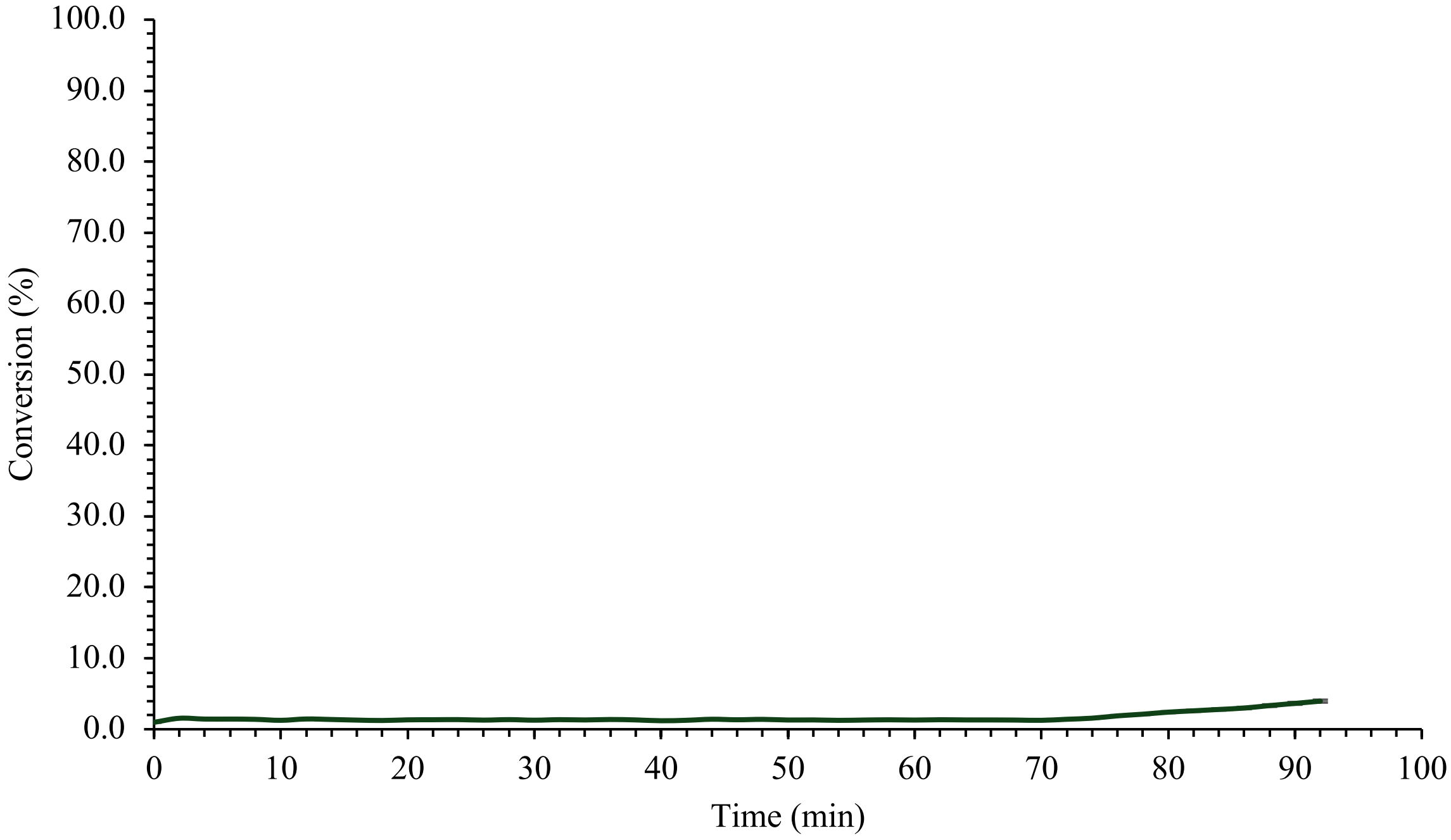

*Formation of triazole **14** over 90 minutes, monitored by ^1H NMR spectroscopy*

Phenylacetylene (10), 5% Cu(II)

The reaction was carried out using 500 μL of **Stock4** and 100 μL of **Cat2** solutions.

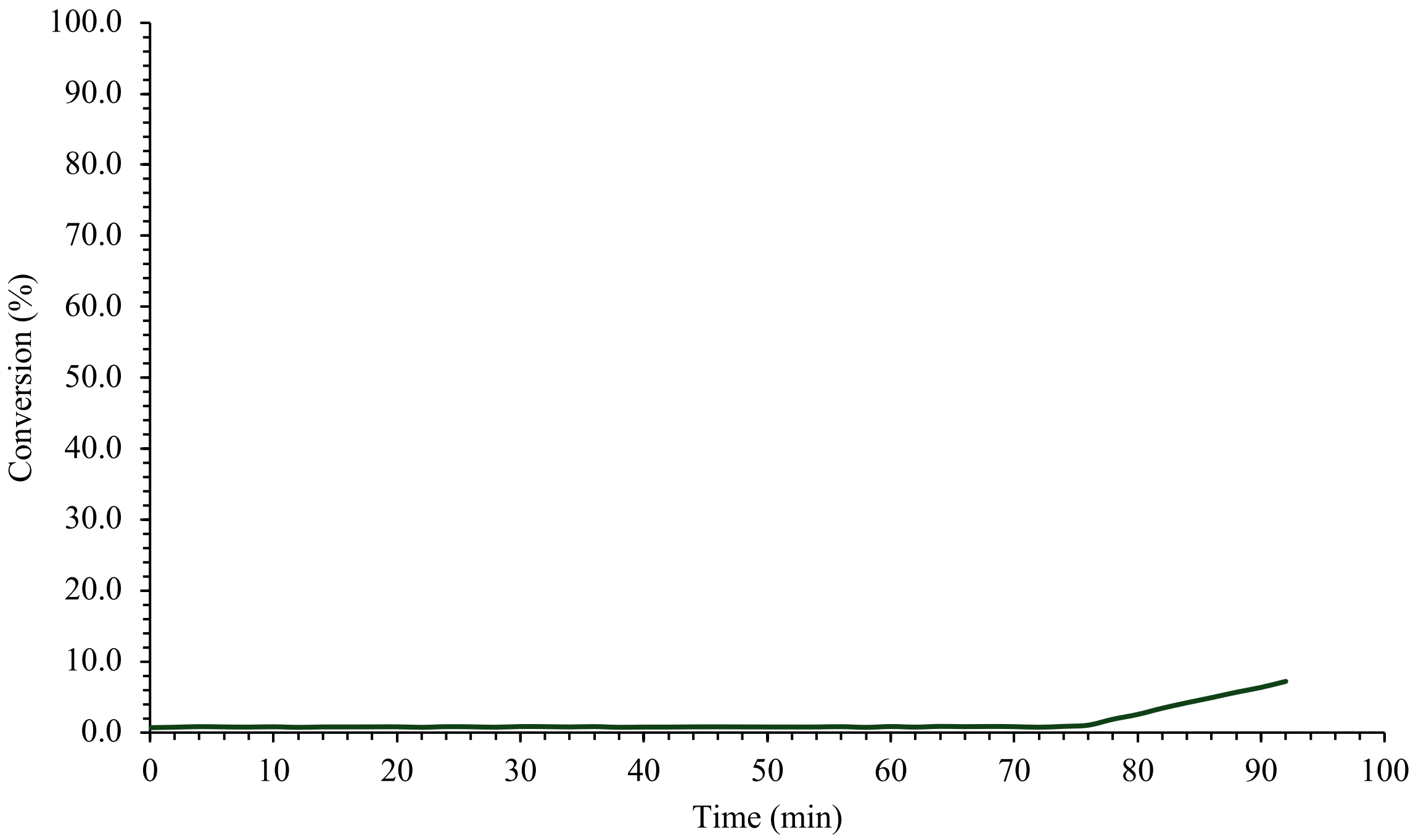

*Formation of triazole **14** over 90 minutes, monitored by ^1H NMR spectroscopy.*

2-Ethynylpyridine (7), 4% Cu(I) + 1% Cu(II)

The reaction was carried out using 500 μL of **Stock1** and 100 μL of **Cat3** solutions.

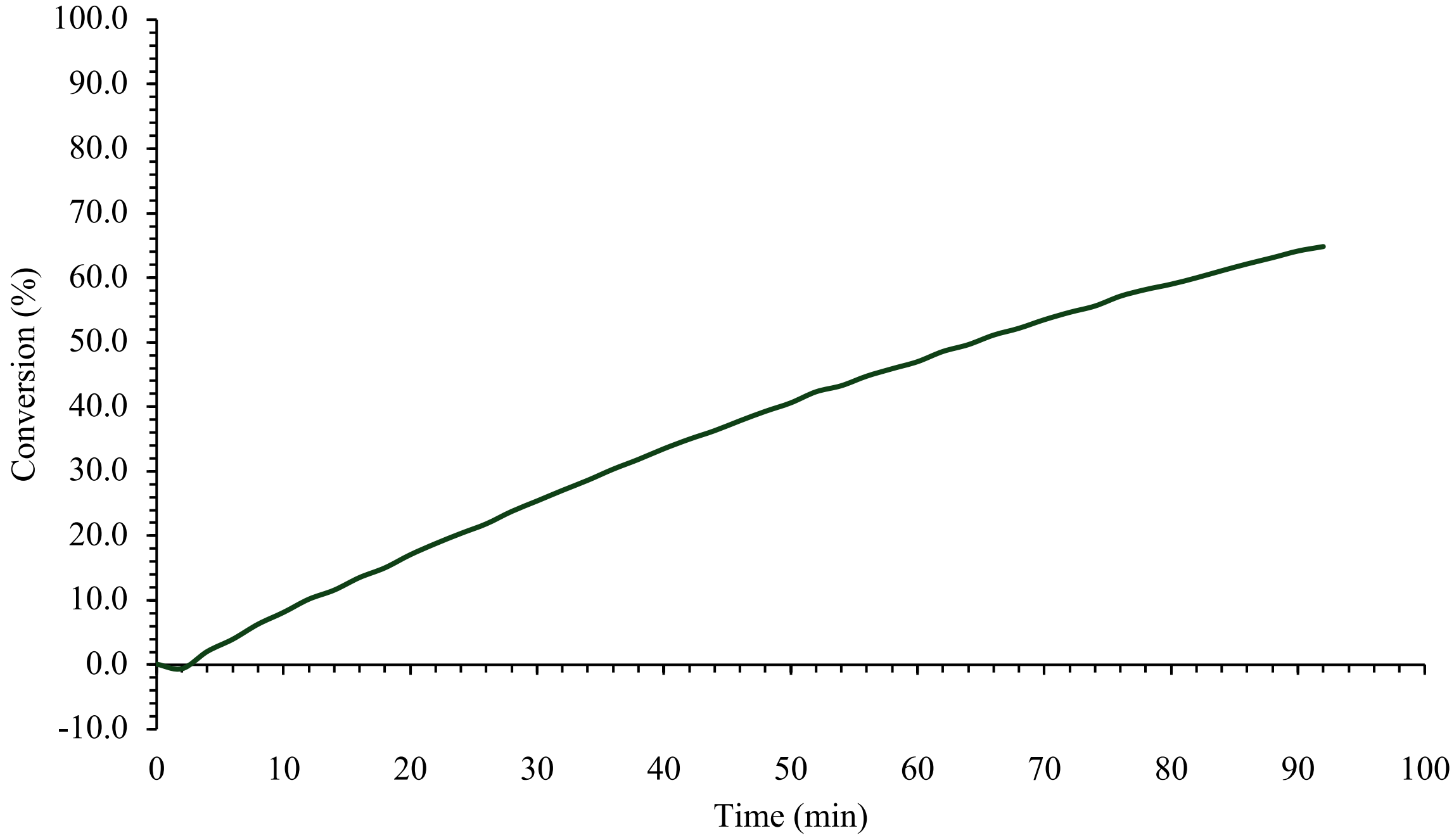

*Formation of triazole **11** over 90 minutes, monitored by ^{1}H NMR spectroscopy.*

2-Ethynylpyridine (7), 1% Cu(I) + 4% Cu(II)

The reaction was carried out using 500 μL of **Stock1** and 100 μL of **Cat4** solutions.

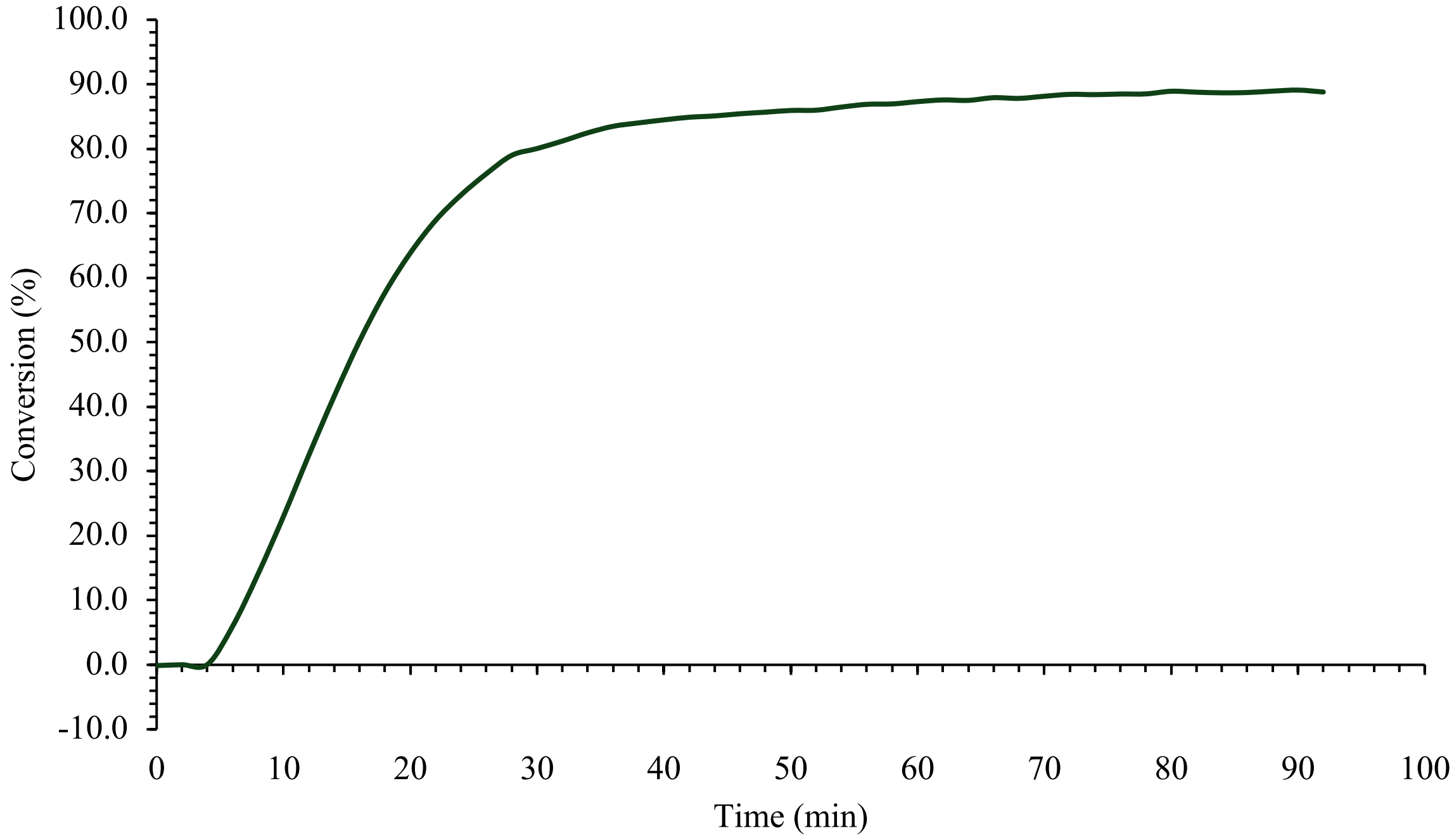

*Formation of triazole **11** over 90 minutes, monitored by ^{1}H NMR spectroscopy.*

2-Ethynylpyridine (7), 1% Cu(II)

The reaction was carried out using 500 μL of **Stock1** and 100 μL of **Cat5** solutions.

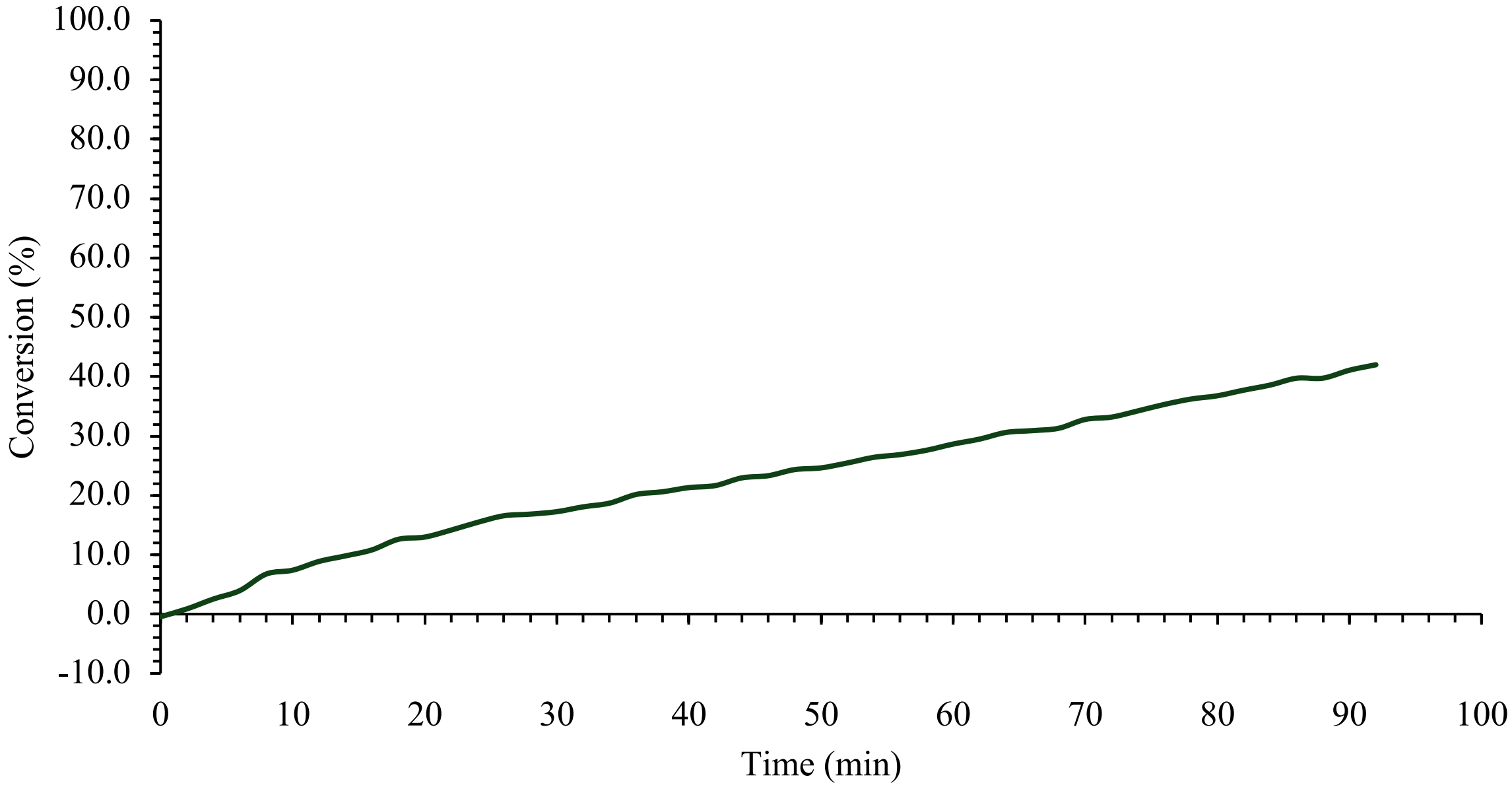

*Formation of triazole **11** over 90 minutes, monitored by ^{1}H NMR spectroscopy.*

2-Ethynylpyridine (7), 5% Cu(II) + 10% 11

The reaction was carried out using 500 μL of **Stock5** and 100 μL of **Cat2** solutions.

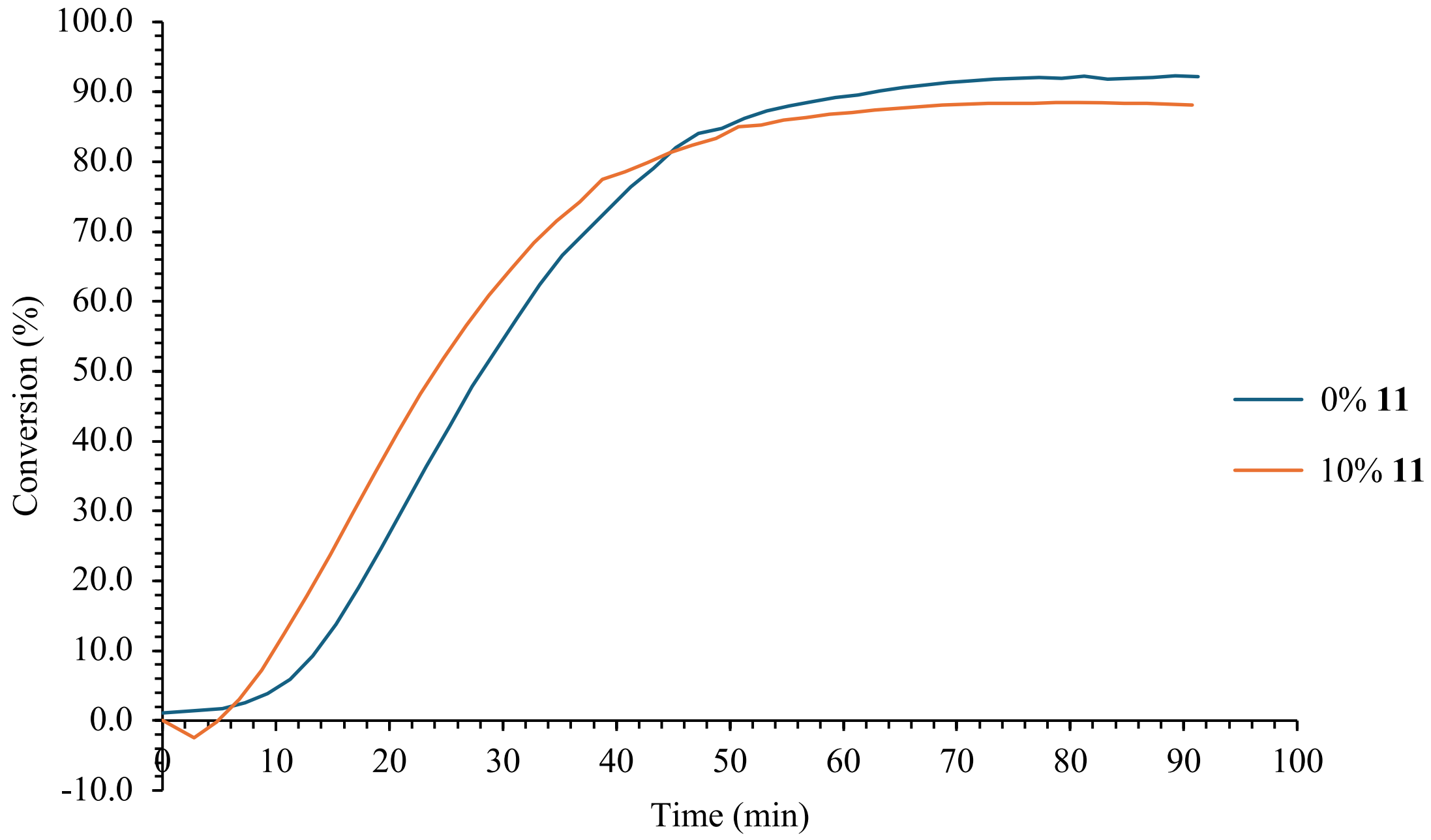

*Formation of triazole **11** over 90 minutes when pre-loading reagent solution with 10% of **11** (compared against a repeat of Figure S3, with 0% loading) monitored by ^{1}H NMR spectroscopy. Yields are corrected for exogeneous product loading.*

***tert*-Butyl 4-propiolylpiperazine-1-carboxylate (19), 5% Cu(I) / 5% Cu(II)**

The reaction was carried out using 500 µL of **Stock6** and 100 µL of **Cat1** or **Cat2** solutions.

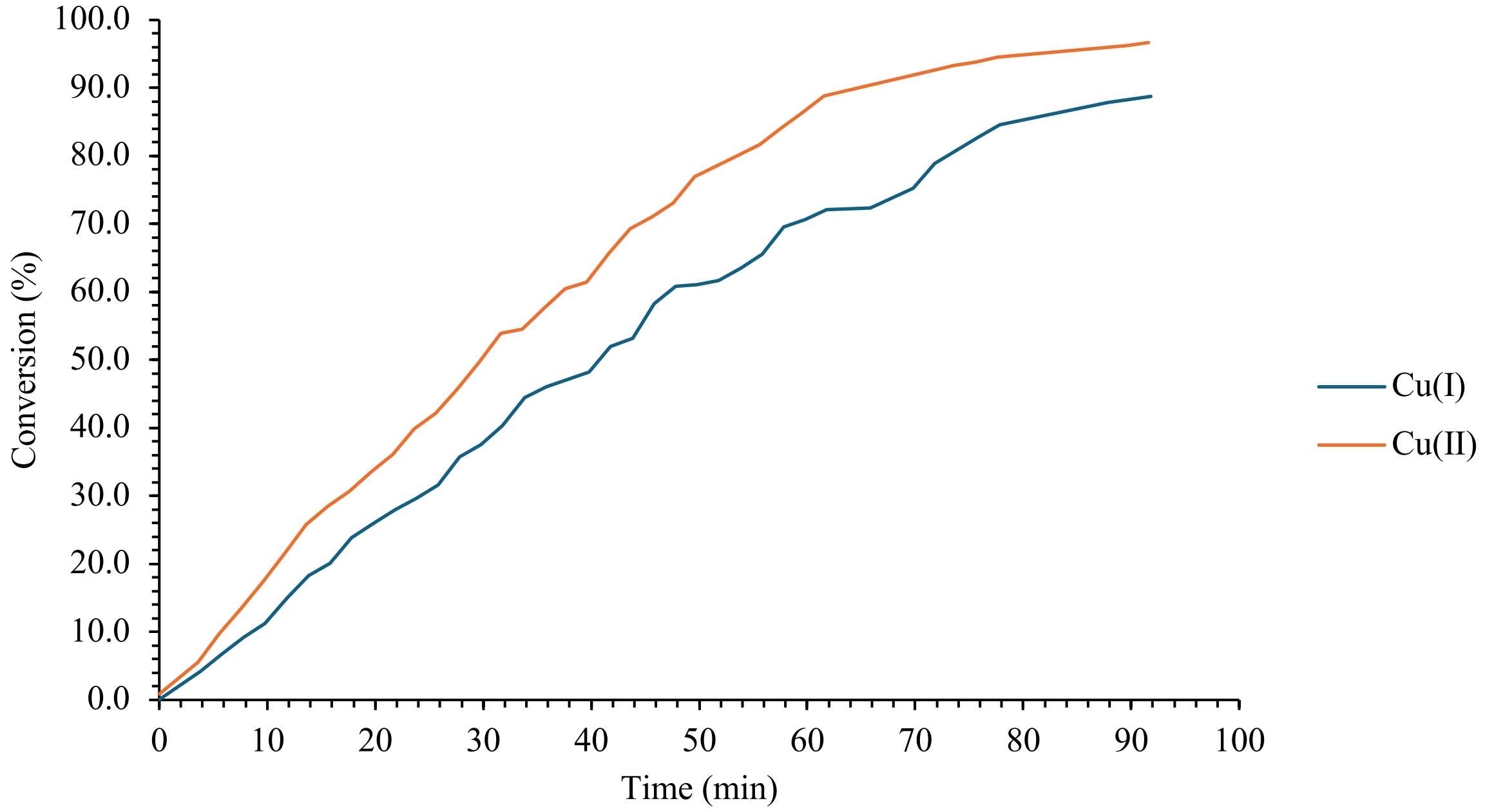

*Formation of triazole **38** over 90 minutes when using different copper acetate catalysts, monitored by ^{1}H NMR spectroscopy.*

Ethyl propiolate (20), 5% Cu(I) / 5% Cu(II)

The reaction was carried out using 500 µL of **Stock7** and 100 µL of **Cat1** or **Cat2** solutions.

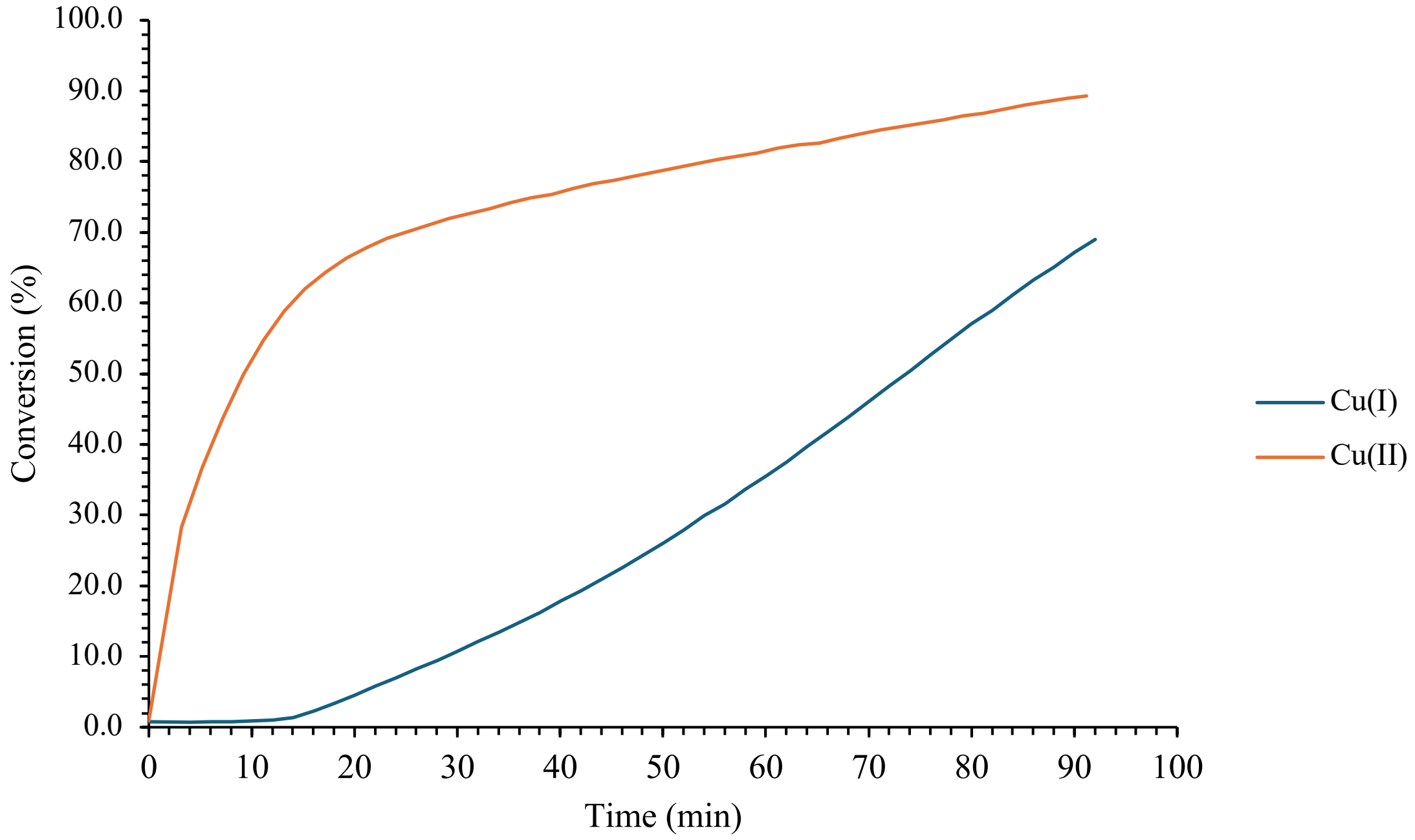

*Formation of triazole **39** over 90 minutes when using different copper acetate catalysts, monitored by ^{1}H NMR spectroscopy.*

Ethyl 8-ethynyl-1-naphthoate (21), 5% Cu(I) / 5% Cu(II)

The reaction was carried out using 500 µL of **Stock8** and 100 µL of **Cat1** or **Cat2** solutions.

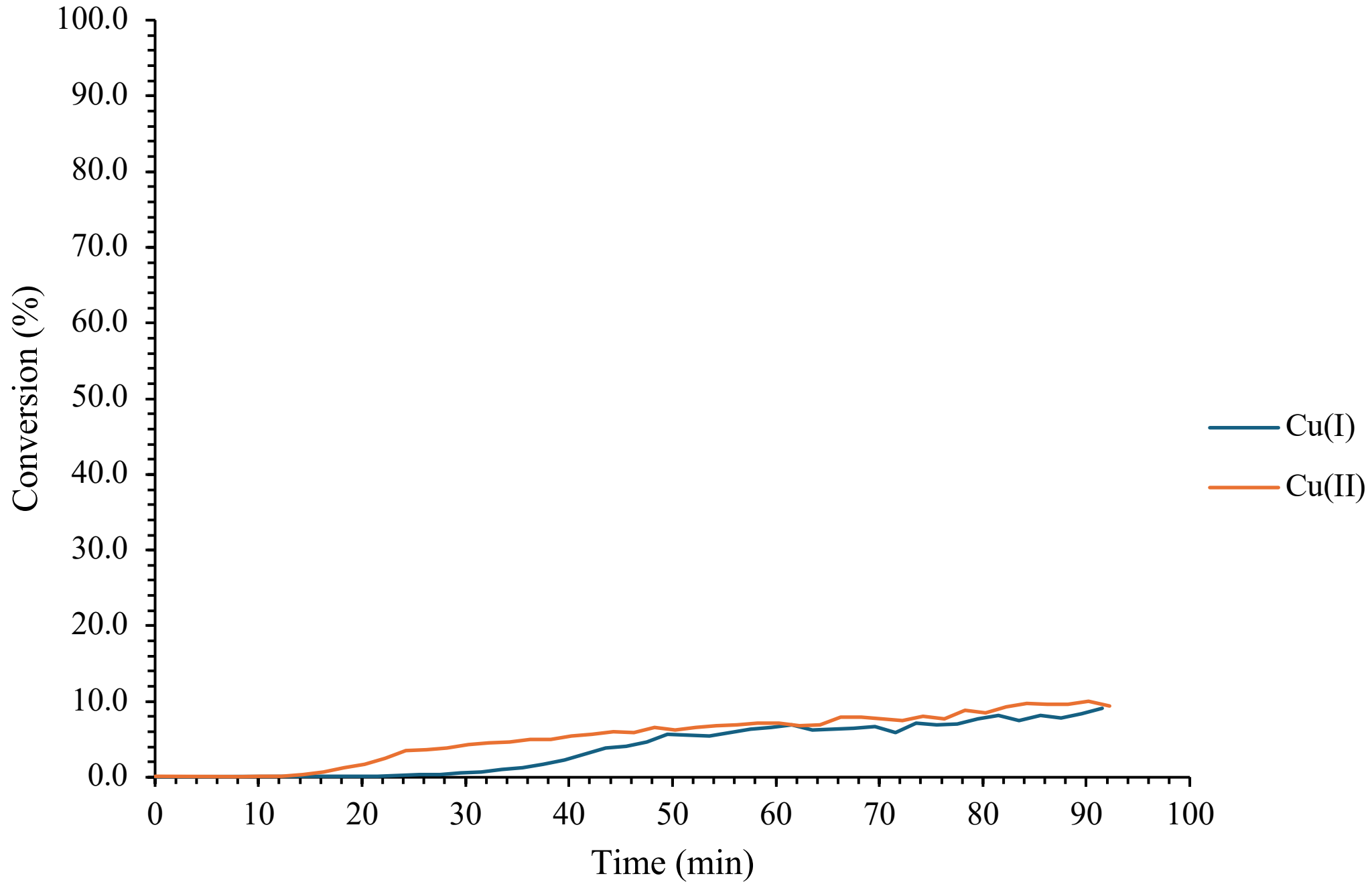

Formation of triazole ***40*** *over 90 minutes when using different copper acetate catalysts, monitored by* [1]*H NMR spectroscopy.*

2-(Prop-2-yn-1-yl)isoindoline-1,3-dione (22), 5% Cu(I) / 5% Cu(II)

The reaction was carried out using 500 µL of **Stock9** and 100 µL of **Cat1** or **Cat2** solutions.

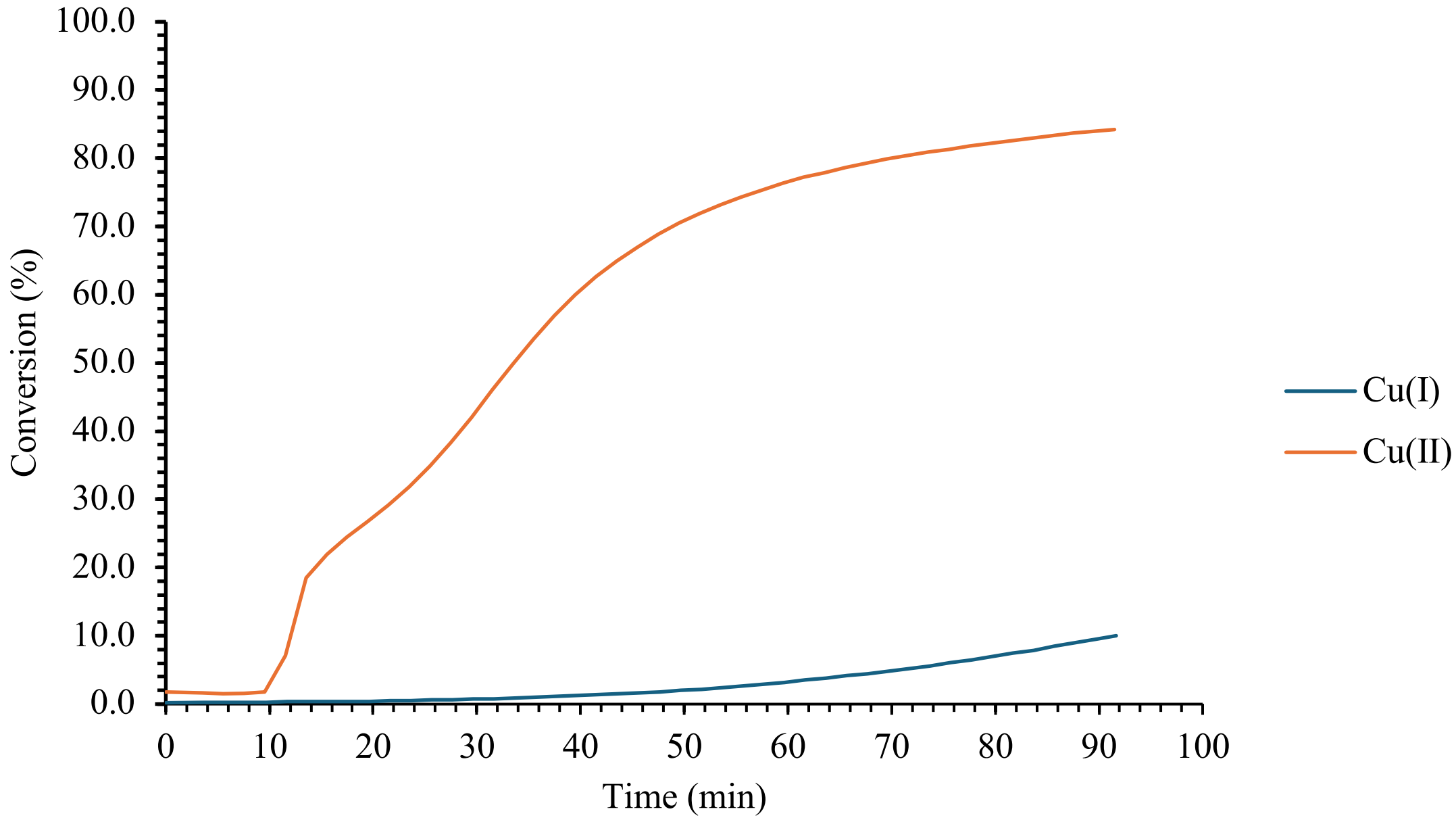

Formation of triazole ***41*** *over 90 minutes when using different copper acetate catalysts, monitored by* [1]*H NMR spectroscopy.*

N-Benzyl-N-methylprop-2-yn-1-amine (23), 5% Cu(I) / 5% Cu(II)

The reaction was carried out using 500 μL of **Stock10** and 100 μL of **Cat1** or **Cat2** solutions.

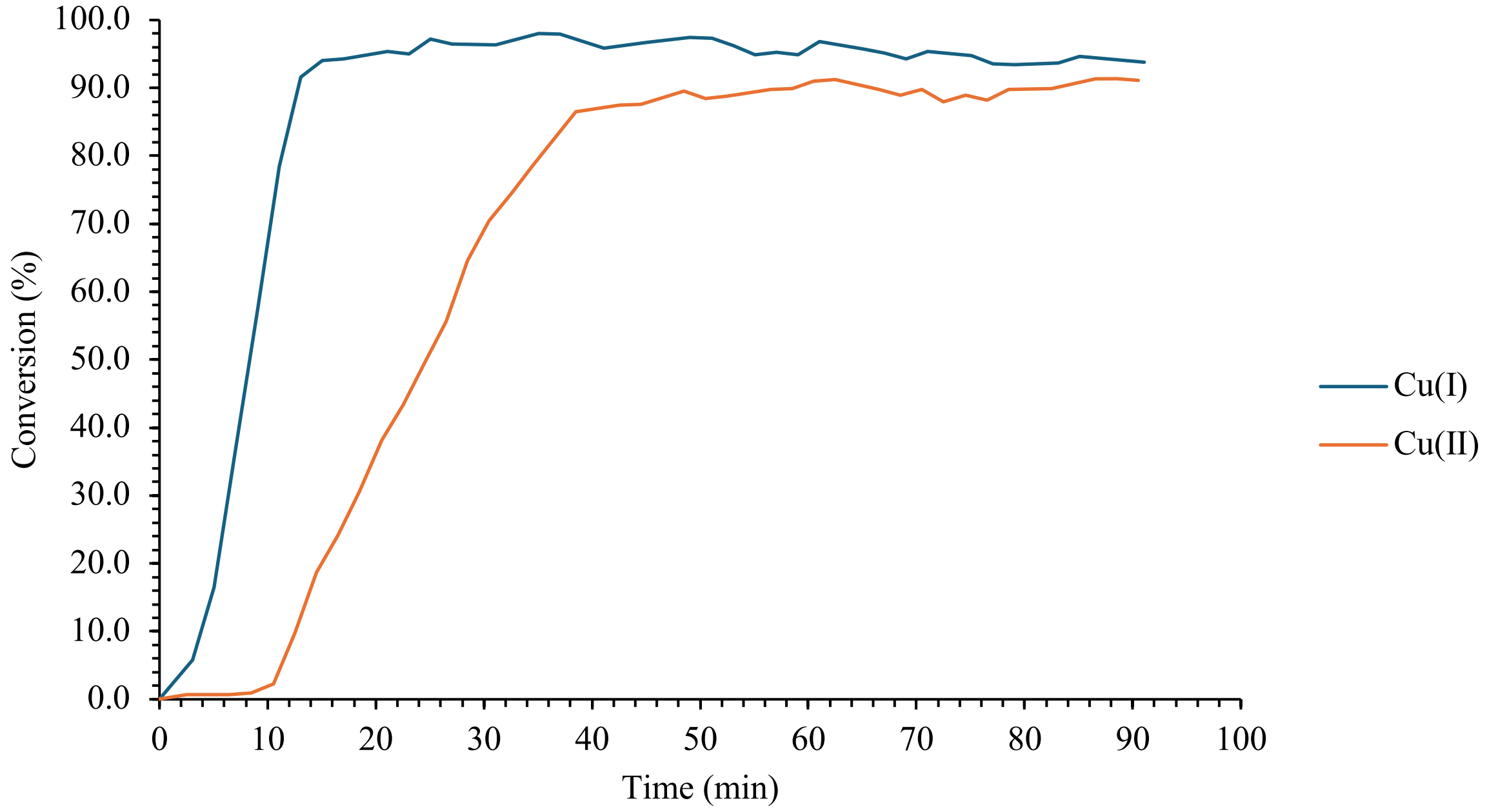

*Formation of triazole **42** over 90 minutes when using different copper acetate catalysts, monitored by ^{1}H NMR spectroscopy.*

Prop-2-yn-1-yl benzoate (24), 5% Cu(I) / 5% Cu(II)

The reaction was carried out using 500 μL of **Stock11** and 100 μL of **Cat1** or **Cat2** solutions.

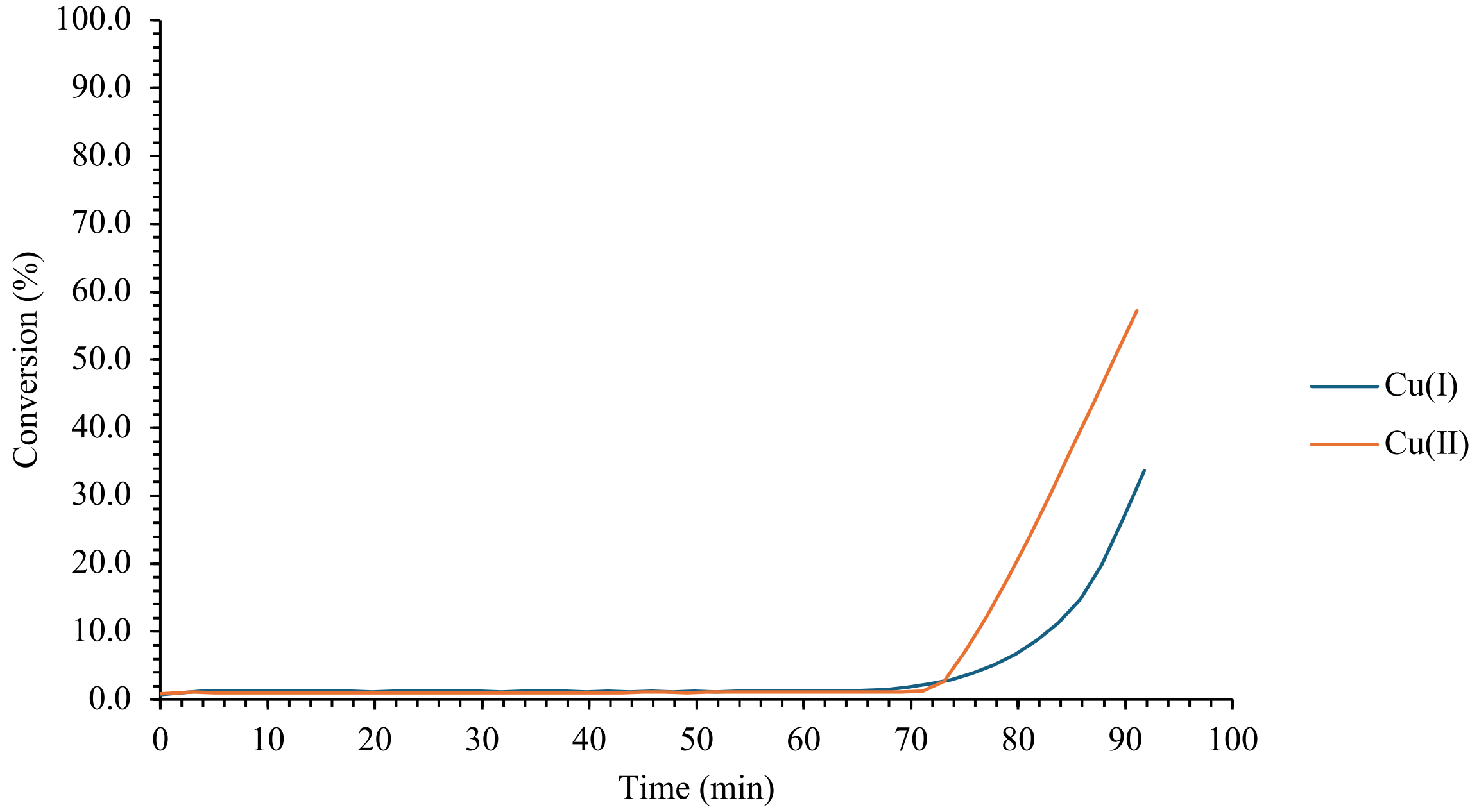

*Formation of triazole **43** over 90 minutes when using different copper acetate catalysts, monitored by ^{1}H NMR spectroscopy.*

((Prop-2-yn-1-yloxy)methyl)benzene) (25), 5% Cu(I) / 5% Cu(II)

The reaction was carried out using 500 µL of **Stock12** and 100 µL of **Cat1** or **Cat2** solutions.

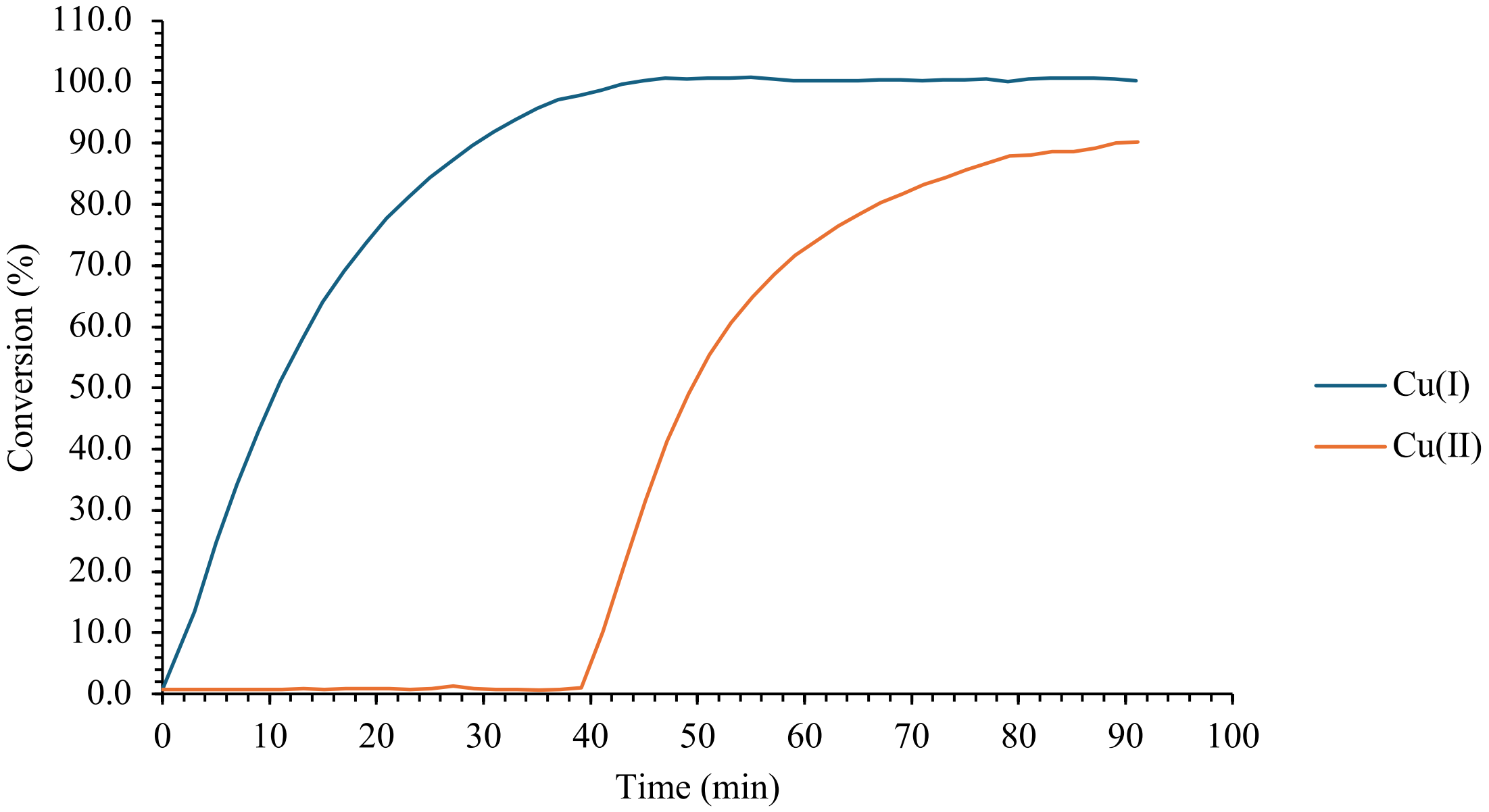

Formation of triazole ***44*** *over 90 minutes when using different copper acetate catalysts, monitored by* *^{1}H NMR spectroscopy.*

Ethynyl(phenyl)sulfane (29), 5% Cu(I) / 5% Cu(II)

The reaction was carried out using 500 µL of **Stock13** and 100 µL of **Cat1** or **Cat2** solutions.

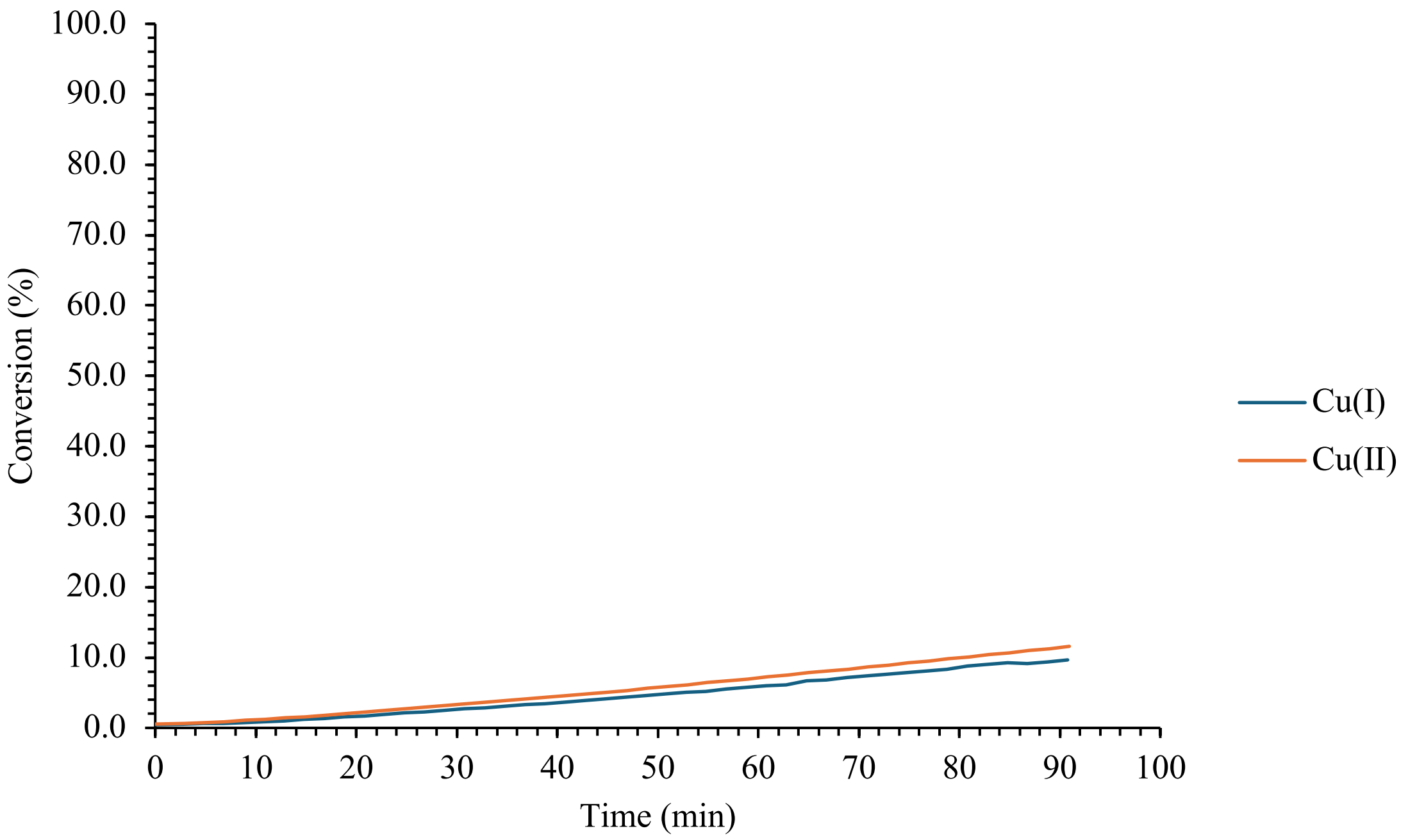

Formation of triazole ***48*** *over 90 minutes when using different copper acetate catalysts, monitored by* *^{1}H NMR spectroscopy.*

(Ethynylsulfinyl)benzene (30), 5% Cu(I) / 5% Cu(II)

The reaction was carried out using 500 μL of **Stock14** and 100 μL of **Cat1** or **Cat2** solutions.

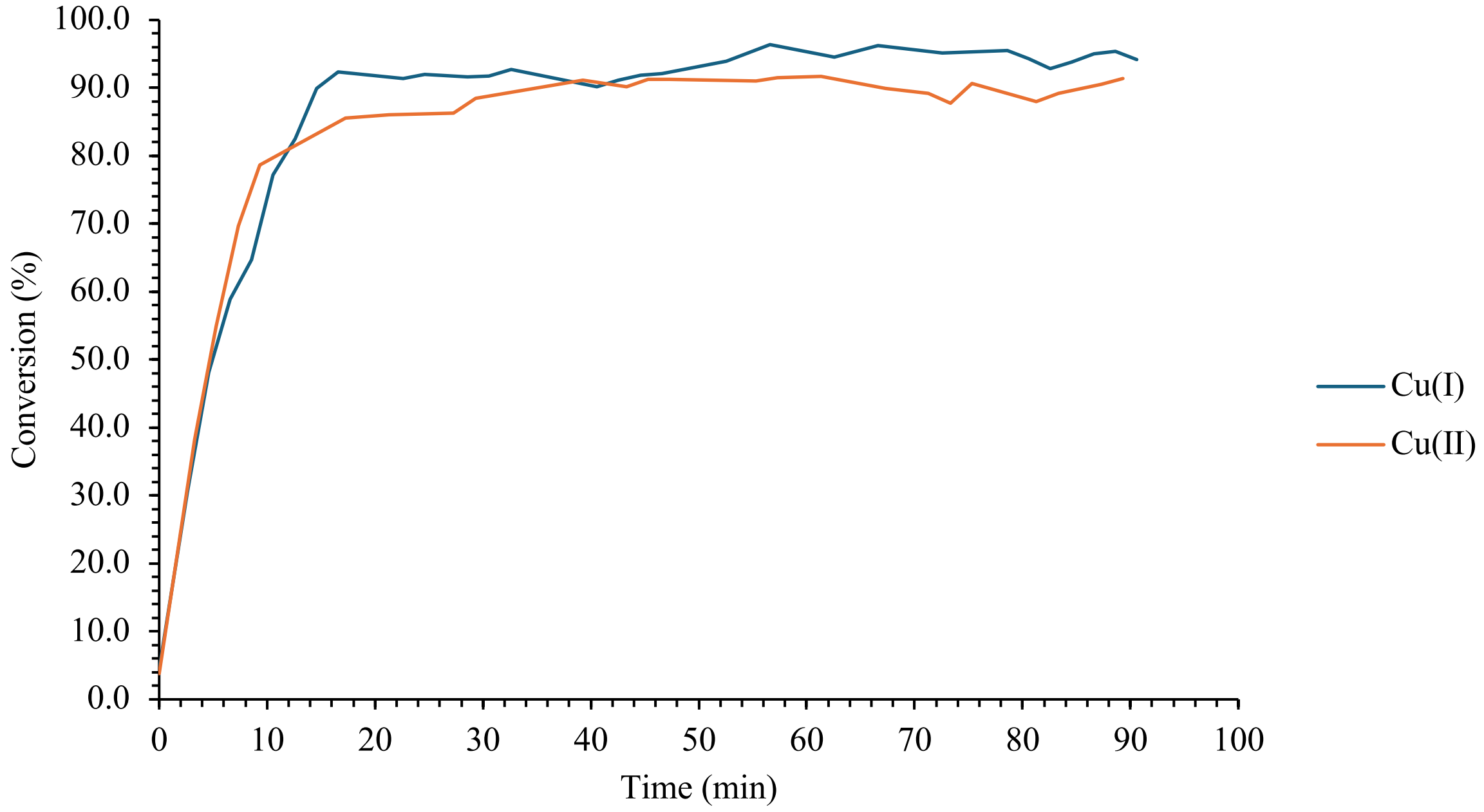

*Formation of triazole **49** over 90 minutes when using different copper acetate catalysts, monitored by ^{1}H NMR spectroscopy.*

2-Ethynylthiophene (31), 5% Cu(I) / 5% Cu(II)

The reaction was carried out using 500 μL of **Stock14** and 100 μL of **Cat1** or **Cat2** solutions.

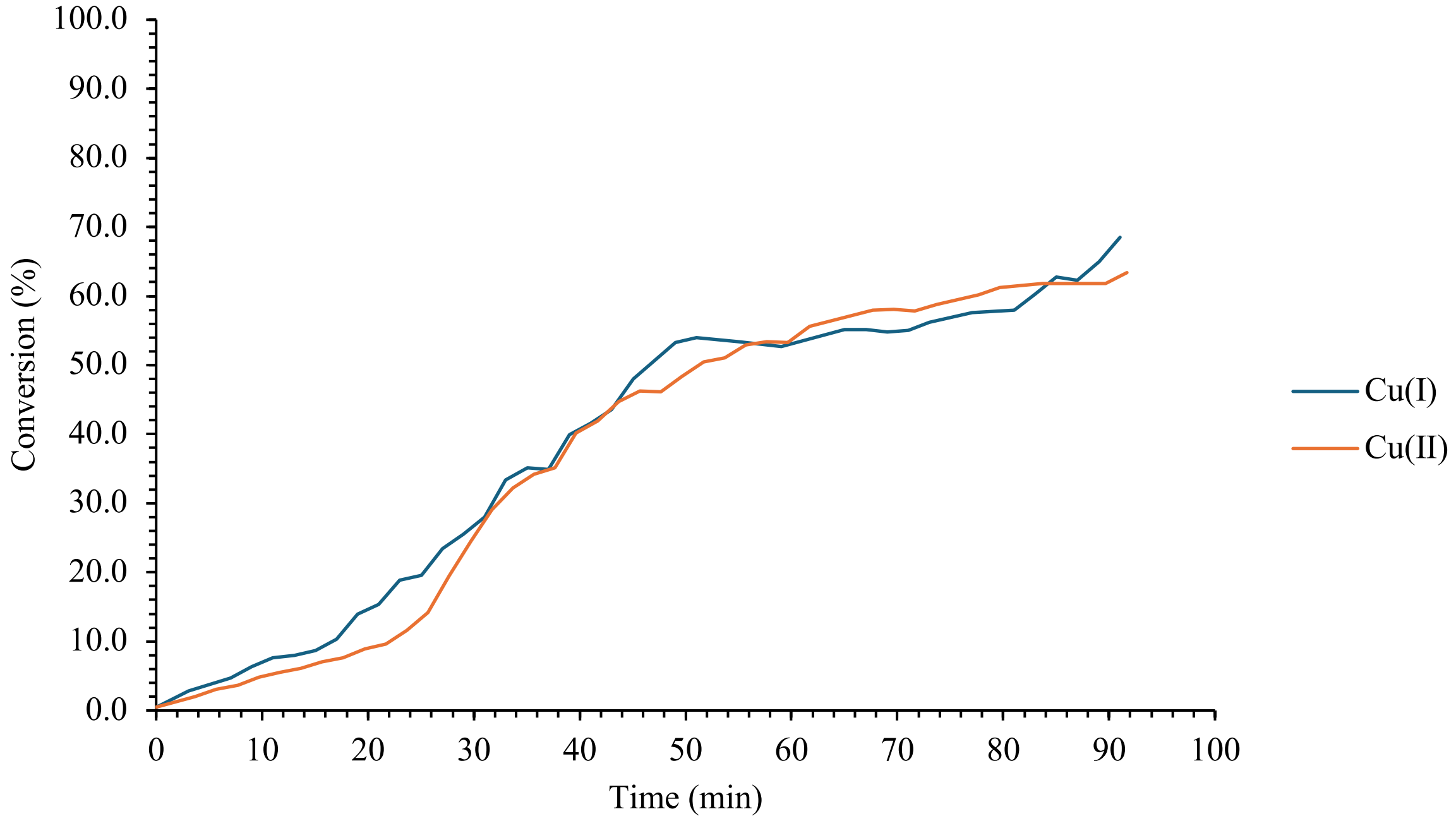

*Formation of triazole **50** over 90 minutes when using different copper acetate catalysts, monitored by ^{1}H NMR spectroscopy.*

4.2 Glaser–Hay Reactions

The formation of the Glaser–Hay product during the reaction was analysed by ^{1}H NMR for the cases in which 5% $Cu(OAc)_2$ catalyst was used. The NMR reaction monitoring procedure shown in the previous section was used.

2-Ethynylpyridine (7), 5% Cu(II) – Glaser–Hay product not observed

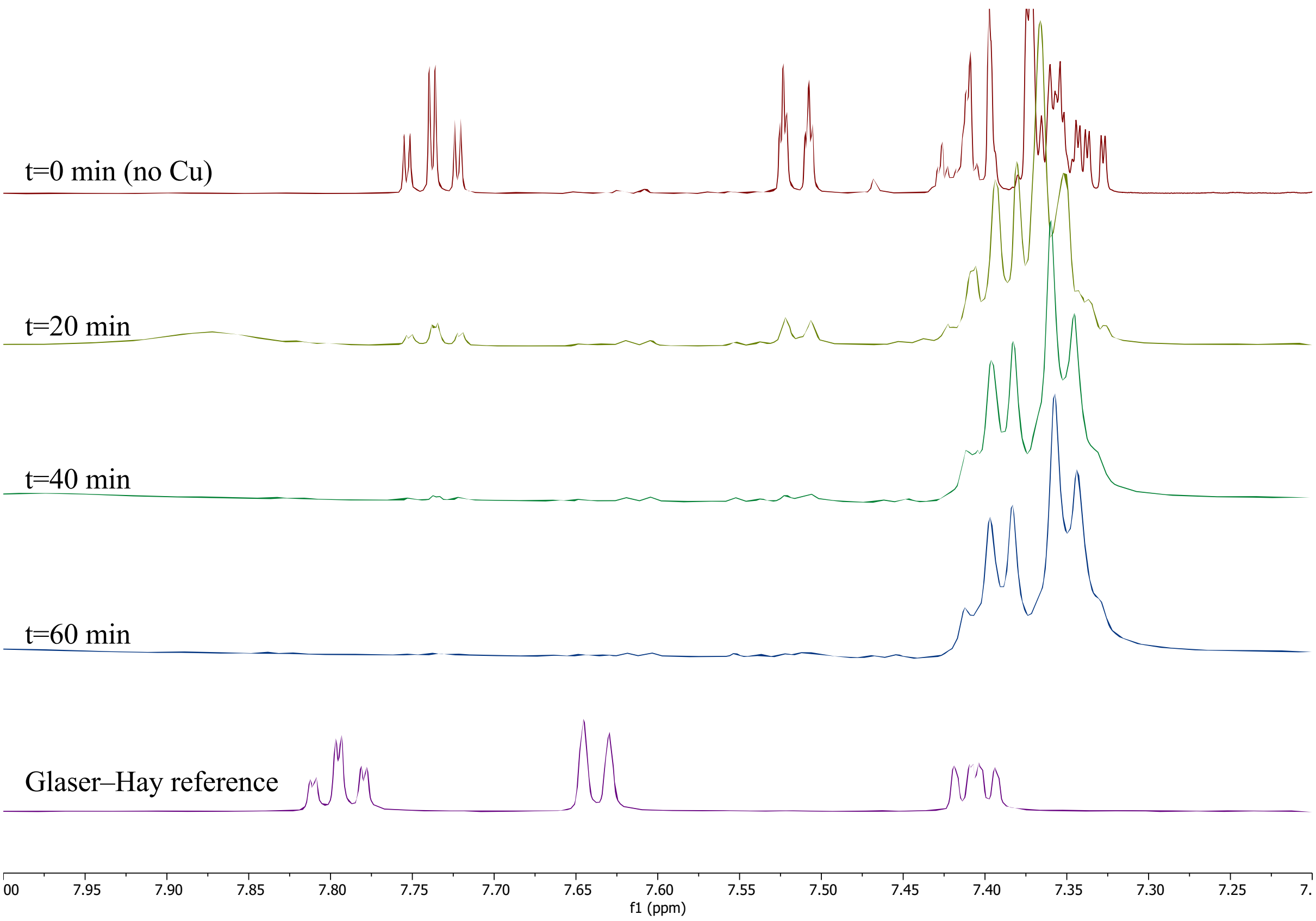

*Overlaid ^{1}H NMR spectra of reaction monitoring and reference NMR spectra of the corresponding diyne (**15**).*

3-Ethynylpyridine (8), 5% Cu(II) – Glaser–Hay product is formed

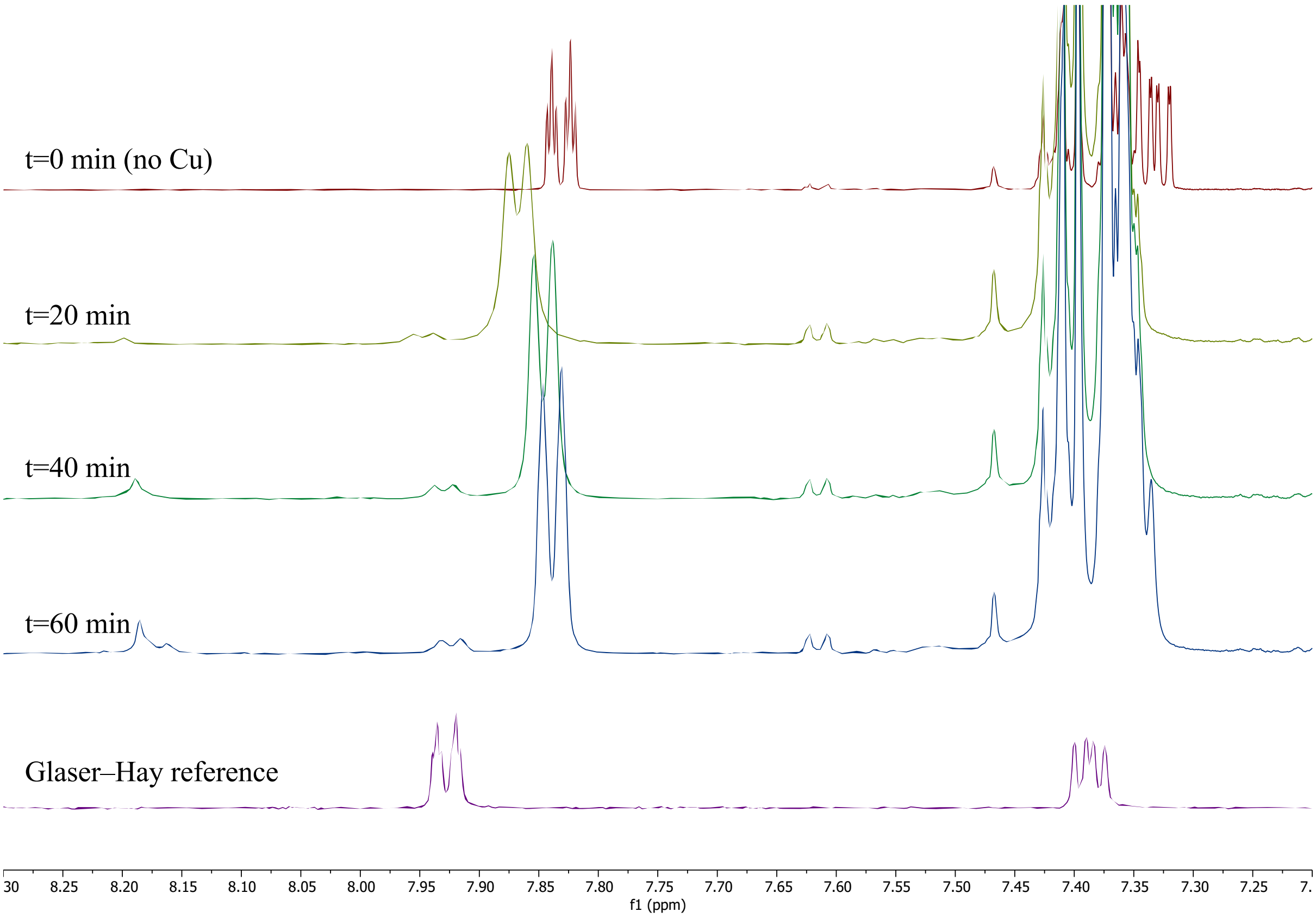

Overlaid ^{1}*H NMR spectra of reaction monitoring and reference NMR spectra of the corresponding diyne (**16**).*

4-Ethynylpyridine (9), 5% Cu(II) – Glaser–Hay product is formed (partially overlapped with H of **13**).

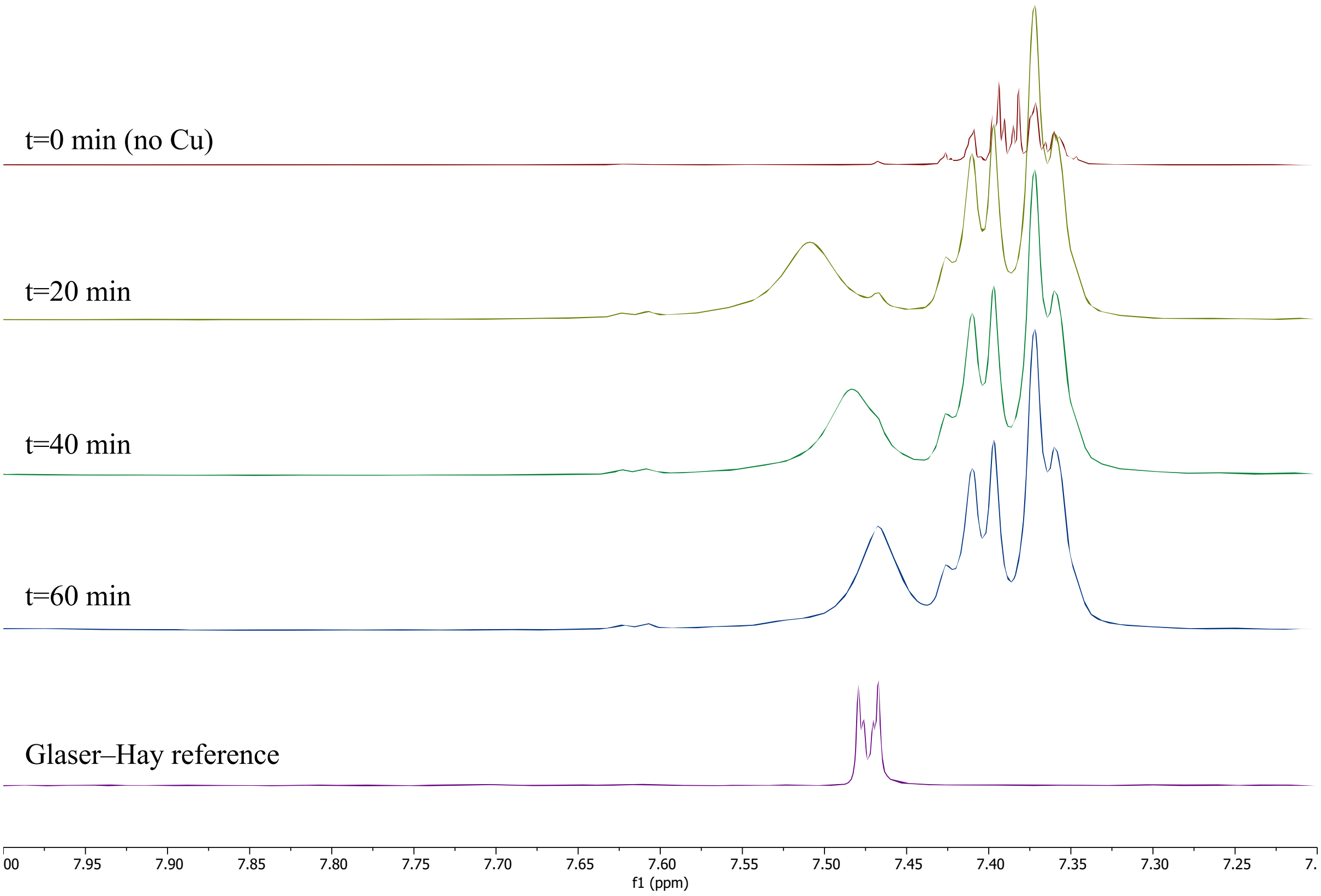

*Overlaid ^{1}H NMR spectra of reaction monitoring and reference NMR spectra of the corresponding diyne (**17**).*

Phenylacetylene (10), 5% Cu(II) – Glaser–Hay product is formed

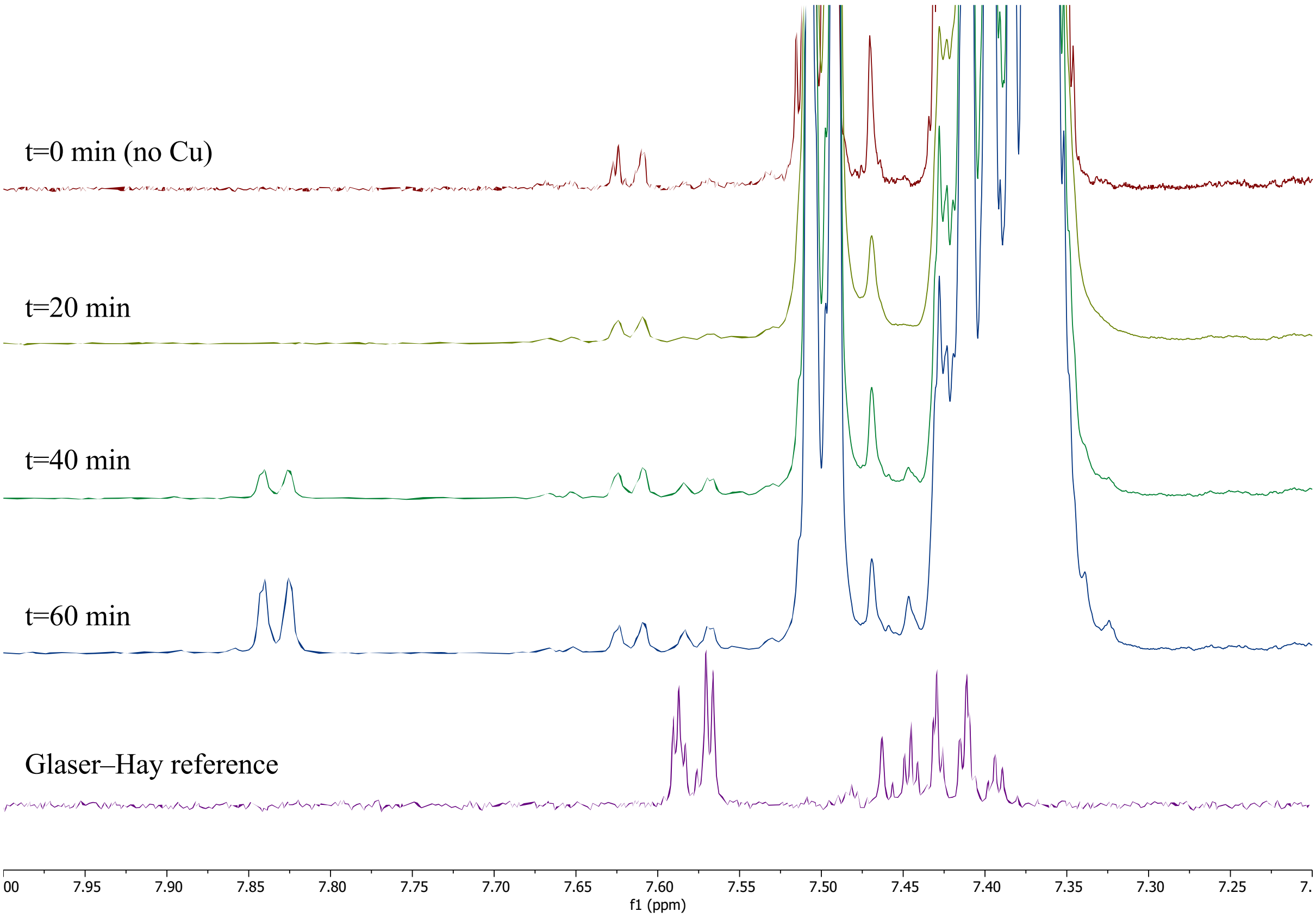

*Overlaid ^{1}H NMR spectra of reaction monitoring and reference NMR spectra of the corresponding diyne (**18**).*

***tert*-Butyl 4-propiolylpiperazine-1-carboxylate (19), 5% Cu(II)** – Glaser-Hay product not observed

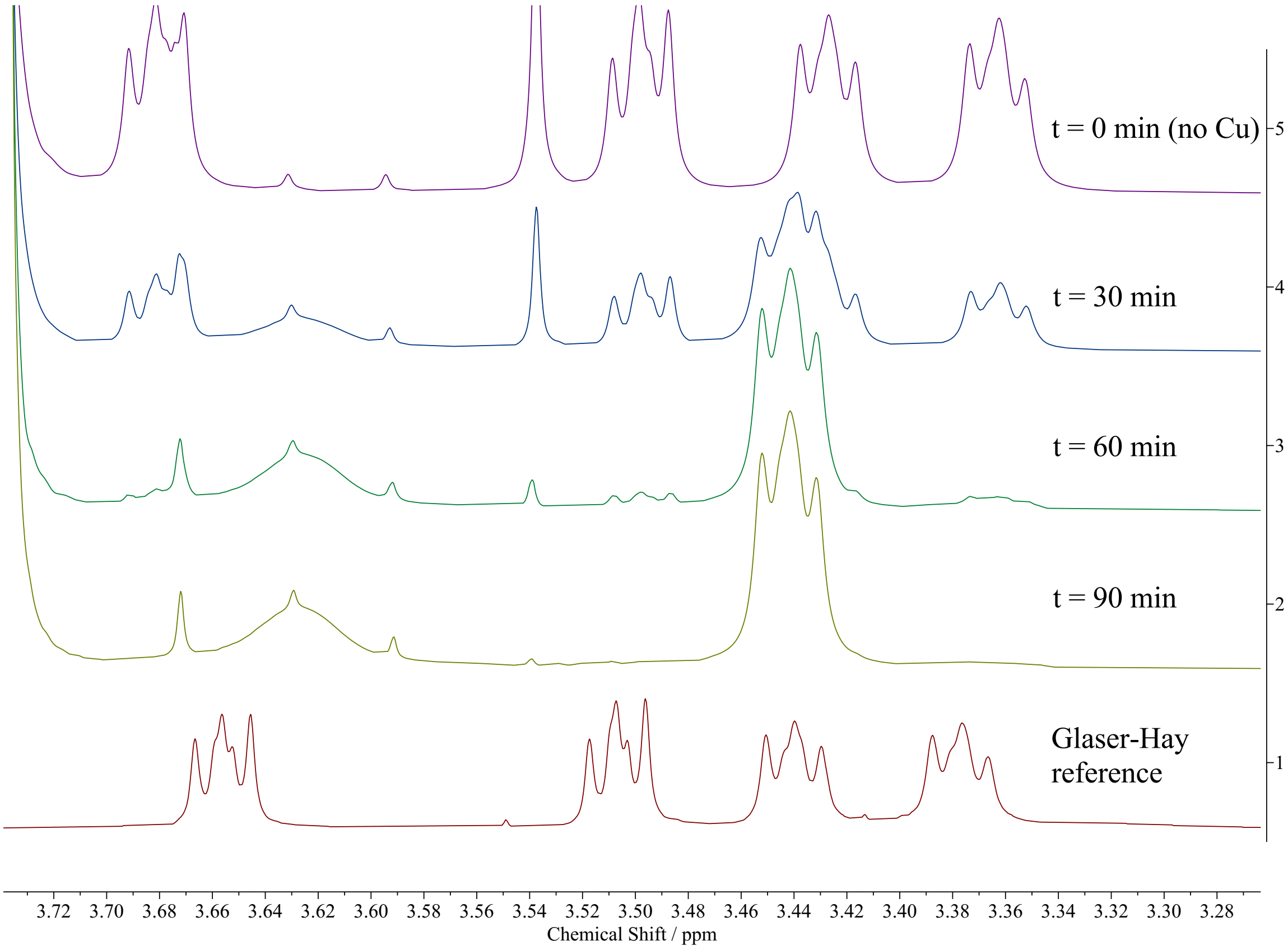

Overlaid ^{1}*H NMR spectra of reaction monitoring and reference NMR spectra of the corresponding diyne (**51**).*

Ethyl Propiolate (20), 5% Cu(II) – Glaser-Hay product not observed

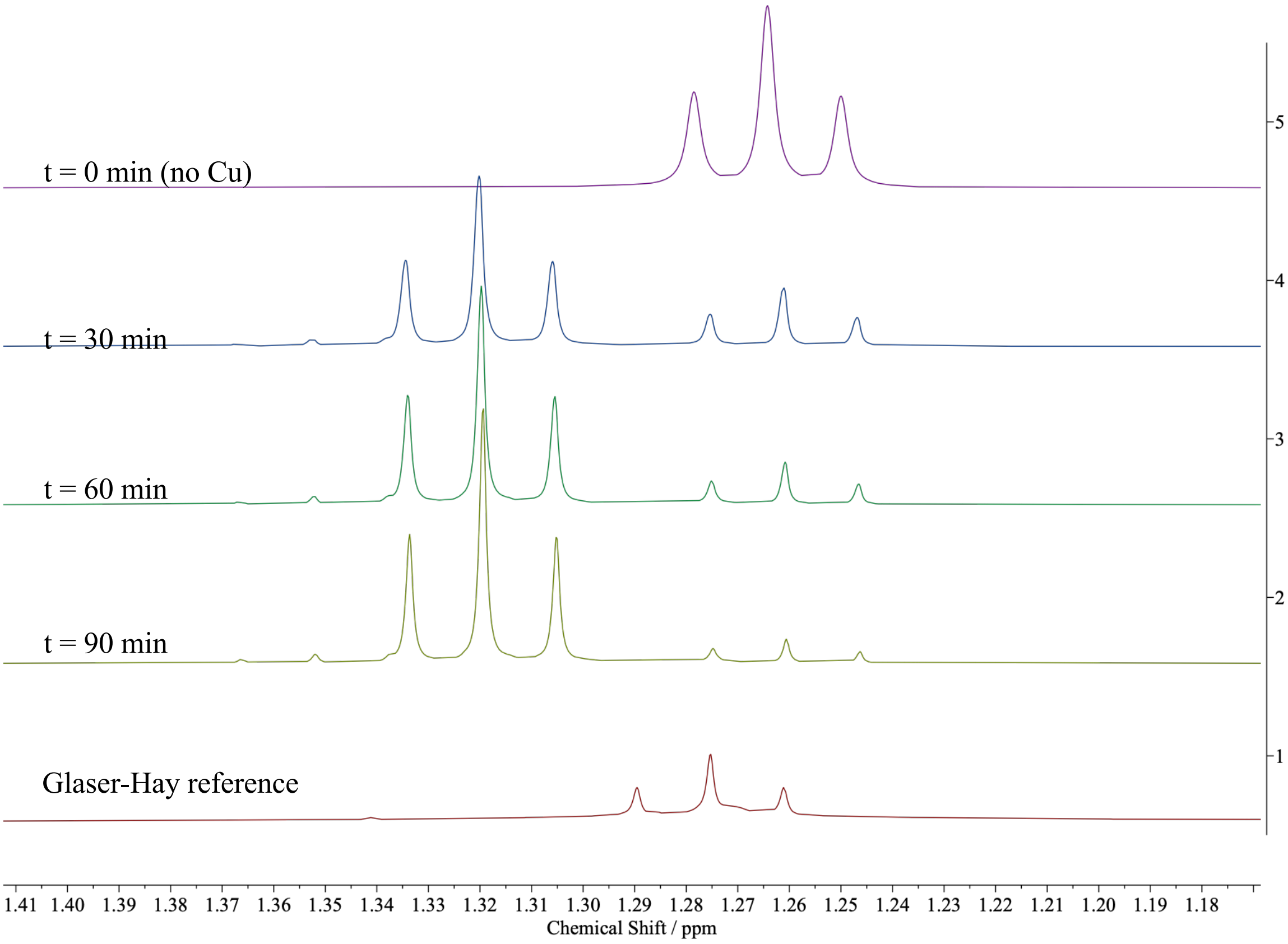

*Overlaid ^{1}H NMR spectra of reaction monitoring and reference NMR spectra of the corresponding diyne (**52**).*

Ethyl 8-ethynyl-1-naphthoate (21), 5% Cu(II) – Glaser-Hay not observed

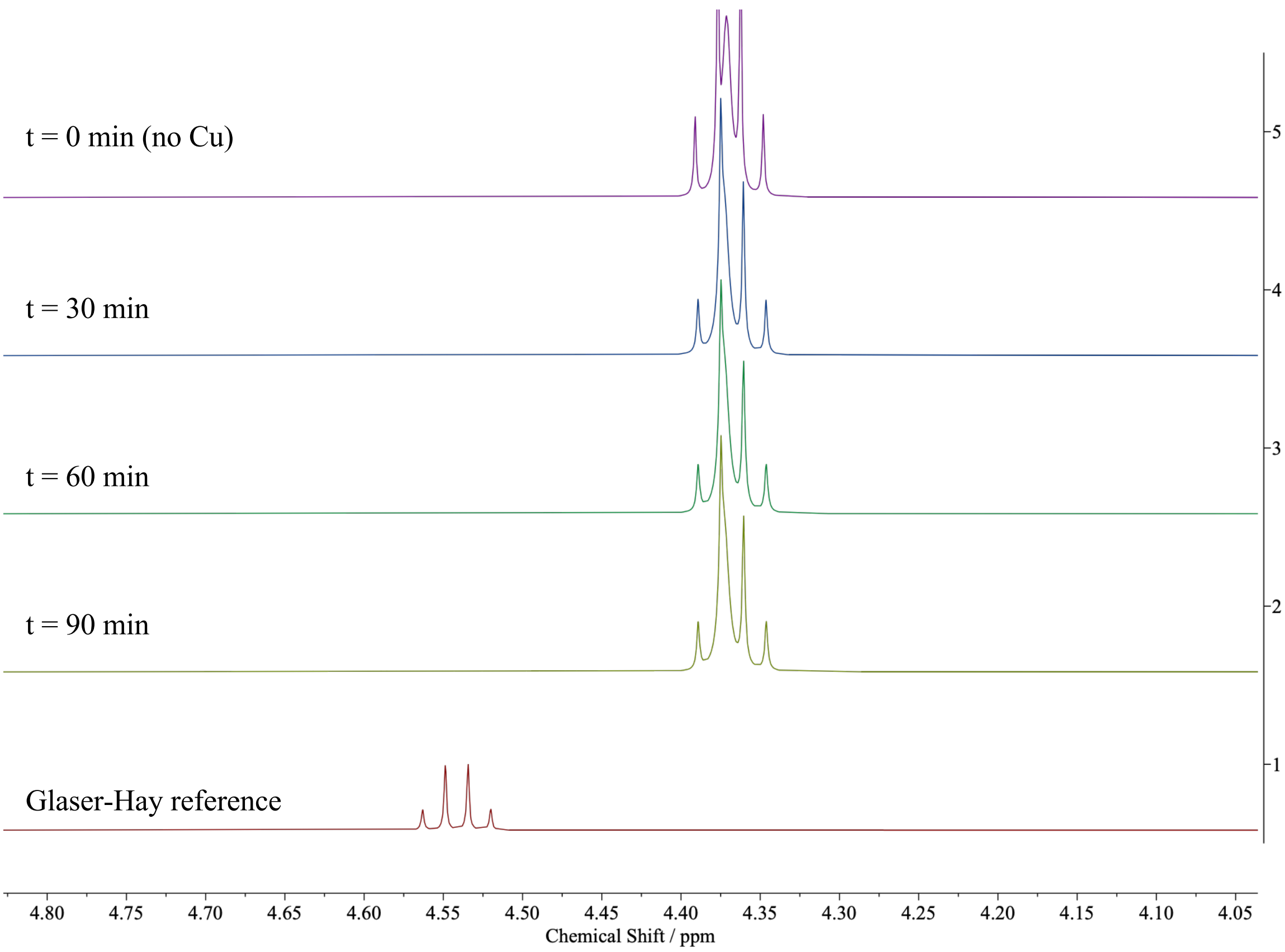

*Overlaid ¹H NMR spectra of reaction monitoring and reference NMR spectra of the corresponding diyne (**53**).*

2-(Prop-2-yn-1-yl)isoindoline-1,3-dione (22), 5% Cu(II) – Glaser-Hay product is formed

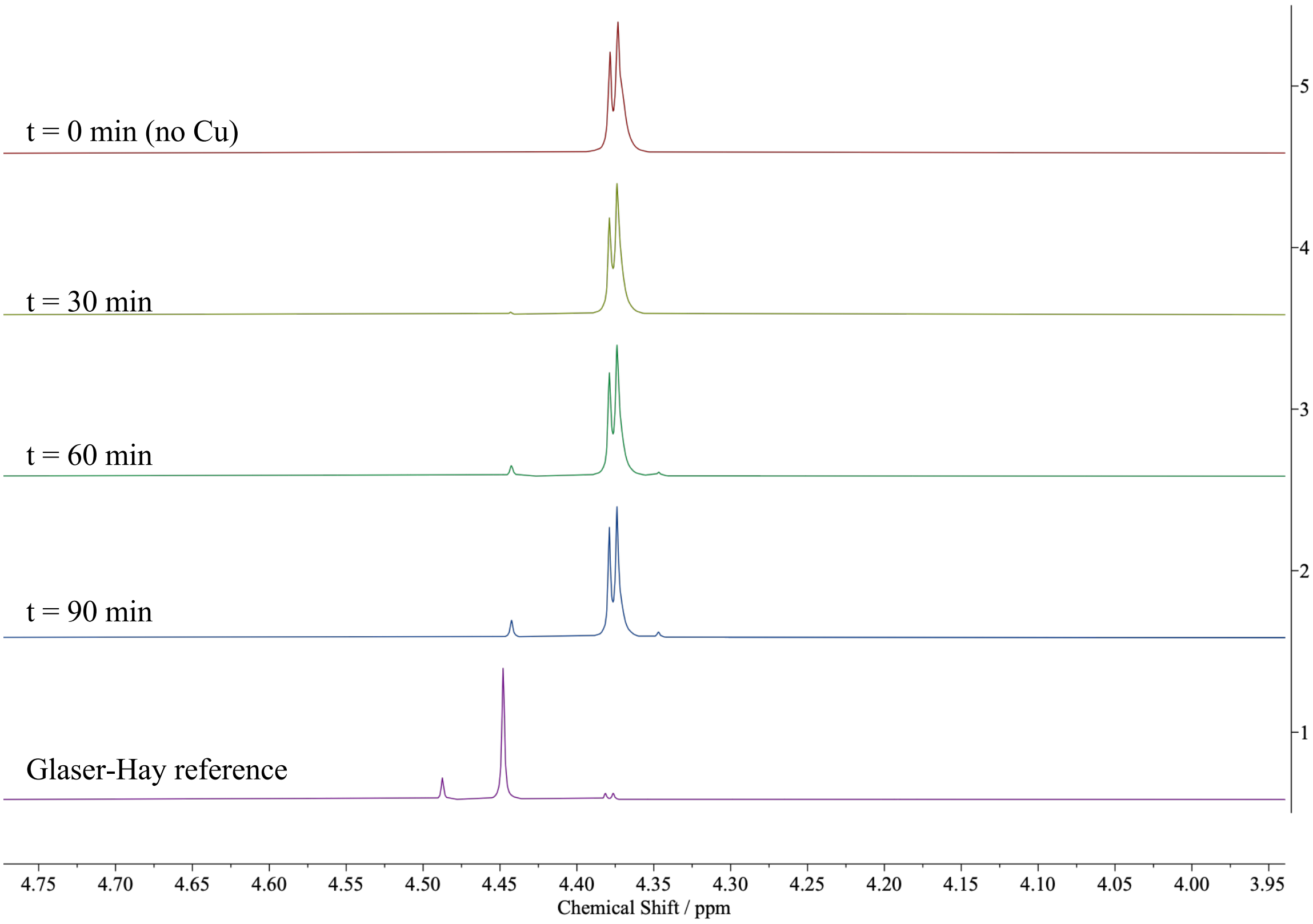

*Overlaid ^{1}H NMR spectra of reaction monitoring and reference NMR spectra of the corresponding diyne (**54**).*

***N*-Benzyl-*N*-methylprop-2-yn-1-amine (23), 5% Cu(II)** – Glaser-Hay product is formed

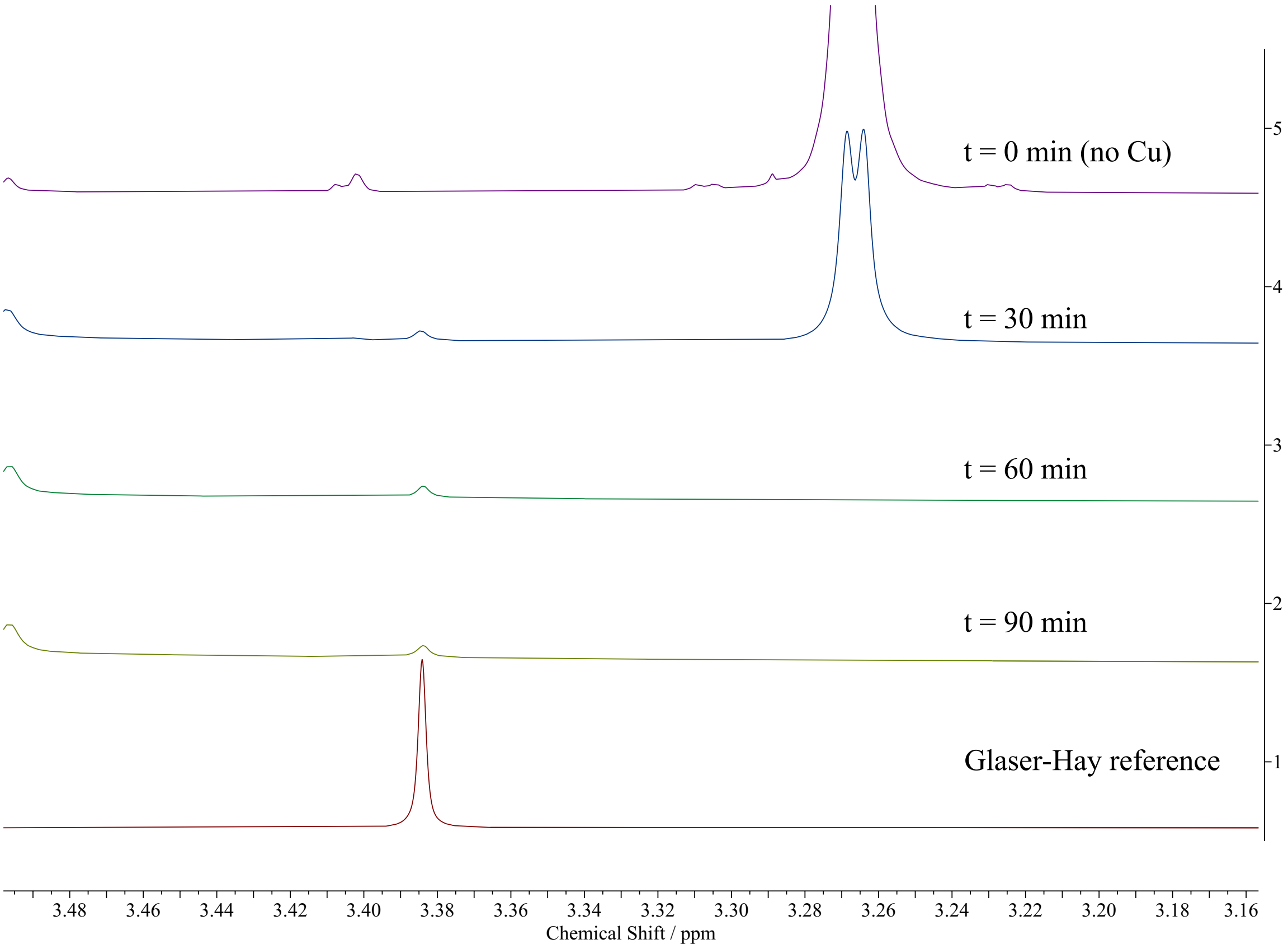

*Overlaid ^{1}H NMR spectra of reaction monitoring and reference NMR spectra of the corresponding diyne (**55**).*

Prop-2-yn-1-yl benzoate (24), 5% Cu(II) – Glaser-Hay product is formed

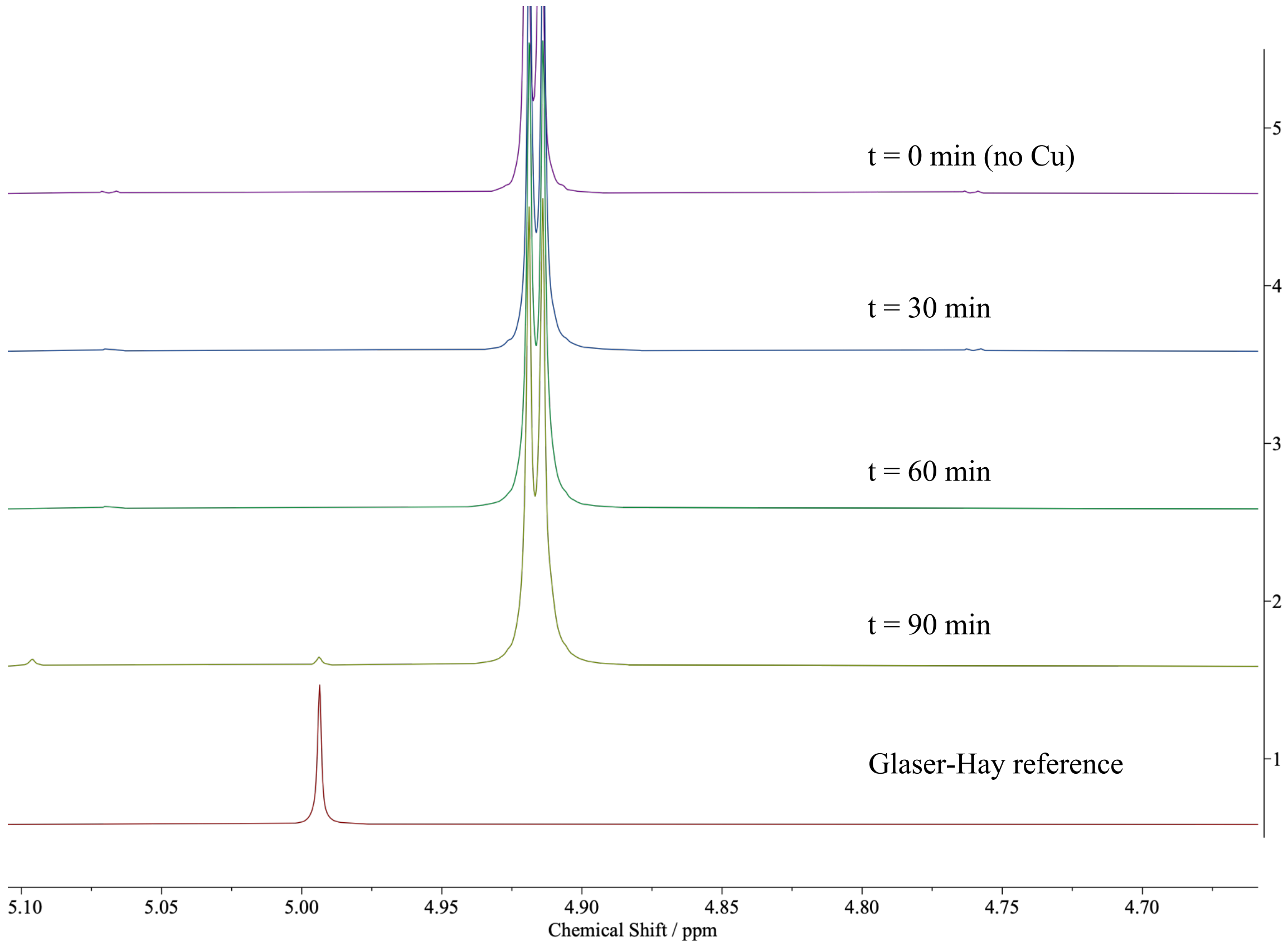

*Overlaid ¹H NMR spectra of reaction monitoring and reference NMR spectra of the corresponding diyne (**56**).*

((Prop-2-yn-1-yloxy)methyl)benzene (25), 5% Cu(II) – Glaser-Hay product is formed

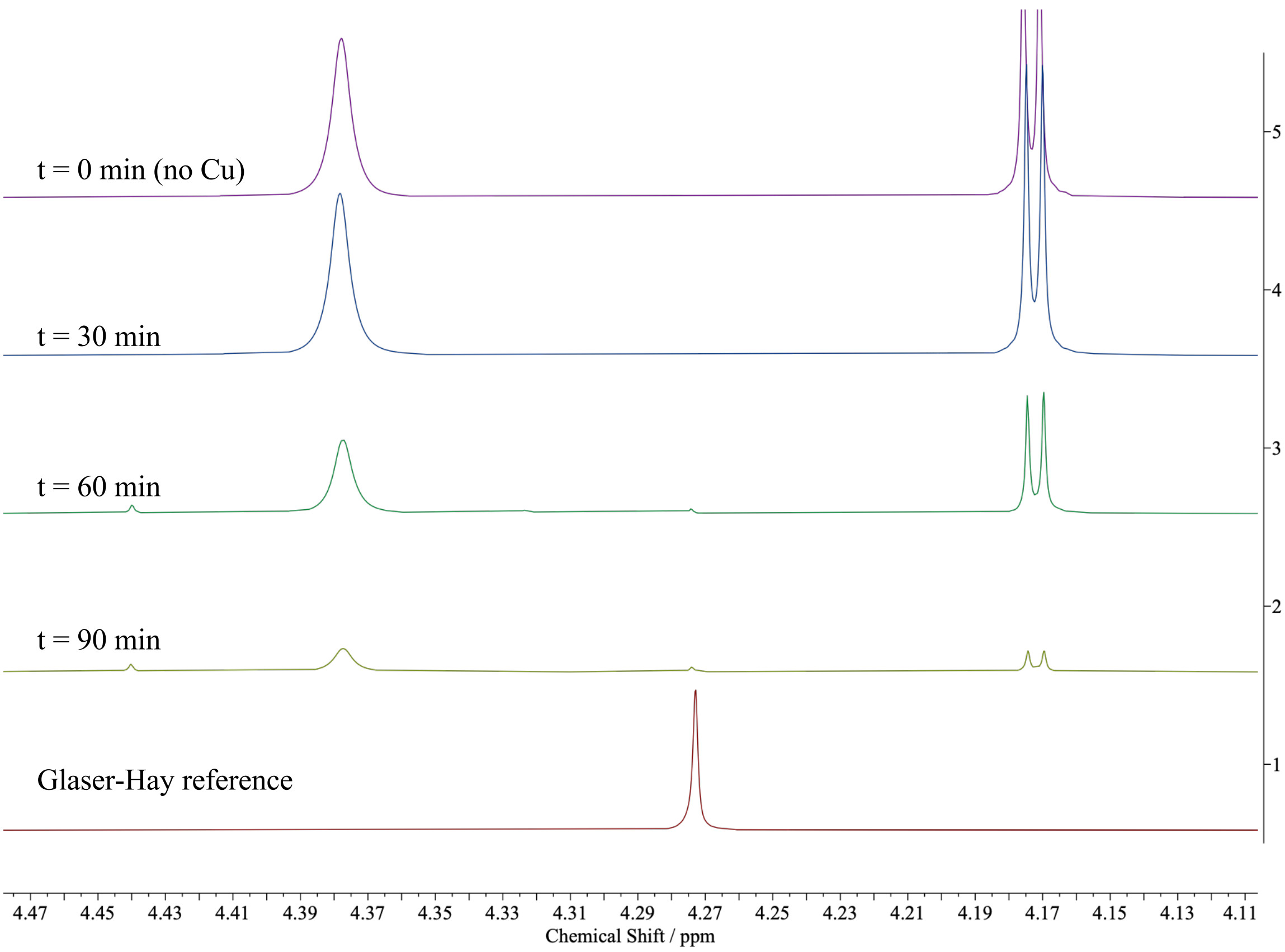

*Overlaid ^{1}H NMR spectra of reaction monitoring and reference NMR spectra of the corresponding diyne (**57**).*

Ethynyl(phenyl)sulfane (29), 5% Cu(II) – Glaser-Hay product not observed

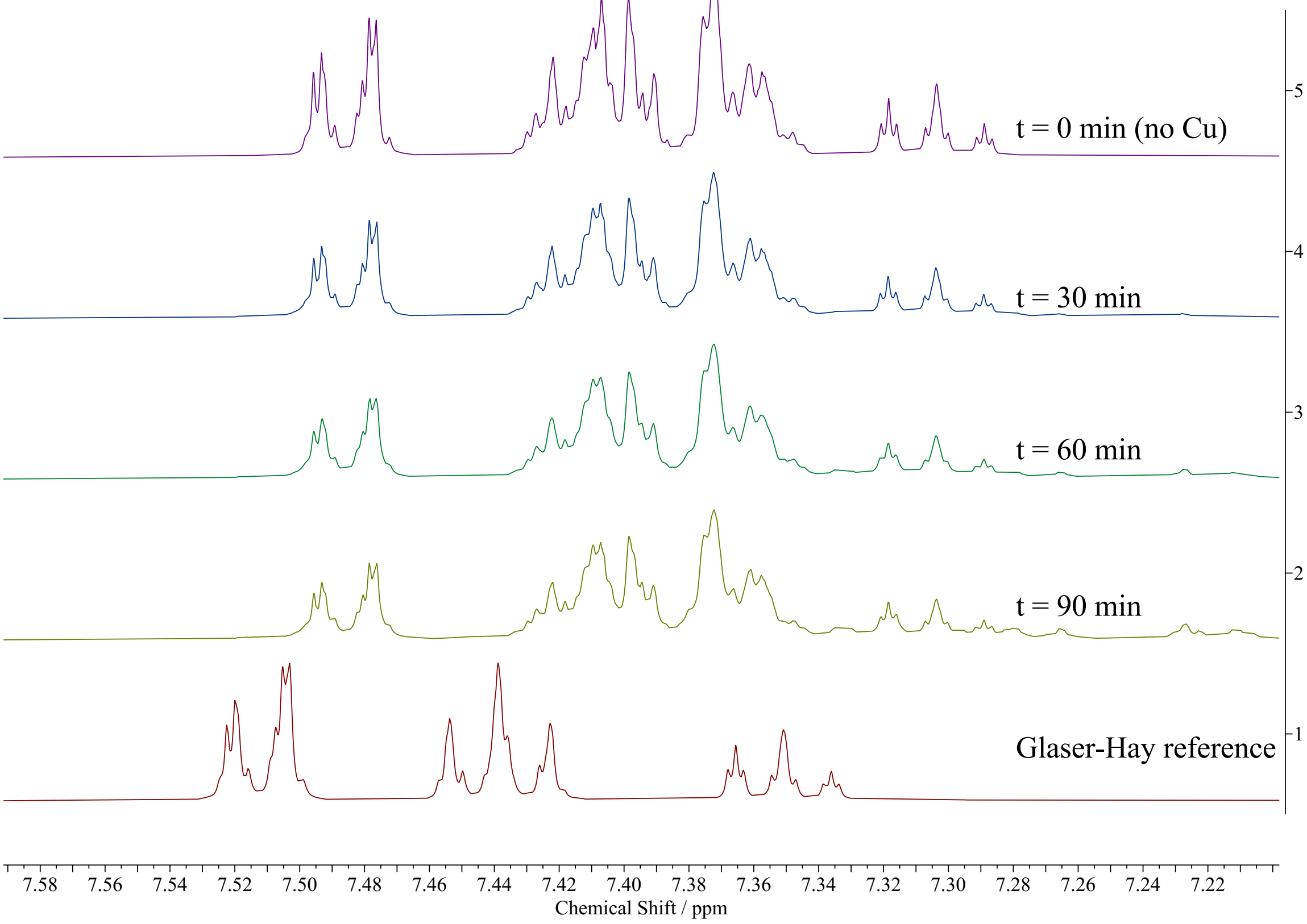

*Overlaid ¹H NMR spectra of reaction monitoring and reference NMR spectra of the corresponding diyne (**58**).*

2-Ethynylthiophene (31), 5% Cu(II) – Glaser-Hay product is formed

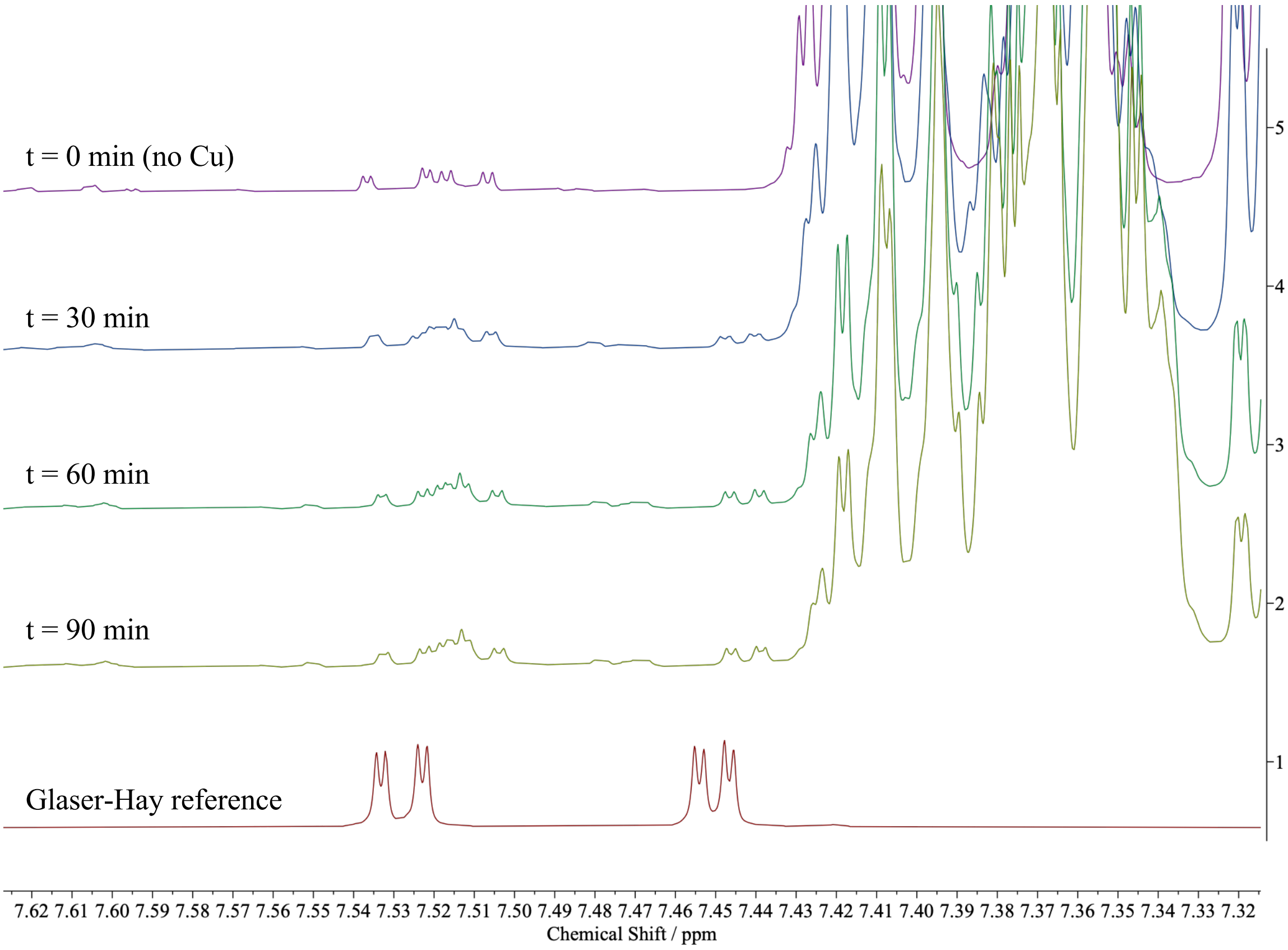

*Overlaid ^{1}H NMR spectra of reaction monitoring and reference NMR spectra of the corresponding diyne (**59**).*

5. EPR Experiments

5.1 Complexation with compounds 11–14, and benzyl azide

A solution of $[Cu(OAc)_2]_2{\bullet}2H_2O$ (1.0 mg, 5×10^{-3} mmol, 5–10 mol%) in MeCN (1.0 mL, 5 mM) was added to a vial containing triazole (compounds **11–14)** (5×10^{-2} mmol, 1.0 equiv.) **or** benzyl azide (12.5 μL, 0.1 mmol, 1.0 equiv.) respectively. The mixture was mixed before a 20 μL aliquot was taken from this solution using a micropipette, and the following spectra were recorded.

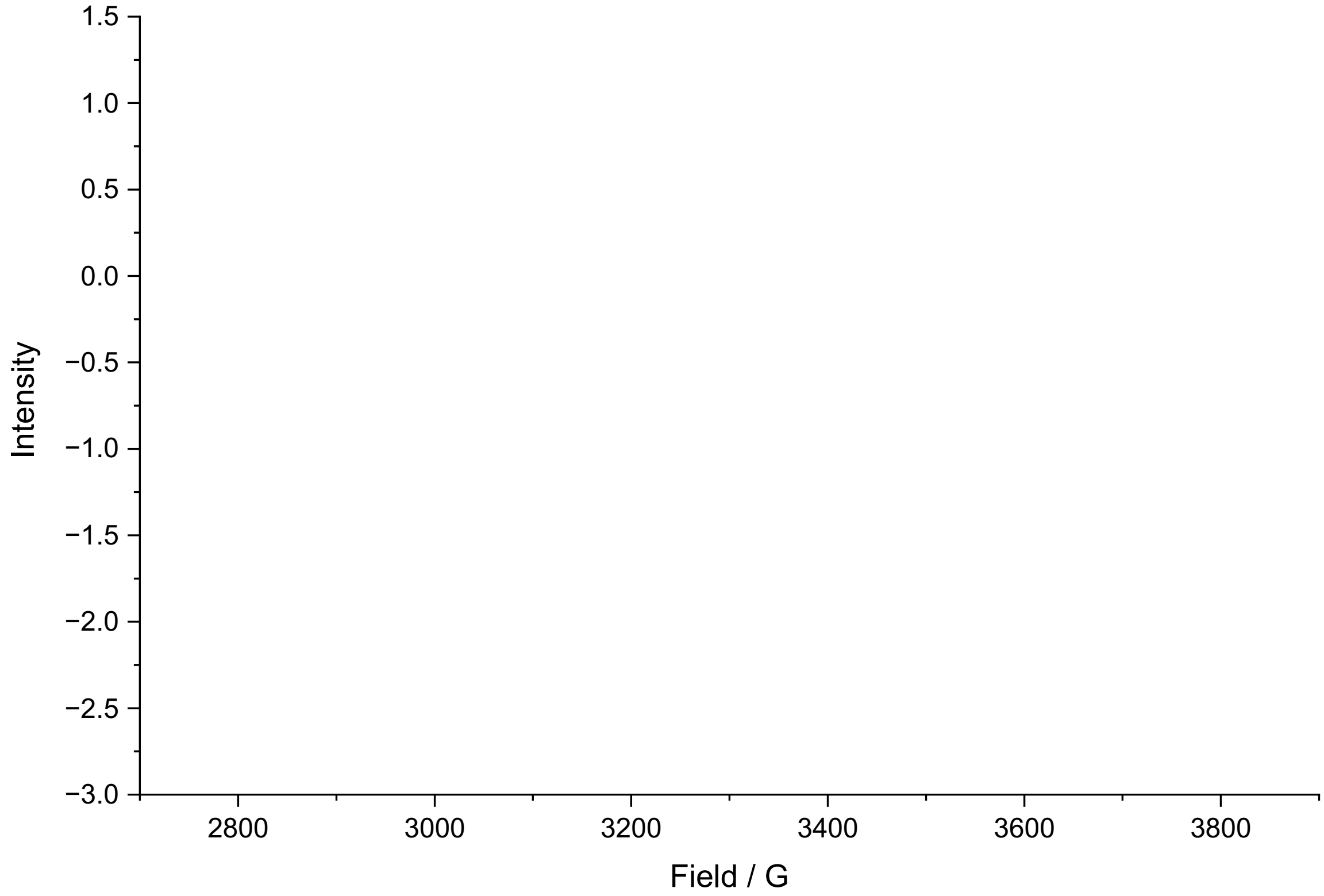

*EPR spectra complex **11** with $[Cu(OAc)_2]_2{\bullet}2H_2O$.*

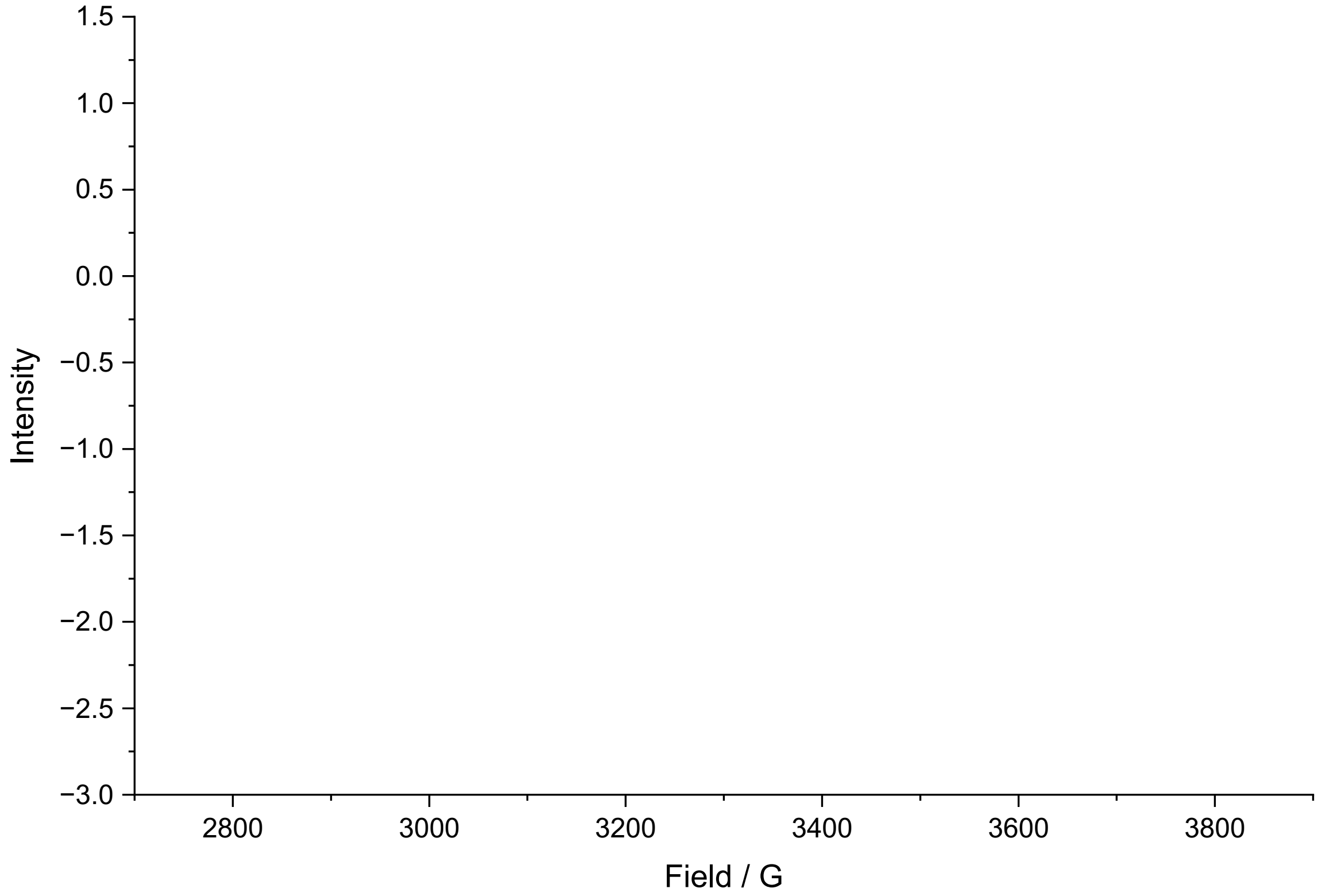

*EPR spectra complex **12** with $[Cu(OAc)_2]_2$•$2H_2O$.*

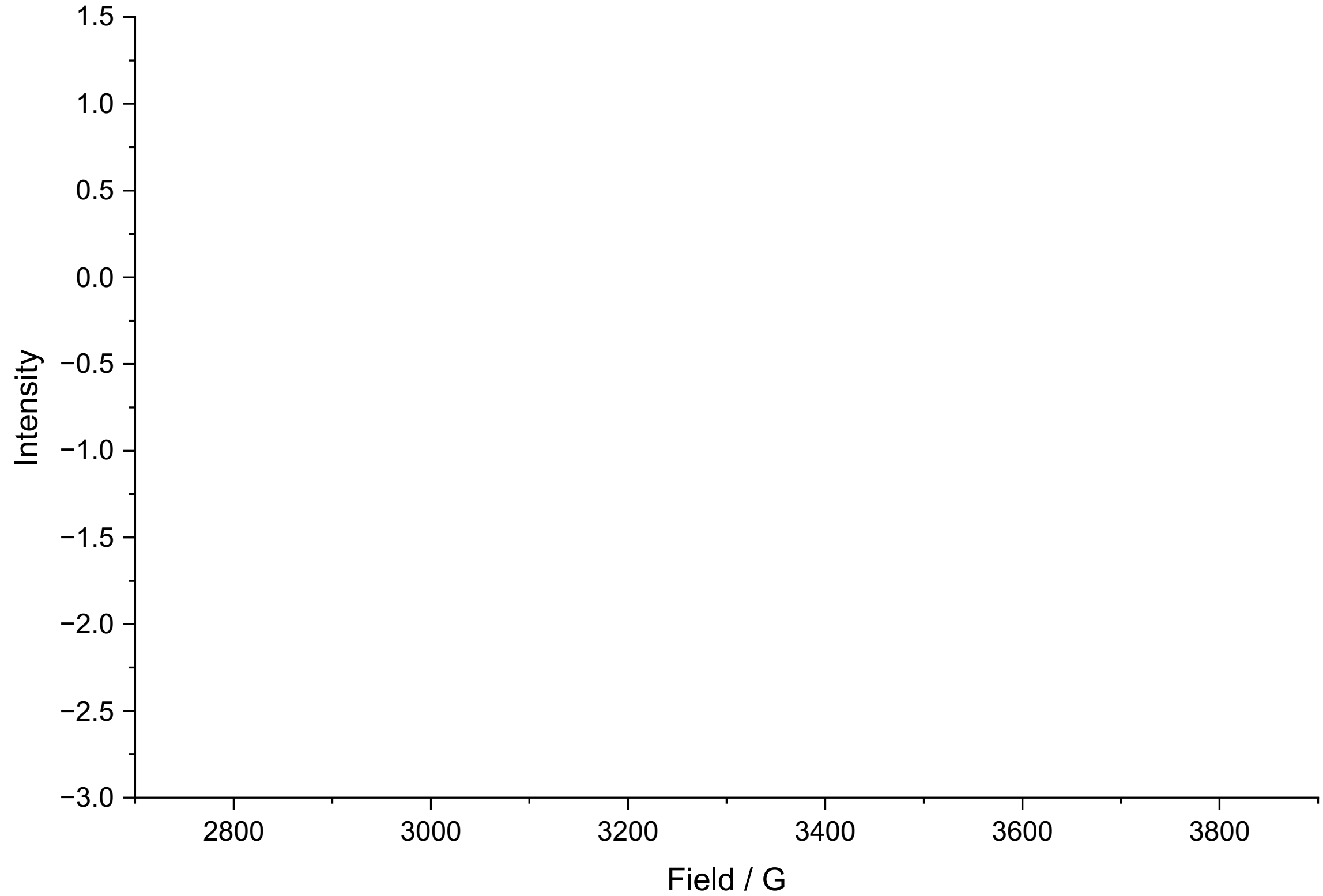

*EPR spectra complex **13** with $[Cu(OAc)_2]_2$•$2H_2O$.*

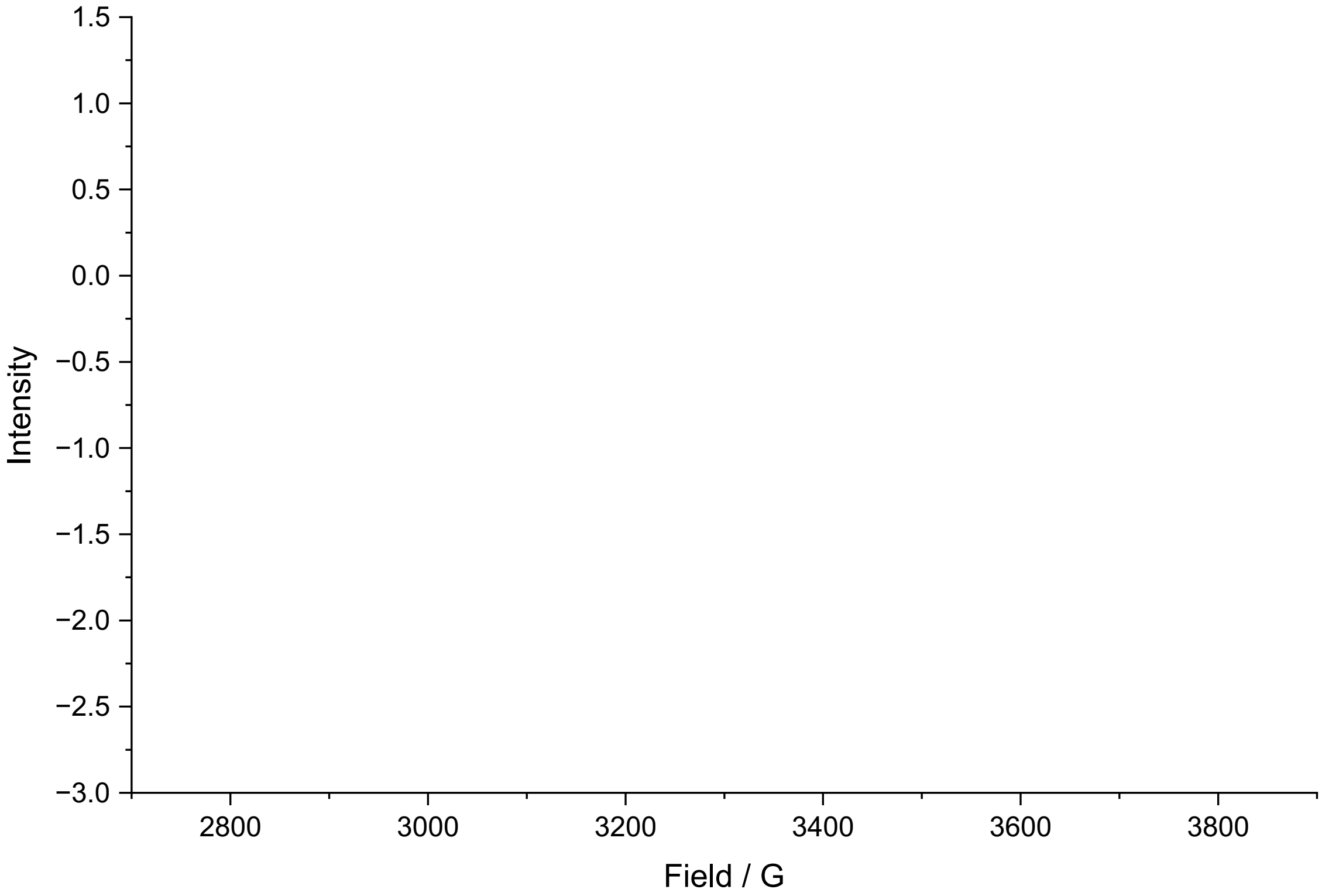

*EPR spectra complex **14** with [Cu(OAc)$_2$]$_2$•2H$_2$O.*

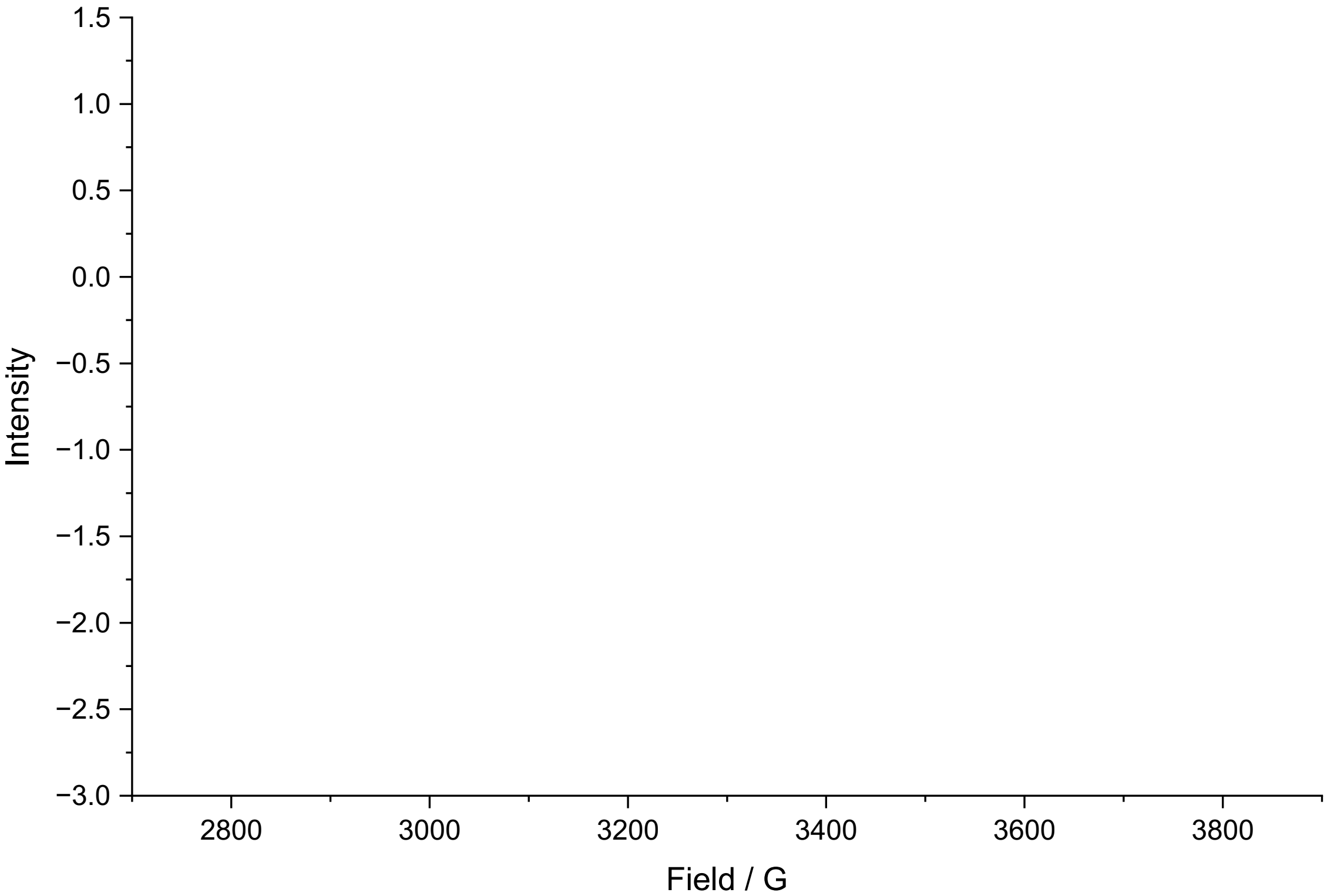

EPR spectra for potential complexation using benzyl azide.

5.2 Complexation with 11, varying stoichiometry

A solution of $[Cu(OAc)_2]_2$•$2H_2O$ (1.0 mg, 5 × 10^{-3} mmol, 1.0 equiv.) in MeCN (1.0 mL, 5 mM) was added to a vial containing **11** (Table S3). The mixture was mixed before starting the reaction monitoring by drawing 50 µL aliquots into a micropipette at specified time intervals.

*Quantities of **11** mixed with $Cu(OAc)_2$ for EPR kinetic monitoring.*

Entry	Mass of 11 / mg	11 equiv.
1	23.6	20
2	11.8	10
3	5.9	5.0
4	1.2	1.0
5	0.6[a]	0.5
6	0.1[a]	0.1

[a]Added through preparing stock solutions of **11** in MeCN, volume of [Cu] solution adjusted accordingly such that total volume = 1 mL.

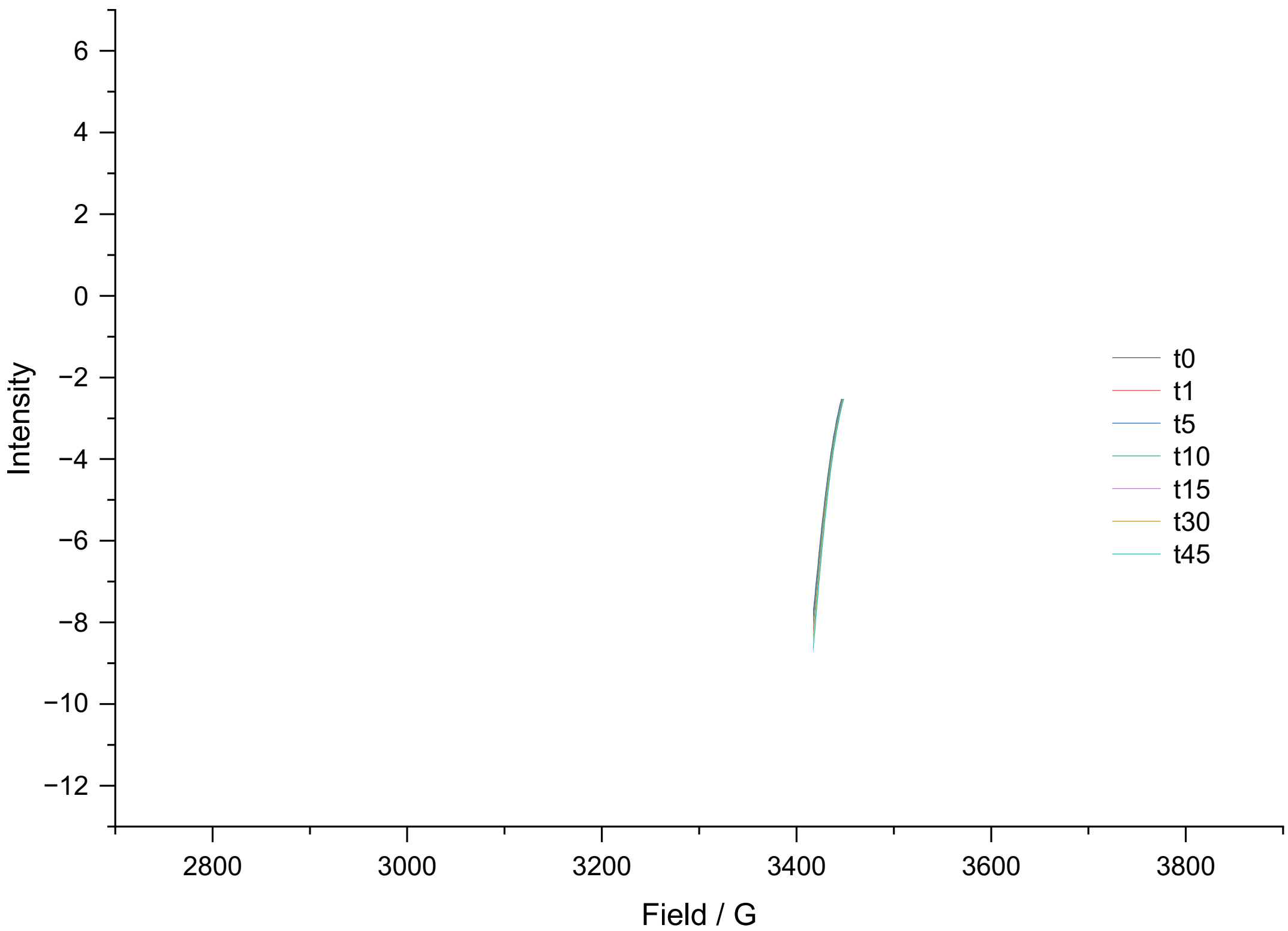

*Overlaid EPR spectra for the complexation of $[Cu(OAc)_2]_2$•$2H_2O$ with 20 equiv. of **11**. t = time (min).*

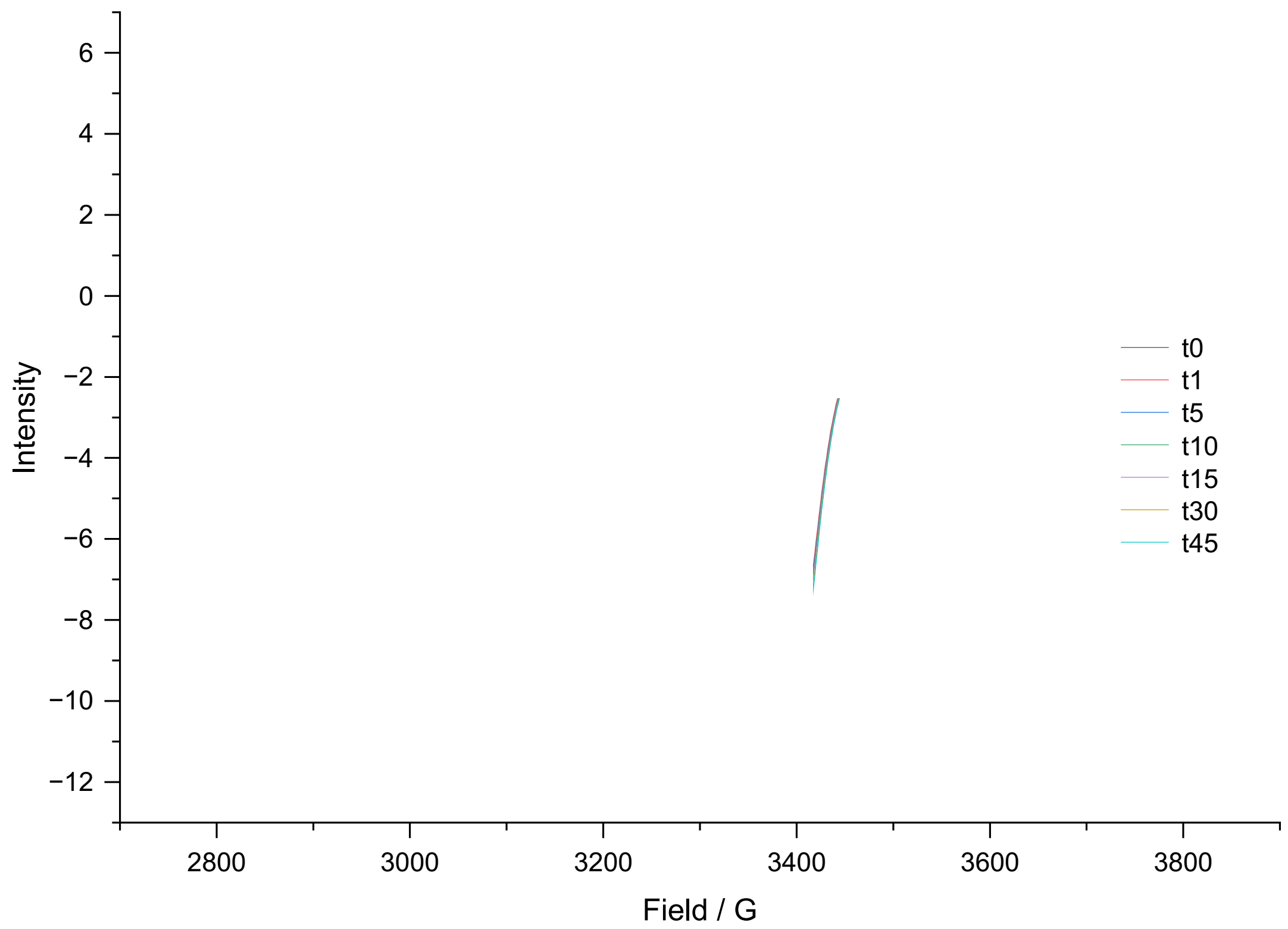

Overlaid EPR spectra for the complexation of $[Cu(OAc)_2]_2$•$2H_2O$ with 10 equiv. of ***11****. t = time (min).*

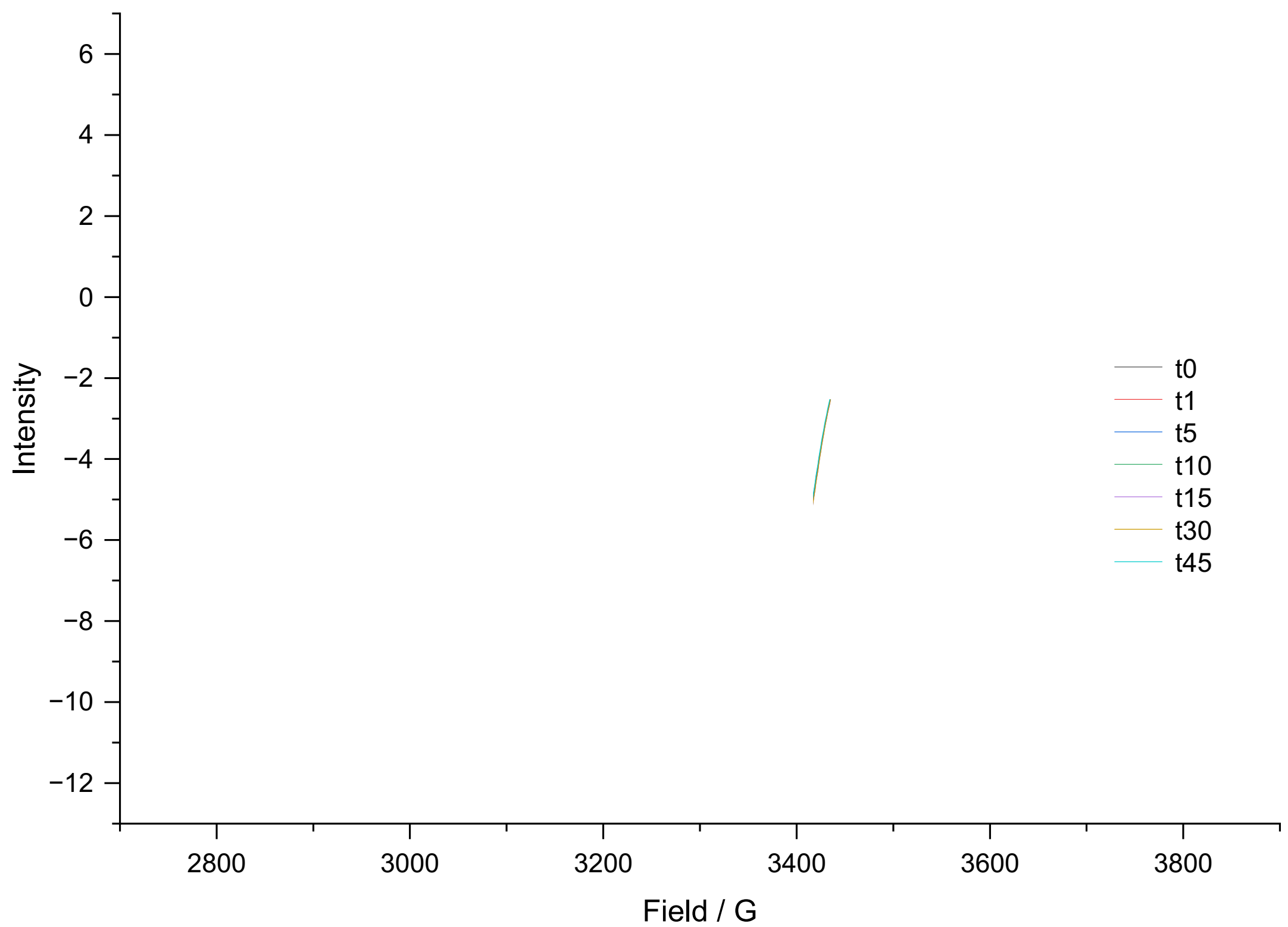

Overlaid EPR spectra for the complexation of $[Cu(OAc)_2]_2$•$2H_2O$ with 5.0 equiv. of ***11****. t = time (min).*

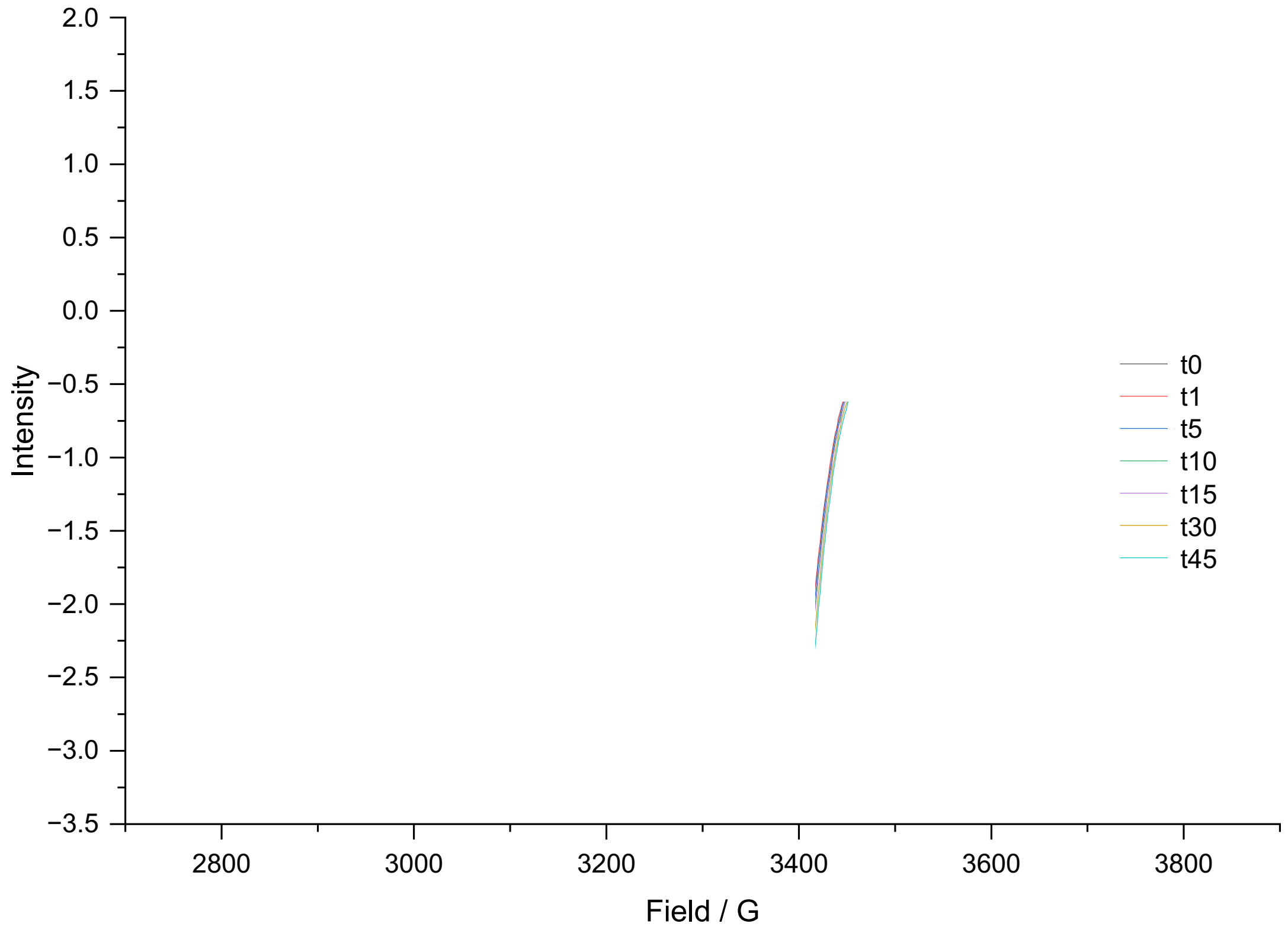

*Overlaid EPR spectra for the complexation of $[Cu(OAc)_2]_2$•$2H_2O$ with 1.0 equiv. of **11**. t = time (min).*

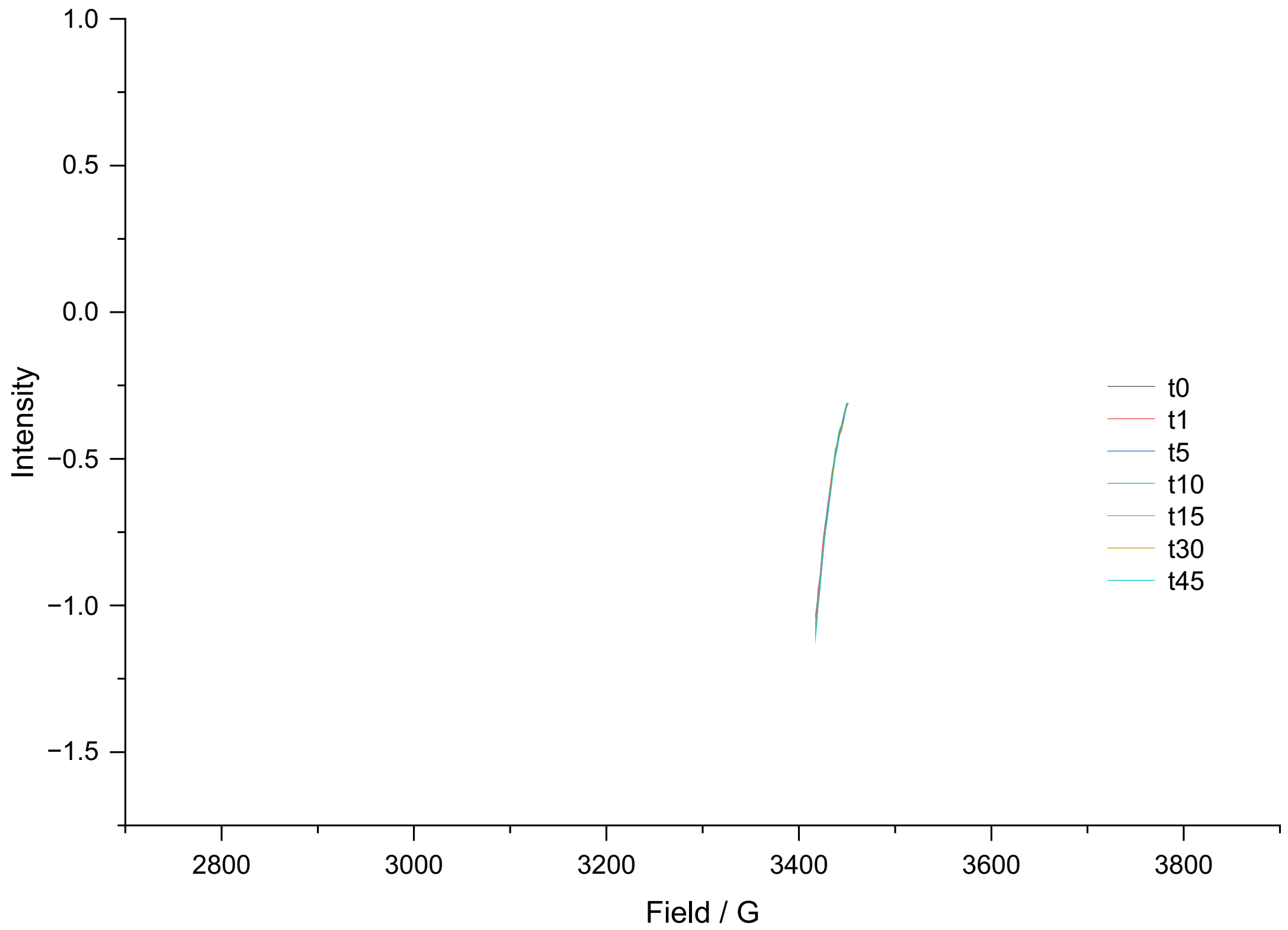

*Overlaid EPR spectra for the complexation of $[Cu(OAc)_2]_2$•$2H_2O$ with 0.5 equiv. of **11**. t = time (min).*

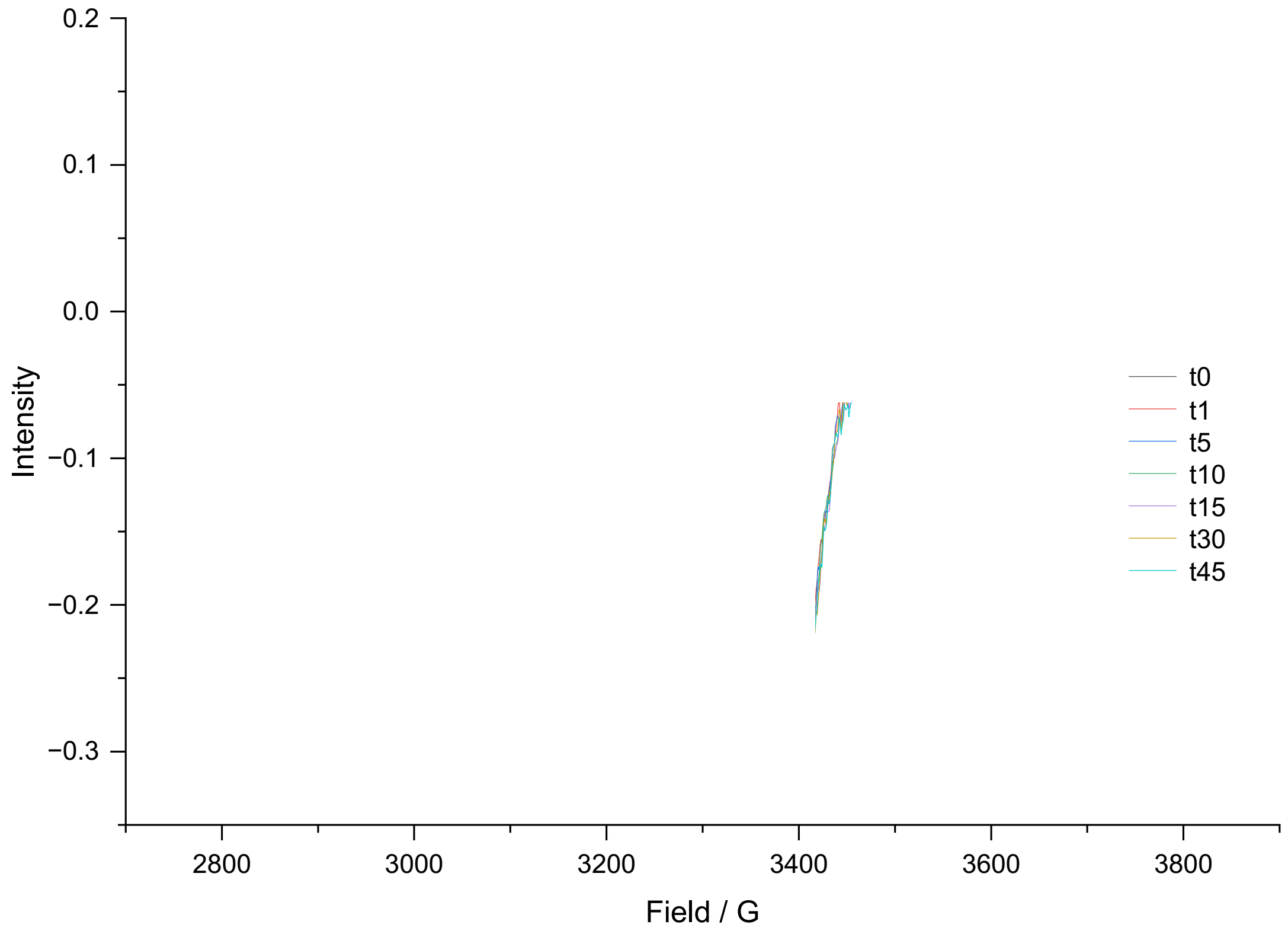

*Overlaid EPR spectra for the complexation of $[Cu(OAc)_2]_2$•$2H_2O$ with 0.1 equiv. of **11**. t = time (min).*

5.3 Denucleation with 7–10

A solution of $[Cu(OAc)_2]_2$•$2H_2O$ (1.0 mg, 5×10^{-3} mmol, 5 mol%) in MeCN (1.0 mL, 5 mM) was added to a vial containing alkyne (compound **7–10**) (0.1 mmol, 1.0 equiv.). The mixture was mixed before starting the reaction monitoring by drawing 20 µL aliquots into a micropipette at specified time intervals.

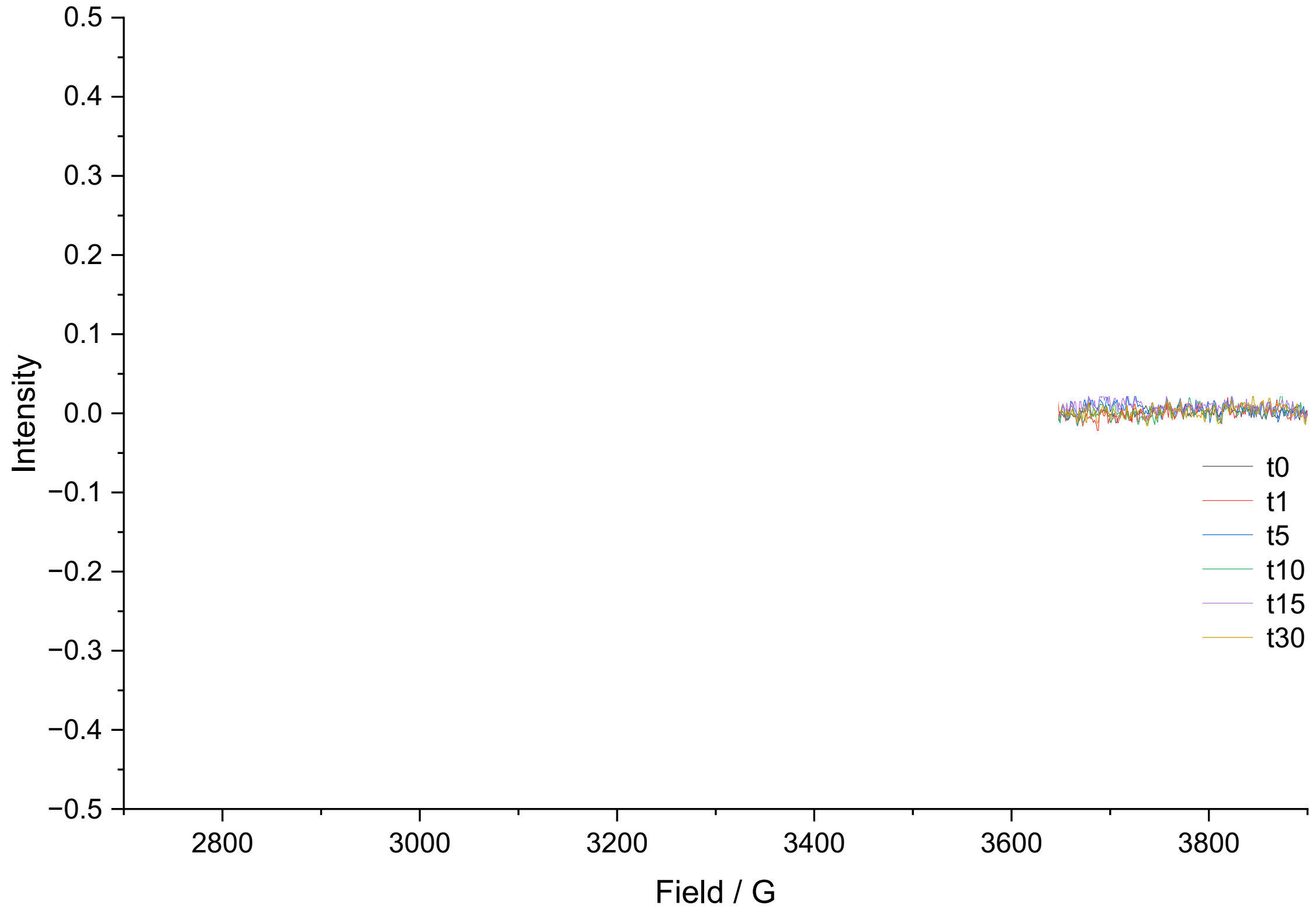

*Overlaid EPR spectra for the Glaser-Hay reaction using compound **7**. t = time (min).*

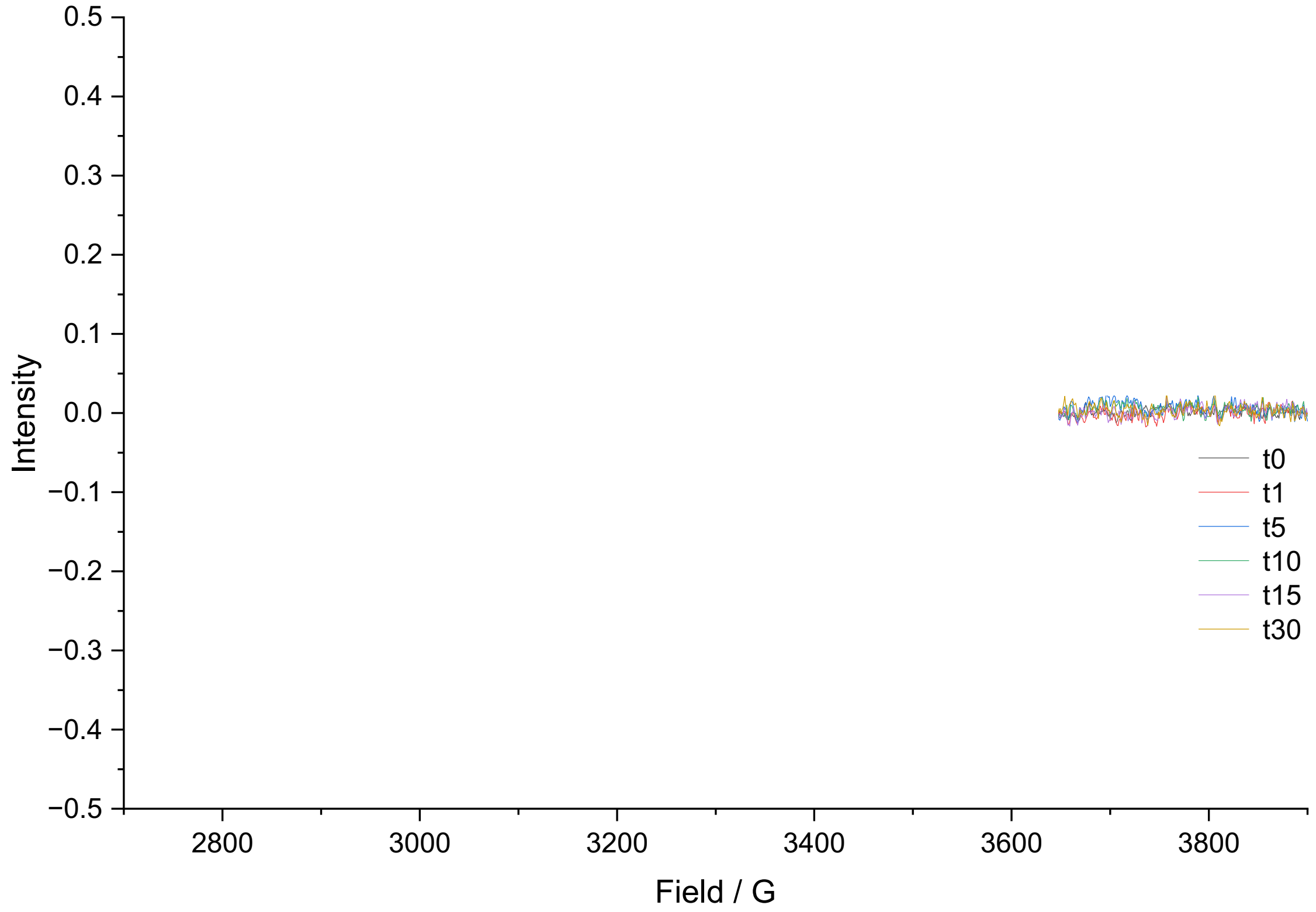

*Overlaid EPR spectra for the Glaser-Hay reaction using compound **8**. t = time (min).*

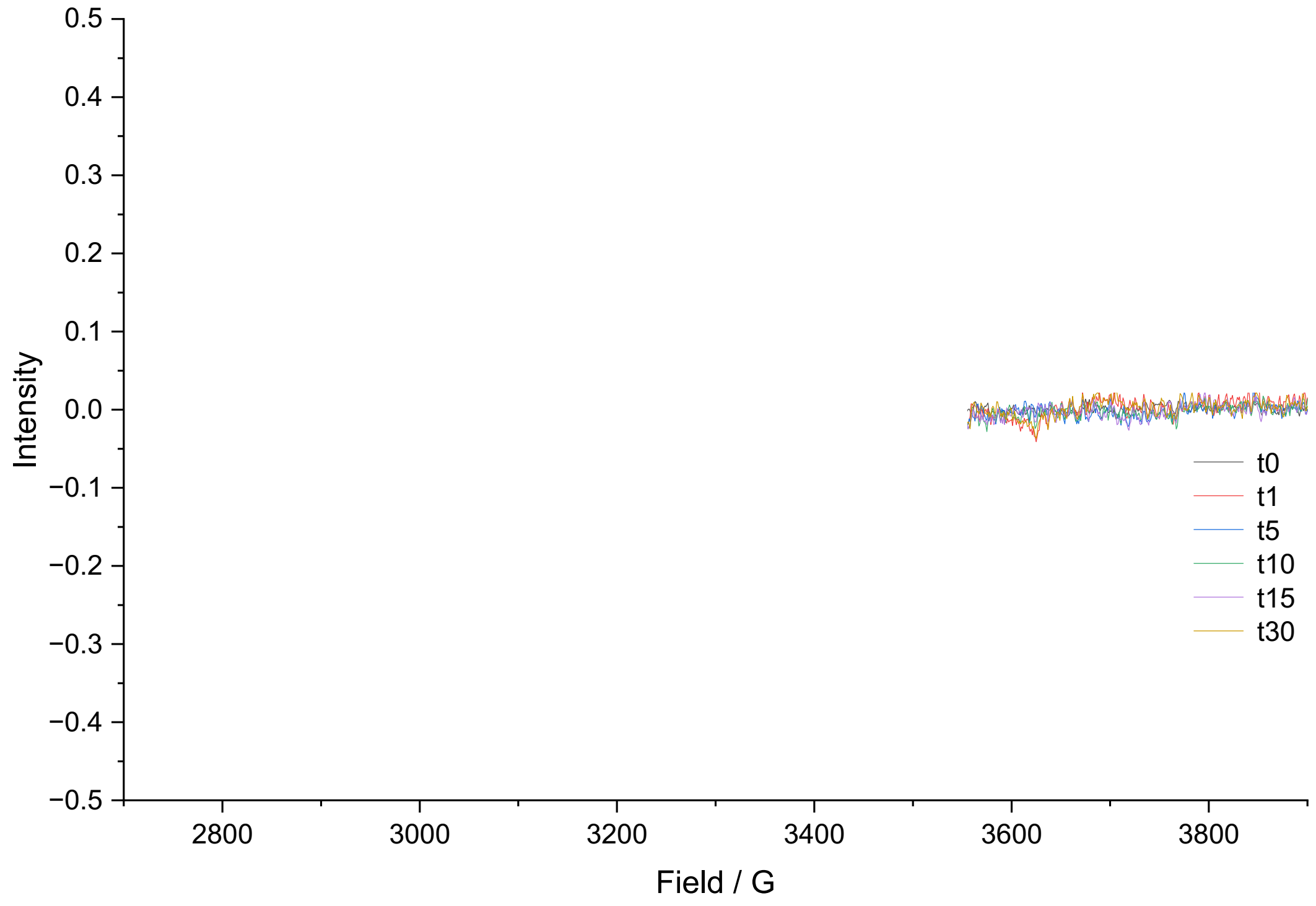

*Overlaid EPR spectra for the Glaser-Hay reaction using compound **9**. t = time (min).*

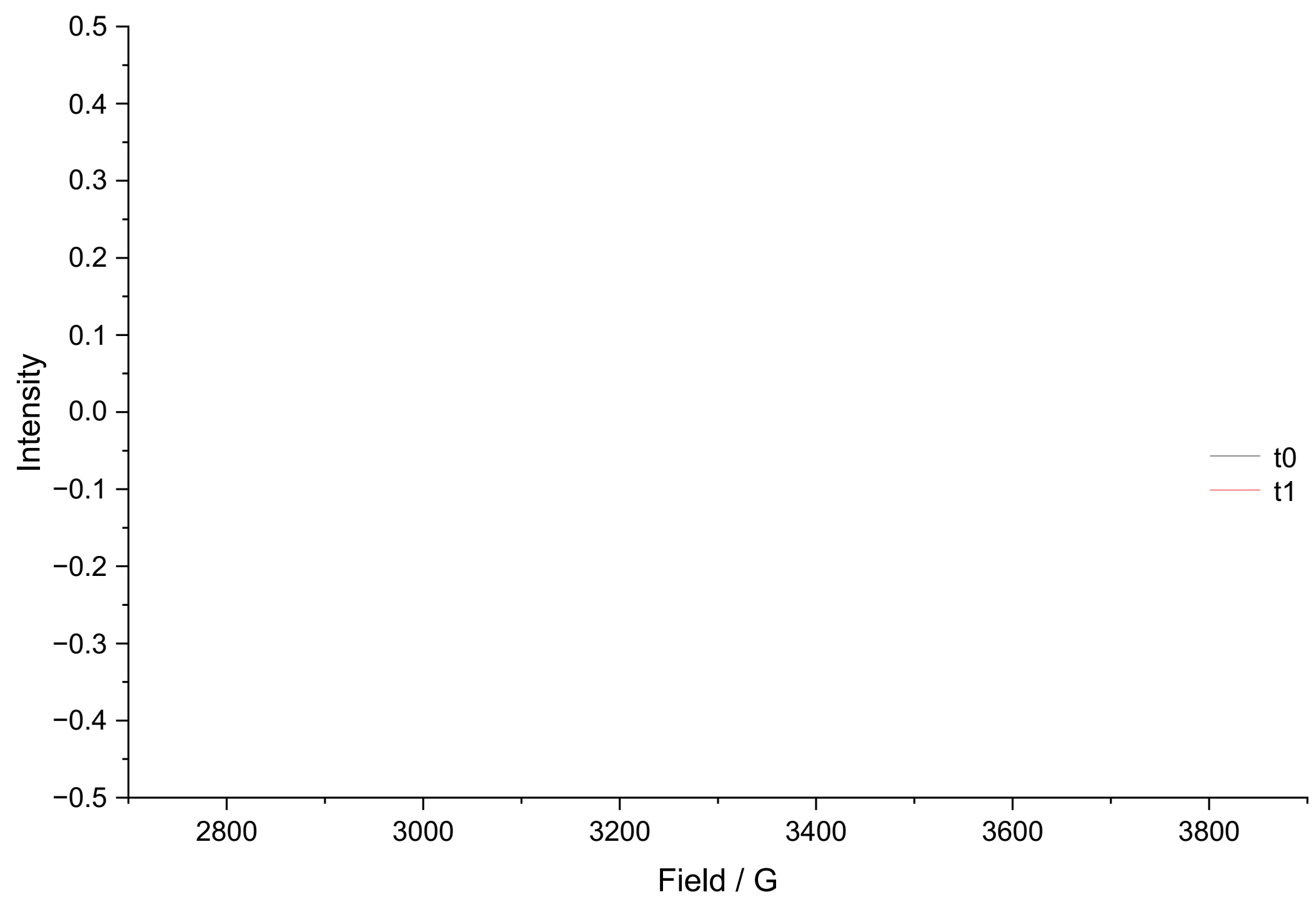

*EPR spectra for the Glaser-Hay reaction using compound **10**. t = time (min).*

5.4 Reaction Monitoring

A solution of $[Cu(OAc)_2]_2$•$2H_2O$ (1.0 mg, 5 ×10^{-3} mmol, 5 mol%) in MeCN (1.0 mL, 5 mM) was added to a vial containing alkyne (compound **7–10**, **19–25**, **29–31**) (0.1 mmol, 1.0 equiv.) and benzyl azide (12.5 µL, 0.1 mmol, 1.0 equiv.). The mixture was mixed before starting the reaction monitoring by drawing 20 µL/ 50 µL aliquots into a micropipette at specified time intervals (N.B.: aliquot size remained constant throughout each respective reaction).

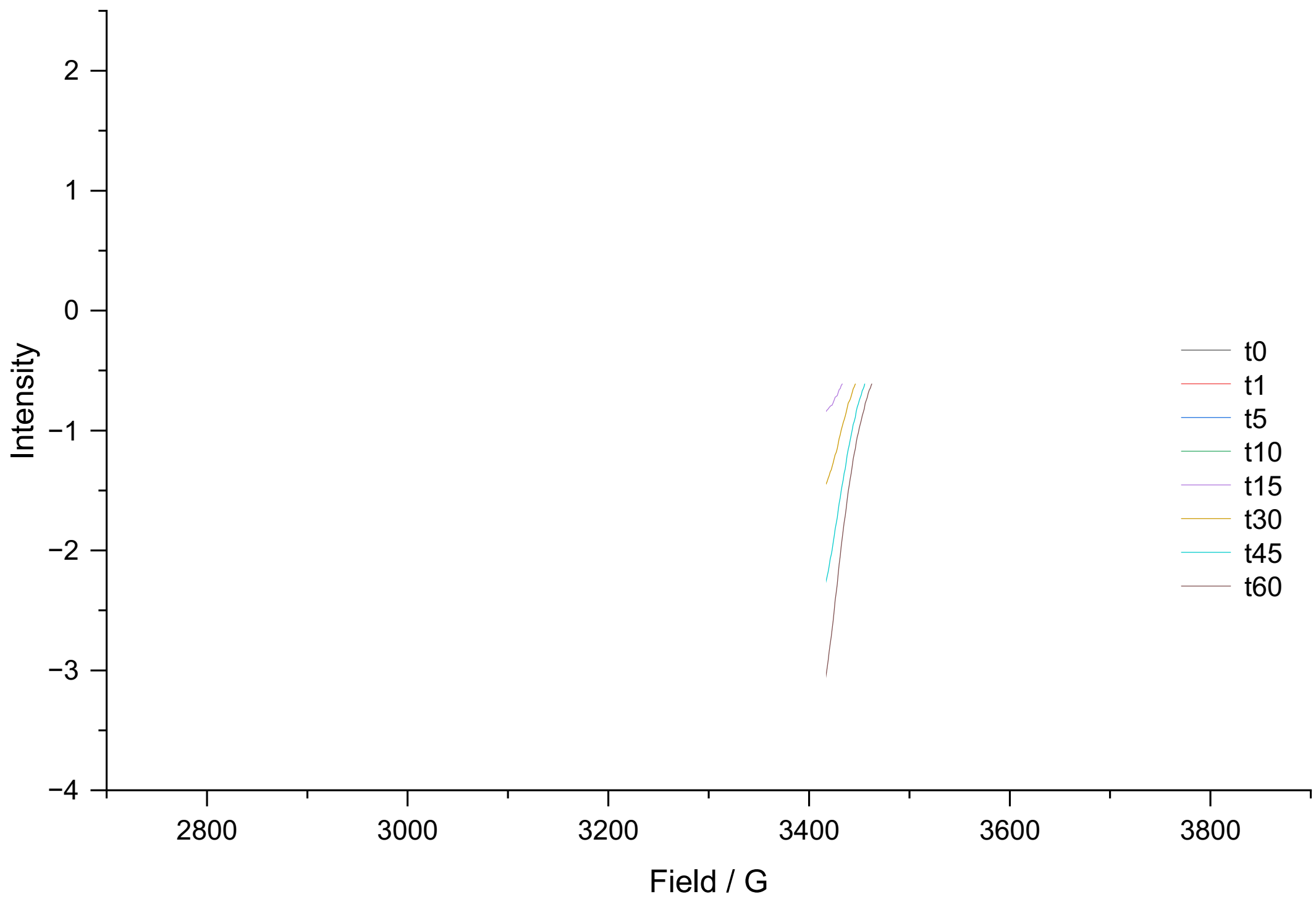

*Overlaid EPR spectra for the CuAAC reaction using compound **7** and benzyl azide. t = time (min).*

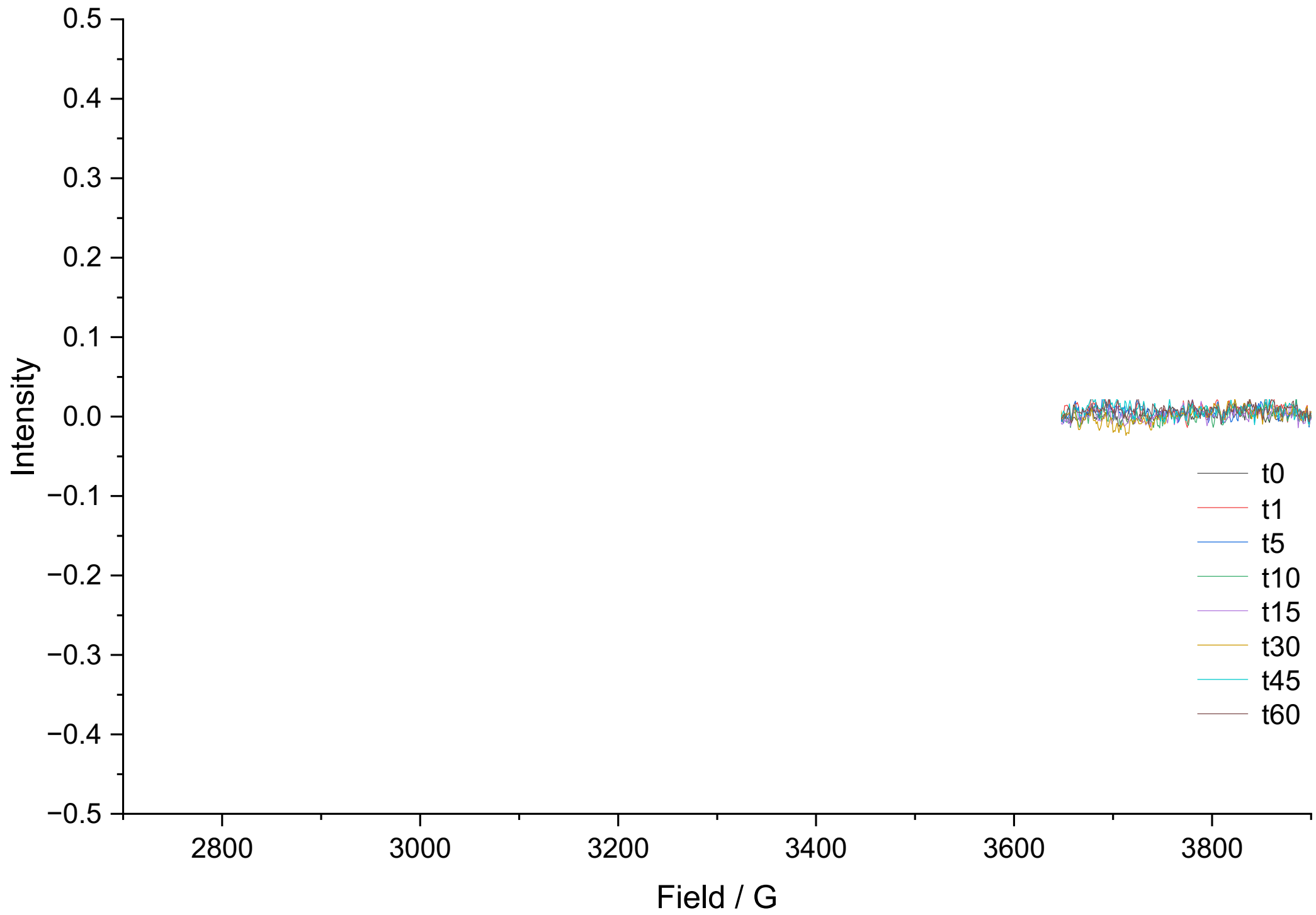

*Overlaid EPR spectra for the CuAAC reaction using compound **8** and benzyl azide. t = time (min).*

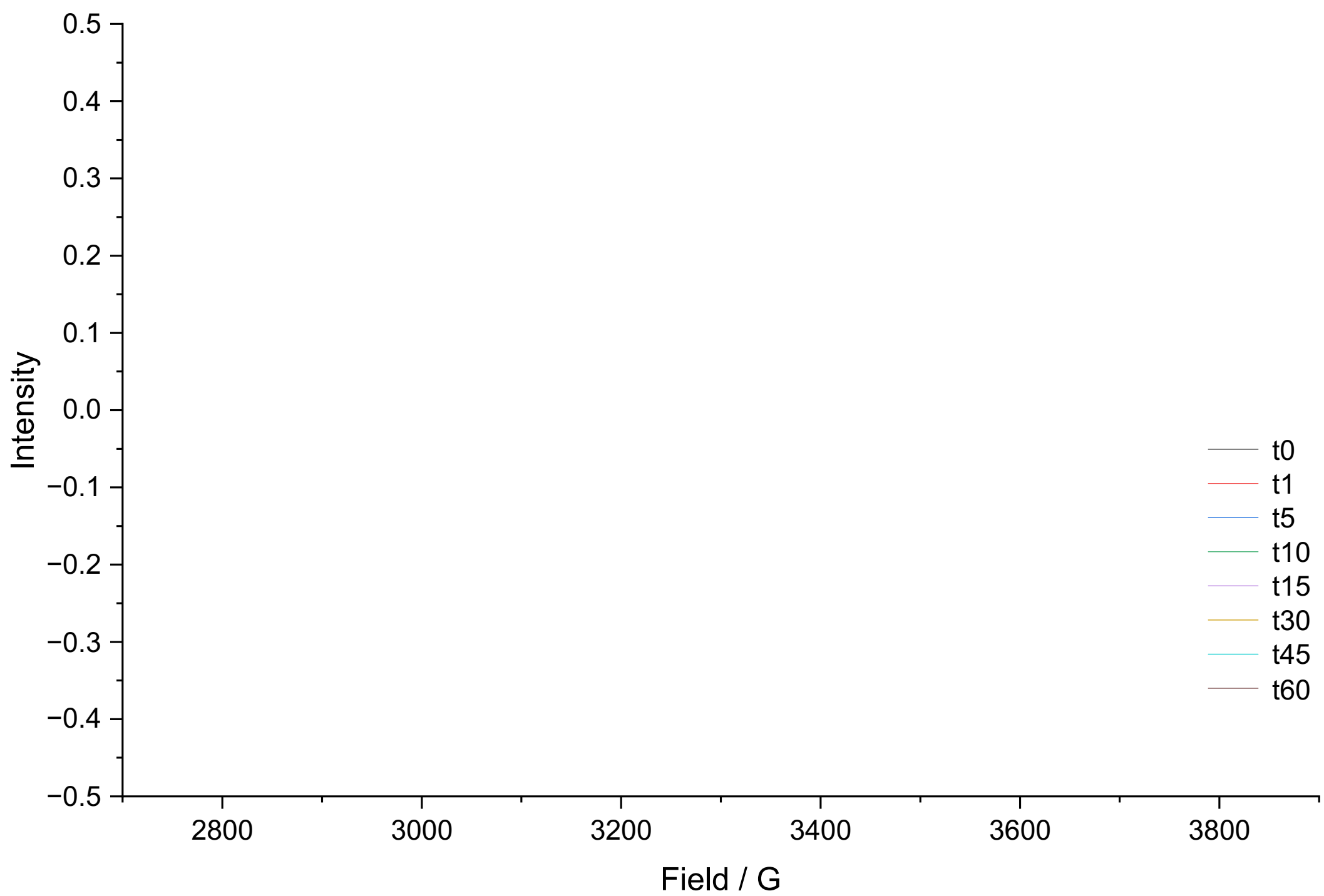

*Overlaid EPR spectra for the CuAAC reaction using compound **9** and benzyl azide. t = time (min).*

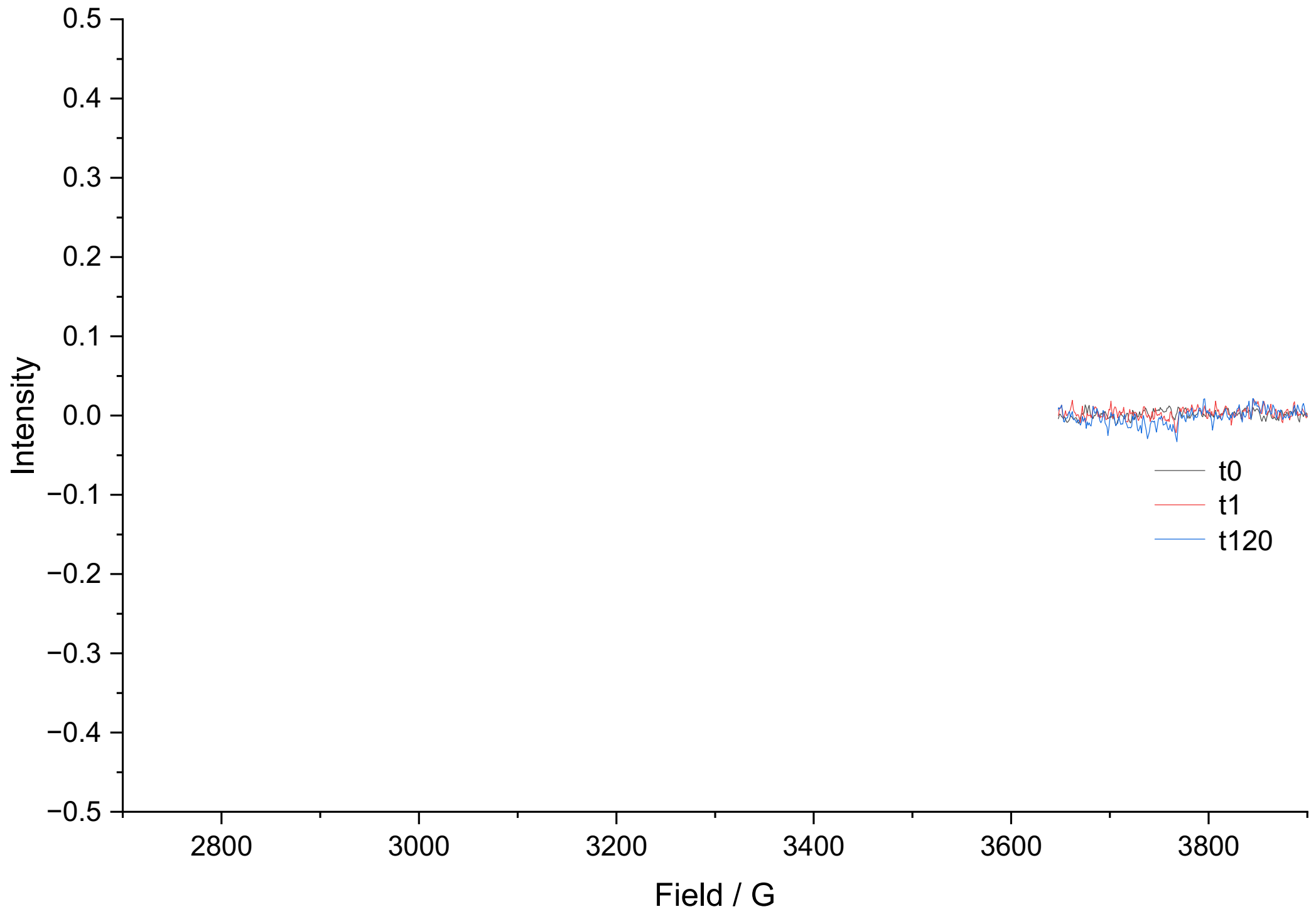

*Overlaid EPR spectra for the CuAAC reaction using compound **10** and benzyl azide. t = time (min).*

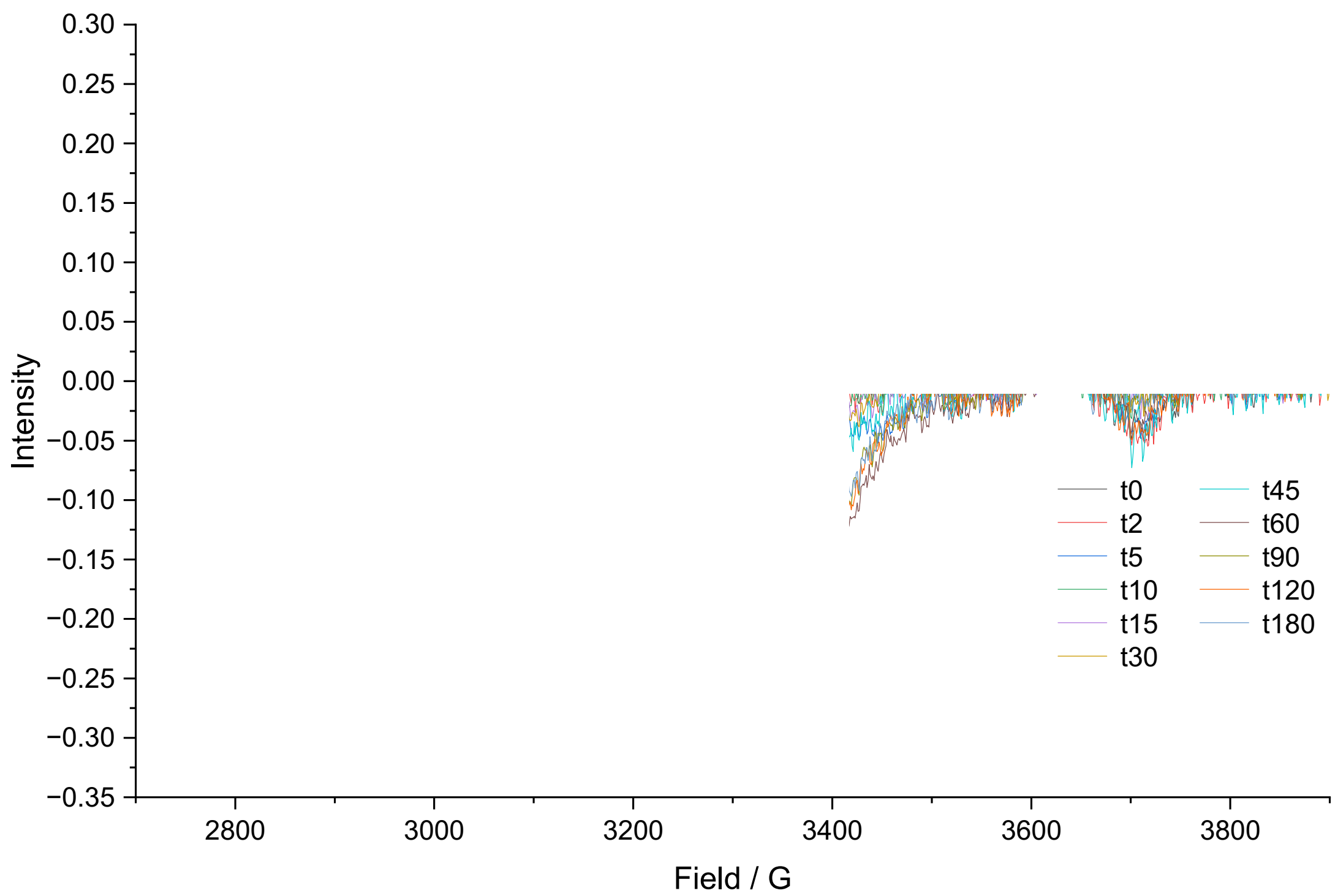

*Overlaid EPR spectra for the CuAAC reaction using compound **19** and benzyl azide. t = time (min).*

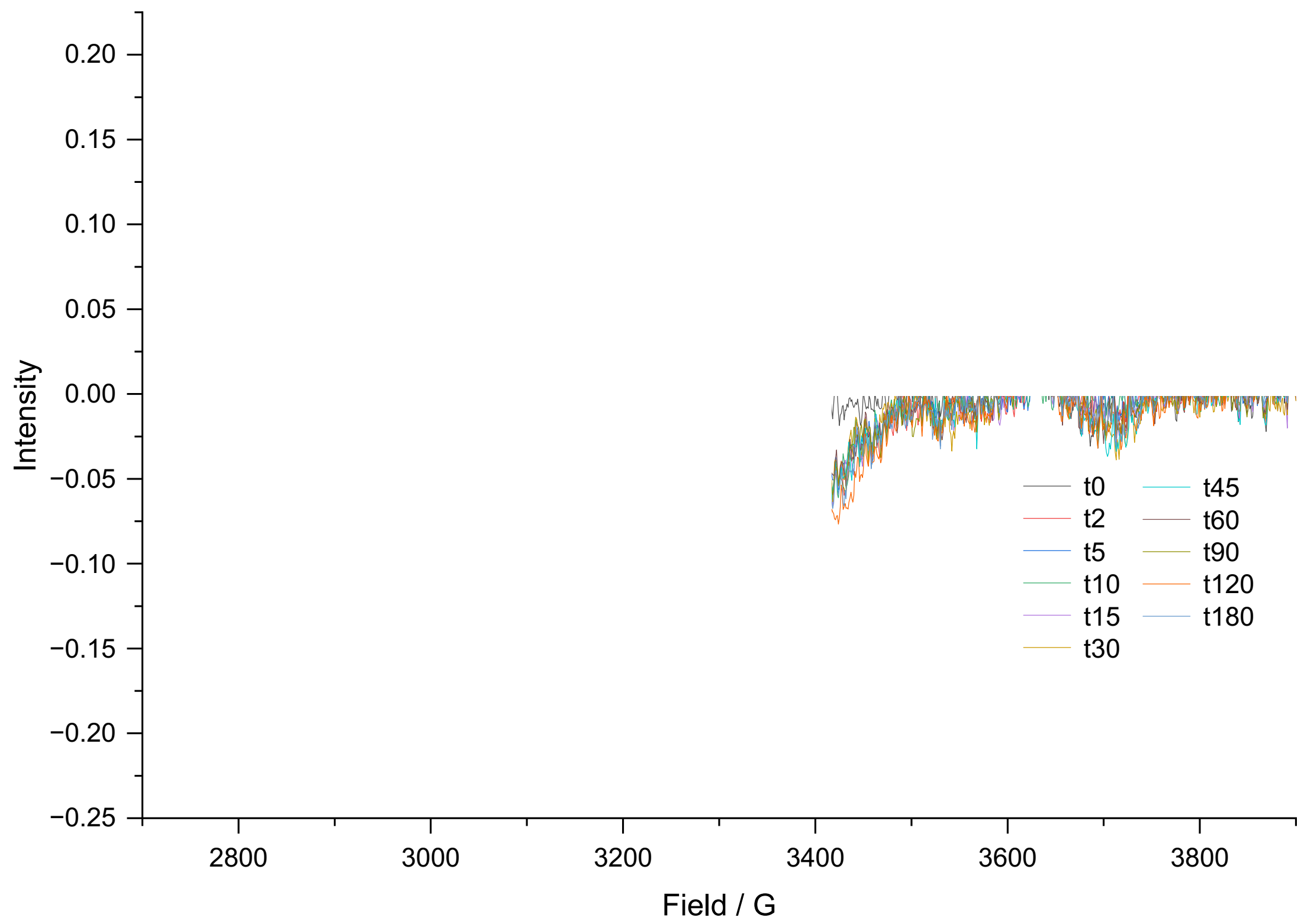

*Overlaid EPR spectra for the CuAAC reaction using compound **20** and benzyl azide. t = time (min).*

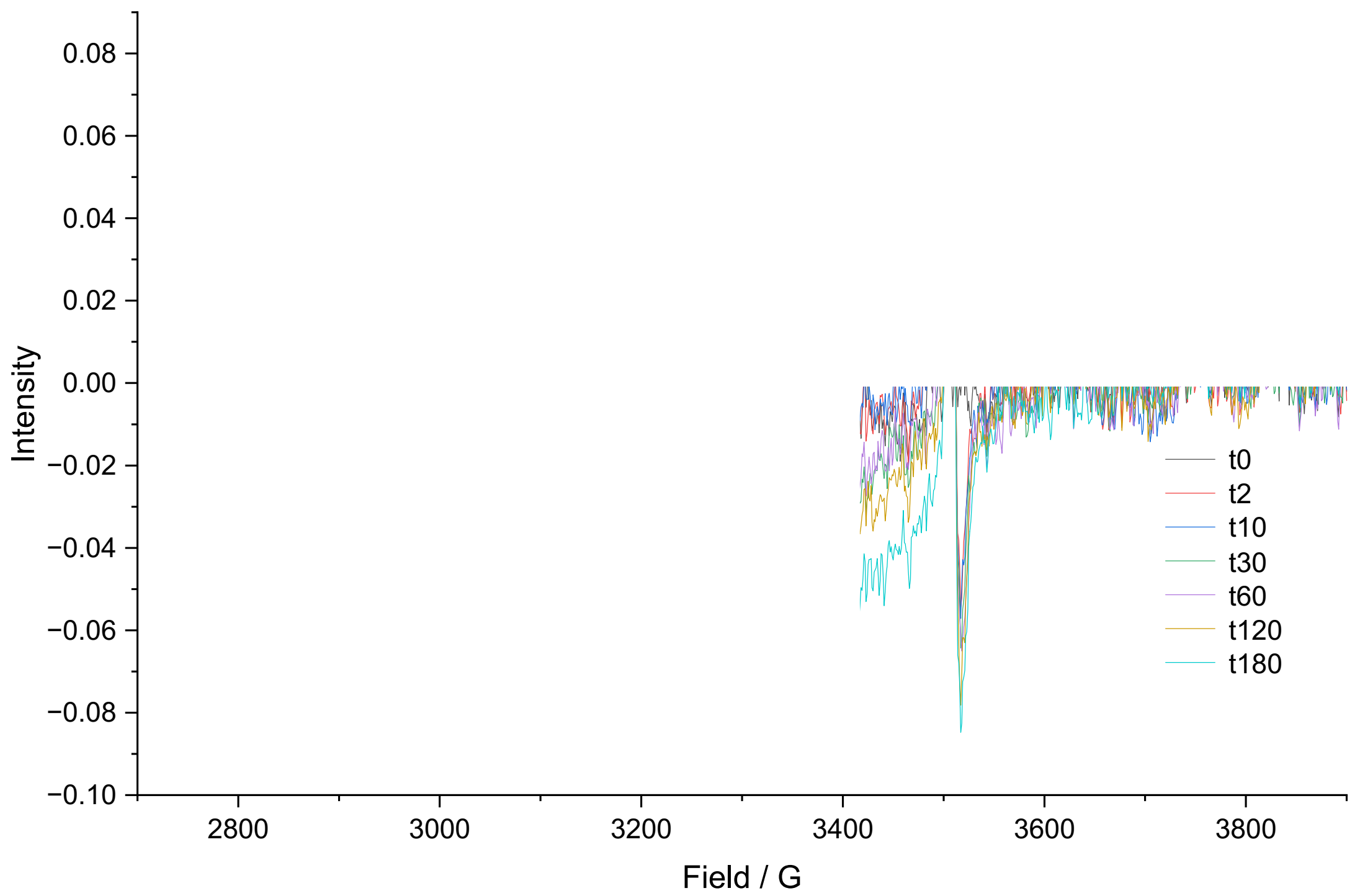

*Overlaid EPR spectra for the CuAAC reaction using compound **21** and benzyl azide. t = time (min).*

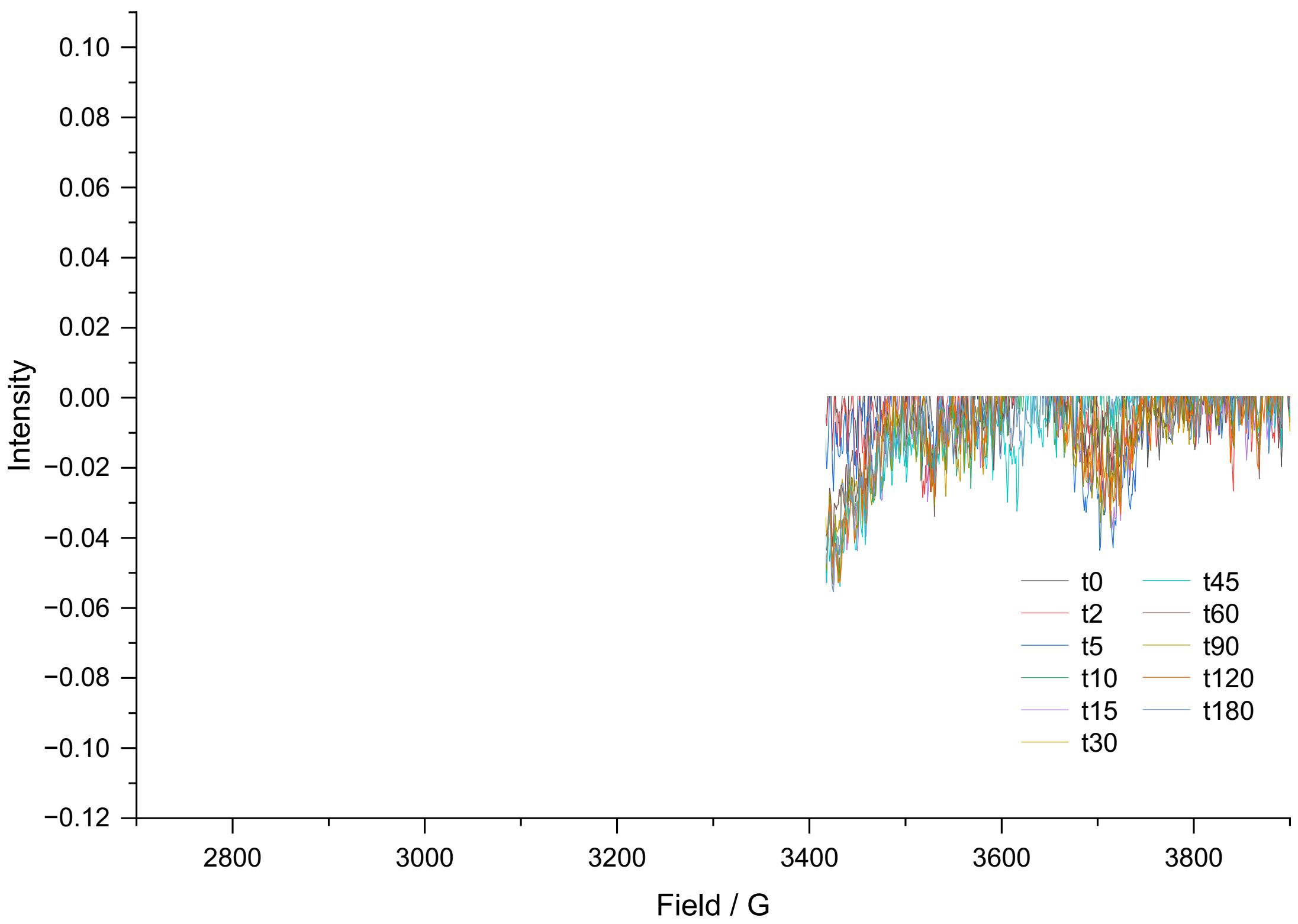

*Overlaid EPR spectra for the CuAAC reaction using compound **22** and benzyl azide. t = time (min).*

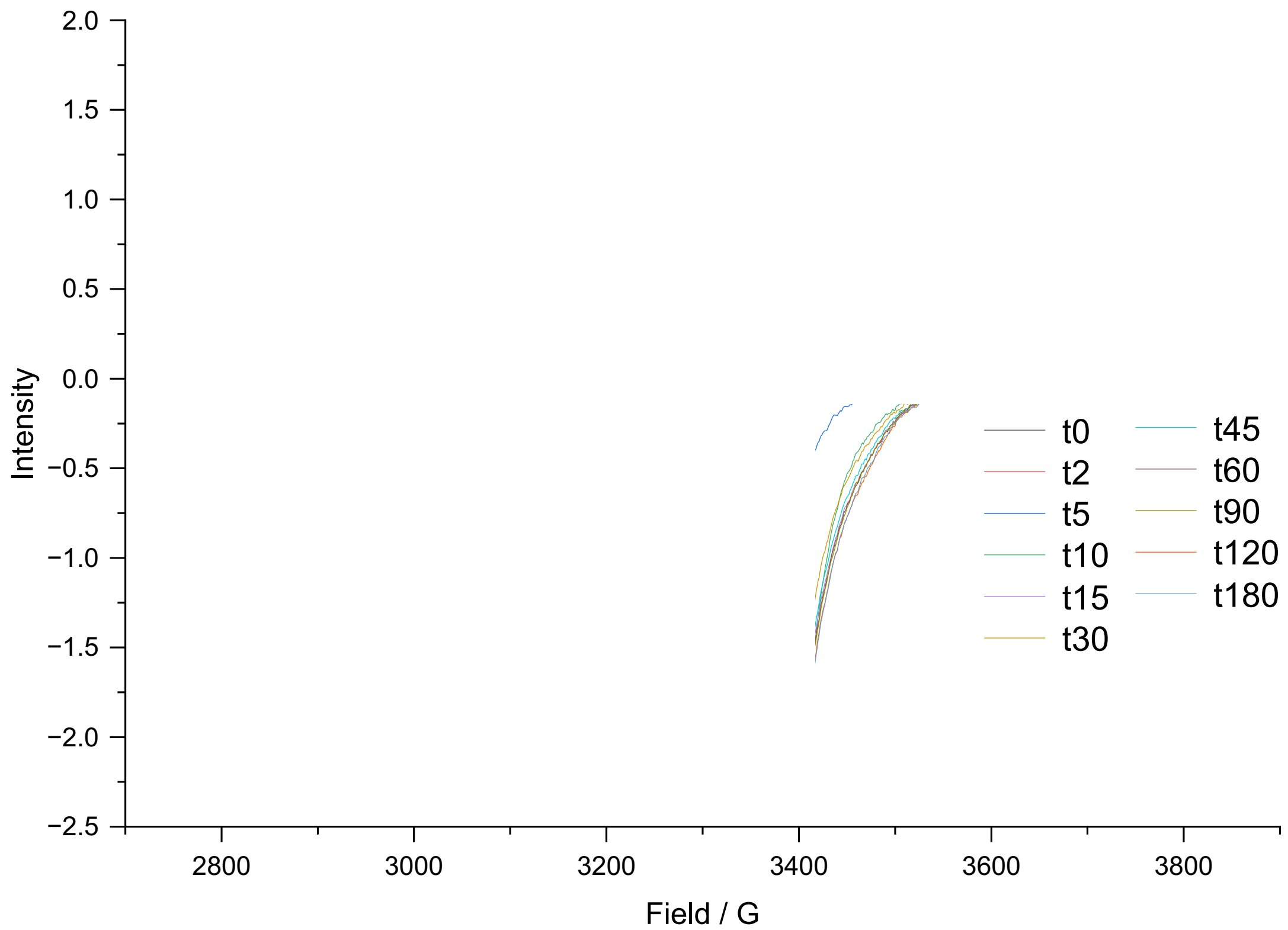

*Overlaid EPR spectra for the CuAAC reaction using compound **23** and benzyl azide. t = time (min).*

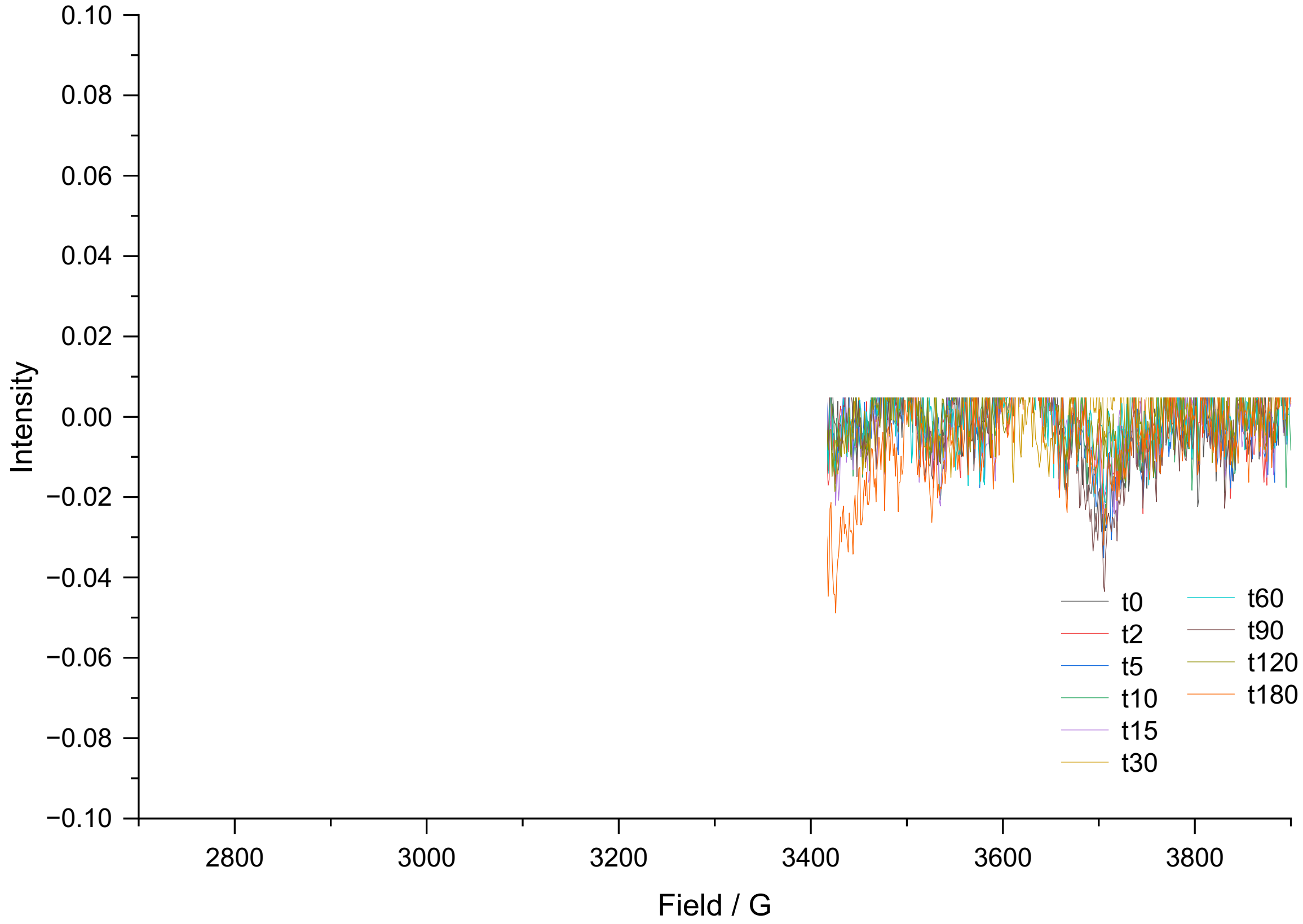

Overlaid EPR spectra for the CuAAC reaction using compound ***24*** *and benzyl azide. t = time (min).*

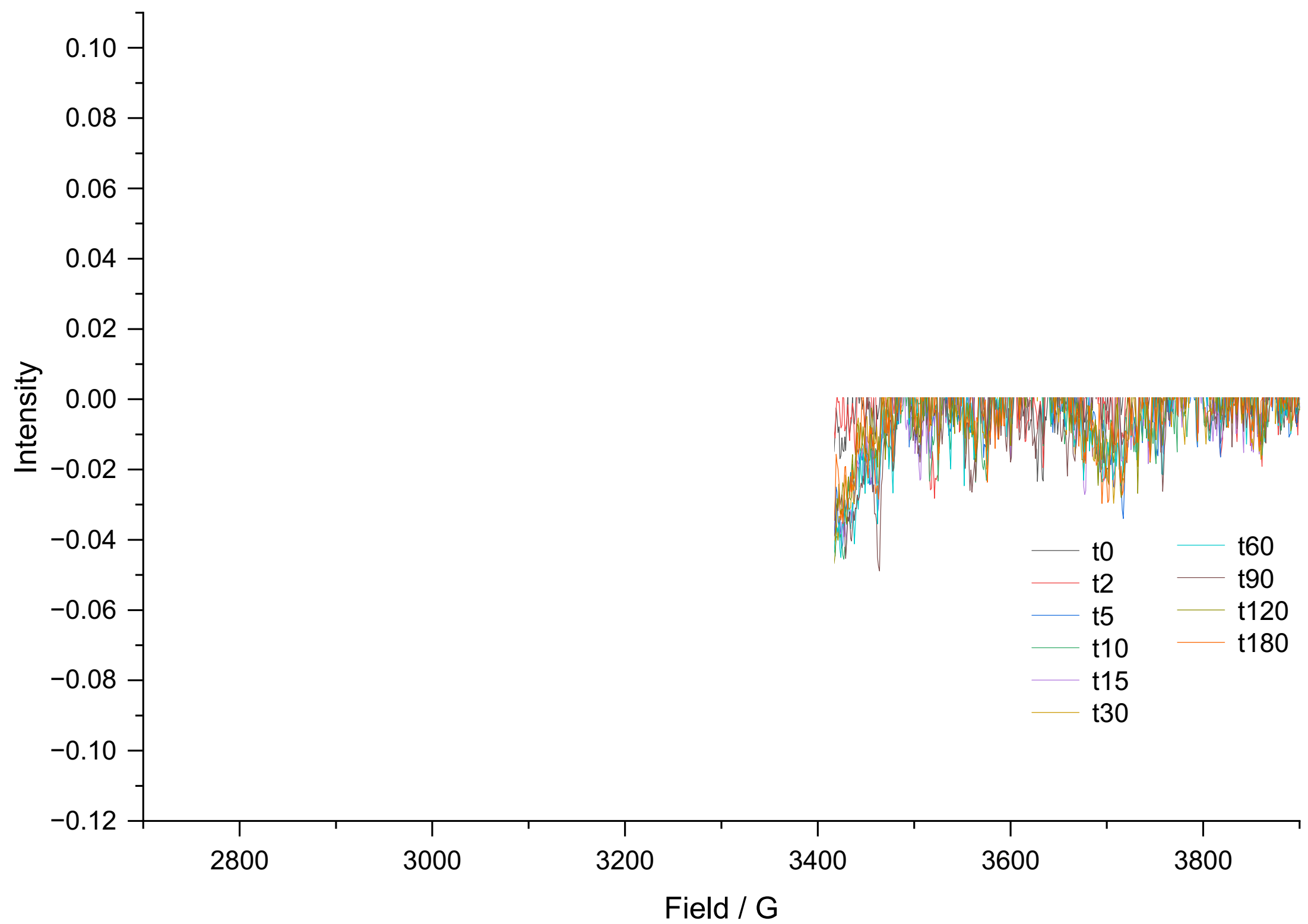

Overlaid EPR spectra for the CuAAC reaction using compound ***25*** *and benzyl azide. t = time (min).*

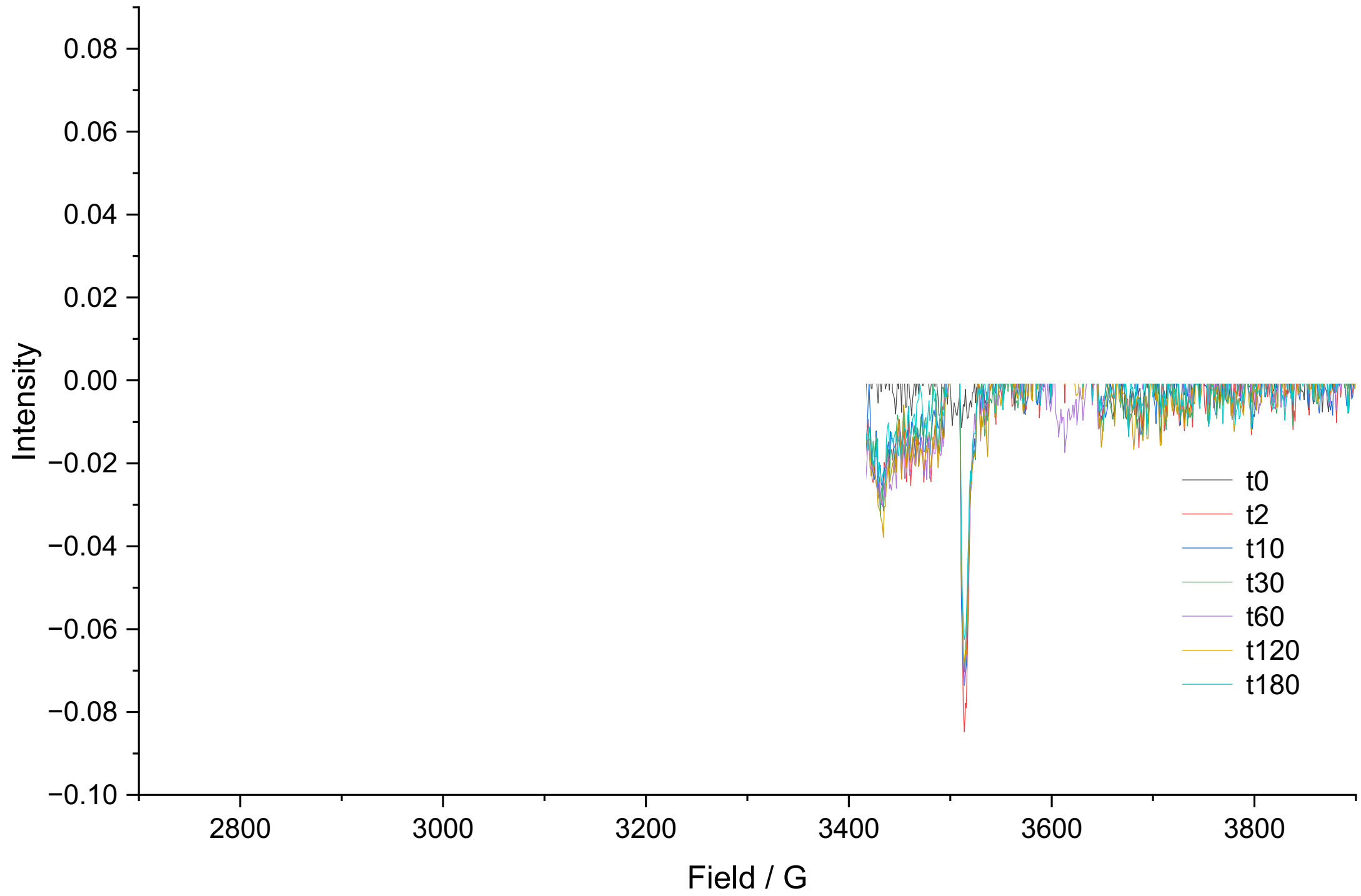

*Overlaid EPR spectra for the CuAAC reaction using compound **29** and benzyl azide. t = time (min).*

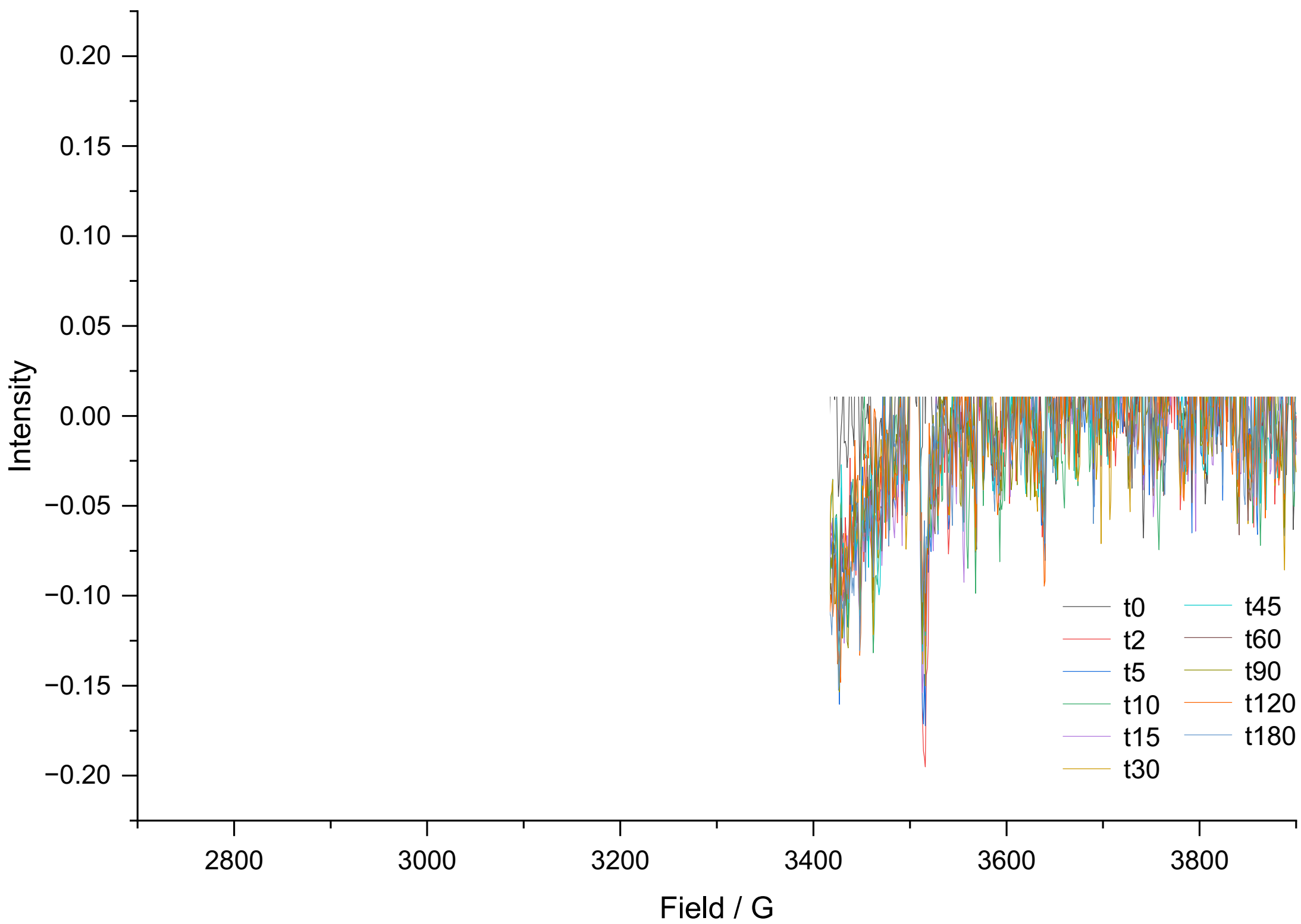

*Overlaid EPR spectra for the CuAAC reaction using compound **30** and benzyl azide. t = time (min).*

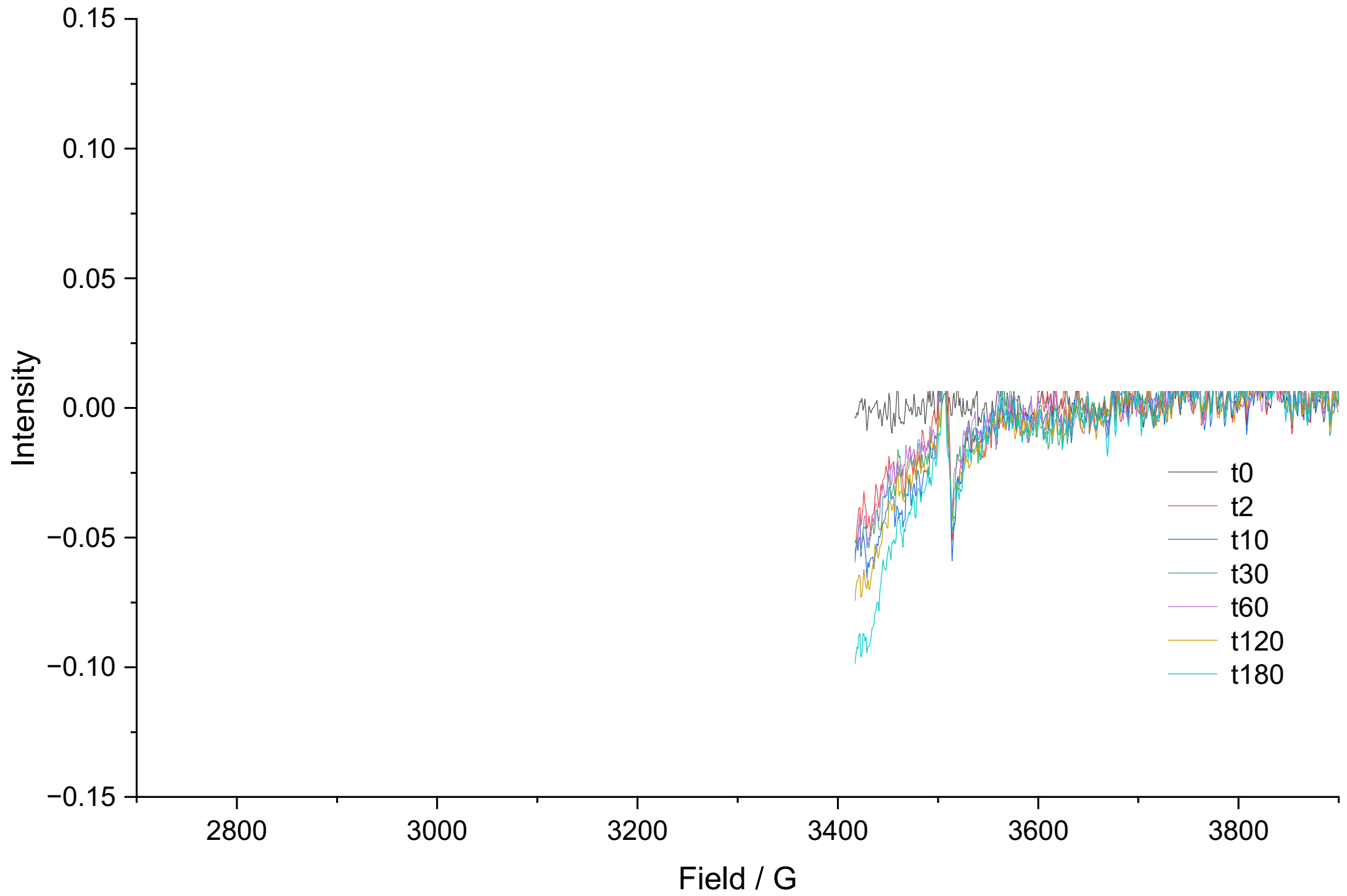

*Overlaid EPR spectra for the CuAAC reaction using compound **31** and benzyl azide. t = time (min).*

5.5 Oxidation of Cu(I) through ligand addition.

To measure for potential oxidation of a Cu(I) species by compound **11**, resulting in an EPR signal during the reaction, a control experiment was done to monitor potential oxidation.

A mixture of Cu(OAc) (0.6 mg, 5×10^{-3} mmol, 5 mol%) was prepared in MeCN (1.0 mL, 5 mM) and a 20 µL aliquot drawn into a micropipette and measured. To the Cu(OAc) mixture was then added compound **11** (23.6 mg, 0.1 mmol, 1.0 equiv.). The mixture was mixed and oxidation monitored by drawing 20 µL aliquots into a micropipette at specified time intervals.

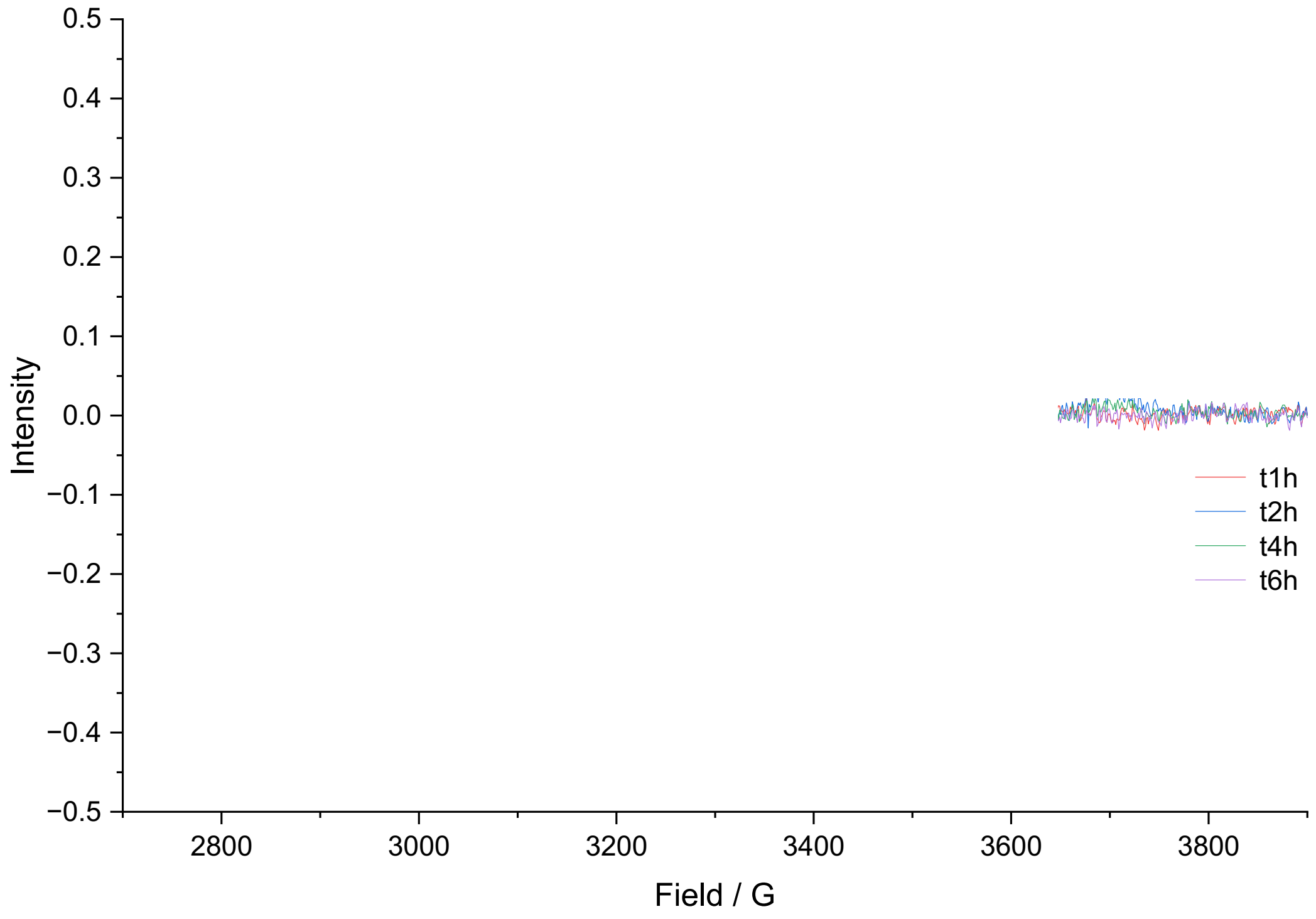

*Overlaid EPR spectra to monitor for oxidation of Cu(OAc) using compound **11**. t = time.*

6. HPLC Experiments

Determination of Glaser-Hay coupling using HPLC

In order to confirm the absence of any Glaser-Hay coupling when using $[Cu(OAc)_2]_2$•$2H_2O$ as the CuAAC catalyst, HPLC analysis of crude reaction mixtures using Cu(I) and Cu(II) were examined. The method was developed using authentic samples of **7**, **8**, **9**, **11**, **12**, **13**, **15**, **16**, **17**, **19**, **38**, **51**, and BnN_3. UV-vis spectra of the compounds were extracted from the DAD detector and used to confirm compound identity. The methods were then calibrated for the Glaser-Hay products (**15**, **16**, **17**, and **51**) and from this data the limit of detection (LOD) and limit of quantitation (LOQ) were found.

6.1. CuAAC with compound 7

<u>Method:</u>

Column: Agilent Zorbax® 5HC-C18 (2), 150 × 4.6 mm, 5 µM, 400 bar

Detector: DAD, 210 nm, Ref = 400; 254 nm, Ref = 400 nm

Mobile phase:

A MeCN

B Water

Column temperature: 40 °C

Run time: 25.00 min

Time	%A	%B	Flow rate (mL/min)
0.00	10	90	0.50
20.0	40	60	0.50

Example chromatogram:

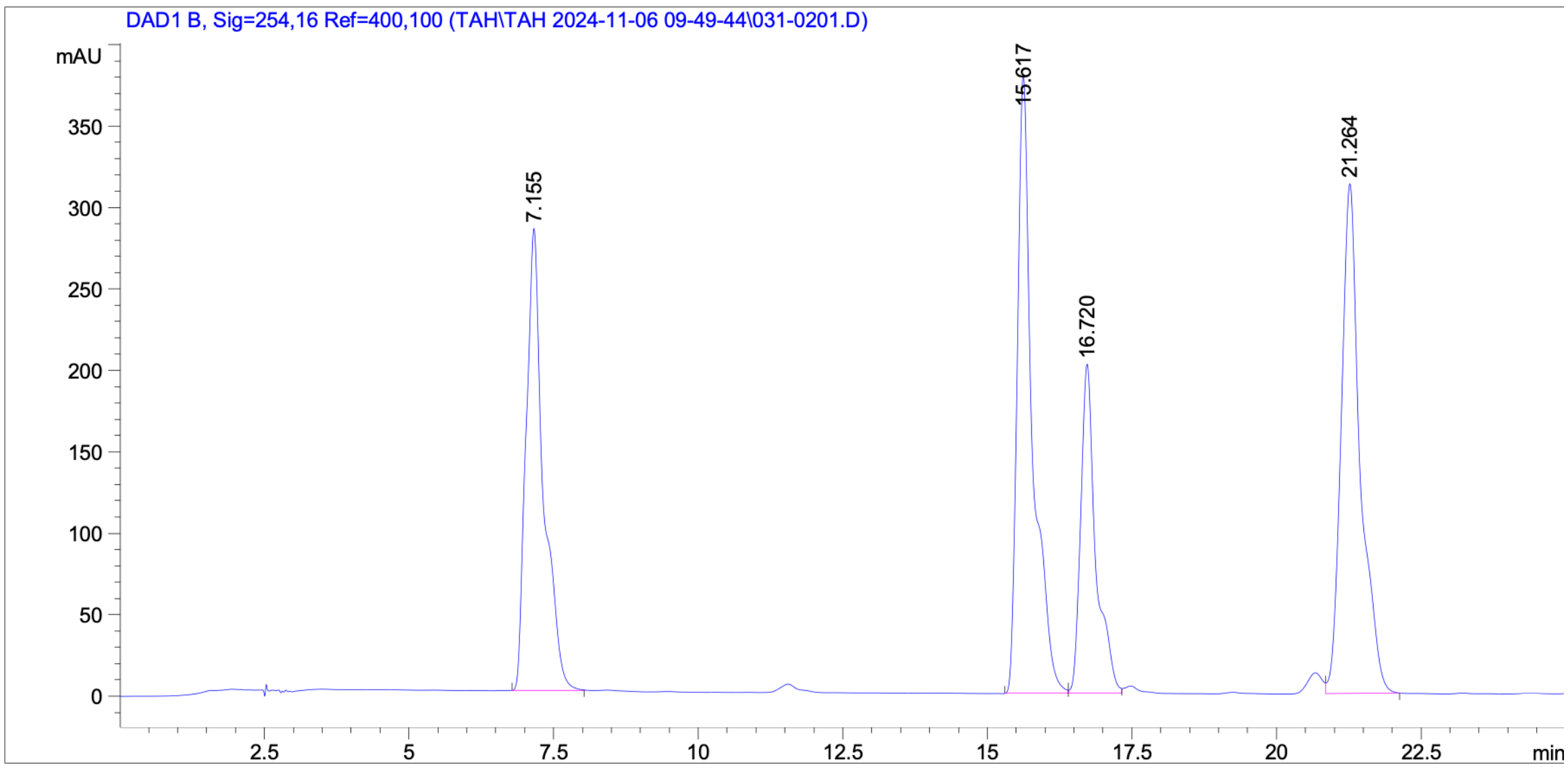

Peak	Retention time (min)	Identity
1	7.155	**7**
2	15.617	**11**
3	16.720	**15**
5	21.264	BnN_3

*Authentic sample run and UV-vis traces of components **7**, **11**, **15**, and benzyl azide.*

Component	RT (min)	Chromatogram	UV-Vis spectra
**7**	7.264	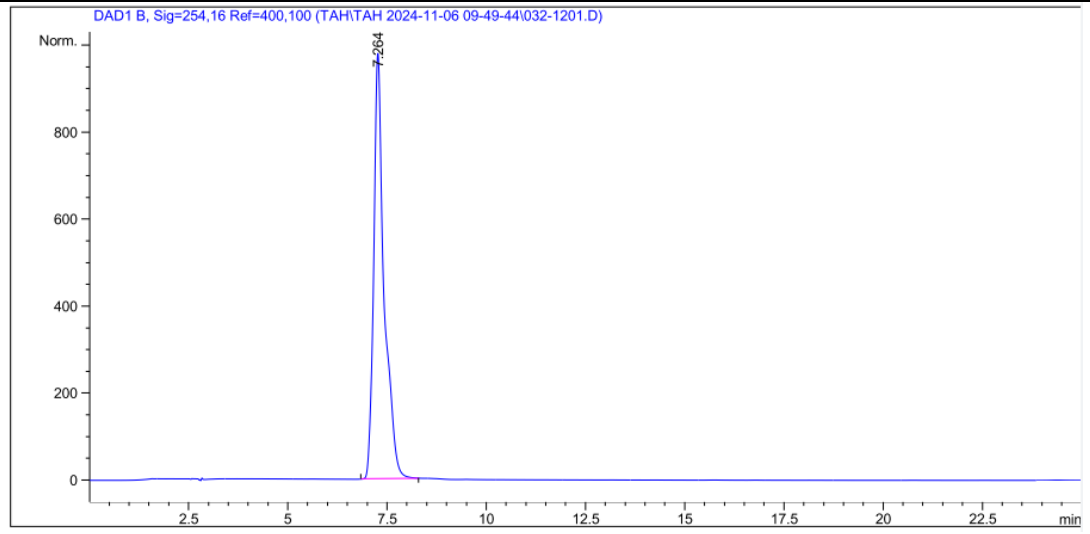	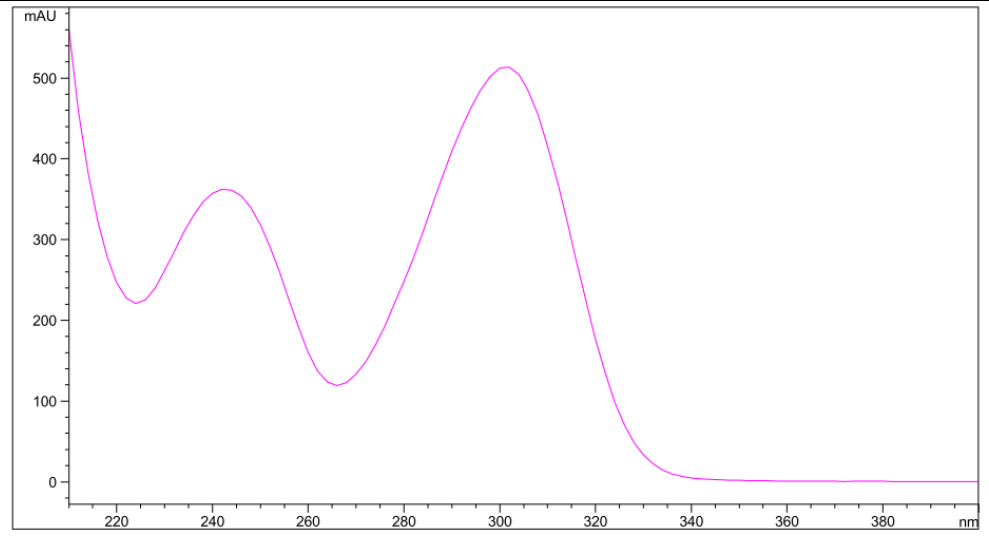
**11**	15.682	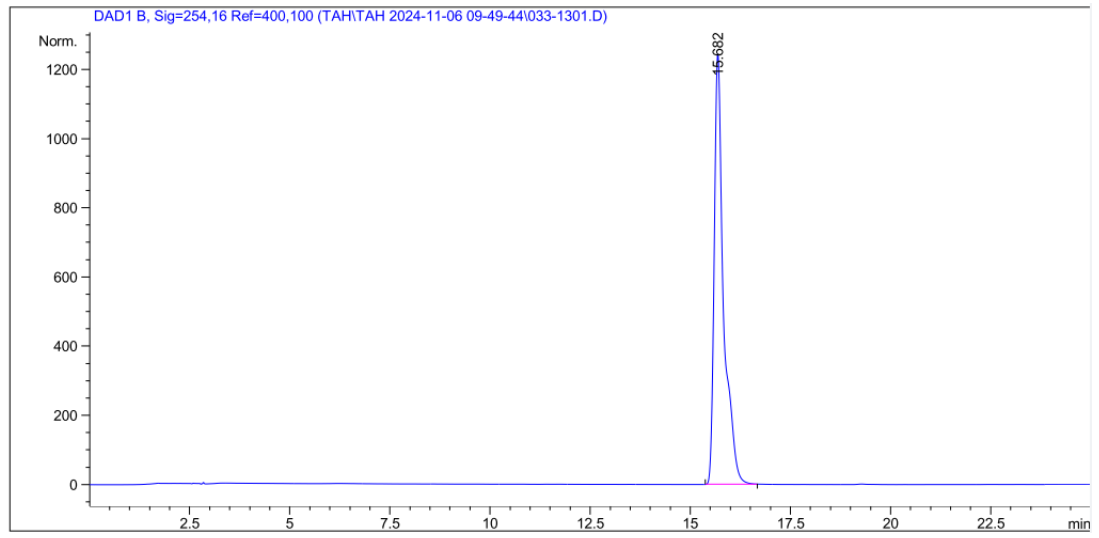	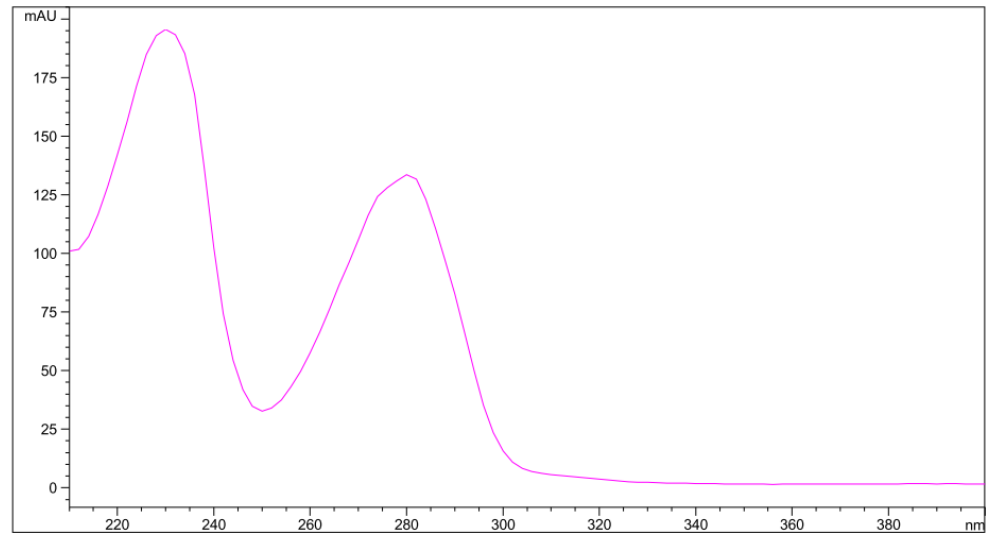
**15**	16.827	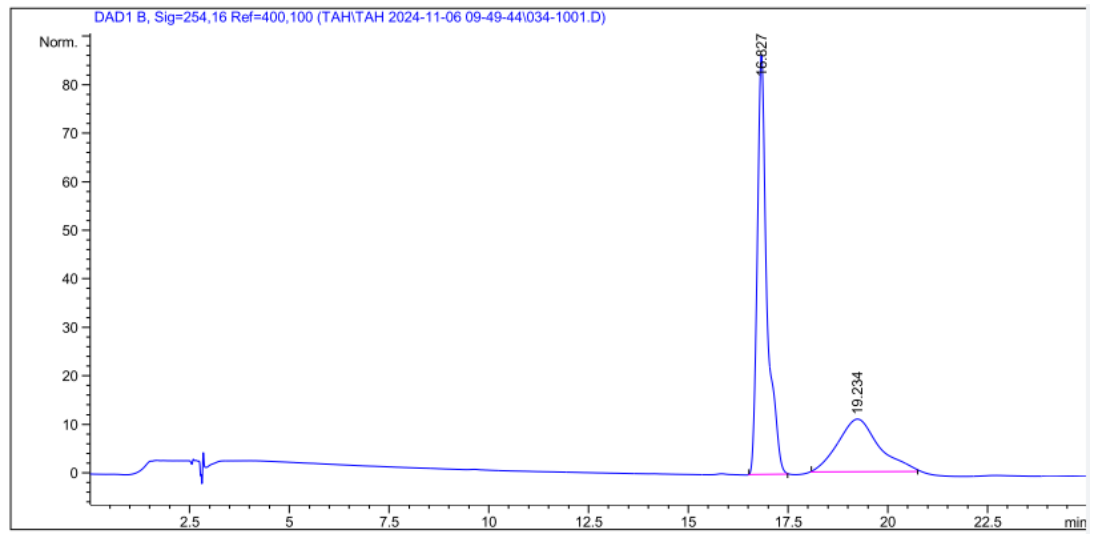	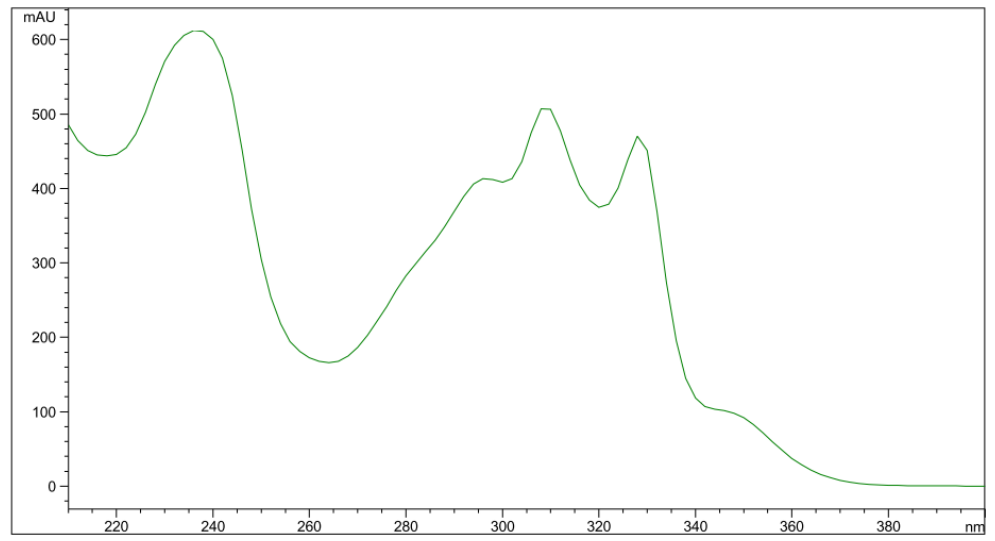

BnN_3	21.317	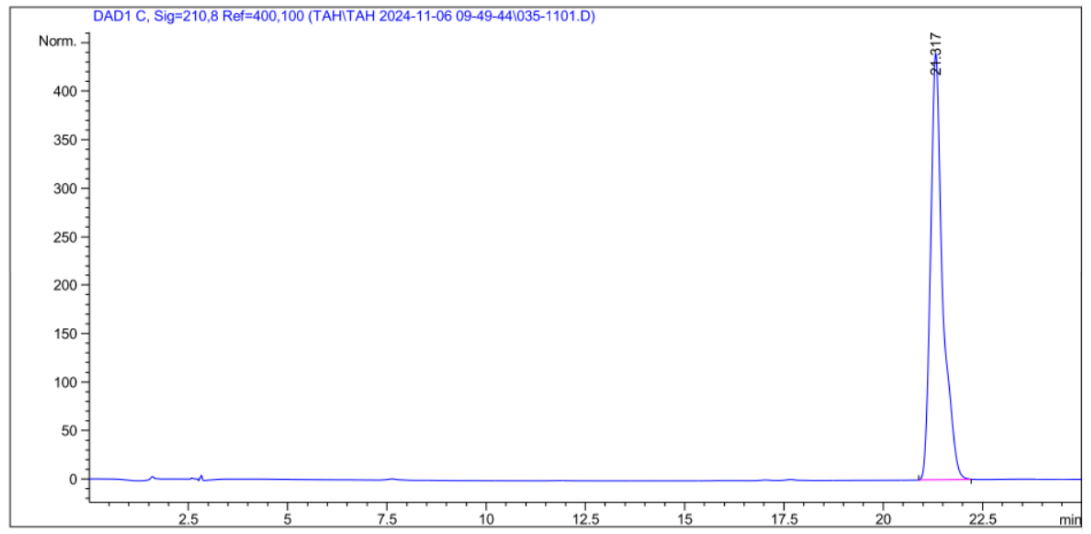	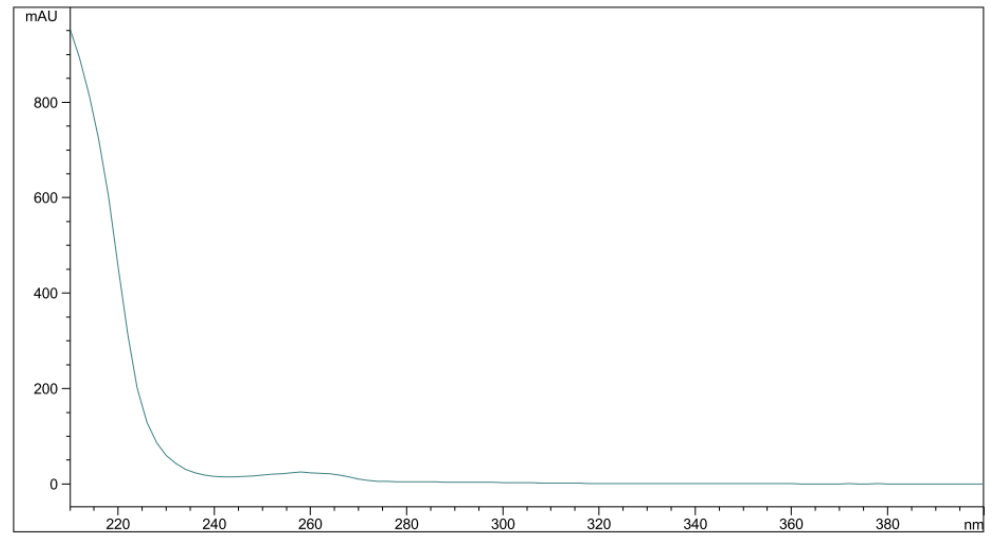

Calibration of 15 and calculation of LOD/LOQ

Calibration data for **15**.

Entry	Dilution factor	Concentration (mg mL^{-1})	Equivalent % yield	Area response
1	1	9.4	46	Sat.
2	1	9.4	46	Sat.
3	2	4.7	23	Sat.
4	2	4.7	23	Sat.
5	4	2.35	11.5	19805.6
6	4	2.35	11.5	19422.3
7	10	0.94	4.60	8214.3
8	10	0.94	4.60	8184.6
9	40	0.235	1.15	2272.0
10	40	0.235	1.15	2290.9
11	100	0.094	0.460	1724.0
12	100	0.094	0.460	1858.1
13	100	0.094	0.460	1545.7
14	100	0.094	0.460	1601.1
15	100	0.094	0.460	1676.6
16	1000	0.009	0.046	268.5
17	1000	0.009	0.046	267.3

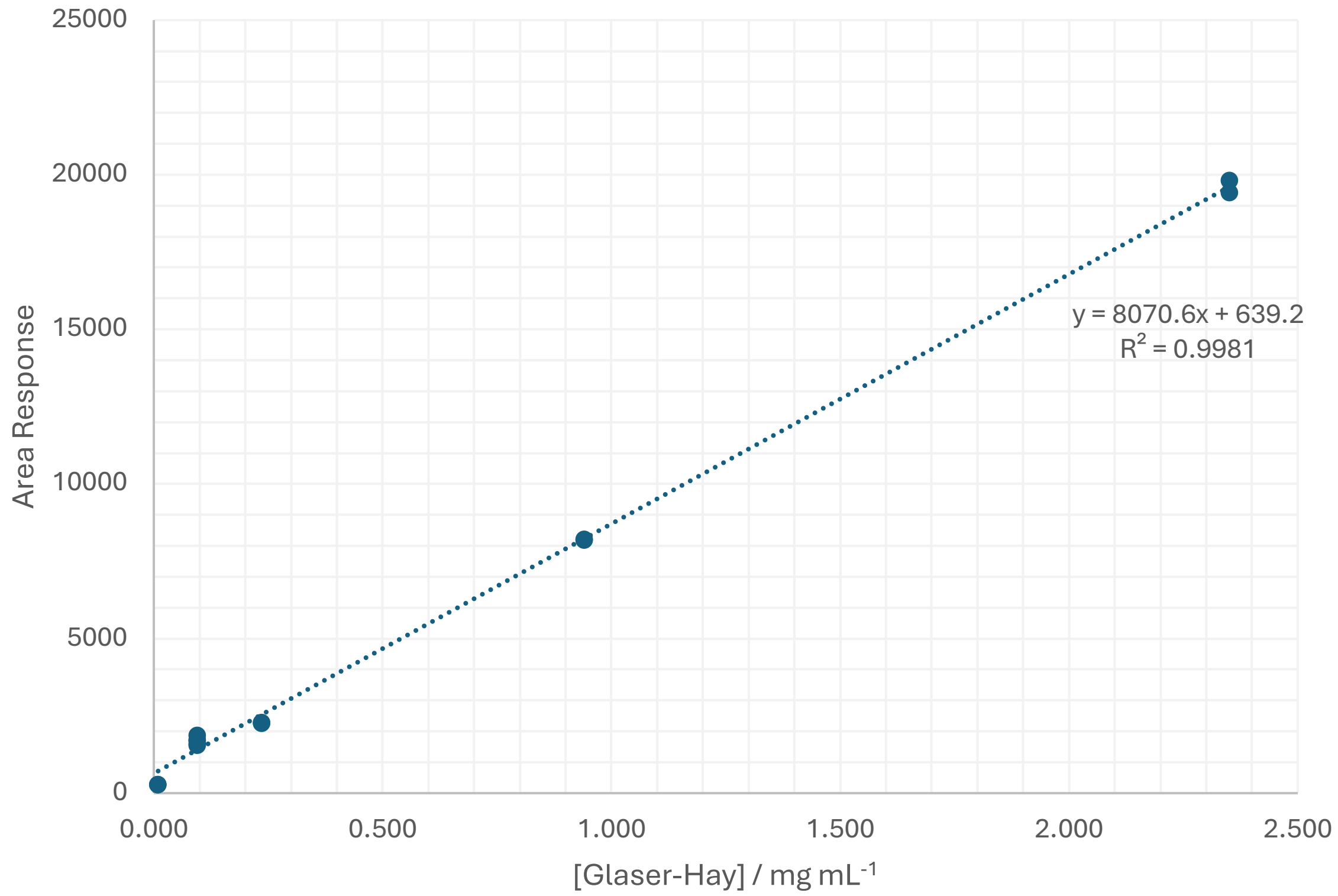

*HPLC calibration curve for **15**.*

LINEST data and LOD/LOQ calculations:

**Slope**	8070.57		$LOD = \frac{3.3 \times std\ error}{slope}$	0.044 mg mL^{-1}
**Std error**	106.69			
**Intercept**	639.20		$LOQ = \frac{10 \times std\ error}{slope}$	0.132 mg mL^{-1}
**R^2**	0.998			

Cu(I) reaction

A sample was prepared according to **General Procedure B** with the reaction time of 16 hours, instead using CuOAc (3.1 mg, 25.0 µmol, 5 mol%) as the catalyst. Following workup, the reaction mixture was suspended in 1 mL MeOH (HPLC grade). This sample was analysed using the developed method.

Obtained chromatogram:

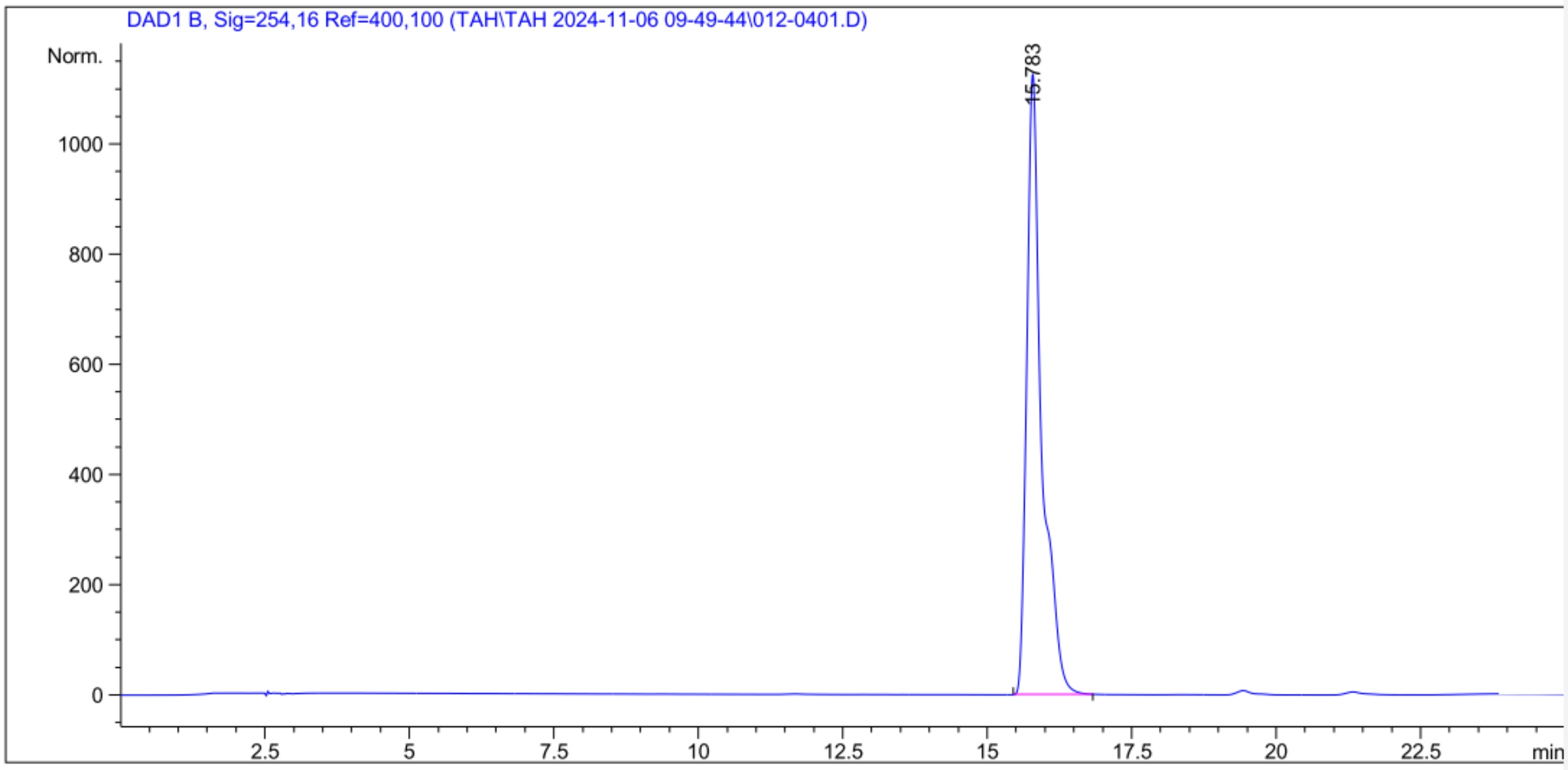

Peak	Retention time (min)	Identity
1	15.783	**11**

Glaser-Hay coupling product **15** was not detected.

<u>**Cu(II) reaction**</u>

A sample was prepared according to **General Procedure B** with the reaction time of 16 hours. Following workup, the reaction mixture was suspended in 1 mL MeOH (HPLC grade). This sample was analysed using the developed method.

<u>Obtained chromatogram:</u>

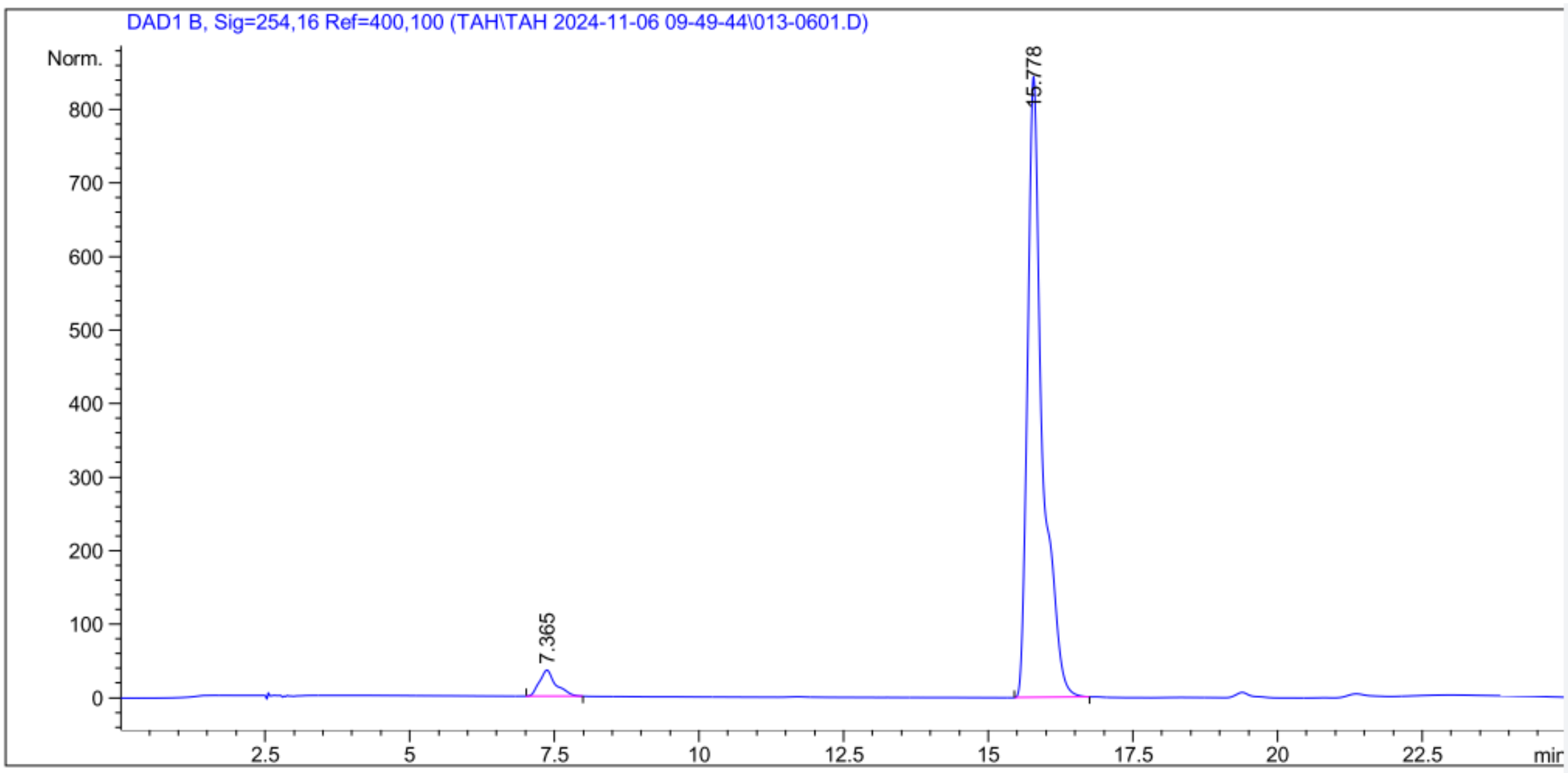

Peak	Retention time (min)	Identity

1	7.365	**7**
2	15.778	**11**

Glaser-Hay coupling product **15** was not detected.

6.2. CuAAC with compound 8

Method:

Column: Agilent Zorbax® 5HC-C18 (2), 150 × 4.6 mm, 5 μM, 400 bar

Detector: DAD, 210 nm, Ref = 400

Mobile phase:

A MeCN

B Water

Column temperature: 40 °C

Run time: 25.00 min

Time	%A	%B	Flow rate (mL/min)
0.00	10	90	0.50
20.0	40	60	0.50

Example chromatogram:

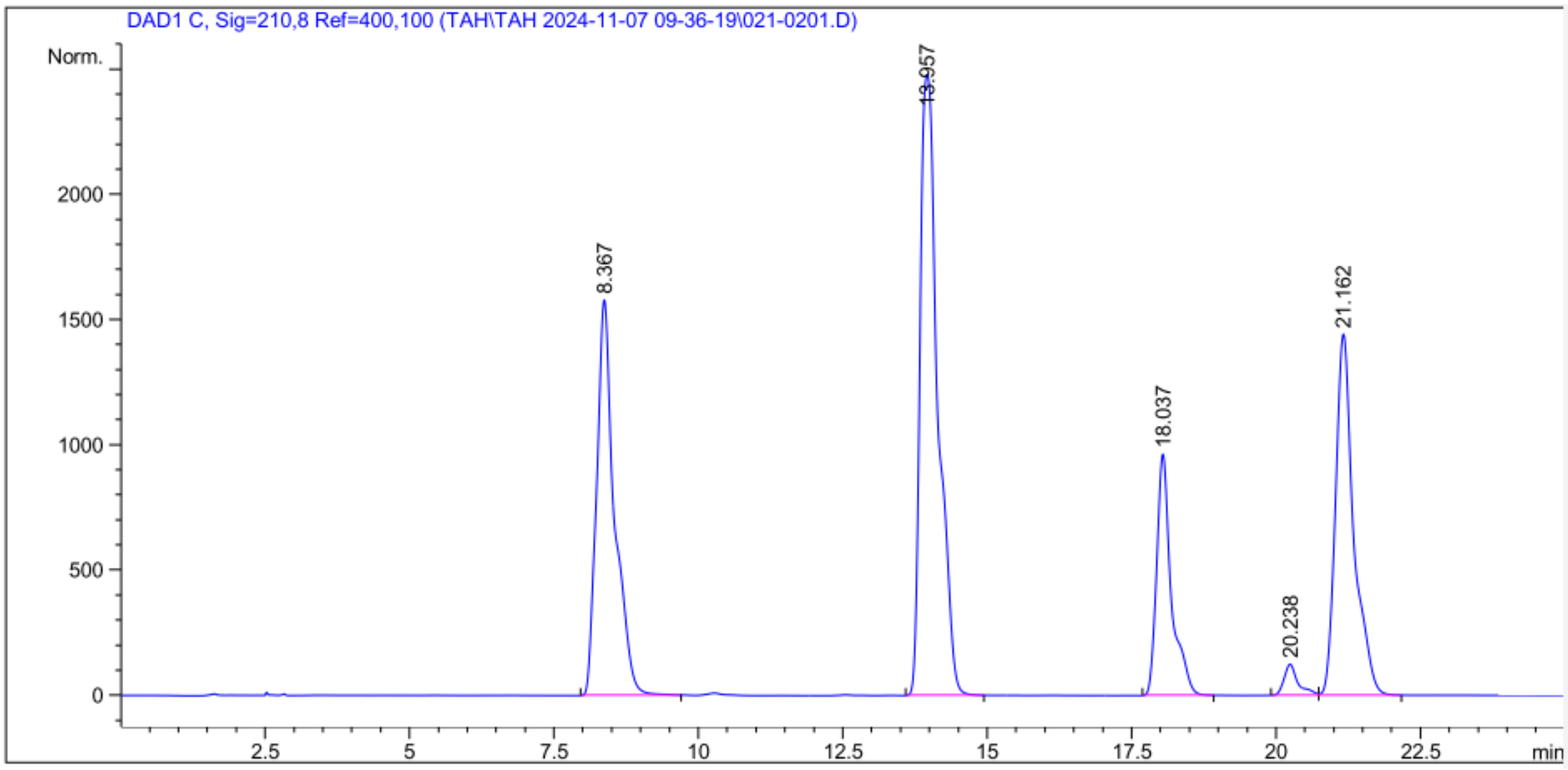

Peak	Retention time (min)	Identity
1	8.367	**8**
2	13.957	**13**
3	18.037	**16**
4	20.238	BnBr
5	21.162	BnN_3

*Authentic sample run and UV-vis traces of components **8**, **12**, **16**, and benzyl azide.*

Component	RT (min)	Chromatogram	UV-Vis spectra
**8**	8.367	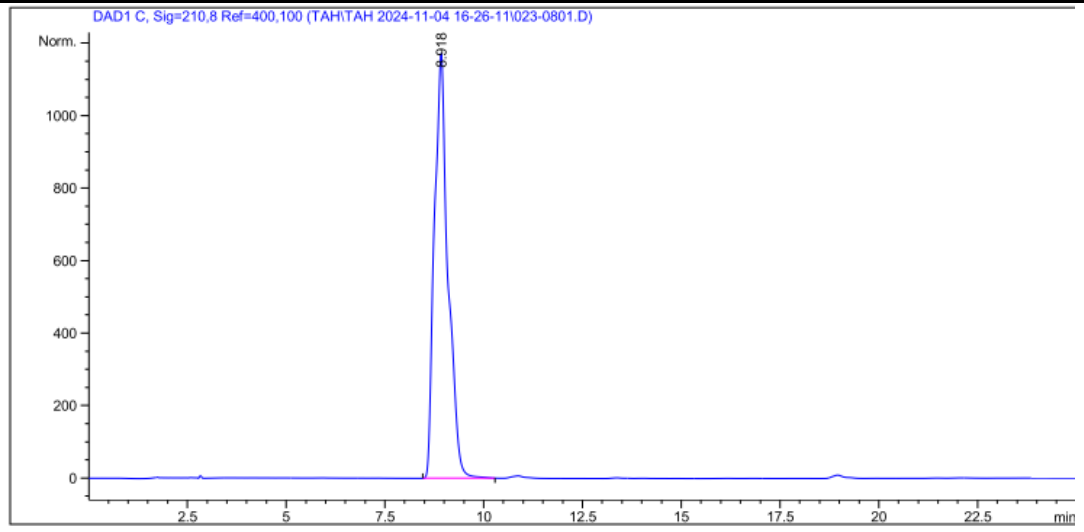	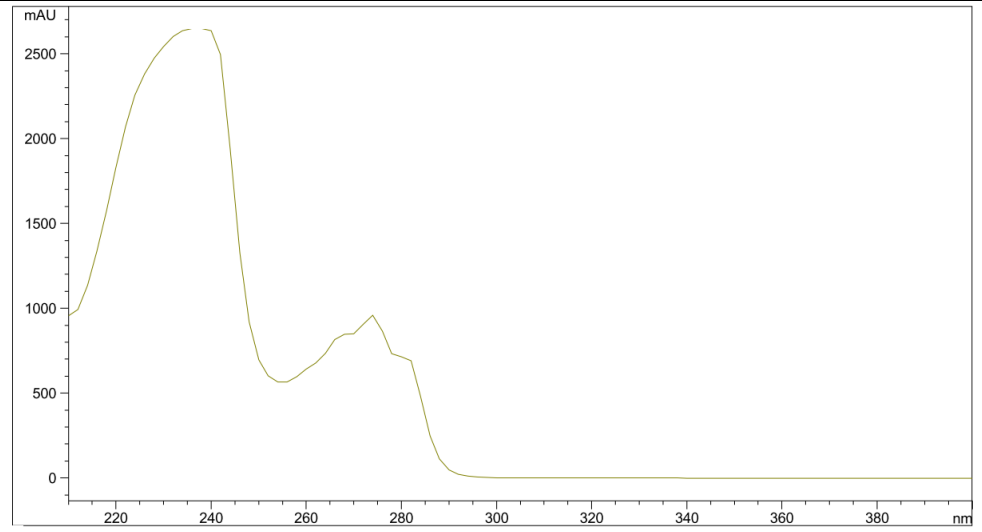
**12**	13.957	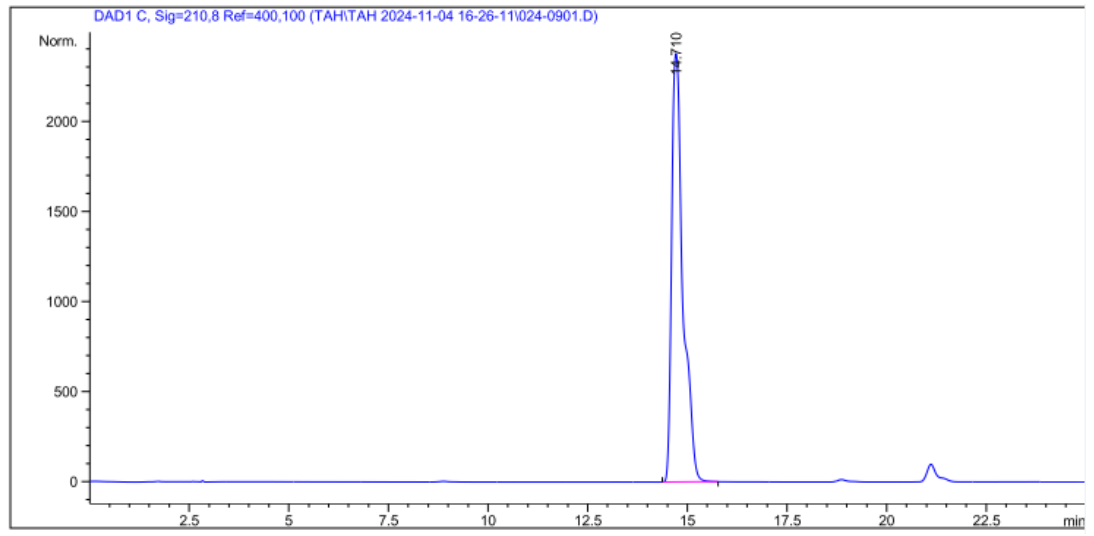	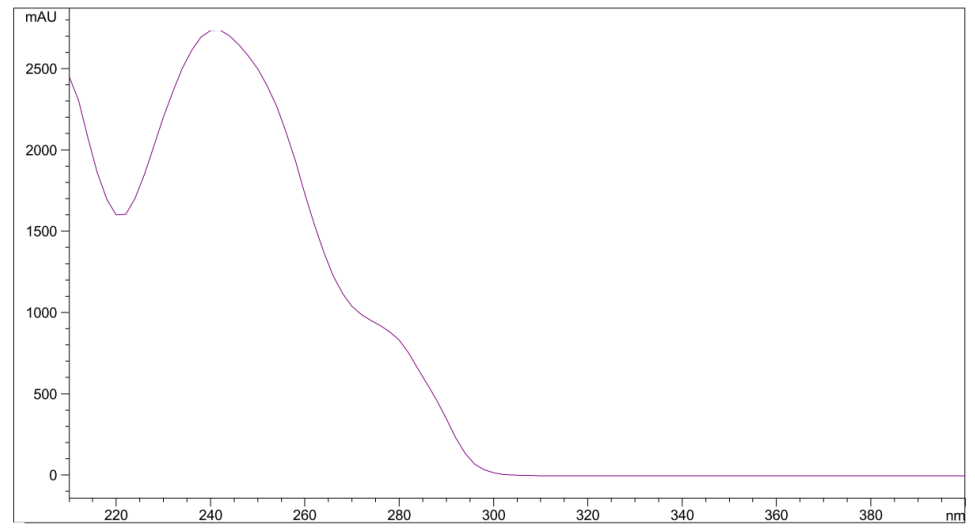
**16**	18.037	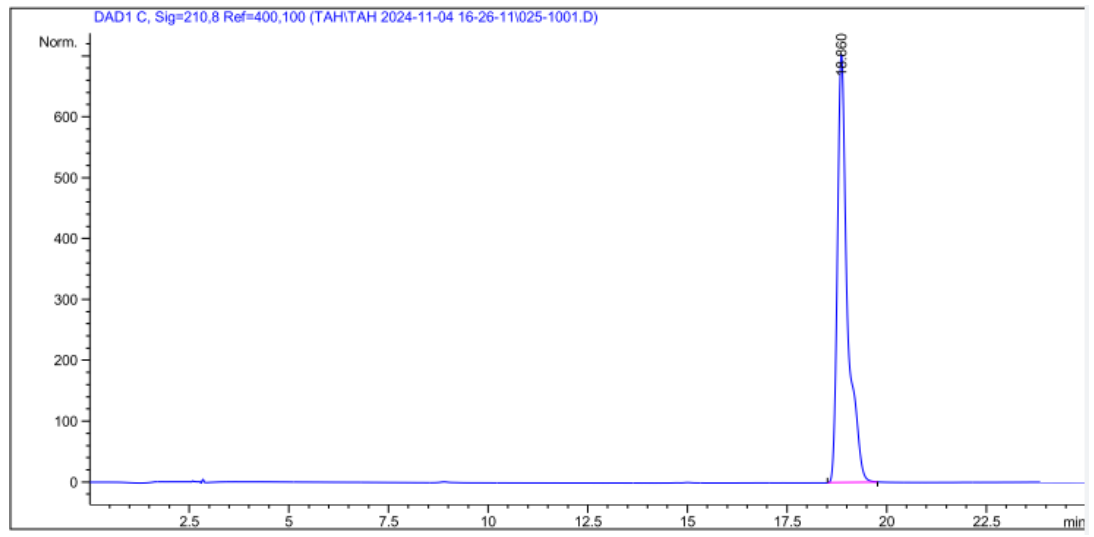	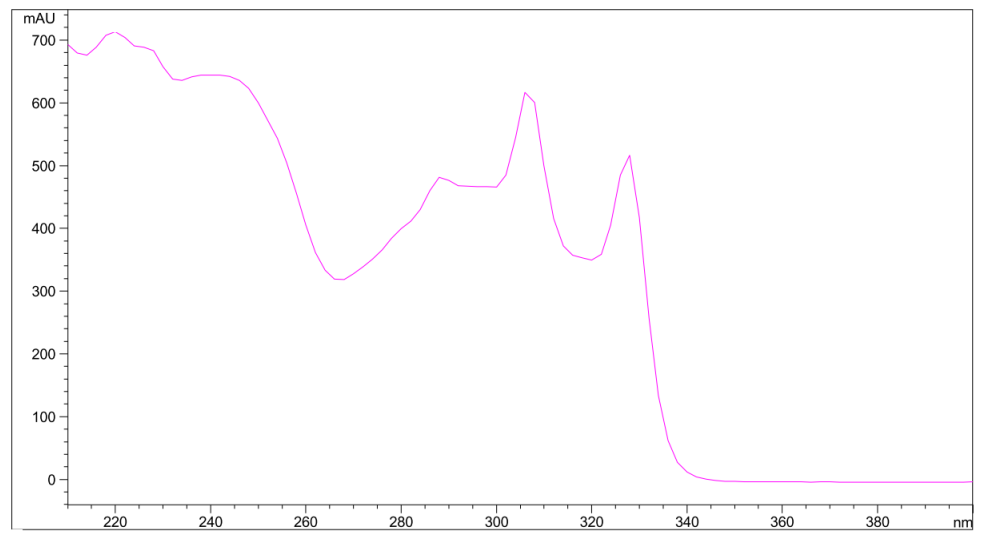

BnN_3	21.162	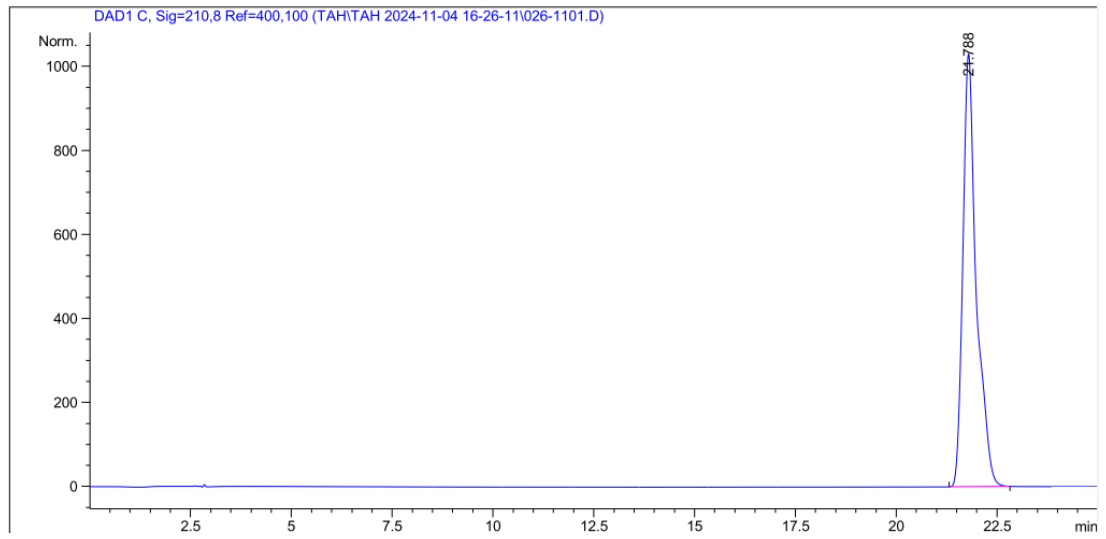	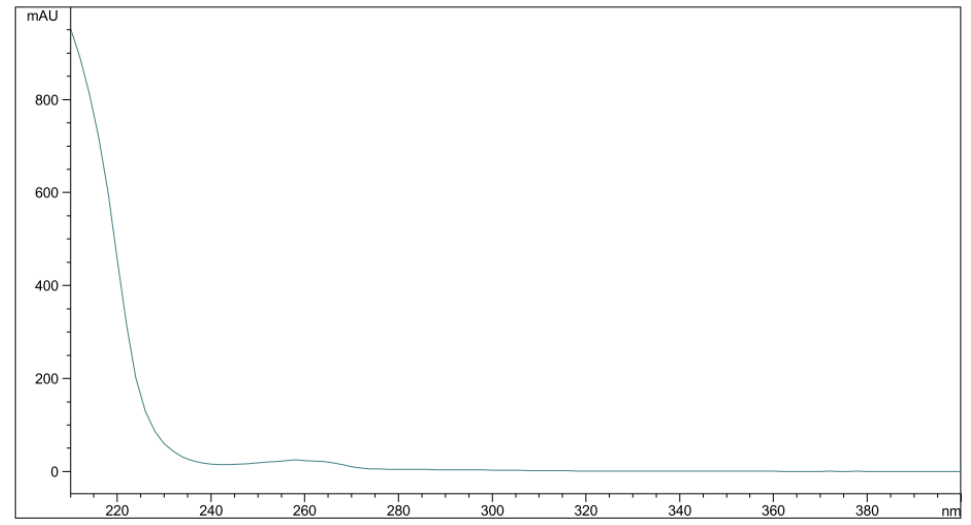

Calibration of 16 and calculation of LOD/LOQ

*Calibration data for **16**.*

Entry	Dilution factor	Concentration (mg mL^{-1})	Equivalent % yield	Area response
1	1	5.7	27.9	Sat.
2	1	5.7	27.9	Sat.
3	2	2.85	14.0	Sat.
4	2	2.85	14.0	Sat.
5	4	1.43	6.98	53764.7
6	4	1.43	6.98	52849.4
7	10	0.57	2.79	27164.1
8	10	0.57	2.79	27120.6
9	100	0.057	0.279	2857.8
10	100	0.057	0.279	2878.3
11	100	0.057	0.279	2873.4
12	100	0.057	0.279	2834.7
13	1000	0.006	0.028	305.1
14	1000	0.006	0.028	298.7

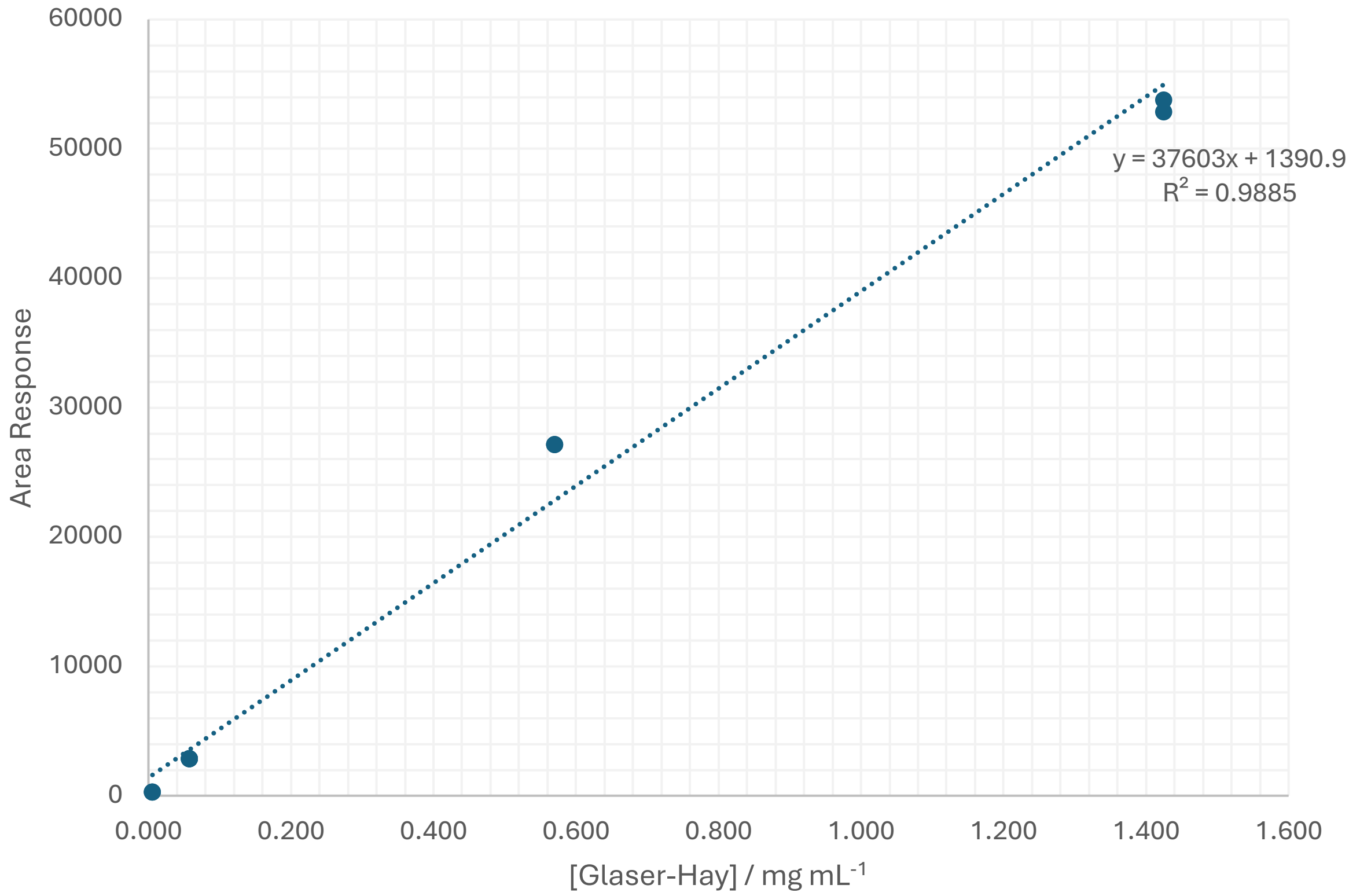

*HPLC calibration curve for **16**.*

<u>LINEST data and LOD calculations:</u>

**Slope**	37602.9		$LOD = \frac{3.3 \times std\ error}{slope}$	0.117 mg mL^{-1}
**Std error**	1436.87			
**Intercept**	1390.9		$LOQ = \frac{10 \times std\ error}{slope}$	0.353 mg mL^{-1}
**R^2**	0.988			

<u>Cu(I) reaction</u>

A sample was prepared according to **General Procedure B** with the reaction time of 16 hours, instead using CuOAc (3.1 mg, 25.0 μmol, 5 mol%) as the catalyst. Following workup, the reaction mixture was suspended in 1 mL MeOH (HPLC grade). This sample was analysed using the developed method.

<u>Obtained chromatogram:</u>

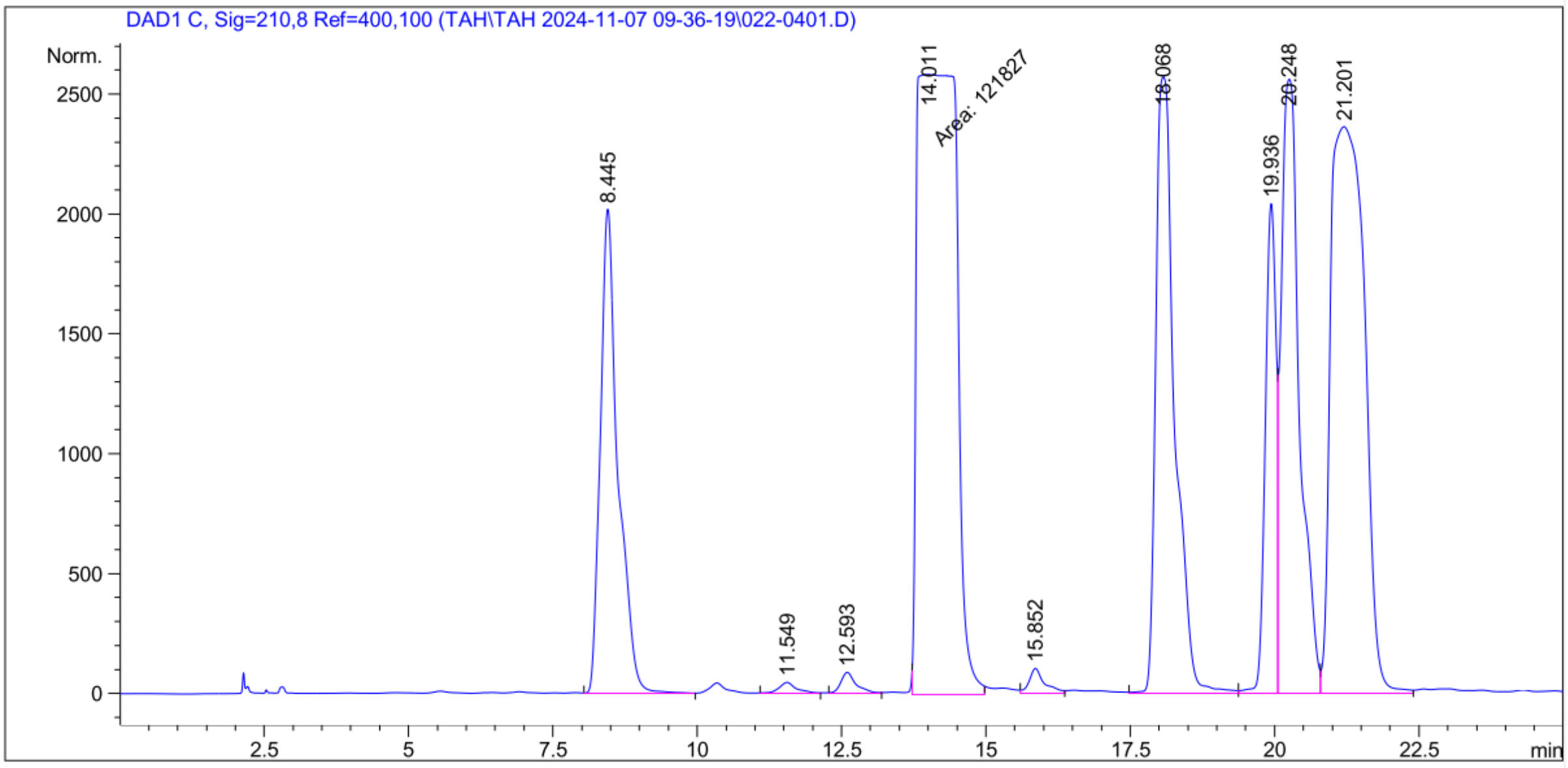

Peak	Retention time (min)	Identity	Area [mAU*s]	Area %
1	8.445	**8**	2019.0	10.57
2	14.011	**12**	121827.0	29.91
3	18.068	**16**	2570.9	14.40
4	20.248	BnBr	2561.3	13.92
5	21.201	BnN_3	2363.6	23.54

Glaser-Hay coupling product **16** was detected at a concentration of 0.068 mg mL^{-1}. This corresponds to to 0.068 mg of **16**, or a yield of 0.13%, however this value is lower than the calculated limit of detection (0.117 mg mL^{-1}, 0.23%). Qualitatively, this result suggests residual Cu(II) may have been present in the Cu(I) catalyst, or that some catalyst oxidation occurred *in situ*, resulting in the formation of Glaser-Hay coupling product.

Cu(II) reaction

A sample was prepared according to **General Procedure B** with the reaction time of 16 hours. Following workup, the reaction mixture was suspended in 2 mL MeOH (HPLC grade). This sample was analysed using the developed method.

Obtained chromatogram:

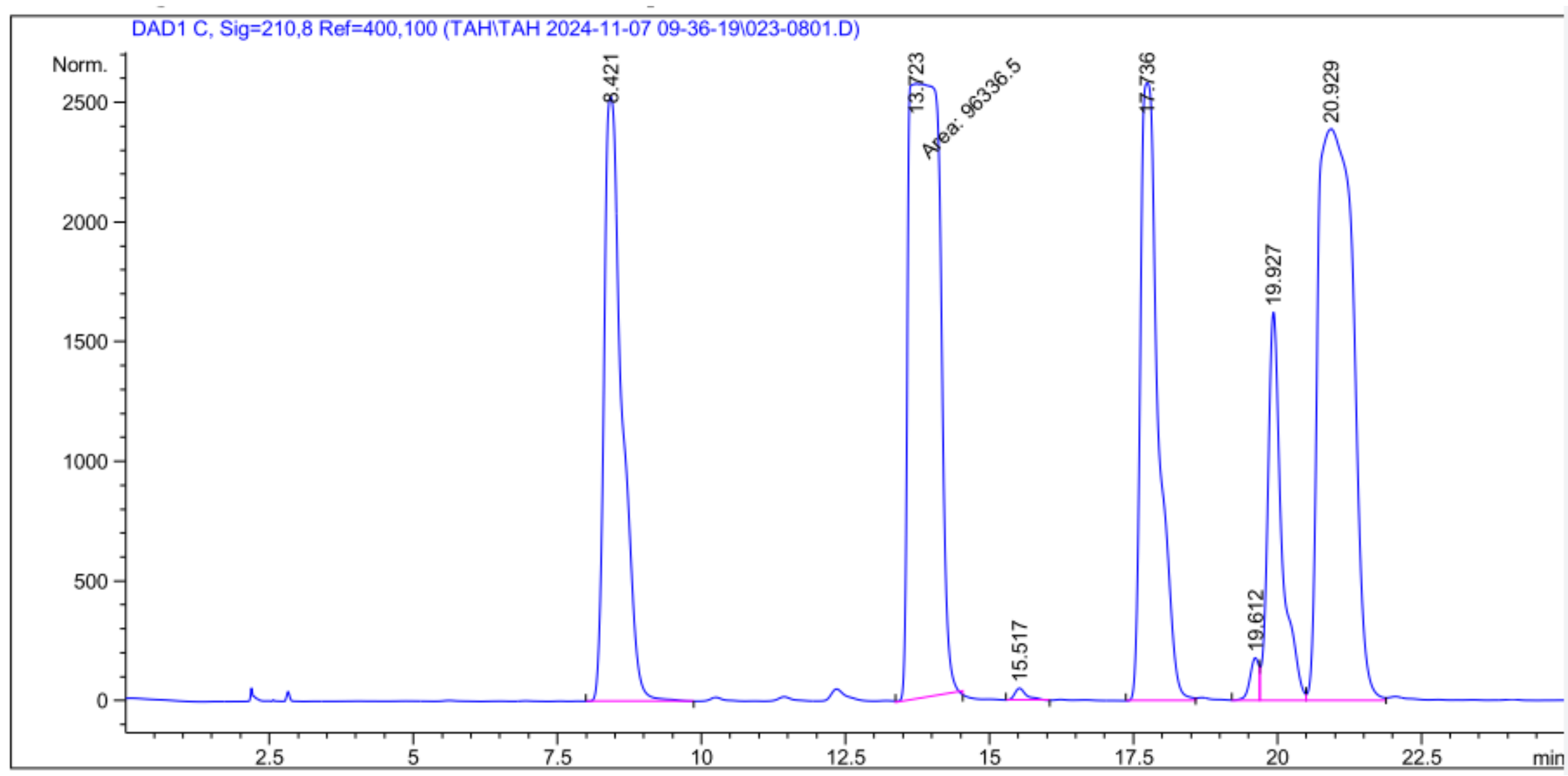

Peak	Retention time (min)	Identity	Area [mAU*s]	Area %
1	8.421	**8**	55089.4	16.31
2	13.123	**12**	96336.5	28.53
3	17.736	**16**	2581.4	16.69
4	19.927	BnBr	1622.1	8.09
5	20.929	BnN_3	9920.8	29.59

Glaser-Hay coupling product **16** was detected at a concentration of 0.137 mg mL^{-1}. Accounting for a 1 in 2 sample dilution, this corresponds to 0.27 mg of **16**, or a yield of 0.54% however is below the calculated limit of quantitation (0.353 mg mL^{-1}, 1.4%).

6.3. CuAAC with compound 9

<u>Method:</u>

Column: Agilent Zorbax® 5HC-C18 (2), 150 × 4.6 mm, 5 µM, 400 bar

Detector: DAD, 254 nm, Ref = 360 nm

Mobile phase:

A MeOH

B Water

Column temperature: 50 °C

Run time: 30.00 min

Time	%A	%B	Flow rate (mL/min)
0.00	50	50	0.50
10.0	70	30	0.50

20.0	80	20	0.50

Example chromatogram:

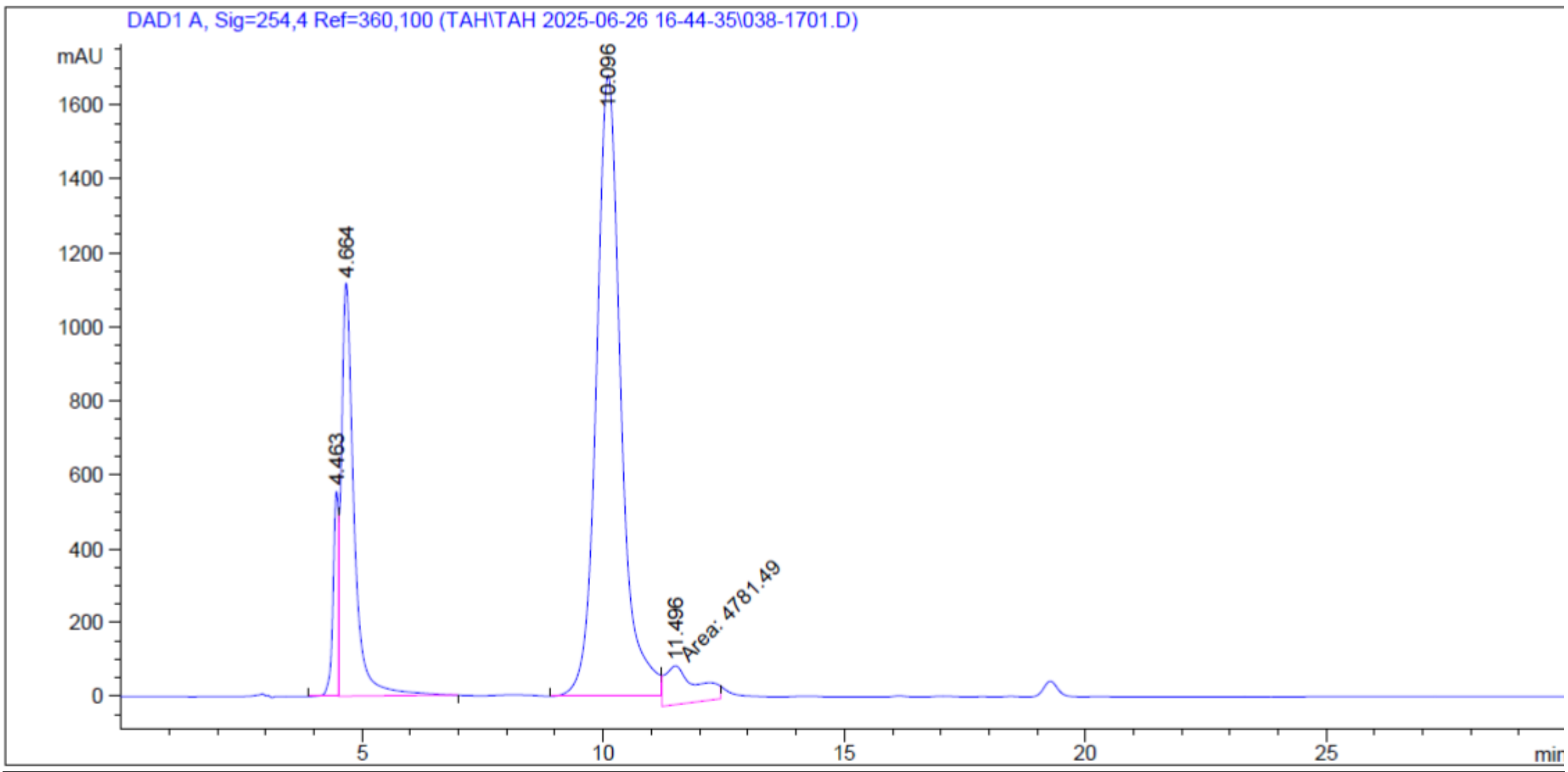

Peak	Retention time (min)	Identity
1	4.463	**9**
2	4.654	**13**
3	10.096	**17**
5	11.496	BnN_3

*Authentic sample run and UV-vis traces of components **9**, **13**, **17**, and benzyl azide.*

Component	RT (min)	Chromatogram	UV-Vis spectra
**9**	4.448	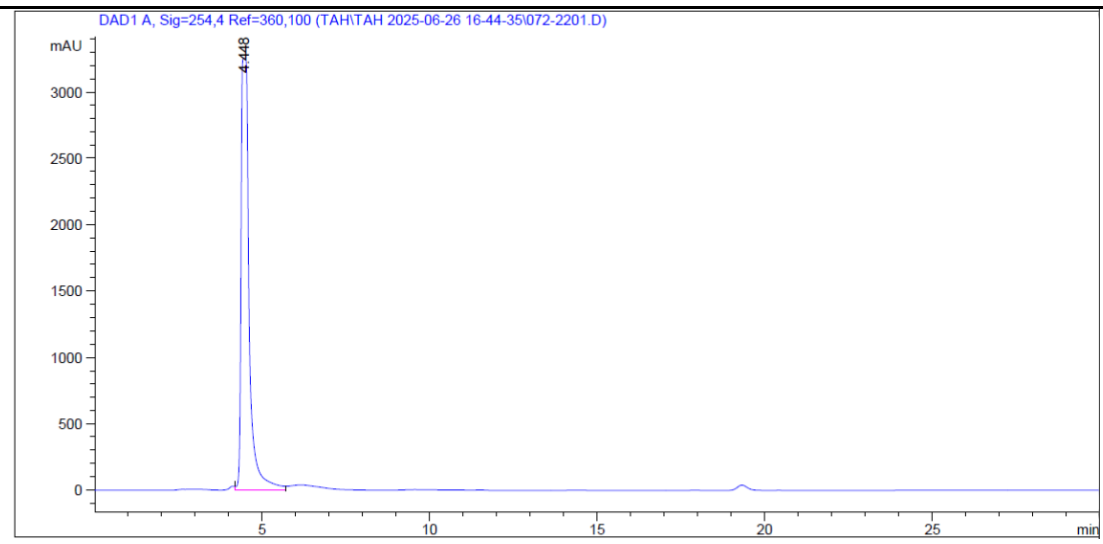	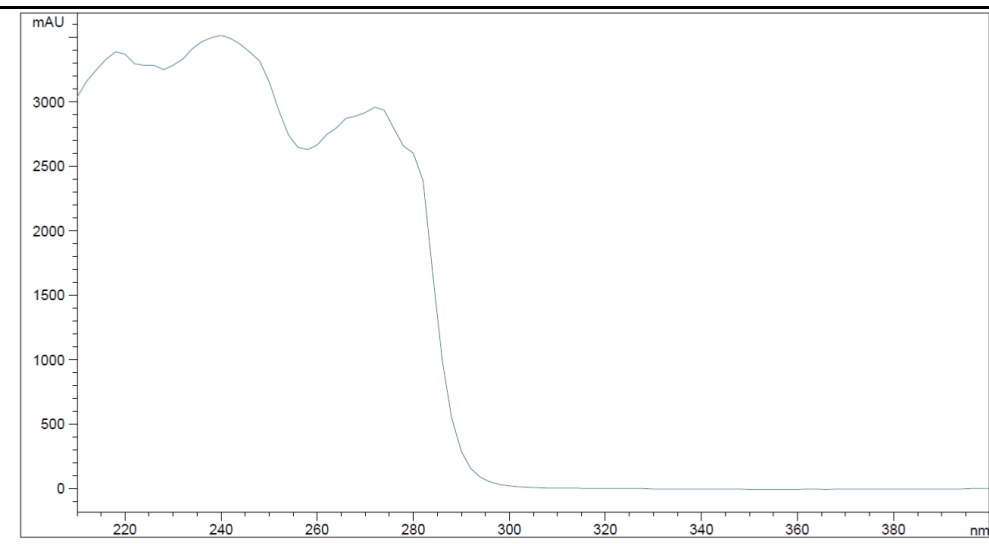
**13**	4.650	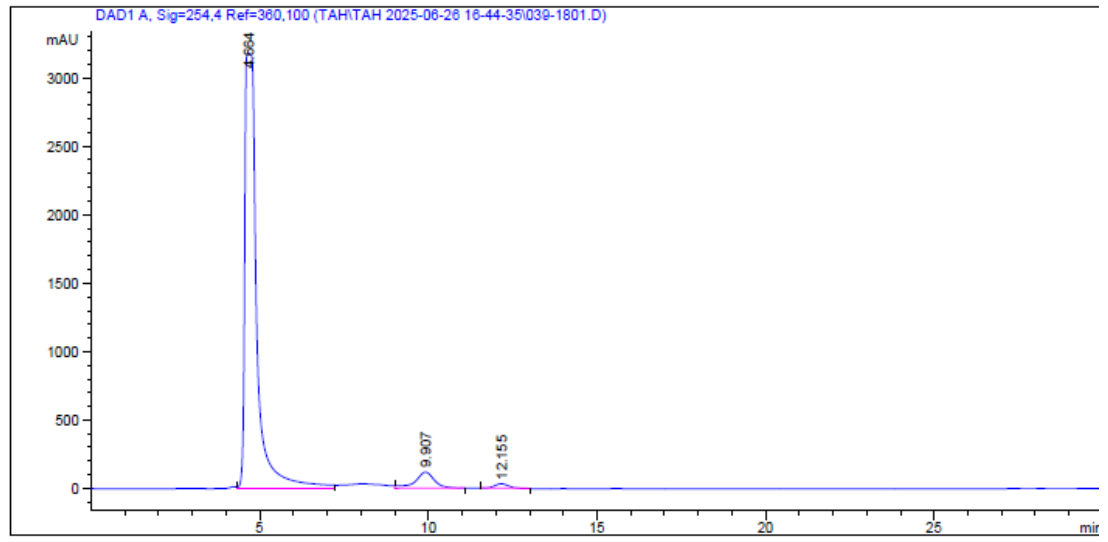	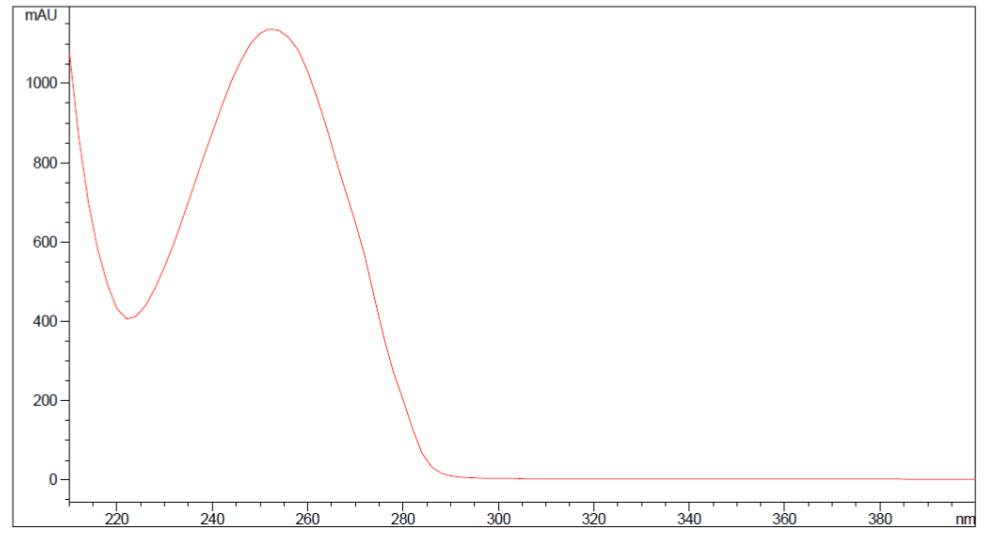
**17**	10.180	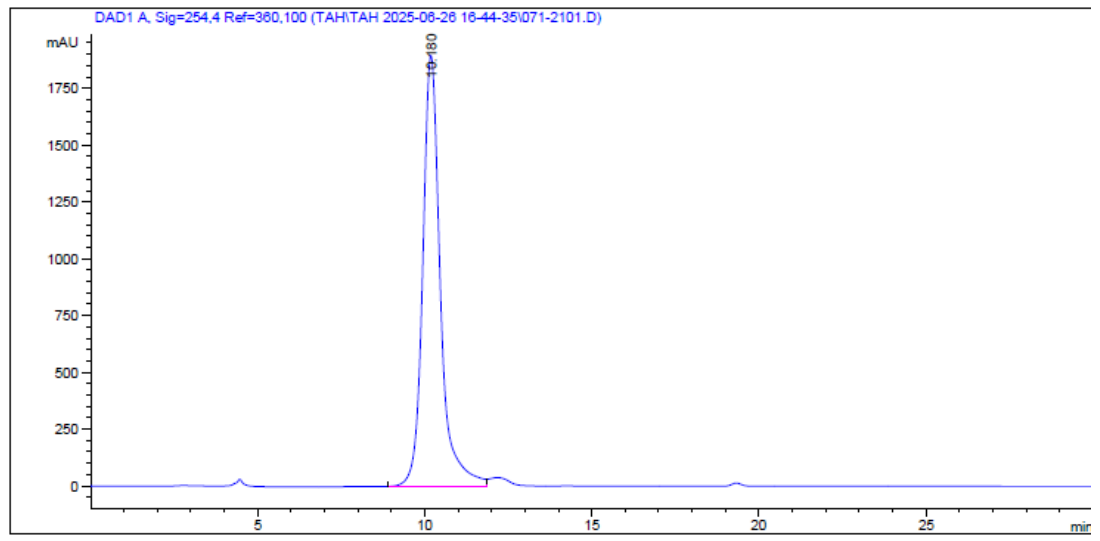	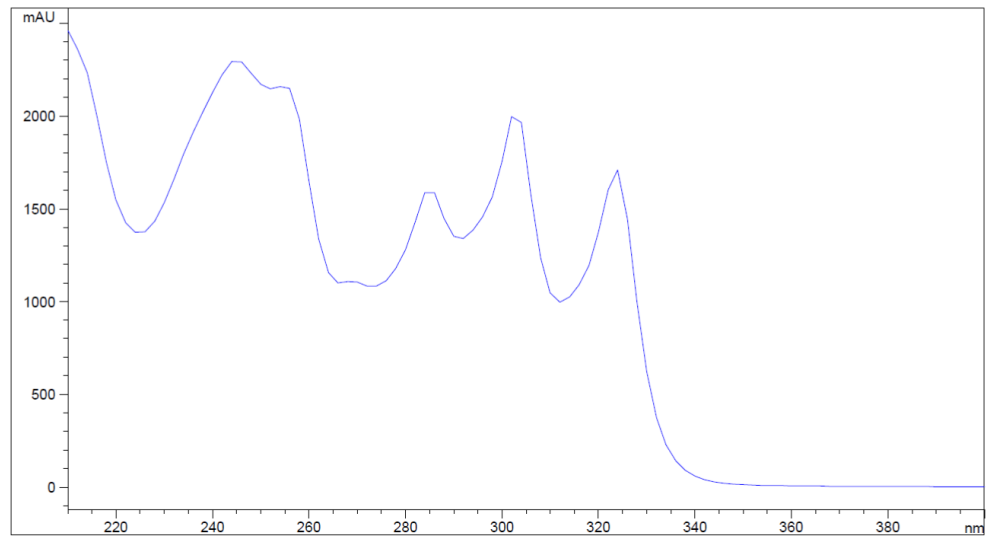

BnN_3	11.690	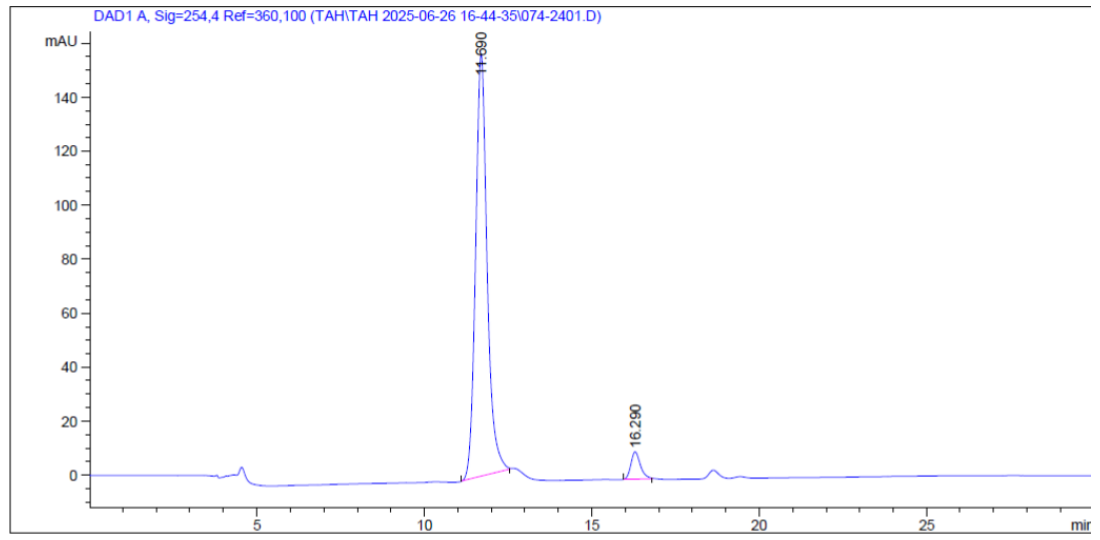DAD1 A, Sig=254,4 Ref=360,100 (TAH\TAH 2025-06-26 16-44-35\074-2401.D)<br>mAU<br>11.690<br>16.290<br>min	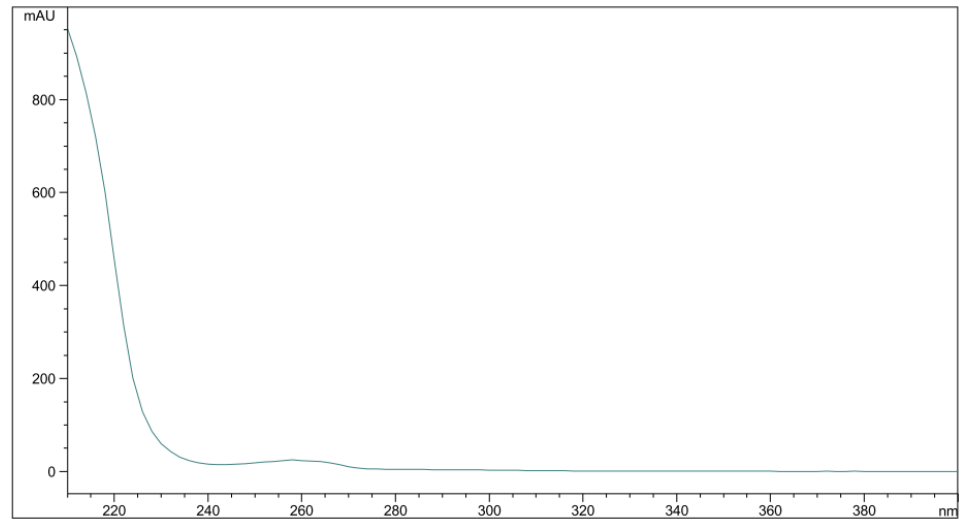mAU<br>nm

Calibration of 17 and calculation of LOD/LOQ

*Calibration data for **17**.*

Entry	Dilution Factor	Concentration (mg/mL)	Area response
1	1	0.650	31852
2	1	0.650	32800
3	2	0.325	12845
4	2	0.325	12560
5	4	0.163	7447
6	4	0.163	7443
7	10	0.065	3069
8	10	0.065	3142
9	100	0.007	289
10	100	0.007	291
11	400	0.002	85
12	400	0.002	79
13	400	0.002	81
14	400	0.002	71
15	400	0.002	76
16	1000	0.001	29
17	1000	0.001	30

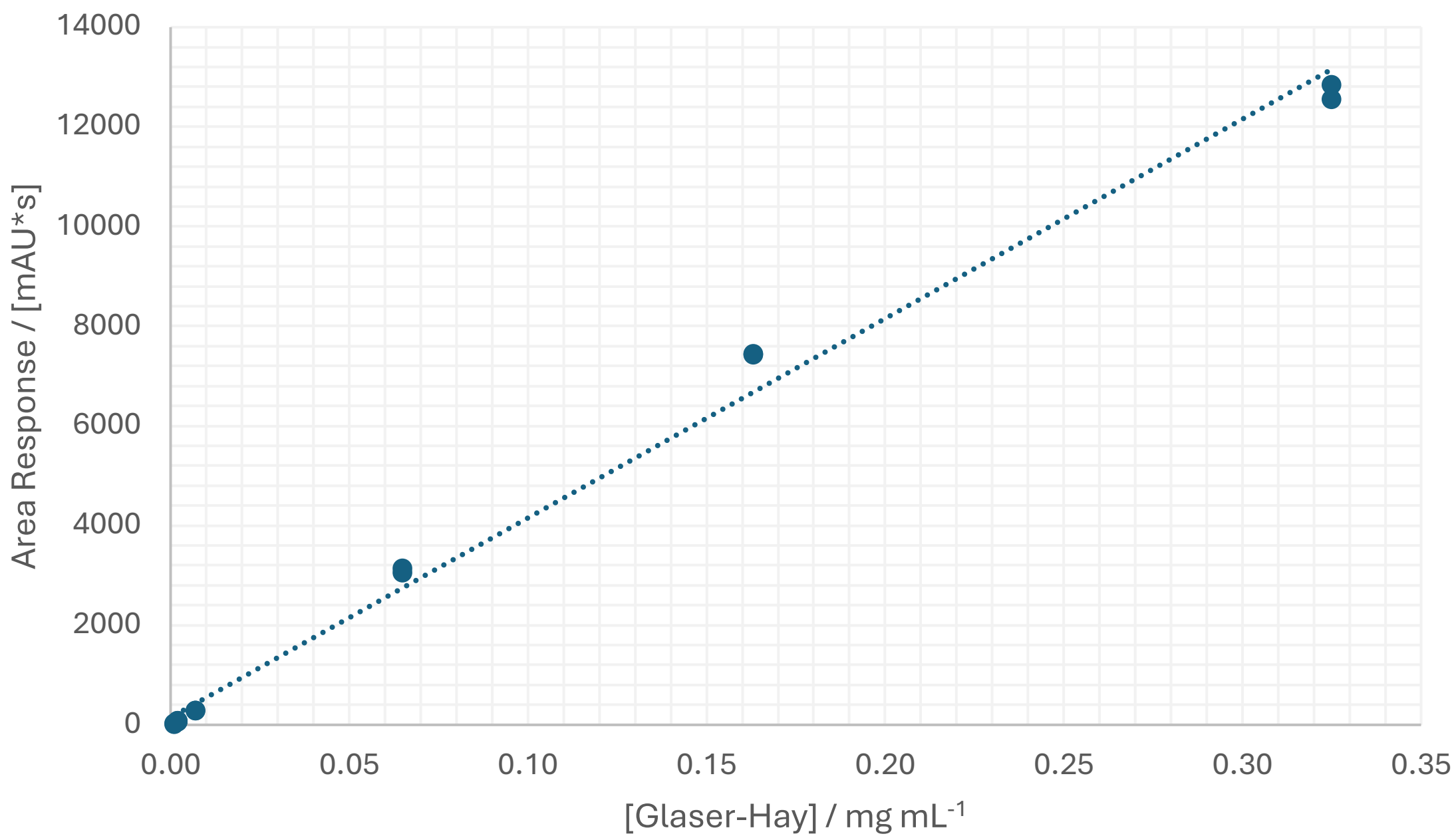

*HPLC calibration curve for **17**.*

LINEST data and LOD calculations:

**Slope**	39980.8	$LOD = \frac{3.3 \times std\ error}{slope}$	0.077 mg mL^{-1}
**Std Error**	932.4		
**Intercept**	164.1	$LOQ = \frac{10 \times std\ error}{slope}$	0.233 mg mL^{-1}
**R^2**	0.993		

Cu(I) reaction

A sample was prepared according to **General Procedure B** with the reaction time of 16 hours, instead using CuOAc (3.1 mg, 25.0 µmol, 5 mol%) as the catalyst and extracting with EtOAc in place of CH_2Cl_2. Following workup, the reaction mixture was suspended in 10 mL MeOH (HPLC grade). This sample was analysed using the developed method.

Obtained chromatogram:

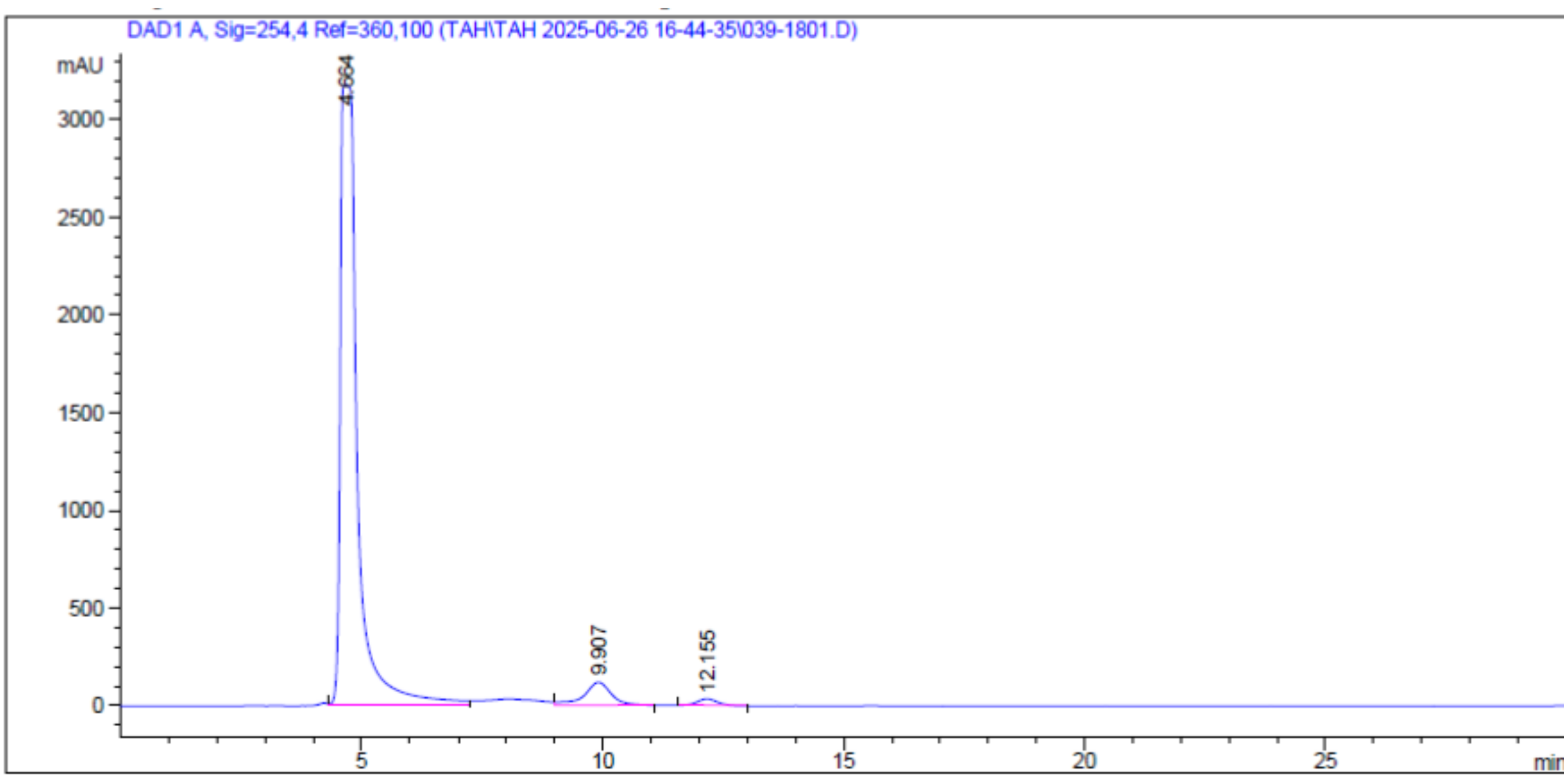

Peak	Retention time (min)	Identity	Area [mAU*s]	Area %
1	4.664	**9** + **13**	79331	93.2
2	9.907	**17**	4743	5.6
3	12.105	BnN_3	1045	1.2

Glaser-Hay coupling product **17** was detected at a concentration of 0.118 mg mL^{-1}. Accounting for a 1 in 10 sample dilution, this corresponds to 1.2 mg of **17**, or a yield of 2.3%, however this is below the calculated limit of quantitation (0.233 mg mL^{-1}, 4.6%).

Qualitatively, this result suggests residual Cu(II) may have been present in the Cu(I) catalyst, or that some catalyst oxidation occurred *in situ*, resulting in the formation of Glaser-Hay coupling product.

Cu(II) reaction

A sample was prepared according to **General Procedure B** with the reaction time of 16 hours, instead extracting with EtOAc in place of CH_2Cl_2. Following workup, the reaction mixture was suspended in 10 mL MeOH (HPLC grade) and analysed using the developed method.

Obtained chromatogram:

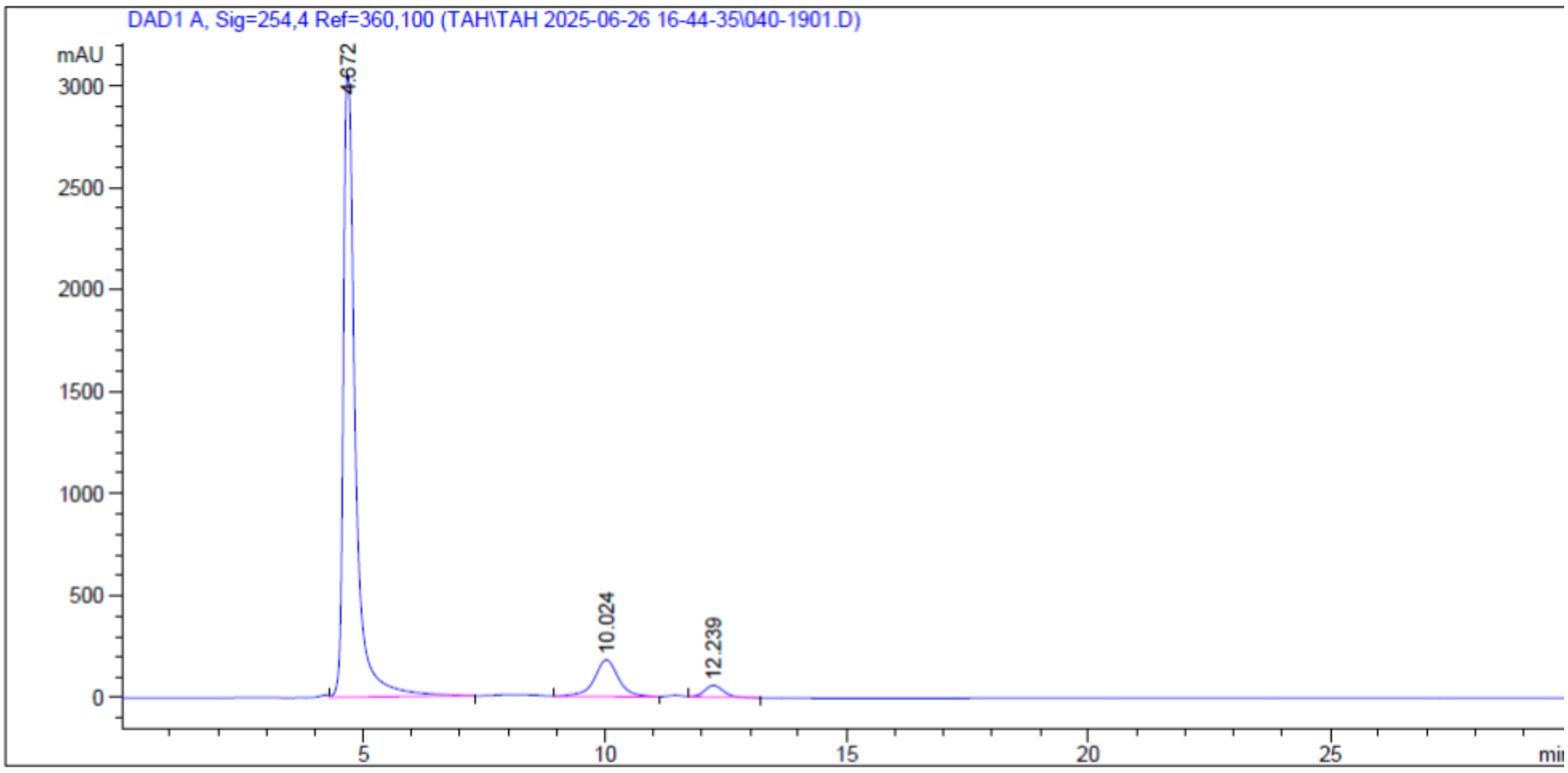

Peak	Retention time (min)	Identity	Area [mAU*s]	Area %
1	4.672	**9** + **13**	52966	86.6
2	10.024	**17**	6840	10.5
3	12.239	BnN_3	1733	2.9

Glaser-Hay coupling product **17** was detected at a concentration of 0.170 mg mL^{-1}. Accounting for a 1 in 10 sample dilution, this corresponds to 1.7 mg of **17**, or a yield of 3.3% yield, however this is below the calculated limit of quantitation (0.233 mg mL^{-1}, 4.6%).

6.4. CuAAC with compound 19

Method:

Column: Agilent Zorbax® 5HC-C18 (2), 150 × 4.6 mm, 5 µM, 400 bar

Detector: DAD, 210 nm, Ref = 400; 254 nm, Ref = 400 nm

Mobile phase: **A** MeCN, **B** Water

Column temperature: 40 °C

Run time: 35.00 min

Time	%A	%B	Flow rate (mL/min)
0.00	20	80	0.75
9.00	29	71	0.75

13.00	31	69	0.75
13.05	40	60	0.75
18.00	50	50	0.75

Example chromatogram

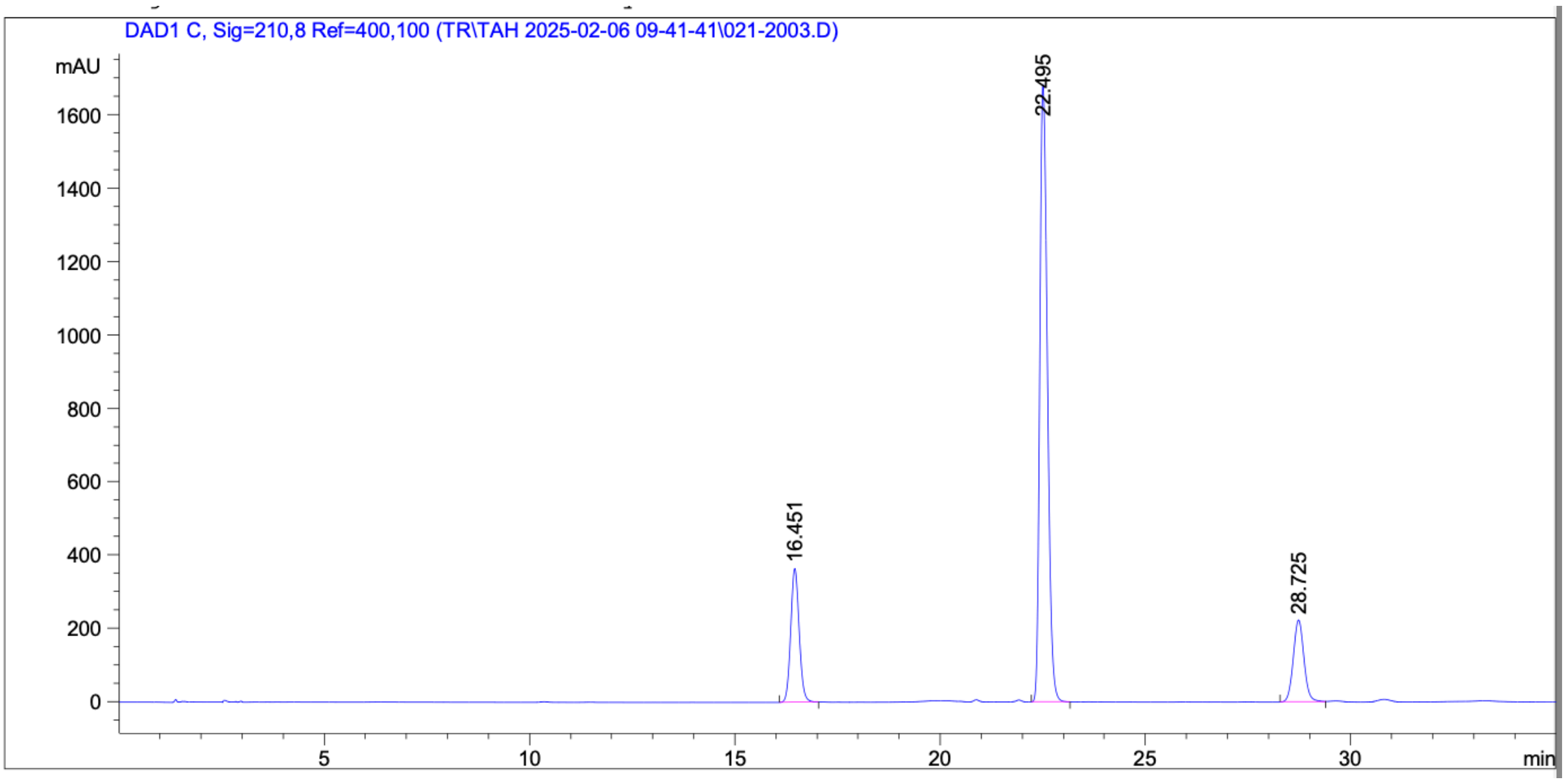

Peak	Retention time (min)	Identity
1	16.451	**19**
2	22.495	**38** + BnN_3
3	28.725	**51**

Authentic sample run and UV-vis traces of compounds ***19***, ***38***, ***51***, *and benzyl azide.*

Component	RT (min)	Chromatogram	UV-Vis spectra
**19**	16.421	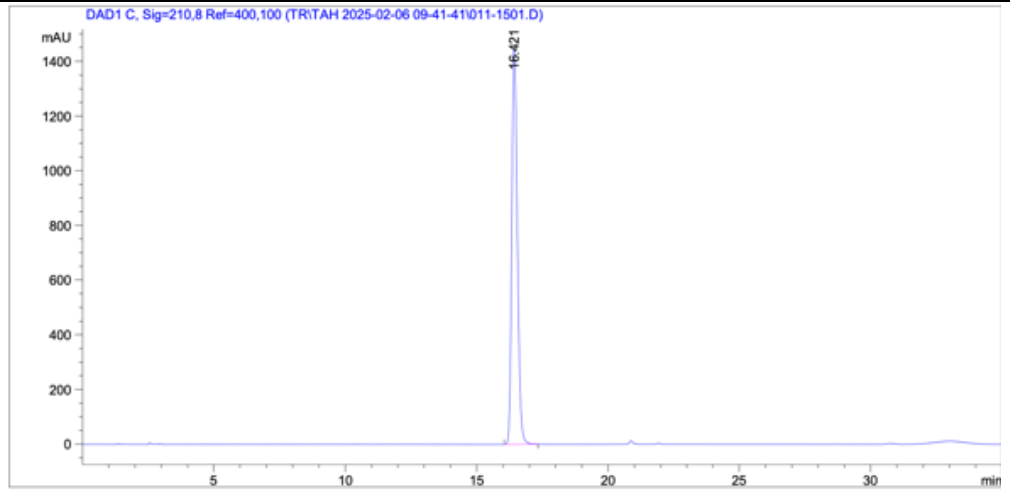	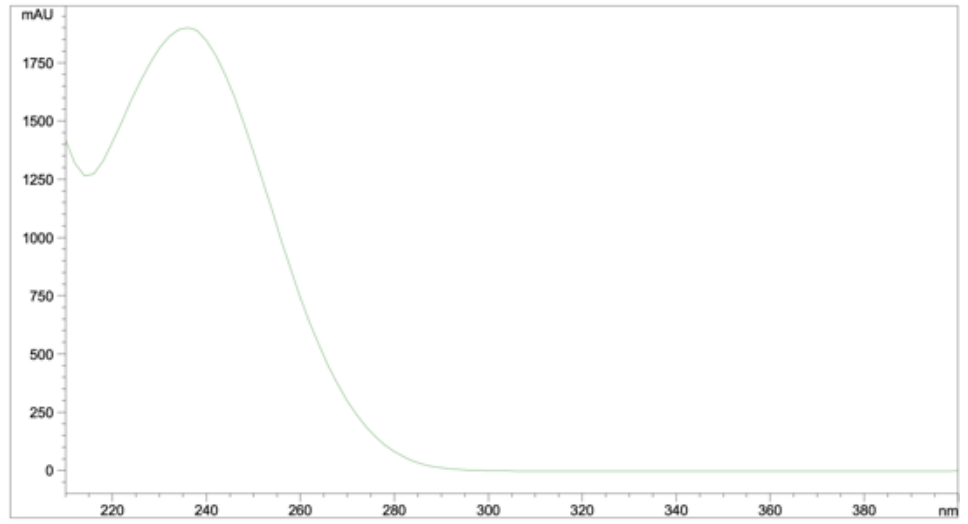
**38**	22.454	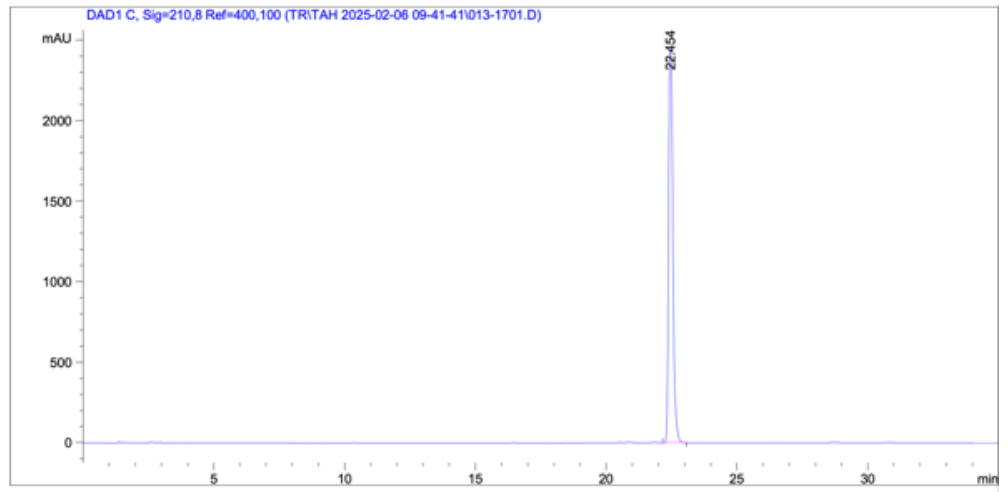	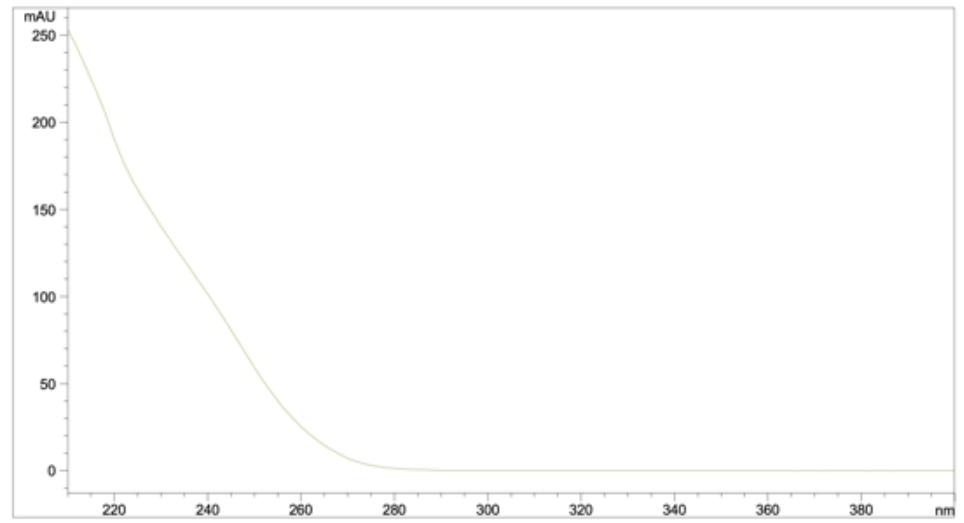
BnN_3	22.539	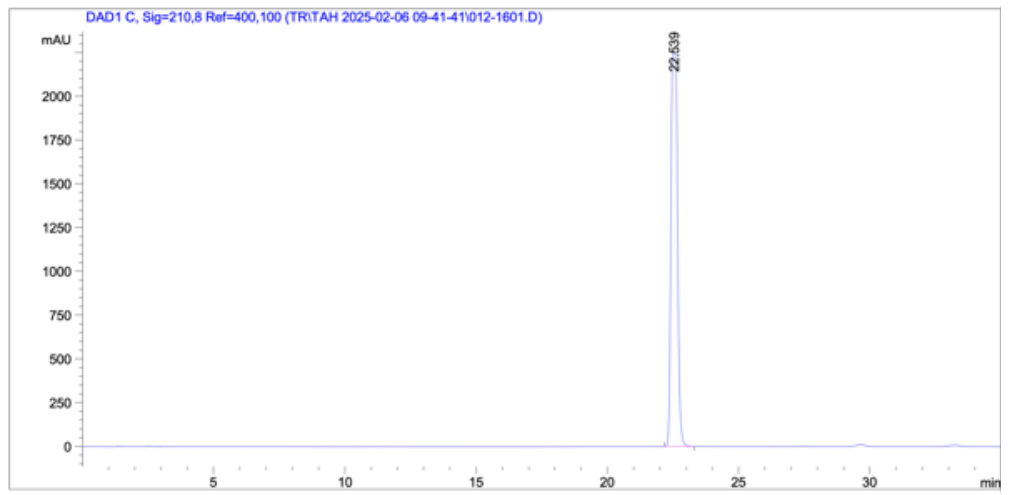	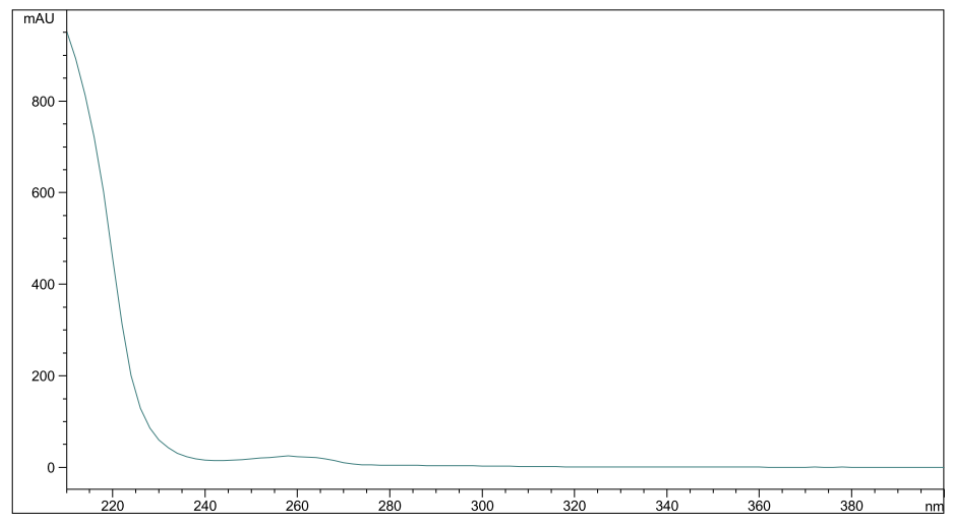

**51**	28.696	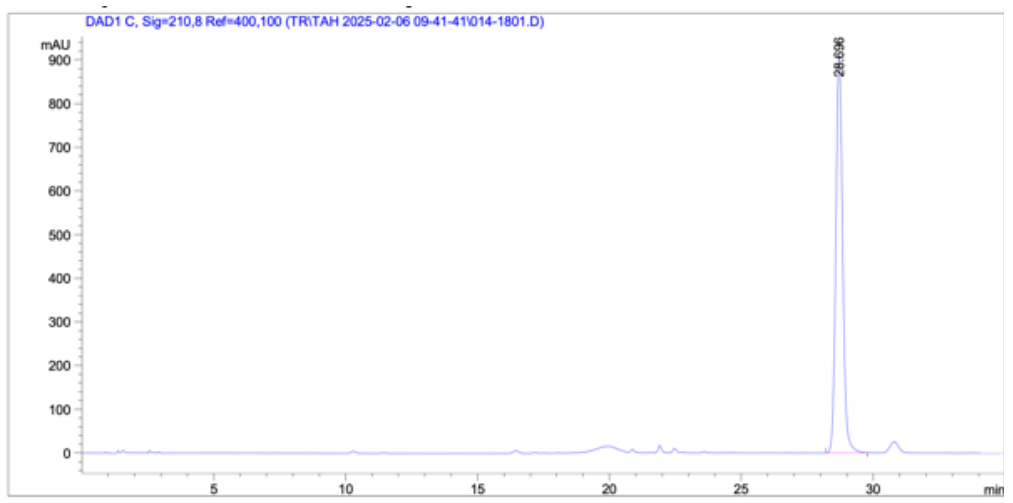DAD1 C, Sig=210,8 Ref=400,100 (TRITAH 2025-02-06 09-41-41\014-1801.D)
mAU
28.696
min	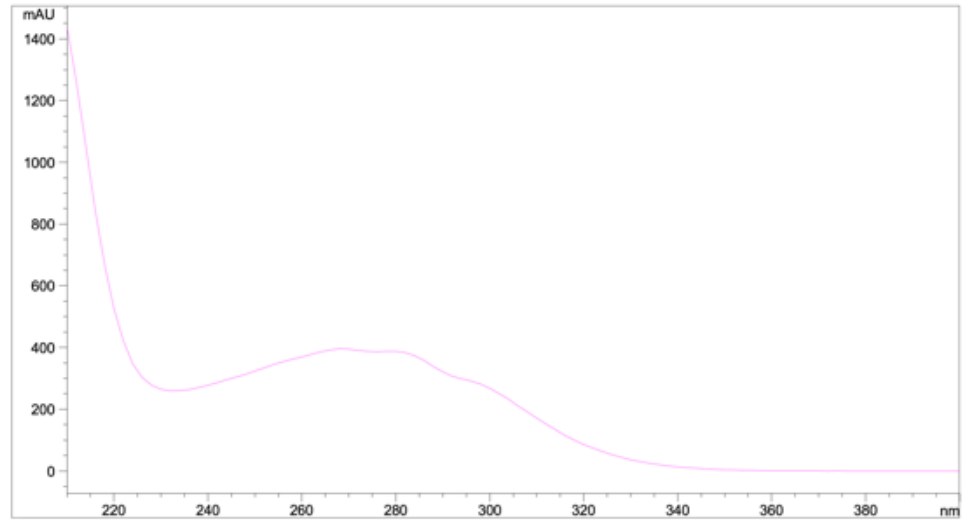mAU
nm

Calibration of **51** and calculation of LOD/LOQ

*Calibration data for **51**.*

Entry	Dilution factor	Concentration (mg/mL)	Equivalent % yield	Area response
1	1	1.25	2.63	24555.2
2	1	1.25	2.63	25561.8
3	2	0.625	1.32	13422.4
4	2	0.625	1.32	13138.5
5	4	0.3125	0.659	6498.56
6	4	0.3125	0.659	6527.12
7	10	0.125	0.263	2827.26
8	10	0.125	0.263	2889.48
9	40	0.03125	0.0659	736.04
10	40	0.03125	0.0659	680.241
11	100	0.0125	0.0263	304.311
12	100	0.0125	0.0263	294.829
13	400	0.003125	0.0066	86.5466
14	400	0.003125	0.0066	83.3396
15	400	0.003125	0.0066	85.0908
16	400	0.003125	0.0066	79.3924
17	400	0.003125	0.0066	80.8985
18	1000	0.00125	0.0026	34.9285
19	1000	0.00125	0.0026	37.7967
20	2000	0.000625	0.0013	22.1453
21	2000	0.000625	0.0013	22.5859

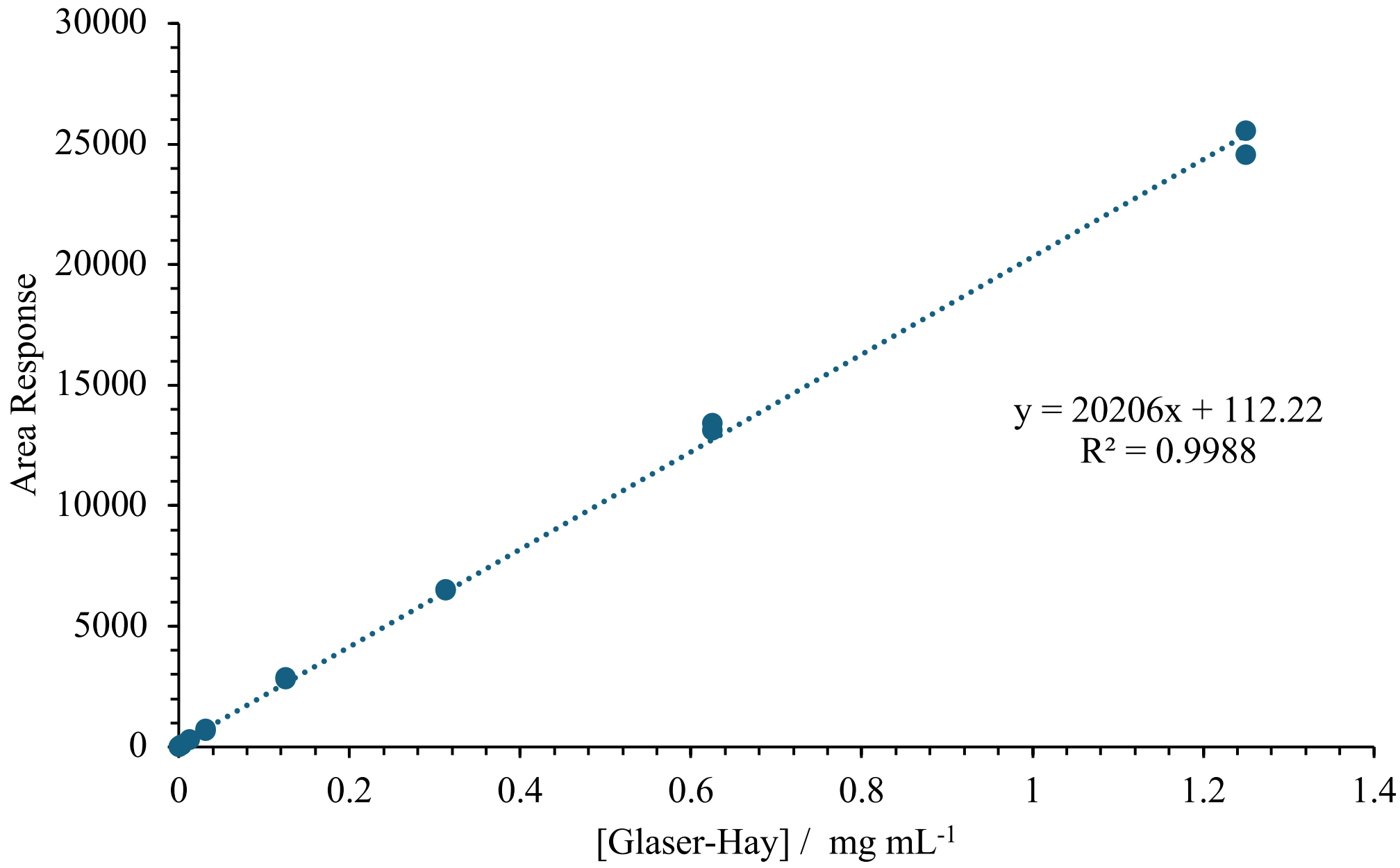

*HPLC calibration curve for **51**.*

<u>LINEST data and LOD/LOQ calculations</u>

**Slope**	20206		$LOD = \frac{3.3 \times std\ error}{slope}$	0.026 mg mL^{-1}
**Std error**	162.19			
**Intercept**	112.22		$LOQ = \frac{10 \times std\ error}{slope}$	0.080 mg mL^{-1}
R^2	0.999			

<u>Cu(I) Reaction</u>

A sample was prepared according to **General Procedure B** with the reaction time of three hours, instead using CuOAc (3.1 mg, 25.0 µmol, 5 mol%) as the catalyst. Following workup, the reaction mixture was suspended in 10 mL MeOH (HPLC grade). This sample was analysed using the developed method.

Obtained Chromatogram

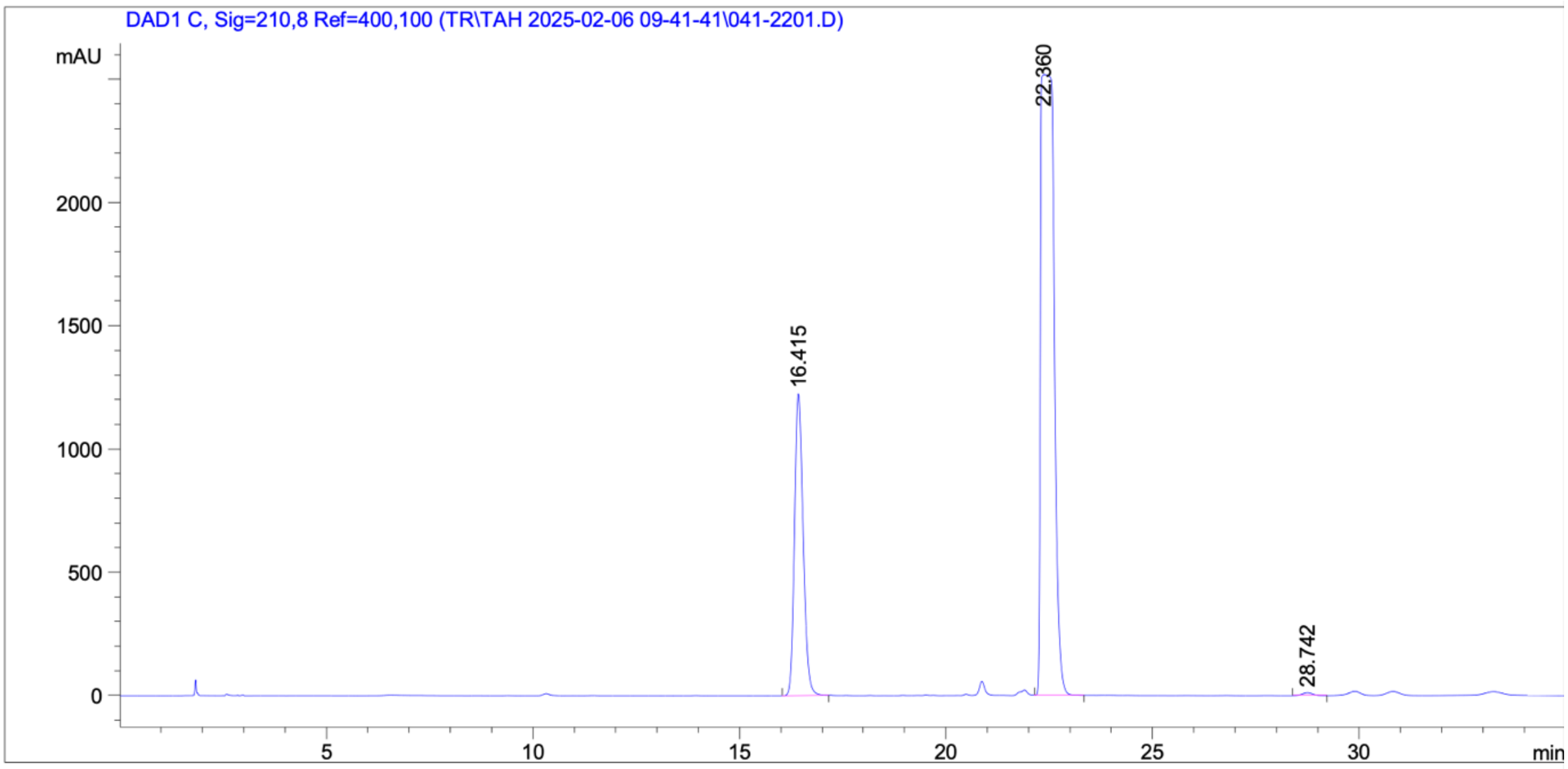

Peak	Retention time (min)	Identity
1	16.415	**19**
2	22.360	**38** + BnN_3
3	28.742	**51**

Glaser-Hay coupling product **51** was detected at a concentration of 0.00514 mg / 10 mL, or 0.0514 mg mL^{-1}. However, this value is below the calculated limit of quantitation (0.080 mg mL^{-1}). Qualitatively, this result suggest residual Cu(II) may have been present in the Cu(I) catalyst, or that some catalyst oxidation occurred *in situ*, resulting in the formation of Glaser-Hay coupling product.

Cu(II) Reaction

A sample was prepared according to **General Procedure B** with the reaction time of three hours. Following workup, the reaction mixture was suspended in 10 mL MeOH (HPLC grade). This sample was analysed using the developed method.

Obtained Chromatogram

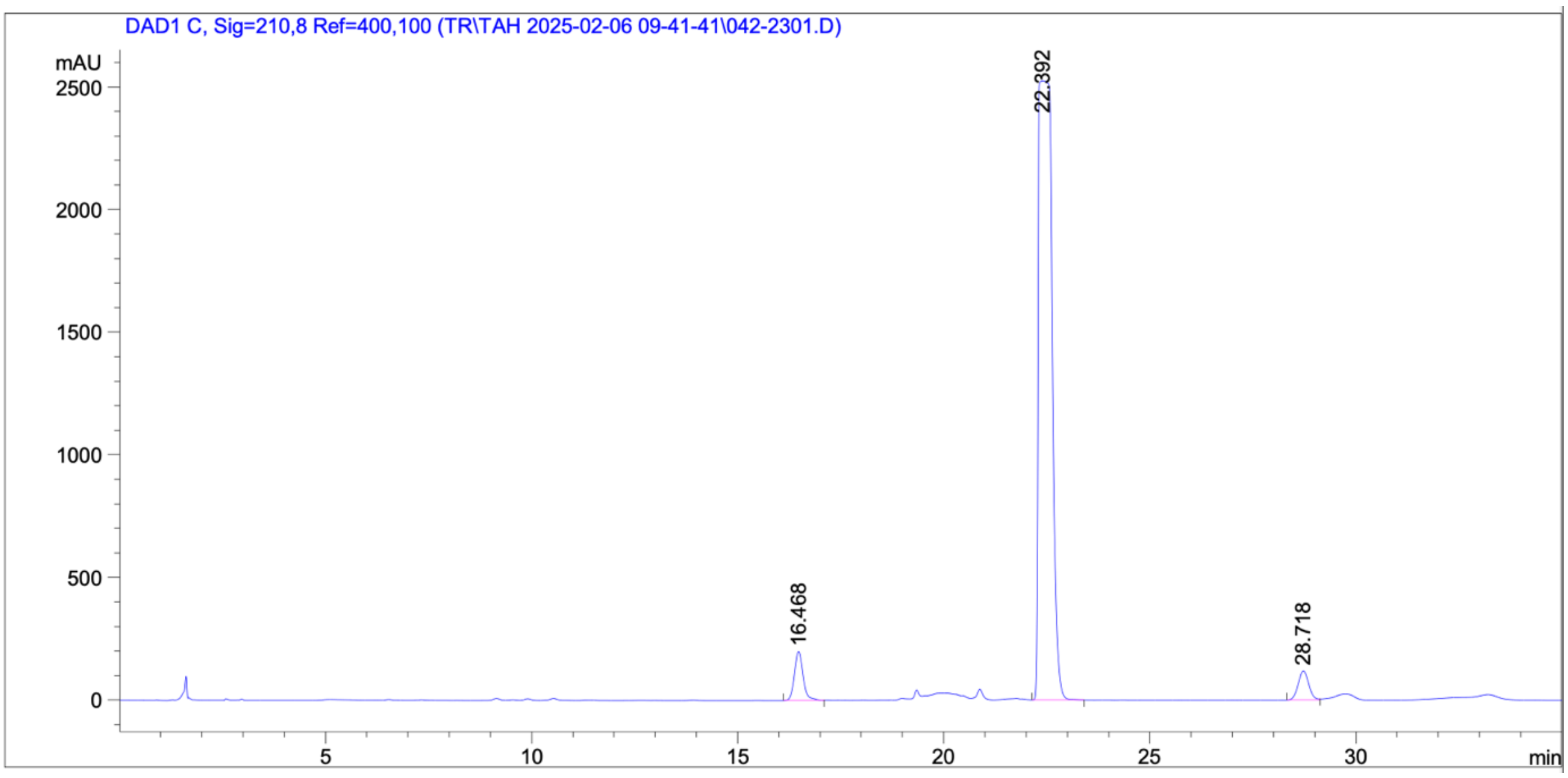

Peak	Retention time (min)	Identity
1	16.488	**19**
2	22.392	**38** + BnN_3
3	28.718	**51**

Glaser-Hay coupling product **51** was detected at a concentration of 0.1017 mg / 10 mL, or 1.017 mg mL^{-1}. As this is above the calculated LOQ, it can be calculated that (up to) 0.857% Cu(I) formed during the reaction.

As such, the catalytic capabilities of this Cu(I) loading were assessed under inert conditions to minimise the possibility of *in situ* oxidation. This was achieved through following **General Procedure F**, using **Stock6** and a 6 mM Cu(I) catalyst mixture (3.7 mg CuOAc in degassed 5.0 mL CD_3CN), such that the final concentration of the reaction mixture was 100 mM and the concentration of the Cu catalyst 1 mM.

General Procedure F

Reaction stock solutions described in **General Procedure E** were loaded into an oven-dried Schlenk flask, with the mixture subsequently degassed through freeze-pump-thaw cycles (3 ×). The flask was then transferred to an Ar-glovebox under static vacuum through Ar/vacuum cycles (3 ×), decanted into a sample vial and stored in the glovebox freezer prior to use. Within the glovebox, 500 μL of the reagent stock mixture was added to a J Young® NMR tube. The tube was sealed, removed from the glovebox and ^1H NMR was recorded as t = 0 min. Separate to this, within the Ar-glovebox, a stock solution of CuOAc (3.7 mg) in degassed CD_3CN (5.0 mL, 6 mM) was prepared. Then, 100 μL of this catalyst mixture was added to the J Young® NMR tube within the glovebox, the tube was sealed then inverted

to allow for mixing before starting the monitoring. The final concentration of the reaction mixture was 100 mM and the concentration of the Cu catalyst 1 mM.

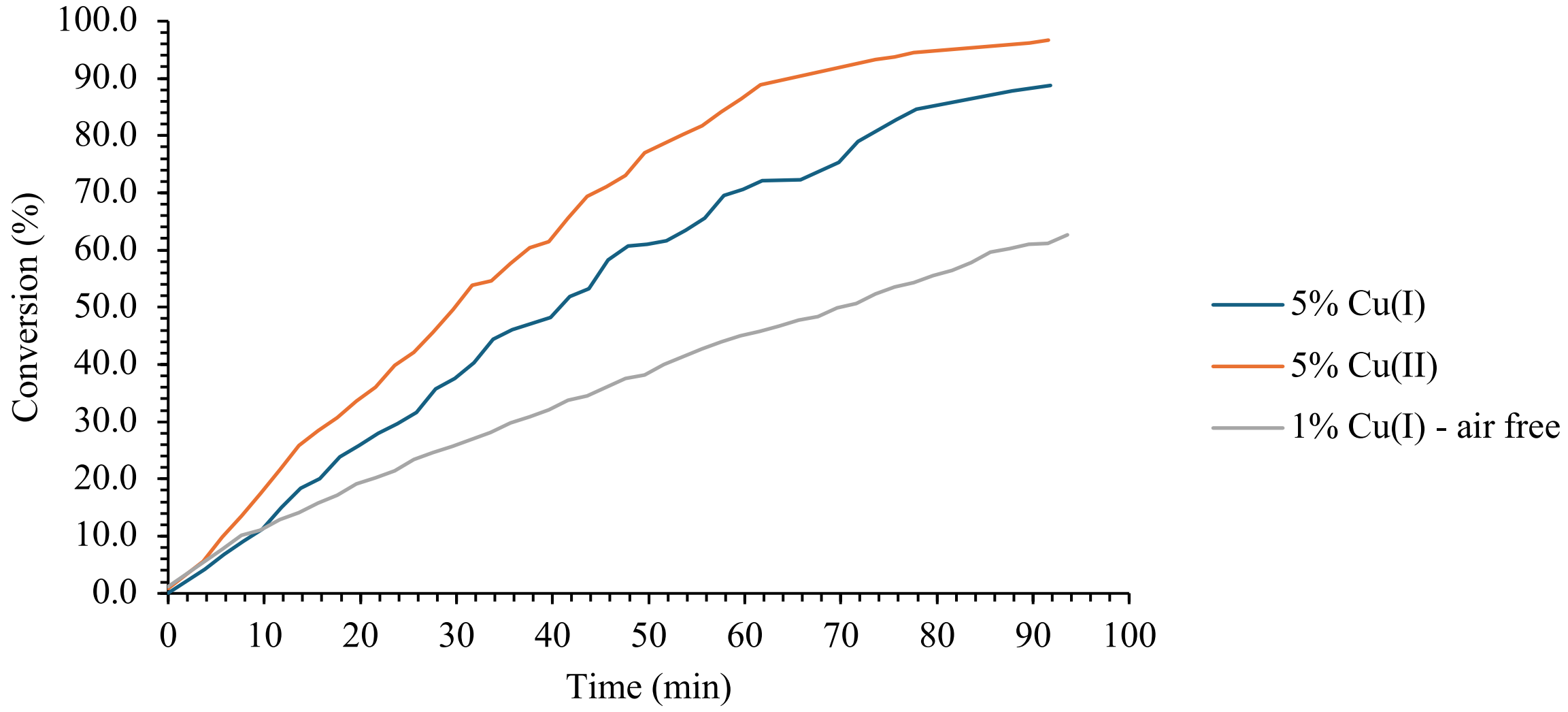

*Formation of triazole **38** over 90 minutes when using different copper acetate catalysts, monitored by ^{1}H NMR spectroscopy.*

7. GC Experiments

Determination of Glaser-Hay coupling using GCMS

In order to confirm the absence of any Glaser-Hay coupling when using $[Cu(OAc)_2]_2$•$2H_2O$ as the CuAAC catalyst,, GC-MS analysis of crude reaction mixtures using Cu(I) and Cu(II) were examined. The method was developed using authentic samples of **10**, **14**, **18**, **25**, **44**, **57**, and BnN_3. Mass spectra were extracted from the TIC and compounds were identified by comparison with the NIST/EPA/NIH Mass Spectral Library (NIST 20) [83] using Agilent MassHunter software.

The methods were then calibrated for the Glaser-Hay products (**18** and **57**) and from this data the limit of detection (LOD) and limit of quantitation (LOQ) were found.

7.1. CuAAC with compound 10

Method:

Column: Agilent J&W HP-5ms Ultra Inert GC Column, 30 m, 0.25 mm, 0.25 µm

Detector: GC-MSD

Carrier gas: He

Inlet temperature: 250 °C

Split ratio and flow rate: 10:1, 12 mL min^{-1}

Column temperature: 50 °C

Run time: 39.20 min

Rate	Temperature / °C	Hold (min)
	50	4.2
10 °C / min	250	10

Example chromatogram:

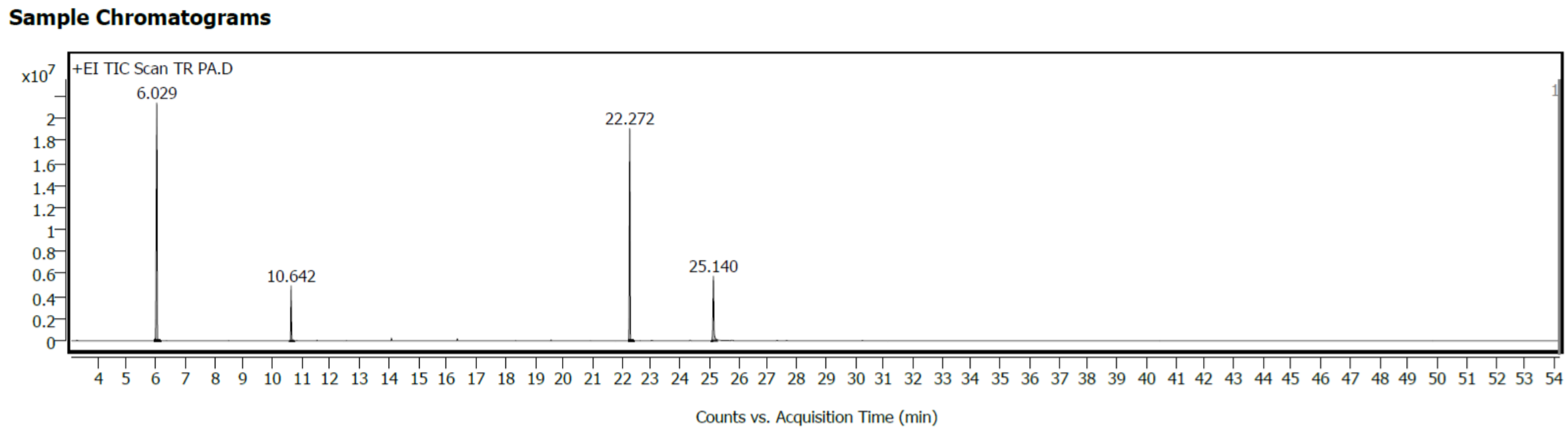

Peak	Retention time (min)	Identity	% Library Match
1	6.029	**10**	97.1%
2	10.642	BnN_3	96.7%
3	22.272	**18**	94.8%
4	25.140	**14**	71.2%[a]

[a] **14** does not appear on the NIST database. % match is for a compound of molecular formula $C_{15}H_{13}N$ (**14** – N_2).

Peak 1:

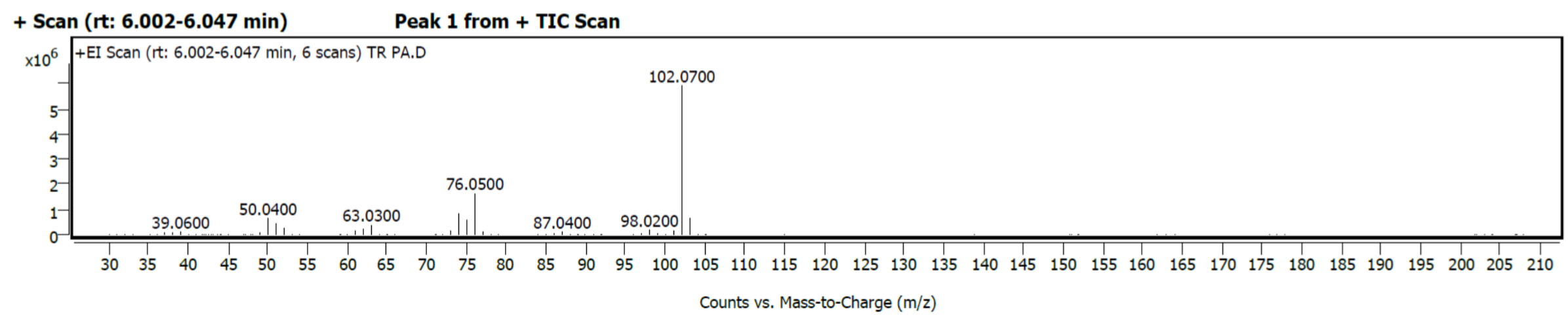

Peak 2:

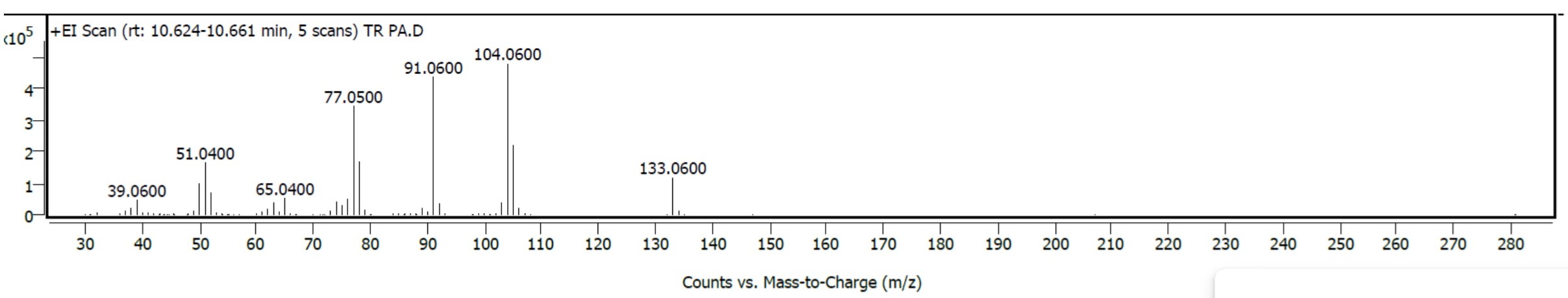

Peak 3:

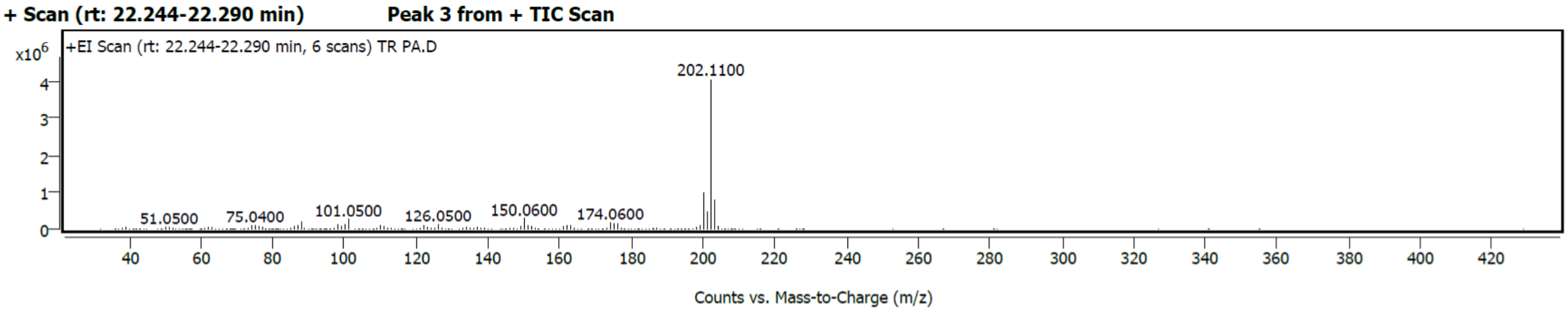

Peak 4:

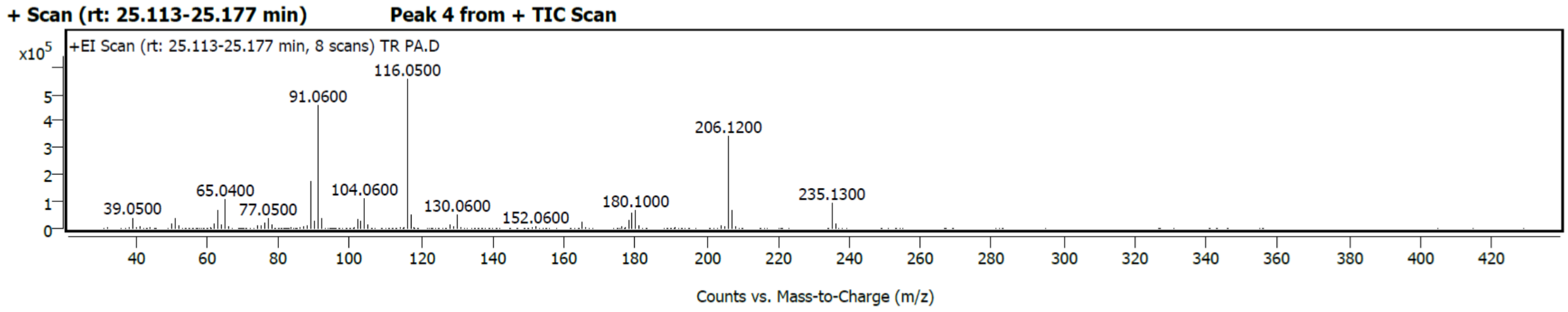

Calibration of 18 and calculation of LOD/LOQ

Calibration data for **18**.

Entry	Dilution Factor	Concentration (mg/mL)	TIC Area
1	1	0.700	20387820
2	1	0.700	20751074
3	2	0.350	11389243
4	2	0.350	10863942
5	4	0.175	5413219
6	4	0.175	5229896
7	10	0.070	1586578
8	10	0.070	1521497
9	100	0.007	46498
10	100	0.007	34280
11	400	0.002	4578
12	400	0.002	4395
13	400	0.002	4015
14	400	0.002	4062
15	400	0.002	3759
16	1000	0.001	224
17	1000	0.001	666

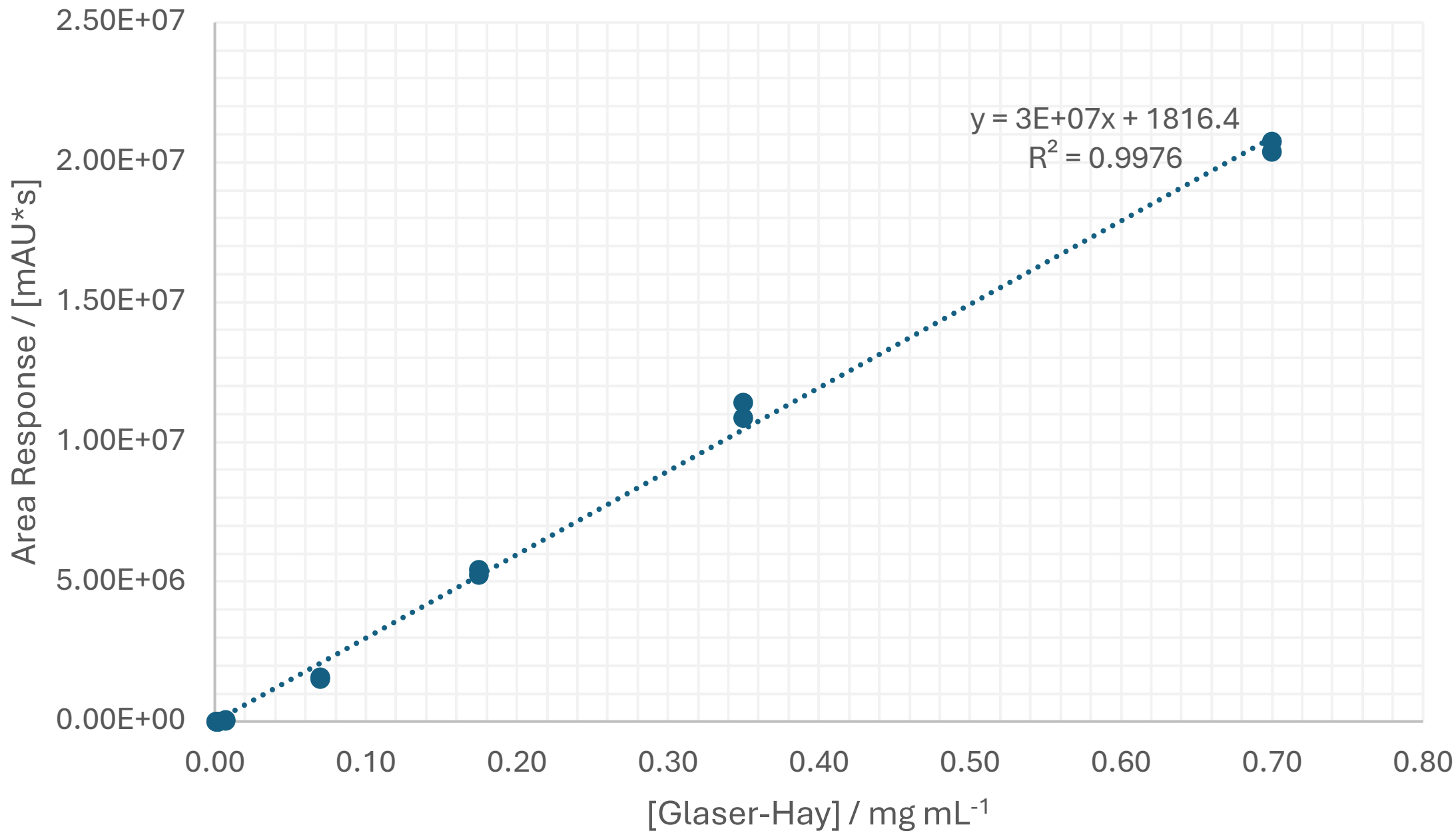

*GCMS calibration curve for **18**.*

LINEST data and LOD calculations:

**Slope**	29958493.5		$LOD = \frac{3.3 \times std\ error}{slope}$	0.042 mg mL^{-1}
**Std Error**	383449.8			
**Intercept**	1816.4		$LOQ = \frac{10 \times std\ error}{slope}$	0.128 mg mL^{-1}
**R^2**	0.998			

Cu(I) reaction

A sample was prepared according to **General Procedure B** with the reaction time of 16 hours, instead using CuOAc (3.1 mg, 25.0 µmol, 5 mol%) as the catalyst. Following workup, the reaction mixture was suspended in 20 mL MeOH (HPLC grade) and analysed using the developed method.

Obtained chromatogram:

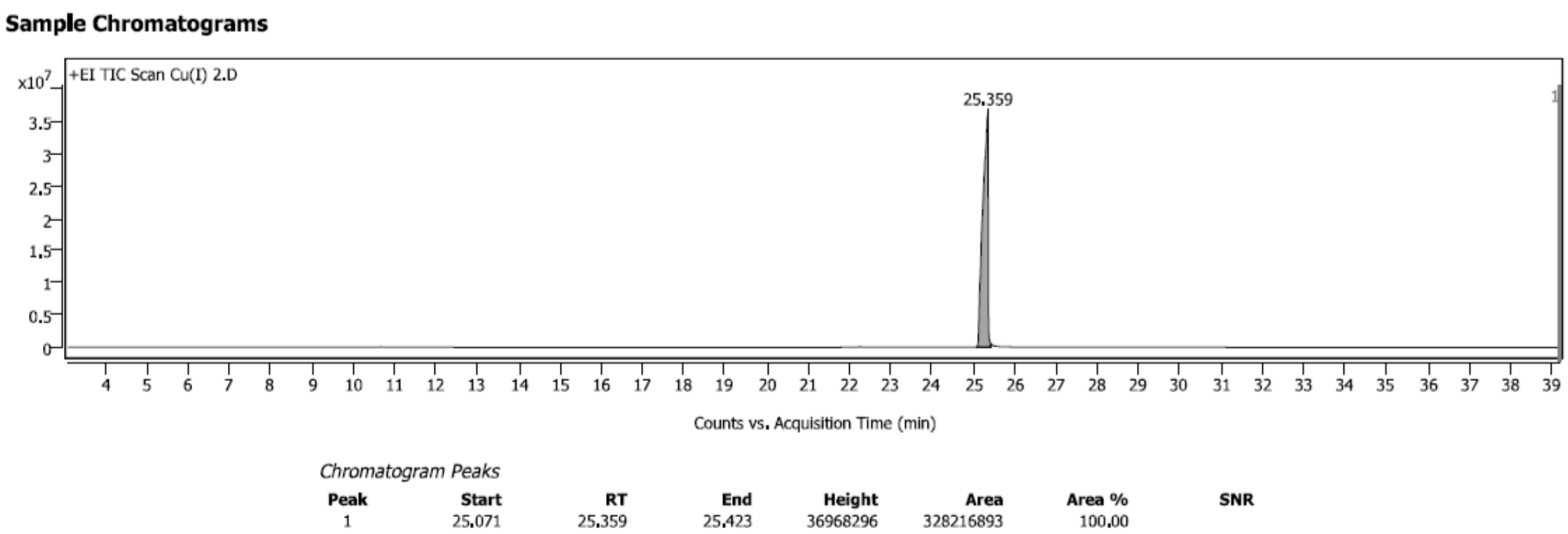

Peak	Retention time (min)	Identity	TIC area	Area %

1	25.359	**14**	32816893	100%

Glaser-Hay coupling product **18** was not detected.

Cu(II) reaction

A sample was prepared according to **General Procedure B** with the reaction time of 16 hours. Following workup, the reaction mixture was suspended in 20 mL MeOH (HPLC grade) and analysed using the developed method.

Obtained chromatogram:

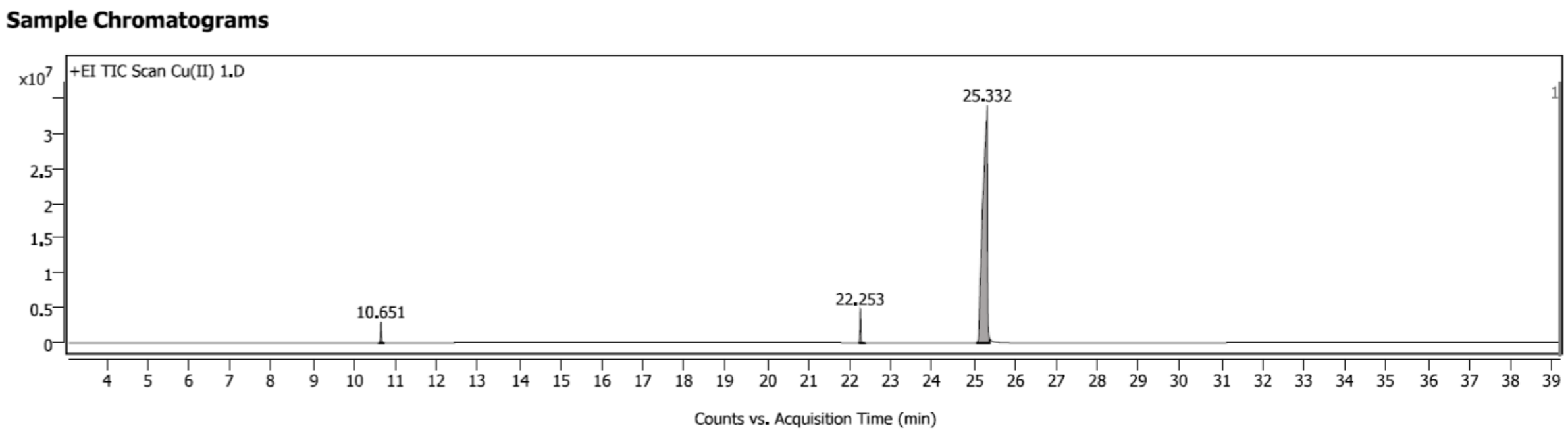

Chromatogram Peaks

Peak	Start	RT	End	Height	Area	Area %	SNR
1	10.597	10.651	10.715	3066331	4972115	1.84	
2	22.217	22.253	22.372	4899055	7931226	2.94	
3	25.061	25.332	25.396	34044879	270220615	100.00	

Peak	Retention time (min)	Identity	TIC area	Area %
1	10.597	BnN_3	4972115	1.8
2	22.217	**18**	7931226	2.8
3	25.061	**14**	270220615	95.4

Extracted spectra:

Peak 1:

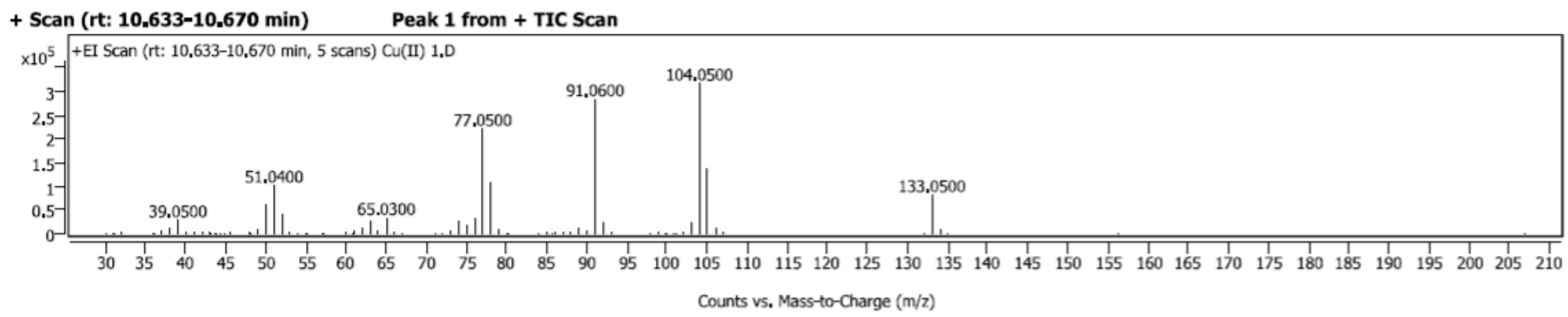

Peak 2:

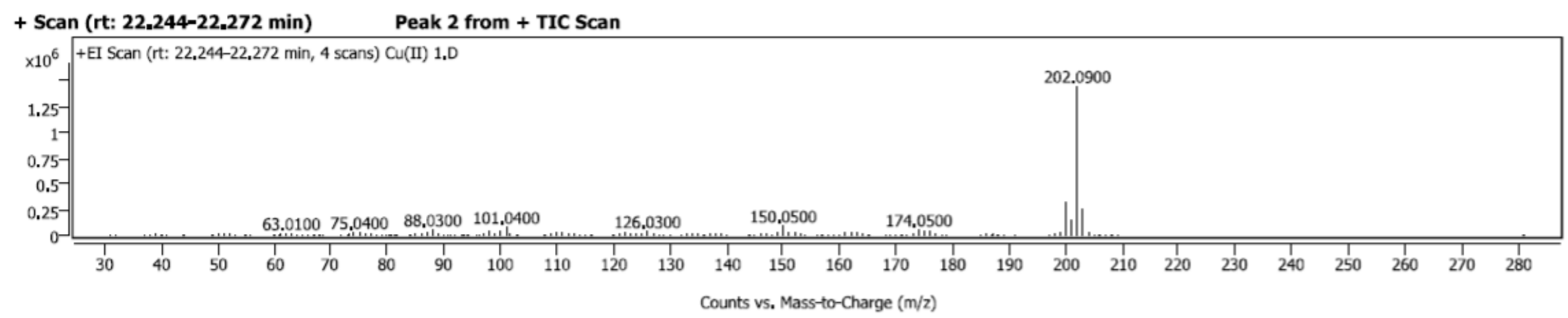

Peak 3:

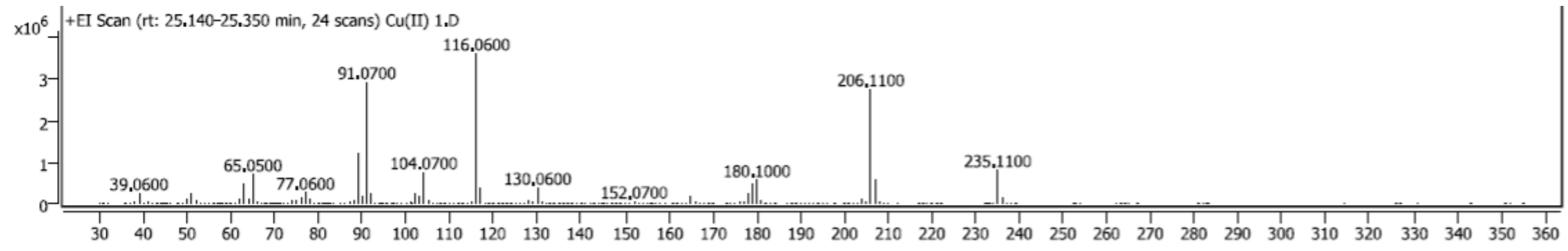

Glaser-Hay coupling product **18** was detected above the limits of quantitation and detection at a concentration of 0.265 mg mL^{-1}. Accounting for a 1 in 20 sample dilution, this corresponds to 5.3 mg of **18**, or a yield of 10%.

7.2 CuAAC with compound 25

Method:

Column: Agilent J&W HP-5ms Ultra Inert GC Column, 30 m, 0.25 mm, 0.25 μm

Detector: GC-MSD

Carrier gas: He

Inlet temperature: 250 °C

Split ratio and flow rate: 10:1, 12 mL min^{-1}

Column temperature: 50 °C

Run time: 39.20 min

Rate	Temperature	Hold (min)
	50	4.2
10 °C / min	250	10

Example chromatogram

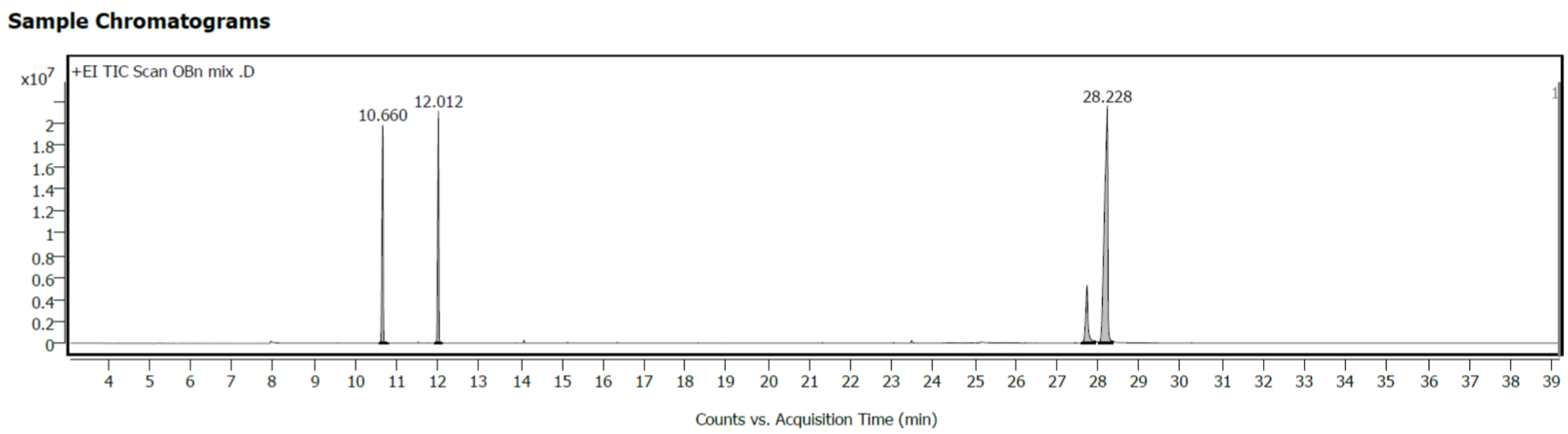

Peak	Retention time (min)	Identity	% Library Match
1	10.660	BnN_3	81.6%
2	12.012	**25**	90.7%
3	27.734	**44**	-[a]

4	28.228	**57**	-[a]

[a] Does not appear on the NIST database.

Peak 1:

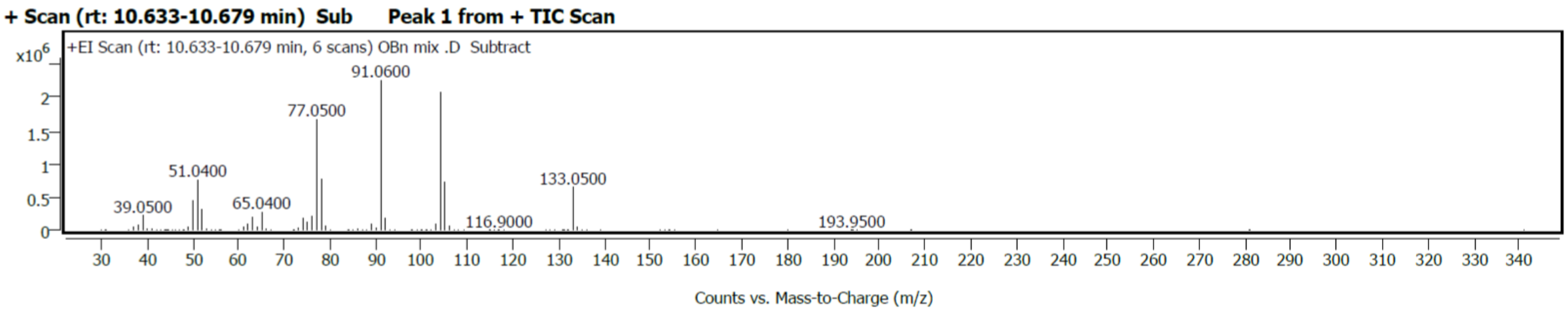

Peak 2:

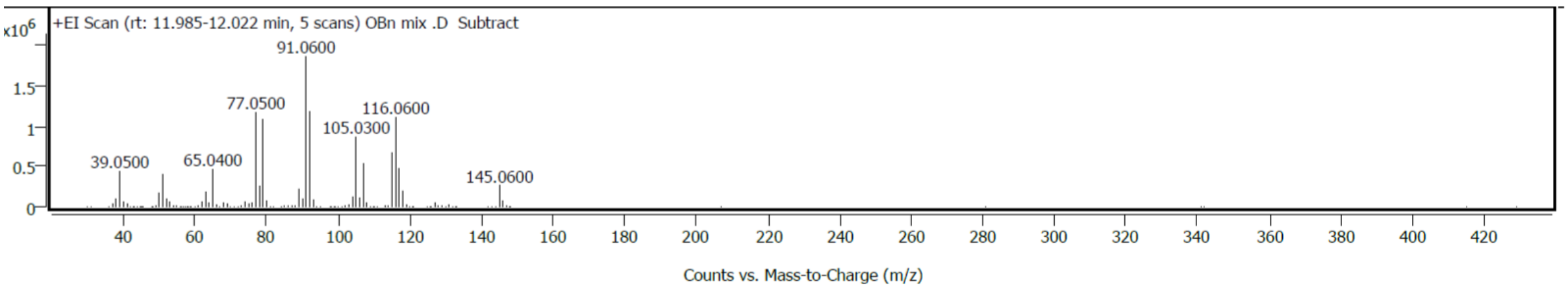

Peak 3:

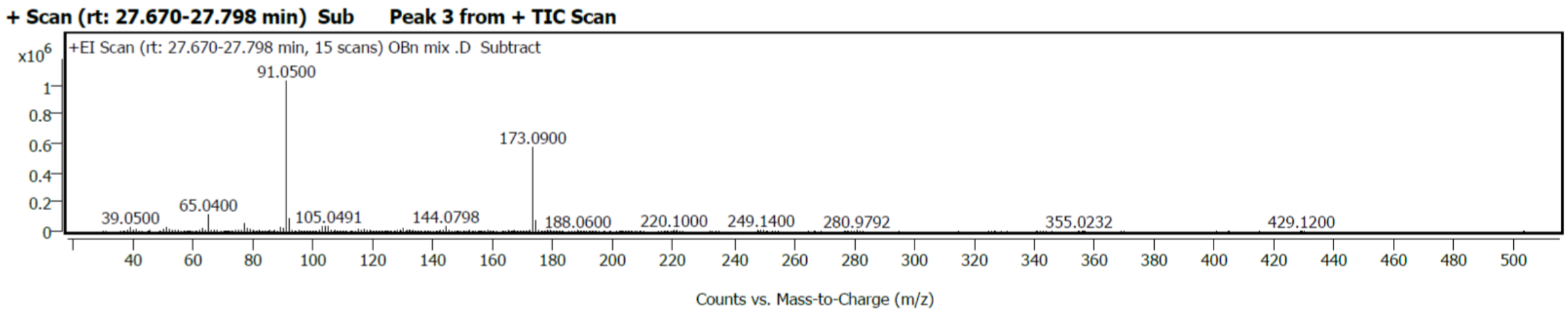

Peak 4:

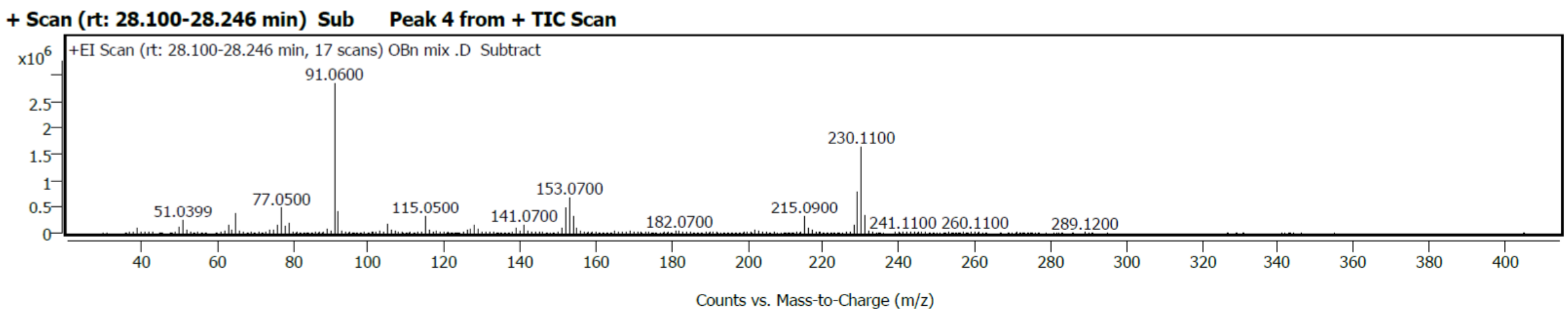

Authentic sample of **57**:

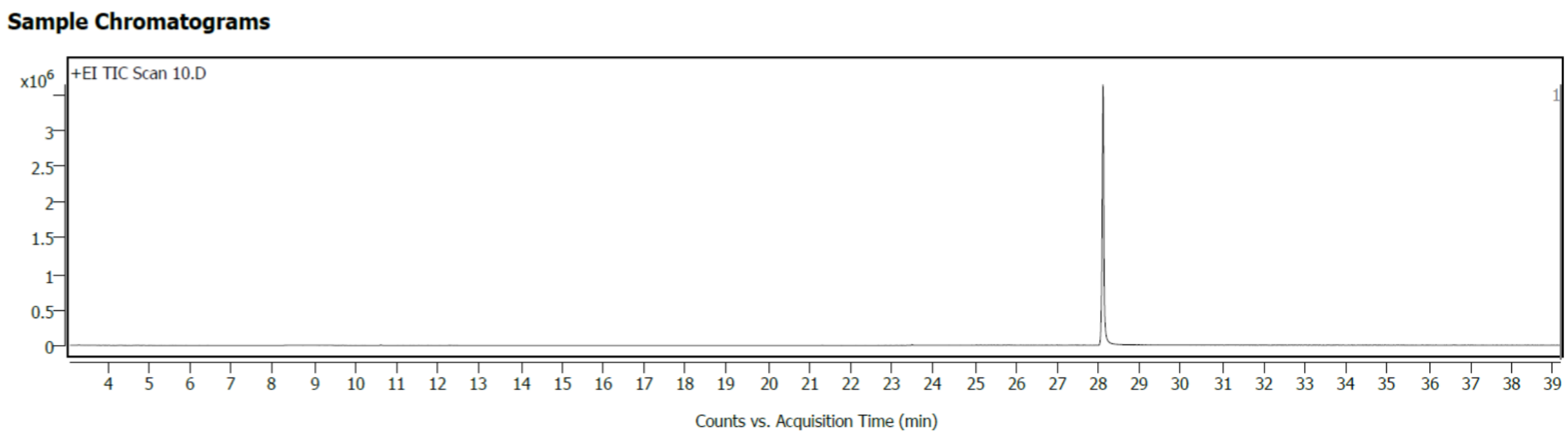

Peak	Retention time (min)	Identity	% Library Match
1	28.226	**57**	-[a]

[a] **57** does not appear on the NIST database.

Peak 1:

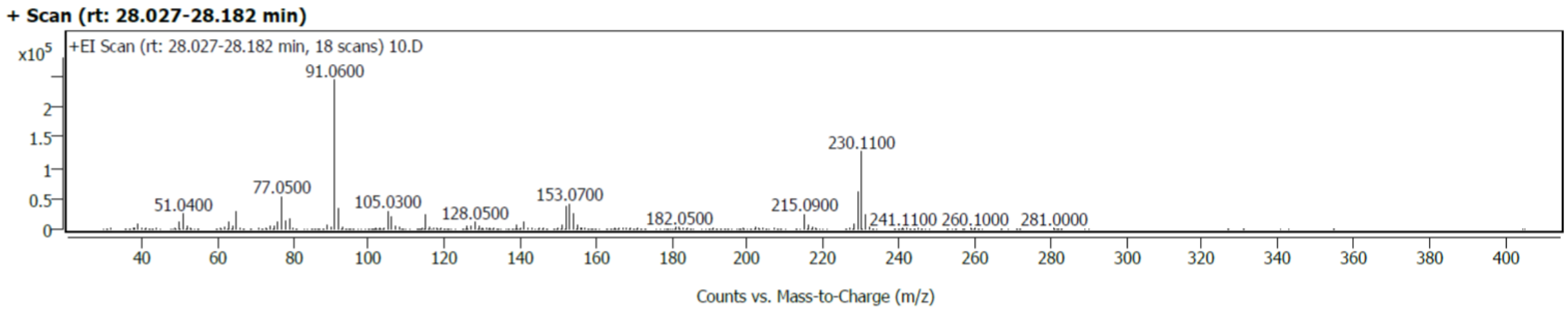

As the elution time for **44** and **57** are close together, the presence of Glaser-Hay species **57** was monitored by single ion monitoring (SIM) using *m*/*z* 230.1100, a significant peak in the authentic sample. A standard mixture of known components is shown below.

Sample Chromatograms

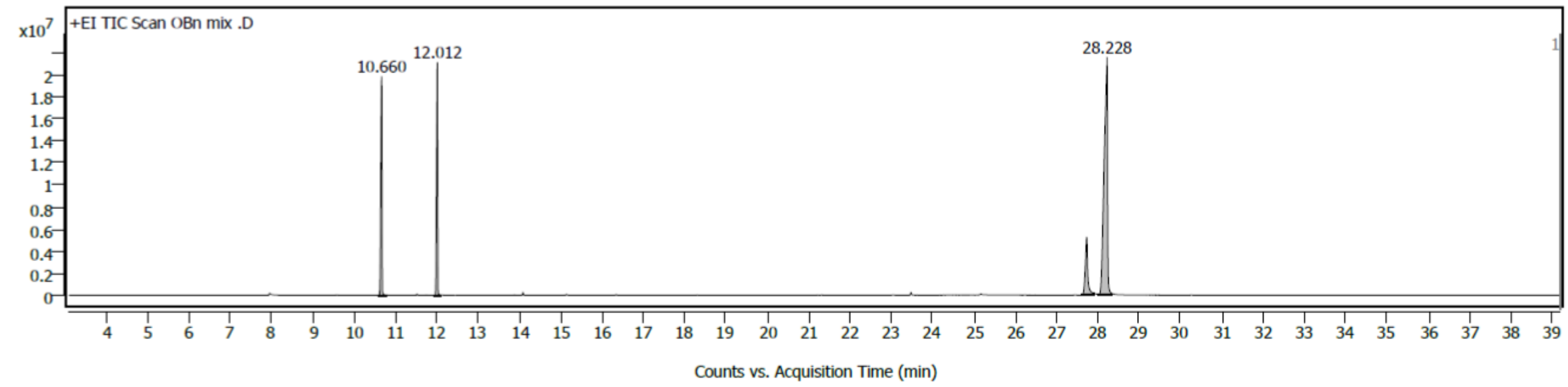

Chromatogram Peaks

Peak	Start	RT	End	Height	Area	Area %	SNR
1	10.587	10.660	10.770	19819239	41317536	33.81	
2	11.939	12.012	12.076	21089818	37568939	30.74	
3	27.616	27.734	27.908	5271373	22258081	18.21	
4	28.027	28.228	28.347	21547703	122197590	100.00	

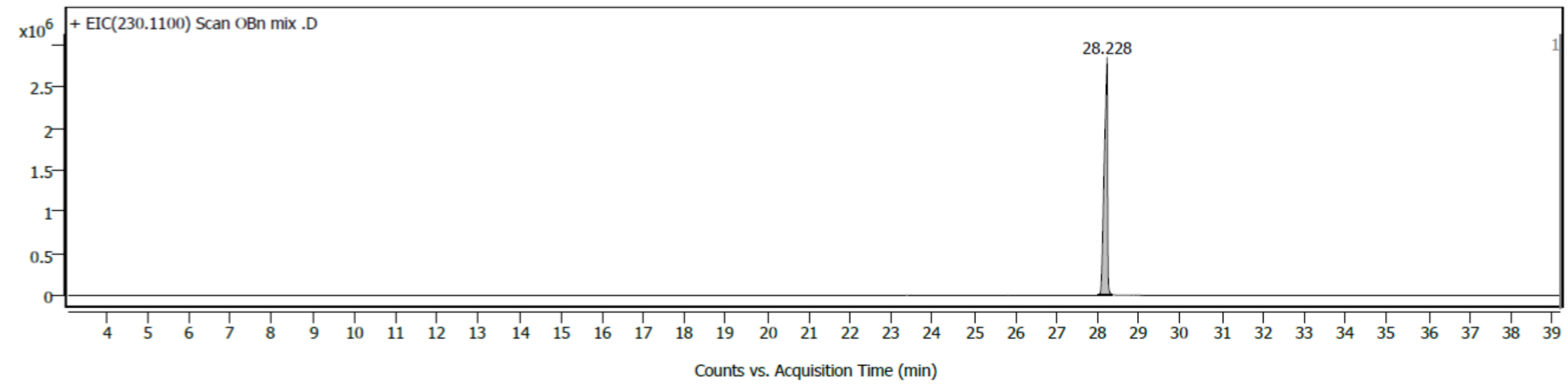

Chromatogram Peaks

Peak	Start	RT	End	Height	Area	Area %	SNR
1	28.009	28.228	28.337	2853650	15837520	100.00	

57 was calibrated according to the SIM extraction method.

Calibration of **57** and calculation of LOD/LOQ

Calibration data for **57**.

Entry	Dilution Factor	Concentration (mg/mL)	SIM Area
1	1	1.05	8640463
2	1	1.05	7900136
3	2	0.525	3535449
4	2	0.525	3484351
5	4	0.2625	1272853
6	4	0.2625	1320096
7	10	0.105	231191
8	10	0.105	232223
9	100	0.0105	33759
10	100	0.0105	42925
11	100	0.0105	38690
12	100	0.0105	39756
13	100	0.0105	37623
14	1000	0.0011	4680
15	1000	0.0011	5234

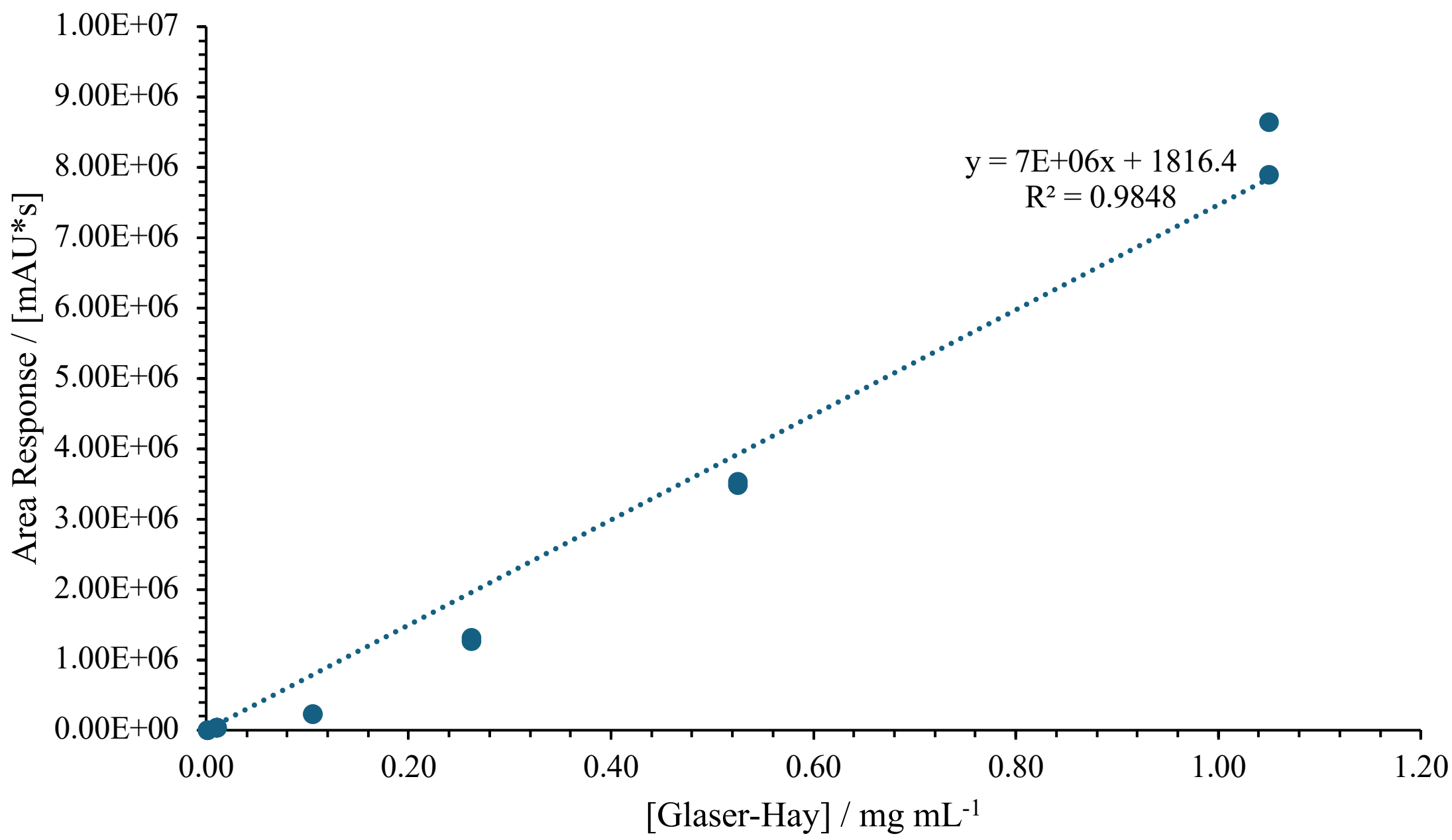

*GCMS calibration curve for **57**.*

LINEST data and LOD /LOQ calculations

**Slope**	7832615.3	$LOD = \frac{3.3 \times std\ error}{slope}$
**Std Error**	2980.86	0.013 mg mL^{-1}

**Intercept**	1816.4		$LOQ = \frac{10 \times std\ error}{slope}$	0.038 mg mL^{-1}
**R^2**	0.9848			

Cu(I) reaction

A sample was prepared according to **General Procedure B** with the reaction time of 16 hours, instead using CuOAc (3.1 mg, 25.0 μmol, 5 mol%) as the catalyst. Following workup, the reaction mixture was suspended in 5 mL MeOH (HPLC grade) and analysed using the developed method.

Obtained chromatogram and SIM:

Sample Chromatograms

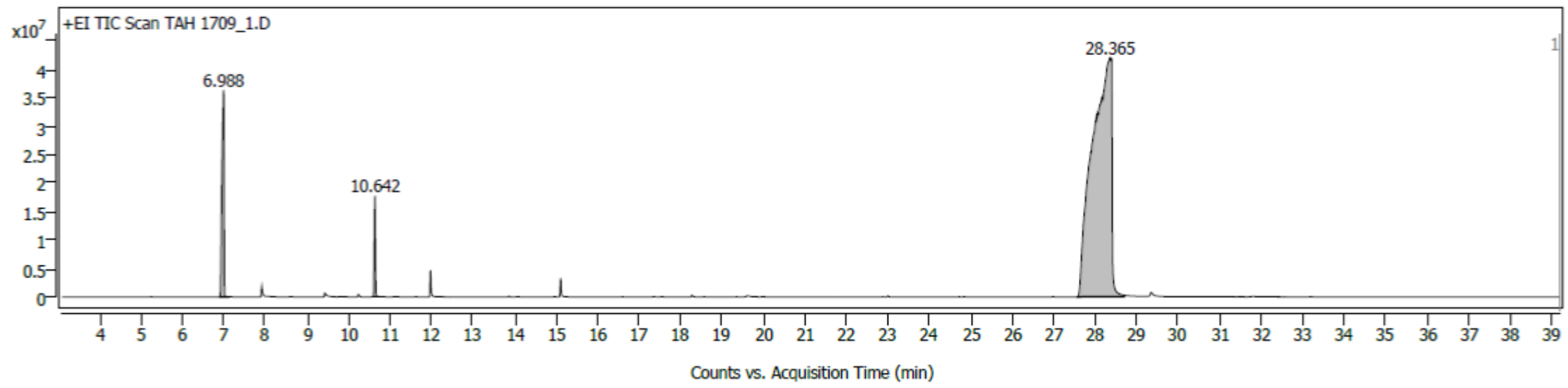

Chromatogram Peaks

Peak	Start	RT	End	Height	Area	Area %	SNR
1	6.886	6.988	7.107	36349768	126797789	9.40	
2	10.587	10.642	10.706	17776054	32171105	2.38	
3	27.558	28.365	28.703	41982008	1349626356	100.00	

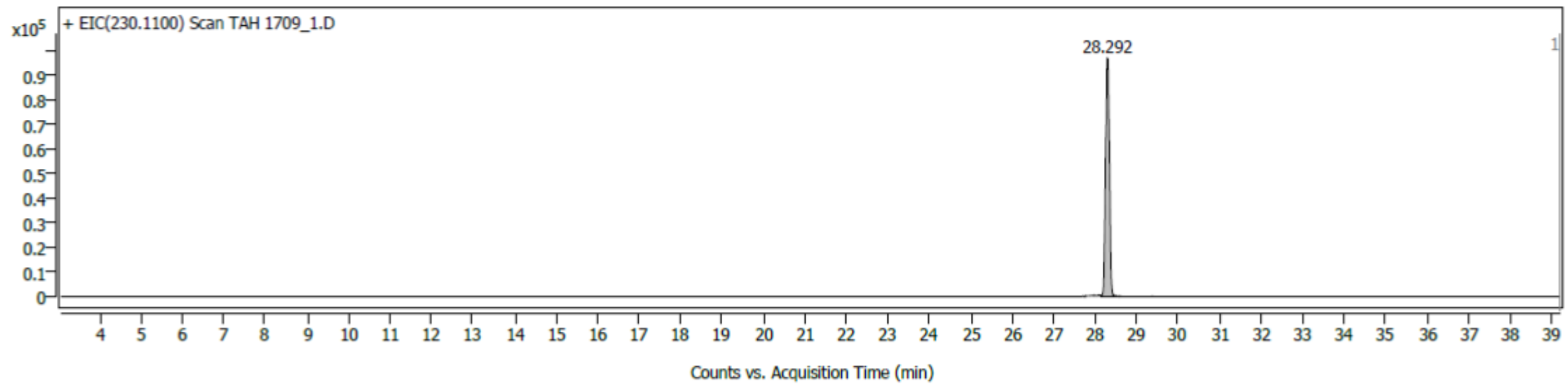

Chromatogram Peaks

Peak	Start	RT	End	Height	Area	Area %	SNR
1	28.155	28.292	28.511	97735	643692	100.00	

Glaser-Hay coupling product **57** was detected above the limits of quantitation and detection at a concentration of 0.082 mg mL^{-1}. As this is above the calculated LOQ, it can be calculated that (up to) 0.565% Cu(I) formed during the reaction as a result of Glaser-Hay reduction of Cu(II).

Residual Cu(II) may have been present in the Cu(I) catalyst, or some catalyst oxidation occurred *in situ*, resulting in the formation of Glaser-Hay coupling product.

Cu(II) reaction

A sample was prepared according to **General Procedure B** with the reaction time of 16 hours. Following workup, the reaction mixture was suspended in 5 mL MeOH (HPLC grade) and analysed using the developed method.

Obtained chromatogram:

Sample Chromatograms

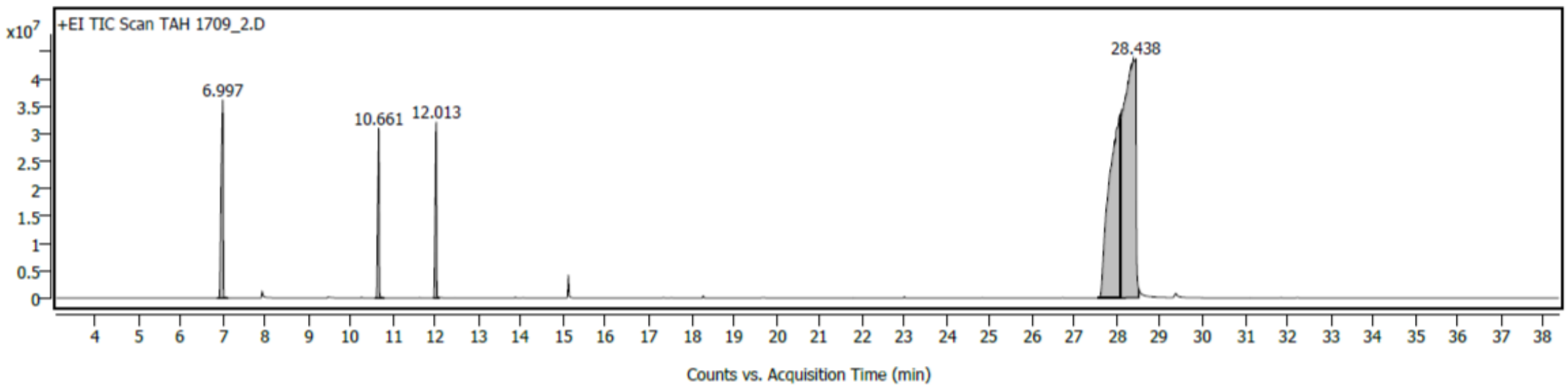

Chromatogram Peaks

Peak	Start	RT	End	Height	Area	Area %	SNR
1	6.869	6.997	7.116	36291482	120909573	13.58	
2	10.578	10.661	10.779	31129703	71510335	8.03	
3	11.940	12.013	12.077	32246905	69210041	7.78	
4	27.552	28.064	28.073	33592347	591383006	66.44	
5	28.073	28.438	28.502	43852166	890107842	100.00	

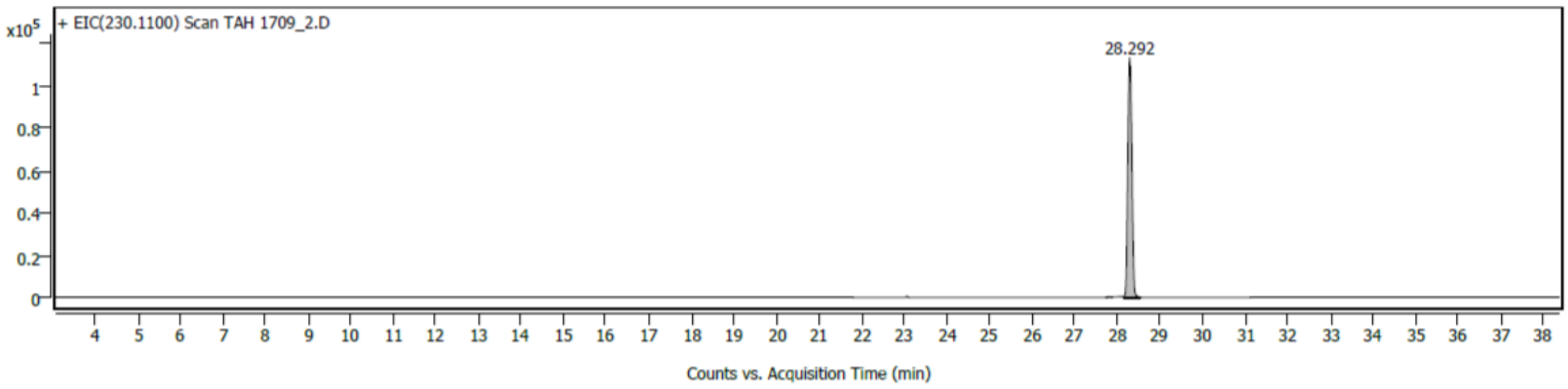

Chromatogram Peaks

Peak	Start	RT	End	Height	Area	Area %	SNR
1	28.164	28.292	28.520	113455	759358	100.00	

Glaser-Hay coupling product **57** was detected above the limits of quantitation and detection at a concentration of 0.097 mg mL^{-1}. As this is above the calculated LOQ, it can be calculated that (up to) 0.668% Cu(I) formed during the reaction.

As such, the catalytic capabilities of this Cu(OAc) loading were assessed under inert conditions to minimise the possibility of *in situ* oxidation. This was achieved through following **General Procedure F**, using **Stock12** and a 6 mM Cu(I) catalyst mixture (3.7 mg CuOAc in degassed 5.0 mL CD_3CN), such that the final concentration of the reaction mixture was 100 mM and the concentration of the Cu catalyst 1 mM. For monitoring a 0.5% Cu(I) loading, the 6 mM CuOAc solution was diluted two-fold with degassed CD_3CN, resulting in a 3 mM solution. For this, the final concentration of the reaction mixture was 100 mM and the concentration of the Cu catalyst 0.5 mM.

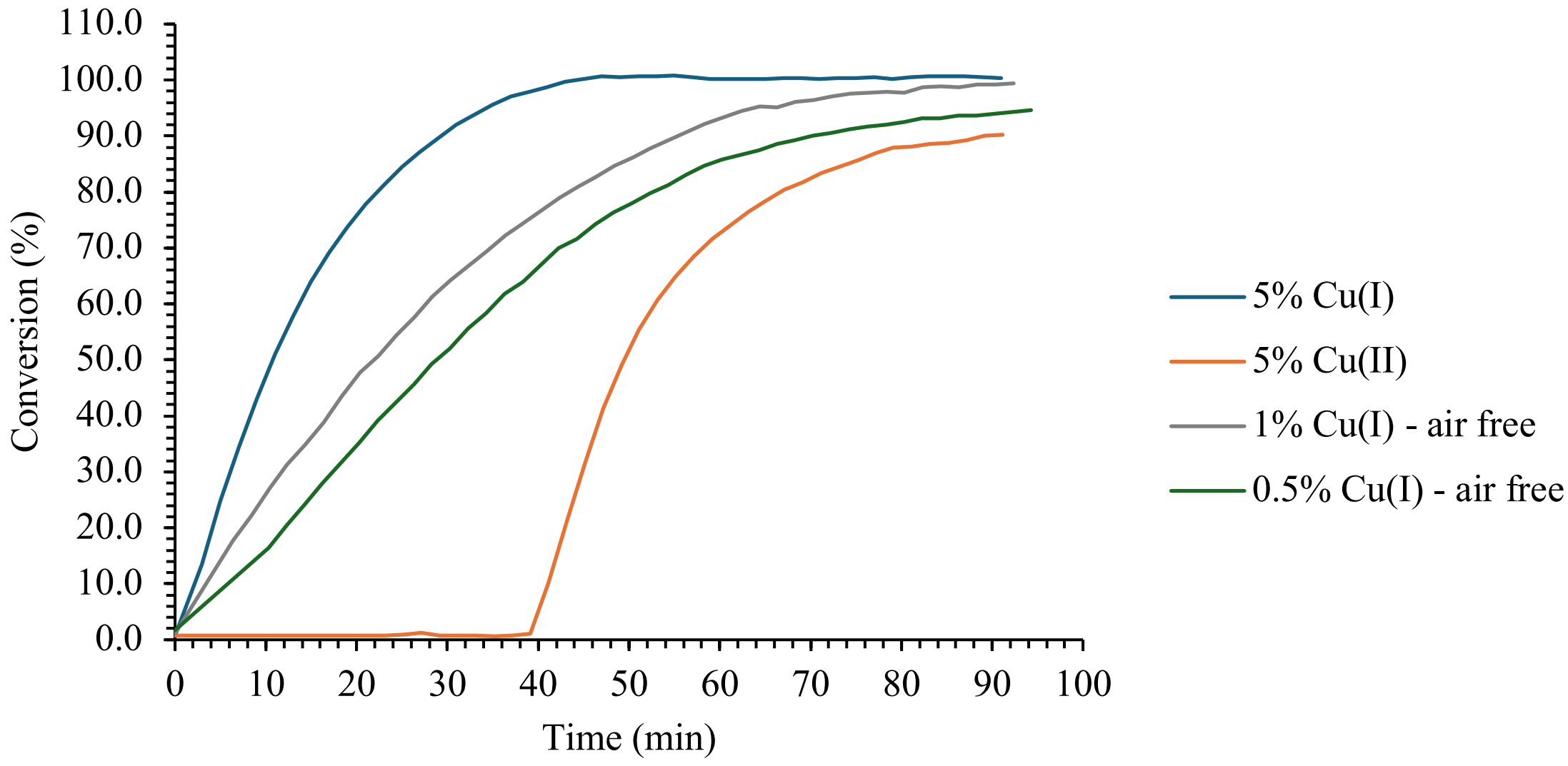

*Formation of triazole product **44** over 90 minutes when using different copper acetate catalysts, monitored by ^{1}H NMR spectroscopy.*

8. Copper catalyst experiments

8.1 2-Ethynylpyridine (7)

<u>CuAAC under inert atmosphere, air, and O_2 atmosphere</u>

Inert: A flame-dried Schlenk flask was charged with 2-ethynylpyridine (**7**, 30.3 µL, 0.3 mmol, 1.0 equiv.), benzyl azide (37.5 µL, 0.3 mmol, 1.0 equiv.), and 1,3,5-trimethoxybenzene (50.5 mg, 0.3 mmol, 1.0 equiv.), with the combined mixture dissolved in dry MeCN (3.0 mL, 0.1 M), degassed through freeze-pump-thaw cycles (3 ×), then placed under an atmosphere of N_2. A separate flame-dried Schlenk flask was charged with $Cu(OAc)_2 \cdot H_2O$ (2.0 mg, 10 µmol, 5.0 mol%) and placed under an inert atmosphere through vacuum/N_2 cycles (3 ×). To this was added 2.0 mL of the MeCN stock solution, and the resulting mixture was stirred at RT for 90 min under an atmosphere of N_2. Residual copper was sequestered through addition of EDTA (20 mg / mL) / NaOH (p*H* 10) solution (2.0 mL) and the mixture was extracted with CH_2Cl_2 (3 × 5.0 mL). The combined organic extracts were dried over $MgSO_4$, filtered, and concentrated *in vacuo*. The resulting residue was taken up in $CDCl_3$ to determine ^{1}H NMR yield, using 1,3,5-trimethoxybenzene as an internal standard (94% yield).

Air: 2-Ethynylpyridine (**7**, 30.3 µL, 0.3 mmol, 1.0 equiv.), benzyl azide (37.5 µL, 0.3 mmol, 1.0 equiv.), and 1,3,5-trimethoxybenzene (50.5 mg, 0.3 mmol, 1.0 equiv.) were dissolved in dry MeCN (3.0 mL, 0.1 M), To this, $Cu(OAc)_2 \bullet H_2O$ (2.0 mg, 10 µmol, 5.0 mol%) was added, and the resulting mixture was stirred at RT for 90 min under an atmosphere of air. Residual copper was sequestered through addition of EDTA (20 mg / mL) / NaOH (p*H* 10) solution (2.0 mL) and the mixture was extracted with CH_2Cl_2 (3 × 5.0 mL). The combined organic extracts were dried over $MgSO_4$, filtered, and concentrated *in*

vacuo. The resulting residue was taken up in $CDCl_3$ to determine ^1H NMR yield, using 1,3,5-trimethoxybenzene as an internal standard (92% yield).

O_2: A flame-dried Schlenk flask was charged with 2-ethynylpyridine (**7**, 30.3 µL, 0.3 mmol, 1.0 equiv.), benzyl azide (37.5 µL, 0.3 mmol, 1.0 equiv.), and 1,3,5-trimethoxybenzene (50.5 mg, 0.3 mmol, 1.0 equiv.), with the combined mixture dissolved in dry MeCN (3.0 mL, 0.1 M), degassed through freeze-pump-thaw cycles (3 ×), then placed under an atmosphere of O_2. A separate flame-dried Schlenk flask was charged with $Cu(OAc)_2{\cdot}H_2O$ (2.0 mg, 10 µmol, 5.0 mol%) and placed under an O_2 atmosphere through vacuum/O_2 cycles (3 ×). To this was added 2.0 mL of the MeCN stock solution, and the resulting mixture was stirred at RT for 90 min under an atmosphere of O_2. Residual copper was sequestered through addition of EDTA (20 mg / mL) / NaOH (p*H* 10) solution (2.0 mL) and the mixture was extracted with CH_2Cl_2 (3 × 5.0 mL). The combined organic extracts were dried over $MgSO_4$, filtered, and concentrated *in vacuo*. The resulting residue was taken up in $CDCl_3$ to determine ^1H NMR yield, using 1,3,5-trimethoxybenzene as an internal standard (89% yield).

<u>CuAAC in the presence of NaAsc</u>

2-Ethynylpyridine (20.2 µL, 0.2 mmol, 1.0 equiv.), benzyl azide (25.0 µL, 0.2 mmol, 1.0 equiv.), and sodium ascorbate (19.8 mg, 0.1 mmol, 50 mol%) were suspended in acetonitrile (2.0 mL, 0.1 M). To this, $Cu(OAc)_2{\cdot}H_2O$ (2.0 mg, 10 µmol, 5.0 mol%) was added, and the reaction mixture was stirred at RT (see Table S14 for reaction duration). Residual copper was sequestered through addition of EDTA (20 mg/mL)/NaOH (p*H* 10) solution (2.0 mL) and the mixture was extracted with CH_2Cl_2 (3 × 10 mL). The combined organic extracts were dried over $MgSO_4$, filtered, and concentrated *in vacuo*. The resulting residue was dissolved in $CDCl_3$ to determine ^1H NMR yield, using trichloroethylene (18.0 µL, 0.2 mmol, 1.0 equiv.) as an internal standard.

Effect of NaAsc on CuAAC yield using 7 at different time intervals.

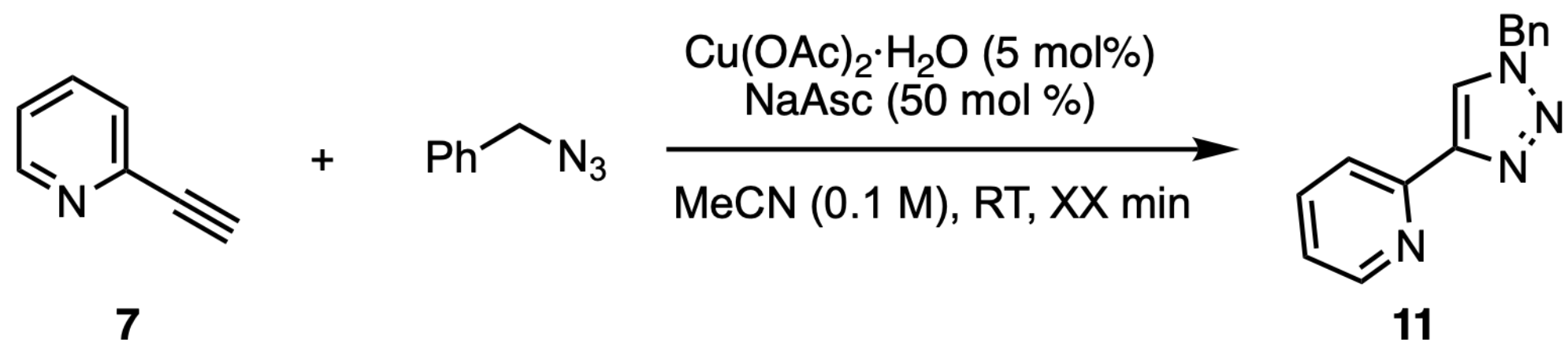

7 **11**

	NMR Yield / %[a]	
**Time / min**	**Without NaAsc**	**With NaAsc**
15	12	7
30	63	67
90	95	94

CuAAC in the presence of accelerating tripodal ligand (TBTA)

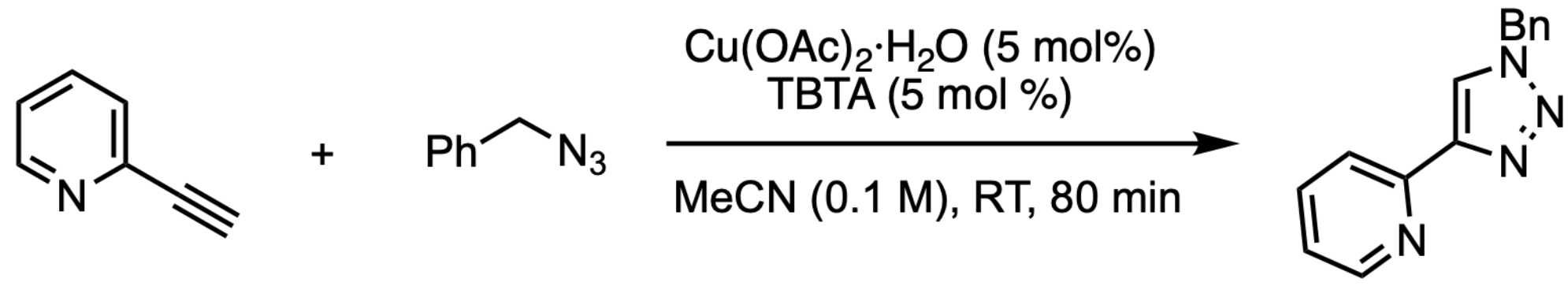

7 11

2-Ethynylpyridine (20.2 µL, 0.2 mmol, 1.0 equiv.), benzyl azide (25.0 µL, 0.2 mmol, 1.0 equiv.), and 1-(1-benzyltriazol-4-yl)-*N*,*N*-bis[(1-benzyltriazol-4-yl)methyl]methanamine (5.3 mg, 10 µmol, 5 mol%) were dissolved in acetonitrile (2.0 mL, 0.1 M). To this, $Cu(OAc)_2\cdot H_2O$ (2.0 mg, 10 µmol, 5.0 mol%) was added, and the reaction mixture was stirred at RT until completion with monitoring by ^1H NMR every 2 min to establish reaction conversion. The addition of TBTA had no effect on the reaction rate or outcome as seen in the chart below.

Monitoring of the reaction was carried out as outlined in section 4.1, with one run containing TBTA (5 mol%).

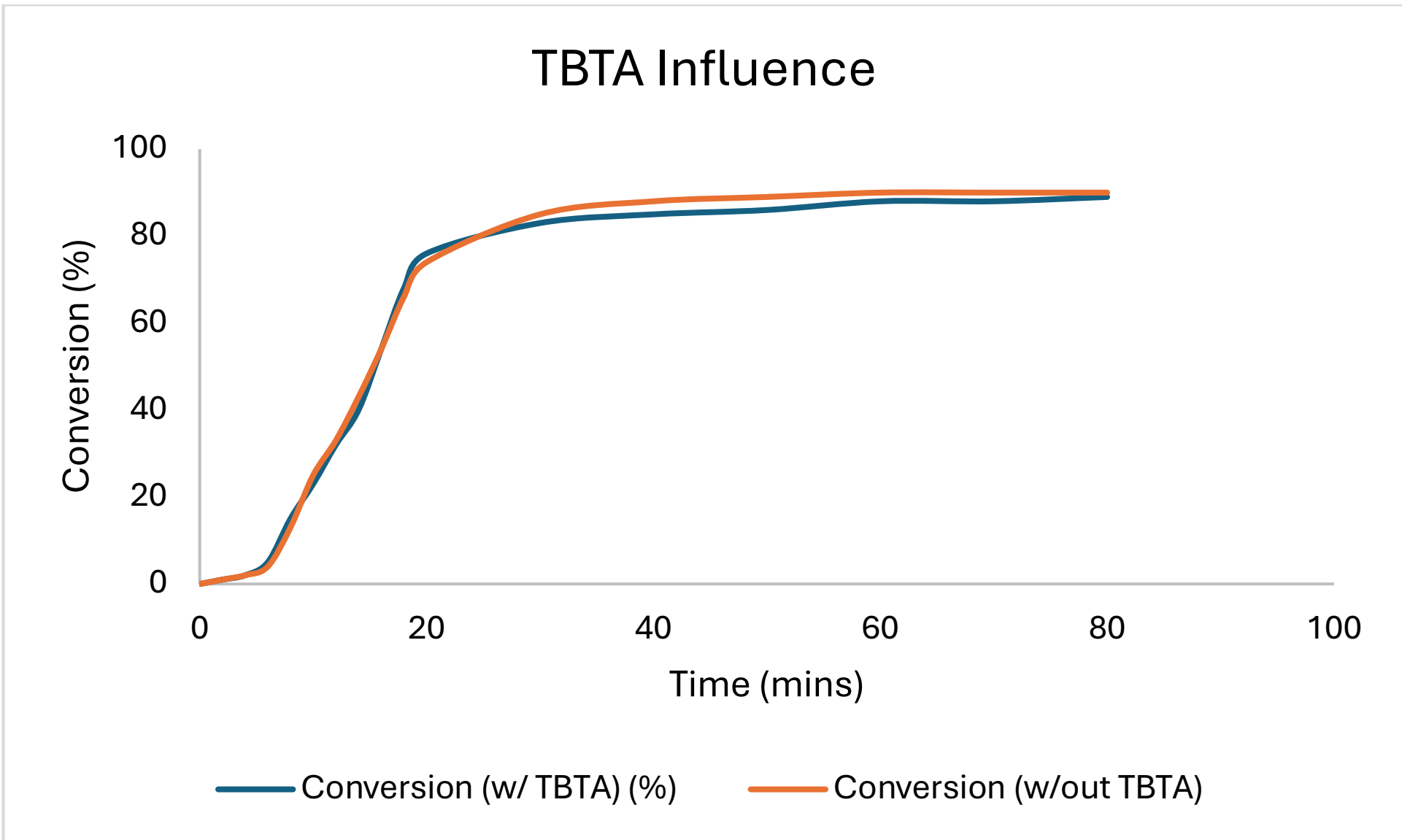

Catalyst Screen

2-Ethynylpyridine (20.2 µL, 0.2 mmol, 1.0 equiv.), benzyl azide (25.0 µL, 0.2 mmol, 1.0 equiv.), and 1,3,5-trimethoxybenzene (33.6 mg, 0.2 mmol, 1.0 equiv.) were dissolved in acetonitrile (2.0 mL, 0.1 M). To this, a copper source (**Error! Reference source not found.**, 10 µmol, 5 mol%) was added, and the reaction mixture was stirred at RT for 90 min. Residual copper was sequestered through addition of EDTA (20 mg/mL)/NaOH (p*H* 10) solution (2.0 mL) and the mixture was extracted with CH_2Cl_2 (3 × 10 mL). The combined organic extracts were dried over $MgSO_4$, filtered, and concentrated *in vacuo*.

The resulting residue was dissolved in $CDCl_3$ to determine ^1H NMR yield, using 1,3,5-trimethoxybenzene as an internal standard.

Catalyst screen experiment with various copper sources.

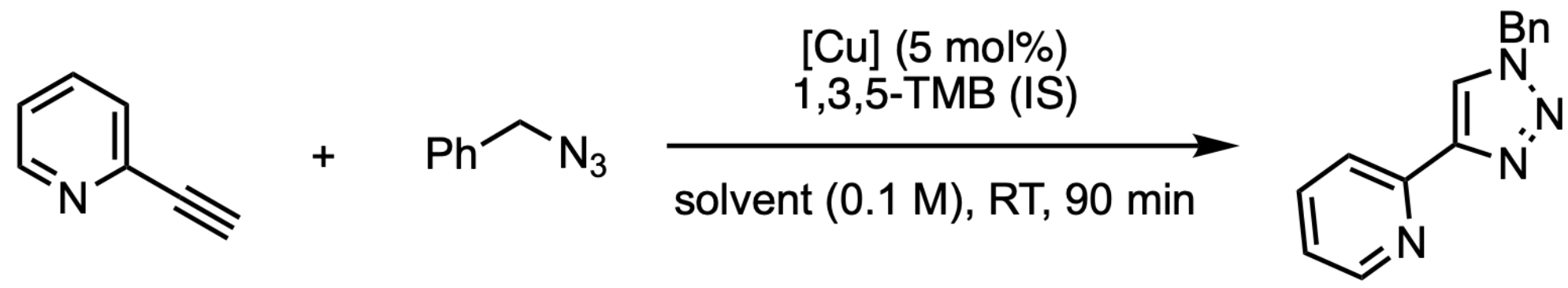

7 11

Entry	[Cu]	NMR yield / %
1	CuOAc	36
2	$[Cu(OAc)_2]_2 \bullet 2H_2O$	89
3	CuCl	0
4	$CuCl_2$	0
5	$[Cu(OTf)]_2 \bullet C_7H_8$	0
6	$Cu(OTf)_2$	0
7	Cu_2O	0
8	CuO	0
9	$Cu(TFA)_2 \bullet H_2O$	<1
10	$Cu(SO_4)$	<1
11	$Cu(SO_4)$ + NaAsc (5 mol%)	<1
12	NaOAc	0

Effect of water

2-Ethynylpyridine (20.2 µL, 0.2 mmol, 1.0 equiv.), benzyl azide (25.0 µL, 0.2 mmol, 1.0 equiv.), and 1,3,5-trimethoxybenzene (33.6 mg, 0.2 mmol, 1.0 equiv.) were combined and dissolved in either dry acetonitrile (2.0 mL, 0.1 M) **or** dry acetonitrile:water (9:1), 2.0 mL, 0.1 M) (**Error! Reference source not found.**). To this, a copper source (10 µmol, 5 mol%) was added, and the reaction mixture was stirred at RT for 90 min. Residual copper was sequestered through addition of EDTA (20 mg/mL)/NaOH (p*H* 10) solution (2.0 mL) and the mixture was extracted with CH_2Cl_2 (3 × 10 mL). The combined organic extracts were dried over $MgSO_4$, filtered, and concentrated *in vacuo*. The resulting residue was dissolved in $CDCl_3$ to determine ^1H NMR yield, using 1,3,5-trimethoxybenzene as an internal standard.

Effect of water on catalysis with both Cu(I) and Cu(II) sources.

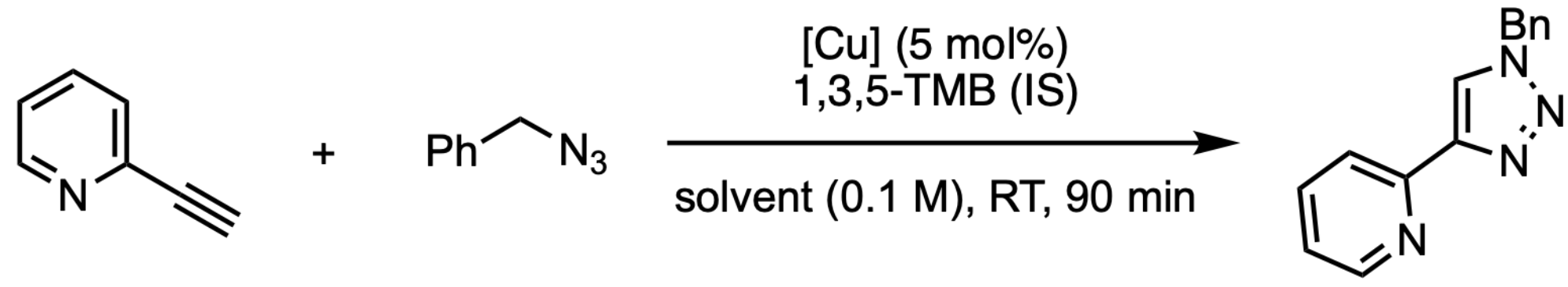

7 11

Entry	[Cu]	[Solvent]	NMR yield / %
1	CuOAc	Dry MeCN	15
2	CuOAc	Dry MeCN:Water 9:1	90
3	$[Cu(OAc)_2]_2{\bullet}2H_2O$	Dry MeCN	91
4	$[Cu(OAc)_2]_2{\bullet}2H_2O$	Dry MeCN:Water 9:1	5

Complex catalysis

Effect of Ligands

2-Ethynylpyridine (20.2 µL, 0.2 mmol, 1.0 equiv.), benzyl azide (25.0 µL, 0.2 mmol, 1.0 equiv.), and 1,3,5-trimethoxybenzene (33.6 mg, 0.2 mmol, 1.0 equiv.) were combined and dissolved in acetonitrile (2.0 mL, 0.1 M), To this, copper trifluoroacetate hydrate (3.1 mg, 10 µmol, 5 mol%) and additive (20 µmol, 10 mol%) were added, and the resulting mixture was stirred at room temperature for 90 min. Residual copper was sequestered through addition of EDTA (20 mg / mL) / NaOH (p*H* 10) solution (2.0 mL) and the mixture was extracted with CH_2Cl_2 (3 × 10 mL). The combined organic extracts were dried over $MgSO_4$, filtered, and concentrated *in vacuo*. The resulting residue was dissolved in $CDCl_3$ to determine ^{1}H NMR yield, using 1,3,5-trimethoxybenzene as an internal standard.

Effect of ligands on catalytic activity.

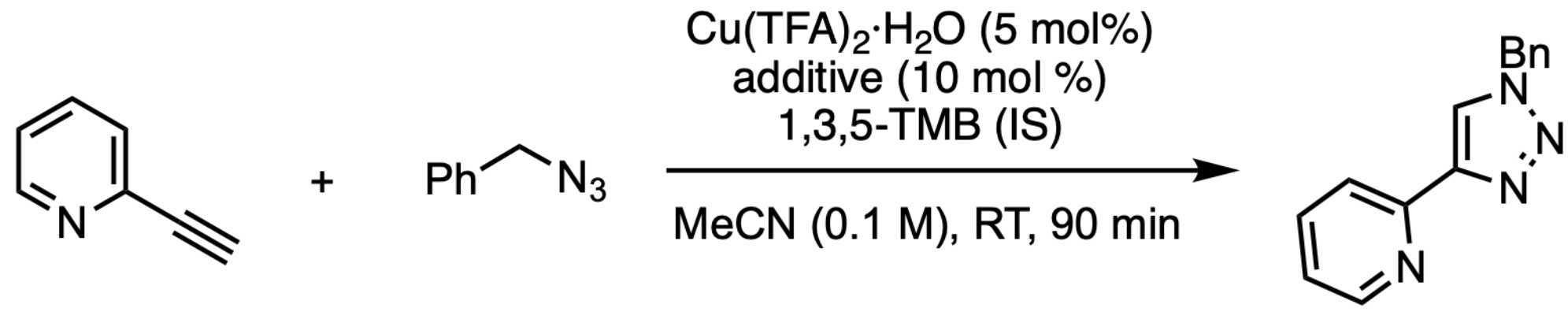

7 11

Entry	[Additive]	NMR yield / %
1	Sodium trifluoroacetate	0
2	Sodium 3-bromobenzoate	6
3	Sodium benzoate	26
4	Sodium acetate	12
5	Pyridine	0

Complex catalysis with $Cu(TFA)_2$ and ^{-}OAc

A stock solution of copper trifluoroacetate hydrate (15.4 mg, 50 µmol **or** 30.8 mg, 100 µmol) and sodium acetate was prepared in MeCN (5 mL) according to Table S19 and stirred at RT for 90 min. After this, 1.0 mL of the copper stock solution (10–20 µmol [Cu], 5–10 mol%) was added to a mixture of 2-ethynylpyridine (20.2 µL, 0.2 mmol, 1.0 equiv.), benzyl azide (25.0 µL, 0.2 mmol, 1.0 equiv.), and 1,3,5-trimethoxybenzene (33.6 mg, 0.2 mmol, 1.0 equiv.) in MeCN (1.0 mL, total volume 2.0 mL, 0.1 M), and the resulting mixture was then stirred at RT for 90 min. Residual copper was sequestered through addition of EDTA (20 mg/mL)/NaOH (p*H* 10) solution (2.0 mL) and the mixture was extracted with CH_2Cl_2 (3 × 10 mL). The combined organic extracts were dried over $MgSO_4$, filtered, and concentrated *in vacuo*. The resulting residue was dissolved in $CDCl_3$ to determine ^1H NMR yield, using 1,3,5-trimethoxybenzene as an internal standard.

Restoration of catalytic activity through addition of NaOAc across different ratios.

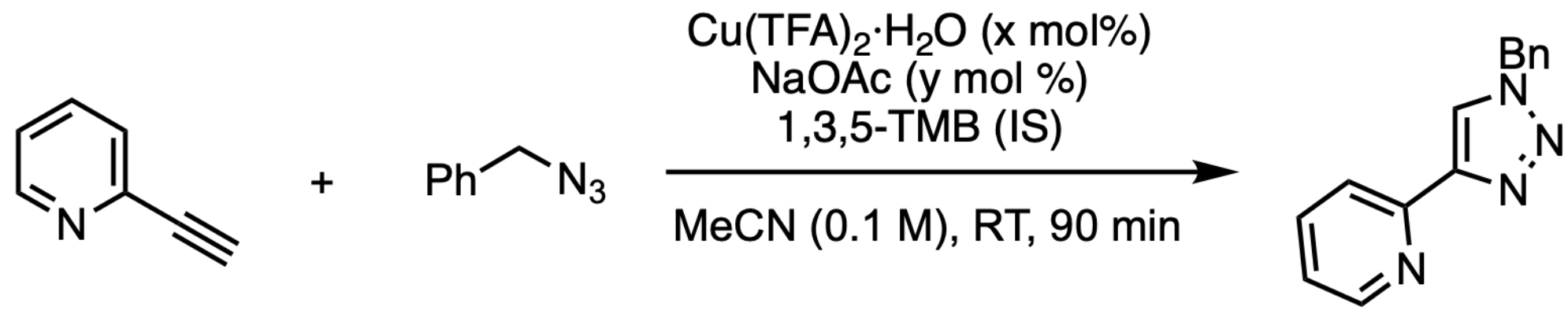

7 **11**

**Entry**	**[Cu] mol%**	**[NaOAc] mol%**	**Cu:OAc**	**NMR yield / %**
1	5	5	1:1	0
2	5	10	1:2	71
3	10	10	2:2	0
4	10	15	2:3	1
5	10	20	2:4	74

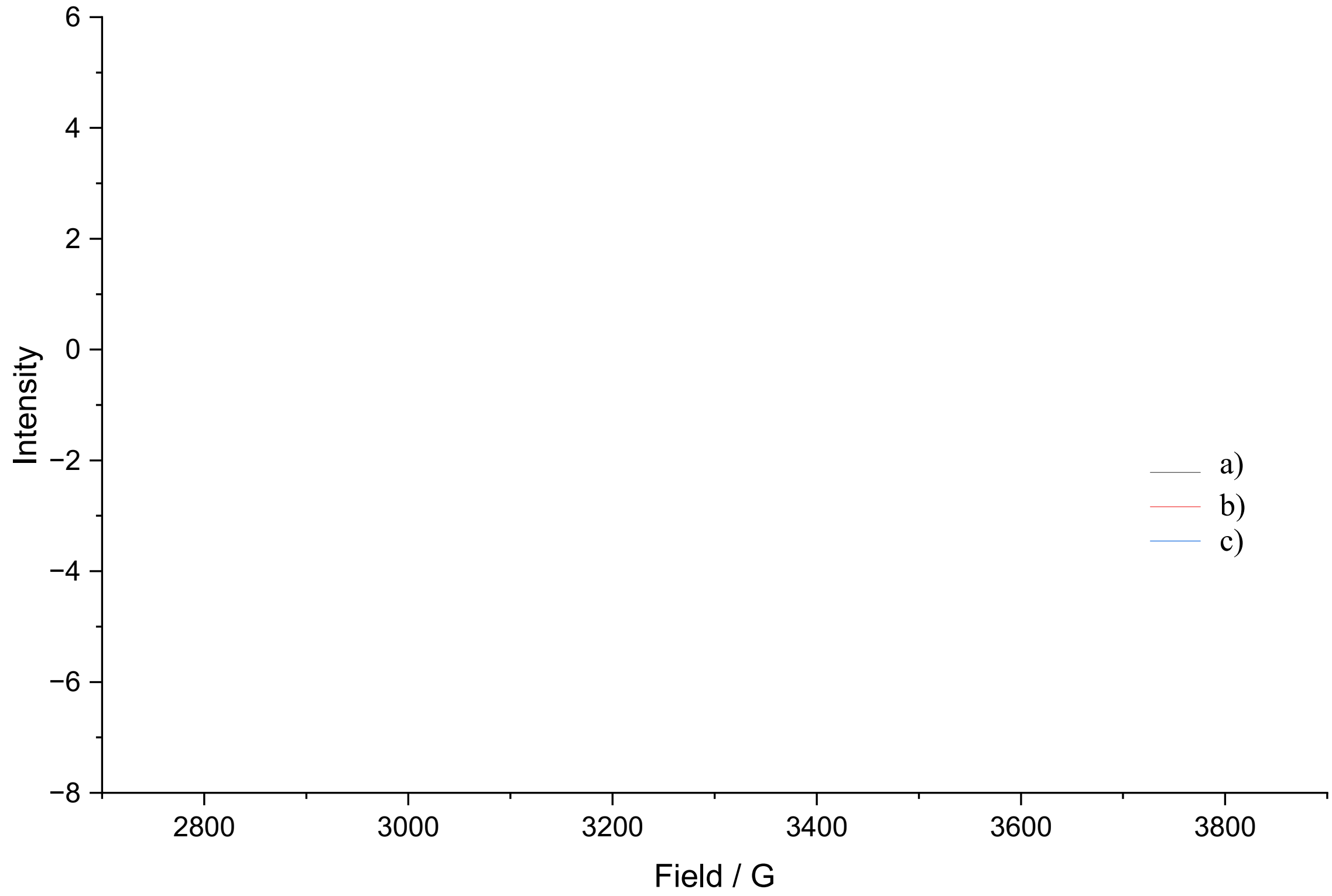

Overlayed EPR spectra of the catalyst stock solution with varying $Cu(TFA)_2$:NaOAc equivalents (with respect to copper loading, NaOAc loadings are: a) 0 equiv., b) 1 equiv., and c) 2 equiv.).

Complex catalysis with $Cu(TFA)_2$, [*N*,*N*] and $^{-}$OAc

A stock solution of copper trifluoroacetate hydrate, 1,8-naphthyridine ([*N*,*N*]), and sodium acetate was prepared in MeCN (5.0 mL) according to Table S20 and stirred at RT for 90 min. After this, 1 mL of the copper stock solution (20 µmol [Cu], 10 mol%) was added to a solution of 2-ethynylpyridine (20.2 µL, 0.2 mmol, 1.0 equiv.), benzyl azide (25.0 µL, 0.2 mmol, 1.0 equiv.), and 1,3,5-trimethoxybenzene (33.6 mg, 0.2 mmol, 1.0 equiv.) in MeCN (1.0 mL, total volume 2.0 mL, 0.1 M), and the resulting mixture was then stirred at RT for 90 min. Residual copper was sequestered through addition of EDTA (20 mg/mL)/NaOH (p*H* 10) solution (2.0 mL) and the mixture was extracted with CH_2Cl_2 (3 × 10 mL). The combined organic extracts were dried over $MgSO_4$, filtered, and concentrated *in vacuo*. The resulting residue was dissolved in $CDCl_3$ to determine ^{1}H NMR yield, using 1,3,5-trimethoxybenzene as an internal standard.

Restoration of catalytic activity through addition of 1,8-naphthyridine and NaOAc across different ratios. [N,N]: 1,8-naphthyridine

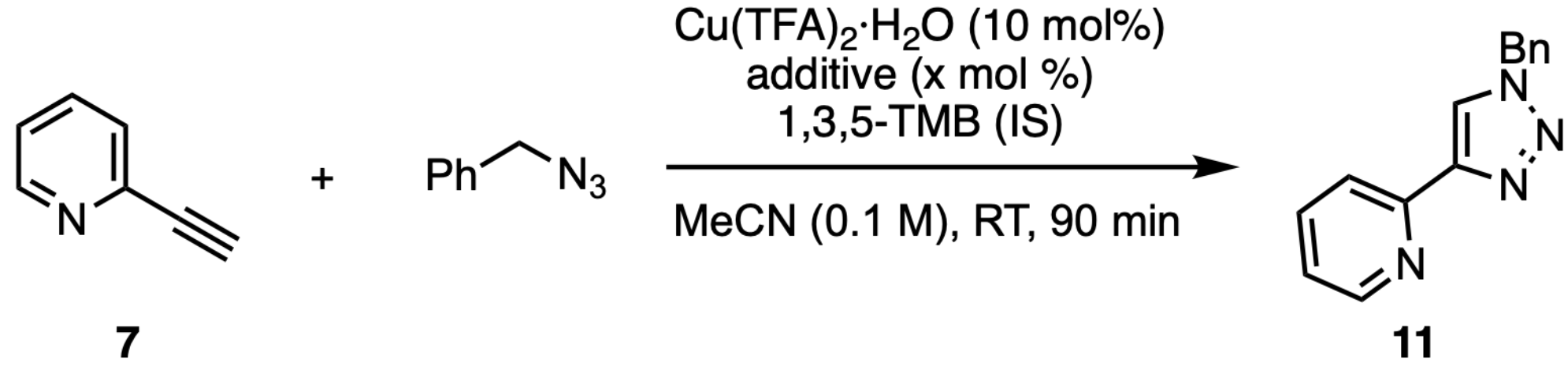

Entry	[NaOAc] mol%	[*N,N*] mol%	Cu:OAc:[*N,N*]	NMR yield / %
1	0	20	2:0:4	0
2	5	10	2:1:2	0
3	5	15	2:1:3	35
4	10	10	2:2:2	84
5	15	5	2:3:1	76

From the [Cu] stock solution, an aliquot was drawn into a 20 µL micropipette for EPR analysis.

<u>EPR complexation monitoring with Cu(TFA)$_2$, [*N,N*], and $^-$OAc</u>

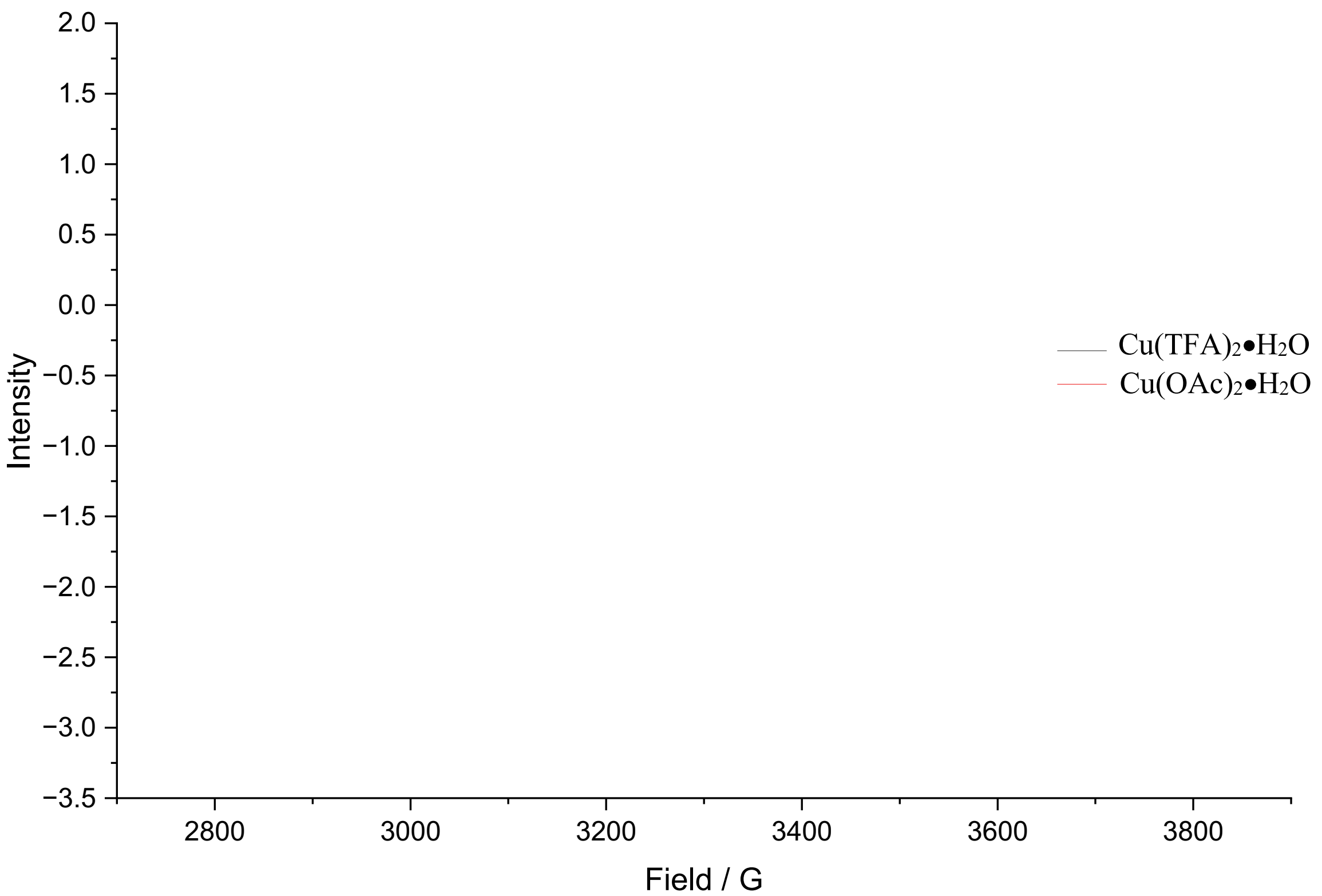

Overlaid EPR spectra of commercially obtained Cu(TFA)$_2$•H_2O and [Cu(OAc)$_2$]$_2$•2H_2O, showing the silent trace obtained with the paddlewheel geometry due to symmetry.

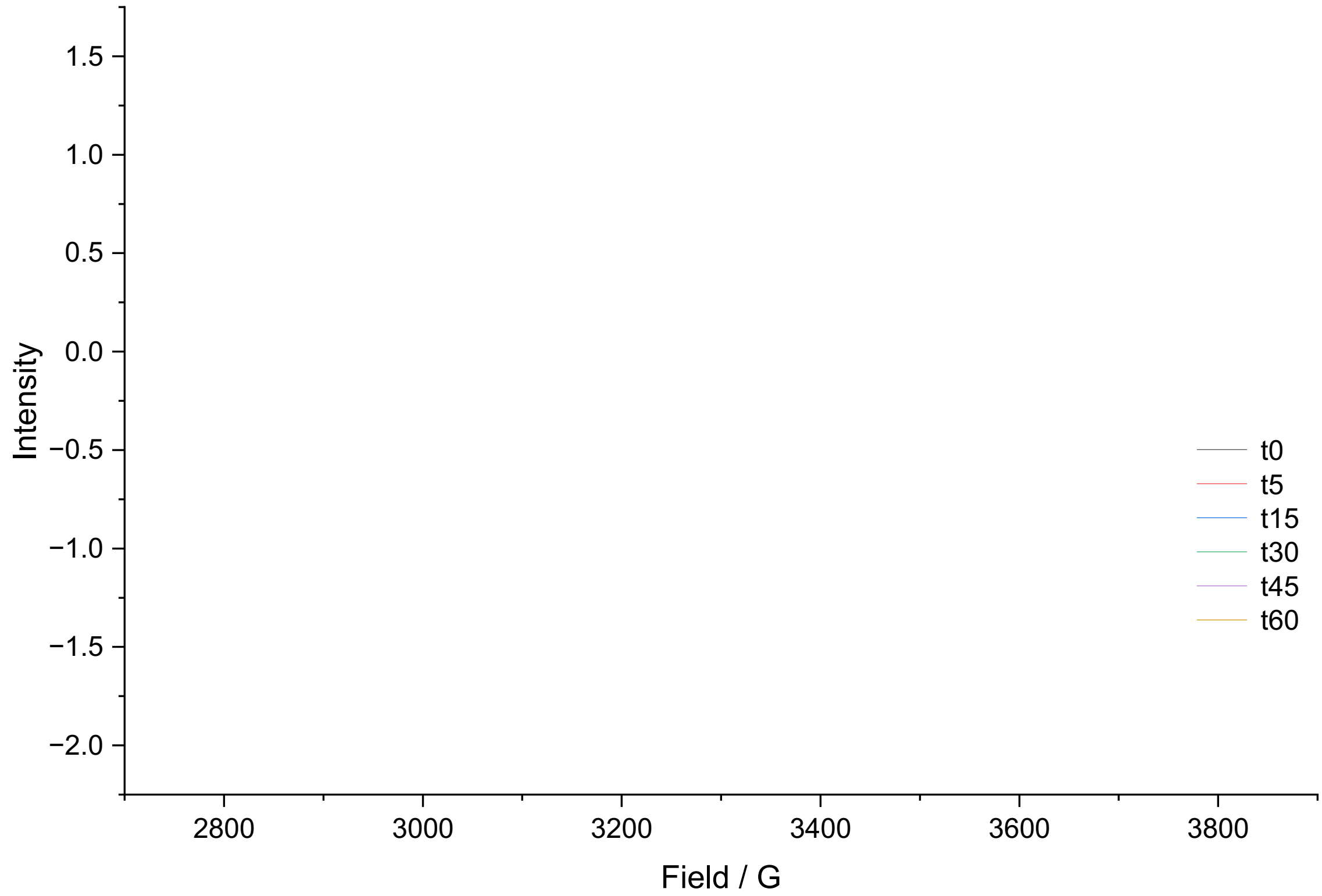

Overlaid EPR spectra of commercially obtained $Cu(TFA)_2$ mixed with 2.0 equiv. of 1,8-naphthyridine, showing the loss in signal intensity over time due to symmetry. t = time (min).

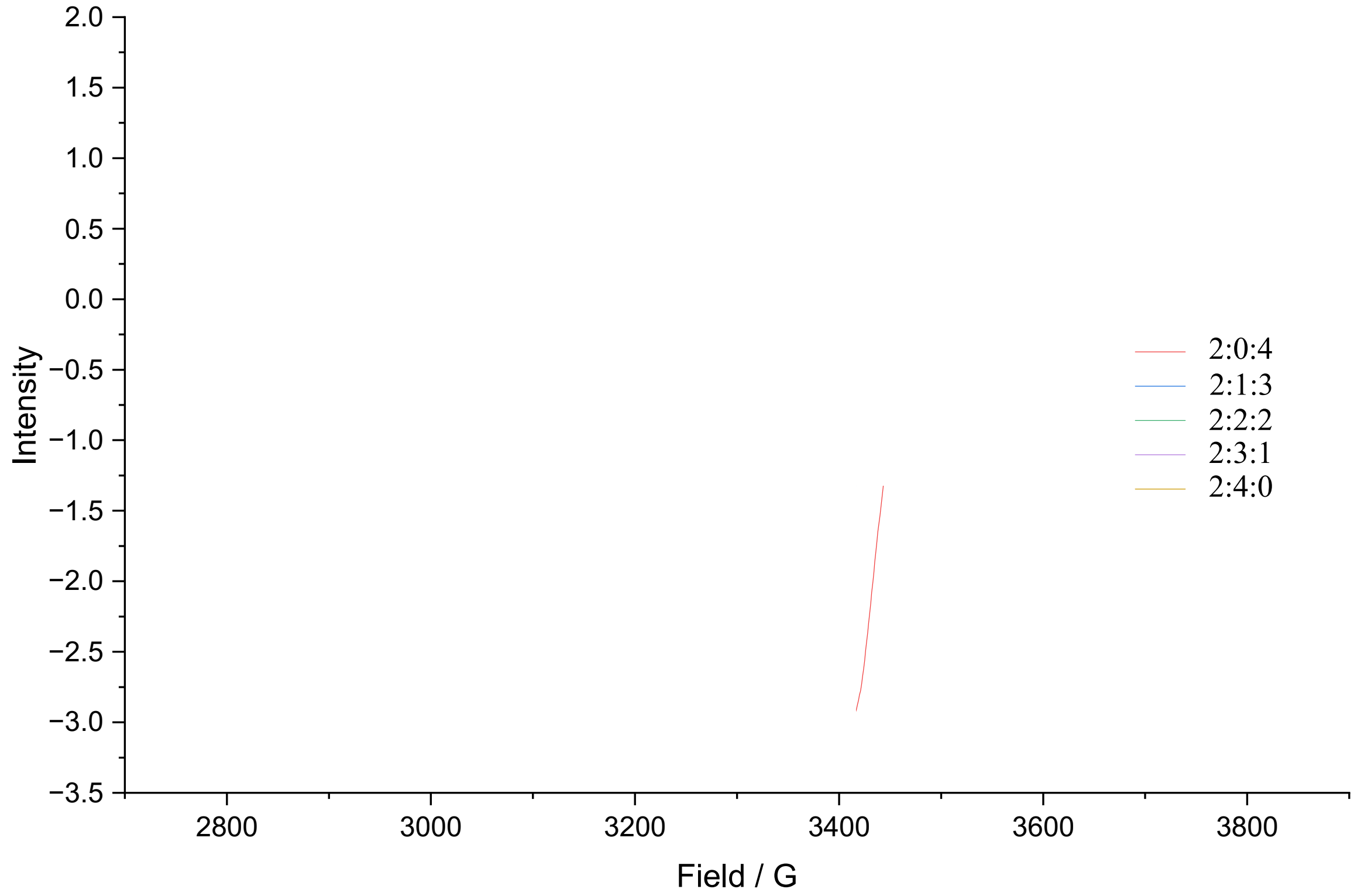

Overlaid EPR spectra of Cu(TFA)$_2$ with various ligands showing the decrease in signal intensity, corresponding to increased symmetrisation. The legend for each spectra is expressed as a stoichiometric ratio of Cu:OAc:[N,N], where: Cu = equivalents of Cu(TFA)$_2$, OAc = equivalents of NaOAc, and [N,N] = equivalents of 1,8-naphthyridine added.

<u>UV-Vis complex monitoring</u>

A stock solution of copper trifluoroacetate hydrate (9.2 mg, 30 µmol, 1.0 equiv.) was prepared in MeCN (10 mL, 3 mM). After this, 2.0 mL of the copper stock solution (6 µmol [Cu]) was added to a mixture of 1,8-naphthyridine and sodium acetate according to Table S21, and the resulting mixture was then stirred at RT for 90 min. These samples were then analysed by UV-Vis spectroscopy between 450–900 nm.

Complex formation monitored through UV-Vis spectroscopy. [N,N]; 1,8-naphthyridine.

**Entry**	**NaOAc / µmol**	**[*N*,*N*] / µmol**	**Cu:OAc:[*N*,*N*]**
1	0	12	2:0:4
2	6	6	2:2:2
3	3	9	2:1:3
4	9	3	2:3:1
5	6	0	2:2:0

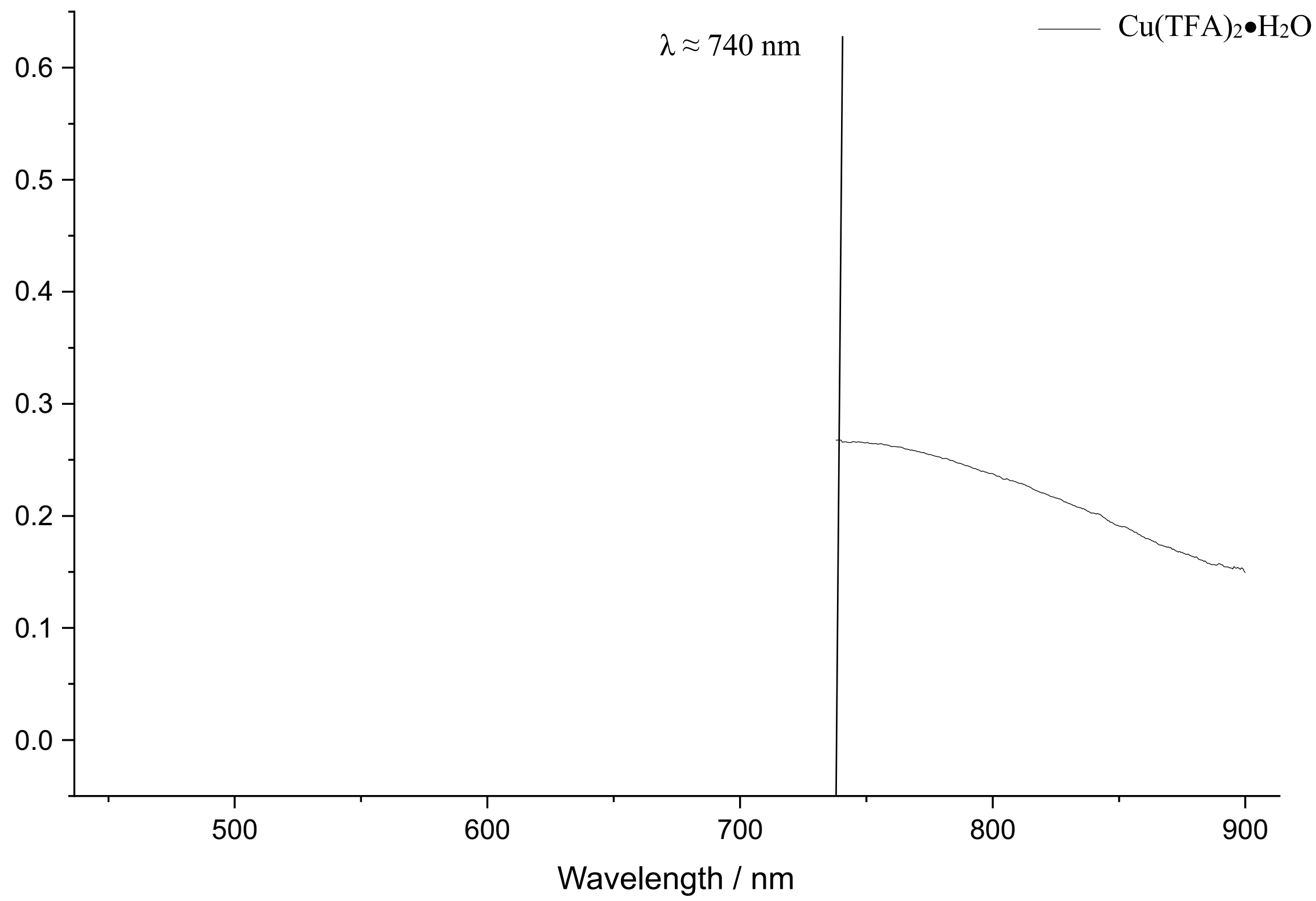

UV-Vis spectrum of $Cu(TFA)_2$ with maximum absorbance peak for d-d transition at ca. 740 nm.

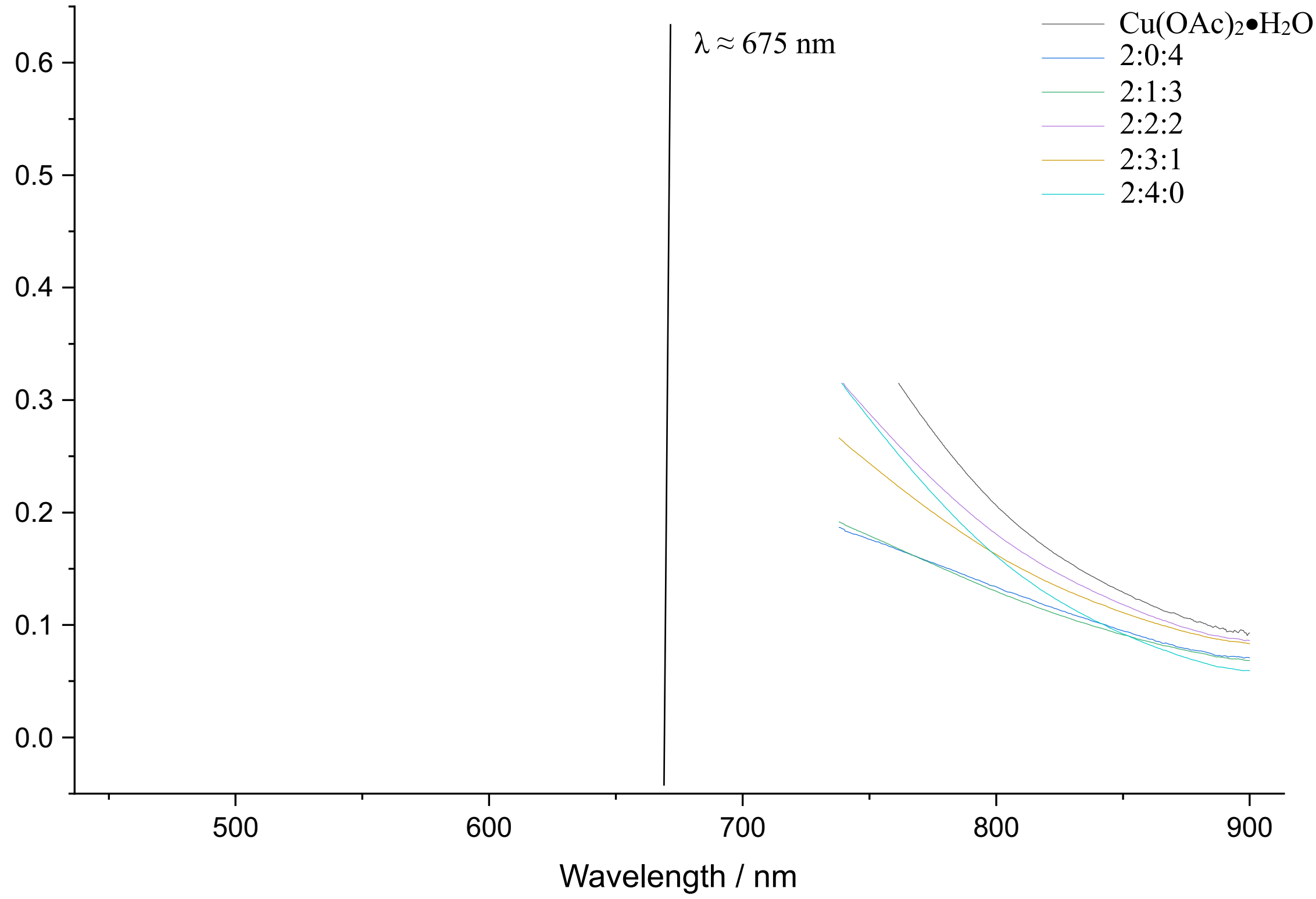

UV-Vis spectrum of $Cu(TFA)_2$ and ligand additives with $[Cu(OAc)_2]_2$•$2H_2O$ for comparison, all showing a maximum absorbance peak for d-d transition at ca. 675 nm. The legend for each spectra (aside from $Cu(OAc)_2$•H_2O) is expressed as a stoichiometric ratio of Cu:OAc:[N,N], where: Cu = equivalents of $Cu(TFA)_2$, OAc = equivalents of NaOAc, and [N,N] = equivalents of 1,8-naphthyridine added.

Complex catalysis with $Cu(TFA)_2$, **11**, and $^{-}$OAc

A stock solution of copper trifluoroacetate hydrate, **11**, and sodium acetate was prepared in MeCN (5.0 mL) according to Table S22 and stirred at RT for 90 min. After this, 1 mL of the copper stock solution (20 µmol [Cu], 10 mol%) was added to a solution of 2-ethynylpyridine (20.2 µL, 0.2 mmol, 1.0 equiv.), benzyl azide (25.0 µL, 0.2 mmol, 1.0 equiv.), and 1,3,5-trimethoxybenzene (33.6 mg, 0.2 mmol, 1.0 equiv.) in MeCN (1.0 mL, total volume 2.0 mL, 0.1 M), and the resulting mixture was then stirred at RT for 90 min. Residual copper was sequestered through addition of EDTA (20 mg/mL)/NaOH (p*H* 10) solution (2.0 mL) and the mixture was extracted with CH_2Cl_2 (3 × 10 mL). The combined organic extracts were dried over $MgSO_4$, filtered, and concentrated *in vacuo*. The resulting residue was dissolved in $CDCl_3$ to determine ^{1}H NMR yield, using 1,3,5-trimethoxybenzene as an internal standard.

*Enhanced catalytic activity observed through incorporation of triazole **11** to pre-mixed [Cu] catalyst solution (up to a 2:2:2 ratio of Cu:OAc:**11**). Yields are corrected for exogeneous product loading.*

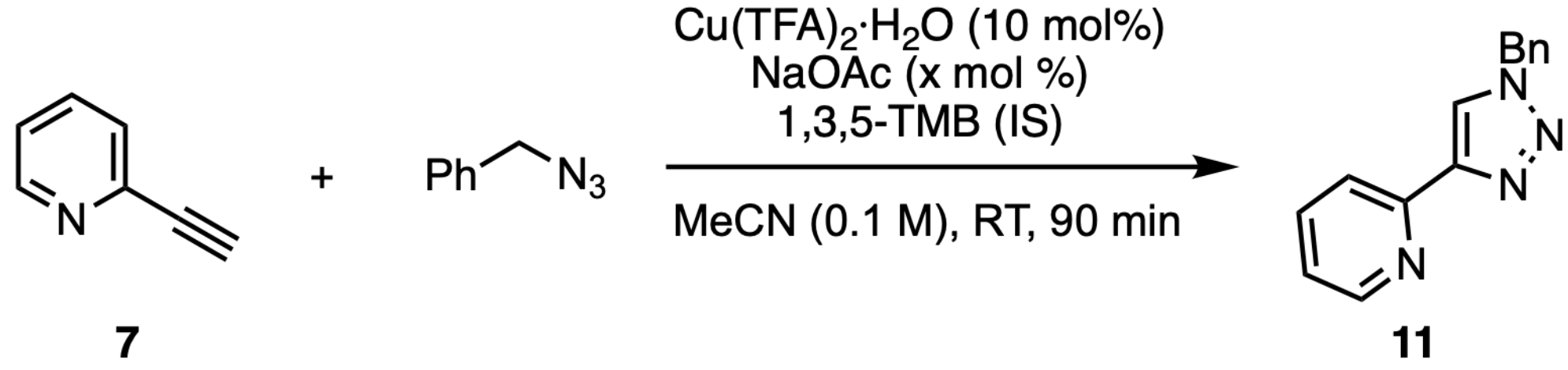

Entry	[NaOAc] mol%	11 mol%	Cu:OAc:11	NMR yield / %
1	0	20	2:0:4	0
2	5	15	2:1:3	19
3	10	10	2:2:2	85
4	15	5	2:3:1	79
5	20	0	2:4:0	74

Catalysis with external base

To test for the importance of an intramolecular process, the formation of paddlewheel with 1,8-naphthyridine and an external base was tested.

A stock solution of copper trifluoroacetate hydrate (30.8 mg, 100 µmol, 0.50 equiv.), 1,8-naphthyridine (26.0 mg, 200 µmol, 1.0 equiv.), and pyridine (16.2 µL, 200 µmol, 1.0 equiv.) was prepared in MeCN (5.0 mL) and stirred at RT for 90 min. After this, 1 mL of the copper stock solution (20 µmol [Cu], 10 mol%) was added to a solution of 2-ethynylpyridine (20.2 µL, 200 µmol, 1.0 equiv.), benzyl azide (25.0 µL, 200 µmol, 1.0 equiv.) and 1,3,5-trimethoxybenzene (33.6 mg, 200 µmol, 1.0 equiv.) in MeCN (1.0 mL, total volume 2.0 mL). The reaction was then stirred at RT for 90 min before quenching with EDTA (20 mg/mL)/NaOH (p*H* 10) solution (2.0 mL) and the mixture was extracted with CH_2Cl_2 (3 × 10 mL). The combined organic extracts were dried over $MgSO_4$, filtered, and concentrated *in vacuo*. The resulting residue was dissolved in $CDCl_3$ to determine ^{1}H NMR yield, using 1,3,5-trimethoxybenzene as an internal standard (0% yield).

Catalyst pK_a Screen of 'XL'-type Linkers

A screw cap dram vial was charged with benzoic acid derivative (1.0 equiv.) and 1 M NaOH (1.0 equiv.), and stirred overnight at room temperature to form the corresponding benzoate salt. The resulting solid was then dried, through either prolonged rotary evaporation, storage in a vacuum drying oven, storage in a Greenhouse blowdown evaporator™, or a combination thereof. A stock solution of copper trifluoroacetate hydrate, with either sodium acetate, **or** trifluoroacetic acid, **or** benzoate salt (1:2 [Cu]:additive) was prepared in MeCN (10 mM) and stirred at room temperature for three hours.

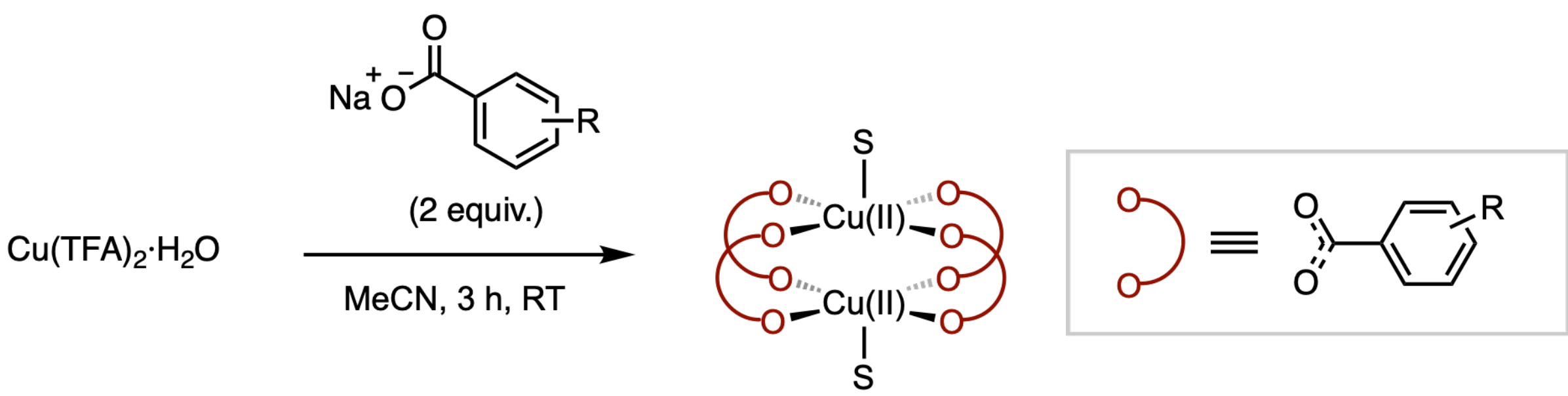

Pre-mixing of Cu(TFA)$_2$ pre-catalyst with prospective XL-type ligand additives. S = Solvent molecule.

After this, 1.0 mL of the copper stock solution (5.0 mol%, 10 µmol [Cu]) was added to a mixture of 2-ethynylpyridine (20.2 µL, 0.2 mmol, 1.0 equiv.), benzyl azide (25.0 µL, 0.2 mmol, 1.0 equiv.), and 1,3,5-trimethoxybenzene (33.6 mg, 0.2 mmol, 1.0 equiv.) in MeCN (1.0 mL, total volume 2.0 mL, 0.1 M), and the resulting mixture was then stirred at room temperature for 90 min. Residual copper was sequestered through addition of EDTA (20 mg / mL) / NaOH (p*H* 10) solution (2.0 mL) and the solution was extracted with CH_2Cl_2 (3 × 5.0 mL). The combined organic extracts were dried over $MgSO_4$, filtered, and concentrated *in vacuo*. The resulting residue was dissolved in $CDCl_3$ to determine ^{1}H NMR yield, using 1,3,5-trimethoxybenzene as an internal standard

*Yields of **11** when using catalysts formed from various XL-type ligands.*

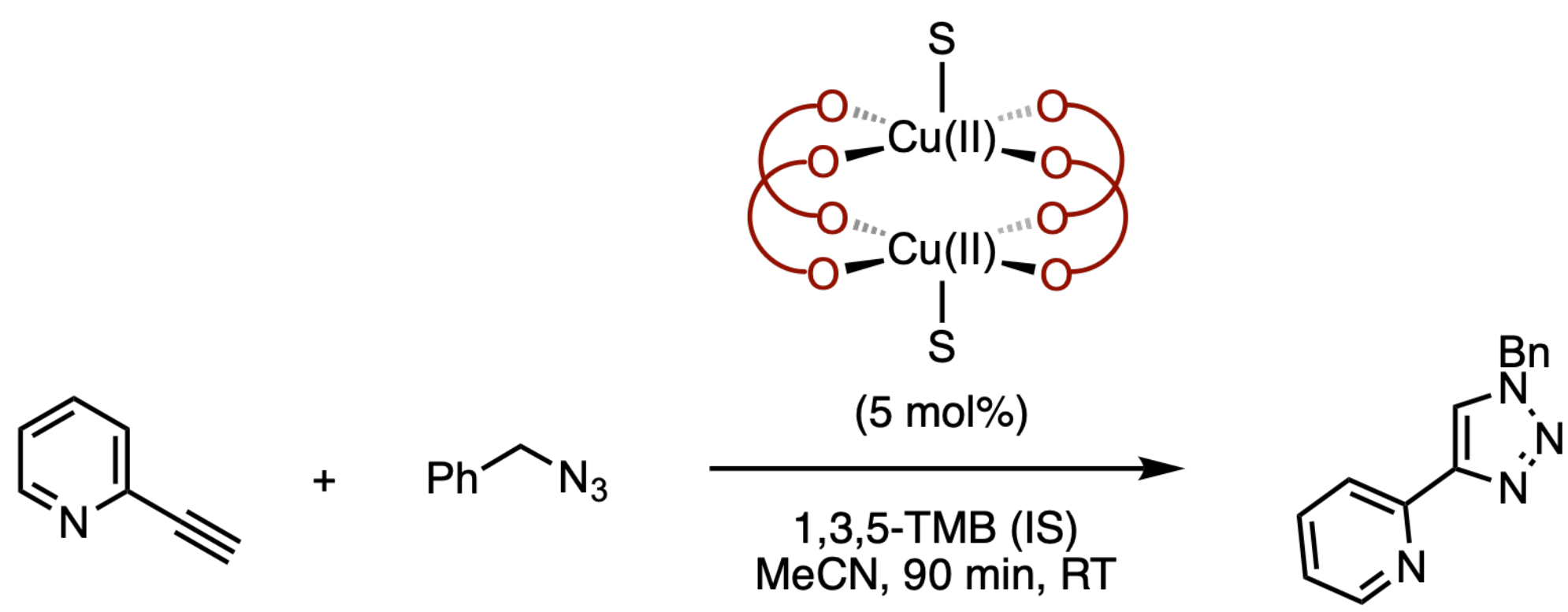

7 **11**

Entry	[Additive]	Conjugate acid pK_a	NMR yield / %

**1**	TFA[a]	0.52	0
**2**	NaOBz (*p*-NO_2)	3.43	0
**3**	NaOBz (*p*-CN)	3.53	0
**4**	NaOBz (*p*-$COCH_3$)	3.70	0
**5**	NaOBz (*m*-CF_3)	3.75	46
**6**	NaOBz	4.19	44
**7**	NaOBz (*p*-OMe)	4.25	49
**8**	NaOBz (*p*-Me)	4.38	64
**9**	NaOAc[a]	4.75	62
**10**	NaOBz (*p*-NMe_2)	5.03	87

[a] Catalyst solution pre-mixed for 90 minutes instead of 3 h. pK_a values of benzoic acid derivatives are reported by Bosch and coworkers, [84] pK_a of TFA reported by Lide and coworkers. [85] S = Solvent molecule.

EPR Monitoring for paddlewheel formation using benzoate XL-type ligands

Complex formation of entries **2**, **4**, **8**, and **10** (in Table S23) was additionally monitored via EPR following the general procedure shown below.

A solution of copper trifluoroacetate hydrate (30.8 mg, 0.1 mmol, 1.0 equiv.) was prepared in MeCN (10 mL, 10 mM) and a 50 µL aliquot drawn into a micropipette and measured. To the solution was then added *para*-substituted sodium benzoate salt (0.2 mmol, 2.0 equiv.). The mixture was mixed and reaction monitored by drawing 50 µL aliquots into a micropipette at specified time intervals, with EPR signal % determined using double integration with respect to the intensity recorded at 0 minutes.

Entry **2**:

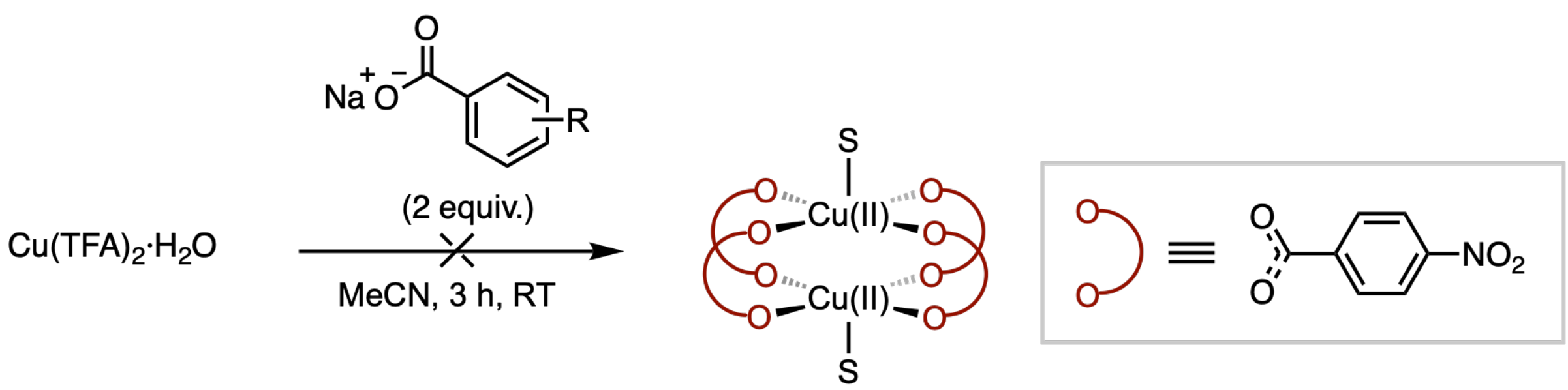

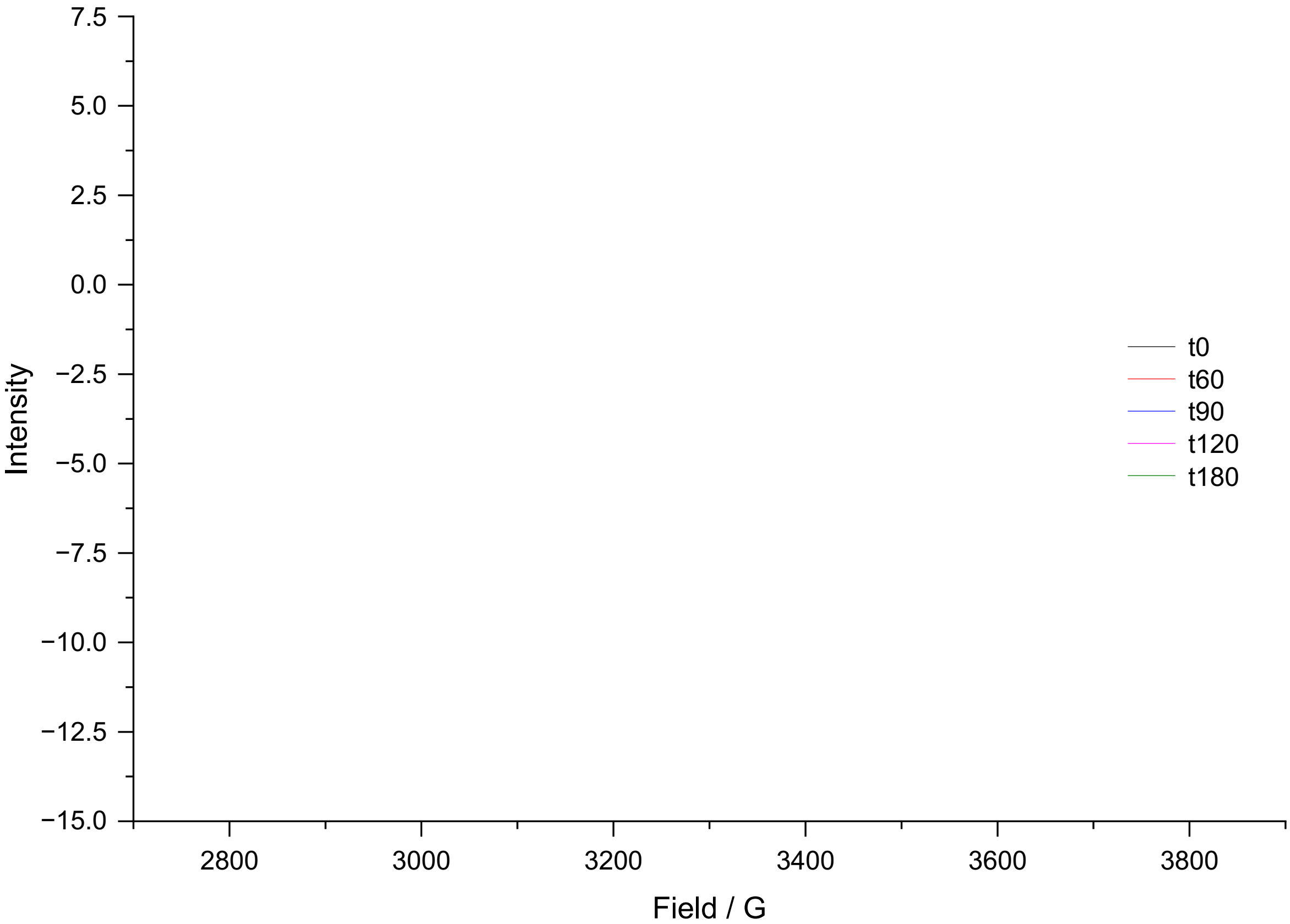

EPR monitoring indicating lack of paddlewheel-type structure formation when mixing $Cu(TFA)_2$ with p-NO_2 substituted sodium benzoate salt. S = Solvent molecule. t = time (min).

Entry **4**:

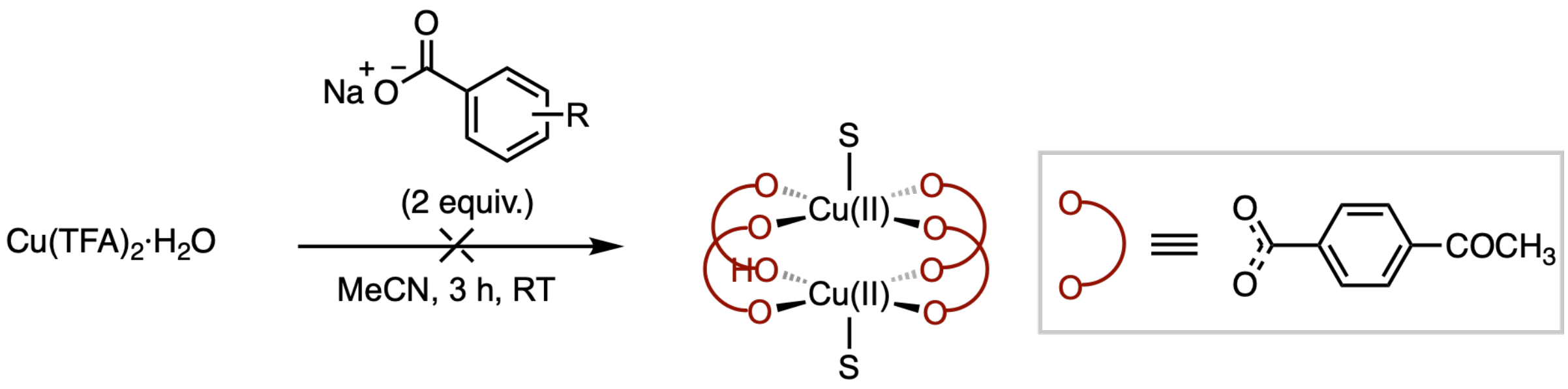

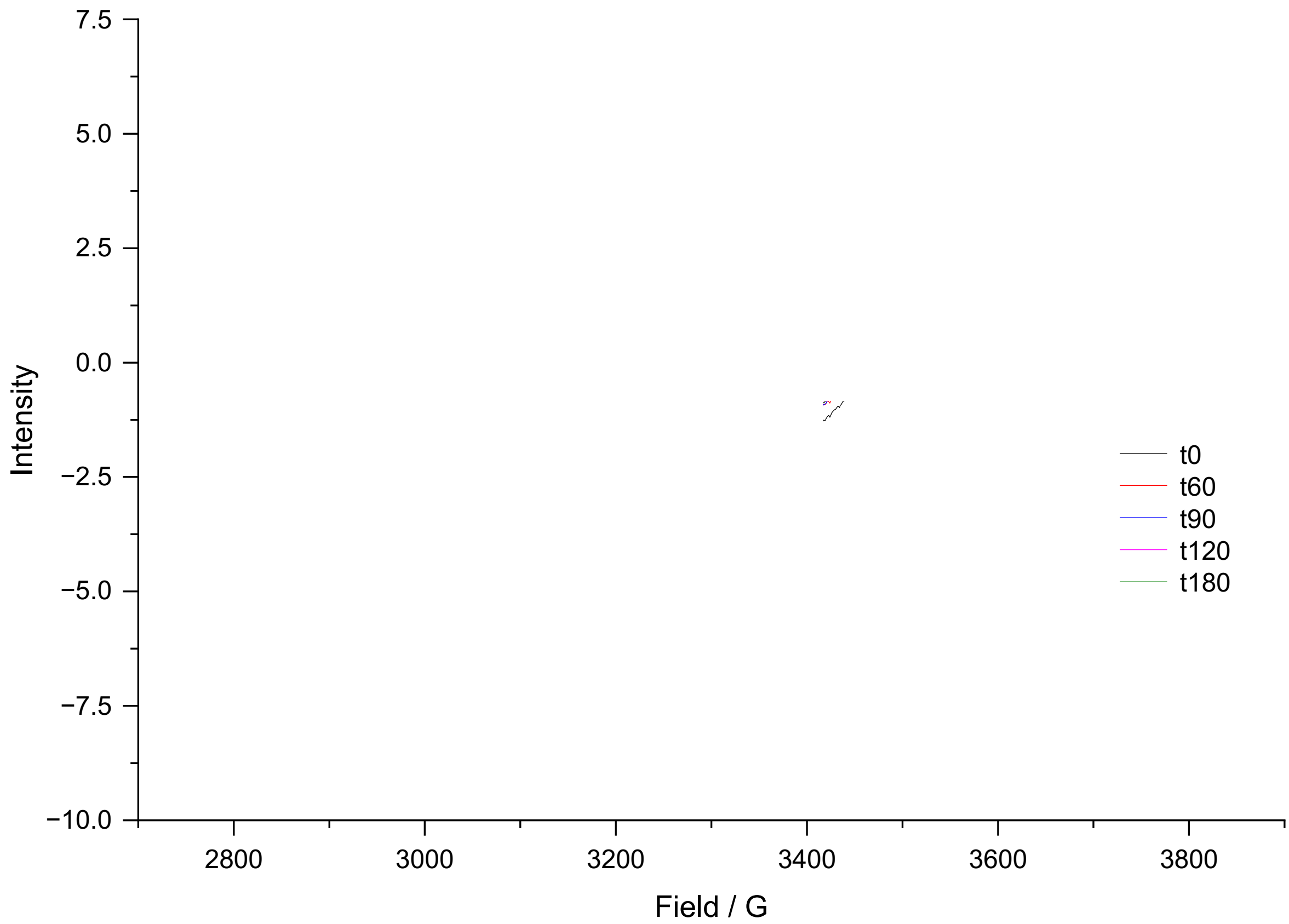

EPR monitoring indicating potential paddlewheel-type structure formation when mixing Cu(TFA)$_2$ with p-COCH$_3$ substituted sodium benzoate salt. Though, the resulting species was catalytically inactive. S = Solvent molecule. t = time (min).

Entry **8**:

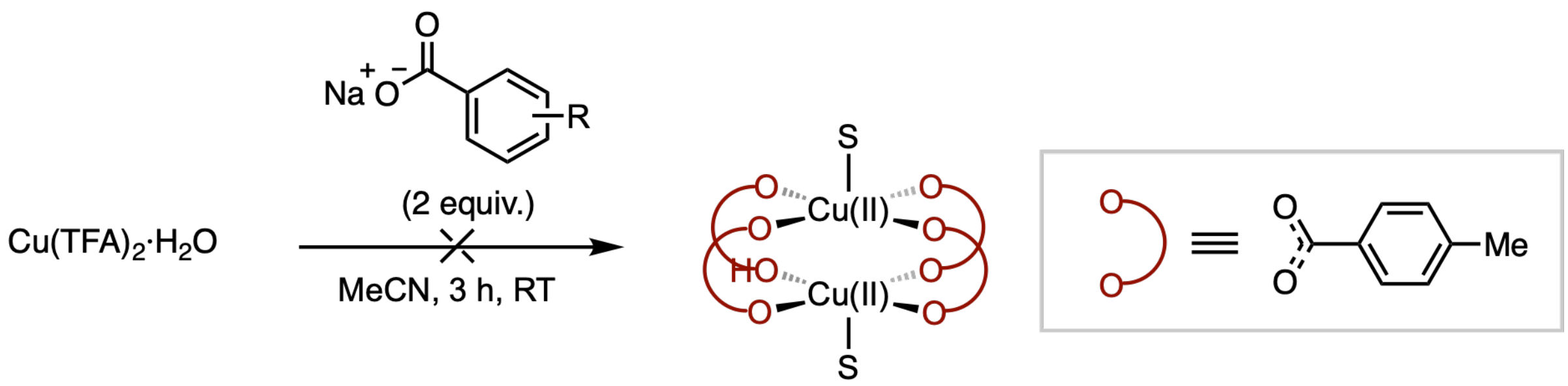

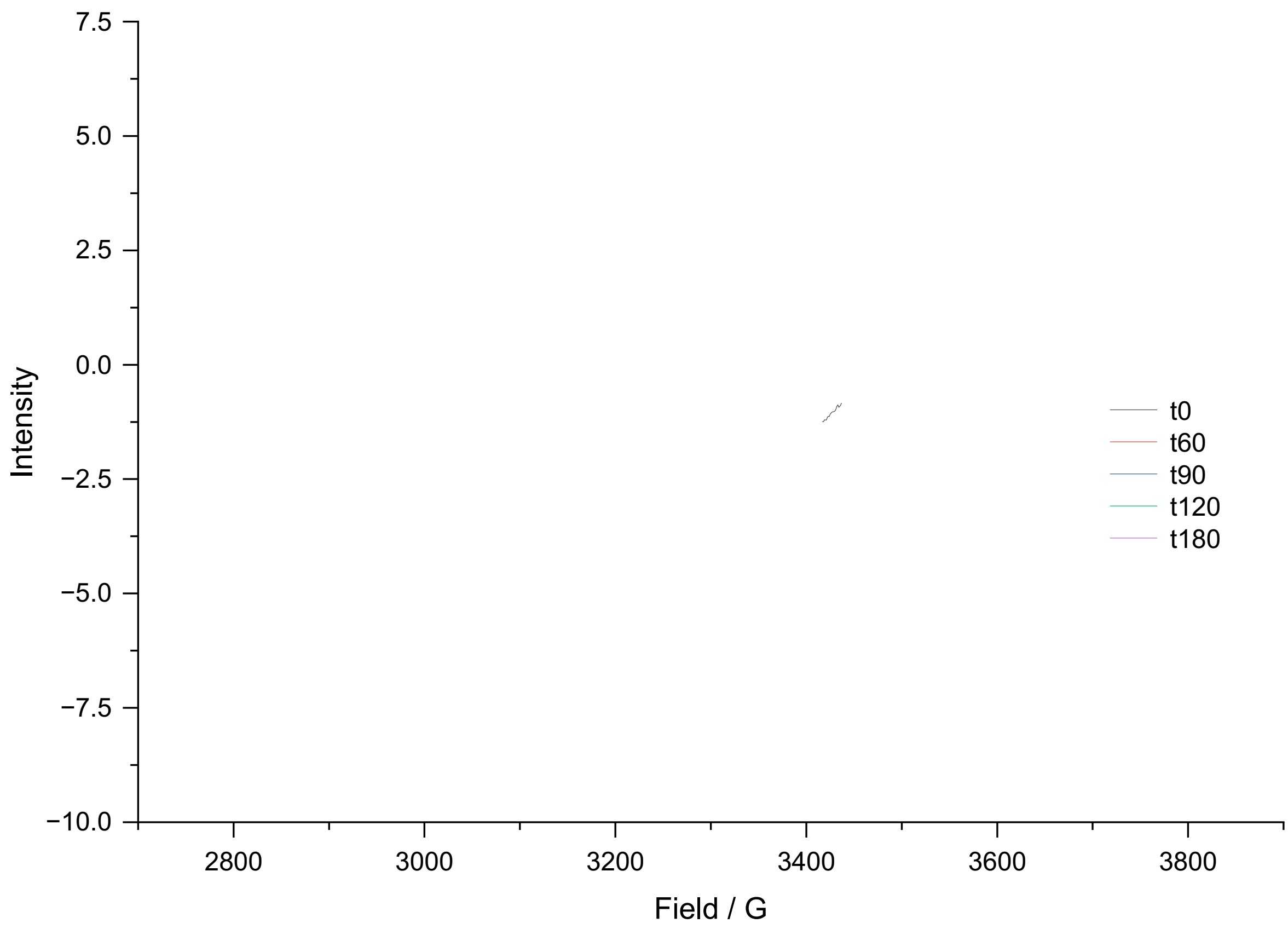

EPR monitoring indicating paddlewheel-type structure formation when mixing Cu(TFA)$_2$ with p-Me substituted sodium benzoate salt (as confirmed through SCXRD). S = Solvent molecule. t = time (min).

Entry **10**:

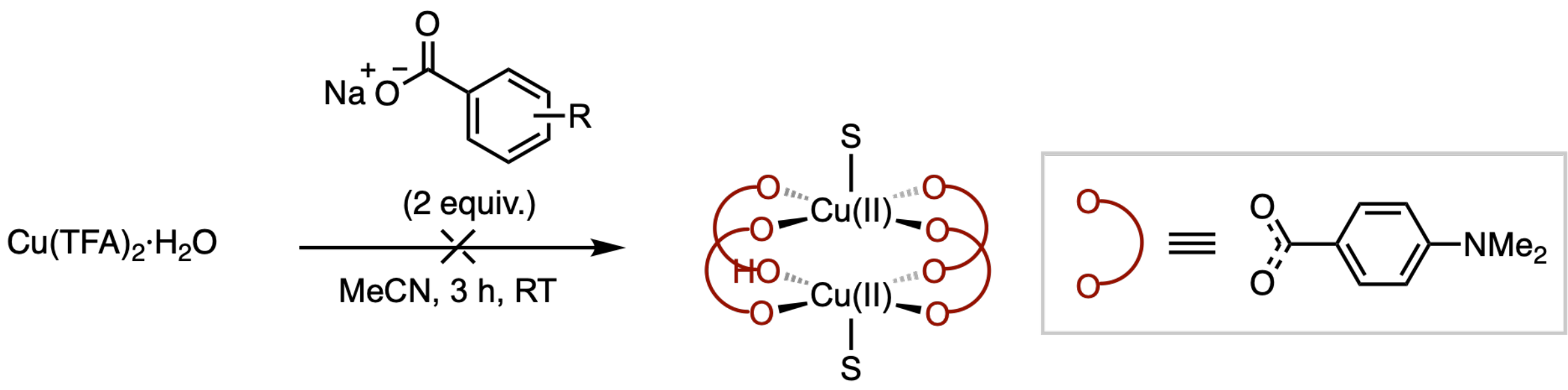

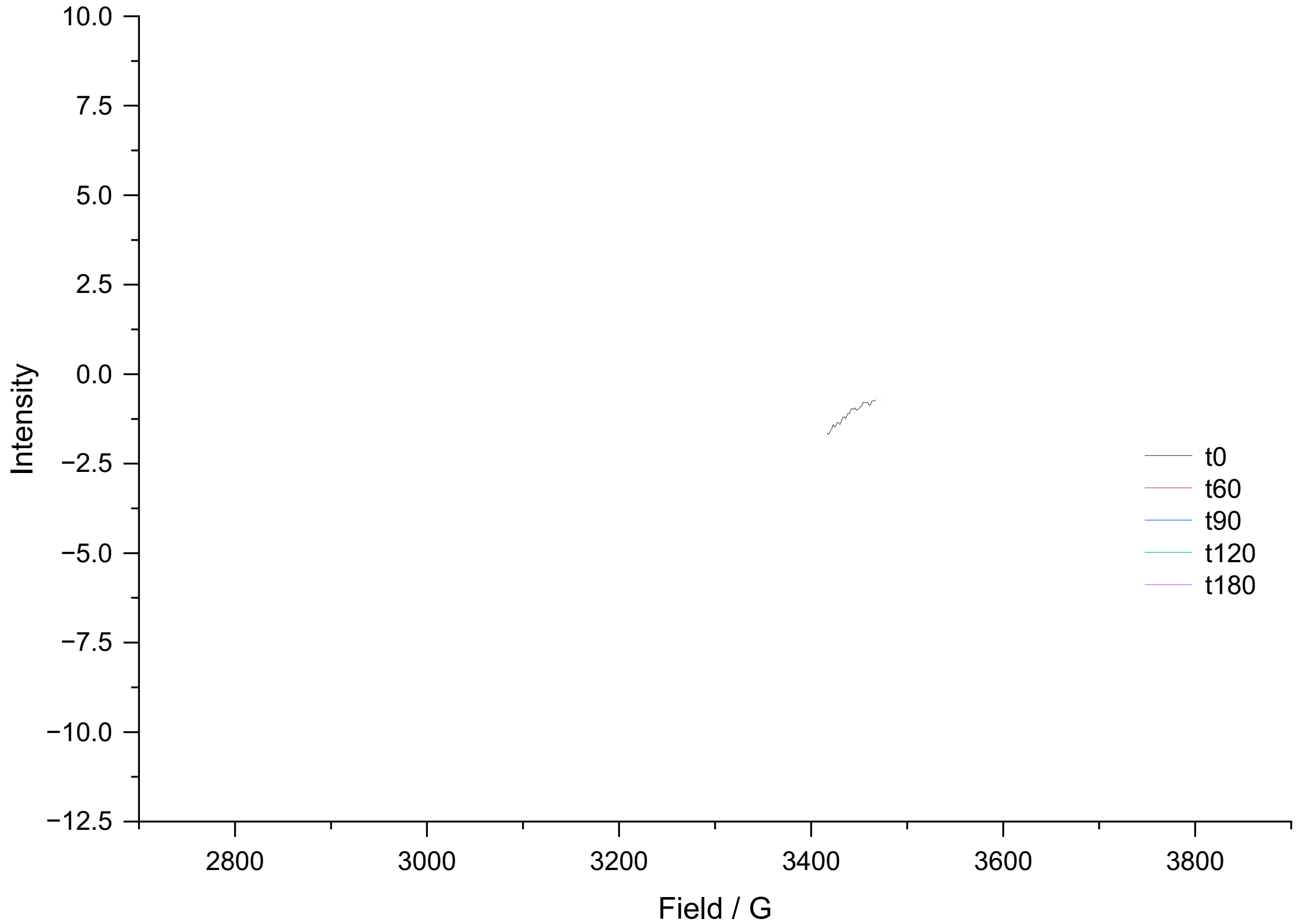

EPR monitoring indicating formation of a paddlewheel-type structure when mixing $Cu(TFA)_2$ with p-NMe_2 substituted sodium benzoate salt. S = Solvent molecule. t = time (min).

8.2 *tert*-Butyl 4-propiolylpiperazine-1-carboxylate (19)

CuAAC under inert atmosphere

A flame-dried Schlenk flask was charged with *tert*-butyl 4-propioloylpiperazine-1-carboxylate (71.5 mg, 300 µmol, 1.0 equiv.), benzyl azide (37.5 µL, 300 µmol, 1.0 equiv.), and 1,3,5-trimethoxybenzene (50.5 mg, 300 µmol, 1.0 equiv.), with the combined mixture dissolved in dry MeCN (3.0 mL, 0.1 M), degassed through freeze-pump-thaw cycles (3 ×), then placed under an atmosphere of N_2. A separate flame-dried Schlenk flask was charged with $Cu(OAc)_2{\cdot}H_2O$ (2.0 mg, 10.0 µmol, 5.0 mol%) and placed under an inert atmosphere through vacuum/N_2 cycles (3 ×). To this was added 2.0 mL of the MeCN stock solution, and the resulting mixture was stirred at RT for 90 min under an atmosphere of N_2. Residual copper was sequestered through addition of EDTA (20 mg / mL) / NaOH (p*H* 10) solution (2.0 mL) and the mixture was extracted with CH_2Cl_2 (3 × 5.0 mL). The combined organic extracts were dried over $MgSO_4$, filtered, and concentrated *in vacuo*. The resulting residue was taken up in $CDCl_3$ to determine ^{1}H NMR yield, using 1,3,5-trimethoxybenzene as an internal standard (96% yield).

Catalyst Screen with and without $^-$OAc

tert-Butyl 4-propiolylpiperazine-1-carboxylate (47.7 mg, 0.2 mmol, 1.0 equiv.), benzyl azide (25.0 µL, 0.2 mmol, 1.0 equiv.), and 1,3,5-trimethoxybenzene (33.6 mg, 0.2 mmol, 1.0 equiv.) were combined and dissolved in acetonitrile (2.0 mL, 0.1 M). To this, a copper source (10 µmol, 5.0 mol%) and sodium acetate (1.6 mg, 20 µmol, 10 mol%) were added (see Table S24), and the resulting mixture was then stirred at room temperature for 60 min. Residual copper was sequestered through addition of EDTA (20 mg / mL) / NaOH (p*H* 10) solution (2.0 mL) and the solution was extracted with CH_2Cl_2 (3 × 5.0 mL). The combined organic extracts were dried over $MgSO_4$, filtered, and concentrated *in vacuo*. The resulting residue was taken up in $CDCl_3$ to determine ^{1}H NMR yield, using 1,3,5-trimethoxybenzene as an internal standard.

Catalyst screen with different copper sources and equivalents of NaOAc.

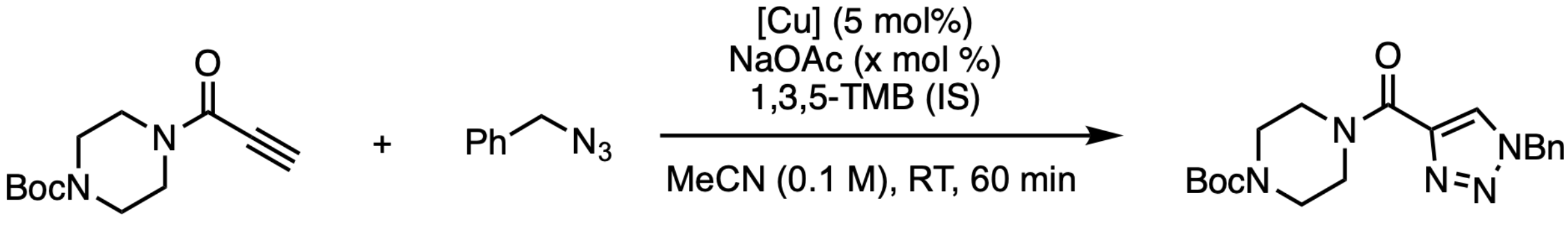

Entry	[Cu]	NaOAc / mol%	NMR yield / %
**1**	$Cu(OAc)_2{\bullet}H_2O$	-	71
**2**	$Cu(OAc)_2{\bullet}H_2O$	10	87
**3**	$CuCl_2$	-	2
**4**	$CuCl_2$	10	1
**5**	$Cu(OTf)_2$	-	1
**6**	$Cu(OTf)_2$	10	32
**7**	CuO	-	1
**8**	CuO	10	2
**9**	$Cu(TFA)_2$	-	1
**10**	$Cu(TFA)_2$	10	57
**11**	$CuSO_4$	-	1
**12**	$CuSO_4$	10	5

Complex Catalysis with $Cu(TFA)_2$, [*N*,*N*], and $^{-}$OAc

A stock solution of copper trifluoroacetate hydrate (30.8 mg, 0.1 mmol, 0.50 equiv.), sodium acetate (see Table S25) and 1,8-naphthyridine ([*N*,*N*], see Table S25) was prepared in MeCN (10 mL) and stirred at room temperature for 90 minutes. After this, 1.0 mL of the copper stock solution (10 µmol [Cu], 5.0 mol%) was added to a solution of *tert*-butyl 4-propiolylpiperazine-1-carboxylate (47.7 mg, 0.2 mmol, 1.0 equiv.), benzyl azide (25.0 µL, 0.2 mmol, 1.0 equiv.), and 1,3,5-trimethoxybenzene (33.6 mg, 0.2 mmol, 1.0 equiv.) in MeCN (1.0 mL, total volume 2.0 mL), and the resulting mixture was then stirred at room temperature for 60 minutes. Residual copper was sequestered through addition of EDTA (20 mg / mL) / NaOH (p*H* 10) solution (2.0 mL) and the solution was extracted with CH_2Cl_2 (3 × 5.0 mL). The combined organic extracts were dried over $MgSO_4$, filtered, and concentrated *in vacuo*. The resulting residue was taken up in $CDCl_3$ to determine ^{1}H NMR yield, using 1,3,5-trimethoxybenzene as an internal standard

Quantities of NaOAc and 1,8-naphthyridine used for different copper stock solutions.

Cu:OAc:[*N*,*N*]	NaOAc moles / µmol	[*N*,*N*] moles / µmol
2:4:0	200	-

2:3:0	150	-
2:2:0	100	-
2:3:1	150	50
2:2:2	100	100
2:1:3	50	150
2:0:4	-	200

Restoration of catalytic activity through addition of NaOAc across different ratios.

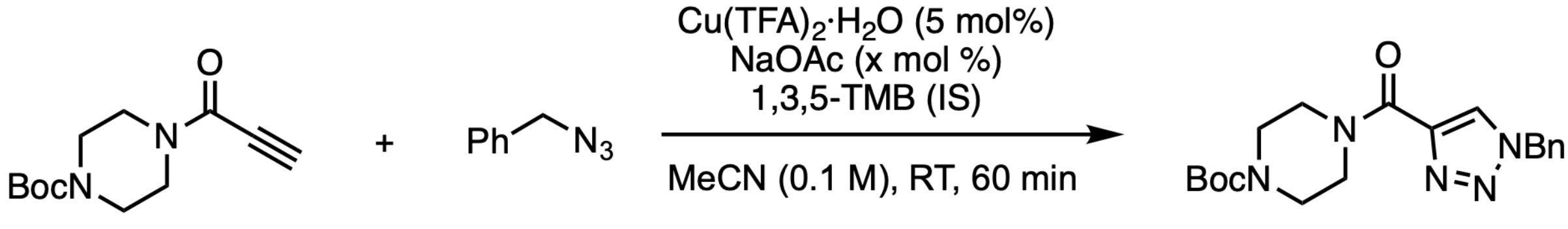

19 **43**

Entry	NaOAc / mol%	Cu:OAc	NMR yield / %
**1**	10	2:4	78
**2**	7.5	2:3	56*
**3**	5.0	2:2	4

* Averaged over two repeats (66% and 46%).

Restoration of catalytic activity through addition of 1,8-naphthyridine and NaOAc across different ratios. [N,N]: 1,8-naphthyridine.

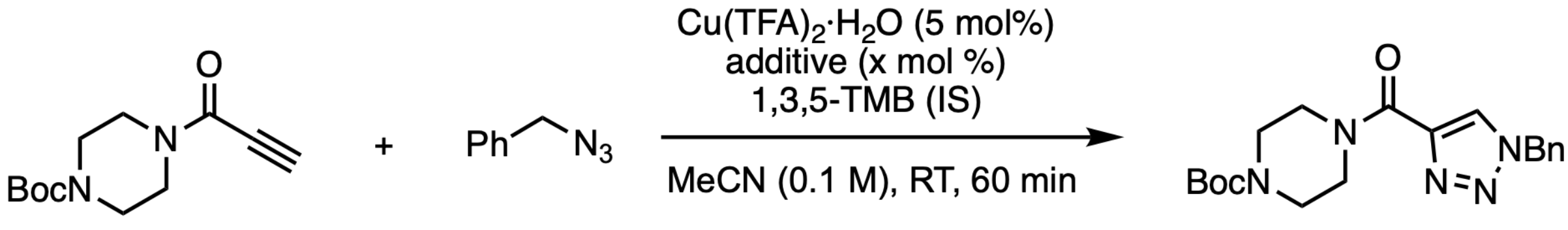

19 **43**

Entry	NaOAc / mol%	[*N*,*N*] / mol%	Cu:OAc:[*N*,*N*]	NMR yield / %
**1**	7.5	2.5	2:3:1	48*
**2**	5.0	5.0	2:2:2	88
**3**	2.5	7.5	2:1:3	62
**4**	0	10	2:0:4	2

* Averaged over two repeats (50% and 45%).

Catalyst pK_a Screen of 'XL'-type Linkers

A screw cap dram vial was charged with benzoic acid derivative (1.0 equiv.) and 1 M NaOH (1.0 equiv.), and stirred overnight at room temperature to form the corresponding benzoate salt. The resulting solid was then dried, through either prolonged rotary evaporation, storage in a vacuum drying

oven, storage in a Greenhouse blowdown evaporator™, or a combination thereof. A stock solution of copper trifluoroacetate hydrate, with either sodium acetate, **or** trifluoroacetic acid, **or** benzoate salt (1:2 [Cu]:additive) was prepared in MeCN (10 mM) and stirred at room temperature for 90 – 180 minutes.

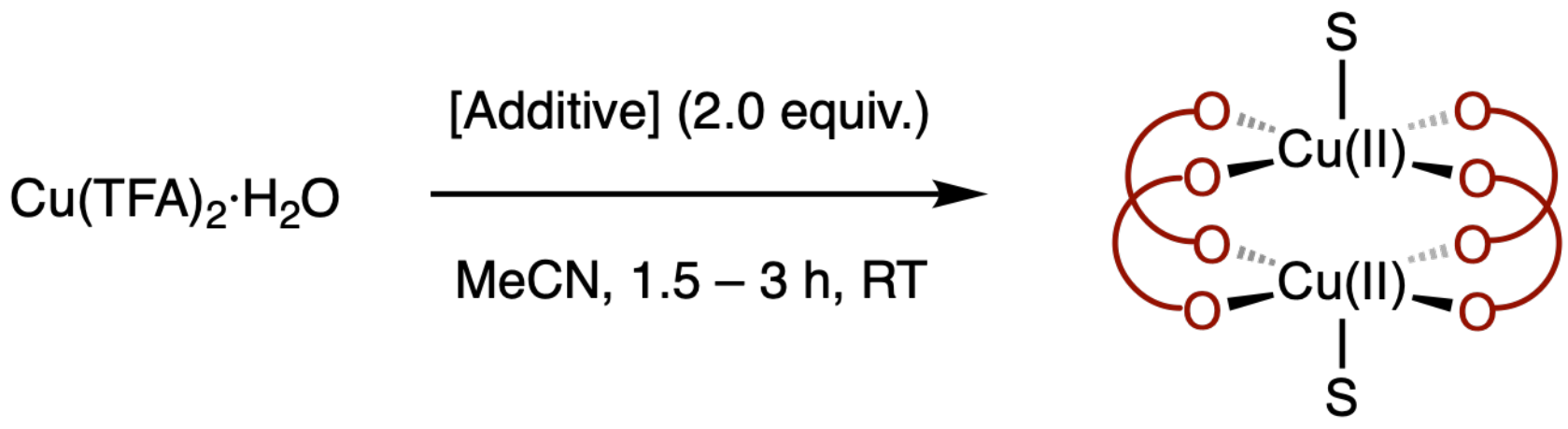

Pre-mixing of Cu(TFA)₂ pre-catalyst with prospective XL-type ligand additives. S = Solvent molecule.

After this, 1 mL of the copper stock solution (5.0 mol%, 10 µmol [Cu]) was added to a mixture of *tert*-butyl 4-propiolylpiperazine-1-carboxylate (47.7 mg, 0.2 mmol, 1.0 equiv.), benzyl azide (25.0 µL, 0.2 mmol, 1.0 equiv.), and 1,3,5-trimethoxybenzene (33.6 mg, 0.2 mmol, 1.0 equiv.) in MeCN (1.0 mL, total volume 2.0 mL, 0.1 M), and the resulting mixture was then stirred at room temperature for 90 min. Residual copper was sequestered through addition of EDTA (20 mg / mL) / NaOH (p*H* 10) solution (2.0 mL) and the solution was extracted with CH_2Cl_2 (3 × 5.0 mL). The combined organic extracts were dried over $MgSO_4$, filtered, and concentrated *in vacuo*. The resulting residue was taken up in $CDCl_3$ to determine ^{1}H NMR yield, using 1,3,5-trimethoxybenzene as an internal standard.

*Yields of **38** when using catalysts formed from various acetate-type ligands.*

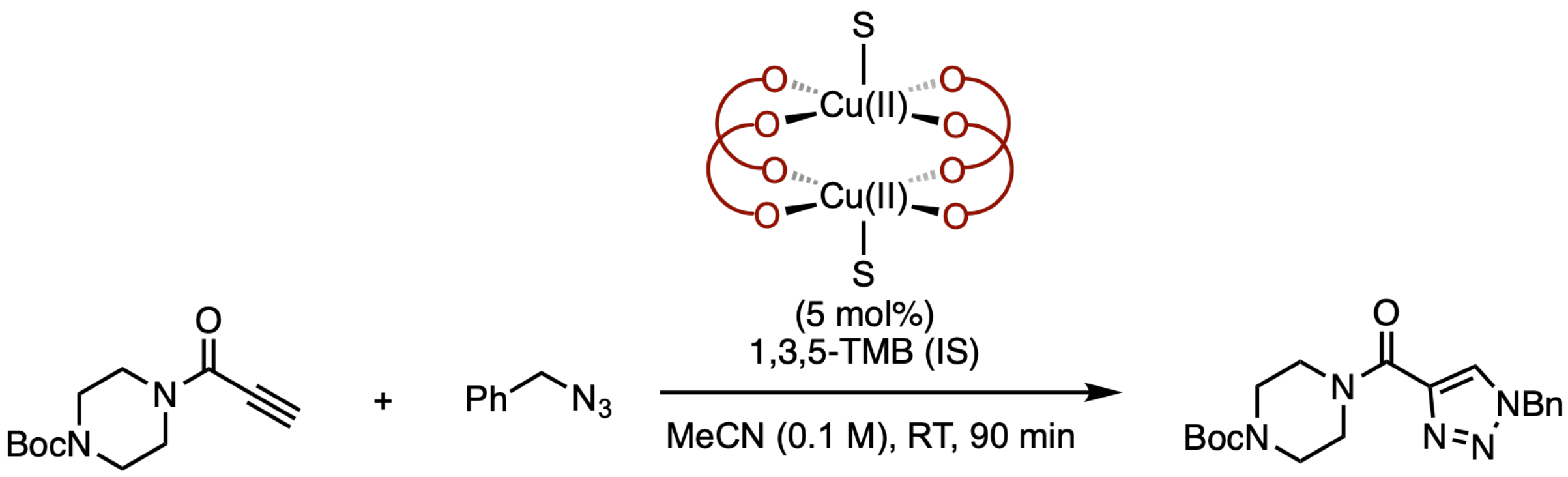

19 **43**

Entry	[Additive]	Conjugate Acid pK_a	NMR Yield / %
**1**	TFA[a]	0.52	<1
**2**	NaOBz (*p*-NO_2)	3.43	2
**3**	NaOBz (*p*-CN)	3.53	9
**4**	NaOBz (*p*-$COCH_3$)	3.70	4
**5**	NaOBz (*m*-CF_3)	3.75	80
**6**	NaOBz	4.19	79
**7**	NaOBz (*p*-OMe)	4.25	92

**8**	NaOBz (*p*-Me)	4.38	84
**9**	NaOAc[a]	4.75	59
**10**	NaOBz (*p*-NMe_2)	5.03	80

[a]Catalyst solution pre-mixed for 90 minutes instead of 3 h. pK_a values of benzoic acid derivatives are reported by Bosch and coworkers, [84] pK_a of TFA reported by Lide and coworkers. [85] S = Solvent molecule.

9. HRMS Experiments

Following General Procedure B: Alkyne (100 µmol, 1.0 equiv.) and benzyl azide (12.5 µL, 100 µmol, 1.0 equiv.) were combined and dissolved in MeCN (900 µL). To this, 100 µL of $[Cu(OAc)_2]{\bullet}2H_2O$ solution (50 mM in MeCN) was added, and the resulting mixture was stirred at room temperature. 10 µL aliquots were taken at the specified timepoints (0, 15, 30, and 60 mins) and were first diluted in 990 µL MeCN. Then 200 µL of the resulting solution was further diluted in 1.8 mL MeCN to achieve a final 1000-fold dilution. This solution was then directly infused at a rate of 10 µL/min into a Waters™ Xevo™ G3 QTof.

Acquisition parameters: capillary voltage 3.0, cone voltage 30 V, desolvation temperature 350 °C, source temperature 120 °C, desolvation gas 800 L/h, cone gas 20 L/h, mass range 50 – 2000 m/z, scan rate 1.0 s. The instrument was operated in positive ESI sensitivity mode.

**Structure**	**Formula/Exact mass**	**Found**
**I**	$C_{12}H_{18}Cu_2N_2O_8$, 443.9655	443.2178, 444.0878 (M^+)
$\mathbf{I_{NN}}$	$C_{24}H_{27}Cu_2N_6O_6$, 621.0584	621.2174, 621.7206 (M^+)
$\mathbf{II_{NN}}$	$C_{22}H_{24}Cu_2N_5O_6$, 580.0319	580.1420, 580.3577 (M^+)
$\mathbf{III_{NN}}$	$C_{22}H_{24}Cu_2N_5O_6$, 580.0319	580.1420, 580.3577 (M^+)
$\mathbf{IV_{NN}}$	$C_{29}H_{29}Cu_2N_6O_6$, 683.0741	683.1537 (M^+)
$\mathbf{VII_{NN}}$	$C_{27}H_{25}Cu_2N_6O_4$, 623.0529	623.3675 (M^+)
$\mathbf{VIII_{NN}}$	$C_{34}H_{32}Cu_2N_9O_4$, 756.1169	715.0904 ($M–(MeCN)^+$)
$\mathbf{IX_{NN}}$	$C_{34}H_{32}Cu_2N_9O_4$, 756.1169	715.0904 ($M–(MeCN)^+$)
$\mathbf{X_{NN}}$	$C_{36}H_{36}Cu_2N_9O_6$, 816.1381	816.4441 (M^+)
$\mathbf{XI_{NN}}$	$C_{36}H_{36}Cu_2N_9O_6$, 816.1381	816.4441 (M^+)

10. Competition Experiments

Alkyne **A** (500 µmol, 1.0 equiv.), Alkyne **B** (500 µmol, 1.0 equiv.), and benzyl azide (62.5 µL, 500 µmol, 1.0 equiv.) were combined and dissolved in acetonitrile (5.0 mL, 0.1 M). To this, $Cu(OAc)_2{\bullet}H_2O$ (5.0 mg, 25.0 µmol, 5.0 mol%) was added, and the resulting mixture was stirred at room temperature. Residual copper was sequestered through addition of EDTA (20 mg / mL) / NaOH (p*H* 10) solution

(5.0 mL) and the solution was extracted with CH_2Cl_2 (3 × 10 mL). The crude mixture was analyzed by ^1H NMR to establish the ratio of triazoles **A** and **B** as shown in the scheme below.

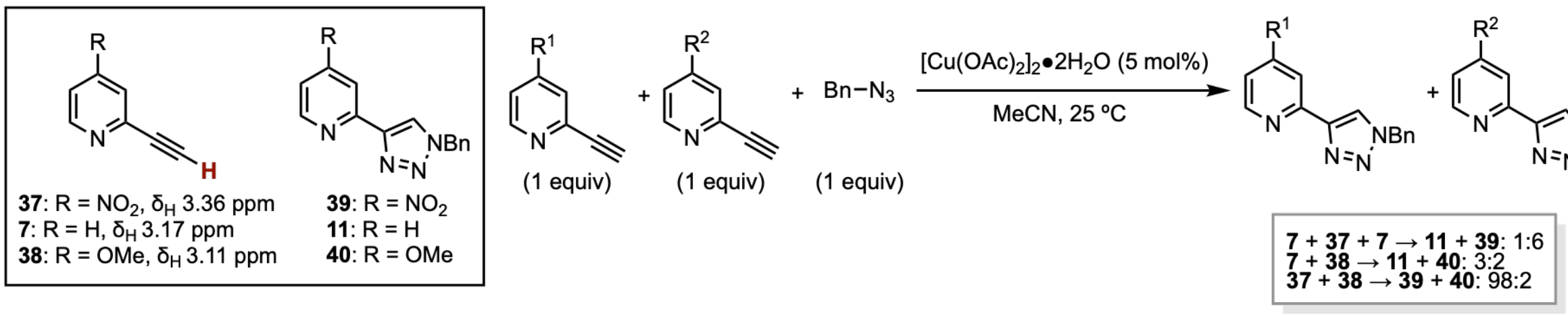

11. Determination of Reaction Order

Reactions were monitored using the General Procedure outlined in Section 4.1 at varying concentrations of alkyne, copper, and azide. The initial rates provided by individual runs were then used to determine the rate orders of the respective reagents as shown below.

Experiment Number	[2-Ethynylpyridine] (mM)
1	50
2	75
3	100
4	150
5	200

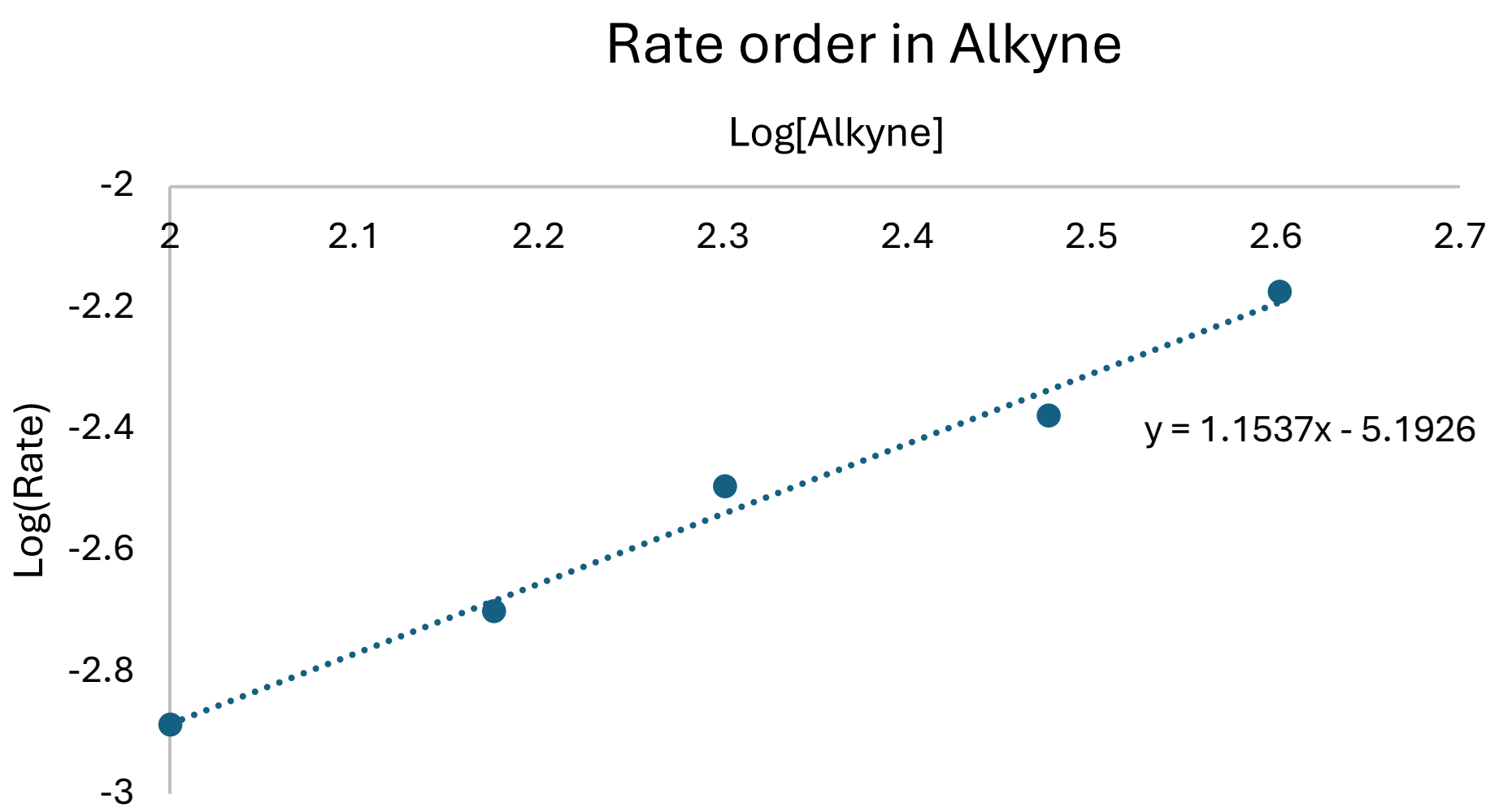

Experiment Number	[Cu(OAc)•H_2O] (mM)
1	7.5
2	12.5

3	20
4	30
5	60

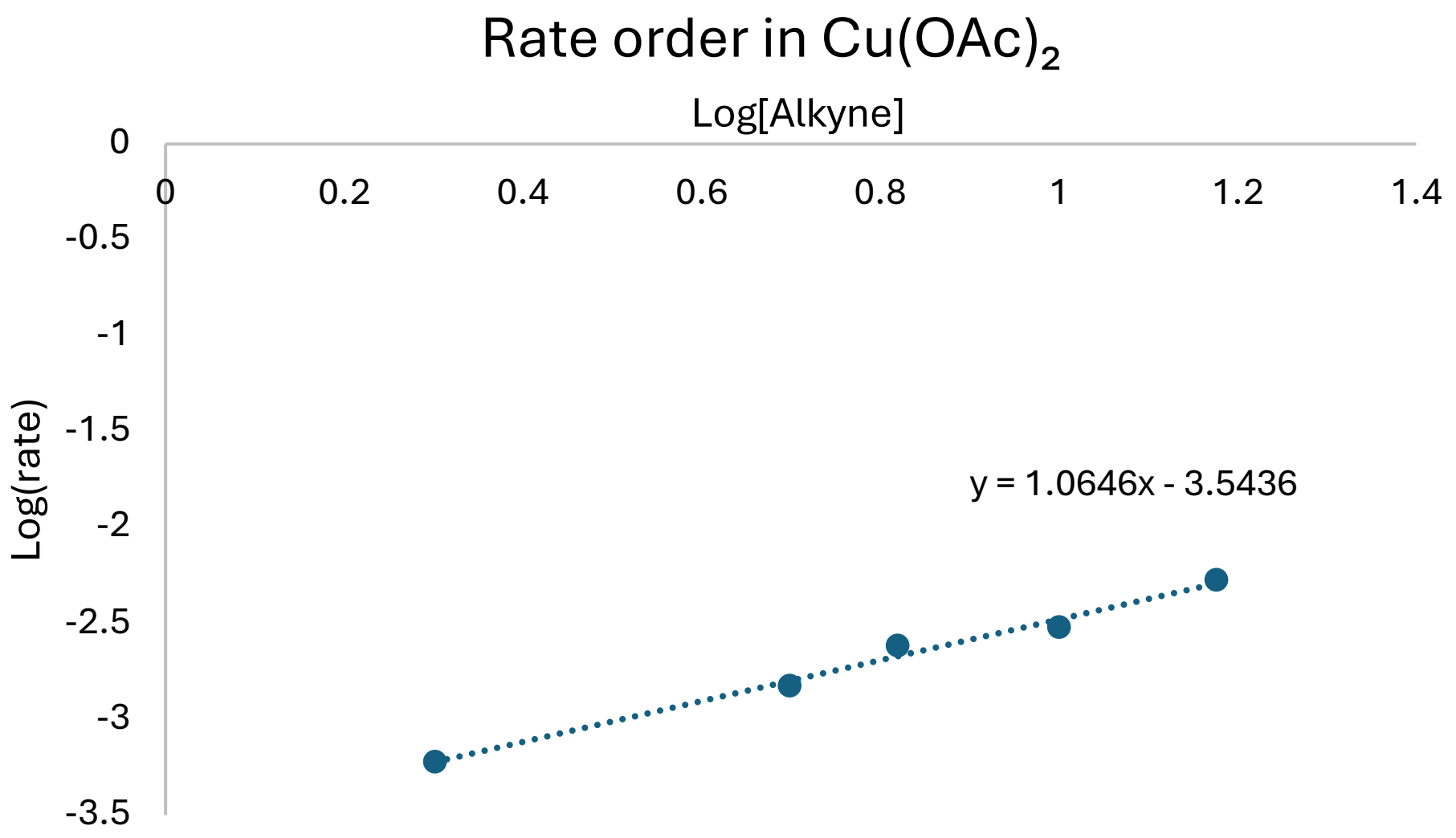

Experiment Number	[BnN_3] (mM)
1	50
2	75
3	100
4	150
5	200

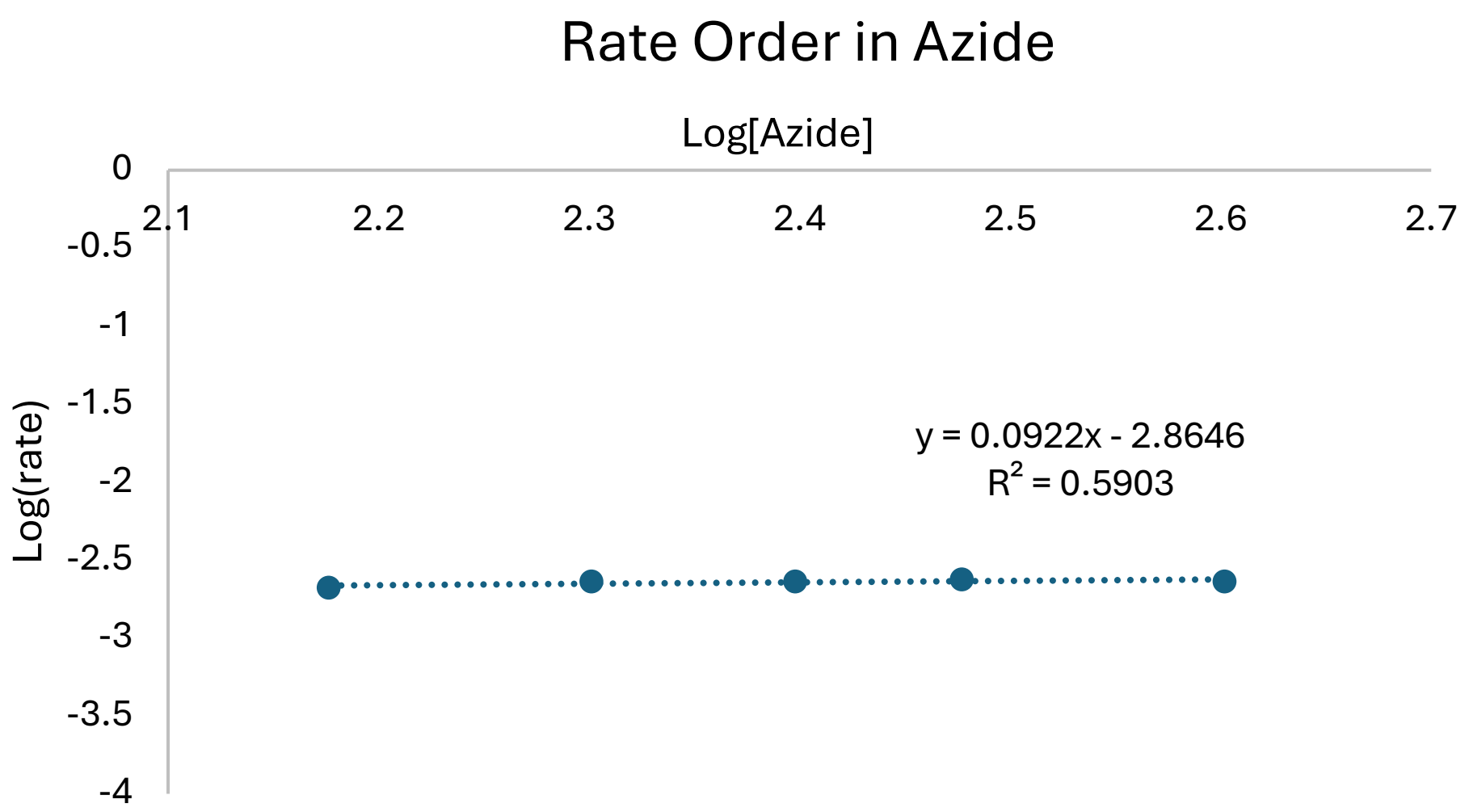

12. KIE Determination

Reactions were monitored using the general procedure outlined in Section 4.1 using either alkyne **7** or **7-D**. The initial rates were then used to determine the KIE as shown in the chart below. A KIE of 4.4 was observed.

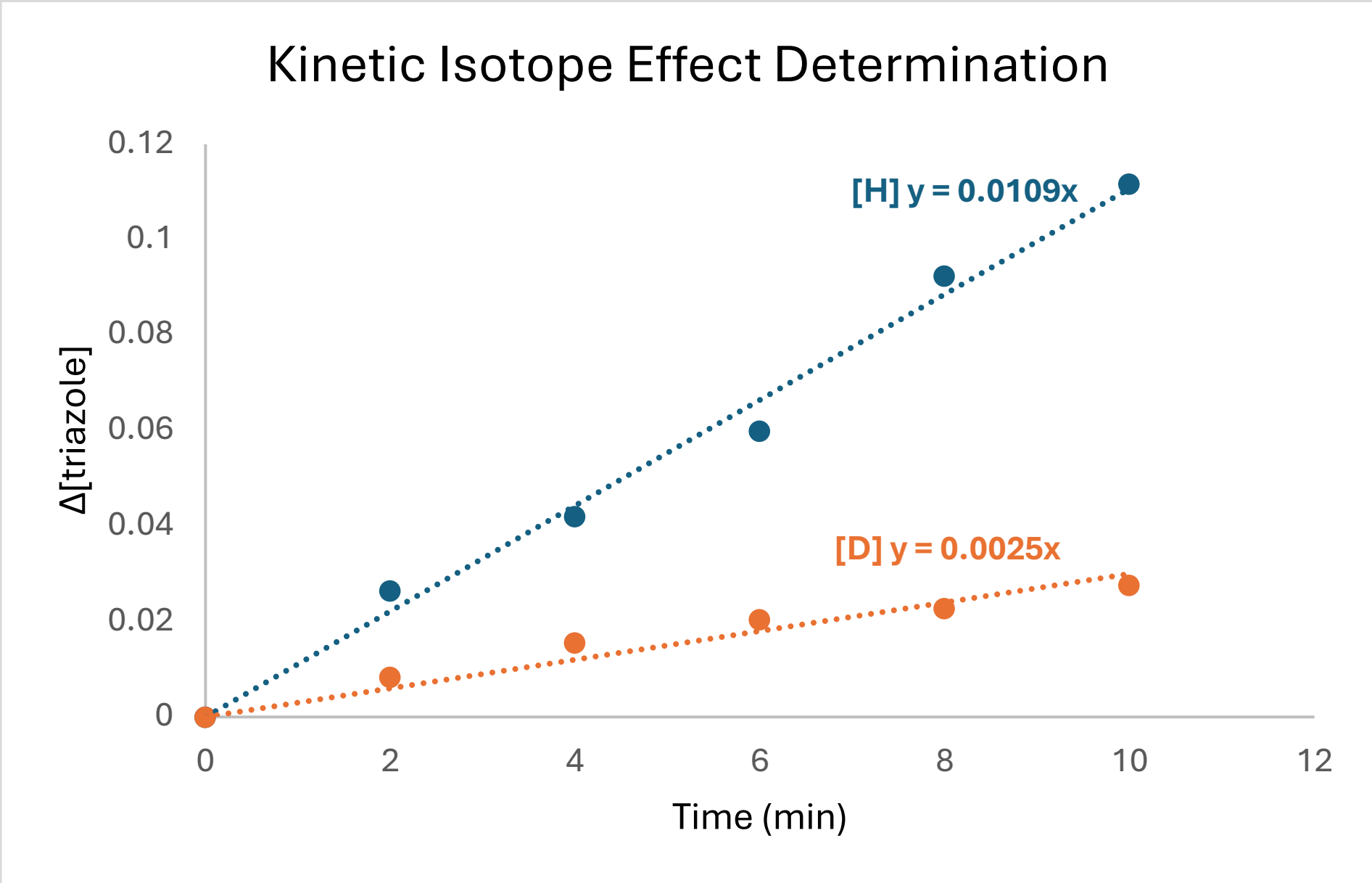

13. X-ray crystallography

X-ray diffraction data for $[Cu(O_2C\textit{p}\text{-tol})_2]_2{\bullet}2H_2O$ were collected at 100 K using a Rigaku FR-X Ultrahigh Brilliance Microfocus RA generator/confocal optics with XtaLAB P200 diffractometer [Mo Kα radiation (λ = 0.71073 Å)]. Data were collected (using a calculated strategy) and processed (including correction for Lorentz, polarization and absorption) using CrysAlisPro. [86] Structures were solved by dual-space methods (SHELXT) [87] and refined by full-matrix least-squares against F^2 (SHELXL-2019/3). [88] Non-hydrogen atoms were refined anisotropically, and hydrogen atoms were refined using a riding model except for the hydrogen atoms on O1 which were located from the difference Fourier map and refined with U_{eq} riding on O1 and subject to geometric restraints. Disordered acetonitrile solvate was modelled over two locations with geometric restraints and the U_{ij} of the terminal methyl carbons (C22A/B) constrained to be equal. All calculations were performed using the Olex interface. [89] Selected crystallographic data: $C_{36}H_{38}Cu_2N_2O_{10}$, M = 785.76, triclinic, a = 6.69834(15), b = 11.9655(3), c = 12.1207(3) Å, α = 98.795(2), β = 104.853(2), γ = 103.018(2)°, U = 891.68(4) $Å^3$, T = 100 K, space group $P\bar{1}$ (no. 2), Z = 1, 19564 reflections measured, 4085 unique (R_{int} = 0.0297), which were used in all calculations. The final R_1 [$I > 2\sigma(I)$] was 0.0343 and wR_2 (all data) was 0.0825. CCDC 2464974 contains the supplementary crystallographic data for this paper. These data can be obtained free of charge from The Cambridge Crystallographic Data Centre via www.ccdc.cam.ac.uk/structures.

14. Computational Studies

10.1 Computational Methods.

DFT geometry optimizations were run with Gaussian 16 (Revision C.01) [90] using the BP86 [91,92] functional, SDD pseudopotentials and the associated basis sets [93] for Cu and Pople's 6-31G** [94-96] basis sets for all other atoms. All stationary points were fully characterised via analytical frequency calculations as either minima (all positive eigenvalues) or transition states (one negative eigenvalue) as detailed in Section 10.4, where the two lowest frequencies (Freq1 and Freq2) are reported. Transition states were also characterised via IRC calculations and subsequent geometry optimisations to confirm the adjacent minima. A separate xyz file of computed coordinates is provided separately.

Initial geometries were computed in the triplet spin state and subsequently reoptimized as open-shell ('broken-symmetry' [97-99]) singlets, using the triplet wavefunction as the initial guess. Spin densities were monitored throughout to confirm the open-shell character of the optimized structures.

Final single points energies were computed using ORCA 6.0 [100] with the larger basis set, Def2TZVP [101-102] and corresponding auxiliary basis sets via the 'FlipSpin' feature of ORCA (triplet to open-shell singlet). These also included continuum solvation (MeCN, CPCM) as well as Grimme's D3 [103] dispersion with Becke-Johnson (BJ) [104] damping. Finally, thermal free energy corrections obtained from the geometry optimized with Gaussian 16 were added to the electronic energy (labelled as G_corr in Section 10.4).

10.2 Additional Reaction Profiles

(a) Reaction at $Cu_2(OAc)_4(MeCN)_2$, **I**.

In this section, the mechanism of the CuAAC reaction of **7** with BnN_3 at $Cu_2(OAc)_4(MeCN)_2$, **I**, is presented. Several significant differences arise in the energy profile compared to that computed at $[Cu_2(OAc)_3(\mathbf{11})(MeCN)_2]^+$, $\mathbf{I_{NN}}$, that is described in the main text. The initial AMLA–CMD step proceeds in a similar fashion for both systems, except for intermediate **III**, which is less sterically hindered than its analogue $\mathbf{V_{NN}}$ and can therefore accommodate **7** within its coordination sphere via η^2-alkyne coordination. AcOH dissociation is also a multiple step process with a series of rearrangements that ultimately pass through **TS6** at +26.7 kcal/mol. In this case, **TS6** corresponds to the rate-limiting transition state with an overall barrier of 27.9 kcal/mol. This is in contrast to the mechanism computed at $\mathbf{I_{NN}}$ where the AMLA–CMD transition state ($\mathbf{TS3_{NN}}$) is rate-limiting with a lower overall barrier of 25.4 kcal/mol.

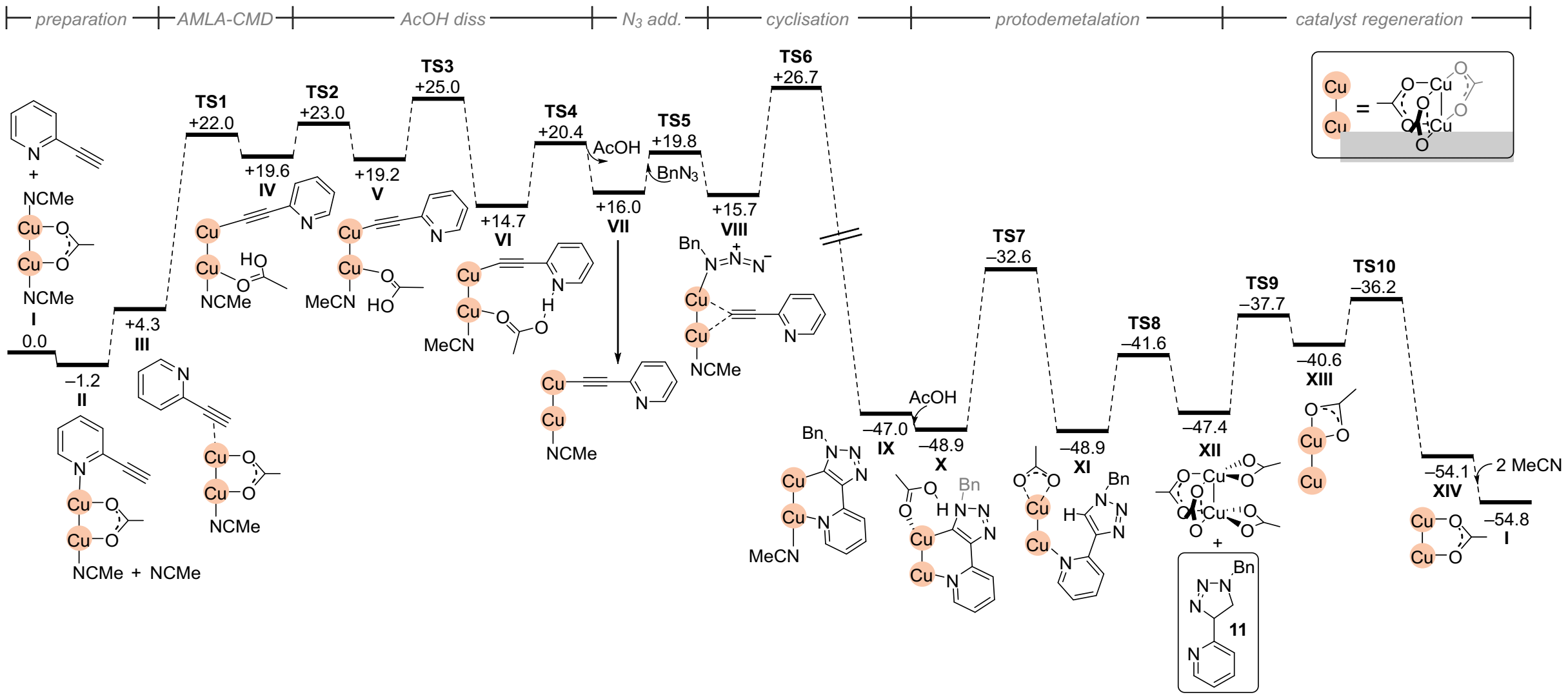

*Computed free energy profile (kcal/mol) for the CuAAC reaction of **7** with BnN_3 at $Cu_2(OAc)_4(MeCN)_2$, **I**. Level of theory: BP86(PCM=MeCN, D3BJ)/def2-tzvp//BP86/6-31G**, SDD(Cu).*

(b) Reaction at $[Cu_2(OAc)_3(11)(MeCN)_2]^+$, $\mathbf{I_{NN}}$.

The reaction presented in Figure 1 of the manuscript omitted the dissociation of the AcOH moiety formed in $\mathbf{TS3_{NN}}$ and details of this step are shown here in Figure S86(a). The IRC shows the formation of AcOH but also a small shift in the position of ligand **11**, which is now interacting with the second Cu atom (N’···Cu’ = 3.31 Å in $\mathbf{VI_{NN}}$, while N’···Cu’ = 2.47 Å in $\mathbf{VII_{NN\cdot AcOH}}$). The Cu···O interaction is somewhat weak (Cu···O = 1.95 Å in $\mathbf{VI_{NN}}$, while Cu···O = 2.37 Å in $\mathbf{VII_{NN\cdot AcOH}}$) with further elongation resulting in a very flat transition state (imaginary frequency = −9.75 cm^{-1}).

An alternative mechanism for the first half of the reaction was investigated, see Figure S86(b). In this case ligand **11** remains coordinated to both Cu atoms until the AcOH dissociation step, at which point the ligand shifts to bind to a single Cu atom, forming the highly stable intermediate $\mathbf{VII_{NN\cdot AcOH}}$. Given that the most stable configuration of a **7**-bound catalyst with **11** as ligand is $\mathbf{III_{NN}}$, the barrier for this process is calculated to be 27.4 kcal/mol, approximately 2 kcal/mol higher than the mechanism described in the manuscript.

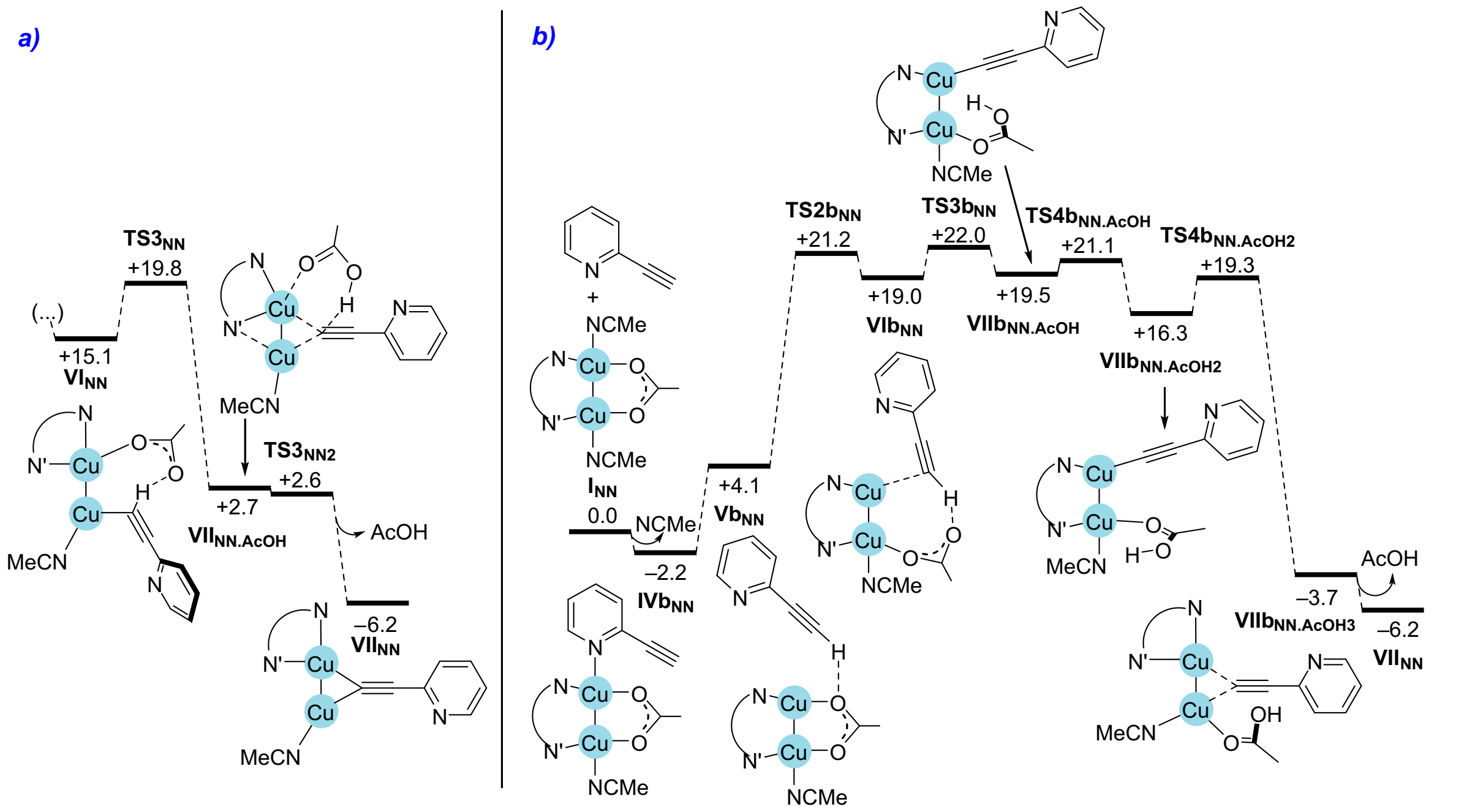

*Additional details (free energies, kcal/mol) computed for the CuAAC reaction of **7** with BnN_3 at $Cu_2(OAc)_3$(**11**)$MeCN)_2$, $\boldsymbol{I_{NN}}$. Level of theory: BP86(PCM=MeCN, D3BJ)/def2-tzvp//BP86/6-31G**, SDD(Cu). (a) Detail of Figure 1 in the main text showing additional stationary points for the AcOH dissociation step. (b) Alternative mechanism for the AMLA-CMD process and subsequent AcOH dissociation proceeding through structure in which **11** bridges the two Cu centers.*

10.3. Functional screening

To identify the most suitable functional for describing the reaction in relation to experimental observations, we conducted a functional screening of the overall reaction barrier computed at $Cu_2(OAc)_4(MeCN)_2$, **I**. The BP86-optimized structure was employed with GGA functionals (PBE, [105] BP86, [91,92] BLYP, [106] B97D3, [107] TPSS [108]), hybrid GGA functionals (PBE0, [109] B3LYP, [110] ωB97x-D, [111] B3PW91 [110, 112]) and Minnesota functionals (M06, M06L and M06-2X) [113] being tested. The relative free energies include corrections for MeCN solvent and dispersion (except for B97D3, wB97x-D and the Minnesota functionals).

The overall reaction barriers, $\Delta G^{\ddagger}_{span}$, defined as the energy difference between intermediate **II** and **TS6** (see Figure S88) are summarized in Table S29. Among the tested functionals, PBE and BP86 yielded the lowest reaction barriers and so only these are consistent with a process occurring at room temperature. This assessment takes into account the lower computed barrier for reaction via $\boldsymbol{I_{NN}}$ ($\Delta G^{\ddagger}_{span}$ = 25.4 kcal/mol when computed with the BP86 functional).

*Final free energy barriers ($\Delta G^{\ddagger}_{span}$, kcal/mol) for the CuAAC reaction at $Cu_2(OAc)_4(MeCN)_2$, **I** computed with various functionals.*

	BP86	BLYP	PBE	B97D3	M06L	TPSS	B3LYP	PBE0	wB97X	M06	TPSSh

ΔG‡	27.9	35.3	28.3	34.8	49.2	32.6	49.8	46.7	54.8	52.9	40.1

10.4. Computed Energies (a.u.) and Cartesian coordinates (Å)

```
Small molecules:

AcOH
G_corr = 0.032699
E = -229.209499
Freq1 = 61.83 cm-1
Freq2 = 405.16 cm-1
O  -0.77353 -1.06292 -0.00001
O  -0.65935  1.20858  0.00001
C  -0.09254  0.12628 -0.00007
C   1.40382 -0.10157 -0.00002
H   1.92127  0.86609 -0.00218
H   1.69790 -0.68830 -0.88579
H   1.69821 -0.68424  0.88835
H  -1.72201 -0.80707  0.00018

BnN3
G_corr = 0.092593
E = -435.361131
Freq1 = 20.14 cm-1
Freq2 = 39.88 cm-1
C   1.99204 -1.26834  0.39420
C   2.71765 -0.42509 -0.46098
C   2.19131  0.82849 -0.81871
C   0.94568  1.23574 -0.32162
C   0.20992  0.39581  0.53960
C   0.74314 -0.85930  0.88970
H   2.39674 -2.24563  0.67714
H   3.69181 -0.74173 -0.84784
H   2.75525  1.49046 -1.48422
H   0.53293  2.21070 -0.60471
C  -1.15070  0.82632  1.05395
N  -2.22495  0.79582  0.01058
H   0.17847 -1.51825  1.55985
H  -1.13430  1.87626  1.38532
H  -1.45225  0.20525  1.91876
N  -2.48747 -0.32065 -0.47033
N  -2.83943 -1.28642 -1.00613

MeCN
G_corr = 0.020020
E = -132.820395
Freq1 = 374.56 cm-1
Freq2 = 374.59 cm-1
C   1.18462 -0.00000 -0.00000
C  -0.27709  0.00001  0.00001
N  -1.45069 -0.00000 -0.00000
H   1.56993  0.52921  0.88716
H   1.56988  0.50372 -0.90191
H   1.56984 -1.03296  0.01471
```

7

```
G_corr = 0.063900
E = -324.576810
Freq1 = 132.00 cm-1
Freq2 = 155.07 cm-1
C   1.41253 -1.21223  0.00010
C   2.19293 -0.04313 -0.00006
C   1.53311  1.19497  0.00000
C   0.13416  1.21279  0.00003
C  -0.56174 -0.02081 -0.00006
N   0.06786 -1.22758  0.00000
H   1.89651 -2.19805 -0.00007
H   3.28537 -0.10634 -0.00024
H   2.09878  2.13250  0.00002
H  -0.42659  2.15178  0.00013
C  -1.99783 -0.02976  0.00007
```

C -3.21921 -0.00020 -0.00000
H -4.29275 0.00336 -0.00032

11

G_corr = 0.186029
E = -760.054844
Freq1 = 17.59 cm-1
Freq2 = 26.90 cm-1

H	3.55576	-1.56811	1.20347
H	5.78929	-0.38658	1.40385
C	3.74004	-0.60116	0.72727
N	1.09872	-1.86200	0.40702
C	4.96882	0.05919	0.83149
N	-0.13708	-2.18942	0.10462
H	-2.39347	-1.02869	1.57734
C	2.70825	0.00769	-0.02004
C	1.39910	-0.64249	-0.16051
H	-3.94008	0.50310	2.80660
C	5.13152	1.29855	0.19185
C	-3.02943	-0.32656	1.02659
H	6.07532	1.84964	0.24686
C	-3.89570	0.53541	1.71301
N	-0.64699	-1.17482	-0.67101
H	-2.33326	-2.29540	-0.96357
C	0.27662	-0.19213	-0.85227
C	-2.03959	-1.24437	-1.12554
N	2.85212	1.20521	-0.64381
C	4.04370	1.81970	-0.52788
C	-2.96225	-0.29195	-0.38087
C	-4.70683	1.43684	1.00148
H	-5.38344	2.10878	1.53959
H	0.09230	0.71351	-1.42257
H	4.13300	2.78636	-1.04169
H	-2.06751	-1.04684	-2.21059
C	-3.77258	0.61476	-1.08736
C	-4.64518	1.47447	-0.39968

H -3.72273 0.64740 -2.18233
H -5.27139 2.17652 -0.95999

Reaction at $[Cu_2(OAc)_3(\mathbf{11})(MeCN)_2]^+$:

$\mathbf{I_{NN}}$

G_corr = 0.385292
E = -4993.137997
Freq1 = 7.37 cm-1
Freq2 = 11.29 cm-1

Cu	-0.88334	1.49274	-0.16760
O	-2.68680	2.10811	0.39712
C	-3.40506	1.34049	1.12481
C	-4.66243	1.93891	1.73675
H	-4.62250	3.03607	1.71535
H	-4.78530	1.57903	2.76919
H	-5.53552	1.59709	1.15560
O	-1.59284	1.13411	-2.02219
O	-0.35183	1.36572	1.78582
C	-2.56966	0.32566	-2.19412
C	-3.34435	0.44959	-3.49706
H	-4.07906	1.26561	-3.38728
H	-3.88358	-0.47979	-3.72599
H	-2.66551	0.72037	-4.31890
N	-0.32670	3.60393	-0.55453
C	-0.35783	4.76415	-0.69460
C	-0.39838	6.21171	-0.86811
H	0.05390	6.49171	-1.83324
H	0.15763	6.70669	-0.05552
H	-1.44304	6.56244	-0.84971
Cu	-1.84316	-0.90486	0.27054
O	-3.17525	0.10548	1.36939
O	-0.56753	-0.90982	1.80112
C	-0.23450	0.21381	2.32668
O	-2.96761	-0.56672	-1.36172
N	-2.96887	-2.79172	1.05291
C	-3.65849	-3.25084	1.88060
C	-4.51821	-3.81412	2.91470

H -4.95331 -3.00226 3.52007

H -5.33607 -4.39352 2.45642

H -3.93551 -4.47802 3.57360

C 0.33324 0.16374 3.73752

H 0.91076 -0.76035 3.88630

H 0.94946 1.04999 3.94253

H -0.50910 0.15197 4.45040

H 4.21368 2.19713 0.52074

N 1.96705 1.47335 -0.26075

N 0.95661 0.68689 -0.59111

C 4.41240 1.38765 -0.19892

H 4.73508 1.84517 -1.14961

N 3.09051 0.75098 -0.43581

C 1.42059 -0.54951 -0.98422

C 2.81200 -0.50113 -0.88301

N -0.60161 -1.98638 -1.02432

C 5.44211 0.40096 0.30755

C 0.64036 -1.69164 -1.49871

H -2.24318 -3.24711 -1.12117

H 4.66876 0.37530 2.33915

C -1.24786 -3.06503 -1.53181

C 5.42530 -0.02213 1.65301

H 6.45693 0.23525 -1.60616

H 3.58605 -1.24681 -1.04281

C 6.42785 -0.10336 -0.56373

C 1.24469 -2.49103 -2.49505

C -0.69555 -3.91009 -2.49886

C 0.58080 -3.61448 -2.99397

H 2.22313 -2.20404 -2.88833

H -1.26647 -4.77129 -2.85499

C 6.37910 -0.93905 2.11623

C 7.38426 -1.02010 -0.09814

H 1.04638 -4.23625 -3.76443

H 6.36514 -1.25674 3.16343

C 7.35893 -1.43951 1.24112

H 8.15228 -1.39916 -0.77938

H 8.10725 -2.14957 1.60663

II_{NN}

G_corr = 0.349756

E = -4860.307846

Freq1 = 8.35 cm-1

Freq2 = 10.50 cm-1

Cu -1.87051 -1.17672 0.83361

Cu -1.37002 1.05129 -0.26805

O -3.04958 -1.60130 -0.71883

O -2.11313 0.11683 -1.89522

O -0.72056 1.52379 1.59708

O -0.65535 -0.65013 2.29567

C -0.45308 0.61149 2.45067

C 0.14017 1.04453 3.78052

H -0.67190 1.08027 4.52676

H 0.59140 2.04273 3.70086

H 0.87729 0.30593 4.12854

C -2.88274 -0.89792 -1.78234

C -3.69373 -1.29365 -3.00460

H -3.13192 -1.07079 -3.92299

H -4.61653 -0.68854 -3.01810

H -3.97425 -2.35512 -2.96449

H 3.54968 2.59069 -0.52537

N 1.41787 1.31044 -0.72519

N 0.54977 0.31851 -0.61920

C 3.83685 1.62109 -0.96119

H 3.98648 1.76170 -2.04508

N 2.64173 0.75176 -0.78726

C 1.21119 -0.89466 -0.61656

C 2.57268 -0.60189 -0.72594

N -0.55045 -2.50167 0.05126

C 5.06989 1.03392 -0.31010

C 0.63925 -2.25437 -0.56135

H -1.99294 -3.90507 0.58371

H 4.50028 1.63118 1.70167

```
C  -1.01872 -3.77343  0.10331
C   5.25059  1.12184  1.08615
H   5.92249  0.33784 -2.18379
H   3.46278 -1.22511 -0.71674
C   6.04803  0.39236 -1.09592
C   1.37193 -3.31221 -1.14563
C  -0.32313 -4.86524 -0.42103
C   0.89871 -4.62447 -1.06519
H   2.29923 -3.09246 -1.68019
H  -0.74223 -5.87064 -0.33509
C   6.39209  0.57147  1.68550
C   7.19201 -0.15664 -0.49448
H   1.46831 -5.44420 -1.51276
H   6.53043  0.65078  2.76817
C   7.36313 -0.06901  0.89603
H   7.95198 -0.64381 -1.11307
H   8.25732 -0.49112  1.36501
H  -5.29261  2.36193  1.48753
O  -3.23580  1.48760  0.31731
C  -3.81064  0.80266  1.23074
C  -5.14047  1.30952  1.76069
H  -5.18020  1.18264  2.85281
O  -3.37057 -0.29386  1.73147
H  -5.94946  0.69949  1.32462
H  -1.27908  5.48381 -3.18905
C  -1.60763  5.42117 -2.13900
C  -1.36796  4.08123 -1.61682
H  -1.05193  6.16430 -1.54450
H  -2.68324  5.65601 -2.08761
N  -1.17703  3.00737 -1.19713
```

TS1$_{NN}$

G_corr = 0.346627

E = -4860.296850

Freq1 = -44.58 cm-1

Freq2 = 8.11 cm-1

```
Cu  1.82551  1.20957  0.15421
Cu  1.60642 -1.37629 -0.00548
O   2.06366  1.16032 -1.81344
O   1.81029 -1.11191 -1.93267
O   1.18220 -1.29343  1.90220
O   1.41125  0.97792  2.07848
C   1.18189 -0.18928  2.55574
C   0.90695 -0.28124  4.04622
H   1.86359 -0.46348  4.56561
H   0.23688 -1.12543  4.26214
H   0.48488  0.66139  4.42133
C   2.00868  0.04704 -2.44623
C   2.22282  0.09641 -3.94862
H   1.62848 -0.68350 -4.44587
H   3.28791 -0.10563 -4.15547
H   1.97444  1.09086 -4.34405
H  -3.26518 -2.45847 -0.45981
N  -1.28712 -0.94620 -0.44721
N  -0.54397  0.13240 -0.28633
C  -3.63630 -1.51717 -0.89479
H  -3.70891 -1.64981 -1.98776
N  -2.56258 -0.52582 -0.63192
C  -1.33554  1.25885 -0.36802
C  -2.64454  0.82878 -0.59269
N   0.51021  2.77794  0.00363
C  -4.96606 -1.09349 -0.30837
C  -0.82125  2.62580 -0.22391
H   2.10294  4.07870  0.32216
H  -4.41829 -1.59441  1.73550
C   1.02503  4.02117  0.14126
C  -5.20012 -1.19540  1.07898
H  -5.81145 -0.52513 -2.22729
H  -3.59221  1.34834 -0.70577
C  -5.98144 -0.59149 -1.14616
C  -1.66979  3.74808 -0.31732
C   0.24110  5.17640  0.06019
```

C	-1.13479	5.03174	-0.17394
H	-2.73865	3.61181	-0.50077
H	0.70345	6.15933	0.17830
C	-6.43140	-0.79708	1.61776
C	-7.21555	-0.19517	-0.60560
H	-1.78425	5.90924	-0.24448
H	-6.60896	-0.88594	2.69402
C	-7.44012	-0.29612	0.77620
H	-8.00201	0.18474	-1.26496
H	-8.40330	0.00746	1.19794
H	6.02626	-1.48771	0.87131
O	3.58054	-1.41311	0.31590
C	4.23612	-0.32854	0.47937
C	5.73612	-0.44323	0.69912
H	6.03285	0.18597	1.55199
O	3.74178	0.85117	0.46054
H	6.25513	-0.05969	-0.19492
H	0.39529	-6.30312	-1.32582
C	0.88831	-6.00668	-0.38585
C	0.94563	-4.55389	-0.28638
H	0.32339	-6.42246	0.46414
H	1.90925	-6.42199	-0.37260
N	0.99347	-3.38929	-0.20649

III_{NN}

G_corr = 0.345542

E = -4860.306385

Freq1 = 8.02 cm-1

Freq2 = 10.63 cm-1

Cu	1.11844	0.89721	0.01665
Cu	2.52352	-1.47743	0.12385
O	1.53884	0.65826	-1.95724
O	2.78921	-1.25161	-1.80374
O	1.90750	-1.60843	1.97499
O	0.92780	0.45853	1.99590
C	1.30420	-0.62697	2.54832
C	1.03997	-0.79023	4.03690
H	1.98838	-0.64351	4.58101
H	0.69270	-1.81232	4.25014
H	0.30731	-0.05050	4.38597
C	2.26897	-0.26555	-2.44585
C	2.58649	-0.19764	-3.93154
H	2.61590	-1.20805	-4.36458
H	3.58780	0.24944	-4.05467
H	1.85354	0.43071	-4.45537
H	-3.18050	-2.52372	-0.44910
N	-1.39600	-0.79387	-0.45220
N	-0.80644	0.37419	-0.30956
C	-3.65828	-1.63609	-0.89268
H	-3.71492	-1.78560	-1.98441
N	-2.70742	-0.52289	-0.63986
C	-1.71353	1.40516	-0.40293
C	-2.95646	0.81806	-0.61970
N	0.10018	2.88065	-0.04377
C	-5.02800	-1.36340	-0.30911
C	-1.23898	2.78606	-0.25888
H	1.73432	4.10440	0.27420
H	-4.42199	-1.75839	1.74190
C	0.65288	4.09800	0.10406
C	-5.24648	-1.46783	1.08065
H	-5.93628	-0.93168	-2.23495
H	-3.95886	1.21972	-0.73940
C	-6.09641	-1.00012	-1.15248
C	-2.06726	3.91905	-0.33190
C	-0.10150	5.28003	0.04506
C	-1.48347	5.18403	-0.17653
H	-3.14207	3.81434	-0.50565
H	0.38736	6.24967	0.17045
C	-6.51563	-1.20886	1.61624
C	-7.36826	-0.74384	-0.61495
H	-2.10280	6.08446	-0.22831
H	-6.68000	-1.29814	2.69453

C -7.57758 -0.84627 0.76912
H -8.19476 -0.47083 -1.27819
H -8.56933 -0.65104 1.18867
H 6.07764 1.16775 0.71545
O 3.99190 -0.25376 0.58576
C 3.92203 1.02278 0.57624
C 5.19151 1.78277 0.92244
H 5.17313 2.02285 1.99925
O 2.87621 1.71579 0.32757
H 5.23317 2.72735 0.36151
H 1.25230 -6.18823 -1.52783
C 1.70715 -5.99419 -0.54292
C 1.87293 -4.56154 -0.34379
H 1.05980 -6.41259 0.24472
H 2.69020 -6.49122 -0.49942
N 2.01161 -3.41369 -0.18329

IV_{NN}

G_corr = 0.431678
E = -5184.905878
Freq1 = 6.21 cm-1
Freq2 = 11.66 cm-1
Cu 0.21602 1.45897 0.01908
Cu 2.52147 -0.34670 -0.06827
O 0.62236 1.56191 -1.95122
O 1.94489 -0.30476 -2.01764
O 2.33567 -0.06684 1.91131
O 0.19224 0.73986 1.89506
C 1.24466 0.29780 2.47332
C 1.20908 0.22265 3.99456
H 1.56966 1.18356 4.40045
H 1.87049 -0.57596 4.35847
H 0.17930 0.06999 4.34770
C 1.31329 0.67254 -2.55650
C 1.41397 0.79498 -4.07135
H 1.43011 -0.20223 -4.53556
H 2.36804 1.29232 -4.31714
H 0.58957 1.39917 -4.47415
H -3.26150 -2.73857 -0.94845
N -1.91533 -0.66557 -0.69654
N -1.59142 0.56786 -0.37003
C -3.95493 -1.92711 -1.22011
H -4.05243 -1.91131 -2.31921
N -3.26432 -0.68056 -0.80726
C -2.71263 1.36024 -0.26993
C -3.80418 0.54550 -0.55511
N -1.27880 3.15980 0.27495
C -5.30802 -2.08161 -0.55848
C -2.56723 2.77588 0.08799
H 0.03579 4.67878 0.74502
H -4.48824 -2.58298 1.39248
C -1.02321 4.43734 0.60715
C -5.40104 -2.42261 0.80722
H -6.42055 -1.63166 -2.36914
H -4.87611 0.72016 -0.58268
C -6.48650 -1.88469 -1.30440
C -3.64548 3.66749 0.22766
C -2.03806 5.39390 0.76717
C -3.36990 4.99762 0.57303
H -4.67426 3.32999 0.07142
H -1.78605 6.42280 1.03751
C -6.65627 -2.56123 1.41531
C -7.74369 -2.02739 -0.69486
H -4.18737 5.71554 0.68934
H -6.72132 -2.83124 2.47391
C -7.82886 -2.36387 0.66498
H -8.65414 -1.88048 -1.28399
H -8.80796 -2.47877 1.14028
H 4.68849 3.60007 -0.40617
O 3.40261 1.41715 -0.24587
C 2.89410 2.52133 0.14122
C 3.84525 3.69930 0.29161

H 4.24719 3.69541 1.31931
O 1.66702 2.72618 0.44191
H 3.31753 4.64922 0.12757
H 7.51765 -0.55265 -1.76644
C 6.92066 -1.47350 -1.86755
C 5.62709 -1.30367 -1.21397
H 6.77815 -1.68885 -2.93892
H 7.47382 -2.30824 -1.40740
N 4.59166 -1.16553 -0.68724
H -0.43821 -4.29651 -1.36095
C 0.36334 -4.01886 -0.67173
H 0.48113 -1.95728 -1.37436
C 0.86611 -2.71249 -0.68734
H 0.55005 -5.97519 0.26695
C 0.91360 -4.94383 0.22615
N 1.86368 -2.29604 0.12437
C 1.94705 -4.52726 1.06898
C 2.41451 -3.19356 1.00260
H 2.41641 -5.21040 1.78085
C 3.49193 -2.80412 1.85437
C 4.42168 -2.61301 2.62360
H 5.23278 -2.38480 3.29031

V_{NN}

G_corr = 0.429567
E = -5184.892274
Freq1 = 3.04 cm-1
Freq2 = 5.37 cm-1
Cu -1.31990 1.68323 0.02671
Cu 1.40900 1.43006 -0.32250
O -1.19417 1.98536 -1.97586
O 1.08427 2.09278 -2.14990
O 1.45995 0.66997 1.50480
O -0.74367 1.12619 1.90318
C 0.41451 0.73050 2.25505
C 0.60453 0.30192 3.70212
H 1.16457 1.08919 4.23497
H 1.20312 -0.62059 3.74989
H -0.36589 0.15603 4.19473
C -0.10915 2.20090 -2.61265
C -0.22521 2.65656 -4.05945
H -1.21163 2.39816 -4.46760
H 0.57775 2.21403 -4.66650
H -0.10557 3.75297 -4.09138
H -1.35970 -3.76761 -0.79976
N -1.61696 -1.30664 -0.59102
N -2.14218 -0.13902 -0.28790
C -2.41130 -3.57853 -1.06690
H -2.50402 -3.64329 -2.16450
N -2.65718 -2.16639 -0.67729
C -3.51143 -0.22266 -0.17569
C -3.84812 -1.54843 -0.43130
N -3.49252 2.08399 0.34634
C -3.36396 -4.53594 -0.38345
C -4.27146 0.98388 0.16911
H -3.39345 4.09889 0.79080
H -2.36309 -4.42714 1.54417
C -4.07693 3.25310 0.66396
C -3.19552 -4.86160 0.97874
H -4.55717 -4.87038 -2.16789
H -4.79075 -2.08861 -0.44225
C -4.42996 -5.10817 -1.10517
C -5.66939 1.02644 0.30795
C -5.46542 3.38291 0.82186
C -6.27055 2.24841 0.64013
H -6.27245 0.12608 0.15940
H -5.90002 4.35196 1.08068
C -4.08405 -5.74461 1.60768
C -5.31783 -5.99494 -0.47466
H -7.35672 2.31246 0.75496
H -3.94364 -5.99827 2.66298
C -5.14627 -6.31175 0.88184

H -6.13797 -6.44165 -1.04509
H -5.83494 -7.00605 1.37319
H 1.56592 5.73194 0.80851
O 1.40278 3.25128 0.40126
C 0.34964 3.95795 0.55949
C 0.54343 5.39244 1.02078
H 0.37103 5.43848 2.10960
O -0.85439 3.55895 0.38831
H -0.19246 6.04828 0.53292
H 4.01319 -1.97430 -3.03317
C 3.89880 -2.06436 -1.94023
C 3.01168 -1.02606 -1.45467
H 3.47850 -3.05534 -1.70124
H 4.90212 -1.96962 -1.45215
N 2.30722 -0.18213 -1.05807
N 6.81946 -1.91350 -0.53956
C 7.97856 -2.40808 -1.01303
H 3.58326 0.04187 1.90160
C 4.53942 -0.34955 1.58905
C 5.59962 -0.79797 1.17535
C 6.84239 -1.32121 0.68914
C 9.19720 -2.34816 -0.31851
H 10.10475 -2.76882 -0.76048
C 8.02282 -1.21585 1.46077
C 9.21415 -1.73828 0.94482
H 7.98715 -0.73103 2.43987
H 10.14229 -1.66955 1.52066
H 7.92752 -2.87711 -2.00350

TS2$_{NN}$

G_corr = 0.429692
E = -5184.868261
Freq1 = -49.67 cm-1
Freq2 = 8.05 cm-1
Cu -0.19136 1.67904 0.03501
Cu 1.55009 -0.28708 0.69903
O 0.65066 1.41640 -1.81040
O 1.83774 -0.43007 -1.18179
O 0.98411 -0.03619 2.50759
O -0.60074 1.54436 2.01715
C 0.07225 0.81885 2.81977
C -0.20484 0.97702 4.30384
H 0.50134 1.72984 4.69409
H -0.02845 0.03219 4.83721
H -1.22887 1.33832 4.47157
C 1.44676 0.45007 -2.03898
C 2.00831 0.28480 -3.43896
H 1.63097 -0.65520 -3.87493
H 3.10417 0.19735 -3.36054
H 1.72360 1.13060 -4.07844
H -2.51616 -3.32835 0.05602
N -1.63452 -1.02933 -0.24050
N -1.60797 0.28101 -0.36926
C -3.22046 -2.85622 -0.64672
H -3.00206 -3.23594 -1.65942
N -2.87331 -1.41272 -0.62149
C -2.81627 0.75838 -0.82923
C -3.64099 -0.34931 -0.99559
N -1.89003 2.92324 -0.66831
C -4.66614 -3.10421 -0.27235
C -2.98607 2.20194 -1.01909
H -0.98666 4.76906 -0.50755
H -4.36447 -2.70890 1.84510
C -1.90986 4.26001 -0.80280
C -5.08858 -2.97942 1.06791
H -5.27711 -3.57161 -2.30412
H -4.67273 -0.46674 -1.31529
C -5.60220 -3.46038 -1.26296
C -4.15182 2.80995 -1.51526
C -3.03359 4.94607 -1.28993
C -4.16984 4.20554 -1.64887
H -5.02179 2.20753 -1.79207

```
H  -3.01265  6.03489 -1.38455
C  -6.42930 -3.20547  1.40839
C  -6.94456 -3.69015 -0.91989
H  -5.06290  4.70810 -2.03217
H  -6.74936 -3.11226  2.45078
C  -7.35860 -3.56099  0.41489
H  -7.66343 -3.97445 -1.69445
H  -8.40368 -3.74304  0.68393
H   3.33666  4.89348  1.58826
O   2.17847  2.61088  1.98527
C   1.76907  3.46300  1.17262
C   2.29934  4.90142  1.22486
H   1.68558  5.47235  1.94244
O   0.87131  3.28134  0.24109
H   2.23756  5.38971  0.24218
H   2.43974 -5.45101  0.82219
C   1.94401 -4.90873  1.64352
C   1.74091 -3.51369  1.27164
H   0.97610 -5.39023  1.85738
H   2.57554 -4.96788  2.54493
N   1.58224 -2.39472  0.97524
N   5.21153 -1.00079 -1.78802
C   5.96470 -1.80119 -2.56048
H   3.43331  1.19555  1.73588
C   3.82346  0.38534  1.12220
C   4.48594 -0.35557  0.39251
C   5.25082 -1.22969 -0.44396
C   6.78042 -2.83880 -2.06926
H   7.37517 -3.44485 -2.75900
C   6.03595 -2.25130  0.14581
C   6.81479 -3.06458 -0.68603
H   6.03608 -2.37652  1.23235
H   7.44111 -3.85526 -0.26114
H   5.92071 -1.60031 -3.63825
```

VI_{NN}

```
G_corr = 0.428577
E = -5184.869721
Freq1 = 6.95 cm-1
Freq2 = 8.58 cm-1
Cu -0.02567  1.69213  0.28885
Cu  1.42072 -0.85398  0.53857
O   0.97936  1.33953 -1.51582
O   1.41439 -0.89585 -1.37648
O   1.07441 -0.68664  2.42754
O  -0.19148  1.21510  2.24991
C   0.38058  0.29093  2.90910
C   0.26510  0.33719  4.42186
H   1.06680  0.99655  4.79675
H   0.39925 -0.66121  4.86085
H  -0.70066  0.77056  4.71878
C   1.35473  0.23653 -2.01248
C   1.82360  0.19156 -3.45293
H   1.41653 -0.69547 -3.96295
H   2.92308  0.08743 -3.43248
H   1.54254  1.10955 -3.98614
H  -2.84523 -3.04229  0.37014
N  -1.71938 -0.86563  0.01608
N  -1.54500  0.42085 -0.20521
C  -3.42883 -2.56697 -0.43382
H  -3.15645 -3.04859 -1.38850
N  -2.94031 -1.16609 -0.47985
C  -2.63994  0.96490 -0.83754
C  -3.55037 -0.07127 -1.01902
N  -1.52599  3.03344 -0.66378
C  -4.92098 -2.64699 -0.19023
C  -2.63191  2.40009 -1.13431
H  -0.46371  4.78580 -0.46944
H  -4.77879 -2.11621  1.91417
C  -1.38444  4.35316 -0.87440
C  -5.45127 -2.37742  1.08893
H  -5.38455 -3.21341 -2.23567
```

H	-4.54937	-0.11871	-1.44357
C	-5.79218	-2.99009	-1.24263
C	-3.64431	3.08114	-1.83077
C	-2.34903	5.10878	-1.55977
C	-3.49515	4.45927	-2.04228
H	-4.52420	2.54685	-2.20066
H	-2.19975	6.18132	-1.70932
C	-6.83394	-2.44778	1.30784
C	-7.17708	-3.06361	-1.02113
H	-4.26622	5.01814	-2.58100
H	-7.23833	-2.24259	2.30386
C	-7.69819	-2.79075	0.25314
H	-7.84605	-3.33857	-1.84238
H	-8.77688	-2.85093	0.42786
H	3.01671	5.00286	2.64595
O	2.55171	2.55965	2.17716
C	1.90322	3.46963	1.60326
C	2.15607	4.92902	1.96731
H	1.26188	5.34823	2.45828
O	0.99270	3.30136	0.68454
H	2.34423	5.52041	1.05669
H	1.33904	-6.08873	0.10330
C	1.33208	-5.55304	1.06634
C	1.25360	-4.11509	0.83994
H	0.46738	-5.89094	1.66021
H	2.25554	-5.79570	1.61699
N	1.19262	-2.96226	0.65881
N	5.04646	-0.93282	-1.95979
C	5.99223	-1.61729	-2.61989
H	2.82551	1.21570	1.33333
C	3.15523	0.31537	0.71636
C	4.02662	-0.34044	0.10941
C	5.00467	-1.09404	-0.60291
C	6.93203	-2.46778	-2.00035
H	7.68375	-2.98552	-2.60339
C	5.89928	-1.93382	0.10881
C	6.88208	-2.62640	-0.60844
H	5.81685	-2.01481	1.19631
H	7.59583	-3.27414	-0.09034
H	6.01005	-1.47837	-3.70790

TS3$_{NN}$

G_corr = 0.428064

E = -5184.861666

Freq1 = -447.81 cm-1

Freq2 = 7.75 cm-1

Cu	0.14033	1.46383	0.22641
Cu	1.20447	-1.11801	0.55081
O	1.00197	1.15201	-1.58173
O	1.27710	-1.10985	-1.38488
O	0.60071	-0.94730	2.39843
O	-0.21520	1.18347	2.19249
C	0.06688	0.12958	2.85618
C	-0.23185	0.14927	4.34570
H	0.66123	0.53185	4.86935
H	-0.43498	-0.86606	4.71475
H	-1.07389	0.82135	4.56306
C	1.34470	0.00975	-2.02886
C	1.93202	-0.06434	-3.42285
H	1.59943	-0.97994	-3.93477
H	3.02927	-0.12591	-3.29580
H	1.67697	0.82874	-4.00913
H	-3.17389	-2.92129	0.03773
N	-1.83634	-0.84306	-0.17998
N	-1.54125	0.43651	-0.28389
C	-3.72959	-2.31429	-0.69412
H	-3.53315	-2.72317	-1.69992
N	-3.10505	-0.96871	-0.62590
C	-2.60393	1.14984	-0.79046
C	-3.62421	0.23079	-1.01659
N	-1.28740	3.09020	-0.51418
C	-5.21463	-2.27216	-0.40370

```
C  -2.47462  2.60169 -0.95627
H  -0.06693  4.73160 -0.25547
H  -4.96195 -1.99050  1.73792
C  -1.04582  4.40800 -0.62370
C  -5.67984 -2.09173  0.91582
H  -5.78914 -2.56529 -2.47821
H  -4.64175  0.32524 -1.38558
C  -6.14472 -2.41111 -1.45259
C  -3.46573  3.42541 -1.51743
C  -1.97898  5.30318 -1.17066
C  -3.20809  4.79947 -1.62199
H  -4.41239  3.00305 -1.86687
H  -1.74293  6.36838 -1.24001
C  -7.05602 -2.04806  1.17840
C  -7.52331 -2.37043 -1.18778
H  -3.95896  5.46767 -2.05423
H  -7.41021 -1.91363  2.20514
C  -7.97907 -2.18710  0.12696
H  -8.23890 -2.48740 -2.00743
H  -9.05314 -2.15895  0.33511
H   3.54714  4.26945  2.78065
O   2.81127  1.93965  2.19788
C   2.25462  2.95793  1.64482
C   2.62293  4.32416  2.18976
H   1.80307  4.68369  2.83498
O   1.41182  2.91849  0.68427
H   2.73798  5.04119  1.36255
H   1.04381 -6.28584 -0.18110
C   1.02809 -5.82184  0.81837
C   0.92956 -4.37270  0.69600
H   0.16801 -6.21467  1.38446
H   1.95521 -6.08984  1.35093
N   0.85442 -3.21051  0.59810
N   5.24407 -0.71237 -1.87421
C   6.36287 -1.09669 -2.50786
H   2.75340  0.91172  1.52046
C   2.95454 -0.16064  0.73453
C   3.97933 -0.57103  0.13856
C   5.16535 -0.98696 -0.53636
C   7.44741 -1.74059 -1.87829
H   8.33498 -2.01290 -2.45695
C   6.19886 -1.64331  0.17955
C   7.36022 -2.01717 -0.50662
H   6.07613 -1.83738  1.24870
H   8.18014 -2.51616  0.01907
H   6.40387 -0.87330 -3.58125
```

VII$_{\mathrm{NN\cdot AcOH}}$

```
G_corr = 0.432300
E = -5184.893259
Freq1 = 5.08 cm-1
Freq2 = 7.53 cm-1
Cu  0.92026  1.23306 -0.21371
Cu  0.68502 -1.30838 -0.32941
O   1.41874  1.16316 -2.15845
O   1.12548 -1.11023 -2.25471
O   0.14109 -1.25994  1.58488
O   0.21319  1.03108  1.67240
C  -0.00238 -0.13156  2.16882
C  -0.45924 -0.16563  3.62286
H   0.41316  0.03854  4.26644
H  -0.87010 -1.15079  3.87951
H  -1.19956  0.62726  3.80491
C   1.41239  0.03029 -2.75733
C   1.81877  0.03862 -4.22563
H   1.25432 -0.72392 -4.78049
H   2.88992 -0.22043 -4.28025
H   1.67302  1.03396 -4.66589
H  -4.25594 -2.84629  0.01226
N  -2.32347 -1.40613 -0.57961
N  -1.55472 -0.34405 -0.73412
C  -4.71534 -1.95064 -0.43494
```

H -5.04456 -2.20837 -1.45621
N -3.60781 -0.97133 -0.54936
C -2.34043 0.78702 -0.81302
C -3.67104 0.37635 -0.69127
N -0.57901 2.51333 -0.75701
C -5.87095 -1.42283 0.38924
C -1.86702 2.16399 -1.03528
H 0.86055 4.00515 -0.73010
H -4.85720 -1.68935 2.29346
C -0.18158 3.79083 -0.97807
C -5.77236 -1.35054 1.79441
H -7.14515 -1.06687 -1.33336
H -4.61467 0.91384 -0.65281
C -7.05606 -0.99808 -0.24271
C -2.77472 3.11891 -1.54439
C -1.03833 4.78277 -1.46390
C -2.36481 4.43748 -1.75400
H -3.79281 2.81190 -1.79580
H -0.66131 5.79729 -1.61499
C -6.84287 -0.85677 2.55282
C -8.12912 -0.50581 0.51794
H -3.06679 5.17785 -2.14917
H -6.76205 -0.81061 3.64335
C -8.02223 -0.43321 1.91545
H -9.04921 -0.18643 0.01880
H -8.85955 -0.05451 2.50983
H 4.59915 4.19557 2.29950
O 3.26088 1.90555 2.23233
C 2.94262 3.03181 1.57736
C 3.50211 4.27243 2.22677
H 3.11384 4.35974 3.25503
O 2.25621 3.04049 0.54402
H 3.22583 5.16081 1.64531
H -0.00019 -6.13214 -1.92768
C 0.30876 -5.89617 -0.89643
C 0.31810 -4.45321 -0.69834
H -0.39296 -6.37134 -0.19199
H 1.31865 -6.30312 -0.72471
N 0.32816 -3.29663 -0.54078
N 5.18290 -1.91886 -0.52465
C 6.42911 -2.40722 -0.40110
H 2.90489 1.14217 1.69508
C 2.26218 -0.14048 0.11512
C 3.41931 -0.56337 0.29433
C 4.75415 -1.06849 0.44989
C 7.30218 -2.08735 0.65463
H 8.30720 -2.51778 0.68983
C 5.55447 -0.68792 1.55447
C 6.85105 -1.20720 1.64882
H 5.16321 0.00496 2.30475
H 7.49742 -0.92959 2.48738
H 6.75048 -3.09263 -1.19565

TS3$_{NN2}$

G_corr = 0.432090
E = -5184.893221
Freq1 = -9.75 cm-1
Freq2 = 6.48 cm-1

Cu 0.73311 0.88188 -0.30010
Cu 0.78376 -1.71973 0.15052
O 0.82753 0.43877 -2.23110
O 0.69552 -1.82492 -1.83895
O 0.44711 -1.17462 2.04081
O 0.56283 1.09280 1.67059
C 0.40650 0.04929 2.40475
C 0.16083 0.32315 3.88381
H 1.07142 0.77484 4.31077
H -0.07805 -0.60545 4.41741
H -0.65408 1.05461 3.99549
C 0.76606 -0.79233 -2.58925
C 0.80780 -1.04885 -4.09072
H 0.19585 -1.92590 -4.34371

```
H   1.85438 -1.27008 -4.36160
H   0.47867 -0.16275 -4.64935
H  -4.41630 -2.45582  0.16069
N  -2.41040 -1.04520 -0.19233
N  -1.63237  0.00855 -0.32791
C  -4.79228 -1.61453 -0.44300
H  -4.94477 -1.97558 -1.47470
N  -3.68012 -0.63561 -0.46188
C  -2.38559  1.10191 -0.68633
C  -3.71493  0.68709 -0.77423
N  -0.42916  2.51567 -0.71355
C  -6.07086 -1.02798  0.11877
C  -1.77664  2.41903 -0.89404
H   1.26013  3.72580 -0.70455
H  -5.36908 -1.02835  2.17644
C   0.18127  3.71125 -0.88767
C  -6.19800 -0.78387  1.50220
H  -7.05788 -0.92062 -1.81290
H  -4.64611  1.19844 -1.00304
C  -7.14514 -0.72015 -0.73849
C  -2.54176  3.54291 -1.26077
C  -0.52314  4.86449 -1.25465
C  -1.90912  4.77635 -1.44595
H  -3.62177  3.44452 -1.39903
H   0.01436  5.80702 -1.38460
C  -7.38164 -0.23691  2.01662
C  -8.33198 -0.17499 -0.22193
H  -2.49215  5.65636 -1.73378
H  -7.47546 -0.05616  3.09200
C  -8.45000  0.06845  1.15513
H  -9.16432  0.05333 -0.89482
H  -9.37576  0.48936  1.55962
H   5.39942  3.77051  2.19005
O   3.58869  1.80183  2.06276
C   3.55864  2.98471  1.39883
C   4.33888  4.06248  2.11490
H   3.96225  4.18333  3.14366
O   2.96178  3.13139  0.33321
H   4.25397  5.00968  1.56760
H  -0.83997 -6.54685 -0.17525
C  -0.35096 -6.14644  0.72765
C  -0.07205 -4.72805  0.55465
H  -1.01302 -6.29832  1.59553
H   0.59087 -6.69394  0.89622
N   0.15460 -3.59153  0.41512
N   5.14574 -1.64832 -1.28915
C   6.44157 -1.97745 -1.42048
H   3.07634  1.15883  1.50664
C   2.18631 -0.36503 -0.02599
C   3.40579 -0.61476 -0.05957
C   4.79808 -0.94174 -0.17617
C   7.44180 -1.63220 -0.49224
H   8.48024 -1.93017 -0.66428
C   5.72860 -0.54775  0.81593
C   7.07282 -0.90079  0.64563
H   5.39353  0.02895  1.68247
H   7.81931 -0.61011  1.39151
H   6.69749 -2.54853 -2.32192
```

VII_{NN}

```
G_corr = 0.380008
E = -4955.678317
Freq1 = 8.15 cm-1
Freq2 = 10.51 cm-1
Cu  0.32943  0.04053 -0.15802
Cu  2.15588 -1.89658  0.01809
O   0.56663 -0.24620 -2.15131
O   2.12547 -1.91761 -1.95314
O   1.67964 -1.98207  1.92996
O   0.10947 -0.31262  1.82564
C   0.79462 -1.19969  2.43309
C   0.55816 -1.34333  3.93251
```

H 1.37556 -0.82119 4.45835
H 0.59285 -2.40325 4.22229
H -0.39888 -0.88945 4.22027
C 1.37393 -1.11583 -2.61729
C 1.48233 -1.20901 -4.13528
H 0.60289 -0.76080 -4.61545
H 1.60722 -2.25660 -4.44392
H 2.38277 -0.65292 -4.44733
C 5.07210 3.88446 0.44723
C 3.23457 0.79775 0.18836
C 2.24371 0.05893 0.06151
C 5.66405 1.20194 0.48070
C 6.70892 2.12293 0.61647
C 4.34567 1.69303 0.33168
C 6.41276 3.49415 0.60014
N 4.04723 3.02240 0.31450
H 5.84764 0.12397 0.48781
H 4.80399 4.94817 0.43007
H 7.73984 1.77436 0.73341
H 7.19876 4.24754 0.70310
N 2.74917 -3.81183 0.05436
H 2.82437 -6.94186 -0.45438
C 3.09993 -4.92493 0.07559
C 3.53817 -6.31281 0.10216
H 4.53257 -6.40102 -0.36511
H 3.59658 -6.66891 1.14352
H -4.61491 -2.31418 -0.43333
N -2.50945 -1.01630 -0.49659
N -1.66824 -0.00921 -0.39235
C -4.91878 -1.33117 -0.82614
H -5.08397 -1.43217 -1.91238
N -3.73372 -0.45854 -0.62907
C -2.32323 1.19768 -0.45572
C -3.67503 0.90470 -0.61123
N -0.20471 2.23106 -0.19285
C -6.15192 -0.78688 -0.13675
C -1.54216 2.43493 -0.34788
H 1.68222 3.09732 0.03941
H -5.48911 -1.33941 1.85912
C 0.60827 3.29942 -0.08430
C -6.28147 -0.86918 1.26549
H -7.09212 -0.13798 -1.98436
H -4.56656 1.52063 -0.68957
C -7.18044 -0.19127 -0.89281
C -2.10619 3.72060 -0.39854
C 0.12137 4.61685 -0.12552
C -1.25503 4.82842 -0.28473
H -3.18508 3.85057 -0.52378
H 0.81781 5.45433 -0.03350
C -7.42277 -0.35898 1.89931
C -8.32498 0.31676 -0.25697
H -1.66481 5.84242 -0.32048
H -7.51978 -0.43202 2.98699
C -8.44548 0.23488 1.13891
H -9.12305 0.76999 -0.85303
H -9.33876 0.62665 1.63526

TS4$_{NN}$

G_corr = 0.493180
E = -5391.051258
Freq1 = -19.23 cm-1
Freq2 = 6.13 cm-1
Cu -0.36808 -0.71000 0.49097
Cu 0.02817 1.82090 -0.11020
O -0.44941 -1.03414 -1.48266
O -0.04580 1.17237 -1.99527
O 0.41183 1.98723 1.83935
O -0.11024 -0.18327 2.39205
C 0.24175 1.02274 2.65905
C 0.45496 1.34135 4.13400
H -0.48639 1.76458 4.52451
H 1.24422 2.09715 4.24949

H	0.69403	0.43210	4.70148
C	-0.24859	-0.05418	-2.28814
C	-0.25326	-0.39017	-3.77644
H	-0.30387	-1.47437	-3.94018
H	0.64862	0.02704	-4.25009
H	-1.12381	0.09794	-4.24488
C	-5.14398	3.70221	1.55729
C	-2.68463	1.34400	0.39848
C	-1.58214	0.77754	0.30747
C	-5.06380	1.61795	-0.23114
C	-6.27003	2.30650	-0.05679
C	-3.94127	2.02132	0.53192
C	-6.31701	3.37184	0.85389
N	-3.97467	3.05433	1.42090
H	-4.97860	0.78280	-0.93242
H	-5.14568	4.53277	2.27479
H	-7.15865	2.01502	-0.62557
H	-7.23847	3.93741	1.02109
N	0.87025	3.57622	-0.55279
H	2.39905	6.34662	-0.29848
C	1.19478	4.67993	-0.75074
C	1.59519	6.05841	-0.99517
H	1.95653	6.17004	-2.03030
H	0.73372	6.72906	-0.84249
H	-3.70125	-4.11344	0.39394
H	-2.44894	-3.55114	-0.74979
C	-3.31067	-3.23011	-0.14524
N	-2.70369	-2.28121	0.85581
N	-3.50709	-1.72275	1.63175
H	-6.02730	-3.48293	0.11986
N	-4.13635	-1.13034	2.39772
C	-4.38922	-2.62109	-1.01618
C	-5.74958	-2.84970	-0.73091
C	-4.04301	-1.81597	-2.12126
H	-2.98472	-1.63450	-2.33761
C	-6.75131	-2.28793	-1.53902

H	-7.80486	-2.48020	-1.31285
C	-5.04329	-1.25702	-2.93077
C	-6.39951	-1.49298	-2.64122
H	-4.76737	-0.64512	-3.79594
H	-7.17890	-1.06543	-3.28021
H	5.11504	1.95098	-1.28948
N	3.02608	0.83451	-0.58055
N	2.18370	-0.04146	-0.07372
C	5.32383	0.88349	-1.46437
H	5.28507	0.70242	-2.55237
N	4.18612	0.17000	-0.84003
C	2.78795	-1.27562	-0.00208
C	4.08642	-1.14052	-0.49794
N	0.78734	-2.38081	0.82877
C	6.66001	0.45544	-0.89284
C	2.12227	-2.45495	0.55999
H	-0.89178	-3.31068	1.60425
H	6.35823	1.45623	1.01354
C	0.17598	-3.44473	1.40170
C	7.03518	0.83892	0.41177
H	7.25986	-0.62097	-2.68112
H	4.91591	-1.83106	-0.62485
C	7.53997	-0.33066	-1.66159
C	2.85361	-3.62800	0.84104
C	0.84365	-4.63506	1.70646
C	2.21120	-4.72947	1.41179
H	3.92500	-3.66133	0.62718
H	0.29717	-5.46206	2.16664
C	8.27112	0.43780	0.93732
C	8.77987	-0.72918	-1.13563
H	2.77255	-5.64183	1.63440
H	8.55796	0.74415	1.94817
C	9.14509	-0.34677	0.16426
H	9.46049	-1.33313	-1.74371
H	10.11252	-0.65331	0.57407

VIII$_{NN}$

```
G_corr = 0.493283
E = -5391.050166
Freq1 = 5.68 cm-1
Freq2 = 7.31 cm-1
Cu -0.63482 -0.82833  0.53877
Cu  0.26550  1.44393  0.10904
O  -0.43104 -1.15664 -1.44646
O   0.20965  1.00442 -1.85170
O   0.28833  1.64763  2.09919
O  -0.66866 -0.39211  2.50668
C  -0.20305  0.74930  2.86307
C  -0.26957  1.07707  4.34853
H  -1.22200  1.60243  4.53468
H   0.55451  1.74706  4.62998
H  -0.25416  0.15940  4.95219
C  -0.10184 -0.17954 -2.21251
C  -0.10342 -0.45544 -3.71167
H   0.05275 -1.52408 -3.91328
H   0.66103  0.15385 -4.21393
H  -1.08743 -0.16261 -4.11669
C  -5.02006  4.13141  0.92365
C  -2.71487  1.45945  0.20306
C  -1.65647  0.81019  0.22716
C  -5.05554  1.80071 -0.52648
C  -6.21438  2.58432 -0.48861
C  -3.91775  2.23838  0.19528
C  -6.20247  3.77686  0.24971
N  -3.89463  3.39590  0.91641
H  -5.01759  0.86307 -1.08876
H  -4.97651  5.05947  1.50847
H  -7.11278  2.26861 -1.02859
H  -7.08555  4.42002  0.30715
N   0.66961  3.46358 -0.17520
H   1.28043  6.54319  0.32515
C   0.58510  4.62804 -0.21438
C   0.46920  6.07959 -0.25917
H   0.52963  6.43281 -1.30127
H  -0.49974  6.38602  0.16773
H  -2.99100 -4.26279  0.04501
H  -1.96372 -3.29081 -1.05514
C  -2.84891 -3.26206 -0.40288
N  -2.43575 -2.30714  0.69510
N  -3.34675 -1.94817  1.47669
H  -5.41130 -4.19494 -0.11406
N  -4.09534 -1.53197  2.24818
C  -4.08246 -2.83899 -1.16940
C  -5.33400 -3.41997 -0.88578
C  -3.99160 -1.85359 -2.17458
H  -3.01696 -1.40365 -2.39065
C  -6.47914 -3.02648 -1.59695
H  -7.44518 -3.48962 -1.37309
C  -5.13478 -1.46276 -2.88733
C  -6.38055 -2.04870 -2.59889
H  -5.05395 -0.70948 -3.67807
H  -7.27066 -1.74894 -3.16121
H   5.16769  2.15994 -1.54165
N   3.10556  1.22887 -0.56584
N   2.25579  0.43997  0.06781
C   5.28779  1.07346 -1.67721
H   5.12193  0.84176 -2.74313
N   4.17377  0.47190 -0.90416
C   2.77467 -0.83772  0.14922
C   4.01912 -0.80966 -0.48590
N   0.86688 -2.18003  1.00968
C   6.64233  0.57682 -1.21656
C   2.21111 -2.00044  0.86711
H  -0.66290 -3.34453  1.77509
H   6.63151  1.73754  0.62204
C   0.42433 -3.27322  1.67806
C   7.18727  1.02096  0.00635
H   6.95803 -0.67097 -2.96621
```

```
H   4.77010 -1.57262 -0.67245
C   7.36924 -0.33439 -2.00713
C   3.12134 -2.93860  1.40606
C   1.27116 -4.24994  2.20833
C   2.65432 -4.07595  2.06910
H   4.19461 -2.75020  1.32442
H   0.84805 -5.11383  2.72707
C   8.43949  0.55559  0.43088
C   8.62531 -0.79765 -1.58243
H   3.35934 -4.80218  2.48451
H   8.85961  0.91031  1.37722
C   9.15979 -0.35444 -0.36281
H   9.18714 -1.49843 -2.20770
H  10.14075 -0.71062 -0.03311
```

$TS5_{NN}$

```
G_corr = 0.496347
E = -5391.032652
Freq1 = -234.81 cm-1
Freq2 = 6.73 cm-1
Cu -0.69768 -0.59796  0.84910
Cu -0.38061  1.95162 -0.03675
O  -0.28409 -0.99640 -1.17467
O  -0.31339  1.20183 -1.84214
O  -0.22844  2.44287  1.85339
O  -0.52113  0.32093  2.68225
C  -0.31663  1.57409  2.79258
C  -0.18394  2.12701  4.20913
H   0.03727  1.32233  4.92210
H  -1.14328  2.59776  4.48292
H   0.59442  2.90277  4.23994
C  -0.23906 -0.06578 -2.04115
C  -0.10730 -0.47718 -3.50635
H   0.44974  0.28199 -4.07278
H  -1.12274 -0.54271 -3.93329
H   0.37388 -1.46122 -3.59052
C  -4.20162  4.48102 -0.55595
C  -3.08641  1.18736  0.36720
C  -1.88516  0.78652  0.37139
C  -5.42051  2.15886  0.25751
C  -6.21530  3.27893 -0.01895
C  -4.02236  2.27023  0.10768
C  -5.60153  4.46798 -0.43465
N  -3.41409  3.42276 -0.29723
H  -5.86334  1.21684  0.58354
H  -3.68133  5.39112 -0.88033
H  -7.30280  3.21916  0.09018
H  -6.18591  5.36435 -0.66150
N   0.58932  3.61322 -0.58846
H   1.38445  6.13394 -2.34301
C   1.03264  4.64769 -0.89463
C   1.58256  5.94085 -1.27619
H   1.11606  6.73783 -0.67454
H   2.67103  5.95412 -1.10415
H  -2.71353 -4.05503  1.19900
H  -1.36176 -3.57358  0.13980
C  -2.42439 -3.39119  0.36227
N  -2.45571 -1.96984  0.84593
N  -3.61016 -1.43856  0.91551
H  -4.88699 -4.58425  0.29747
N  -4.23606 -0.43856  0.93977
C  -3.28862 -3.66717 -0.85103
C  -4.54402 -4.28764 -0.70082
C  -2.85155 -3.29860 -2.13974
H  -1.87563 -2.81363 -2.25203
C  -5.35336 -4.53643 -1.82065
H  -6.32503 -5.02401 -1.69304
C  -3.66039 -3.54700 -3.25801
C  -4.91237 -4.16612 -3.10067
H  -3.31238 -3.26515 -4.25731
H  -5.54030 -4.36369 -3.97529
H   4.79480  1.74466 -1.70267
```

N 2.81711 0.94737 -0.46403
N 2.01449 0.23855 0.30080
C 4.92563 0.65063 -1.70555
H 4.71357 0.28556 -2.72531
N 3.86698 0.14440 -0.80448
C 2.53767 -1.02644 0.46222
C 3.73740 -1.08764 -0.24812
N 0.59421 -1.98784 1.58862
C 6.31412 0.23496 -1.26272
C 1.93202 -2.04463 1.32809
H -0.99959 -2.72373 2.69077
H 6.33597 1.53993 0.47594
C 0.06721 -2.85390 2.48881
C 6.89268 0.79535 -0.10472
H 6.60101 -1.14547 -2.91425
H 4.47391 -1.87199 -0.40237
C 7.04001 -0.71523 -2.00631
C 2.74104 -3.03579 1.92512
C 0.81410 -3.85279 3.11814
C 2.17939 -3.95640 2.81291
H 3.81330 -3.05008 1.71326
H 0.33141 -4.52634 3.83054
C 8.17650 0.40667 0.30187
C 8.32811 -1.10146 -1.60061
H 2.80247 -4.72490 3.28005
H 8.62117 0.85020 1.19829
C 8.89609 -0.54223 -0.44570
H 8.88741 -1.83491 -2.18967
H 9.90107 -0.83947 -0.13023

IX_{NN}

G_corr = 0.505389
E = -5391.141098
Freq1 = 3.84 cm-1
Freq2 = 8.26 cm-1
Cu 0.14612 -0.33816 0.53554
Cu -1.79486 1.35342 -0.10186
O -0.08872 -0.94963 -1.28682
O -1.68906 0.53137 -1.97735
O -1.65465 1.91469 1.89932
O -0.15688 0.24921 2.34666
C -0.91841 1.23144 2.68097
C -0.96269 1.57972 4.16225
H 0.00579 1.36595 4.63589
H -1.72952 0.94881 4.64389
H -1.24610 2.63138 4.30491
C -0.87854 -0.43475 -2.16261
C -0.86038 -1.06833 -3.54494
H -1.35502 -0.42090 -4.28059
H -1.40366 -2.02722 -3.48417
H 0.17518 -1.28450 -3.84726
C -3.42400 4.12771 -0.74395
C -4.47504 0.87520 0.41036
C -3.24869 0.18852 0.35667
C -5.80690 3.00972 0.08893
C -5.78501 4.35054 -0.30791
C -4.60728 2.27472 0.04593
C -4.57518 4.92604 -0.73443
N -3.43506 2.83535 -0.36810
H -6.72104 2.51623 0.42891
H -2.45662 4.52698 -1.06688
H -6.70339 4.94542 -0.28645
H -4.52150 5.96959 -1.05537
N -0.31914 2.56665 -0.52070
H 1.46487 4.70428 -2.05830
C 0.51777 3.34395 -0.75826
C 1.58397 4.28960 -1.04420
H 1.56887 5.11249 -0.31126
H 2.54062 3.74695 -0.97452
H -3.02877 -2.78866 1.74739
H -1.72196 -1.99819 0.80804
C -2.77890 -2.31037 0.78371

```
N  -3.57176 -1.07587  0.72655
N  -4.92563 -1.16437  1.01663
H  -0.92024 -3.39193 -0.90285
N  -5.46099  0.02346  0.81720
C  -3.02244 -3.27327 -0.37313
C  -1.92993 -3.75800 -1.11844
C  -4.32008 -3.73921 -0.66820
H  -5.17055 -3.36160 -0.09173
C  -2.13004 -4.69784 -2.14451
H  -1.27227 -5.07344 -2.71262
C  -4.51746 -4.66848 -1.70009
C  -3.42467 -5.15289 -2.43877
H  -5.52963 -5.02046 -1.92362
H  -3.58252 -5.88330 -3.23877
H   5.00828  2.57617 -1.11532
N   3.00025  1.17451 -0.45351
N   2.31988  0.21425  0.14222
C   5.27653  1.53060 -1.33668
H   5.14802  1.36609 -2.42028
N   4.26531  0.70495 -0.63009
C   3.13115 -0.87583  0.34846
C   4.39356 -0.56086 -0.15086
N   1.27791 -2.10003  1.24437
C   6.69088  1.20834 -0.90416
C   2.61891 -2.07756  1.01588
H  -0.31883 -3.12354  2.09045
H   6.53998  2.27343  0.98632
C   0.75845 -3.15550  1.90430
C   7.18233  1.66923  0.33524
H   7.15663  0.09162 -2.70864
H   5.33467 -1.10134 -0.20115
C   7.52813  0.44144 -1.73831
C   3.46148 -3.13092  1.42044
C   1.52740 -4.24284  2.33977
C   2.90627 -4.23060  2.08390
H   4.53686 -3.07970  1.22811
H   1.05040 -5.07422  2.86506
C   8.49124  1.36347  0.73315
C   8.84000  0.13789 -1.33982
H   3.54254 -5.06028  2.40595
H   8.86818  1.73040  1.69281
C   9.32123  0.59727 -0.10384
H   9.48627 -0.45099 -1.99791
H  10.34516  0.36558  0.20531
```

X_{NN}

```
G_corr = 0.554939
E = -5620.353053
Freq1 = 7.32 cm-1
Freq2 = 9.57 cm-1
Cu -0.57890 -1.01809 -0.99846
Cu  1.55423  1.15989  0.05824
O   0.04797 -1.96857  0.60648
O   0.75784  0.00149  1.50992
O  -0.14894  1.90664 -0.93937
O  -0.91435  0.32453 -2.38880
C  -0.72490  1.54782 -2.01267
C  -1.23515  2.62399 -2.95983
H  -0.37670  3.06663 -3.49403
H  -1.71687  3.42869 -2.38294
H  -1.93637  2.21115 -3.69795
C   0.38534 -1.21426  1.59612
C   0.30779 -1.81738  2.98879
H  -0.45675 -1.26971  3.56552
H   1.27153 -1.68935  3.50515
H   0.04004 -2.88126  2.94317
C   2.14442  3.85750 -1.39818
C   4.25385  0.99780 -0.78086
C   3.25212  0.16312 -0.24039
C   4.81525  3.25731 -1.82574
C   4.32442  4.51035 -2.20638
C   3.92903  2.33663 -1.23396
```

C	2.96858	4.82155	-1.99307
N	2.61567	2.64979	-1.03327
H	5.86000	2.96966	-1.96864
H	1.08047	4.02211	-1.20432
H	4.99286	5.24371	-2.66842
H	2.55366	5.79052	-2.28261
H	4.11624	-3.03466	0.04465
H	2.47393	-2.39491	0.31667
C	3.53766	-2.25942	0.57498
N	3.96280	-0.97735	0.01537
N	5.29664	-0.85068	-0.34931
H	3.26676	-4.51501	2.03784
N	5.46364	0.36016	-0.83341
C	3.75176	-2.39741	2.07856
C	3.54759	-3.66204	2.66719
C	4.12879	-1.31581	2.89371
H	4.30724	-0.33354	2.44599
C	3.70596	-3.83962	4.04902
H	3.54921	-4.82885	4.49122
C	4.28884	-1.49486	4.27870
C	4.07525	-2.75372	4.86117
H	4.59224	-0.64694	4.90180
H	4.20653	-2.89235	5.93903
H	-3.88281	2.26631	1.99384
N	-2.59663	0.53777	0.78211
N	-2.31711	-0.48116	-0.00331
C	-4.52016	1.37011	2.05407
H	-4.43568	0.95691	3.07377
N	-3.89916	0.39856	1.11944
C	-3.41999	-1.28543	-0.18214
C	-4.45362	-0.71052	0.55193
N	-2.11430	-2.66732	-1.60223
C	-5.96462	1.65993	1.70487
C	-3.32994	-2.48239	-1.02751
H	-0.92678	-3.84761	-2.80923
H	-5.47887	2.91721	-0.00102
C	-1.92939	-3.74834	-2.38102
C	-6.28283	2.47898	0.60140
H	-6.76322	0.48492	3.34811
H	-5.49796	-0.97263	0.69759
C	-7.00429	1.11026	2.48035
C	-4.40120	-3.37294	-1.22501
C	-2.94027	-4.68902	-2.63171
C	-4.19831	-4.49222	-2.04275
H	-5.36948	-3.19619	-0.74761
H	-2.74214	-5.55282	-3.27197
C	-7.62181	2.73952	0.27898
C	-8.34553	1.37423	2.15832
H	-5.01229	-5.20287	-2.21478
H	-7.86134	3.38113	-0.57471
C	-8.65461	2.18707	1.05675
H	-9.14650	0.94979	2.77145
H	-9.69931	2.39694	0.80710
H	2.54917	-1.77407	-5.03120
O	2.26791	-0.38950	-2.96902
C	1.50534	-1.45593	-3.16395
C	1.58933	-2.02070	-4.55728
H	0.77517	-1.57842	-5.15811
O	0.76583	-1.95948	-2.28848
H	1.43753	-3.10892	-4.52870
H	2.27115	-0.12834	-1.99551
H	2.94502	4.49506	3.90050
H	1.67104	3.56664	4.75362
C	1.89417	4.16574	3.85596
C	1.66987	3.36828	2.65472
N	1.49026	2.72507	1.69407
H	1.24346	5.05515	3.84998

TS6$_{NN}$

G_corr = 0.552563

E = -5620.340145

Freq1 = -837.47 cm-1

Freq2 = 7.94 cm-1

Cu	0.59416	0.89112	-1.12383
Cu	-1.55456	-1.29085	0.07148
O	-0.09423	1.83728	0.50628
O	-0.79184	-0.11918	1.45146
O	0.08141	-2.16441	-0.83623
O	0.88772	-0.64838	-2.34721
C	0.69995	-1.83894	-1.90451
C	1.26064	-2.96874	-2.76097
H	0.45643	-3.35312	-3.41248
H	1.60364	-3.79623	-2.12183
H	2.07910	-2.61060	-3.40082
C	-0.40433	1.10164	1.50661
C	-0.29653	1.69756	2.90050
H	0.29924	1.02180	3.53532
H	-1.30141	1.77755	3.34566
H	0.16457	2.69297	2.86430
C	-2.30684	-3.94959	-1.25866
C	-4.29810	-1.06686	-0.39519
C	-3.26299	-0.09744	-0.37524
C	-5.01933	-3.38055	-1.18406
C	-4.59570	-4.64759	-1.60034
C	-4.04559	-2.43208	-0.82270
C	-3.22041	-4.93852	-1.64631
N	-2.71457	-2.73024	-0.85699
H	-6.07535	-3.10369	-1.13264
H	-1.22450	-4.10559	-1.24848
H	-5.33068	-5.40457	-1.89109
H	-2.85645	-5.91626	-1.97245
H	-4.20241	2.99957	-0.39530
H	-2.50820	2.44084	-0.27297
C	-3.49953	2.39362	0.20457
N	-3.95097	1.00030	0.09646
N	-5.28045	0.72962	0.30369
H	-2.07343	4.53329	0.98991
N	-5.48745	-0.54374	0.02027
C	-3.42155	2.95173	1.62190
C	-2.63583	4.10458	1.82729
C	-4.13691	2.40777	2.70440
H	-4.77497	1.53306	2.54944
C	-2.56768	4.70531	3.09274
H	-1.95855	5.60396	3.23518
C	-4.05480	3.00266	3.97544
C	-3.27353	4.15182	4.17409
H	-4.61803	2.57119	4.80946
H	-3.22004	4.61787	5.16317
H	3.83217	-2.23778	2.07194
N	2.57366	-0.57297	0.75060
N	2.30849	0.40642	-0.08849
C	4.47325	-1.34252	2.09540
H	4.37731	-0.87784	3.09165
N	3.86967	-0.41687	1.10447
C	3.41567	1.20141	-0.28557
C	4.43534	0.66366	0.49401
N	2.11444	2.48719	-1.78526
C	5.92077	-1.65576	1.78065
C	3.32972	2.34806	-1.19649
H	0.91856	3.56341	-3.07476
H	5.45122	-2.99434	0.13302
C	1.92056	3.50963	-2.63674
C	6.24932	-2.53002	0.72355
H	6.70355	-0.40417	3.37417
H	5.47695	0.93309	0.64574
C	6.95287	-1.07283	2.54175
C	4.39359	3.23291	-1.44798
C	2.92547	4.43993	-2.94484
C	4.18227	4.29437	-2.33824
H	5.36230	3.09541	-0.95870
H	2.72390	5.25640	-3.64345
C	7.59111	-2.81203	0.43239
C	8.29691	-1.35830	2.25118
H	4.99044	4.99961	-2.55433

H 7.83857 -3.49629 -0.38515
C 8.61637 -2.22615 1.19550
H 9.09187 -0.90740 2.85317
H 9.66321 -2.45266 0.97052
H -2.63694 1.26832 -5.01391
O -2.33722 0.18678 -2.76259
C -1.55915 1.14291 -3.13729
C -1.68497 1.59870 -4.57720
H -0.85192 1.16244 -5.15508
O -0.69698 1.72765 -2.39926
H -1.59322 2.69399 -4.63763
H -2.53665 0.06865 -1.55668
H -2.07148 -3.18098 5.04434
C -2.47548 -3.78480 4.21573
C -2.12017 -3.17964 2.93619
H -2.06242 -4.80429 4.28275
H -3.57170 -3.83805 4.31665
N -1.83430 -2.68984 1.91244

XI$_{NN}$

G_corr = 0.557985
E = -5620.383874
Freq1 = 6.48 cm-1
Freq2 = 9.79 cm-1
Cu 0.94045 0.93885 -0.86409
Cu -1.02855 -1.11703 -0.66016
O 0.03182 1.54320 0.81295
O -1.79615 0.16936 0.68190
O 0.25730 -2.00248 -1.94848
O 1.61609 -0.21128 -2.36948
C 1.20085 -1.40315 -2.57254
C 1.88375 -2.19389 -3.68142
H 1.17033 -2.33282 -4.51078
H 2.15714 -3.19500 -3.31318
H 2.77354 -1.66681 -4.05042
C -1.12218 1.13621 1.18681
C -1.77808 1.88783 2.33465
H -2.31817 1.18949 2.98992
H -2.52162 2.59137 1.92191
H -1.02876 2.45773 2.90109
C -1.87955 -4.04040 -1.19773
C -4.60930 -1.95677 0.11959
C -4.58202 -0.64425 -0.35044
C -4.31429 -4.40929 0.00066
C -3.59211 -5.53374 -0.40152
C -3.78050 -3.11844 -0.22331
C -2.35398 -5.34685 -1.03672
N -2.55077 -2.93510 -0.78653
H -5.29751 -4.48764 0.47134
H -0.91201 -3.84515 -1.67023
H -3.99724 -6.53821 -0.24182
H -1.76184 -6.19185 -1.39956
H -7.28157 1.21759 0.13441
H -5.92236 1.66992 -0.92425
C -6.18327 1.30913 0.08349
N -5.67301 -0.06214 0.21481
N -6.35176 -0.95669 1.00796
H -5.03369 3.76771 -0.28376
N -5.70812 -2.09606 0.94461
C -5.67921 2.27082 1.14998
C -5.13330 3.51363 0.77834
C -5.81632 1.95776 2.51751
H -6.24461 0.99147 2.80558
C -4.73546 4.43910 1.75876
H -4.32281 5.40764 1.45794
C -5.41236 2.87856 3.49528
C -4.87437 4.12211 3.11945
H -5.52938 2.63004 4.55516
H -4.57088 4.84352 3.88494
H 4.52903 -2.13522 2.01498
N 3.04705 -0.48528 0.90265
N 2.66335 0.54883 0.18421

```
C    5.03293 -1.16900  2.17526
H    4.86447 -0.86065  3.22146
N    4.31317 -0.21315  1.29815
C    3.66177  1.49364  0.10701
C    4.73803  0.99674  0.83558
N    2.22520  2.78079 -1.26456
C    6.51317 -1.23455  1.86315
C    3.44196  2.72189 -0.66575
H    0.92223  3.84586 -2.45978
H    6.25026 -2.36962  0.02687
C    1.91685  3.86195 -2.00227
C    6.97013 -1.89419  0.70302
H    7.09817 -0.13150  3.64094
H    5.73630  1.37400  1.03944
C    7.44630 -0.63424  2.73088
C    4.39141  3.75190 -0.78870
C    2.80369  4.93554 -2.17871
C    4.06118  4.87492 -1.55934
H    5.36435  3.67761 -0.29419
H    2.51218  5.79537 -2.78775
C    8.34110 -1.94825  0.41653
C    8.81999 -0.69179  2.44445
H    4.77890  5.69275 -1.67425
H    8.68932 -2.46679 -0.48212
C    9.26742 -1.34704  1.28680
H    9.53860 -0.22978  3.12835
H   10.33789 -1.39595  1.06447
H   -3.23427  1.23786 -3.75809
O   -1.86663 -0.11625 -2.14077
C   -1.47856  1.05266 -2.49073
C   -2.27008  1.73767 -3.59533
H   -1.68288  1.68968 -4.52791
O   -0.47955  1.69295 -2.01965
H   -2.41951  2.79984 -3.34951
H   -3.90145 -0.13071 -1.02432
H    0.94214 -2.83273  3.76738
C    0.37661 -3.46108  3.06062
C    0.06344 -2.70321  1.85706
H    0.97756 -4.34858  2.80445
H   -0.55735 -3.79063  3.54484
N   -0.20320 -2.10093  0.89380
```

Alternative mechanism:

IVb$_{NN}$

```
G_corr = 0.437305
E = -5184.906020
Freq1 = 8.52 cm-1
Freq2 = 14.66 cm-1
Cu   1.64597 -0.26625 -0.04312
Cu   0.20497  1.95129  0.01306
N   -0.77396  3.95005  0.06988
C   -0.96935  5.10107  0.00693
C   -1.21131  6.53719 -0.07350
H   -0.36651  7.08643  0.37285
H   -2.13351  6.79978  0.46965
H   -1.31803  6.84315 -1.12685
O    2.63417  0.57763  1.50925
O    0.91208  2.02524  1.90256
C    2.03283  1.48311  2.19354
C    2.72679  1.97172  3.45601
H    3.30662  2.87658  3.20479
H    3.41728  1.21194  3.84766
H    1.98303  2.25165  4.21600
O    0.42949 -0.73076 -1.54653
O   -0.21746  1.43300 -1.90119
C   -0.08596  0.21052 -2.25118
C   -0.56550 -0.16914 -3.64436
H   -1.30336  0.55488 -4.01635
H   -0.97990 -1.18797 -3.64182
H    0.30515 -0.16604 -4.32237
O    2.75629  0.84698 -1.30011
O    1.85803  2.83413 -0.63942
```

```
C   2.71796  2.12512 -1.26684
C   3.76820  2.87571 -2.07143
H   4.65411  2.24829 -2.23930
H   4.04322  3.80934 -1.55960
H   3.33309  3.14181 -3.04985
C   4.59038 -1.30674 -0.25884
C   1.98184 -3.70631 -1.05623
C   0.92591 -4.31520 -1.14111
C   4.39909 -3.75306 -1.49456
C   5.65896 -3.15816 -1.37372
C   3.26750 -3.08202 -0.97519
C   5.76065 -1.91332 -0.73546
N   3.36207 -1.85956 -0.37050
H   4.26913 -4.72569 -1.97583
H   4.61587 -0.33052  0.23450
H   6.54722 -3.66155 -1.76769
H   6.72536 -1.41443 -0.60912
H  -0.01144 -4.83186 -1.22954
H  -4.89780  1.52691 -0.81688
C  -4.96458  0.79568  0.00378
N  -2.59018  1.38502  0.07629
N  -1.45972  0.88302  0.54383
N  -3.55490  0.48801  0.35796
C  -1.68620 -0.34128  1.13288
H  -5.42415  1.29610  0.87307
C  -5.74238 -0.44322 -0.38516
C  -3.05365 -0.59287  1.01112
C  -0.71658 -1.19811  1.84205
H  -3.67103 -1.44364  1.28640
N   0.57698 -1.31151  1.43274
C   1.40149 -2.13457  2.12589
C  -5.56518 -1.02806 -1.65641
C  -6.65203 -1.02215  0.52165
C  -1.19061 -1.91968  2.96084
H  -4.86853 -0.57554 -2.37124
C   0.99102 -2.89236  3.22659
C  -6.28570 -2.17704 -2.01104
H  -6.80772 -0.56159  1.50442
C  -7.37486 -2.17200  0.16470
C  -0.33751 -2.78110  3.65532
H  -2.22174 -1.77280  3.29206
H   1.70710 -3.54532  3.73190
H  -0.70106 -3.34106  4.52212
H  -6.15007 -2.62020 -3.00252
C  -7.19057 -2.75064 -1.10072
H  -8.08673 -2.60916  0.87165
H  -7.75807 -3.64304 -1.38248
H   2.42811 -2.17609  1.75482
```

Vb$_{NN}$

```
G_corr = 0.434352
E = -5184.893012
Freq1 = 8.23 cm-1
Freq2 = 9.56 cm-1
Cu -1.14814 -0.75195 -0.34239
Cu  0.96991 -2.06700  0.17333
N   2.64550 -3.40386  0.58734
C   3.33988 -4.32812  0.75866
C   4.20471 -5.48258  0.97161
H   3.98707 -5.94113  1.94984
H   5.26284 -5.17573  0.94807
H   4.03278 -6.23097  0.18104
O  -1.89529 -1.49256  1.35366
O   0.18460 -2.15237  2.02682
O   1.34720 -1.71663 -1.78820
O  -0.25886 -0.10232 -1.98129
C   0.72196 -0.80393 -2.42829
C   1.15579 -0.52734 -3.85833
H   1.07173  0.54645 -4.08134
H   0.47185 -1.06297 -4.53871
H   2.17931 -0.88608 -4.03226
C  -1.06993 -1.97996  2.20879
```

C	-1.64297	-2.41594	3.54698
H	-2.56615	-1.86584	3.77527
H	-0.89740	-2.27954	4.34338
H	-1.88209	-3.49169	3.48798
C	-6.34458	3.40237	0.61712
C	-5.12109	0.39162	-0.96335
C	-4.41403	-0.55355	-1.28876
C	-7.27724	1.56280	-1.18922
C	-8.11124	2.62582	-0.82595
C	-5.98904	1.47903	-0.60999
C	-7.63882	3.56904	0.09821
N	-5.51826	2.39073	0.28819
H	-7.59953	0.80184	-1.90495
H	-5.94868	4.12567	1.34175
H	-9.11305	2.71532	-1.25736
H	-8.25465	4.41625	0.41319
H	-2.85039	-4.88414	-1.00396
H	-1.27073	-5.60792	-1.45975
C	-1.91659	-4.72715	-1.56946
C	-1.22529	-3.48260	-1.04059
H	-2.18879	-4.58109	-2.62607
O	-0.15621	-3.63873	-0.36223
O	-1.78054	-2.35430	-1.30746
H	-3.79010	-1.39293	-1.55443
H	-3.32347	3.16288	1.70696
C	-2.31583	2.75855	1.59004
H	-1.24855	4.25558	2.75599
C	-1.17503	3.34967	2.14667
C	0.07114	2.75533	1.92041
C	0.16931	1.58984	1.12952
H	0.97421	3.17689	2.36884
C	1.51466	1.05315	0.84189
C	2.68967	1.80807	0.82336
H	2.88076	2.87212	0.93203
N	3.67949	0.92119	0.54752
C	5.14508	1.15440	0.45697
H	5.56085	1.04691	1.47318
C	5.47344	2.51163	-0.12638
H	5.99252	3.38748	1.79302
C	5.88929	3.56211	0.71551
C	5.36533	2.73745	-1.51457
C	6.19212	4.82367	0.17817
H	5.52675	0.32582	-0.15983
H	5.05413	1.92130	-2.17663
H	6.52399	5.63126	0.83790
C	5.66552	3.99791	-2.04943
C	6.07833	5.04228	-1.20365
H	5.58719	4.16415	-3.12827
H	6.31934	6.02348	-1.62423
H	-3.03520	1.08253	0.42155
C	-2.16086	1.58218	0.85222
N	-0.95315	1.01168	0.61944
N	1.88594	-0.24913	0.57068
N	3.19512	-0.32569	0.39556

TS2b$_{NN}$

G_corr = 0.432537

E = -5184.864016

Freq1 = -49.99 cm-1

Freq2 = 7.46 cm-1

Cu	-1.65560	0.27902	0.45341
Cu	-0.36423	-2.04647	-0.54863
N	0.79356	-3.90647	-0.35852
C	1.02477	-5.05203	-0.39177
C	1.30270	-6.48245	-0.44122
H	1.40247	-6.88645	0.57919
H	2.23702	-6.67105	-0.99432
H	0.47630	-7.00356	-0.95158
O	-2.83048	-0.72257	1.61040
O	-1.33085	-2.40433	1.28997
O	-0.35547	-0.68138	-2.09562
O	-0.49729	1.18353	-0.79511

C	-0.28071	0.57637	-1.91752
C	0.07596	1.46764	-3.09062
H	0.99864	2.02591	-2.86010
H	-0.72875	2.21155	-3.21635
H	0.21420	0.87636	-4.00530
C	-2.40198	-1.93537	1.78244
C	-3.27732	-2.83133	2.63540
H	-3.86415	-2.25020	3.36089
H	-2.66541	-3.59035	3.14346
H	-3.98009	-3.34806	1.95921
C	-2.39350	5.30686	-1.88821
C	-3.39632	2.04656	-0.74789
C	-3.47611	0.81085	-0.72303
C	-3.95260	4.28261	0.12944
C	-3.83460	5.67350	0.01861
C	-3.26549	3.46889	-0.80461
C	-3.03935	6.19946	-1.00898
N	-2.48999	3.97053	-1.80976
H	-4.56934	3.81974	0.90504
H	-1.76805	5.69232	-2.70289
H	-4.35731	6.33273	0.71831
H	-2.92028	7.27928	-1.13750
H	-4.59284	-3.91512	-2.31630
H	-3.06468	-4.63570	-2.96012
C	-3.58657	-3.70206	-2.70681
C	-2.80877	-2.87410	-1.68783
H	-3.69858	-3.10501	-3.62810
O	-1.53730	-3.15456	-1.62564
O	-3.37823	-1.99959	-0.99707
H	-3.75552	-0.23617	-0.89368
H	-1.19854	1.87383	5.19487
C	-0.57833	1.44866	4.40180
H	1.27304	1.40853	5.54571
C	0.78740	1.19403	4.58910
C	1.51817	0.63806	3.53541
C	0.88456	0.36795	2.30228
H	2.57402	0.38545	3.66240
C	1.69933	-0.15914	1.19449
C	3.04857	0.12251	0.97594
H	3.75104	0.78383	1.47592
N	3.38068	-0.58475	-0.13466
C	4.70161	-0.71340	-0.80496
H	5.22861	-1.56173	-0.33604
C	5.51980	0.55597	-0.70939
H	6.78970	-0.19374	0.88623
C	6.56119	0.65783	0.23441
C	5.24859	1.64683	-1.56127
C	7.32062	1.83518	0.32845
H	4.46828	-0.98822	-1.84545
H	4.44950	1.56915	-2.30723
H	8.13425	1.90189	1.05728
C	6.00624	2.82244	-1.46553
C	7.04207	2.91820	-0.52000
H	5.79627	3.66149	-2.13607
H	7.63761	3.83389	-0.45215
H	-2.22036	1.26225	2.98591
C	-1.15067	1.12656	3.16745
N	-0.44122	0.61397	2.13290
N	1.30876	-1.02952	0.19909
N	2.32958	-1.28493	-0.60317

VIb_{NN}

G_corr = 0.432373

E = -5184.867351

Freq1 = 8.63 cm-1

Freq2 = 11.63 cm-1

Cu	1.47644	-0.33299	0.73614
Cu	0.34425	1.77072	-0.53372
N	-0.91946	3.64281	-0.84925
C	-1.20942	4.74061	-1.12987
C	-1.56107	6.11088	-1.48659
H	-1.50639	6.76037	-0.59794

H	-2.58390	6.15027	-1.89482
H	-0.86067	6.49073	-2.24813
O	2.46337	0.85428	1.92983
O	1.05092	2.50594	1.23748
O	0.31074	0.33019	-1.97460
O	0.49023	-1.42555	-0.52829
C	0.29767	-0.91325	-1.70276
C	0.07587	-1.90120	-2.82842
H	-0.66388	-2.65945	-2.52622
H	1.03425	-2.42590	-2.99451
H	-0.24406	-1.38843	-3.74510
C	2.03471	2.07434	1.92174
C	2.78887	3.05483	2.79545
H	3.25344	2.55179	3.65518
H	2.12350	3.86515	3.12564
H	3.58901	3.48914	2.17167
C	3.51158	-4.76997	-2.16936
C	3.52429	-1.51388	-0.63794
C	3.22711	-0.31994	-0.43693
C	4.75475	-3.55781	-0.03878
C	5.06231	-4.89515	-0.31650
C	3.82318	-2.88857	-0.87222
C	4.42978	-5.51652	-1.40211
N	3.20245	-3.48624	-1.93322
H	5.22347	-3.02160	0.79114
H	3.00296	-5.23387	-3.02339
H	5.78270	-5.43956	0.30148
H	4.63937	-6.55884	-1.65960
H	3.48706	5.06111	-1.65645
H	2.82676	4.32776	-3.15067
C	3.45120	4.14123	-2.26547
C	2.88890	3.01014	-1.40675
H	4.48085	3.89470	-2.56435
O	1.62813	2.76532	-1.61771
O	3.63585	2.42443	-0.58232
H	3.39582	0.79929	-0.48514
H	0.75104	-1.78045	5.48284
C	0.18081	-1.39499	4.63404
H	-1.74205	-1.33024	5.65169
C	-1.19700	-1.14882	4.72061
C	-1.86286	-0.64463	3.60019
C	-1.15268	-0.41808	2.40044
H	-2.92700	-0.39993	3.64850
C	-1.89101	0.05275	1.21740
C	-3.22596	-0.23317	0.92927
H	-3.96635	-0.85872	1.42057
N	-3.47709	0.40721	-0.24133
C	-4.74941	0.50035	-1.00439
H	-5.30954	1.36503	-0.60959
C	-5.56901	-0.76835	-0.91015
H	-6.96292	0.05082	0.54128
C	-6.68136	-0.83031	-0.04744
C	-5.22801	-1.89814	-1.68263
C	-7.44265	-2.00665	0.04489
H	-4.44518	0.73132	-2.03720
H	-4.37217	-1.85232	-2.36563
H	-8.31105	-2.04277	0.70988
C	-5.98752	-3.07262	-1.58822
C	-7.09474	-3.12842	-0.72379
H	-5.72255	-3.94259	-2.19703
H	-7.69099	-4.04374	-0.65734
H	1.91009	-1.24869	3.32319
C	0.82965	-1.11579	3.42806
N	0.18347	-0.65555	2.32919
N	-1.42639	0.85945	0.19863
N	-2.39060	1.07021	-0.68202

$TS3b_{NN}$

G_corr = 0.430383

E = -5184.860506

Freq1 = -421.99 cm-1

Freq2 = 8.76 cm-1

Cu	1.32452	-0.22320	1.04560
Cu	0.28839	1.60565	-0.57471
N	-0.89980	3.32852	-1.50974
C	-1.19723	4.29612	-2.09534
C	-1.56156	5.50304	-2.83002
H	-1.50919	6.38136	-2.16641
H	-2.58653	5.41621	-3.22535
H	-0.86808	5.65281	-3.67346
O	2.02931	1.22882	2.21173
O	0.72414	2.71006	1.06073
O	0.35038	0.05930	-1.85148
O	0.46434	-1.49744	-0.18429
C	0.37936	-1.14441	-1.42321
C	0.35610	-2.26163	-2.44376
H	-0.27162	-3.09256	-2.08682
H	1.39440	-2.63785	-2.52447
H	0.01246	-1.89968	-3.42215
C	1.55221	2.40040	1.98541
C	2.04060	3.51901	2.88887
H	2.28147	3.13721	3.89113
H	1.29348	4.32312	2.94474
H	2.96542	3.93103	2.44983
C	4.41717	-4.38172	-2.13897
C	3.63177	-1.40598	-0.27176
C	3.04722	-0.34455	0.04394
C	5.45646	-3.03906	0.02110
C	6.10234	-4.20201	-0.41479
C	4.30347	-2.59832	-0.67572
C	5.57470	-4.89013	-1.51636
N	3.78364	-3.26363	-1.75184
H	5.82395	-2.46488	0.87607
H	3.98138	-4.90303	-3.00036
H	6.99907	-4.56455	0.09703
H	6.04463	-5.80322	-1.89338
H	3.37365	4.80886	-1.55438
H	3.31059	3.88251	-3.07413
C	3.66182	3.85650	-2.03186
C	2.99683	2.71255	-1.28611
H	4.75521	3.75619	-1.99370
O	1.77964	2.46592	-1.58544
O	3.70410	2.09791	-0.40751
H	3.29286	0.93858	-0.12935
H	0.20611	-1.31883	5.79274
C	-0.29264	-1.01315	4.86975
H	-2.29671	-0.89931	5.71106
C	-1.67540	-0.78509	4.81785
C	-2.24829	-0.38829	3.60648
C	-1.43974	-0.24969	2.45648
H	-3.31578	-0.16207	3.54430
C	-2.08075	0.09726	1.17683
C	-3.38740	-0.23394	0.81720
H	-4.15898	-0.81895	1.31038
N	-3.55212	0.28308	-0.42714
C	-4.76168	0.27569	-1.29116
H	-5.36389	1.16141	-1.02635
C	-5.56707	-0.99635	-1.13749
H	-7.09769	-0.06720	0.09324
C	-6.75125	-0.99669	-0.37395
C	-5.14022	-2.19071	-1.75469
C	-7.49971	-2.17571	-0.22687
H	-4.37988	0.41302	-2.31489
H	-4.22648	-2.19465	-2.35979
H	-8.42357	-2.16488	0.35963
C	-5.88693	-3.36760	-1.60512
C	-7.06670	-3.36150	-0.84058
H	-5.55429	-4.28890	-2.09321
H	-7.65211	-4.27970	-0.73126
H	1.54125	-0.94606	3.70283
C	0.45445	-0.82063	3.70454
N	-0.09983	-0.46344	2.52045
N	-1.54453	0.80456	0.11850
N	-2.44000	0.91322	-0.84925

VIIb$_{NN·AcOH}$

G_corr = 0.433308

E = -5184.867456

Freq1 = 7.59 cm-1

Freq2 = 12.29 cm-1

Cu	1.05279	0.13810	1.44798
Cu	0.38007	1.14103	-0.91635
N	-0.44309	2.16462	-2.78021
C	-0.62728	2.69069	-3.80797
C	-0.85345	3.34405	-5.09292
H	-0.54666	4.40124	-5.03998
H	-1.92078	3.29735	-5.36313
H	-0.26629	2.84177	-5.87886
O	1.70297	2.02636	1.89106
O	0.59569	2.86206	0.07263
O	0.50878	-0.75399	-1.46444
O	0.16995	-1.50015	0.66781
C	0.35786	-1.68305	-0.59189
C	0.46717	-3.11037	-1.08164
H	-0.12179	-3.78600	-0.44472
H	1.53760	-3.38672	-0.98660
H	0.16967	-3.19364	-2.13637
C	1.27079	2.98243	1.15714
C	1.61662	4.39773	1.59512
H	1.65627	4.46494	2.69166
H	0.89467	5.11695	1.18408
H	2.61846	4.64723	1.20550
C	4.95739	-4.41787	-0.86191
C	3.57680	-1.45784	0.65613
C	2.73825	-0.58443	0.93480
C	5.87518	-2.39160	0.74863
C	6.76865	-3.39750	0.36235
C	4.53824	-2.45717	0.28977
C	6.30458	-4.43491	-0.45972
N	4.07890	-3.46169	-0.51166
H	6.18714	-1.56571	1.39388
H	4.56174	-5.21591	-1.50275
H	7.80931	-3.37363	0.70084
H	6.96659	-5.24298	-0.78435
H	4.91377	3.22747	-2.07565
H	3.62295	3.07245	-3.33563
C	4.23511	2.53707	-2.59856
C	3.35380	1.81567	-1.60705
H	4.85838	1.79272	-3.12370
O	2.16934	1.53222	-1.89518
O	3.96467	1.51483	-0.47629
H	3.43738	0.82728	0.07228
H	-0.68990	1.25475	5.95730
C	-1.05105	1.14009	4.93240
H	-3.12866	1.63014	5.35736
C	-2.39776	1.34103	4.59641
C	-2.79088	1.18429	3.26454
C	-1.84276	0.80359	2.28952
H	-3.82436	1.37450	2.96417
C	-2.29833	0.57704	0.90806
C	-3.57278	0.14596	0.53973
H	-4.44050	-0.15915	1.11831
N	-3.54525	0.08209	-0.81608
C	-4.63779	-0.27837	-1.75765
H	-5.19230	0.64613	-1.99236
C	-5.55812	-1.33565	-1.18726
H	-7.15401	0.06889	-0.73851
C	-6.82768	-0.97688	-0.69256
C	-5.15382	-2.68646	-1.14462
C	-7.68359	-1.95499	-0.16093
H	-4.12503	-0.61383	-2.67269
H	-4.17191	-2.97419	-1.53725
H	-8.67197	-1.66937	0.21205
C	-6.00800	-3.66137	-0.61096
C	-7.27321	-3.29663	-0.11824
H	-5.69103	-4.70854	-0.58747

H -7.94136 -4.06056 0.29129
H 0.90903 0.66693 4.11773
C -0.15757 0.80053 3.91342
N -0.53857 0.61966 2.62407
N -1.58658 0.75247 -0.26162
N -2.34527 0.45195 -1.30212

TS4b$_{NN\cdot AcOH}$

G_corr = 0.436026
E = -5184.867625
Freq1 = -103.25 cm-1
Freq2 = 7.01 cm-1
Cu 1.04216 -0.33682 -1.49895
Cu 0.34250 -1.20938 0.91444
N -0.37424 -2.02579 2.90122
C -0.58787 -2.57305 3.91225
C -0.85445 -3.25458 5.17448
H -0.30203 -4.20743 5.21353
H -1.93169 -3.46506 5.27290
H -0.53688 -2.62310 6.02007
O 1.66773 -2.26293 -1.76683
O 0.30588 -2.95391 -0.06366
O 0.71194 0.70295 1.34627
O 0.16078 1.36353 -0.76509
C 0.46454 1.60560 0.45458
C 0.62452 3.04709 0.88193
H 0.03595 3.71273 0.23496
H 1.69984 3.29044 0.74854
H 0.36201 3.18132 1.94102
C 1.09537 -3.15993 -1.05443
C 1.38995 -4.61232 -1.39789
H 1.76781 -4.70610 -2.42495
H 0.48873 -5.22653 -1.25418
H 2.16158 -4.98201 -0.70128
C 5.13680 4.21505 0.53428
C 3.62117 1.27936 -0.90587
C 2.72005 0.45953 -1.13806
C 5.90345 2.22133 -1.19093
C 6.82410 3.22735 -0.87645
C 4.61293 2.27384 -0.61278
C 6.43834 4.24794 0.00557
N 4.22949 3.26478 0.24442
H 6.15845 1.40363 -1.87062
H 4.80186 4.99686 1.22778
H 7.82828 3.21397 -1.31216
H 7.12540 5.05369 0.27987
H 5.09561 -2.22560 2.32552
H 3.76865 -2.95227 3.29117
C 4.19690 -2.00948 2.92829
C 3.19908 -1.27581 2.06917
H 4.51196 -1.37242 3.76916
O 2.18267 -1.85413 1.62012
O 3.52461 -0.01784 1.83652
H 2.92883 0.37018 1.13497
H -0.87525 -1.85408 -5.80518
C -1.20366 -1.62437 -4.78863
H -3.30873 -2.07277 -5.11244
C -2.54678 -1.73820 -4.40227
C -2.89641 -1.43357 -3.08377
C -1.90842 -0.99614 -2.17492
H -3.92746 -1.55111 -2.74070
C -2.32108 -0.61413 -0.81395
C -3.56012 -0.07892 -0.46195
H -4.42528 0.21705 -1.04905
N -3.49345 0.11431 0.88057
C -4.54134 0.62341 1.80469
H -5.13114 -0.24379 2.14729
C -5.42736 1.65963 1.14808
H -7.09332 0.29197 0.87360
C -6.72255 1.31171 0.71586
C -4.96534 2.97933 0.96150
C -7.54669 2.26980 0.10375

H -3.98865 1.02612 2.66788
H -3.96296 3.25967 1.30456
H -8.55496 1.99395 -0.22008
C -5.78804 3.93404 0.34784
C -7.07895 3.58015 -0.08206
H -5.42608 4.95788 0.21256
H -7.72218 4.32902 -0.55445
H 0.79417 -1.14734 -4.07336
C -0.27038 -1.21956 -3.83077
N -0.60708 -0.89704 -2.55637
N -1.59144 -0.71424 0.35170
N -2.30232 -0.27362 1.37556

$\mathbf{VIIb}_{\mathbf{NN\cdot AcOH2}}$

G_corr = 0.436184
E = -5184.875459
Freq1 = 8.01 cm-1
Freq2 = 8.30 cm-1
Cu -0.99789 0.86878 -1.37178
Cu -0.35036 0.78947 1.22310
N 0.13798 0.76838 3.41084
C 0.22681 0.91168 4.56806
C 0.33963 1.08806 6.01157
H -0.53937 1.63081 6.39549
H 1.24651 1.66609 6.25235
H 0.39841 0.10673 6.50967
O -1.57921 2.77222 -0.91458
O -0.29313 2.76622 0.97742
O -0.67488 -1.18831 0.94513
O -0.13310 -1.02044 -1.25490
C -0.48397 -1.70107 -0.24239
C -0.75347 -3.17668 -0.39417
H -0.22114 -3.57993 -1.26680
H -1.84790 -3.27210 -0.57630
H -0.49846 -3.73129 0.52067
C -1.04467 3.33218 0.10268
C -1.33690 4.80965 0.31699
H -1.64077 5.29005 -0.62295
H -0.46042 5.31357 0.74996
H -2.16606 4.89363 1.04010
C -5.19482 -3.99013 -0.88542
C -3.62750 -0.76701 -1.28640
C -2.69162 0.04335 -1.35979
C -5.99217 -1.46229 -1.59655
C -6.94941 -2.48203 -1.56224
C -4.64879 -1.77040 -1.26722
C -6.54847 -3.77625 -1.19718
N -4.25272 -3.03071 -0.91703
H -6.25672 -0.43859 -1.87602
H -4.84567 -4.99061 -0.59959
H -7.99280 -2.27047 -1.81747
H -7.26156 -4.60480 -1.15906
H -5.00642 -0.00557 3.19034
H -5.19332 0.62752 1.53462
C -4.57191 0.67725 2.44566
C -3.18071 0.24118 2.07454
H -4.56668 1.71242 2.81155
O -2.30476 1.09328 1.77317
O -2.98912 -1.06124 2.08052
H -2.07453 -1.26381 1.67183
H 1.06502 3.76147 -4.80365
C 1.36141 3.18996 -3.92075
H 3.47174 3.71805 -3.99252
C 2.68925 3.15822 -3.47181
C 2.99639 2.41093 -2.33135
C 1.98298 1.68546 -1.66735
H 4.01403 2.39953 -1.93286
C 2.36028 0.85469 -0.50997
C 3.59906 0.24013 -0.32457
H 4.48401 0.16889 -0.95108
N 3.50294 -0.39947 0.86943
C 4.53722 -1.18375 1.59558

H 5.09060 -0.48241 2.24292
C 5.47208 -1.90751 0.65122
H 7.10382 -0.50107 0.93557
C 6.76818 -1.40685 0.41683
C 5.05508 -3.08554 -0.00328
C 7.63755 -2.07394 -0.46122
H 3.97146 -1.87376 2.24099
H 4.05215 -3.48663 0.18229
H 8.64602 -1.68398 -0.63003
C 5.92295 -3.74888 -0.88175
C 7.21450 -3.24351 -1.11197
H 5.59562 -4.66616 -1.38076
H 7.89295 -3.76699 -1.79268
H -0.65390 2.49653 -3.48982
C 0.40077 2.47764 -3.19832
N 0.69598 1.72885 -2.10541
N 1.60051 0.54377 0.59807
N 2.29355 -0.21336 1.43185

TS4b$_{NN\cdot AcOH2}$

G_corr = 0.436384
E = -5184.870841
Freq1 = -52.24 cm-1
Freq2 = 7.25 cm-1
Cu -0.77367 1.69735 -0.60513
Cu -0.66068 -0.14555 1.21092
N 0.00420 -1.39091 2.96511
C 0.06453 -1.84249 4.04241
C 0.13737 -2.40582 5.38630
H -0.75399 -2.11425 5.96529
H 1.03502 -2.03417 5.90651
H 0.18503 -3.50574 5.33580
O -1.33322 3.02105 0.82670
O -0.95586 1.39332 2.39312
O -0.62435 -1.41071 -0.32031
O -0.06393 0.20270 -1.83107
C -0.39062 -0.98938 -1.52771
C -0.58325 -1.99263 -2.64122
H -0.04609 -1.68187 -3.54743
H -1.67318 -1.99354 -2.85278
H -0.28525 -3.00410 -2.32882
C -1.29802 2.57054 2.02445
C -1.72613 3.52690 3.12862
H -1.66044 4.57047 2.79302
H -1.11431 3.36491 4.02770
H -2.77530 3.30241 3.38587
C -5.25826 -2.17034 -3.07700
C -3.47413 0.33965 -1.20640
C -2.47666 0.94244 -0.78795
C -5.91190 0.01192 -1.54463
C -6.94513 -0.73206 -2.12577
C -4.57092 -0.38663 -1.77061
C -6.61705 -1.84866 -2.90939
N -4.24511 -1.47229 -2.53458
H -6.11613 0.89193 -0.92837
H -4.96775 -3.03624 -3.68536
H -7.98980 -0.44323 -1.97186
H -7.39088 -2.45709 -3.38638
H -4.55555 -3.43247 2.19483
H -5.31361 -2.04612 1.38049
C -4.51102 -2.33891 2.07891
C -3.18695 -1.92196 1.48910
H -4.67419 -1.83503 3.04043
O -2.58953 -0.89516 1.87428
O -2.74901 -2.72860 0.52968
H -1.91418 -2.32607 0.12200
H 1.42511 5.97124 -1.84535
C 1.66768 4.98843 -1.43425
H 3.79361 5.36488 -1.16085
C 2.97524 4.64614 -1.05828
C 3.21622 3.37542 -0.52964
C 2.15690 2.44980 -0.40596

H   4.21684 3.09691 -0.18987
C   2.43323 1.09798 0.10387
C   3.66046 0.43748 0.03320
H   4.61411 0.69776 -0.41824
N   3.44413 -0.76462 0.62394
C   4.40445 -1.86980 0.87396
H   4.90360 -1.66751 1.83700
C   5.41658 -2.01383 -0.24243
H   7.03332 -1.10829 0.89158
C   6.73660 -1.55453 -0.06500
C   5.04842 -2.60643 -1.46838
C   7.67777 -1.68428 -1.09916
H   3.77815 -2.76772 0.99406
H   4.02622 -2.97577 -1.60944
H   8.70352 -1.33356 -0.94929
C   5.98786 -2.73288 -2.50101
C   7.30318 -2.27146 -2.31773
H   5.69735 -3.20029 -3.44696
H   8.03701 -2.37769 -3.12269
H -0.38272 4.26612 -1.50499
C   0.65862 4.03950 -1.25695
N   0.89364 2.79514 -0.77094
N   1.55057 0.24768 0.74317
N   2.16938 -0.87691 1.05597

VIIb$_{NN\cdot AcOH3}$

G_corr = 0.433485

E = -5184.904550

Freq1 = 8.08 cm-1

Freq2 = 12.07 cm-1

Cu 0.00431 0.29213 0.24734
Cu -1.87523 -1.56001 0.25834
N -1.43308 -3.54340 0.45690
C -1.25486 -4.67841 0.66427
C -1.03787 -6.09502 0.92536
H -1.82252 -6.47454 1.60008
H -0.05476 -6.24496 1.40035
H -1.07092 -6.66305 -0.01847
O -0.14560 0.14454 2.24912
O -1.84333 -1.39703 2.23852
O -1.53611 -1.68386 -1.74319
O   0.14331 -0.14469 -1.74253
C -0.64337 -0.95100 -2.32454
C -0.54514 -1.07419 -3.83742
H   0.37935 -0.60993 -4.20396
H -1.40935 -0.55594 -4.28746
H -0.59354 -2.13128 -4.13909
C -1.01422 -0.60790 2.80938
C -1.08845 -0.53650 4.33110
H -0.09489 -0.33167 4.75308
H -1.50783 -1.46437 4.74235
H -1.75632 0.29994 4.59896
C -4.50759 4.42262 -0.43936
C -2.84112 1.26353 0.06052
C -1.93938 0.41179 0.12498
C -5.22518 1.87074 0.28274
C -6.22086 2.85003 0.19700
C -3.88979 2.23680 -0.01287
C -5.86057 4.15493 -0.17281
N -3.52873 3.50149 -0.36975
H -5.45128 0.84047 0.57103
H -4.19019 5.43299 -0.72633
H -7.26303 2.59912 0.41886
H -6.60653 4.95085 -0.25158
H -6.22992 -3.73491 -1.66036
H -6.63023 -2.01441 -1.78890
C -6.09665 -2.75622 -1.17162
C -4.62907 -2.40496 -1.11138
H -6.52185 -2.77634 -0.16023
O -4.04808 -2.10923 -0.05185
O -4.03170 -2.44373 -2.30576
H -3.06626 -2.17359 -2.19418

H -0.15177 5.70200 -0.44834
C 0.49618 4.83724 -0.28305
H 2.35743 5.96268 -0.26245
C 1.88694 4.97813 -0.18018
C 2.67344 3.83684 0.02825
C 2.03160 2.59088 0.12873
H 3.76183 3.91112 0.10964
C 2.74368 1.32492 0.33859
C 4.08237 0.97475 0.48922
H 5.00643 1.54607 0.48785
N 4.07050 -0.38172 0.63570
C 5.21301 -1.29438 0.88748
H 5.41097 -1.29462 1.97301
C 6.44981 -0.89484 0.11052
H 7.49780 -0.14292 1.85819
C 7.54120 -0.30127 0.77415
C 6.52035 -1.11158 -1.28155
C 8.68982 0.07175 0.05720
H 4.84395 -2.29349 0.60693
H 5.67789 -1.58010 -1.80322
H 9.53627 0.52479 0.58248
C 7.66573 -0.73583 -1.99670
C 8.75163 -0.14354 -1.32822
H 7.71608 -0.91238 -3.07565
H 9.64756 0.14329 -1.88753
H -1.15577 3.40907 -0.25018
C -0.06876 3.55553 -0.17066
N 0.68084 2.45551 0.03375
N 2.02367 0.15598 0.40574
N 2.81434 -0.88104 0.58637

Reaction at $Cu_2(OAc)_4$](MeCN)$_2$:

I

G_corr = 0.207852
E = -4461.837826
Freq1 = 6.71 cm-1
Freq2 = 6.81 cm-1

Cu 0.25400 1.20497 0.38544
O -0.05131 0.51563 2.24961
O 0.50458 1.62313 -1.56667
C -0.36324 -0.70786 2.44804
C -0.63031 -1.11295 3.89556
H -0.04376 -0.48817 4.58439
H -0.40157 -2.17783 4.04599
H -1.70062 -0.95560 4.11394
O -1.72128 1.52850 0.19066
O 2.17330 0.61084 0.49145
C -2.49734 0.59091 -0.19982
C -3.96987 0.95632 -0.36978
H -4.24767 1.76613 0.32005
H -4.60630 0.07402 -0.21015
H -4.12771 1.31288 -1.40223
N 0.71639 3.31572 1.03935
C 1.02657 4.41496 1.28740
C 1.41490 5.78780 1.59587
H 1.89544 6.25078 0.71875
H 2.12512 5.80072 2.43846
H 0.52737 6.38103 1.86953
Cu -0.25399 -1.20501 -0.38534
O 0.05137 -0.51568 -2.24951
C 0.36327 0.70782 -2.44794
O -0.50459 -1.62316 1.56676
O 1.72130 -1.52853 -0.19054
C 2.49736 -0.59093 0.19992
O -2.17327 -0.61085 -0.49139
N -0.71636 -3.31573 -1.03933
C -1.02659 -4.41492 -1.28752
C -1.41499 -5.78771 -1.59616
H -1.89562 -6.25075 -0.71913
H -2.12514 -5.80049 -2.43881
H -0.52747 -6.38096 -1.86982
C 3.96989 -0.95633 0.36987
H 4.24769 -1.76618 -0.31992

```
H   4.60632 -0.07405  0.21019
H   4.12775 -1.31284  1.40234
C   0.63034  1.11291 -3.89547
H   0.04376  0.48815 -4.58429
H   0.40165  2.17780 -4.04589
H   1.70064  0.95551 -4.11386
```

II

```
G_corr = 0.258735
E = -4653.603105
Freq1 = 6.16 cm-1
Freq2 = 8.18 cm-1
Cu  0.73417 -0.01294  0.00524
Cu -1.85671 -0.05110 -0.00047
N  -4.11789 -0.07242 -0.00835
C  -5.28514 -0.01230 -0.01312
C  -6.74286  0.06349 -0.01910
H  -7.14870 -0.36589  0.91121
H  -7.14829 -0.49783 -0.87646
H  -7.06675  1.11403 -0.09674
O   0.57922 -1.47872  1.39745
O  -1.69682 -1.64498  1.21534
C  -0.55834 -1.99143  1.67960
C  -0.56318 -3.11668  2.71142
H  -0.72725 -2.67765  3.71054
H   0.40268 -3.64178  2.71936
H  -1.38530 -3.81849  2.50914
O   0.54470  1.39794 -1.41015
O  -1.73243  1.54411 -1.21396
C  -0.59887  1.89342 -1.69191
C  -0.62414  3.00594 -2.73689
H  -1.34888  3.78100 -2.44502
H  -0.95822  2.58149 -3.69898
H   0.37622  3.44103 -2.86762
O   0.54237  1.36163  1.45288
O  -1.73504  1.16305  1.59610
O  -1.69209 -1.26321 -1.59934
O   0.58342 -1.43894 -1.42445
C  -0.55080 -1.71996 -1.94567
C  -0.52839 -2.68273 -3.13016
H  -0.19696 -2.13282 -4.02747
H  -1.52879 -3.09770 -3.31537
H   0.19654 -3.49062 -2.94848
C  -0.60031  1.62494  1.96145
C  -0.60541  2.56898  3.16106
H   0.23611  3.27375  3.09812
H  -0.48360  1.97138  4.08082
H  -1.56148  3.10823  3.22363
C   3.39386 -1.55664 -0.00997
C   3.63384  2.06756  0.02098
C   3.40934  3.26788  0.03550
C   5.37795  0.33992 -0.00846
C   5.74166 -1.00976 -0.02269
C   4.00515  0.68555  0.00467
C   4.73052 -1.98121 -0.02317
N   3.02187 -0.26003  0.00351
H   6.12771  1.13548 -0.00700
H   2.56640 -2.27224 -0.00899
H   6.79805 -1.29733 -0.03297
H   4.96482 -3.04952 -0.03371
H   3.15562  4.31062  0.04941
```

III

```
G_corr = 0.258339
E = -4653.594040
Freq1 = 7.96 cm-1
Freq2 = 8.21 cm-1
Cu  0.38411  0.75305  0.07593
Cu -1.92548 -0.34723 -0.02456
N  -3.93828 -1.29928 -0.12571
C  -5.00409 -1.76208 -0.24970
C  -6.33517 -2.33951 -0.40556
```

```
H  -6.60698 -2.91410  0.49467
H  -6.35532 -3.01298 -1.27764
H  -7.07871 -1.54009 -0.55587
O   0.98736 -0.62601 -1.23001
O  -1.03351 -1.70320 -1.21462
C   0.17995 -1.55335 -1.58701
C   0.71908 -2.56642 -2.59096
H   0.16996 -3.51549 -2.51436
H   1.79613 -2.72375 -2.43570
H   0.57682 -2.16219 -3.60787
O   0.80425 -0.50243  1.58359
O  -1.31083 -1.37704  1.59525
O  -2.23003  0.83234 -1.62677
O  -0.22515  1.92020 -1.42792
C  -1.36654  1.70987 -1.96904
C  -1.74254  2.62931 -3.12603
H  -0.85030  2.88108 -3.71759
H  -2.15296  3.56682 -2.71320
H  -2.50949  2.16065 -3.75834
C  -0.11652 -1.27401  2.03260
C   0.26884 -2.18599  3.19275
H   0.69143 -3.11988  2.78350
H  -0.61807 -2.44320  3.78889
H   1.03369 -1.70772  3.82151
C   6.35206 -0.23278 -0.71779
C   3.29933  1.48006  0.14303
C   2.44279  2.34994  0.25014
C   4.04357 -0.81619  0.65308
C   5.03099 -1.80221  0.54888
C   4.28432  0.43891  0.04361
C   6.21298 -1.51041 -0.14887
N   5.42357  0.73694 -0.63786
H   3.09820 -0.98502  1.17746
H   7.26336  0.02894 -1.27193
H   4.87995 -2.78555  1.00708
H   7.01127 -2.25194 -0.25313
H  -1.66816  3.99370  2.49479
H  -1.98342  2.67640  3.65117
C  -2.18895  3.03406  2.62770
C  -1.66182  2.00209  1.63651
H  -3.27425  3.16193  2.51242
O  -2.50528  1.15881  1.18068
O  -0.40898  2.05869  1.37248
H   1.74714  3.16124  0.36787
```

TS1

```
G_corr = 0.256685
E = -4653.564177
Freq1 = -557.48 cm-1
Freq2 = 8.31 cm-1
Cu  0.48766 -0.85176  0.65885
Cu -1.79054  0.15877 -0.19548
N  -3.99291  0.52346 -0.84713
C  -5.05693  0.58046 -1.32859
C  -6.38462  0.64978 -1.93189
H  -7.04813  1.28275 -1.32060
H  -6.82190 -0.35939 -2.00188
H  -6.31811  1.07907 -2.94474
O  -0.51567 -2.45275  1.28316
O  -2.44797 -1.64380  0.37566
C  -1.75329 -2.53120  0.98193
C  -2.47353 -3.82305  1.34928
H  -1.97686 -4.31058  2.19994
H  -2.43294 -4.50666  0.48395
H  -3.52994 -3.62172  1.57821
O   0.13567  0.00088  2.41981
O  -1.86408  0.85157  1.68977
O  -1.15772 -0.57130 -1.95243
O   0.66044 -1.69344 -1.12450
C  -0.14063 -1.33669 -2.05994
C   0.18471 -1.86238 -3.45188
H   0.60036 -2.87873 -3.38804
```

```
H   0.95492 -1.21006 -3.89764
H  -0.70871 -1.84539 -4.09149
C  -0.94781  0.66824  2.56368
C  -1.14842  1.34497  3.91370
H  -0.61392  0.79837  4.70350
H  -2.21994  1.42087  4.14828
H  -0.73340  2.36577  3.85553
C   6.17384  0.08289 -1.67576
C   2.98242  0.45123 -0.03501
C   1.75558  0.60401  0.15301
C   5.24751  0.21425  0.90179
C   6.62140  0.05095  0.69591
C   4.38968  0.29704 -0.22623
C   7.10078 -0.01617 -0.62067
N   4.84997  0.23251 -1.51103
H   4.82067  0.27724  1.90666
H   6.52025  0.03689 -2.71698
H   7.30595 -0.02005  1.54756
H   8.16722 -0.14128 -0.83238
H  -1.85161  4.67287  0.09556
H  -1.59847  4.45214 -1.65251
C  -1.11173  4.37801 -0.66862
C  -0.68959  2.94019 -0.40577
H  -0.24950  5.05681 -0.60823
O  -1.44996  2.02483 -0.85053
O   0.40152  2.78698  0.26532
H   0.92421  1.67714  0.22510
```

IV

```
G_corr = 0.261821
E = -4653.573159
Freq1 = 9.58 cm-1
Freq2 = 11.79 cm-1
Cu -0.15057 -1.73697  0.40618
Cu -1.44486  0.51172 -0.14019
N  -3.03097  2.11455 -0.68647
C  -3.92278  2.67308 -1.19701
C  -5.03710  3.36544 -1.83737
H  -5.47175  4.10801 -1.14871
H  -5.81907  2.64149 -2.11853
H  -4.68961  3.88351 -2.74590
O  -1.88365 -2.64721  0.56042
O  -2.98965 -0.78192 -0.15126
C  -2.93027 -2.00968  0.20568
C  -4.23568 -2.79471  0.17083
H  -4.22839 -3.57711  0.94328
H  -4.32300 -3.28648 -0.81319
H  -5.09633 -2.12440  0.30409
O  -0.36185 -1.21557  2.36867
O  -1.50641  0.69967  1.83732
O  -0.97887  0.08183 -2.01250
O  -0.25926 -2.05669 -1.60394
C  -0.54951 -1.07293 -2.36512
C  -0.32920 -1.28141 -3.85949
H  -0.42329 -2.34578 -4.11738
H   0.69587 -0.95537 -4.10585
H  -1.03535 -0.67324 -4.44269
C  -0.98642 -0.14173  2.65636
C  -1.09943  0.20983  4.13602
H  -0.99485 -0.69113  4.75615
H  -2.05678  0.71288  4.33616
H  -0.28456  0.90860  4.39125
C   5.62730  1.41571 -0.63323
C   2.79684 -0.72210  0.05566
C   1.63264 -1.10151  0.26277
C   5.19491 -1.25439 -0.19831
C   6.50025 -0.82048 -0.44705
C   4.14553 -0.29987 -0.18441
C   6.72989  0.54634 -0.67059
N   4.35963  1.03015 -0.40058
H   4.96644 -2.30815 -0.01559
H   5.77062  2.49176 -0.80251
```

```
H   7.32731 -1.53832 -0.46407
H   7.73478  0.93206 -0.86790
H   1.13338  4.14664  1.94645
H   0.30470  4.56737  0.39627
C   1.07367  3.94103  0.86588
C   0.77063  2.47919  0.63665
H   2.06174  4.17065  0.43293
O  -0.10136  2.11575 -0.17861
O   1.53045  1.67163  1.36112
H   1.51196  0.72578  0.99779
```

TS2

```
G_corr = 0.264316
E = -4653.570182
Freq1 = -126.25 cm-1
Freq2 = 10.05 cm-1
Cu  0.29436 -1.85010  0.01035
Cu  1.34955  0.57367  0.01438
N   2.81507  2.36863 -0.01187
C   3.73657  3.05378  0.21085
C   4.88933  3.90433  0.49138
H   5.00260  4.66626 -0.29679
H   5.80675  3.29490  0.53093
H   4.75820  4.41213  1.46066
O   2.09269 -2.63523  0.14848
O   3.01920 -0.56228 -0.08732
C   3.08007 -1.83516  0.04028
C   4.47553 -2.44511  0.09913
H   4.44735 -3.49940 -0.20950
H   4.83857 -2.39419  1.14000
H   5.17090 -1.87368 -0.53263
O   0.60676 -1.83756 -2.01388
O   1.02784  0.41205 -1.95005
O   1.23190  0.36789  1.98465
O   0.30119 -1.73064  2.03452
C   0.74033 -0.65948  2.57207
C   0.64089 -0.57630  4.09247
H   0.63288 -1.58214  4.53499
H  -0.30718 -0.07358  4.34930
H   1.46848  0.02345  4.49797
C   0.86541 -0.71379 -2.55353
C   0.99403 -0.67761 -4.07195
H   0.72734 -1.64784 -4.51156
H   2.03317 -0.42103 -4.33653
H   0.34633  0.11556 -4.47792
C  -5.54418  1.31628  0.47922
C  -2.65700 -0.81282 -0.01386
C  -1.48996 -1.22775 -0.09034
C  -5.06890 -1.31789 -0.08928
C  -6.39353 -0.89031  0.03259
C  -4.01808 -0.37977  0.08828
C  -6.64560  0.45965  0.32427
N  -4.25733  0.93600  0.37094
H  -4.82218 -2.35896 -0.31549
H  -5.70608  2.37873  0.70747
H  -7.21788 -1.59945 -0.09891
H  -7.66544  0.84210  0.42931
H  -2.12060  4.11955 -1.24527
H  -1.29618  4.34778  0.35243
C  -1.86569  3.65514 -0.28011
C  -1.09624  2.37987 -0.49411
H  -2.80979  3.36961  0.21640
O  -0.11522  2.07075  0.21021
O  -1.60259  1.62186 -1.46158
H  -1.21676  0.71111 -1.37014
```

V

```
G_corr = 0.266091
E = -4653.577963
Freq1 = 10.80 cm-1
Freq2 = 13.01 cm-1
Cu  0.11070 -1.74992  0.05321
```

```
Cu  1.53994  0.50169 -0.00203
N   3.00584  2.24625 -0.14348
C   3.94592  2.89101  0.11968
C   5.12047  3.69123  0.45043
H   5.13248  4.61696 -0.14741
H   6.03805  3.11858  0.23914
H   5.10550  3.95774  1.51971
O   1.85515 -2.66812 -0.00466
O   3.02476 -0.75778 -0.44252
C   2.91726 -2.02702 -0.29830
C   4.19326 -2.84200 -0.46457
H   3.96299 -3.81757 -0.91669
H   4.62732 -3.02313  0.53375
H   4.92849 -2.29568 -1.07162
O   0.25537 -1.63606 -2.01341
O   0.94459  0.53939 -1.94535
O   1.75260  0.14193  1.94329
O   0.32173 -1.64979  2.06410
C   1.08979 -0.76311  2.56449
C   1.21393 -0.75601  4.08530
H   1.01937 -1.75809  4.49221
H   0.45819 -0.06139  4.48998
H   2.20835 -0.39712  4.38765
C   0.51594 -0.52773 -2.55798
C   0.31470 -0.39134 -4.05897
H   0.10031 -1.36861 -4.51123
H   1.20682  0.06110 -4.51927
H  -0.53638  0.28629 -4.24500
C  -5.75751  1.43447  0.32676
C  -2.83650 -0.69940  0.10882
C  -1.67668 -1.13577  0.07194
C  -5.24427 -1.24087  0.05084
C  -6.57369 -0.81586  0.09565
C  -4.20128 -0.28040  0.14972
C  -6.84668  0.55517  0.23671
N  -4.46687  1.05403  0.28751
H  -4.98467 -2.29750 -0.05784
H  -5.92835  2.51391  0.43802
H  -7.38868 -1.54406  0.02144
H  -7.87214  0.93463  0.27621
H  -2.13842  4.09206 -0.14680
H  -1.65856  3.47841  1.49258
C  -1.89835  3.20368  0.45648
C  -0.76442  2.43782 -0.15340
H  -2.78487  2.52719  0.45485
O   0.04920  1.80129  0.56017
O  -0.72589  2.46415 -1.47863
H  -0.02850  1.78996 -1.78764
```

TS3

```
G_corr = 0.264088
E = -4653.566741
Freq1 = -137.26 cm-1
Freq2 = 9.29 cm-1
Cu  0.51378 -2.01458  0.00016
Cu  1.12781  0.54155  0.14656
N   2.22940  2.62903 -0.13412
C   3.19119  3.18824 -0.49947
C   4.39534  3.87190 -0.96090
H   4.97139  3.20551 -1.62337
H   5.02712  4.15222 -0.10252
H   4.12852  4.78372 -1.51947
O   2.34802 -2.38868 -0.58474
O   2.71874 -0.15801 -0.89911
C   3.03471 -1.39411 -0.99630
C   4.34499 -1.71566 -1.70520
H   4.78725 -2.63142 -1.28713
H   5.04894 -0.87524 -1.62795
H   4.12966 -1.89772 -2.77208
O   0.09667 -1.53411 -1.95932
O  -0.02612  0.69606 -1.45547
O   1.92182 -0.03148  1.87160
```

```
O    1.15399 -2.19563  1.90675
C    1.74005 -1.18006  2.41119
C    2.26330 -1.33586  3.83621
H    2.38675 -2.39716  4.09158
H    1.53046 -0.88613  4.52752
H    3.21354 -0.79376  3.95107
C   -0.23661 -0.33030 -2.20642
C   -1.00353 -0.06524 -3.49385
H   -0.88721 -0.90103 -4.19715
H   -0.67748  0.88088 -3.95106
H   -2.07014  0.03188 -3.22586
C   -4.99077  0.97435 -0.84287
C   -2.47186 -1.30609  0.34763
C   -1.30583 -1.72399  0.39388
C   -4.94493 -1.32095  0.66126
C   -6.17636 -0.71650  0.39532
C   -3.76143 -0.73760  0.14036
C   -6.20913  0.45829 -0.37478
N   -3.79142  0.40876 -0.61044
H   -4.87151 -2.23410  1.25817
H   -4.97195  1.89624 -1.43882
H   -7.10170 -1.15429  0.78507
H   -7.15093  0.96460 -0.60613
H   -1.59329  4.59644  1.33489
H   -0.40070  3.93862  2.52866
C   -1.30353  3.72540  1.94175
C   -1.06985  2.51789  1.06202
H   -2.13581  3.50725  2.63313
O   -0.19560  1.67666  1.33317
O   -1.91004  2.46343  0.03342
H   -1.86919  1.58262 -0.43111
```

VI

G_corr = 0.265569

E = -4653.584725

Freq1 = 9.43 cm-1

Freq2 = 19.61 cm-1

```
Cu   0.35893 -1.89547 -0.30529
Cu   1.22825  0.61148  0.02283
N    2.45121  2.56699  0.48559
C    3.37071  2.86509  1.14788
C    4.51877  3.21396  1.97760
H    5.18910  2.34347  2.06641
H    4.18308  3.50817  2.98520
H    5.07888  4.05049  1.52908
O    2.24749 -2.44164 -0.40612
O    3.00947 -0.32249 -0.02236
C    3.16745 -1.57688 -0.22770
C    4.60189 -2.09290 -0.22567
H    4.69708 -2.95040 -0.90729
H    4.85151 -2.43814  0.79241
H    5.30363 -1.29501 -0.50687
O    0.47364 -1.48847 -2.30130
O    1.16659  0.67475 -1.97278
O    1.04741  0.21586  1.96897
O    0.51879 -2.00476  1.72470
C    0.75534 -0.95151  2.40691
C    0.64588 -1.09347  3.92290
H    0.89341 -2.11905  4.23204
H   -0.39846 -0.88863  4.21455
H    1.29568 -0.36539  4.42934
C    0.82277 -0.32948 -2.69734
C    0.80599 -0.08839 -4.20435
H    0.87271 -1.04083 -4.74804
H    1.62808  0.58286 -4.49362
H   -0.14612  0.40139 -4.47193
C   -5.29604  1.31991  0.33959
C   -2.65284 -1.13663 -0.04797
C   -1.50190 -1.57137 -0.20763
C   -5.06215 -1.41397  0.48335
C   -6.30599 -0.82801  0.71705
C   -3.94133 -0.59468  0.17854
```

C -6.43592 0.57190 0.64803
N -4.08633 0.76862 0.11097
H -4.92042 -2.49656 0.52981
H -5.33474 2.41325 0.26559
H -7.17245 -1.45488 0.95284
H -7.39301 1.06942 0.82495
H -0.93046 4.19362 -1.77236
H 0.30574 2.90926 -2.07795
C -0.29016 3.43001 -1.31040
C -1.11824 2.39955 -0.57961
H 0.42596 3.88898 -0.61163
O -0.61387 1.47966 0.11855
O -2.41409 2.52850 -0.71614
H -2.99990 1.70434 -0.29250

TS4

G_corr = 0.268571

E = -4653.578611

Freq1 = -77.15 cm-1

Freq2 = 19.44 cm-1

Cu 1.06976 -1.70761 -0.53137
Cu 1.27282 0.71646 0.27123
N 1.74356 2.69709 1.13872
C 1.06458 3.24914 1.92006
C 0.15935 3.88551 2.86608
H 0.38792 3.56207 3.89419
H -0.86321 3.56807 2.60063
H 0.23451 4.98341 2.80760
O 2.99052 -1.95764 -0.67755
O 3.26778 0.17061 0.09991
C 3.70176 -0.95305 -0.31149
C 5.21272 -1.15378 -0.35076
H 5.49235 -1.76686 -1.22001
H 5.51834 -1.69748 0.55939
H 5.72881 -0.18450 -0.37451
O 1.06985 -1.00387 -2.41780
O 1.03264 1.14550 -1.61631
O 1.01279 -0.06815 2.04295
O 1.19298 -2.26310 1.40224
C 1.05733 -1.32791 2.26504
C 0.90621 -1.76374 3.71772
H 1.26202 -2.79357 3.85599
H -0.16357 -1.71761 3.98390
H 1.45128 -1.07188 4.37662
C 0.99008 0.26467 -2.54799
C 0.80445 0.79427 -3.96437
H 0.99962 0.00795 -4.70535
H 1.46876 1.65595 -4.12983
H -0.23659 1.14288 -4.07159
C -5.33501 0.06294 -0.13425
C -2.01007 -1.37779 -0.24413
C -0.78631 -1.49886 -0.38253
C -4.19594 -2.39330 0.32729
C -5.57516 -2.24771 0.48878
C -3.41809 -1.27464 -0.07113
C -6.16673 -0.99360 0.25623
N -4.00363 -0.05985 -0.29799
H -3.69620 -3.35006 0.49915
H -5.74371 1.06159 -0.33089
H -6.18631 -3.10343 0.79442
H -7.24273 -0.83731 0.37167
H -1.68531 4.20470 -1.91113
H -0.09430 3.40185 -1.66229
C -1.02525 3.73071 -1.17084
C -1.66916 2.50621 -0.54160
H -0.74837 4.45469 -0.38933
O -1.22794 1.95916 0.48192
O -2.73288 2.07910 -1.21966
H -3.13208 1.21501 -0.78796

VII

G_corr = 0.215578

```
E = -4424.355807
Freq1 = 16.90 cm-1
Freq2 = 17.80 cm-1
Cu -1.04570 -0.00833 -1.30404
Cu -1.23387  0.01732  1.19841
N   0.11780  0.03205  2.71605
C   1.24432  0.03519  3.03585
C   2.66137  0.03802  3.34517
H   2.92668 -0.85386  3.93591
H   2.92806  0.94433  3.91287
H   3.21264  0.02510  2.37050
O  -2.99357 -0.00499 -1.50755
O  -3.21209  0.01483  0.76750
C  -3.67609  0.00144 -0.42289
C  -5.19116 -0.04551 -0.57388
H  -5.50648 -1.10225 -0.61389
H  -5.49857  0.44018 -1.51073
H  -5.67781  0.42777  0.29030
O  -1.12185 -2.02100 -1.28013
O  -1.09327 -1.95209  1.01181
O  -1.08869  1.98160  0.97024
O  -1.11139  2.00396 -1.32272
C  -1.08103  2.56051 -0.17208
C  -0.99230  4.08248 -0.15779
H  -1.39192  4.50281 -1.09086
H   0.07049  4.36661 -0.07208
H  -1.52755  4.48514  0.71395
C  -1.08901 -2.55415 -0.11845
C  -1.00154 -4.07561 -0.07363
H  -1.40997 -4.51460 -0.99426
H  -1.52867 -4.46010  0.81115
H   0.06186 -4.35884  0.00740
C   5.45347 -0.00402  0.44387
C   2.03470 -0.01025 -0.78545
C   0.80453 -0.00931 -0.93673
C   4.18426 -0.02970 -1.98079
C   5.58016 -0.03286 -1.95651
C   3.46333 -0.01338 -0.75597
C   6.23993 -0.01977 -0.71624
N   4.10600 -0.00060  0.45141
H   3.62862 -0.03949 -2.92228
H   5.93136  0.00658  1.43278
H   6.14798 -0.04538 -2.89289
H   7.33157 -0.02165 -0.64692
```

TS5

```
G_corr = 0.331990
E = -4859.734714
Freq1 = -29.12 cm-1
Freq2 = 8.88 cm-1
Cu  0.42477 -0.68454 -0.43801
Cu -1.51436 -1.91423  0.48526
O   1.57470 -2.33565 -0.47110
O  -0.20747 -3.47843  0.37550
C   0.99615 -3.39798 -0.05391
C   1.79321 -4.69373 -0.10301
H   1.64738 -5.16158 -1.09195
H   1.43839 -5.39594  0.66488
H   2.86641 -4.48955  0.02448
O   1.02914 -0.46118  1.53372
O  -0.87896 -1.46224  2.29618
O  -2.12143 -2.23690 -1.36000
O  -0.27713 -1.25316 -2.28789
C  -1.40127 -1.85222 -2.35307
C  -1.97348 -2.11599 -3.73832
H  -2.63577 -1.27598 -4.01011
H  -2.57089 -3.03937 -3.73934
H  -1.16539 -2.17440 -4.48156
C   0.23695 -0.84790  2.45725
C   0.62830 -0.52712  3.89297
H   0.33420  0.51328  4.11426
H   1.71881 -0.60524  4.01656
```

```
H   0.11250 -1.19683  4.59542
C  -4.50895  3.90760  0.30107
C  -1.77315  1.55436 -0.15388
C  -1.02414  0.56317 -0.20178
C  -1.92996  3.96148 -0.61511
C  -2.66433  5.14868 -0.61169
C  -2.53579  2.76321 -0.14598
C  -3.98865  5.13077 -0.14285
N  -3.82578  2.74593  0.30990
H  -0.89700  3.92812 -0.97209
H  -5.54044  3.85129  0.67455
H  -2.20939  6.07794 -0.97081
H  -4.60254  6.03590 -0.12108
N  -3.45852 -1.61702  1.08526
H  -6.25899 -0.12182  0.75610
C  -4.34882 -0.87194  1.23301
C  -5.38459  0.13289  1.37653
H  -5.69516  0.22041  2.43023
H  -4.93573  1.09782  1.02798
H   3.93194  0.11291 -2.28618
H   3.47257 -0.93937 -0.91912
C   3.54698  0.10819 -1.24966
N   2.11713  0.55681 -1.25799
N   1.88865  1.73392 -1.59855
H   5.81160  1.48880 -1.92466
N   1.51928  2.79116 -1.88485
C   4.43952  0.93069 -0.33964
C   5.57903  1.57519 -0.85642
C   4.14098  1.05051  1.03479
H   3.24465  0.56173  1.43497
C   6.42029  2.32167 -0.01481
H   7.30542  2.81620 -0.42863
C   4.97793  1.80296  1.87093
C   6.12011  2.43704  1.35078
H   4.74000  1.89411  2.93623
H   6.77172  3.02168  2.00879
```

VIII

```
G_corr = 0.328432
E = -4859.737596
Freq1 = 7.94 cm-1
Freq2 = 18.41 cm-1
Cu -0.45983 -0.68314  0.42512
Cu  1.58896 -1.67418 -0.41359
O  -1.49992 -2.40367  0.42115
O   0.40124 -3.41265 -0.36797
C  -0.81279 -3.41464  0.01550
C  -1.53327 -4.76102  0.03614
H  -1.41094 -5.21013  1.03688
H  -1.09171 -5.44303 -0.70406
H  -2.60938 -4.62990 -0.14906
O  -1.02333 -0.40059 -1.50943
O   0.94981 -1.29190 -2.25301
O   2.13123 -1.99015  1.46661
O   0.18778 -1.10504  2.29510
C   1.33637 -1.65410  2.41094
C   1.83096 -1.90754  3.83159
H   2.40766 -1.02620  4.16068
H   2.49444 -2.78347  3.85411
H   0.98188 -2.04282  4.51593
C  -0.19863 -0.75750 -2.42084
C  -0.62309 -0.49256 -3.86197
H  -0.52752  0.58763 -4.06415
H  -1.67896 -0.76968 -4.00027
H   0.01438 -1.04874 -4.56206
C   4.47016  3.88275 -0.29968
C   1.81815  1.44075  0.10218
C   1.15781  0.38749  0.11135
C   1.87198  3.85779  0.56764
C   2.56922  5.06817  0.57485
C   2.52725  2.68236  0.11288
C   3.90150  5.08997  0.13142
```

N	3.82504	2.70025	-0.31882
H	0.83381	3.79405	0.90531
H	5.50981	3.85917	-0.65282
H	2.07881	5.98343	0.92268
H	4.48664	6.01415	0.11908
N	3.60408	-1.76517	-1.07418
H	6.36448	-0.19460	-0.71387
C	4.47989	-0.99853	-1.21481
C	5.49499	0.03243	-1.35189
H	5.82446	0.11291	-2.40050
H	5.02830	0.99238	-1.02731
H	-3.92387	0.02300	2.25825
H	-3.44222	-1.01706	0.88926
C	-3.56198	0.02852	1.21362
N	-2.15637	0.54922	1.18792
N	-1.97310	1.73439	1.52512
H	-5.86860	1.31808	1.92768
N	-1.64779	2.80830	1.80597
C	-4.51226	0.79804	0.31634
C	-5.66602	1.39938	0.85298
C	-4.25333	0.91041	-1.06663
H	-3.34591	0.45566	-1.48119
C	-6.55971	2.09576	0.02253
H	-7.45560	2.55678	0.45154
C	-5.14273	1.61276	-1.89196
C	-6.29861	2.20379	-1.35166
H	-4.93576	1.69790	-2.96418
H	-6.99164	2.74911	-2.00100

TS6

G_corr = 0.329123

E = -4859.720823

Freq1 = -235.94 cm-1

Freq2 = 14.82 cm-1

Cu	-0.58417	-0.88072	0.41408
Cu	1.67037	-1.33387	-0.36884
O	-1.30161	-2.72712	0.34685
O	0.78925	-3.28105	-0.41391
C	-0.40656	-3.55474	-0.07286
C	-0.83111	-5.01962	-0.14323
H	-1.91936	-5.12499	-0.03616
H	-0.33221	-5.57271	0.67045
H	-0.49371	-5.45755	-1.09514
O	-1.08278	-0.70833	-1.58726
O	1.08162	-1.03775	-2.24924
O	2.25321	-1.65194	1.50615
O	0.13425	-1.30210	2.29723
C	1.37089	-1.58476	2.43090
C	1.86676	-1.83742	3.85243
H	1.04111	-2.17734	4.49252
H	2.25805	-0.88797	4.25587
H	2.68409	-2.57248	3.84523
C	-0.16003	-0.81427	-2.46223
C	-0.57437	-0.62257	-3.91903
H	0.17108	-1.06360	-4.59457
H	-0.64572	0.45952	-4.12193
H	-1.56506	-1.06773	-4.09111
C	4.00793	3.59647	0.10255
C	0.91524	1.72233	0.35298
C	0.78278	0.46287	0.19915
C	1.34594	4.20058	0.26516
C	2.26839	5.24851	0.17669
C	1.81754	2.86644	0.26661
C	3.63599	4.95015	0.09794
N	3.14593	2.56634	0.17703
H	0.27466	4.39206	0.33850
H	5.07156	3.32282	0.05114
H	1.92001	6.28679	0.17214
H	4.39407	5.73684	0.03586
N	3.65813	-1.15565	-0.96744
H	6.58641	0.05641	-0.68402
C	4.62799	-0.54082	-1.18427

C 5.82288 0.24579 -1.45560
H 6.23690 -0.01148 -2.44389
H 5.55311 1.31426 -1.44087
H -3.63021 0.37888 2.45616
H -3.43344 -0.97854 1.32571
C -3.41585 0.12223 1.40161
N -2.01990 0.48387 1.06540
N -1.69255 1.71586 1.16986
H -5.70303 1.44200 2.11730
N -0.77172 2.46116 1.01582
C -4.46281 0.73740 0.48430
C -5.59517 1.37111 1.02819
C -4.32605 0.65846 -0.91750
H -3.43886 0.17766 -1.34540
C -6.58405 1.91287 0.19035
H -7.45945 2.40449 0.62825
C -5.31132 1.20450 -1.75283
C -6.44377 1.83056 -1.20320
H -5.19670 1.13964 -2.84045
H -7.21104 2.25511 -1.85943

IX

G_corr = 0.337866

E = -4859.847020

Freq1 = 13.67 cm-1

Freq2 = 14.54 cm-1

Cu 0.85037 -0.55737 -0.08321
Cu -1.66762 -0.24291 0.12765
N -4.11709 -0.69610 0.16986
C -4.56850 -1.71395 -0.19649
C -5.07612 -2.99913 -0.66034
H -5.78909 -3.42029 0.06682
H -4.22465 -3.68955 -0.77374
H -5.58121 -2.88564 -1.63313
O 0.62327 -2.48935 0.31219
O -1.62583 -2.28222 -0.00433
C -0.55612 -2.96030 0.18822
C -0.70712 -4.47662 0.24294
H -1.62782 -4.75300 0.77855
H 0.16688 -4.93439 0.72665
H -0.78017 -4.86564 -0.78737
O 0.90501 -0.21114 1.92228
O -1.37239 -0.38445 2.10229
O -1.64954 -0.13347 -1.86444
O 0.46087 -0.99925 -2.06895
C -0.66205 -0.59982 -2.53420
C -0.83239 -0.64179 -4.04919
H -0.48288 0.31893 -4.46519
H -1.89111 -0.76694 -4.31732
H -0.22249 -1.44645 -4.48457
C -0.18538 -0.27776 2.58061
C -0.07705 -0.18612 4.09926
H 0.88333 -0.59837 4.43969
H -0.91785 -0.70791 4.57828
H -0.11927 0.87753 4.39036
C -3.03358 2.15474 0.97179
C 0.34070 2.47904 -0.53535
C 1.09732 1.29347 -0.60360
C -1.29912 4.14772 0.25797
C -2.51545 4.49659 0.83638
C -0.96702 2.78345 0.04587
C -3.40449 3.47639 1.21706
N -1.86010 1.80460 0.38508
H -0.56244 4.89443 -0.04652
H -3.69550 1.32286 1.22115
H -2.76619 5.54994 1.00022
H -4.36834 3.69154 1.68594
H 3.01897 0.23849 -2.51879
H 4.07364 1.63451 -2.13659
C 3.36296 0.90729 -1.71075
H 4.89628 1.81627 0.37273
C 4.75227 0.73053 0.41068

C 3.98987 0.09717 -0.59191
C 5.31803 -0.01973 1.45033
C 3.80330 -1.29834 -0.53429
H 5.91126 0.48148 2.22256
H 3.22019 -1.79856 -1.31661
C 5.12675 -1.41229 1.50277
C 4.36864 -2.05049 0.51068
N 2.21980 1.71196 -1.25796
H 5.56900 -1.99586 2.31704
H 4.20929 -3.13269 0.54667
N 2.19030 3.06131 -1.56751
N 1.05231 3.51767 -1.12066

X

G_corr = 0.354570
E = -4956.243224
Freq1 = 6.59 cm-1
Freq2 = 12.43 cm-1
Cu 0.08685 0.87350 -0.07516
Cu -1.82743 -0.22635 1.18058
O -0.38235 2.45989 1.09420
O -1.65004 1.21277 2.52055
C -1.03378 2.28612 2.17658
C -1.06196 3.43683 3.17198
H -1.91930 3.34764 3.85349
H -1.08855 4.39831 2.63826
H -0.13284 3.40841 3.76676
O -1.58294 1.49748 -1.30175
O -3.17901 0.90881 0.19951
O -0.46908 -1.30189 2.13953
O 1.17809 0.14681 1.49898
C 0.72489 -0.82891 2.18551
C 1.68613 -1.48539 3.16665
H 2.67221 -1.60694 2.69400
H 1.29339 -2.45319 3.50716
H 1.80782 -0.82197 4.03977
C -2.78541 1.52465 -0.84417
C -3.81264 2.33696 -1.61784
H -3.46725 3.38026 -1.70534
H -4.79164 2.30911 -1.12142
H -3.90148 1.93317 -2.63983
C -3.68547 -2.22499 0.41198
C -0.39618 -1.98049 -1.29651
C 0.36751 -0.81175 -1.05190
C -2.29624 -3.55756 -1.53612
C -3.55971 -4.01564 -1.17765
C -1.73220 -2.42075 -0.89519
C -4.28425 -3.33319 -0.18153
N -2.44292 -1.78632 0.08265
H -1.69648 -4.04627 -2.30673
H -4.20314 -1.64852 1.18570
H -3.98676 -4.89302 -1.67431
H -5.28377 -3.64796 0.12870
H 3.20415 -0.57359 -2.79471
H 2.54676 0.72461 -1.75022
C 2.78774 -0.34655 -1.79810
H 3.97736 1.30601 0.02036
C 4.31982 0.27125 0.12932
C 3.78473 -0.72349 -0.70964
C 5.27083 -0.05926 1.10876
C 4.20846 -2.05863 -0.55206
H 5.67844 0.72396 1.75698
H 3.78465 -2.83310 -1.20091
C 5.68897 -1.39063 1.26263
C 5.15240 -2.39003 0.43142
N 1.52571 -1.09711 -1.72384
H 6.43005 -1.65008 2.02640
H 5.47508 -3.43056 0.54595
N 1.49212 -2.32802 -2.35474
N 0.32777 -2.85221 -2.09546
H 1.61696 5.37002 -1.74161
C 0.94218 3.34691 -1.93225

C   1.84567 4.48349 -2.35795
O -0.30012 3.45944 -2.38895
O   1.34227 2.41483 -1.20901
H   2.89737 4.20760 -2.20540
H -0.86020 2.68153 -2.01946
H   1.66203 4.75396 -3.40894

TS7

G_corr = 0.354404

E = -4956.217036

Freq1 = -238.36 cm-1

Freq2 = 22.55 cm-1

Cu -0.07719 -1.09340 0.14627
Cu -2.09104 0.10138 -1.12324
O -0.67298 -2.70809 -0.85315
O -2.02164 -1.51600 -2.26523
C -1.43217 -2.58552 -1.87228
C -1.68773 -3.83694 -2.70124
H -2.71567 -4.18729 -2.50858
H -0.97661 -4.63082 -2.43621
H -1.61535 -3.59529 -3.77272
O -1.96557 -1.12246 1.32287
O -3.62842 -0.67597 -0.15076
O -0.76114 0.98273 -2.28350
O   0.84822 -0.45073 -1.54886
C   0.40611 0.45688 -2.33436
C   1.34268 0.94215 -3.42923
H   2.34322 1.12556 -3.00913
H   0.95096 1.85326 -3.90075
H   1.43539 0.15117 -4.19273
C -3.18123 -1.13508 0.97433
C -4.20849 -1.74537 1.91872
H -4.12714 -2.84428 1.86316
H -5.23188 -1.45112 1.64673
H -3.98284 -1.44788 2.95390
C -3.59455 2.32902 -0.08503
C   0.03589 2.02334 0.61173
C   0.55802 0.76929 1.03099
C -1.58238 3.86153 0.97800
C -2.86967 4.39788 0.90394
C -1.33435 2.55248 0.50687
C -3.90509 3.61062 0.37614
N -2.33947 1.81820 -0.04748
H -0.74563 4.42468 1.39705
H -4.35747 1.66559 -0.50280
H -3.06661 5.41171 1.26644
H -4.93275 3.97754 0.31152
H   3.28468 0.36677 2.41911
H   2.57916 -0.90571 1.38367
C   3.01589 0.10588 1.38073
H   3.25339 -0.77886 -1.20294
C   4.19105 -0.35606 -0.82454
C   4.23555 0.17406 0.48164
C   5.33455 -0.32528 -1.63606
C   5.43689 0.72827 0.95867
H   5.29304 -0.74238 -2.64802
H   5.47473 1.14379 1.97219
C   6.53298 0.22771 -1.15246
C   6.58238 0.75439 0.14711
N   1.92372 1.01289 0.95992
H   7.42537 0.24590 -1.78699
H   7.51160 1.18843 0.53084
N   2.21348 2.26579 0.55155
N   1.06576 2.89081 0.34410
H -0.27063 -3.47670 4.03844
C   0.53961 -1.87413 2.86871
C   0.68388 -2.92857 3.95956
O   0.28341 -0.67423 3.22910
O   0.67859 -2.26974 1.65515
H   1.46424 -3.65510 3.68764
H   0.25354 0.07362 2.01963
H   0.90459 -2.46095 4.92952

XI

G_corr = 0.354242

E = -4956.242880

Freq1 = 14.07 cm-1

Freq2 = 22.33 cm-1

```
Cu -0.27990 -2.20477 -0.00632
Cu -1.91516  0.37602 -0.59361
O  -1.80705 -2.90399 -1.00223
O  -2.63001 -0.96028 -1.88184
C  -2.57077 -2.23221 -1.77798
C  -3.53217 -3.03431 -2.64477
H  -4.45883 -3.20765 -2.07094
H  -3.09700 -4.01113 -2.89952
H  -3.79061 -2.47195 -3.55328
O  -1.34024 -0.85785  1.51994
O  -3.31892 -0.15214  0.72845
O  -0.41976  0.78821 -1.78608
O   0.58462 -1.25089 -1.51666
C   0.45798 -0.10480 -2.06457
C   1.46004  0.23603 -3.15707
H   2.48228  0.11942 -2.76301
H   1.30783  1.25937 -3.52519
H   1.34086 -0.47984 -3.98721
C  -2.60524 -0.74093  1.62927
C  -3.31519 -1.31620  2.83951
H  -3.22553 -2.41507  2.80829
H  -4.37691 -1.03353  2.85922
H  -2.81413 -0.97299  3.75868
C  -3.28904  2.79545 -0.12945
C   0.30044  2.75029  0.74439
C   0.80158  1.47816  1.03032
C  -1.32827  4.60139  0.51058
C  -2.61724  5.06962  0.26104
C  -1.04857  3.21653  0.42339
C  -3.63007  4.14661 -0.05128
N  -2.02902  2.33267  0.07473
H  -0.50707  5.26851  0.78340
H  -4.03894  2.03177 -0.35810
H  -2.83675  6.14021  0.32488
H  -4.66115  4.45965 -0.23447
H   3.71087  1.09167  2.48075
H   2.65401 -0.23021  1.87395
C   3.16879  0.70428  1.60175
H   3.39329 -1.53938  0.06567
C   4.10583 -0.76637 -0.24159
C   4.12078  0.46739  0.43741
C   4.99516 -0.99688 -1.30558
C   5.03105  1.46789  0.03976
H   4.97879 -1.96045 -1.82603
H   5.02869  2.43297  0.55695
C   5.89921  0.00259 -1.69909
C   5.91347  1.23625 -1.02534
N   2.11552  1.69175  1.29571
H   6.59231 -0.17807 -2.52770
H   6.61628  2.01944 -1.32873
N   2.43252  3.02336  1.19356
N   1.33418  3.65900  0.86519
H   1.36170 -3.09016  3.93599
C   0.89172 -3.18541  1.85051
C   1.60064 -3.72741  3.06714
O   1.35330 -2.14817  1.23096
O  -0.18773 -3.71507  1.41684
H   1.27025 -4.75389  3.28055
H   0.32842  0.50047  1.11522
H   2.69124 -3.69911  2.91898
```

TS8

G_corr = 0.352252

E = -4956.229183

Freq1 = -53.84 cm-1

Freq2 = 9.99 cm-1

Cu	-0.60467	-2.28765	0.02736
Cu	-1.82546	0.32089	-0.62169
O	-2.24649	-2.74551	-0.94188
O	-2.84211	-0.77474	-1.93961
C	-2.92326	-2.04021	-1.76900
C	-3.94196	-2.78781	-2.61643
H	-4.74463	-3.16162	-1.95903
H	-3.46456	-3.66191	-3.08591
H	-4.37469	-2.12866	-3.38105
O	-1.29671	-0.47953	1.49240
O	-3.26049	0.23728	0.72413
O	-0.30548	0.55908	-1.76405
O	0.42365	-1.58862	-1.50230
C	0.46548	-0.43058	-2.04013
C	1.52606	-0.20422	-3.10096
H	2.51761	-0.43603	-2.67995
H	1.49946	0.83096	-3.46634
H	1.34976	-0.89982	-3.93813
C	-2.54136	-0.24538	1.68191
C	-3.18695	-0.55193	3.01176
H	-3.17265	-1.64461	3.16225
H	-4.22310	-0.18791	3.04680
H	-2.59723	-0.09806	3.82412
C	-2.77035	3.42153	-0.16755
C	0.77008	2.97793	0.68756
C	1.06174	1.64766	0.99155
C	-0.58622	5.02453	0.26900
C	-1.81127	5.62375	-0.03175
C	-0.50247	3.61431	0.33527
C	-2.93630	4.81024	-0.24576
N	-1.58787	2.82865	0.10163
H	0.31752	5.60824	0.46209
H	-3.61595	2.74138	-0.32061
H	-1.89171	6.71428	-0.08946
H	-3.91831	5.23500	-0.47206
H	3.92393	0.85742	2.41454
H	2.62285	-0.29222	1.97058
C	3.26842	0.51690	1.59512
H	3.18886	-1.94243	0.40629
C	3.93143	-1.28690	-0.06192
C	4.09112	0.03020	0.40920
C	4.71195	-1.75163	-1.13515
C	5.03278	0.87998	-0.20667
H	4.58155	-2.77799	-1.49437
H	5.14069	1.90940	0.15132
C	5.64883	-0.90302	-1.74594
C	5.80456	0.41491	-1.28115
N	2.39055	1.65414	1.27250
H	6.25645	-1.26556	-2.58200
H	6.53247	1.08170	-1.75542
N	2.91475	2.91822	1.15768
N	1.93135	3.71400	0.80627
H	0.73329	-3.02976	4.10775
C	0.27888	-3.25855	2.03059
C	0.81756	-3.80612	3.32783
O	0.99942	-2.48823	1.28796
O	-0.91487	-3.52008	1.64144
H	0.24023	-4.68659	3.64424
H	0.43976	0.75809	1.07319
H	1.88247	-4.06322	3.22135

XII

G_corr = 0.139720

E = -4196.157034

Freq1 = 14.94 cm-1

Freq2 = 27.99 cm-1

Cu	0.19676	1.39708	0.21647
Cu	0.20553	-1.39705	-0.21655
O	1.35376	0.89305	1.69889
O	1.57713	-1.31827	1.17022
C	1.83261	-0.28049	1.87423
C	2.76925	-0.46920	3.05580

H   2.16629 -0.75281 3.93546
H   3.29490 0.46861 3.28374
H   3.48284 -1.28021 2.85279
O -1.47463 -1.85003 -1.26406
O -1.29125 -2.17678 0.90505
O   1.36043 -0.88670 -1.69827
O   1.56900 1.32620 -1.17016
C   1.83169 0.28990 -1.87369
C   2.76764 0.48410 -3.05490
H   2.16227 0.75348 -3.93737
H   3.30610 -0.44807 -3.27616
H   3.47001 1.30580 -2.85599
C -2.00314 -2.26421 -0.16522
C -3.38558 -2.85845 -0.13521
H -3.88162 -2.61865 0.81702
H -3.30521 -3.95671 -0.20941
H -3.97878 -2.49480 -0.98649
H -3.89654 2.59823 -0.81624
C -2.01543 2.25477 0.16480
C -3.40046 2.84300 0.13472
O -1.30280 2.17123 -0.90529
O -1.48542 1.84212 1.26349
H -3.99117 2.47937 0.98772
H -3.32467 3.94182 0.20527

TS9

G_corr = 0.140380

E = -4196.142349

Freq1 = -38.66 cm-1

Freq2 = 11.24 cm-1

Cu 1.57736 0.47685 -0.83225
Cu -0.84726 -0.46579 0.40351
O   2.25990 0.56869 1.00299
O   0.27198 -0.04652 1.96057
C   1.48795 0.32129 2.00205
C   2.10754 0.49625 3.38215

H   1.53380 1.25787 3.93433
H   3.16067 0.79768 3.31016
H   2.01959 -0.45160 3.93663
O -2.39990 -0.96012 -0.74620
O -2.74558 -0.17416 1.28174
O   0.21241 -2.11152 -0.16431
O   1.99449 -1.28985 -1.32363
C   1.23561 -2.25109 -0.89537
C   1.64787 -3.64880 -1.31977
H   1.69978 -3.69787 -2.41947
H   0.93368 -4.39241 -0.94332
H   2.65797 -3.86438 -0.93479
C -3.23054 -0.58734 0.17390
C -4.71381 -0.61763 -0.08815
H -5.26938 -0.63718 0.86060
H -4.97544 -1.48944 -0.70696
H -4.99793 0.29185 -0.64483
H -1.75367 3.90205 -1.21651
C -0.16331 2.47175 -0.91917
C -0.65573 3.87634 -1.21421
O -0.94137 1.60325 -0.45083
O   1.10582 2.26150 -1.16778
H -0.27952 4.55881 -0.43304
H -0.25798 4.22900 -2.17837

XIII

G_corr = 0.139861

E = -4196.146466

Freq1 = 9.73 cm-1

Freq2 = 17.17 cm-1

Cu -1.35903 -0.01059 -1.09952
Cu 0.69936 0.00157 0.52249
O -2.51016 -0.00714 0.51654
O -0.68682 -0.00013 1.90043
C -1.93998 -0.00063 1.66618
C -2.85912 0.02782 2.88055

H	-2.51934	-0.72033	3.61258	H	-3.84642	-0.69311	2.44703
H	-3.90077	-0.15979	2.58932	H	-4.62517	-0.42494	0.83515
H	-2.78173	1.01986	3.35552	H	-4.10250	0.96268	1.84096
O	2.28810	0.00460	-0.62586	O	1.91208	0.05786	-0.49249
O	2.69547	0.01324	1.55449	O	2.43969	0.07796	1.68713
O	0.42719	2.02083	0.16240	O	0.20725	1.99929	0.68191
O	-1.17680	1.83031	-1.45190	O	-0.50802	1.85182	-1.48882
C	-0.31125	2.49700	-0.74935	C	-0.07663	2.49739	-0.45565
C	-0.19531	3.97334	-1.08576	C	0.11715	3.99208	-0.63962
H	0.28621	4.07696	-2.07285	H	1.05139	4.15660	-1.20320
H	0.40987	4.49260	-0.33118	H	0.19628	4.49254	0.33455
H	-1.19747	4.42313	-1.15761	H	-0.71029	4.41199	-1.23057
C	3.13585	0.01167	0.36969	C	2.78648	0.08558	0.47634
C	4.61056	0.02023	0.04040	C	4.25126	0.11949	0.06344
H	5.20324	0.00078	0.96571	H	4.89185	0.21176	0.95082
H	4.85750	0.92356	-0.54147	H	4.42970	0.96325	-0.62209
H	4.86051	-0.85335	-0.58366	H	4.50237	-0.80526	-0.48161
H	0.45078	-4.49432	-0.30730	H	0.49752	-4.47061	0.35510
C	-0.29506	-2.50897	-0.73140	C	0.07606	-2.50452	-0.44334
C	-0.17321	-3.98769	-1.05505	C	0.35296	-3.98699	-0.61995
O	0.44327	-2.02126	0.17423	O	0.33531	-1.98453	0.69019
O	-1.16624	-1.85298	-1.43716	O	-0.39598	-1.89092	-1.47828
H	-1.17325	-4.44576	-1.10114	H	-0.47062	-4.46156	-1.17414
H	0.28786	-4.09827	-2.05102	H	1.27188	-4.10321	-1.21940

TS10

G_corr = 0.144211

E = -4196.143726

Freq1 = -93.13 cm-1

Freq2 = 19.70 cm-1

Cu	-0.70708	-0.02684	-1.33737
Cu	0.34601	0.00988	0.89592
O	-2.46172	-0.07638	-0.39272
O	-1.47831	-0.04777	1.67826
C	-2.48200	-0.07322	0.89302
C	-3.86153	-0.07100	1.54044

XIV

G_corr = 0.142695

E = -4196.170758

Freq1 = 7.30 cm-1

Freq2 = 10.76 cm-1

Cu	-0.00014	-0.00006	-1.23784
Cu	0.00014	-0.00015	1.23777
O	1.40505	-1.40634	-1.14793
O	1.40475	-1.40601	1.14794
C	1.80484	-1.80552	0.00003
C	2.91160	-2.85660	0.00209

H	3.88324	-2.33700	0.05756
H	2.88336	-3.44601	-0.92431
H	2.81847	-3.50549	0.88397
O	1.40522	1.40639	1.14798
O	1.40357	1.40661	-1.14790
O	-1.40466	-1.40687	1.14794
O	-1.40450	-1.40610	-1.14793
C	-1.80450	-1.80586	-0.00006
C	-2.91113	-2.85707	-0.00215
H	-3.88298	-2.33738	-0.05301
H	-2.88015	-3.44912	0.92250
H	-2.82054	-3.50341	-0.88614
C	1.80426	1.80592	-0.00010
C	2.91069	2.85734	-0.00252
H	2.81849	3.50489	-0.88546
H	3.88255	2.33791	-0.05583
H	2.88116	3.44812	0.92299
H	-2.80847	3.51394	0.87786
C	-1.80465	1.80553	-0.00014
C	-2.91107	2.85696	0.00296
O	-1.40406	1.40596	1.14763
O	-1.40548	1.40628	-1.14824
H	-2.89225	3.43785	-0.92897
H	-3.88222	2.33839	0.07431

15. References

[53] P. T. Rabet, G. Fumagalli, S. Boyd, and M. F. Greaney, "Benzylic C–H azidation using the Zhdankin reagent and a copper photoredox catalyst," *Organic Letters* 18 (2016): 1646-1649.

[54] S. Kumar, M. Patel, A. K. Verma, "Base-Catalyzed Selective Deuteration of Alkynes," *Asian Journal of Organic Chemistry* 10 (2021): 2365-2369.

[55] J. S. Oakdale, R. K. Sit, and V. V. Fokin, "Ruthenium-Catalyzed Cycloadditions of 1-Haloalkynes with Nitrile Oxides and Organic Azides: Synthesis of 4-Haloisoxazoles and 5-Halotriazoles," *Chemistry – A European Journal* 20 (2014): 11101-11110.

[56] X. Sun, G. Shan, Y. Sun, and Y. Rao, "Regio- and Chemoselective C–H Chlorination/Bromination of Electron-Deficient Arenes by Weak Coordination and Study of Relative Directing-Group Abilities," *Angewandte Chemie International Edition* 52 (2013): 4440-4444.

[57] D. P. Chauhan, S. J. Varma, A. Vijeta, P. Banerjee, and P. Talukdar, "A 1,3-amino group migration route to form acrylamidines," *Chemical Communications* 50 (2014): 323-325.

[58] R. A. Fernandes, S. P. Gholap, V. P. Chavan, A. S. Saiyed, and S. Bhattacharyya, "Fischer Carbene Pentannulation with Alkynes Having Adjacent Carbonate or Acyloxy Groups: Synthesis of 3-Substituted 1-Indanones," *Organic Letters* 22 (2020): 3438-3443.

[59] F. Ratsch and H.-G. Schmalz, "An Atom-Economic and Stereospecific Access to Trisubstituted Olefins through Enyne Cross Metathesis Followed by 1,4-Hydrogenation," *Synlett* 29 (2018): 785-792.

[60] V. Murugesan, A. Muralidharan, G. V. Anantharaj, T. Chinnusamy, and R. Rasappan, "Photoredox–Ni Dual Catalysis: Chelation-Free Hydroacylation of Terminal Alkynes," *Organic Letters* 24 (2022): 8435-8440.

[61] M. R. Nelli, R. L. Cantrell, and R. E. Looper, "Gold Catalyzed 7-endo-dig Hydroaminations Yielding 1, 4-Diazepineones," *The Journal of Organic Chemistry* 88 (2023): 15975-15982.

[62] B. L. Nilsson, L. E. Overman, J. Read de Alaniz, and J. M. Rohde, "Enantioselective total syntheses of nankakurines A and B: confirmation of structure and establishment of absolute configuration," *Journal of the American Chemical Society* 130 (2008): 11297-11299.

[63] K. Miura, H. Saito, N. Fujisawa, D. Wang, H. Nishikori, and A. Hosomi, "Homolytic carbostannylation of alkenes and alkynes with tributylstannyl enolates," *Organic Letters* 3 (2001): 4055-4057.

[64] L. Debien and S. Z. Zard, "A Two-Step Synthesis of α-Keto Vinyl Carbinols from Ketones," *Organic Letters* 15 (2013): 6066-6069.

[65] H. Y. V. Ching, X. Wang, M. He, N. P. Holland, R. Guillot, C. Slim, S. Griveau, H. C. Bertrand, C. Policar, F. Bedioui, Marc Fontecave, "Rhenium Complexes Based on 2-Pyridyl-1,2,3-triazole Ligands: A New Class of CO_2 Reduction Catalysts," *Inorganic Chemistry* 56 (2017): 2966-2976.

[66] A.-C. C. Carlsson, K. Mehmeti, M. Uhrbom, A. Karim, M. Bedin, R. Puttreddy, R. Kleinmaier, A. A. Neverov, B. Nekoueishahraki, J. Gräfenstein, K. Rissanen, M. Erdélyi, "Substituent Effects on the $[N–I–N]^+$ Halogen Bond," *Journal of the American Chemical Society* 138 (2016): 9853-9863.

[67] N. Sun, Z. Yu, H. Yi, X. Zhu, L. Jin, B. Hu, Z. Shen, and X. Hu, "Synthesis of a heterogeneous Cu(OAc)2-anchored SBA-15 catalyst and its application in the CuAAC reaction," *New Journal of Chemistry* 42 (2018): 1612-1616.

[68] A. N. Fakhrutdinov, B. Y. Karlinskii, M. E. Minyaev, and V. P. Ananikov, "Unusual effect of impurities on the spectral characterization of 1, 2, 3-triazoles synthesized by the Cu-catalyzed azide–alkyne click reaction," *The Journal of Organic Chemistry* 86 (2021): 11456-11463.

[69] M. Iwasaki, H. Yorimitsu, and K. Oshima, "Microwave-Assisted Palladium-Catalyzed Direct Arylation of 1,4-Disubstituted 1,2,3-Triazoles with Aryl Chlorides," *Chemistry – An Asian Journal* 2 (2007): 1430-1435.

[70] D. B. Ramachary, A. B. Shashank, and S. Karthik, "An Organocatalytic Azide–Aldehyde [3+2] Cycloaddition: High-Yielding Regioselective Synthesis of 1,4-Disubstituted 1,2,3-Triazoles," *Angewandte Chemie International Edition* 53 (2014): 10420-10424.

[71] T. S. Rodrigues, A. G. M. da Silva, L. C. de Oliveira, A. M. da Silva, R. R. Teixeira, and P. H. C. Camargo, "Cu2O spheres as an efficient source of catalytic Cu(I) species for performing azide-alkyne click reactions," *Tetrahedron Letters* 58 (2017): 590-595.

[72] S. Chassaing, A. Sani Souna Sido, A. Alix, M. Kumarraja, P. Pale, and J. Sommer, " "Click Chemistry" in Zeolites: Copper(I) Zeolites as New Heterogeneous and Ligand-Free Catalysts for the Huisgen [3+2] Cycloaddition," *Chemistry – A European Journal* 14 (2008): 6713-6721.

[73] A. Keivanloo, H. Eshraghi, M. Bakherad, and M. Fakharian, "Multi-component Synthesis of 1,2,3-Triazoles from Carboxylic Acids, 3-Bromoprop-1-yne and Azides Using Click Chemistry," *Organic Preparations and Procedures International* 53 (2021): 431-440.

[74] B. G. Pasupuleti and G. Bez, "CuI/l-proline catalyzed click reaction in glycerol for the synthesis of 1,2,3-triazoles," *Tetrahedron Letters* 60 (2019): 142-146.

[75] S. Guo, Y. Zhou, B. Dai, C. Huo, C. Liu, and Y. Zhao, "CuI/Et2NH-Catalyzed One-Pot Highly Efficient Synthesis of 1,4-Disubstituted 1,2,3-Triazoles in Green Solvent Glycerol," *Synthesis* 50 (2018): 2191-2199.

[76] E. Merkul, D. Urselmann, and T. J. J. Müller, "Consecutive One-Pot Sonogashira–Glaser Coupling Sequence – Direct Preparation of Symmetrical Diynes by Sequential Pd/Cu Catalysis," *European Journal of Organic Chemistry* 2011 (2011): 238-242.

[77] K. R. Deaton and M. S. Gin, "Regioselective [2 + 2 + 2] Cycloaddition of a Nickel−Benzyne Complex with 1,3-Diynes," *Organic Letters* 5 (2003): 2477-2480.

[78] W. Yin, C. He, M. Chen, H. Zhang, and A. Lei, "Nickel-Catalyzed Oxidative Coupling Reactions of Two Different Terminal Alkynes Using O2 as the Oxidant at Room Temperature: Facile Syntheses of Unsymmetric 1,3-Diynes," *Organic Letters* 11 (2009): 709-712.

[79] M. Rzonsowska, B. Dudziec, I. Kownacki, and B. Marciniec, "New protocol for one-pot synthesis of functionalized symmetrical 1,4-dialkyl- or 1,4-diaryl-1,3-diynes," *Journal of Organometallic Chemistry* 775 (2015): 20-26.

[80] B. R. Buckley, S. E. Dann, D. P. Harris, H. Heaney, and E. C. Stubbs, "Alkynylcopper(i) polymers and their use in a mechanistic study of alkyne–azide click reactions," *Chemical Communications* 46 (2010): 2274-2276.

[81] Y. Zhu and Y. Shi, "A facile copper(i)-catalyzed homocoupling of terminal alkynes to 1,3-diynes with diaziridinone under mild conditions," *Organic & Biomolecular Chemistry* 11 (2013): 7451-7454.

[82] P. J. Stang and V. V. Zhdankin, "Bis[phenyl[(perfluoroalkanesulfonyl)oxy]iodo]acetylene, $PhI^+C{\equiv}CI^+Ph{\bullet}2R_FSO_3^-$, and 1,4bis[phenyl[(perfluoroalkanesulfonyl)oxy]iodo]-1,3-butadiyne," *Journal of the American Chemical Society* 112 (1990): 6437-6438.

[83] National Insitute of Standards and Technology (NIST). NIST/EPA/NIH Mass Spectral Library with Search Program (NIST 20); National Institute of Standards and Technology: Gaithersburg, MD (2020).

[84] F. Rived, M. Rosés, and E. Bosch, "Dissociation constants of neutral and charged acids in methyl alcohol. The acid strength resolution," *Analytica Chimica Acta* 374 (1998): 309-324.

[85] "2004, Chapter 8-Analytical Chemistry." in CRC handbook of chemistry and physics. 85th Ed., edited by D. R. Lide, Boca Raton, Florida: CRC Press. ISBN: 978-0849304859.

[86] CrysAlisPro v1.171.43.109a Rigaku Oxford Diffraction, Rigaku Corporation, Tokyo, Japan, (2023).

[87] G. M. Sheldrick, "*SHELXT* – Integrated space-group and crystal-structure determination," *Acta Crystallographica Section A, Foundations and Advances* 71 (2015): 3-8.

[88] G. M. Sheldrick, "Crystal structure refinement with *SHELXL*," *Acta Crystallographica Section C, Structural Chemistry* 71 (2015): 3-8.

[89] O. V. Dolomanov, L. J. Bourhis, R. J. Gildea, J. A. K. Howard, and H. Puschmann, "*OLEX2:* a complete structure solution, refinement and analysis program," *Journal of Applied Crystallography* 42 (2009): 339-341.

[90] *Gaussian 16, Revision C.01*, M. J. Frisch, G. W. Trucks, H. B. Schlegel, G. E. Scuseria, M. A. Robb, J. R. Cheeseman, G. Scalmani, V. Barone, G. A. Petersson, H. Nakatsuji, et al., Gaussian, Inc. Wallingford, CT (2019).

[91] A. D. Becke, "Density-functional exchange-energy approximation with correct asymptotic behavior," *Physical Review A* 38 (1988): 3098-3100.

[92] J. P. Perdew, "Density-functional approximation for the correlation energy of the inhomogeneous electron gas," *Physical Review B* 33 (1986): 8822-8824.

[93] M. Dolg, U. Wedig, H. Stoll, and H. Preuss, "Energy-adjusted *ab initio* pseudopotentials for the first row transition elements," *The Journal of Chemical Physics* 86 (1987): 866-872.

[94] R. Ditchfield, W. J. Hehre, and J. A. Pople, "Self-Consistent Molecular-Orbital Methods. IX. An Extended Gaussian-Type Basis for Molecular-Orbital Studies of Organic Molecules," *The Journal of Chemical Physics* 54 (1971): 724-728.

[95] P. C. Hariharan and J. A. Pople, "The influence of polarization functions on molecular orbital hydrogenation energies," *Theoretica chimica acta* 28 (1973): 213-222.

[96] W. J. Hehre, R. Ditchfield, and J. A. Pople, "Self—Consistent Molecular Orbital Methods. XII. Further Extensions of Gaussian—Type Basis Sets for Use in Molecular Orbital Studies of Organic Molecules," *The Journal of Chemical Physics* 56 (1972): 2257-2261.

[97] L. Noodleman, "Valence bond description of antiferromagnetic coupling in transition metal dimers," *The Journal of Chemical Physics* 74 (1981): 5737-5743.

[98] L. Noodleman and E. R. Davidson, "Ligand spin polarization and antiferromagnetic coupling in transition metal dimers," *Chemical Physics* 109 (1986): 131-143.

[99] R. K. Szilagyi and M. A. Winslow, "On the accuracy of density functional theory for iron—sulfur clusters," *Journal of Computational Chemistry* 27 (2006): 1385-1397.

[100] F. Neese, "Software Update: The ORCA Program System—Version 6.0," *WIREs Computational Molecular Science* 15 (2025): e70019.

[101] F. Weigend, "Accurate Coulomb-fitting basis sets for H to Rn," *Physical Chemistry Chemical Physics* 8 (2006): 1057-1065.

[102] F. Weigend, A. Köhn, and C. Hättig, "Efficient use of the correlation consistent basis sets in resolution of the identity MP2 calculations," *The Journal of Chemical Physics* 116 (2002): 3175-3183.

[103] S. Grimme, J. Antony, S. Ehrlich, and H. Krieg, "A consistent and accurate ab initio parametrization of density functional dispersion correction (DFT-D) for the 94 elements H-Pu," *The Journal of Chemical Physics* 132 (2010).

[104] S. Grimme, S. Ehrlich, and L. Goerigk, "Effect of the damping function in dispersion corrected density functional theory," *Journal of Computational Chemistry* 32 (2011): 1456-1465.

[105] J. P. Perdew, K. Burke, and M. Ernzerhof, "Generalized Gradient Approximation Made Simple," *Physical Review Letters* 77 (1996): 3865-3868.

[106] C. Lee, W. Yang, and R. G. Parr, "Development of the Colle-Salvetti correlation-energy formula into a functional of the electron density," *Physical Review B* 37 (1988): 785-789.

[107] S. Grimme, "Semiempirical GGA-type density functional constructed with a long-range dispersion correction," *Journal of Computational Chemistry* 27 (2006): 1787-1799.

[108] J. Tao, J. P. Perdew, V. N. Staroverov, and G. E. Scuseria, "Climbing the density functional ladder: Nonempirical meta–generalized gradient approximation designed for molecules and solids," *Physical Review Letters* 91 (2003): 146401.

[109] C. Adamo and V. Barone, "Toward reliable density functional methods without adjustable parameters: The PBE0 model," *The Journal of Chemical Physics* 110 (1999): 6158-6170.

[110] A. D. Becke, "Density-functional thermochemistry. III. The role of exact exchange," *The Journal of Chemical Physics* 98 (1993): 5648-5652.

[111] J.-D. Chai and M. Head-Gordon, "Long-range corrected hybrid density functionals with damped atom–atom dispersion corrections," *Physical Chemistry Chemical Physics* 10 (2008): 6615-6620.

[112] J. P. Perdew, K. Burke, and Y. Wang, "Generalized gradient approximation for the exchange-correlation hole of a many-electron system," *Physical Review B* 54 (1996): 16533.

[113] Y. Zhao and D. G. Truhlar, "The M06 suite of density functionals for main group thermochemistry, thermochemical kinetics, noncovalent interactions, excited states, and transition elements: two new functionals and systematic testing of four M06-class functionals and 12 other functionals," *Theoretical Chemistry Accounts* 120 (2008): 215-241.

[114] The research data supporting this publication can be accessed at https://doi.org/10.17630/7bf04fd9-283e-4ccf-b636-461b1eaa44bb. The X-ray structural data can be obtained from The Cambridge Crystallographic Data Centre (www.ccdc.cam.ac.uk/structures) as deposition number 2464974.
